# Revised classification and catalogue of global Nepticulidae and Opostegidae (Lepidoptera, Nepticuloidea)

**DOI:** 10.3897/zookeys.628.9799

**Published:** 2016-10-31

**Authors:** Erik J. van Nieukerken, Camiel Doorenweerd, Robert J. B. Hoare, Donald R. Davis

**Affiliations:** 1 Naturalis Biodiversity Center, PO Box 9517, NL-2300 RA Leiden, The Netherlands; 2 Landcare Research Ltd., Private Bag 92–170, Auckland, New Zealand; 3 Department of Entomology, National Museum of Natural History, MRC 105, Smithsonian Institution, PO Box 37012, Washington, DC 20013–7012, USA

**Keywords:** Taxonomy, leaf miners, checklist, history, new synonyms, new combinations

## Abstract

A catalogue of all named Nepticulidae and Opostegidae is presented, including fossil species. The catalogue is simultaneously published online in the scratchpad http://nepticuloidea.info/ and in Catalogue of Life (http://www.catalogueoflife.org/col/details/database/id/172). We provide a historical overview of taxonomic research on Nepticuloidea and a brief ‘state of the art’. A DNA barcode dataset with 3205 barcodes is made public at the same time, providing DNA barcodes of ca. 779 species, of which 2563 are identified as belonging to 444 validly published species. We recognise 862 extant and 18 fossil species of Nepticulidae in 22 extant genera and the fossil form genus *Stigmellites*. We count 192 valid Opostegidae species in 7 genera, without fossils. We also list seven dubious Nepticulidae names that cannot be placed due to absent type material and poor descriptions, 18 unavailable names in Nepticulidae that cannot be placed and we also list the 33 names (including four fossils) that once were placed as Nepticulidae or Opostegidae but are now excluded. All synonyms and previous combinations are listed. The generic classification follows the Molecular phylogeny that is published almost simultaneously. Subfamilies and tribes are not recognised, Trifurculinae Scoble, 1983 is synonymised with Nepticulidae Stainton, 1854 and Opostegoidinae Kozlov, 1987 is synonymised with Opostegidae Meyrick, 1893. The status of *Casanovula* Hoare, 2013, *Etainia* Beirne, 1945, *Fomoria* Beirne, 1945, *Glaucolepis* Braun, 1917, *Menurella* Hoare, 2013, *Muhabbetana* Koçak & Kemal, 2007 and *Zimmermannia* Hering, 1940 is changed from subgenus to full genus, whereas two genera are considered synonyms again: *Manoneura* Davis, 1979, a synonym of *Enteucha* Meyrick, 1915 and *Levarchama* Beirne, 1945, a synonym of *Trifurcula* Zeller, 1848. We propose 87 new combinations in Nepticulidae and 10 in Opostegidae, largely due to the new classification, and re-examination of some species. We propose the following 37 new synonymies for species (35 in Nepticulidae, 2 in Opostegidae):

*Stigmella acerifoliella* Dovnar-Zapolski, 1969 (unavailable, = *Stigmella acerna* Puplesis, 1988), *Stigmella nakamurai* Kemperman & Wilkinson, 1985 (= *Stigmella palionisi* Puplesis, 1984), *Nepticula amseli* Skala, 1941 (unavailable = *Stigmella birgittae* Gustafsson, 1985), *Stigmella cathepostis* Kemperman & Wilkinson, 1985 (= *Stigmella microtheriella* (Stainton, 1854)), *Stigmella populnea* Kemperman & Wilkinson, 1985 (= *Stigmella nivenburgensis* (Preissecker, 1942)), *Nepticula obscurella* Braun, 1912 (revised synonymy, = *Stigmella myricafoliella* (Busck, 1900)), *Nepticula mandingella* Gustafsson, 1972 (= *Stigmella wollofella* (Gustafsson, 1972)), *Stigmella rosaefoliella pectocatena* Wilkinson & Scoble, 1979 (= *Stigmella centifoliella* (Zeller, 1848)), *Micropteryx pomivorella* Packard, 1870 (= *Stigmella oxyacanthella* (Stainton, 1854)), *Stigmella crataegivora* Puplesis, 1985 (= *Stigmella micromelis* Puplesis, 1985), *Stigmella scinanella* Wilkinson & Scoble, 1979 (= *Stigmella purpuratella* (Braun, 1917)), *Stigmella palmatae* Puplesis, 1984 (= *Stigmella filipendulae* (Wocke, 1871)), *Stigmella sesplicata* Kemperman & Wilkinson, 1985 (= *Stigmella lediella* (Schleich, 1867)), *Stigmella rhododendrifolia* Dovnar-Zapolski & Tomilova, 1978 (unavailable, = *Stigmella lediella* (Schleich, 1867)), *Stigmella oa* Kemperman & Wilkinson, 1985 (= *Stigmella spiculifera* Kemperman & Wilkinson, 1985), *Stigmella gracilipae* Hirano, 2014 (= *Stigmella monticulella* Puplesis, 1984), *Nepticula chaoniella* Herrich-Schäffer, 1863 (= *Stigmella samiatella* (Zeller, 1839)), *Bohemannia piotra* Puplesis, 1984 (= *Bohemannia pulverosella* (Stainton, 1849)), *Bohemannia nipponicella* Hirano, 2010 (= *Bohemannia manschurella* Puplesis, 1984), *Sinopticula sinica* Yang, 1989 (= *Glaucolepis oishiella* (Matsumura, 1931)), *Trifurcula collinella* Nel, 2012 (= *Glaucolepis magna* (A. Laštuvka & Z. Laštuvka, 1997)), *Obrussa tigrinella* Puplesis, 1985 (= *Etainia trifasciata* (Matsumura, 1931)), *Microcalyptris vittatus* Puplesis, 1984 and *Microcalyptris arenosus* Falkovitsh, 1986 (both = *Acalyptris falkovitshi* (Puplesis, 1984)), *Ectoedemia castaneae* Busck, 1913, *Ectoedemia heinrichi* Busck, 1914 and *Ectoedemia helenella* Wilkinson, 1981 (all three = *Zimmermannia bosquella* (Chambers, 1878)), *Ectoedemia chloranthis* Meyrick, 1928 and *Ectoedemia acanthella* Wilkinson & Newton, 1981 (both = *Zimmermannia grandisella* (Chambers, 1880)), *Ectoedemia coruscella* Wilkinson, 1981 (= *Zimmermannia mesoloba* (Davis, 1978)), *Ectoedemia piperella* Wilkinson & Newton, 1981 and *Ectoedemia reneella* Wilkinson, 1981 (both = *Zimmermannia obrutella* (Zeller, 1873)), *Ectoedemia similigena* Puplesis, 1994 (= *Ectoedemia turbidella* (Zeller, 1848)), *Ectoedemia andrella* Wilkinson, 1981 (= *Ectoedemia ulmella* (Braun, 1912)), *Nepticula canadensis* Braun, 1917 (= *Ectoedemia minimella* (Zetterstedt, 1839)), *Opostega rezniki* Kozlov, 1985 (= *Opostega cretatella* Chrétien, 1915), *Pseudopostega cyrneochalcopepla* Nel & Varenne, 2012 (= *Pseudopostega chalcopepla* (Walsingham, 1908)). *Stigmella caryaefoliella* (Clemens, 1861) and *Zimmermannia bosquella* (Chambers, 1878) are taken out of synonymy and re-instated as full species. Lectotypes are designated for *Trifurcula obrutella* Zeller, 1873 and *Nepticula grandisella* Chambers, 1880.

## Introduction

Names of organisms are the key to the biological literature, but even in our digital age it is often a challenge to find and apply the correct names. Taxonomic internet databases, spawned from global projects such as GBIF (Gbif Secretariat 2016) and Species 2000 (Catalogue of Life: Roskov et al. 2015) are growing in number and are impressively linked together, but in many cases lack informative and reliable content. Catalogue of Life is especially poor for Lepidoptera, being based on an online version of the Cardindex of the Natural History Museum in London (LepIndex: Beccaloni et al. 2005). This is a wonderful resource for taxonomists, but not an authoritative catalogue with modern classification and names. The plans for an update depend on external funding and may never materialize with the current taxonomic funding climate (I. Kitching personal communication).

Any online only publication of a catalogue has the disadvantage that nomenclatorial changes are unavailable and still need to be published in a unchangeable format on paper or as pdf file following the amendments made to the Code (International Commission on Zoological Nomenclature 2012). It is understandable that many taxonomists prefer paper publications, evident from several recent catalogues for Lepidoptera (Pterophoridae, Coleophoridae, Psychidae, Notodontidae, Yponomeutoidea) that cannot yet be found in an online database (Gielis 2003; Baldizzone et al. 2006; Sobczyk 2011; Schintlmeister 2013; Lewis and Sohn 2015). Fortunately other authors followed a printed catalogue soon with an online database version, e.g. for Gracillariidae (De Prins and De Prins 2005; De Prins and De Prins 2016) and Tortricidae (Brown et al. 2003; Gilligan et al. 2014) and for other groups there are online-only catalogues that are actively maintained, e.g. Pyraloidea (Nuss et al. 2003–2015) and part of the butterflies (Häuser et al. 2012). Still, for the largest part of Lepidoptera there are still no global catalogues available. With our contribution we hope to fill a very small gap of the megadiverse order Lepidoptera.

For some years we have been preparing an online catalogue of Nepticuloidea in a so-called scratchpad (van Nieukerken 2016), which has several advantages, but also the cited disadvantage and the lack of an easy overview. The most recent Nepticuloidea catalogue was published in a book (Diškus and Puplesis 2003), using a somewhat different generic classification. Our phylogenetic study, to be published simultaneously with this catalogue (Doorenweerd et al. 2016a), resulted in the need for a new classification, which has consequences for the names of many taxa. We therefore decided to publish this static catalogue to fix the state of the art, make the necessary nomenclatorial changes, whereas at the same time the Nepticuloidea scratchpad version 2.0 (http://nepticuloidea.info/) is released with additional information and illustrations, and will be updated continuously. We are also happy to announce that at the same time this catalogue is made available to the Catalogue of Life (http://www.catalogueoflife.org/col/details/database/id/172) and GBIF. This catalogue is not only the taxonomic summary of the published knowledge on these families, it also contains many original data, such as new synonymies and taxonomic placement of species, based on our many years of research of these insects worldwide; some of these results are formalised here, but will be detailed elsewhere. In the material and methods section we discuss our various choices for this catalogue, such as species concepts, use of DNA barcodes, and nomenclatorial issues.

To place the list in a broader context we give an account of the taxonomic history and history of research on Nepticulidae and Opostegidae. We begin the results section with a review of the “state of the art” of Nepticuloidea in the various biogeographic regions.

### History of taxonomic research on Nepticulidae

The first species of Nepticulidae that was described, was *Stigmella anomalella* (Goeze, 1783) of which Degeer (1752) described and illustrated the larva in detail, while using the term “miner” (“mineuse”) for the first time (van Nieukerken 2008). Later, Degeer (1771) described the adult that he reared from these mines. Degeer’s descriptions are still interesting reading these days, and were fully copied and translated by Stainton et al. (1855). Since Degeer did not use binominal nomenclature in these works, the species was only validly named later by Goeze (1783) as *Phalaena anomalella*. This makes *Ectoedemia occultella* (Linnaeus, 1767) the first formally named nepticulid (as Phalaena (Tinea) occultella), although Linnaeus himself had yet no notion of the life history of the small moth that he found on his windows (Robinson and Nielsen 1983). Five more names were given to Nepticulidae in the 18^th^ century, all named in *Tinea*, but just two of these are still considered valid: *Stigmella aurella* and *Stigmella hybnerella*. Even though Schrank already in 1802 gave the generic name *Stigmella* to his rose leafminer *Tinea rosella* (a junior synonym of *Stigmella anomalella*), this was overlooked by most 19^th^ century authors, and in the first decades of the 19^th^ century, species were still placed in *Tinea* (e.g. by Haworth 1828), the first author to describe a number of species from the London area, nine in total) or in a number of other “tineid” genera such as *Microsetia* (Stephens 1834; Bedell 1848), *Oecophora* and *Elachista* (Kollar 1832; Zetterstedt 1838–1840; Duponchel [1842–1845]) and *Caloptilia* (Hübner 1816–1826), now all belonging in different families, but together these authors named only about ten species of Nepticulidae.

**Figures 1–9.**
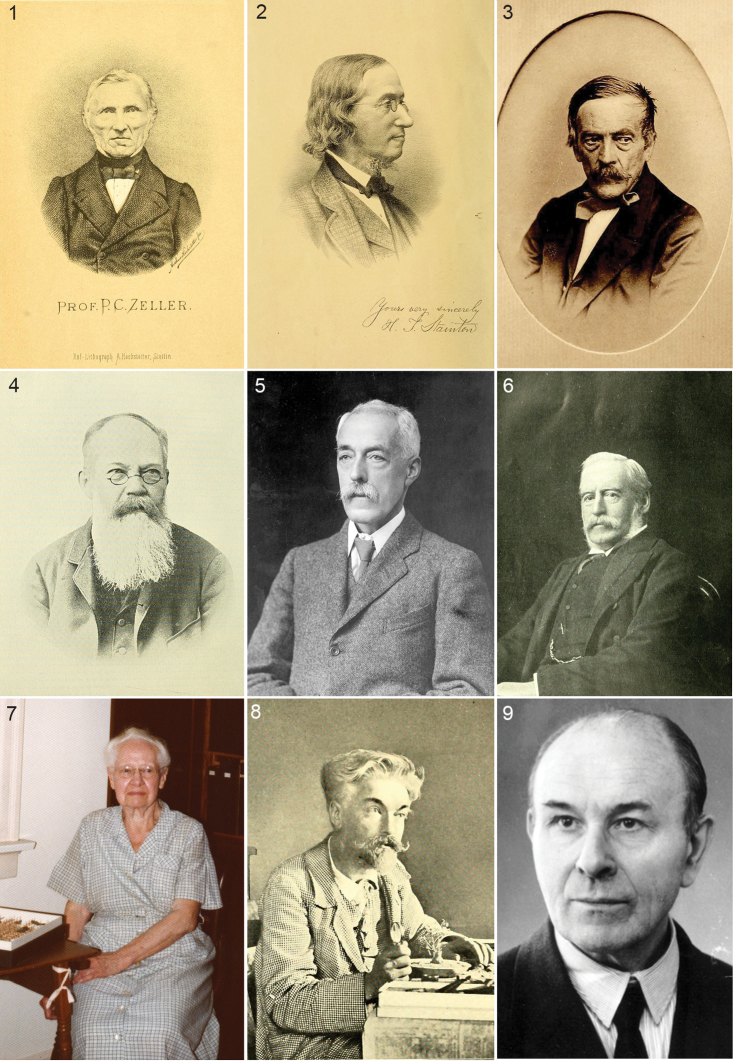
Portraits of Lepidopterists who described Nepticuloidea. **1** Philipp Christoph Zeller (Stainton 1883) **2** Henry Tibats Stainton from Douglas and Mclachlan (1893) **3** Gottlieb August Wilhelm Herrich-Schäffer, ca 1870 (Kraatz 1875) **4** Maximilian Ferdinand Wocke (Dittrich 1907) **5** Edward Meyrick (Clarke 1955) **6** Lord Walsingham (Durrant 1920) **7** Annette Francis Braun, in 1973 in her home in Cincinnati, Ohio (photo Mignon Davis) **8** Pierre Chrétien, Digne, May 1903 (Oberthür 1915) **9** Erich Martin Hering. Figs 1–6 and 8 from Biodiversity Heritage Library, 9 from Zobodat.

Towards the second half of the 19^th^ century, things changed rapidly, in Europe attributable to four naturalists: in Germany Philipp Christoph Zeller (1808–1883) (Fig. 1) and Gottlieb August Wilhelm Herrich-Schäffer (1799–1874, Regensburg) (Fig. 3), in Great Britain Henry Tibbats Stainton (1822–1892) (Fig. 2) and in Switzerland Heinrich Frey (1822–1890). All four were lepidopterists with a broad interest, studying not only all Lepidoptera from their own country, but also from many exotic countries.

The genus *Nepticula* was formally erected in a meeting report by von Heyden (1843), but he did not publish much on this genus, and it was Zeller (1848) who made a detailed description of the genus and several species. Some years earlier Zeller (1839) had described several Nepticulidae (in his “Versuch einer naturgemässen Eintheilung der Schaben”) together with other leafminers in the genus *Lyonetia*, subgenus Bucculatrix, in which he placed ten nepticulid species together with six that we still consider as *Bucculatrix* (Bucculatricidae). All these descriptions were based on adults that were collected in the field without knowledge of the life history (except DeGeer’s work!). Stainton and his British followers drastically changed this situation, by eagerly rearing leafminers and many other microlepidoptera, resulting in a proliferation of new species discoveries. Following a few initial species descriptions, his book “Insecta Brittanica. Lepidoptera: Tineina” (Stainton 1854) contained the first extensive treatment of Nepticulidae – at that point recognised as a family with two genera (*Nepticula* and *Trifurcula*) – with detailed information on leafmines and biology of 32 species in total (see Table 1). Stainton continued publishing discoveries the next decades, partly using his own periodicals “The Entomologist’s Weekly Intelligencer” and “The Entomologist’s Annual”, and later in the still running journal “Entomologist’s Monthly Magazine” that he founded with entomologist friends (Emmet 1992). His magnum opus is the 13 volume work “The natural history of the Tineina”, that he wrote collaboratively with Zeller, Frey and J.W. Douglas. Nepticulidae are treated in volumes 1 and 7 (Stainton et al. 1855; Stainton et al. 1862). These books are best known for the exquisite hand coloured plates with details of all life stages of many Microlepidoptera. During his life Stainton described 39 species of Nepticulidae, of which currently 30 are still considered valid names. With that record he is the most productive 19^th^ century nepticulid taxonomist.

**Table 1.** Number of Nepticulidae species (valid and invalid) described per first author for authors who described at least ten valid species, including extant and fossil species.

First author	Valid spp	Invalid spp	Total
Puplesis (Stonis)	176	22	198
Scoble	73	1	74
Meyrick	67	6	73
van Nieukerken	55		55
Klimesch	38	10	48
Stainton	30	9	39
Braun	26	11	37
Remeikis	24		24
Kemperman	23	13	36
Laštuvka, A.	21		21
Vári	20		20
Laštuvka, Z.	17		17
Diškus	15		15
Donner	14		14
Clemens	13	7	20
Zeller	12	5	17
Chambers	12	12	24
Hoare	12		12
Herrich-Schäffer	11	10	21
Frey	10	8	18
Hirano	10	2	12
96 authors	201		
**Total**	**880**		

Soon after Stainton’s Insecta Britannica, Frey wrote two books dealing with respectively Swiss and European Nepticulidae (Frey 1856; 1857), that he also partly had reared himself. In the same period, Herrich-Schäffer’s large multivolume work “Systematische Bearbeitung der Schmetterlinge von Europa” (1843–1855) was concluded by volumes 5 and 6, dealing with Nepticulidae in volume 5, heft 67 (Herrich-Schäffer 1855a) (the book was issued in parts, the plates often earlier than the text, and the bibliography is very complicated, see Hemming 1937). He apparently did not rear nepticulids himself, but included information on rearing from others, such as Frey and Heyden. He also named several species that Frey intended to name (as he did later, in 1856), but changed the endings of the names deliberately into “–ella”. This explains pairs of names such as: *Nepticula aeneofasciella* Herrich-Schäffer, 1855 and *Nepticula aeneofasciata* Frey, 1856. The practice to use consistent endings for larger groups of Lepidoptera dated from Linnaeus, who used –ella for what he called *Tinea*, -ana for *Tortrix*, -alis for *Pyralis* etc. (Emmet 1991). In the 19^th^ century there were no hard nomenclatorial rules yet, and specialists often changed names deliberately following this practice, see e.g. also several listed synonyms (incorrect subsequent spellings) by Doubleday (1859). Herrich-Schäffer named several nepticulids hidden in catalogues and a report on a collecting trip to Engadin (Herrich-Schäffer 1863b; c), names that have completely been forgotten and were never listed in catalogues, but only cited in two other papers (Snellen 1873; Segerer 1997). As far as these names are valid, they can be considered nomina oblita once their identities have been established, since they were never used as valid names since 1899 (ICZN art. 23.9.1.1), and do not compete with junior synonyms (reversal of precedence, ICZN art. 23).

By 1860 the number of species (all from Europe) had been tripled since 1848: from 24 to 77 (see Fig. 24B). In Europe the work on describing Nepticulidae continued both in Britain and in Central Europe, particularly with Herman von Heinemann (1812–1871) in Braunschweig (Heinemann 1861; 1862a; b) and Maximilian Ferdinand Wocke (1820–1906) (Fig. 4) in Breslau, who contributed many shorter papers (Wocke 1862; 1865; 1877). Jointly they wrote a larger fauna: Die Schmetterlinge Deutschlands und der Schweiz, partly finished after Heinemann’s death (Heinemann and Wocke [1876]). After *ca* 1880 taxonomic work on Nepticulidae in Europe slowed down considerably.

Meanwhile, in the United States, James Brackenridge Clemens (1825–1867) in Easton, Pennsylvania, had started studies on microlepidoptera. He collected the leafmines actively and described them in a few papers, but in contrast to Stainton he did not wait for successful rearing results and described many new species solely on the basis of leafmines (Clemens 1861; 1862a; Clemens 1862b; Clemens 1865), often resulting in a conundrum for future taxonomists. There are no types of the mines left, and more than once multiple species are known to feed on the host plant from which he described a single species. Such species have to be interpreted carefully and sometimes Neotypes need to be selected (Busck 1903; Wilkinson and Scoble 1979). A second North American pioneer was Vactor Tousey Chambers (1830–1883) from Covington, Kentucky, who added more species on the basis of mines and larvae, but also many on the basis of adults (Chambers 1873; 1875b; 1878a). There are more types left of his species (in the Museum of Comparative Zoology, Cambridge, Mass.), but also many missing and reconstructing identities can still be cumbersome (Busck 1903; Miller and Hodges 1990). Here we resolve three of his names.

The first Nepticulidae described from other regions than Europe or the USA, were two species described from Colombia by Zeller (1877), although Francis Walker’s (Walker 1864) *Stigmella maoriella* from New Zealand already predated these. However, Walker, often criticised for his numerous useless descriptions, was totally unaware he was naming a nepticulid and placed it in *Tinea*.

In the late 19^th^ century Edward Meyrick (1854–1939) (Fig. 5) started his long career of describing numerous exotic Microlepidoptera, totalling approximately 20,000 species, including 68 Nepticulidae and 54 Opostegidae. The first Nepticulidae he described were three species from New Zealand (Meyrick 1889) where he lived in 1882–1883 (Hudson 1938). Up to 1935 Meyrick described many Nepticulidae from southern Africa, India, Australia, New Zealand and South America. His descriptions are typically very short, without illustrations, and only some of the species were reared (by other collectors who sent the specimens to him). When studying genitalia became more common practice, Meyrick refused to see the necessity and continued in a similar manner, relying almost completely on venation and colour pattern (Clarke 1955). As a result almost none of the species he described can be recognised from the description alone and the study of types is essential. Fortunately, most of those are kept in the Natural History Museum in London, and some in other collections, including the Ditsong Museum of Natural History in Pretoria. Those types that still exist have been studied by Malcolm Scoble, Erik van Nieukerken, Robert Hoare and co-authors and Rimantas Puplesis (now Jonas Stonis). Meyrick named only two nepticulid genera: *Acalyptris* and *Enteucha*, the latter he placed in Opostegidae (Davis 1985).

Quite a different approach was followed in the early 20^th^ century in North America, where August Busck (1870–1944), a Danish immigrant, started his studies at the Division of Entomology of the U.S. Department of Agriculture and somewhat later Annette Frances Braun (1884–1978) (Fig. 7), who became the leading microlepidopterists of North America. Both made many careful descriptions of often reared species, but most of these before genitalia studies became fashionable (Busck 1900; 1913; 1914a; Braun 1912; 1914; 1917; 1923; 1925b).

In Europe, in addition to *Nepticula*, only two additional small genera were recognised on the basis of the venation: *Trifurcula* Zeller, 1848 and *Bohemannia* Stainton, 1859 (Zeller 1848; Stainton 1859a). Although many treatments of the family were published in Europe, the classification did not change for almost a century (Heinemann 1862a; b; Frey 1880; Snellen 1882; Meyrick 1895; Tutt 1899; Spuler and Meess 1910; Heinemann and Wocke [1876]). Walsingham (1908a) had shown that the senior name *Stigmella* Schrank, 1802 should be used instead of *Nepticula*, but few authors followed him until this case was settled by Wilkinson (1978) in favour of the senior name *Stigmella*. Between them, Busck and Braun erected four more genera on the basis of venation: *Ectoedemia* Busck, 1907 (also based on the galling habit), *Obrussa* Braun, 1915, *Glaucolepis* Braun, 1917 and *Microcalyptris* Braun, 1925 (Busck 1907; Braun 1915; 1917; 1925b) (see Table 2). It is strange that these authors who relied so much on venation in describing genera, did not scrutinize the many *Nepticula* species, amongst which were still many species with venation different from *Stigmella*, and more similar to their new genera. Also the name *Trifurcula* was several times misapplied to species with completely different venations, now belonging in *Zimmermannia* (*atrifrontella* Stainton, *obrutella* Zeller), *Bohemannia* (*pulverosella* Stainton) or *Acalyptris* (*minimella* Rebel).

**Table 2.** Number of genera of Nepticuloidea named per author, including replacement names.

Author	# Valid genera	# Invalid genera
Davis (1 with Stonis)	4	3
Scoble	4	1
Hoare	3	
van Nieukerken	3	1
Beirne	2	3
Kozlov	2	
Meyrick	2	
Zeller	2	
Borkowski	1	1
Braun	1	2
Busck	1	
Hering	1	
Koçak	1	1
Schrank	1	
Stainton	1	
Müller-Rutz		2
Börner		1
Heyden		1
Puplesis		1
Strand		1
Yang		1

In Europe, during the first decades of the 20^th^ century, some more southern areas were explored by Lord Walsingham (Thomas de Grey, 6th Baron Walsingham, 1843–1919) (Fig. 6), who collected in France, Spain, the Canary Islands, Morocco and Algeria (Walsingham 1891; 1904; 1908a; b; 1911) and reared several of these species from their mines. Also Pierre Chrétien (1846–1934) (Fig. 8) reared several species from hitherto unexpected host plants in France and Algeria (*Bupleurum* in Apiaceae, *Linum* in Linaceae, *Launaea* [as *Zollikofferia*] in Asteraceae); these species are now all placed in *Glaucolepis* (Chrétien 1904; 1907; 1914).

**Figures 10–16.**
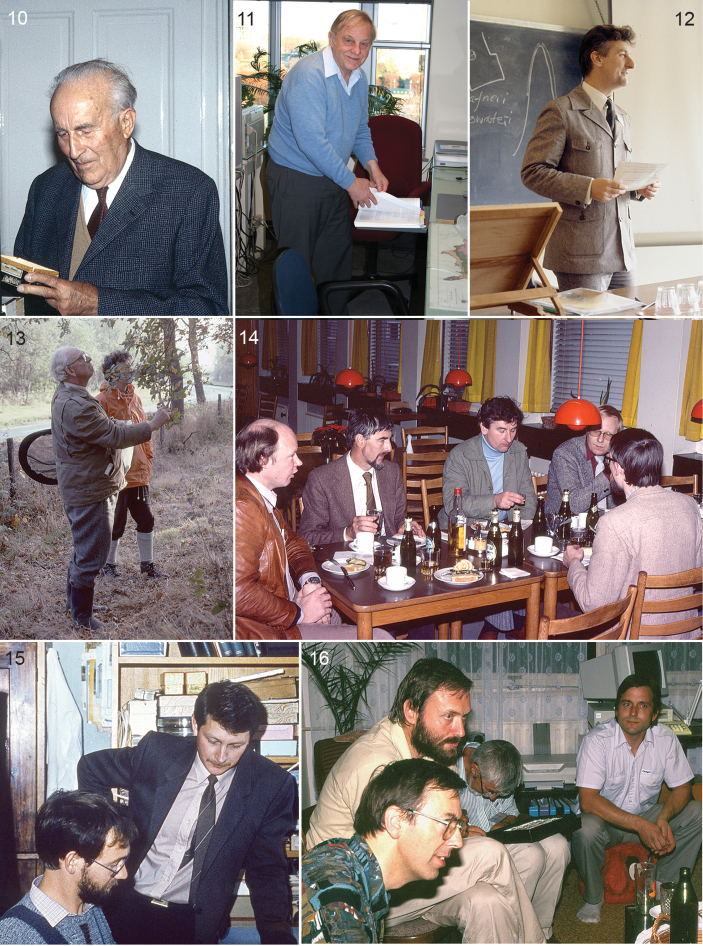
Specialists on Nepticuloidea in 20^th^ century. **10** Joseph Klimesch, in his house, Linz, October 1983 **11** Donald R. Davis in his office, December 2006, USNM, Washington DC **12** Christopher Wilkinson, April 1982, Cambridge, European Congress of Lepidopterology **13** Arthur Maitland Emmet, with C Wilkinson, Essex, UK, October 1979 **14** Meeting for preparation of the Nepticulidae volumes for Fauna Entomologica Scandinavica, Copenhagen, October 1980: from left: Ebbe S. Nielsen, Roland Johansson, Chris Wilkinson, Bert Gustafsson, Niels Peder Kristensen **15** Rimantas Puplesis, standing (now Jonas R. Stonis), Erik J. van Nieukerken, in Zoological Institute, Leningrad, September 1985 **16** Get together of leafminer enthusiasts at house of Aleš Laštůvka, Prostejov, Czech Rep., September 1994, from left: Steven Whitebread, Zdeněk Laštůvka, Roland Johansson, Aleš Laštůvka. Scans from transparencies by Erik J. van Nieukerken.

In the 20th century the study of genitalia gradually became standard in Lepidoptera and the first study on nepticulids was by Petersen (1930b), who divided *Nepticula* into a number of species groups based on the male genitalia. The first following him was Hering (Hering 1942; 1943), but his first genitalia drawings were published by Klimesch (Klimesch 1940a; b; c; d), who in the last of these papers also shows his first own genitalia drawing (of *Acalyptris platani*). The single contribution by Bryan P. Beirne (1918–1998) was particularly important. He finished the British series of Lepidoptera genitalia started by Pierce and Metcalfe (1935) – who excluded most Nepticulidae, apart from *Trifurcula*, in their ‘tineid’ volume, referring to Petersen’s work – , and described the male genitalia of the British species (Beirne 1945). While doing so he also erected five new genera with names based on Irish mythology (*Dechtiria*, *Levarchama*, *Fedalmia*, *Etainia* and *Fomoria*) and split *Stigmella* into *Stigmella* and *Nepticula*. From then on describing a new species without illustrating the male genitalia seldom occurred anymore, and the late 1940’s showed several examples of this (Doets 1947; Janse 1948; Klimesch 1948a; b; Hartig 1949; Ford 1950a). Josef Wilhelm Klimesch (1902–1997) (Fig. 10) was one of the most productive authors of this period, and again in the 1970’s after his retirement (Gusenleitner 1988; Deschka 1998; Aspöck 2003). He not only carefully illustrated the genitalia, he also paid considerable attention to the leafmines and biology, with detailed illustrations and descriptions. Possibly because of the greater demands that including genitalia imposed on authors, the descriptions of new species decreased considerably during the 1950’s and 1960’s, only to rise again from 1978 on. Between 1950 and 1978 only 60 new species were named, most by Josef Klimesch in Europe and Lajos Vári in South Africa (Klimesch 1951b; 1953a; b; 1975c; Vári 1955; 1963).

Quite a reverse development occurred in the first half of the 20^th^ century that involved an increased interest in the mine form and the host plant, especially promoted by Erich Martin Hering (1893–1967) (Fig. 9), an entomologist in Berlin. Although he was also a very active and experienced taxonomist in Lepidoptera and Diptera, who introduced the use of genitalia characters, he also gave often undue weight to characters of leafmines, for example in the *Stigmella ulmivora* group, where he recognised three species on the basis of leafmine form and frass pattern, that are in fact ecological forms of one species. However, with his many publications, Hering’s work was an outstanding contribution to the study of leafminers, and his identification keys are still a much used reference (Hering 1957), as is his general book on the biology of leafminers (Hering 1951). Also Victor Hugo Otto Skala (1875–1952) studied leafminers intensively, but he also named many species on the basis of the mines only (which are unavailable after 1930, ICZN art. 13.6.2), provided with very poor and minute illustrations and brief descriptions (example: Skala 1939a; b; c; d; e; f; g; h), and only few of the names he introduced, and based on adults, remain valid today (*Ectoedemia klimeschi* (Skala, 1933), *Fomoria groschkei* (Skala, 1943)).

During the 1970’s the study of nepticulids suddenly received considerable increase in support from several sources, following a general trend for greater investment in taxonomy and science at large and an increase of amateur interest. In Sweden amateur Roland Johansson (Figs 14, 16) revised the confusing oak feeding *Stigmella ruficapitella* group (Johansson 1971). In the same paper he provided an updated checklist of Scandinavian and British species, within a new framework of two genera: *Nepticula* and *Trifurcula*, the latter embracing all of Beirne’s genera. He also introduced the use of species groups. At about the same time Alfred Borkowksi wrote a number of papers on the faunistics and taxonomy of Nepticulidae in Poland (Borkowski 1969; 1970a; b; 1972a; b). In one of these, he introduced a new generic classification, following partly Beirne (1945), using male genitalia, and combined this with the study of venation (Borkowski 1972a). In Britain Arthur Maitland Emmet (1908–2001) (Harley 2001) (Fig. 13) started to work on this group and many other Microlepidoptera for the new series “The Moths and Butterflies of Great Britain and Ireland” (MBGBI). He followed upon Johansson’s and Borkowski’s work and treated the Nepticulidae in the first volume (Emmet 1976), but his many shorter and longer papers in The Entomologist’s Record, often made in preparation of the book, illustrated his in-depth knowledge, and are also enjoyable reading (for example: Emmet 1973a; b; c; 1974a; d; e; f; g). Also in the 1970’s, Josef Klimesch, now retired, started working on the material he collected in southern Europe and the Canary Islands, and described a number of new species from that area (Klimesch 1975c; 1977; 1978b).

**Figures 17–23.**
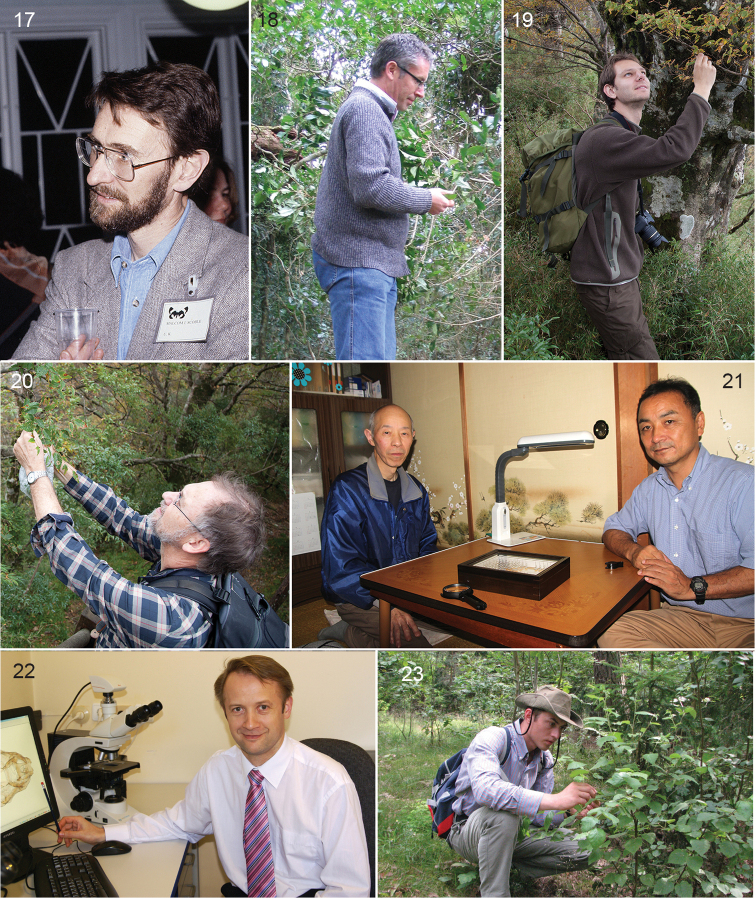
Specialists on Nepticuloidea in 20^th^ and 21^st^ century. **17** Malcolm Scoble, April 1992, Helsinki, Congress SEL **18** Robert J. B. Hoare, collecting *Menurella quintiniae* at type locality, Lamington Park, Queensland, August 2004 **19, 20** Camiel Doorenweerd and Erik J. van Nieukerken collecting leafminers on *Fagus hayatae*, Taiwan, Taipingshan, October 2012 **21** Nagao Hirano and Toshiya Hirowatari, at Hirano’s house, Matsumoto, Japan, September 2014 **22** Arūnas Diškus **23** Andrius Remeikis. Photos by Erik J. van Nieukerken (17, 18, 21) and Shipher Wu (19, 20).

Before 1970, most work on Nepticulidae was carried out independently by either amateur entomologists or isolated professional biologists, but during the 1970’s scientific interest in taxonomy was boosted by funding and for the first time University groups started to work on the taxonomy and phylogeny of the family. Christopher Wilkinson (1936–2010) (Figs 12, 14) developed an interest in the Nepticulidae during a sabbatical at the Canadian National Collection of Insects, Arachnids and Nematodes in Ottawa in the early 1970’s. Bryan P. Beirne (see above), then an applied entomologist in Burnaby, British Columbia, encouraged Wilkinson to revise this group for Canada and the USA. Wilkinson brought this research back to Portsmouth Polytechnic University, where he had worked since 1965, and two of his graduate students (Philip J. Newton and Malcolm J. Scoble) worked jointly with him on North American Nepticulidae, resulting in five papers dealing with all known species and several new ones (Wilkinson 1979; Wilkinson and Scoble 1979; Wilkinson 1981; Wilkinson and Newton 1981; Newton and Wilkinson 1982). Later, Malcolm Scoble (Fig. 17) obtained an academic position at the Transvaal Museum in Pretoria and continued working on the Nepticulidae of South Africa in a global context, gaining his PhD in 1983 (Scoble 1978a; b; 1979; 1980a; b; 1982; 1983). He presented the first cladistic analysis and classification of the family, dividing the family into two subfamilies, Pectinivalvinae and Nepticulinae, and the latter into two tribes, Nepticulini and Trifurculini. He also introduced the use of subgenera in the genus *Ectoedemia* and he named no fewer than 73 species. After these studies, Scoble turned his attention to other microlepidopterans, larger Lepidoptera and management while working, respectively, in the Oxford University Museum of Natural History and the Natural History Museum, London.

**Figure 24A.**
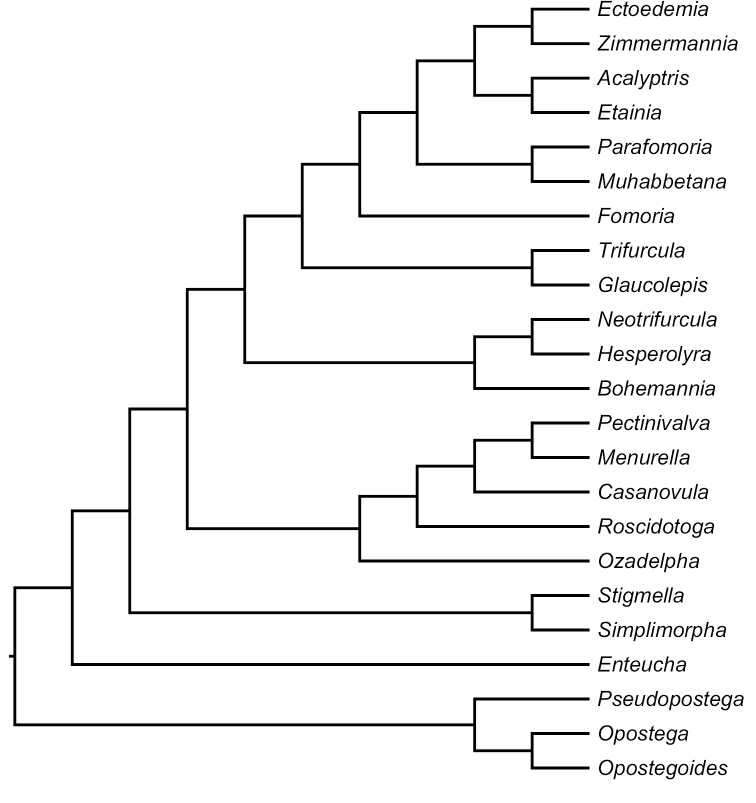
Schematic phylogram of Nepticulidae genera based on the best resolved tree of our Molecular phylogeny (Doorenweerd et al. 2016a).

**Figure 24B.**
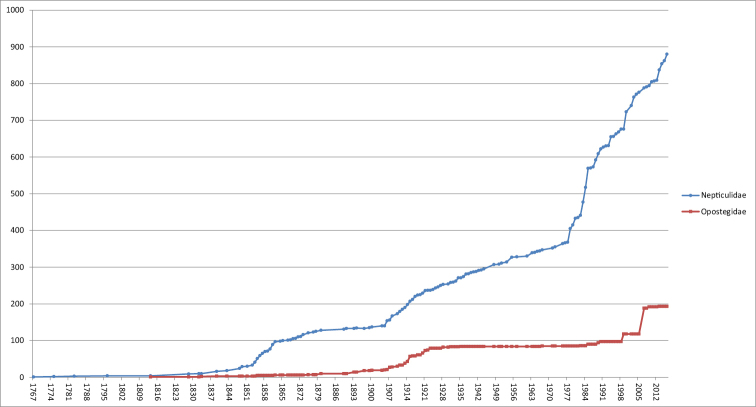
Cumulative number of valid species of Nepticulidae and Opostegidae described per year. Notice the long period that no Opostegidae were described (1935–1967) and the sudden increase in numbers of Nepticulidae since 1978.

Christopher Wilkinson became professor of Animal Systematics and Zoogeography at the Free University of Amsterdam in 1977, and employed Erik J. van Nieukerken (Figs 15, 20) as PhD student and Georgina Bryan, Steph B. J. Menken and later Jacobus J. (Koos) Boomsma as postdocs, all to work on various aspects of the systematics and evolution of Nepticulidae. In Amsterdam, several MSc students worked with Wilkinson on the taxonomy of *Stigmella* species groups in Europe, and the taxonomy of *Stigmella* in Japan and New Zealand (Kemperman et al. 1985; Schoorl et al. 1985; Schoorl and Wilkinson 1986; Donner and Wilkinson 1989). The nepticulid research group (Wilkinson 1982) also published a joint paper on the *Ectoedemia angulifasciella* complex (Wilkinson et al. 1983). Unfortunately, the department was short lived being closed down after budget cuts in 1985, cuts that were particularly detrimental to taxonomy. EvN finished his degree in 1986 after the group had been disbanded (van Nieukerken 1983b; 1985b; 1986b; van Nieukerken and Dop 1987). His thesis built upon Scoble’s results, but mainly based on the Holarctic fauna. He largely kept Scoble’s classification, added two more subgenera to *Ectoedemia*, and included larval and detailed new adult characters in the character matrix. Together with his MSc student, Henk Dop, he also extensively studied antennal ultrastructure. Van Nieukerken and Dop discovered a novel sensillum, which they named sensillum vesiculocladum (van Nieukerken and Dop 1987). The work led to a first checklist of the Western Palearctic species (van Nieukerken 1986a). Steph Menken’s research adopted new methods in the systematics of Nepticulidae by using allozyme characters, not only for species discrimination, but also for studies of population structure and parthenogenesis. Long before the advent of DNA barcoding, studies of allozymes facilitated species identification (Menken and Brouwer 1984; Menken and Wiebosch Steeman 1988; Menken 1990). After these studies, Steph Menken continued working on insect host relationships, particularly on Yponomeutidae as professor at the University of Amsterdam and Koos Boomsma continued his ecological studies, working as a professor in Copenhagen, particularly on social insects. The collaboration started by Menken, Boomsma and van Nieukerken resulted in a review paper published in 2010 on diet breadth in Lepidoptera, which also included important information on the host plants of Nepticulidae (Menken et al. 2010).

Wilkinson had also initiated collaboration to study Chinese Nepticulidae with professor Liu Youqiao from Beijing (Academia Sinica), resulting in a joint collecting trip by Erik van Nieukerken and Hans van Driel to China with Chinese counterparts in 1984 (van Driel and van Nieukerken 1985). After closing of the Amsterdam department, this research was unfortunately not continued. Only much later, van Nieukerken picked up the collaboration with Liu Youqiao and completed one joint paper on Chinese *Stigmella* (van Nieukerken and Liu 2000). Also plans by Wilkinson and Donald R. Davis (Fig. 11) for a volume in the series MONA (Moths of North America north of Mexico) never materialized, but checklists for the North American (Davis and Wilkinson 1983) and Neotropical Nepticulidae (Davis 1984) were completed.

At the same time that the Amsterdam group was active, Rimantas Puplesis (Fig. 15) started his PhD at the Zoological Institute of the Academy of Sciences in Leningrad, Soviet Union, and this resulted in many papers describing new species of Nepticulidae that he and colleagues had collected in Far East Russia (Primorskij Kraj) and Central Asia (Puplesis 1984a; b; c; e; 1985b; c; Puplesis and Ivinskis 1985). He also produced a phylogeny, although not strictly following Hennigian cladistics. From 1985, Rimantas continued his work in Vilnius (Lithuania), Pedagogical University and while his earlier papers were all in the Russian language, from 1988 he published mostly in English, and an important summary of all his earlier work was a book dealing with the Nepticulidae of the former Soviet Union, including 21 new species (Puplesis 1994). He continued his work first in Central Asia, where he had travelled extensively, usually together with his colleague Arūnas Diškus (Fig. 22), who received his PhD in 2005. From 2000 onwards their main interest shifted to the Neotropical fauna, and they travelled and collected particularly in Belize and Ecuador, and studied also the very interesting material earlier collected by Ebbe Nielsen and Ole Karsholt in Argentina and Chile (Nielsen 1980; Nielsen 1985). Puplesis made the first revisions of the Neotropic fauna in collaboration with Gaden S. Robinson (1949–2009) (Beccaloni et al. 2009) from the Natural History Museum in London (Puplesis and Robinson 2000; Puplesis et al. 2002a; 2002b). In 2003, Diškus and Puplesis published a book (Puplesis and Diškus 2003b) – partly in Lithuanian, partly in English – with several revisions of Nepticulidae from Asia and Europe and a first comprehensive global catalogue including both the Nepticuloidea and Tischerioidea (Diškus and Puplesis 2003). In 1996 Rimantas had become professor in Vilnius, and from 2007 onwards published under the name Jonas Rimantas Stonis. With a group of co-workers, including Arūnas Diškus (Fig. 22), Asta Navickaite, Agnė Rocienė and Andrius Remeikis (Fig. 23) he continued working on the Neotropical fauna and collecting, now also in Guatemala, Colombia, Mexico, Peru, Bolivia and Chile (Stonis et al. 2013c; 2013d; 2014; 2016b; Remeikis and Stonis 2015; Stonis and Remeikis 2015), but also revised the earlier described Far East Russian Nepticulidae (Rocienė and Stonis 2013; Stonis and Rocienė 2013) and worked on the Lithuanian and Crimean fauna (Navickaitė et al. 2011; 2014). Puplesis-Stonis and his co-authors have contributed by far the largest number of new species in Nepticulidae, by July 2016 in total ca 225 valid names.

**Figures 25–34.**
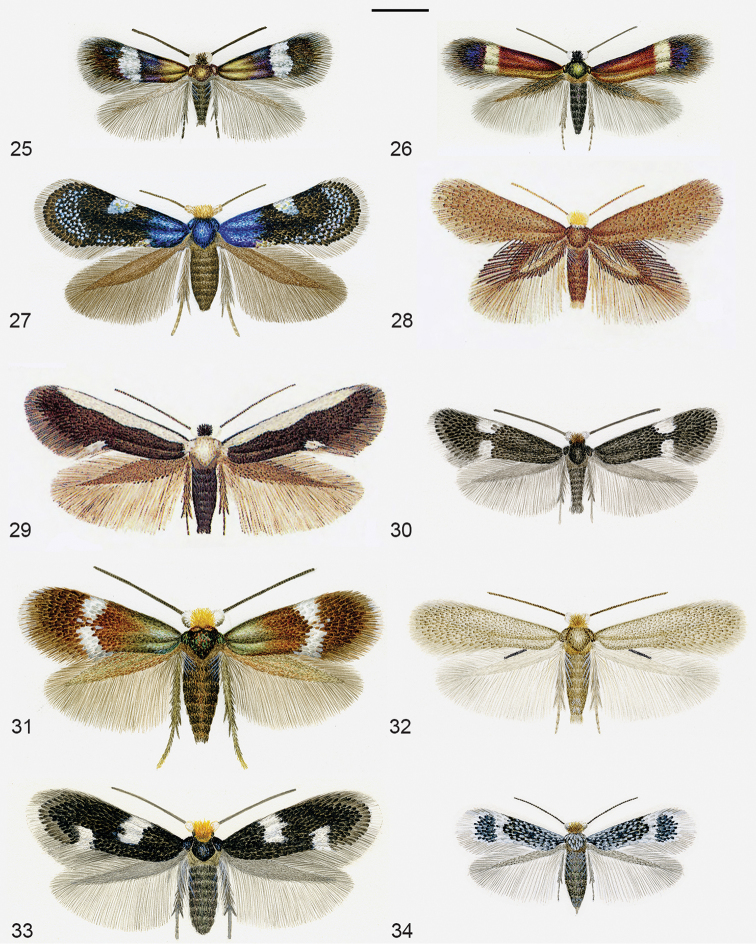
Diversity of Nepticulidae, all on same scale. **25** *Enteucha acetosae*, male, Austria **26** *Stigmella mespilicola*, male, Switzerland, holotype **27** *Roscidotoga callicomae*, female paratype, Australia, NSW **28** *Menurella libera*, male holotype, Australia, NSW **29** *Pectinivalva caenodora*, male holotype, Australia, NSW **30** *Glaucolepis lituanica* , male, Austria **31** *Bohemannia auriciliella*, male, The Netherlands **32** *Trifurcula iberica*, male paratype, Spain **33** *Fomoria weaveri*, female, Sweden **34** *Parafomoria helianthemella*, female, Czech Republic. Scale 1 mm. Watercolours by Roland Johansson, 25, 26, 31 and 33 published earlier by Johansson et al. (1990), 28 and 29 by Hoare et al. (1997). The left wings of 32 and 34 are digitally mirrored images of the right wings. These figures may be reproduced given that their author Roland Johansson and the present publication are credited.

**Figures 35–40.**
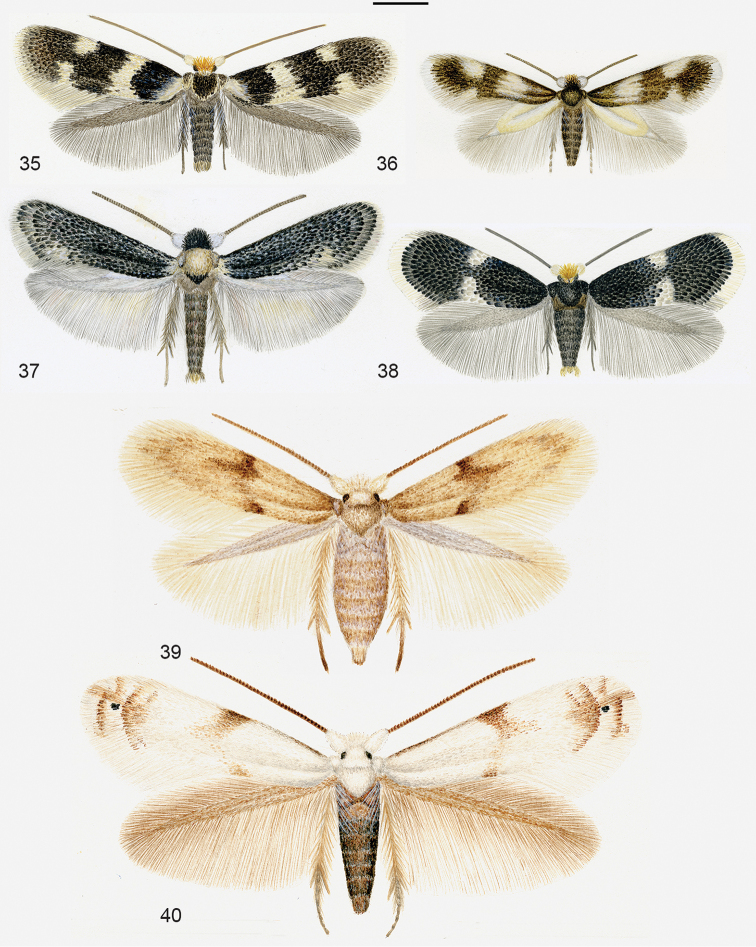
Diversity of Nepticulidae and Opostegidae, all on same scale. **35** *Etainia sericopeza*, male, Sweden **36** *Acalyptris platani*, male, Italy **37** *Zimmermannia atrifrontella*, male, Germany **38** *Ectoedemia klimeschi*, male, Austria **39** *Opostega spatulella*, female, Hungary **40** *Pseudopostega crepusculella*, male, Sweden. Scale 1 mm. Watercolours by Roland Johansson, published earlier by Johansson et al. (1990). These figures may be reproduced given that their author Roland Johansson and the present publication are credited.

In 1986 Erik van Nieukerken obtained a position at the Rijksmuseum van Natuurlijke Historie in Leiden (now Naturalis Biodiversity Center), but his initial responsibilities left him little opportunity to continue taxonomic work before he got a position as curator for microlepidoptera and Arachnida in 1999. Still he was able to finish some studies that he had started in Amsterdam, and continued collaborations with especially Roland Johansson and other Scandinavians: Ebbe Schmidt Nielsen (1950–2001) (Fig. 14), Bert Gustafsson (Fig. 14) and Ole Karsholt. This collaboration led to the two volume set in the series Fauna Entomologica Scandinavica (Johansson et al. 1990), very beautifully illustrated with water colours and line art by Roland Johansson (for examples see Figs 25–40) and also containing the first treatment of all nepticulid larvae of a single fauna (Gustafsson and van Nieukerken 1990). Nielsen, who had moved from Denmark to Canberra, Australia, had been the driving force for completion of this multi-authored work, and he was that again for the completion of the study of the previously described Australian Nepticulidae by him, Johansson and van Nieukerken. It was speeded up when Nielsen had been able to hire in 1994 Robert J. B. Hoare (Fig. 18) as PhD student to work on Australian Nepticulidae (Hoare et al. 1997). Hoare finished his study of Australian Nepticulidae in an unpublished PhD thesis (Hoare 1998), of which most relevant parts have since been published, including the description of the new genus *Roscidotoga* and a revision of *Pectinivalva*, including two subgenera (Hoare 2000a; Hoare 2000b; Hoare and van Nieukerken 2013), that are raised here to genus. After moving to Auckland, New Zealand, Hoare shifted his attention to the Lepidoptera of New Zealand in general.

In the late 1980’s and early 1990’s the Czech brothers Aleš and Zdeněk Laštůvka (Fig. 16) developed an interest in leafmining Lepidoptera, particularly Nepticulidae and Gracillariidae, and the opening of the Iron Curtain provided them with the opportunity to start extensive collecting in the Mediterranean region: Spain, Portugal, France, Italy, Croatia and Greece. They published a general guide for Central European species (Laštůvka and Laštůvka 1997) and many descriptions of new species (e.g. Laštůvka and Laštůvka 1990; 1994; 1998; 2000b; 2007). They also extensively collaborate with van Nieukerken on this fauna, resulting in several of the papers cited below. Aleš Laštůvka also started to illustrate the moths with water colours, as can be seen in several of their papers.

Moving into the 21^st^ century, Roland Johansson and Erik van Nieukerken renewed their collaboration, resulting in a second revision of the oak mining *Stigmella* (van Nieukerken and Johansson 2003). The initiation of a molecular laboratory at Naturalis prompted van Nieukerken to include sampling material for DNA analysis during his extensive collecting in many parts of the world, including Vietnam, Australia, Borneo and from 2010 onwards in the USA, Taiwan, Korea and Japan. Early molecular results in 2004 were shown at the International Entomological Congress in Brisbane (van Nieukerken et al. 2004a), but still considered insufficient for a full publication. Most publications by him and co-authors in the early 2000’s concern detailed European faunistics (van Nieukerken et al. 2004b; 2004c; 2006; van Nieukerken 2006) and two small and one large revision of West Palearctic *Acalyptris*, the subgenus Trifurcula (Levarchama) and *Ectoedemia*, subgenera *Ectoedemia* and *Zimmermannia* (van Nieukerken 2007a; b; van Nieukerken et al. 2010). Meanwhile in Naturalis the DNA barcoding project was adopted, and extracting DNA for barcoding (Ratnasingham and Hebert 2007) became a routine during dissection. Several papers provided barcodes as support for the taxonomy (van Nieukerken 2007a; 2010; Ivinskis et al. 2012; Laštůvka et al. 2013), and with MSc student Camiel Doorenweerd (Fig. 19), they published two larger papers concentrating on DNA barcodes and how to use them in Nepticulidae (van Nieukerken et al. 2012a; 2012b). Camiel Doorenweerd continued as PhD student from 2012 and analysed more genes. This resulted in several phylogeny papers (Doorenweerd et al. 2015a; Doorenweerd et al. 2016a), and some that are still in preparation. In order to improve calibration points for molecular phylogenies, all Nepticulidae fossils were re-assessed and catalogued (Doorenweerd et al. 2015b), and the named ones with formal descriptions are also included in the present catalogue.

After a paper on *Stigmella* from Japan (Kemperman et al. 1985), a collaboration between the Free University of Amsterdam group of Wilkinson and two Japanese leafminer specialists: Hiroshi Kuroko and Tosio Kumata, work on that fauna had slowed down, but a few species were named or discussed (Kuroko 1989; 1990; Kumata and Nakatani 1995; Kuroko 1999; Kuroko 2004; van Nieukerken and Kuroko 2005). The collaboration with Japan is now picked up again by van Nieukerken and Doorenweerd with Toshiya Hirowatari (Fig. 21), first in Osaka University, now professor in Kyushu University in Fukuoka and is expected to soon yield new publications. The study received further impetus from an amateur, Nagao Hirano (Fig. 21), who had collected and reared Nepticulidae extensively and described 12 new species (Hirano 2010; 2014); he also wrote the Nepticulidae texts for the book series “The standard of moths in Japan”, written in Japanese (Hirano 2013). At the same time some small-scale collaborations with China (prof. Li Houhun) and Korea (Bong Woo Lee) resulted in new material for study and will hopefully result in new publications.

Also work on North American Nepticulidae is now being continued collaboratively by van Nieukerken, Doorenweerd, in collaboration with Davis, David Wagner, Charley Eiseman, Greg Pohl and others, and this includes extensive DNA barcoding studies. A checklist of Lepidoptera of Canada with our contribution is now in preparation (Greg Pohl and Jean-François Landry, editors) and some of our data are presented in this catalogue.

## Taxonomic history of Opostegidae

The first opostegids to be named were *Tinea auritella* Hübner, 1813 (now *Pseudopostega auritella*) and *Elachista salaciella* Treitschke, 1833 (now *Opostega salaciella*). Zeller (1839) erected the genus *Opostega* for these two species and his new *Opostega crepusculella*, but he also included other small white species, that are now included in respectively *Phyllocnistis* Zeller, 1848 (Gracillariidae) and *Leucoptera* Hübner, 1825 (Lyonetiidae). Zeller did not select a type species, only much later Walsingham (1914) selected *salaciella* as such. In 1848 Zeller narrowed *Opostega* and only included species now still regarded as Opostegidae, by removing the other species to his new genera *Phyllocnistis* and *Cemiostoma* Zeller, 1848 (a junior synonym of *Leucoptera*). In 1855 five out of the six known European species had been named and two species from North America followed in the next 20 years (Clemens 1862a; Chambers 1875b).

Most early authors placed *Opostega* in the family Lyonetiidae (Stainton 1854; Frey 1856; Stainton 1859a), but Heinemann and Wocke ([1876]) already recognised the similarity with *Nepticula* and placed them in the Nepticulidae. The family name was first used by Meyrick (1893) as Opostegides, but Meyrick considered it to be a subfamily of Tineidae.

After the early descriptions, for a long period new species, mostly from other continents, were almost all named by Edward Meyrick and Lord Walsingham (66 in all) (Table 3). Just single species are attributed to Busck, Chrétien, Eyer, and Kuroko. A. Jefferis Turner (1923) described some species from Australia and O.H. Swezey (1921) discovered that the opostegids in Hawaii (now *Paralopostega*) make leafmines on *Melicope* (Rutaceae) (then named *Pelea*), whereas the life history of most Opostegidae remained a mystery. Only that of *Opostegoides scioterma* had been extensively described by Grossenbacher (1910), under the incorrect name *Opostega nonstrigella* (Davis and Stonis 2007). This species is a cambium miner of *Ribes* (Grossulariaceae), and another species of the same genus, *Opostegoides minodensis* (Kuroko, 1982) was later discovered to be a cambium miner of *Betula*.

**Table 3.** Number of Opostegidae species (valid and invalid) described per first author for authors who described at least six valid species.

First Author	# spp
Davis	74
Meyrick	53
Puplesis (Stonis)	21
Walsingham	11
Turner	6
17 authors	27
**Total**	**192**

In the 20^th^ century there was still no agreement about placement of the single genus *Opostega*, some textbooks placed it in the Nepticulidae, sometimes as subfamily (Spuler and Meess 1910; Hering 1932a), others kept it in Lyonetiidae, including the book first describing the male genitalia of three British species (Pierce and Metcalfe 1935). The first paper that recognised a grouping of families very similar to what we know nowadays was Busck (1914b), who considered the Opostegidae as family, close to Nepticulidae and Tischeriidae, in the Lepidoptera “Aculeates”, rather similar to what we now call lower Heteroneura. This placement was confirmed by larval studies a few years later (Heinrich 1918).

When studying the Opostegidae from the Asian parts of the Soviet Union, Kozlov (1985) realised that the differences in the genitalia of the single recognised genus *Opostega* were large and he erected a new genus, *Opostegoides* Kozlov, and split *Opostega* in two subgenera: *Opostega* and *Pseudopostega* Kozlov. Soon thereafter Davis (1989) completed a generic revision of the family and a phylogenetic analysis, finally resulting in the recognition of three new genera and raising *Pseudopostega* to full genus. The most peculiar new genus was *Notiopostega*, with the single species *Notiopostega atrata* Davis, 1989, with a wingspan of 13–18 mm by far the largest opostegid and largest species of the superfamily, of which the larvae make extremely long mines of up to seven meters in the cambium of *Nothofagus* trees (Carey et al. 1978; Davis 1989).

The four Northern European Opostegidae were reviewed and keyed by van Nieukerken (1990a), and a key for all six European species was presented by van Nieukerken et al. (2004b). Erik van Nieukerken also discovered and confirmed host plants for the northern European *Pseudopostega* species: *Lycopus europaeus* (Lamiaceae) for *Pseudopostega auritella* and *Mentha aquatica* (Lamiaceae) for *Pseudopostega crepusculella*. Both make long linear mines in the bark and gradually go deeper in the stem (van Nieukerken 1990a; Regier et al. 2015).

Rimantas Puplesis also worked extensively on Opostegidae, and revised the Oriental species together with Gaden Robinson (Puplesis and Robinson 1999) and together with Donald Davis they revised the New World species, including 70 new species and one new genus (Davis and Stonis 2007).

## Material and methods

### Taxonomic practice

#### Species

Ultimately, a catalogue or checklist is a list of species, arranged in a linear classification framework. Few taxonomists describe the methodology and philosophy they follow to recognise and delimit species. They often use the simple species concept: “a species is what a taxonomist calls a species” (Kalkman 1987). There are many species concepts, see eg Mallet (2001) for an overview. If we have to choose, we probably adhere most to the phylogenetic or diagnostic species concept (Mallet 2001; Isaac et al. 2004), but agree with Mallet’s notion that “agreement on a unified species-level taxonomy is possible, but will be forthcoming only if we accept that species lack a single, interpretable biological reality over their geographic range and across geological time”. In the taxonomic literature on Nepticuloidea, only Scoble (1983) discussed how he recognised species, but also he concluded that the practice is rather different from the theory. With the explosion of genetic data we have much more information nowadays than just two decades ago, but that does not mean that it has become much easier to recognise species. DNA barcodes are helpful, but can also complicate species discrimination, especially for allopatric populations (see below). In practice we recognise species by a combination of morphological characters, where genitalia are important, but not the only characters, and of the biology: host plant and mine morphology and of DNA data, particularly DNA barcodes and also of distribution data.

All species recognised here are in fact just hypotheses of species, open to further testing by more data and subject to ever ongoing evolution. We change the status of previously recognised species whenever our own research or our interpretation of published research has given reason to do so. This is particularly the case for several North American and Asian species that we (EvN and CD) have been studying the last years, and for which several publications are in preparation.

#### Subspecies

We do not recognise any subspecies. The systematic category in itself is problematic, and is particularly a part of the polytypic biological species concept, that can only be used for species where distributions are known in detail and the amount of hybridisation in border areas has been studied; as such subspecies are mostly used in charismatic groups such as vertebrates and butterflies, and even in these there is a tendency given by the phylogenetic species concept to abandon subspecies and raise them often to full species, particularly in birds (Isaac et al. 2004).

If allopatric populations are morphologically (almost) inseparable, and also share most of the biology, we simply use the same species name over the entire area (e.g. *Ectoedemia occultella* in the entire Palearctic and Nearctic, making the same characteristic mines on *Betula* and morphologically indistinguishable). In the case that there are more differences, we opt for separate species (e.g. *Ectoedemia intimella* and *Ectoedemia insularis*), particularly when consistent morphological differences are paired with a large diagnostic difference in DNA barcodes.

Very few subspecies have been described in the last 50 years in Nepticuloidea; for the few that were named (eg in two species of Neotropical *Pseudopostega*, (Davis and Stonis 2007)) we have raised the subspecies to full species when we see sufficient differences, or just left them as synonyms in other cases (e.g. *Stigmella anomalella pacifica* Puplesis, 1987). More than 50 years ago, the subspecies category had been used sometimes for biological forms (host plant races) and these are treated either as synonyms or raised to species level in a few cases.

#### Higher categories

Our classification of Nepticulidae follows our molecular phylogeny (Doorenweerd et al. 2016a), of which Fig. 24A shows the results in summary. Since the earlier recognised subfamily Nepticulinae and the tribe Nepticulini are not recovered, we abandon the use of subfamily and tribus here. After consultation with various lepidopterists we also choose to drop the subgenus category entirely, after we had to abandon them in *Ectoedemia* to maintain manageable monophyletic entities. In this way our classification resembles that of Diškus and Puplesis (2003).

The genus *Enteucha* is sister to all other Nepticulidae and therefore listed first. *Stigmella* is always split into two large clades that we name “Core *Stigmella*” for the clade with the type species *Stigmella anomalella* and “Non-core *Stigmella*” for the other one. Splitting *Stigmella* was considered impractical, due to a lack of good morphological apomorphies for these clades and the fact that *Stigmella* remains a well supported and recognisable entity.

The new Neotropical genus *Ozadelpha* (van Nieukerken et al. 2016) forms a clade with the former Pectinivalvinae. Also in *Pectinivalva* we have raised the three recently recognised subgenera to full genus (Hoare and van Nieukerken 2013).

This clade is sistergroup to the remaining genera, previously collectively named Trifurculini. Within this clade one can recognise three groups: A poorly supported clade of *Bohemannia* together with the new Neotropic genera *Neotrifurcula* and *Hesperolyra*, a clade of *Glaucolepis* + *Trifurcula* and a clade of the former *Ectoedemia* plus *Acalyptris* and *Parafomoria*. Following the principle of abandoning subgenera, we split *Trifurcula* now into *Trifurcula* and *Glaucolepis*, whereas *Levarchama* is reduced to a species group in *Trifurcula*.

Whereas the finding of the large “*Ectoedemia* clade” occurs in all our analyses, the order of the various genera changes in different analyses. The classification we adopt here follows the best supported phylogeny. The raising of *Muhabbetana* and *Zimmermannia* to full genus is novel, even though Hering (1940) already proposed *Zimmermannia* as full genus, this was never adopted until now.

The classification of Opostegidae follows the treatments by Davis (1989) and Davis and Stonis (2007), but also here we abandon the use of subfamilies, and we do not follow the splitting of *Pseudopostega* in species groups (see below).

#### Species groups

Species groups are a practical category to combine groups of species within large genera that share morphological and biological characters, and have been extensively used in Nepticulidae, particularly *Stigmella* since Johansson (1971). Our molecular studies show that many of these species groups are indeed monophyletic entities (Doorenweerd et al. 2015a; 2016a), but on the other hand there has also been a proliferation of often monotypic species groups for species with different genitalia that cannot be placed easily. We have here recognised especially the well diagnosable species groups that often are also supported by molecular data. These include many of the Holarctic groups in *Stigmella* and *Ectoedemia*. Groups for which we have no or insufficient molecular data have been recognised when morphologically uniform and comprising more than one species, others are listed as unplaced within genera, or the larger clades that we recognise in *Stigmella*. When several groups together form a monophyletic clade, we sometimes use the term “cluster” for that clade.

In *Pseudopostega* we have abandoned all species groups for the time being, and list the species alphabetically by geographic region. We have been unable to find phylogenetic or molecular support for the groupings and found them hard to use. For details for these groups we refer to the revisions that introduced them (Puplesis and Robinson 1999; Davis and Stonis 2007).

Species group names are not governed by the ICZN rules, and for practical reasons we have therefore changed some names that were based on junior synonyms, or in one case replaced it by the name of the type species that is included in the group (*Acalyptris psammophricta* group rather than *repeteki* group). We give the authors who used the group name for the first time, even though they may have used a different composition. Synonyms are not complete, and many group names for single species are not given in synonymy, nor are the many names for *Pseudopostega* groups.

We group a few sibling species in species complexes, but only in cases where these species are really very hard to almost impossible to separate morphologically, or only by either biological or molecular data. This applies only to three complexes in European *Ectoedemia* (van Nieukerken et al. 2012b). Recently, Stonis and Remeikis (2015) recognised two species complexes in Neotropical *Acalyptris*, but since these are simply groupings of rather similar, but diagnosable species, we do not concur to use the term “complex” for such assemblages, because there are multiple similar examples in global Nepticuloidea. The *Stigmella nigriverticella* complex (Remeikis and Stonis 2015) is indeed a complicated group, but the *saginella* group to which the complex belongs, is overall still a taxonomic puzzle that requires more study both morphologically and genetically.

### Order of the list

We order the genera and species groups according to our preferred phylogeny (Doorenweerd et al. 2016a), see also above. Species groups for which we do not have molecular data are listed at the end of each genus, monotypic species groups are largely abandoned, except in cases with an almost fully resolved phylogeny, where a single species does not group with any species group, as in the *Ectoedemia terebinthivora* group (Doorenweerd et al. 2015a), or when we know unnamed species that clearly group with the single species. The form genus *Stigmellites* for unplaced fossils is listed at the end of Nepticulidae and dubious taxa are listed at the end of each family. At the end of the checklist we list excluded taxa with their current taxonomic placement. These taxa were either originally described in *Nepticula* or *Opostega*, or once combined with these genera, but are now considered to belong to other families, in all 29 names, now belonging to 23 species in ca. eight to ten families. The list also includes four taxa of fossil leafmines, previously considered to possibly belong to Nepticulidae (Doorenweerd et al. 2015b).

Species are only listed in a phylogenetic order for those genera and groups where we have a detailed phylogeny, in practice only in *Ectoedemia* and a few species groups in *Stigmella*. Otherwise species are grouped by geographical region (order: WP, EP, OR, AFR, AUS, NEA, NEO) and listed alphabetically. Following the valid name we list the original combination plus those of the junior synonyms, followed by all now invalid subsequent combinations. Usually unavailable names are given at the end of the list of synonyms.

### Distribution

For each species we give the biogeographical region(s) of occurrence with an abbreviation. When the abbreviation is placed in square brackets the species is assumed to be an introduction. For type localities and simple maps of country records we refer to the scratchpad http://nepticuloidea.info/, which is regularly updated, and the Catalogue of Life.

### DNA barcodes and specimen data

Obtaining DNA barcodes has been standard in our taxonomic workflow since ca 2000, particularly for recent material or taxonomic relevant material such as types; in many cases additional genes have been sequenced. Our methodology has been explained in detail elsewhere (van Nieukerken et al. 2012a; 2012b; Doorenweerd et al. 2015a; 2016a). Where our classification in the first place is based on the phylogenetic analyses of several genes, we have used DNA barcodes often to place species that were not involved in those analyses. Further we used the barcodes as additional arguments in deciding about species status, even though there is no absolute criterion. The judgement of the value of barcode distances is particularly problematic in vicariant populations, such as island populations, where exchange of genetic material has ceased often a long time ago (Mutanen et al. 2012; 2016). We only opted for separate species when a large distance was paired with morphological and/or biological differences, shown in sufficient material. In cases when single specimens on an island show large Barcode distances, but hardly any morphological ones, we usually decide against splitting until further material and data are available. This is for instance the case in *Pseudopostega chalcopepla* and some island populations of *Stigmella perpygmaeella*. We have discussed the use of barcodes extensively for *Ectoedemia* and some groups of *Stigmella* (van Nieukerken et al. 2012a; 2012b). Barcodes have also been of great use to couple the unknown sex, eg. in some *Zimmermannia* species, or to link species with unknown life histories to barcoded larvae. We would like to stress the added value of DNA barcodes to taxonomy, and plea for adding barcode data to all taxonomic treatments, whenever possible.

We release here our dataset of barcodes of well identified material as BOLD Dataset DS-NEPCAT (DOI: 10.5883/DS-NEPCAT). This means that identities of specimens included were either identified by us or by colleagues providing the data. Obviously various barcoded larvae that did not match any adult barcodes and could not otherwise be identified may still belong to named species with unknown hosts. This dataset includes DNA barcodes of 3205 specimens, belonging to 779 species (733 Nepticulidae and 46 Opostegidae) of which 2574 specimens belong to 444 formally named species (409 Nepticulidae and 35 species of Opostegidae). The remaining 642 specimens belong to ca. 335 unnamed (or as yet unidentified) species (324 Nepticulidae and 11 Opostegidae). In the dataset 3071 barcodes are assigned a Barcode Identification Number (BIN), representing 900 BIN’s, which belong to 749 recognised species (717 Nepticulidae and 32 Opostegidae). A neighbour joining tree of the barcode data is supplied here as Supplementary material S2. Even though NJ trees contain phylogenetic signal, they cannot be considered as real phylogenetic trees, and species may be completely misplaced on the basis of barcodes alone.

Data for specimens to which we refer in our notes are listed in Supplementary material S1, and when barcoded these data are also available in dataset DS-NEPCAT.

### Host plants

We refrain from compiling a list of host plants here, even though it would have made the catalogue much more complete. A critical catalogue of host plant records requires considerable work on checking literature records, verifying host plant identifications and nomenclature and interpretation. We are working on such a catalogue to publish in the future, including many records of yet unnamed and unidentified species, to provide an insight into the host plant choices of the family. A catalogue of hosts without reference to sources was given by Diškus and Puplesis (2003), and for the West Palearctic and northern European alone by respectively van Nieukerken (1986a) and Johansson et al. (1990) and several more local revisions provide detailed host records. An analysis of all global host records on family level was used for the paper on the evolution of host associations in Lepidoptera (Menken et al. 2010).

### Nomenclatorial practice

The most important source for nomenclatorial practice is the International Code of Zoological Nomenclature (International Commission on Zoological Nomenclature 1999), that helps solving most nomenclatorial issues. However, interpreting the code is often difficult and we therefore highlight how we dealt with some cases in general, whereas in notes we discuss our individual choices where needed.

We include only names that were published as real scientific names, all available names according to ICZN, but also all unavailable names that are formed as real binomial or trinomial names. Unavailable names are marked with a double dagger (‡). For completeness’ sake we also include infrasubspecific names, that are not available according to the Code (ICZN 1.3.4). Many names based on leafmines only (the work of an animal) are unavailable when published after 1930 (ICZN 13.6.2), these are marked with the abbreviation “NNLM” (see below). After screening some of these descriptions, they appeared to be available after all, since part of the description dealt with the larva, eg. the colour, thus making the description available, even though it often is of little use in recognising the species.

We do not include any informal intermittent names (eg *Acalyptris* species 29135) or names given to unnamed barcoded species, as such names easily become obsolete and often are not published on paper or another fixed media anyway. Some of these intermittent names are listed on our scratchpad, though (van Nieukerken 2016) and many are used in the DNA barcodes dataset.

### References

All original descriptions and references with new combinations and synonymies have been examined by EvN (mostly from his own collection of reprints, copies and pdf’s), always trying to establish correct publication dates, which lead to changing a few publication dates of taxa. The Biodiversity Heritage Library (BHL) has been a particular great help, since this contains fully scanned volumes, allowing checking of dates on wrappers of issues and volumes, in addition to those available in our libraries. Nowadays there are many more repositories with scanned journals and books of particular countries, but since they do not always contain scans of wrappers and title pages, this checking is not always easy. It would be a great improvement if all these repositories were accessible through a single online source (BHL?); now it is not always easy to find these.

In the catalogue proper we present citations in the form as recommended by the Code (ICZN art. 50, 51, recommendations 51B–G), thus with comma, and use of ampersand rather than “and”. We always cite the page for the first description and for other nomenclatorial actions.

All references are listed here, and all references for original descriptions are also available in the scratchpad (van Nieukerken 2016). Where possible we added here the doi or url to the references.

### Combinations

We here introduce several new combinations, that became necessary after raising subgenera to full genus. While doing this, we realised that it is often difficult to find where new combinations have been made in the past, since ICZN (in contrast to the Botanical Code) does not have special requirements for new combinations, other than that the publication fulfils availability. In practice many new combinations have been made unintentionally in checklists, faunistic papers, faunas, without marking these, whereas several of the new combinations marked as such in literature (including some of our own) appeared not to have been new at all. In Nepticulidae this is particularly the case with the combinations of many species with *Stigmella*: after the recognition by Walsingham (1908a) that in fact *Stigmella* is valid rather than *Nepticula*, some authors started to use this generic name, but many did not, and this uncertainty continued for 70 years (Wilkinson 1978)! There are in fact not many “official” new combinations in *Stigmella* and for European species many were given without further notice in two faunal works (Gerasimov 1952; Hering 1957). To document the combination history for all names in Nepticuloidea, we here give the first author using the combination – as far as we have been able to ascertain – as author name after the brackets, in a similar fashion as botanists do and as has been recommended by ICZN (Recommendation 51G). Combinations only published online on webpages or databases (including the scratchpad) and not in online journals, are not accepted as validly published, and thus are given here as new combinations when valid. When these combinations are no longer valid and have been used only online, we mention this by adding “online comb.”

One deviation from the code is that we do not change the ending of species-group names to agree with the gender of the generic name (ICZN article 34.2). This follows the practice by most lepidopterists in leading catalogues and checklists, duly discussed by Sommerer (2002) and formally adopted by the Societas Europaea Lepidopterologica in a Resolution during their General Meeting at the 13th European Congress of Lepidopterology in Kørsør (Denmark) on June 4, 2002 (Sommerer *loc. cit.*). In Nepticuloidea this affects only few names anyway, since most generic names are feminine (except *Varius*, which is masculine).

### Synonyms

Similar to the combinations, finding the source for new synonyms is often difficult, and we therefore give the source for all subjective synonymies that we have been able to find. The few cases where we did not find it we leave this open, and we do not give a synonymy author for objective synonyms (names with the same type, such as new names, incorrect subsequent spellings) or for (infra)subspecific names within the same nominal species.

### Types

We have tried to include information on the primary types for each name in the scratchpad (van Nieukerken 2016), including information on the depository, the genitalia slide number and the host plant of the type – when reared. Information on types is provided here only for cases discussed in the notes. We also started adding photos of types to the scratchpad, but this will be far from complete when this paper is published.

Type species of genera and type genera of family group names are always given in the list, using several similar abbreviations as suggested by Pullen et al. (2014).

### Abbreviations and symbols used

AFR
 Afrotropical region 


AUS
 Australian and Pacific regions 


BIN
 Barcode Identification number 


BOLD
 Barcoding of Life Database 


EP
 East Palearctic region 


ICZN
 International Code of Zoological Nomenclature (International Commission on Zoological Nomenclature 1999) 


IOS
 Incorrect original spelling 


ISS
 Incorrect subsequent spelling 


JH
 Junior Homonym [of Genus] 


JPH
 Junior Primary Homonym [of Species] 


JSH
 Junior Secondary Homonym [of Species] 


ND
 Nomen dubium, dubious name, of which identity is unknown and untraceable 


NEA
Nearctic region


NEO
 Neotropical region 


NN
 Nomen nudum, unavailable name failing to conform to ICZN art 12 or 13 


NNLM
 Nomen nudum, for a name after 1931 based on the description of a leafmine (ICZN 13.6.2) 


NO
 Nomen oblitum, forgotten name, not used as valid name after 1900 


OR
 Oriental region 


RN
 Replacement name 


syn
 Synonymised by 


TG
 Type Genus 


TS/OD
 Type Species by Original Designation 


TS/OD,M
 Type Species by Original Designation and Monotypy 


TS/M
 Type Species by Monotypy 


TS/SD
 Type Species by Subsequent Designation 


UE
 Unjustified emendation 


URN
 Unnecessary replacement name 


WP
 West Palearctic region 


† Fossil species


‡ The double dagger is given before an nomenclatorially unavailable name.


1
 Superscript numbers refer to the taxonomic notes after the list. In the online html version of the list automatically hyperlinked. 


## Global Nepticuloidea: the state of the art

Table 4 provides an overview of the diversity of global Nepticuloidea for region and genus. Here we report 862 extant named species of Nepticulidae and 192 Opostegidae, a total of 1054. For the 18 fossil species we refer to our earlier catalogue (Doorenweerd et al. 2015b). We illustrate most genera with watercolours by Roland Johansson in Figures 25–40.

**Table 4.** Diversity of extant Nepticuloidea per geographic region and globally. Numbers are validly described species. When only unnamed species of a certain genus are known from a region this is indicated by a plus sign, brackets indicate occurrence just at the edge of the region. When generic assignment is uncertain, the number is given in *italics*.

	WP	EP	OR	AUS	AFR	NEA	NEO	Global
*Enteucha*	1	+	1			(2)	9	**11**
*Varius*					1			**1**
*Simplimorpha*	1				1			**2**
*Stigmella*	138	115	17	31	50	51	61	**428**
*Ozadelpha*							3	**3**
*Roscidotoga*				4				**4**
*Casanovula*				2				**2**
*Menurella*			1	11				**12**
*Pectinivalva*				7				**7**
*Neotrifurcula*							1	**1**
*Hesperolyra*							4	**4**
*Bohemannia*	3	5						**7**
*Areticulata*					1			**1**
*Glaucolepis*	34	2	1	+		1	*2*	**40**
*Trifurcula*	34		?		*2*			**36**
*Fomoria*	12	7	3	3	22	3	*1*	**48**
*Muhabbetana*	4				28			**32**
*Parafomoria*	8							**8**
*Etainia*	7	4	+		4	2		**16**
*Acalyptris*	23	1	7	+	22	9	32	**93**
*Zimmermannia*	9	4	+			5	+	**17**
*Ectoedemia*	47	29	+		5	14	*1*	**89**
**Nepticulidae Total**	**321**	**167**	**30**	**58**	**136**	**85**	**114**	**862**
# **genera**	**13**	**8**	**6**	**6**	**10**	**7**	**9**	**22**
*Notiopostega*							1	**1**
*Eosopostega*		1	1	+				**2**
*Neopostega*							6	**6**
*Paralopostega*				6				**6**
*Opostegoides*	1	6	15	1	4	1		**28**
*Opostega*	5	3	(1)					**7**
“*Opostega*”				17	3			**20**
*Pseudopostega*	3	2	22	+	8	9	82	**122**
**Opostegidae total**	**9**	**12**	**39**	**24**	**15**	**10**	**89**	**192**
# **genera**	**3**	**4**	**3**	**4**	**3**	**2**	**3**	**8**
**Nepticuloidea Total**	**330**	**179**	**69**	**82**	**151**	**95**	**203**	**1054**
# **genera**	**16**	**12**	**9**	**10**	**13**	**9**	**12**	**30**

There is a strong bias for the West Palearctic with 321 Nepticulidae, but the highest number of Opostegidae is for the Neotropics (89), which probably better reflects the reality. Fig. 24B shows that there is a constant increase in numbers of described species over time, and we are already aware of large numbers of unnamed species from most areas, but particularly the tropics and East Asia. The following lines present a short summary of our current knowledge of Nepticuloidea per region.

*Europe*. As has also become clear from the taxonomic history, until recently most work concentrated on the European fauna, and probably the majority of species have been described by now. Europe, excluding Cyprus, but including Macaronesia, contains 280 named species. We know of about 20 unnamed species of *Trifurcula*, eight *Parafomoria* and a small number of *Stigmella*, particularly *Rhamnus* feeders and several species belonging to the *Stigmella salicis* complex (van Nieukerken et al. 2012a), but otherwise do not expect many more to be discovered. Several key works deal with parts of Europe (Johansson et al. 1990; Laštůvka and Laštůvka 1997; Bengtsson et al. 2008), but this still excludes southern Europe.

The largest genus is *Stigmella*, as in most regions, but also *Ectoedemia* is very diverse here with a particularly rich fauna associated with oaks (van Nieukerken et al. 2010; 2012b). Both genera feed particularly on Fagaceae, Rosaceae, Betulaceae and Salicaceae, and *Stigmella* also on Rhamnaceae and a few other families. Although Europe has no endemic genera, the three Mediterranean genera *Parafomoria*, *Glaucolepis* and *Trifurcula* have by far their largest diversity here, particularly in the Iberian Peninsula, and to a lesser extent in Italy and Greece. Most of these feed on shrubs and some herbs in the typical Mediterranean habitats as Garrigue or Maquis, with *Parafomoria* specialising on Cistaceae, *Trifurcula* on Fabaceae, mostly brooms (Genisteae) and Loteae, whereas *Glaucolepis* has groups of species feeding on Lamiaceae, Apiaceae: *Bupleurum* and Plantaginaceae: *Globularia*. *Fomoria* has a centre of diversity in Greece and Turkey with at least six species feeding on *Hypericum. Acalyptris* also has most species in South East Europe, with the *staticis* group specialised on Plumbaginaceae and often occurring along the sea coast, and four species in the *platani* group on Platanaceae, Loranthaceae and Anacardiaceae. *Etainia* and *Zimmermannia* are widespread in Europe, the first associated with Sapindaceae (*Acer*) and Ericaceae (*Arctostaphylos*), whereas most *Zimmermannia* are barkminers in Fagaceae, and one in *Ulmus*. *Simplimorpha* has one species, *Stigmella promissa*, oligophagous on Anacardiaceae in southern Europea, whereas the single *Enteucha*, *Enteucha acetosae* occurs in Central and Eastern Europe on *Rumex* species (Polygonaceae).

*Western Palearctic Region*. Obviously Europe is part of this region, and its fauna continues along the Mediterranean coasts, but there is a great difference in the knowledge of the faunas inside and outside of Europe. The region has only 50 species that are not known from Europe (the total of 330 minus 280). The typical Mediterranean fauna continues in North Africa, Turkey and the Levant, but has been poorly sampled and will probably contain still many new taxa to discover. Turkey and Iran in particular are promising countries for high diversities, Turkey has a large diversity in such host plant genera as *Quercus* and *Hypericum*. In the desert areas of the Middle East, North Africa and the Arabian peninsula other groups become important, such as the *Acalyptris psammophricta* and *shafirkanus* groups (Puplesis et al. 1996; van Nieukerken 2010) and *Ectoedemia* and *Zimmermannia* are almost absent. Probably the best studied area in this region is Turkmenistan with nearly 50 recorded species (Puplesis and Diškus 2003a).

*Eastern Palearctic Region*. We separate West and East Palearctic more or less following the 64–65 East meridian, from North to South along the rivers Ob, Tobol, Turgay, Aral Sea, Karakum desert and the border between Iran and Afghanistan/Pakistan. In practice this means that we treat in Central Asia Turkmenistan and Iran as West Palearctic, and Afghanistan, Tadzhikistan and most of Uzbekistan and Kazakhstan as East Palearctic. This is a huge area, with major differences between the rather dry and mountainous Central Asia and the almost subtropical forested areas of the Sino-Japanese zone. The 167 named Nepticulidae and 12 Opostegidae certainly only represent the tip of the iceberg. Much work has been done on parts of the former Soviet Union: Central Asia and the Russian Far East (Puplesis 1994; Puplesis and Diškus 2003a; Rocienė and Stonis 2013; Stonis and Rocienė 2013), but for other regions descriptive work is only just beginning. In central Asia the best studied and most diverse country is Tadzhikistan with ca 40 species (Puplesis and Diškus 2003a). In this area *Stigmella* and *Ectoedemia* are particularly common in the mountainous areas, with species feeding particularly on Rosaceae, Salicaceae and Rhamnaceae, but in desert areas *Acalyptris* becomes an important element with members of the *psammophricta* and *shafirkanus* groups of which biologies are mostly completely unknown.

The northern part of the area, Siberia, has many trans-palearctic species, particularly *Stigmella* and *Ectoedemia* species feeding on *Betula* and Ericaceae feeders as *Stigmella lediella* on *Rhododendron* and *Fomoria weaveri* on *Vaccinium vitis-idaea*. A paper on Siberian nepticulids is in preparation (van Nieukerken, Kirichenko et al.).

Eastern Asia is a very rich faunistic area, with very diverse forests containing many potential host plants. The northernmost portion, Far east Russia, notably the Primorye region, has been best studied, with around 70 species known (Puplesis 1994; Rocienė and Stonis 2013; 2014, Stonis and Rocienė 2013). Initial fieldwork in China has resulted in the recognition of a very rich fauna of at least some 200 species, but to date only few have been described (van Driel and van Nieukerken 1985; van Nieukerken and Liu 2000). For Japan we have a working list of at least 120 species, the recent fauna work listed ca 75 (Hirano 2013; Hirowatari 2013). Obviously all these faunas have a large overlap, and are characterised by dominance of *Ectoedemia* and *Stigmella* (Kemperman et al. 1985; Hirano 2010; 2014). On many tree species there are multiple species in both genera, particularly in Fagaceae, Betulaceae, Juglandaceae, Rosaceae, Ulmaceae and Salicaceae, but also the fauna on Ericaceae and Sapindaceae is comparatively rich. Linking species names from species described from adults only to leafmines from which no adults have emerged is still a challenge, but due to an increasing number of available barcodes more and more of these are linked and publications are being prepared. In *Stigmella* there are also a few species feeding on monocots: the grass genus *Oplismenus* with *Stigmella oplismeniella* (Kemperman et al. 1985) and an unnamed species on *Carex* in Japan. The smaller genera *Etainia*, *Fomoria* and *Bohemannia* are relatively rich in this area, and in the more southern parts *Acalyptris* and *Enteucha* become more important, the latter with a number of species feeding on Polygonaceae, to date all unnamed (van Nieukerken 1986b). In the Opostegidae the genus *Opostegoides* is particularly rich with six named species.

*Oriental region*. The number of named species for the oriental region is still very low with only 30 Nepticulidae, but the Opostegidae have been revised (Puplesis and Robinson 1999) and number 39 species. Most of the species were described by Meyrick from India, and some from Nepal by Puplesis and Diškus (2003a). From our fieldwork in Vietnam, Borneo and Taiwan, it is clear that there is a rich fauna of leafmining Nepticulidae in this region, but it is a challenge to find sufficient numbers of larvae that can be reared to adults. We have never been very successful with light collecting adults in this region, but some – often poor – material is available in various collections. The genera *Stigmella* and *Acalyptris* are most diverse in this area, *Ectoedemia* is becoming much rarer further away from the Palearctic, but still occurs on *Rubus* from the North as far as Borneo, forming a complex of closely related species. In Borneo we also discovered the first non-Australian species in the genus *Menurella* (formerly in *Pectinivalva*): *Menurella xenadelpha* on *Syzygium acuminatissimum* (Myrtaceae) (Hoare and van Nieukerken 2013). *Stigmella* species have a wide variety of hosts, including tropical groups such as Dipterocarpaceae, Meliaceae, Phyllanthaceae and Moraceae (*Ficus* species), the large majority belonging to non-core *Stigmella*. There are also species in the *Stigmella betulicola* group feeding on grasses such as *Oplismenus* and Cyperaceae (*Cyperus*): *Stigmella xystodes* (van Nieukerken 2010). *Acalyptris* have an even wider range of hosts throughout the eudicots, most species belonging to the *Acalyptris platani* group.

The Opostegidae are characterised by many species in *Opostegoides* and *Pseudopostega*, and single species in *Opostega* and *Eosopostega* (Puplesis and Robinson 1999).

The fauna of northern parts of the Oriental region, Nepal, northern Vietnam, Taiwan, has a much more Palearctic character, often sharing the same host plant genera and comprises for instance also several *Ectoedemia*, the Polygonaceae feeding *Enteucha* species, including the only named one, *Enteucha diplocosma* and *Fomoria* species feeding both on Lamiaceae: *Callicarpa* and *Vitex* and Hypericaceae (van Nieukerken 2008).

*Afrotropical Region*. The knowledge of the African fauna is very unbalanced, of the 136 named Nepticulidae species the great majority is known from southern Africa, thanks to the diligent rearing work by Lajos Vári, who also named several species (Vári 1955; 1963) and the revisions by Malcolm Scoble (Scoble 1978a; b; 1980a; b; 1983). Further just a few were described from Gambia (Gustafsson 1985) and outside that area just five species have been named; also collections are poor in unidentified material. But even in South Africa much of the diversity is still unknown, and every new collection contains unnamed species. Two monotypic endemic genera *Varius* and *Areticulata* have an uncertain placement and may be synonyms to existing genera. The genera *Simplimorpha* and *Muhabbetana* are near endemic, both also occur in the adjacent Mediterranean region. *Simplimorpha* is specialised on Anacardiaceae, and most African *Muhabbetana* feed on Ebenaceae and Celastraceae. *Fomoria* and *Acalyptris* are relatively diverse genera with broad host ranges. *Stigmella* is the largest genus, as everywhere, but with a dominance of species belonging to non-core *Stigmella*. The *Ectoedemia commiphorella* group has several species feeding on Burseraceae and is possibly sister to the northern hemisphere *Ectoedemia* (Doorenweerd et al. 2015a). Also the group of African *Etainia* species seems to be sister to the Holarctic *Etainia*, but hosts and biology are completely unknown. The two species assigned to *Trifurcula* (Scoble 1980a) may not belong there, a closer study of these species is needed.

The island fauna of the Indian Ocean is still poorly known: two species occur on Aldabra (Seychelles), see below under note 21, and one is named from Madagascar (*Fomoria scobleella*). However, we have seen examples or DNA barcodes of Nepticulidae from Madagascar that show the presence of the genera *Acalyptris*, *Muhabbetana*, *Ectoedemia* and *Stigmella*. No species are known from Réunion or Mauritius.

The African Opostegidae, with 15 named species, have not yet been revised, but we have been able to recombine several “*Opostega*” species here with *Opostegoides* or *Pseudopostega*, the only genera known with certainty from Africa.

*Australian Region*. Australia proper has a very rich and special fauna with an estimate of about 250 species of Nepticulidae as currently available in collections (Hoare 1998), of which approximately 30 have been named. Australia has four (almost) endemic genera: the small genus *Roscidotoga* (4 species) in the eastern rainforest, specialised on plants in the Oxalidales (Hoare 2000a; van Nieukerken et al. 2011), and *Casanovula*, *Menurella* and *Pectinivalva* with around 160 species, all but one (that feeds on *Quintinia*, Paracryphiaceae) feeding on Myrtaceae, with a large number feeding on *Eucalyptus* (Hoare and van Nieukerken 2013). The other large genus is *Stigmella* with an estimated number of at least 80 species, with the most important host families being Rutaceae, Fabaceae (including *Acacia*), Sapindaceae, Euphorbiaceae, Phyllanthaceae and Rhamnaceae (Hoare 1998); a large number of these species belong to non-core *Stigmella*. *Fomoria* has just a few species in the *vannifera* group on Brassicaceae and one in the *weaveri* group on Salicaceae (Hoare 2000b; Doorenweerd et al. 2016a), *Acalyptris* includes ca. seven unnamed species in the *Acalyptris platani* group (hosts Phyllanthaceae, Melastomataceae, Loranthaceae) and there is also one *Glaucolepis* in the *raikhonae* group. There is a rich opostegid fauna with 24 named species, but they have not yet been revised. The genera *Opostegoides* and *Pseudopostega* at least occur here, and there is possibly also a species of *Eosopostega* (Hoare 1998).

The fauna of New Guinea is virtually unknown, we have only seen a few species of *Stigmella*.

New Zealand has a fauna quite unrelated to Australia, only the genus *Stigmella*, with the *Stigmella ogygia* group occurs here, with 27 named and ca 12 unnamed species. Interestingly, its sistergroup is the *epicosma* group in South America. Many New Zealand species feed on shrubby and herbaceous Asteraceae, such as *Olearia* or *Senecio*, but there are also species feeding on Malvaceae, Ericaceae and Nothofagaceae (Donner and Wilkinson 1989). There are no opostegids known from New Zealand.

On the many Pacific islands only few Nepticulidae are known, *Stigmella ebbenielseni*, feeding on *Pipturus* (Urticaceae) was described from Guam (van Nieukerken and van den Berg 2003), and a few other Urticaceae feeding *Stigmella* are reported in the same paper from Polynesia and Fiji. No nepticulids are known from Hawaii, but there occurs the interesting endemic genus *Paralopostega*, with a small radiation of species, making leafmines on *Melicope* (formerly *Pelea*, Rutaceae) (Swezey 1921; Zimmerman 1978, Davis, 1989).

On New Caledonia many mines of Nepticulidae have been seen (RJBH), several on Cunoniaceae, but no serious collecting has taken place. The adults of two species are known, but these have not been placed to genus, and require further study. The fauna could well be diverse and important, in common with the very rich and unusual flora of this island.

*Nearctic region*. The fauna of the Nearctic is relatively poor with 85 named Nepticulidae and ten Opostegidae. The Nepticulidae were revised in the early 1980’s (Wilkinson 1979; 1981; Wilkinson and Scoble 1979; Wilkinson and Newton 1981; Newton and Wilkinson 1982) and the Opostegidae recently (Davis and Stonis 2007), but much material remained unstudied and a lot has been collected since the 1980’s. Two of us (EvN and CD) have been collecting leafmines throughout North America, whereas Davis has draft descriptions of 20 new species and several other people are contributing to a much better knowledge. Some of our results are shown in this list with notes, and several manuscripts are underway. Even though there are still quite a few unnamed species to describe, overall the fauna is not as rich as in the Palearctic, or even Europe alone. We do not know the cause, but it is interesting to note that other groups of leafminers are much more diverse in the Nearctic than in the Palearctic (Tischeriidae, Bucculatricidae, several groups of Gracillariidae).

By far the largest genus is *Stigmella*, with groups specialising on amongst others Fagaceae, Betulaceae, Juglandaceae, Rhamnaceae, Rosaceae and Anacardiaceae; typical species groups for this region are the *saginella* and *quercipulchella* groups with oak feeding species and the *prunifoliella* group feeding on Anacardiaceae, Rhamnaceae (*Ceanothus*) and Rosaceae (*Prunus*); most other species groups are shared with the Palearctic. Particularly in California and Arizona there are largely unstudied radiations of species on *Quercus* and *Ceanothus*. *Ectoedemia* is not nearly as diverse as in the Palearctic, and for instance has not more than three species feeding on oaks, but it has some other hosts including Platanaceae and Cornaceae (*Nyssa*). The genus *Acalyptris* still has a large undiscovered diversity, with several species specialising on Cyperaceae in wetlands, like the type species of *Microcalyptris*, *Acalyptris scirpi*. *Fomoria*, *Etainia* and *Glaucolepis* are small genera with just a few species, but the barkmining *Zimmermannia* has a few more species (even though we synonymise here eight names) and is a widespread element, with associations with Fagaceae, Salicaceae and possibly Betulaceae. The fauna of southern Florida is more Neotropical with its two species of *Enteucha* on seagrape *Coccoloba uvifera* (Polygonaceae) and various other species in *Acalyptris*, *Stigmella* and *Pseudopostega*. In northern North America there are several Holarctic species, particularly feeding on *Betula* (eg *Ectoedemia occultella*, *minimella*) or Ericaceae (*Fomoria weaveri*) and some European species have been introduced (paper in preparation).

*Neotropical Region*. Currently 123 named species of Nepticulidae (plus 13 informally named species) and 89 Opostegidae are known, but due to active research new species are added regularly, particularly by Stonis and co-authors (reviewed by van Nieukerken et al. (2016) and see above). The fauna differs remarkably from most other regions, with three endemic genera in Nepticulidae: *Ozadelpha*, *Neotrifurcula* and *Hesperolyra*, and two endemic genera in Opostegidae: *Notiopostega* and *Neopostega*. Most of the named Nepticulidae belong to *Stigmella*, but also here with endemic species groups: the *epicosma* group, the *eurydesma* group, the *barbata* group and the *purpurimaculae* group. Host plant relationships are also special: as in the New Zealand *ogygia* group, many species in the *epicosma* group, particularly common in the Andes, feed on Asteraceae (Stonis et al. 2015a; 2016b). The *Nothofagus* forest of austral South America is the locality for several endemics, and *Notiopostega* is known to make extremely long mines in the cambium of *Nothofagus* trees. It is possible that species of *Neotrifurcula* are also barkminers of *Nothofagus*, and for the *Stigmella purpurimaculae* group there is a strong suspicion that they make leafmines in *Nothofagus* (Stonis et al. 2014). *Ozadelpha* species are associated with Myrtales: Melastomataceae and Myrtaceae, and the only species of *Hesperolyra* where the host is known also feeds on Myrtaceae. There are nine species of *Enteucha*, and where known they feed on Polygonaceae: *Coccoloba*. For the large number of *Acalyptris* species there are only few host records, including Fabaceae and Verbenaceae. In the northern part of the Neotropics there is greater similarity with the Nearctic fauna, and this is particularly the case for the recently discovered diversity of *Quercus* miners in Guatemala and Colombia (Stonis et al. 2013e; Remeikis and Stonis 2015).

The generic placement of the few Neotropical species now placed in *Ectoedemia*, *Fomoria* and *Glaucolepis* requires further study, *Zimmermannia* occurs in Mesoamerica with an unnamed species with genitalia very similar to *Zimmermannia bosquella* (Puplesis and Robinson 2000).

In the Opostegidae the genus *Pseudopostega* is remarkably diverse with 82 species, unfortunately as yet without any knowledge of host associations (Davis and Stonis 2007).

*Atlantic Islands*. Not a single nepticuloid species is known from the Oceanic Atlantic islands south of Macaronesia (such as St. Helena, Ascension, Tristan da Cunha, the Falklands), but their occurrence still could be possible. The fauna of Macaronesia is mostly endemic, particularly on the Canarian Islands, with mostly Mediterranean or African elements of *Glaucolepis*, *Muhabbetana*, *Fomoria*, *Acalyptris* and *Stigmella*. The fauna of the Azores and Madeira is very poor with respectively one and four species, some of which have been introduced (Karsholt and Vieira 2005; Aguiar and Karsholt 2006). No nepticulids are yet known from Cabo Verde, but are expected to occur there, and in the North Atlantic there are no Nepticulidae known from Greenland or Iceland, nor any of the smaller islands.

## Catalogue

SUPERFAMILY **NEPTICULOIDEA** Stainton, 1854: 295

FAMILY **NEPTICULIDAE** Stainton, 1854: 295 (TG: *Nepticula* Heyden, 1843)

Family Stigmellidae Hampson, 1918: 387 (TG: *Stigmella* Schrank, 1802)

Subfamily Pectinivalvinae Scoble, 1983: 12 (TG: *Pectinivalva* Scoble, 1983) (syn.: Puplesis, 1994: 36)

Subfamily Nepticulinae Stainton, 1854

Subfamily Stigmellinae Hampson, 1918

Subfamily Trifurculinae Scoble, 1983: 16 (TG: *Trifurcula* Zeller, 1848) **syn. n.**

Tribe Nepticulini Stainton, 1854: 295

Tribe Stigmellini Hampson, 1918

Tribe Trifurculini Scoble, 1983: 16 **syn. n.**

***Enteucha*** Meyrick, 1915a: 241 (TS/M: *Enteucha cyanochlora* Meyrick, 1915) ^1^

*Johanssonia* Borkowski, 1972a: 702; JH of *Johanssonia* Selensky, 1914 (Annelida, Hirudinea) (TS/OD,M: *Nepticula acetosae* Stainton, 1854) (syn: van Nieukerken, 1986a: 7)

*Artaversala* Davis, 1978: 219 (TS/OD,M: *Artaversala gilvafascia* Davis, 1978) (syn: van Nieukerken, 1986a: 7)

*Oligoneura* Davis, 1978: 217; JH of *Oligoneura* Bigot, 1878 (Brachiopoda) (TS/OD,M: *Oligoneura basidactyla* Davis, 1978) (syn: van Nieukerken, 1986a: 7)

*Manoneura* Davis, 1979: 276; RN for *Oligoneura* Davis, 1978 (syn: van Nieukerken, 1986a: 7) ^1^

*Johanssoniella* Koçak, 1981: 99; RN for *Johanssonia* Borkowski (syn: van Nieukerken, 1986a: 7)

***Enteucha acetosae*** (Stainton, 1854) van Nieukerken, 1986a: 7 (Fig. 25) WP

‡ *Nepticula acetosae* Shield, 1853: 4153 NN

*Nepticula acetosae* Stainton, 1854: 303

*Nepticula acetosella* Doubleday, 1859: 36 UE

*Nepticula arifoliella* Klimesch, 1940b: 92

*Stigmella acetosae* (Stainton, 1854) Beirne, 1945: 200

*Johanssonia acetosae* (Stainton, 1854) Borkowski, 1972a: 702

*Johanssoniella acetosae* (Stainton, 1854) Koçak, 1981: 99

*Stigmella arifoliella* (Klimesch, 1940) Hering, 1957: 912

‡ Nepticula arifoliella var. altvateri Skala, 1941b: 79

***Enteucha diplocosma*** (Meyrick, 1921) Diškus & Puplesis, 2003: 321 OR

*Nepticula diplocosma* Meyrick, 1921a: 411

***Enteucha acuta*** Puplesis & Diškus in Puplesis et al., 2002: 21 NEO

***Enteucha basidactyla*** (Davis, 1978) van Nieukerken, 1986a: 54 ^1^ NEA,NEO

*Oligoneura basidactyla* Davis, 1978: 218

*Manoneura basidactyla* (Davis, 1978) Davis, 1979: 276

***Enteucha contracolorea*** Puplesis & Robinson, 2000: 20 NEO

***Enteucha cyanochlora*** Meyrick, 1915a: 241 NEO

***Enteucha gilvafascia*** (Davis, 1978) van Nieukerken, 1986a: 54 NEA,NEO

*Artaversala gilvafascia* Davis, 1978: 221

***Enteucha hilli*** Puplesis & Robinson, 2000: 19 NEO

***Enteucha snaddoni*** Puplesis & Robinson, 2000: 21 NEO

***Enteucha trinaria*** (Puplesis & Robinson, 2000) **comb. n.** NEO

*Manoneura trinaria* Puplesis & Robinson, 2000: 23

***Enteucha terricula*** Puplesis & Robinson, 2000: 20 NEO

***Varius*** Scoble, 1983: 14 (TS/OD,M: *Stigmella ochnicola* Vári, 1955)

***Varius ochnicola*** (Vári, 1955) Scoble, 1983: 14 AFR

*Stigmella ochnicola* Vári, 1955: 336

*Varius ochnicolus* (Vári, 1955) Scoble, 1983: 14 [variant]

***Simplimorpha*** Scoble, 1983: 15 (TS/OD,M: *Stigmella lanceifoliella* Vári, 1955)

***Simplimorpha promissa*** (Staudinger, 1871) van Nieukerken, 1986a: 6 WP

*Nepticula promissa* Staudinger, 1871: 325

*Nepticula robiniella* Gustafsson, 1973: 197 (syn: van Nieukerken, 1986a: 6)

*Stigmella promissa* (Staudinger, 1871) Klimesch, 1951b: 64

***Simplimorpha lanceifoliella*** (Vári, 1955) Scoble, 1983: 15 AFR

*Stigmella lanceifoliella* Vári, 1955: 331

***Stigmella*** Schrank, 1802: 169 (TS/SD (Walsingham, 1908a: 1007) ^2^: Phalaena (Tinea) anomalella Goeze, 1783)

*Nepticula* Heyden, 1843: 208 (TS/SD (Walsingham, 1908a: 1007): *Tinea aurella* Fabricius, 1755) (syn: Walsingham, 1908a: 1008) ^2^

*Dysnepticula* Börner, 1925: 370 (TS/OD: Phalaena (Tinea) anomalella Goeze, 1783)

*Astigmella* Puplesis, 1984a: 111 (TS/OD: *Astigmella dissona* Puplesis, 1984) (syn: van Nieukerken, 1986a: 7)

[*Microsetia* Stephens, 1834 *sensu* Kirby, 1897: 313 (TS/incorrect SD by Kirby, 1897: *Nepticula microtheriella* Stainton, 1854) (see Wilkinson 1978: 17)]

### Non-core *Stigmella* ^3^

**ungrouped species in Non-core *Stigmella***

***Stigmella freyella*** (Heyden, 1858) Vári, 1950: 182 WP

*Nepticula freyella* Heyden, 1858: 175

***Stigmella kurilensis*** Puplesis, 1987: 8 EP

***Stigmella ebbenielseni*** van Nieukerken & Van den Berg, 2003: 28 AUS

***Stigmella resplendensella*** (Chambers, 1875) Newton & Wilkinson, 1982: 456 ^4^ NEA

*Nepticula resplendensella* Chambers, 1875b: 118

***Stigmella unifasciella*** (Chambers, 1875) Newton & Wilkinson, 1982: 438 NEA

*Nepticula unifasciella* Chambers, 1875b: 119

***Stigmella gallicola*** van Nieukerken & Nishida in van Nieukerken et al., 2016: 7 NEO

***Stigmella prunifoliella* group** (Newton & Wilkinson, 1982: 385) ^5^

*Stigmella prunetorum* group (Johansson, 1971: 245)

*Stigmella bifasciella* group (Wilkinson & Scoble, 1979: 59)

***Stigmella prunetorum*** (Stainton, 1855) Beirne, 1945: 198 WP,EP

*Nepticula prunetorum* Stainton, 1855: 72

*Nepticula dimidiatella* Herrich-Schäffer, 1855a: 352 (syn: Herrich-Schäffer, 1860: 59)

*Nepticula perpusillella* Herrich-Schäffer, 1855a: 353 (syn: Frey, 1856: 390)

*Nepticula prunetella* Doubleday, 1859: 36 UE

*Nepticula ligustrella* Rössler, 1867: 395 (syn: van Nieukerken & Johansson, 1987: 461)

*Nepticula punctella* Threlfall, 1884: 113 ISS

‡ Nepticula prunetorum var. aviella Skala, 1934b: 6 NNLM

‡ Stigmella prunetorum var. aviella (Skala, 1934b) NNLM

***Stigmella diniensis*** (Klimesch, 1975) Leraut, 1980: 49 WP

*Nepticula diniensis* Klimesch, 1975c: 5

***Stigmella ceanothi*** (Braun, 1910) Newton & Wilkinson, 1982: 387 NEA

*Nepticula ceanothi* Braun, 1910: 172

***Stigmella cerea*** (Braun, 1917) Newton & Wilkinson, 1982: 407 NEA

*Nepticula cerea* Braun, 1917: 172

***Stigmella intermedia*** (Braun, 1917) Wilkinson & Scoble, 1979: 62 NEA

*Nepticula intermedia* Braun, 1917: 171

***Stigmella prunifoliella*** (Clemens, 1861) Newton & Wilkinson, 1982: 385 NEA

*Nepticula prunifoliella* Clemens, 1861: 84

*Nepticula bifasciella* Clemens, 1862: 133 (syn: Newton & Wilkinson, 1982: 385)

*Nepticula serotinaeella* Chambers, 1873: 126 (syn: Braun, 1917: 170)

*Stigmella bifasciella* (Clemens, 1862) Wilkinson & Scoble, 1979: 59

***Stigmella rhoifoliella*** (Braun, 1912) Newton & Wilkinson, 1982: 391 NEA

*Nepticula rhoifoliella* Braun, 1912: 93

***Stigmella gossypii*** (Forbes & Leonard, 1930) Newton & Wilkinson, 1982: 404 NEO

*Nepticula gossypii* Forbes & Leonard, 1930: 149

***Stigmella schinivora*** van Nieukerken, 2016 in van Nieukerken et al., 2016: 15 NEO

***Stigmella ultima* group** (Puplesis, 1984a: 116)

***Stigmella aceris*** (Frey, 1857) Gerasimov, 1952: 222 WP

*Nepticula aceris* Frey, 1857: 386

*Nepticula penicillata* Heinemann & Wocke, [1876]: 744 ^6^ (syn: van Nieukerken & Johansson, 1987: 461)

*Nepticula szöcsi* Klimesch, 1956: 423 (syn: Klimesch, 1978a: 246)

*Nepticula szoecsi* Klimesch, 1956: 423 ISS (syn: Klimesch, 1978a: 246)

***Stigmella acerna*** Puplesis, 1988: 277 ^7^ WP

‡ *Stigmella acerifoliella* Dovnar-Zapolski, 1969: 20 NNLM; **syn. n.** ^7^

***Stigmella bicolor*** Puplesis, 1988: 276 EP

***Stigmella bumbegerensis*** Puplesis, 1984d: 509 EP

***Stigmella kozlovi*** Puplesis, 1984a: 118 EP

***Stigmella monella*** Puplesis, 1984a: 117 EP

***Stigmella orientalis*** Kemperman & Wilkinson, 1985: 21 EP

***Stigmella semiaurea*** Puplesis, 1988: 275 WP,EP

***Stigmella tegmentosella*** Puplesis, 1984a: 117 EP

***Stigmella ultima*** Puplesis, 1984a: 117 EP

*Stigmella japonica* Kemperman & Wilkinson, 1985: 20 (syn: Puplesis, 1994: 81)

***Stigmella ulmivora* group** (Johansson, 1971: 244)

***Stigmella kazakhstanica*** Puplesis in Puplesis et al. 1991: 70 WP,EP

*Stigmella pimschoorli* Puplesis, 1994: 64 (syn: Puplesis et al., 1996: 192)

***Stigmella ulmiphaga*** (Preissecker, 1942) Klimesch, 1948b: 62 WP

*Nepticula ulmiphaga* Preissecker, 1942: 208

*Nepticula gracilivora* Skala, 1942: 6 (syn: Klimesch, 1975c: 4)

***Stigmella ulmivora*** (Fologne, 1860) Beirne, 1945: 199 ^8^ WP

*Nepticula ulmivora* Fologne, 1860: 92

*Nepticula ulmifoliae* Hering, 1931: 531 (syn: Emmet, 1974d: 151)

*Nepticula ulmicola* Hering, 1932: 569 (syn: Emmet, 1974d: 151)

*Stigmella ulmifoliae* (Hering, 1931) Vári, 1944b: xxv

*Stigmella ulmicola* (Hering, 1932) Vári, 1944b: xxv

‡ *Nepticula ulmella* Hofmann, 1858: 191 NN (syn: Segerer, 1997: 188) ^8^

***Stigmella viscerella*** (Stainton, 1853) Beirne, 1945: 199 WP

*Nepticula viscerella* Stainton, 1853: 3958

*Nepticula subvirescens* Meyrick, 1934b: 523 (syn: Diškus & Puplesis, 2003: 343)

*Nepticula tauromeniella* Groschke, 1944: 117 (syn: Klimesch, 1975c: 2)

*Stigmella tauromeniella* (Groschke, 1944) Hering, 1957: 1090

***Stigmella alisa*** Puplesis, 1985c: 63 EP

***Stigmella amuriella*** Puplesis, 1985c: 62 EP

***Stigmella auricularia*** Puplesis, Diškus & Juchnevič in Puplesis & Diškus, 2003a: 245 EP

***Stigmella multispicata*** Rocienė & Stonis in Stonis & Rocienė, 2014: 205 ^9^ EP

***Stigmella nireae*** Kemperman & Wilkinson, 1985: 18 EP

***Stigmella palionisi*** Puplesis, 1984b: 596^7^ EP

*Stigmella nakamurai* Kemperman & Wilkinson, 1985: 16 **syn. n.** ^10^

***Stigmella eurydesma* group** (Puplesis & Robinson, 2000: 32)

***Stigmella albilamina*** Puplesis & Robinson, 2000: 33 NEO

***Stigmella eurydesma*** (Meyrick, 1915a) Davis, 1984: 18 NEO

*Nepticula eurydesma* Meyrick, 1915a: 255

***Stigmella fuscilamina*** Puplesis & Robinson, 2000: 34 NEO

***Stigmella saginella* group** (Wilkinson & Scoble, 1979: 39)

***Stigmella castaneaefoliella*** (Chambers, 1875b) Wilkinson & Scoble, 1979: 44 NEA

*Nepticula castaneaefoliella* Chambers, 1875b: 117

***Stigmella flavipedella*** (Braun, 1914) Wilkinson & Newton, 1981: 31 NEA

*Nepticula flavipedella* Braun, 1914: 19

***Stigmella macrocarpae*** (Freeman, 1967) **comb. n.** ^11^ NEA

*Nepticula latifasciella* Chambers, 1878: 106 JPH of *Nepticula latifasciella* Herrich-Schäffer, 1855 ^11^ (syn: Wilkinson & Scoble, 1979: 47)

*Nepticula macrocarpae* Freeman, 1967: 19

*Stigmella latifasciella* (Chambers, 1878) Wilkinson & Scoble, 1979: 47

***Stigmella nigriverticella*** (Chambers, 1875b) Newton & Wilkinson, 1982: 423 NEA

*Nepticula nigriverticella* Chambers, 1875b: 118

*Nepticula maculosella* Chambers, 1880a: 193 (syn: Braun, 1917: 194)

***Stigmella saginella*** (Clemens, 1861) Wilkinson & Scoble, 1979: 39 NEA

*Nepticula saginella* Clemens, 1861: 85

*Nepticula fuscocapitella* Chambers, 1873: 128 (syn: Braun, 1917: 195)

*Nepticula quercicastanella* Chambers, 1873: 127 (syn: Braun, 1917: 195)

***Stigmella sclerostylota*** Newton & Wilkinson, 1982: 429 NEA

***Stigmella aurifasciata*** Diškus & Stonis in Stonis et al., 2013c: 8 NEO

***Stigmella crassifoliae*** Remeikis & Stonis, 2015: 410 NEO

***Stigmella jaguari*** Remeikis & Stonis in Stonis et al., 2013c: 6 NEO

***Stigmella lauta*** Diškus & Stonis in Stonis et al., 2013c: 6 NEO

***Stigmella robleae*** Remeikis & Stonis, 2015: 411 NEO

***Stigmella sublauta*** Remeikis & Stonis in Stonis et al., 2013c: 8 NEO

***Stigmella paliurella* group** (van Nieukerken, 1986a: 8)

***Stigmella birgittae*** Gustafsson, 1985: 171 ^12^ WP,AFR

*Stigmella omani* Puplesis & Diškus, 2003a: 207 (syn: van Nieukerken, 2010: 493)

‡ *Nepticula amseli* Skala, 1941b: 78 NNLM; **syn. n.** ^12^

***Stigmella abaiella*** Klimesch, 1979: 21 ^13^ WP

***Stigmella ficulnea*** Puplesis & Krasnilnikova, in Puplesis, 1994: 65 ^13^ WP

***Stigmella longicornuta*** Puplesis & Diškus, 2003a: 217 WP

***Stigmella paliurella*** Gerasimov, 1937: 285 ^14^ WP

*Nepticula paliurella* (Gerasimov, 1937) Klimesch, 1940a: 177 ^14^

*Nepticula paliurella* Klimesch, 1940c

***Stigmella turbatrix*** Puplesis, 1994: 66 ^15^ WP,EP

‡ *Stigmella celtivora* Dovnar-Zapolski, 1969: 39 NNLM

***Stigmella zizyphi*** Walsingham, 1911: 190 WP,AFR

*Stigmella ziziphivora* Gustafsson, 1985: 171 (syn: van Nieukerken, 2010: 495)

*Nepticula zizyphi* (Walsingham, 1911) Skala, 1938a: 45

***Stigmella morivora*** Hirano, 2010: 128 EP

***Stigmella sruogai*** Puplesis & Diškus, 2003a: 204 EP

***Stigmella isochalca*** (Meyrick, 1916b) Diškus & Puplesis, 2003: 325 OR

*Nepticula isochalca* Meyrick, 1916b: 6

***Stigmella nepali*** Puplesis & Diškus, 2003a: 206 OR

***Stigmella phyllanthina*** (Meyrick, 1906b) Common, 1990: 156 AUS

*Nepticula phyllanthina* Meyrick, 1906b: 60

***Stigmella naturnella* group** (new)

*Stigmella dissona* group (Puplesis, 1994: 58)

***Stigmella naturnella*** (Klimesch, 1936) Klimesch, 1948b: 65 WP,EP

*Nepticula naturnella* Klimesch, 1936: 205

*Astigmella dissona* Puplesis, 1984a: 112 (syn: van Nieukerken et al., 2004a: 133)

*Stigmella dissona* (Puplesis, 1984a) Puplesis, 1994: 58

***Stigmella mirabella*** (Puplesis, 1984a) Puplesis, 1994: 58 EP

*Astigmella mirabella* Puplesis, 1984a: 112

***Stigmella tiliae* group** (Johansson, 1971: 245)

***Stigmella tiliae*** (Frey, 1856) Beirne, 1945: 198 WP

*Nepticula tiliae* Frey, 1856: 375

***Stigmella sashai*** Puplesis, 1984b: 594 EP

*Stigmella regina* Puplesis, 1984b: 596 (syn: Rocienė & Stonis, 2013: 95)

***Stigmella betulicola* group** (Johansson, 1971: 245)

*Stigmella corylifoliella* group (Wilkinson & Scoble, 1979: 50)

***Stigmella alnetella*** (Stainton, 1856) Beirne, 1945: 198 WP

*Nepticula alnetella* Stainton, 1856: 43

***Stigmella betulicola*** (Stainton, 1856) Beirne, 1945: 198 WP,EP,NEA

*Nepticula betulicola* Stainton, 1856: 42

*Nepticula betulicolella* Doubleday, 1859: 36 UE

Nepticula betulicola var. nanivora Petersen, 1930: 61 (syn: Johansson, 1971: 245)

*Stigmella nanivora* (Petersen, 1930) Hering, 1957: 183

***Stigmella glutinosae*** (Stainton, 1858) Beirne, 1945: 198 WP

*Nepticula glutinosae* Stainton, 1858: 96

*Nepticula distinguenda* Heinemann, 1862b: 305 (syn: Schoorl & Wilkinson, 1986: 234)

*Nepticula rubescens* Heinemann, 1871: 214 (syn: Klimesch, 1950a: 27)

*Nepticula glutinosella* Porritt, 1883: 173 UE

*Stigmella rubescens* (Heinemann, 1871) Gerasimov, 1952: 256

‡ Nepticula glutinosae var. alni-viridis Skala, 1939e: 111 NNLM (syn: Klimesch, 1950a: 27)

‡ Nepticula rubescens var. incanae Skala, 1941a: 57 NNLM (syn: Klimesch, 1950a: 27)

***Stigmella gutlebiella*** A. Laštuvka & Huemer, 2002: 604 WP

***Stigmella luteella*** (Stainton, 1857a) Beirne, 1945: 198 WP,EP

*Nepticula luteella* Stainton, 1857a: 110

*Nepticula luteellina* Skala, 1941b: 79 (syn: Skala, 1948: 121)

***Stigmella microtheriella*** (Stainton, 1854) Fletcher & Clutterbuck, 1945: 59 ^16^ WP,EP,[NEA,AUS]

*Nepticula microtheriella* Stainton, 1854: 302

*Stigmella cathepostis* Kemperman & Wilkinson, 1985: 10 **syn. n.** ^16^

*Microsetia microtheriella* (Stainton, 1854) Kirby, 1897: 313

***Stigmella nivenburgensis*** (Preissecker, 1942) Klimesch, 1951b: 59 ^17^ WP,EP

*Nepticula nivenburgensis* Preissecker, 1942: 209

*Stigmella populnea* Kemperman & Wilkinson, 1985: 13 **syn. n.** ^17^

***Stigmella sakhalinella*** Puplesis, 1984a: 115 WP,EP

*Stigmella discidia* Schoorl & Wilkinson, 1986: 237 (syn: van Nieukerken & Johansson, 1987: 470)

*Nepticula distinguenda* auct. [misapplied] (syn: van Nieukerken & Johansson, 1987: 470)

*Stigmella distinguenda* auct. [misapplied]

***Stigmella attenuata*** Puplesis, 1985c: 62 EP

***Stigmella betulifoliae*** Puplesis & Diškus, 2003a: 179 EP

***Stigmella conchyliata*** Kemperman & Wilkinson, 1985: 11 EP

***Stigmella cornuta*** Rocienė & Stonis in Stonis et al., 2013e: 206 ^18^ EP

***Stigmella excelsa*** Puplesis & Diškus, 2003a: 182 EP

***Stigmella kumashidei*** Hirano, 2014: 20 EP

***Stigmella oplismeniella*** Kemperman & Wilkinson, 1985: 12 EP

***Stigmella pamirbetulae*** Puplesis & Diškus, 2003a: 180 EP

***Stigmella titivillitia*** Kemperman & Wilkinson, 1985: 14 EP

***Stigmella caryaefoliella*** (Clemens, 1861) **stat. rev., comb. n.** ^19^ NEA

*Nepticula caryaefoliella* Clemens, 1861: 84

***Stigmella corylifoliella*** (Clemens, 1861) Wilkinson & Scoble, 1979: 50 NEA

*Nepticula corylifoliella* Clemens, 1861: 83

*Nepticula virginiella* Clemens, 1861: 83 (syn: Braun, 1917: 179)

*Nepticula minimella* Chambers, 1873: 179 (syn: Braun, 1917: 179)

*Nepticula opulifoliella* Braun, 1914: 22 (syn: Wilkinson & Scoble, 1979: 50)

*Nepticula paludicola* Braun, 1917: 177 (syn: Wilkinson & Scoble, 1979: 51)

*Nepticula exasperata* Braun, 1930: 17 (syn: Wilkinson & Scoble, 1979: 51)

***Stigmella juglandifoliella*** (Clemens, 1861) Wilkinson & Scoble, 1979: 57 NEA

*Nepticula juglandifoliella* Clemens, 1861: 84

***Stigmella longisacca*** Newton & Wilkinson, 1982: 436 NEA

***Stigmella myricafoliella*** (Busck, 1900) Grossbeck, 1917: 145 ^20^ NEA

*Nepticula myricafoliella* Busck, 1900: 238

*Nepticula obscurella* Braun, 1912: 95 **syn. n.** ^20^

***Stigmella ostryaefoliella*** (Clemens, 1861) Wilkinson & Scoble, 1979: 54 ^21^ NEA

*Nepticula ostryaefoliella* Clemens, 1861: 83

***Stigmella himalayai*** Puplesis & Diškus, 2003a: 208 OR

***Stigmella xystodes*** (Meyrick, 1916b) Diškus & Puplesis, 2003: 328 WP,OR

*Nepticula xystodes* Meyrick, 1916b: 6

*Nepticula liochalca* Meyrick, 1916b: 6 (syn: van Nieukerken, 2010: 496)

*Nepticula homophaea* Meyrick, 1918b: 181 (syn: van Nieukerken, 2010: 496)

*Stigmella liochalca* (Meyrick, 1916b) Diškus & Puplesis, 2003: 328

*Stigmella homophaea* (Meyrick, 1918b) Diškus & Puplesis, 2003: 363

***Stigmella allophylica*** Scoble, 1978b: 97 AFR

***Stigmella allophylivora*** Gustafsson, 1985: 167 AFR

***Stigmella androflavus*** Scoble, 1978b: 104 AFR

***Stigmella generalis*** Scoble, 1978b: 102 AFR

***Stigmella geranica*** Scoble, 1978b: 96 AFR

***Stigmella hortorum*** Scoble, 1978b: 99 AFR

***Stigmella pelanodes*** (Meyrick, 1920b) **comb. n.** ^22^ AFR

*Nepticula pelanodes* Meyrick, 1920b: 116

***Stigmella potgieteri*** Scoble, 1978b: 99 AFR

***Stigmella satarensis*** Scoble, 1978b: 97 AFR

***Stigmella tragilis*** Scoble, 1978b: 98 AFR

***Stigmella tropicatella*** Legrand, 1965: 27 ^23^ AFR

***Stigmella triumfettica*** Scoble, 1978b: 107 AFR

***Stigmella divina* group** (Puplesis & Diškus, 2003a: 212)

***Stigmella divina*** Puplesis, Diškus & van Nieukerken, 1997: 55 WP

***Stigmella maculifera*** Puplesis & Diškus, 2003a: 212 WP

***Stigmella skulei*** Puplesis & Diškus, 2003a: 213 WP

### Unplaced tropical species - most probably in non-core *Stigmella*

African species

*Stigmella fluida* group (Scoble, 1978b: 92)

*Stigmella ingens* group (Scoble, 1978b: 111)

***Stigmella abachausi*** (Janse, 1948) Scoble, 1978b: 104 AFR

*Nepticula abachausi* Janse, 1948: 162

***Stigmella abutilonica*** Scoble, 1978b: 93 AFR

***Stigmella ampullata*** Scoble, 1978b: 108 AFR

***Stigmella angustivalva*** Scoble, 1978b: 113 AFR

***Stigmella caliginosa*** (Meyrick, 1921b) Scoble, 1983: 43 AFR

*Nepticula caliginosa* Meyrick, 1921b: 140

***Stigmella celtifoliella*** Vári, 1955: 338 AFR

***Stigmella charistis*** Vári, 1963: 71 AFR

***Stigmella confinalis*** Scoble, 1978b: 111 AFR

***Stigmella crotonica*** Scoble, 1978b: 100 AFR

***Stigmella dombeyivora*** Scoble, 1978b: 107 AFR

***Stigmella ficivora*** Gustafsson, 1985: 170 AFR

***Stigmella fluida*** (Meyrick, 1911a) Scoble, 1978b: 94 AFR

*Nepticula fluida* Meyrick, 1911a: 236

***Stigmella galactacma*** (Meyrick, 1924b) Diškus & Puplesis, 2003: 364 AFR

*Nepticula galactacma* Meyrick, 1924b: 89

***Stigmella grewiae*** Scoble, 1978b: 112 AFR

***Stigmella gustafssoni*** (Căpuşe, 1975) Diškus & Puplesis, 2003: 366 AFR

*Nepticula gustafssoni* Căpuşe, 1975: 211

***Stigmella ingens*** (Meyrick, 1913) Scoble, 1978b: 112 AFR

*Nepticula ingens* Meyrick, 1913: 327

***Stigmella irrorata*** (Janse, 1948) Scoble, 1978b: 95 AFR

*Nepticula irrorata* Janse, 1948: 168

***Stigmella letabensis*** Scoble, 1978b: 113 AFR

***Stigmella liota*** Vári, 1963: 73 AFR

***Stigmella maytenivora*** Gustafsson, 1985: 174 AFR

***Stigmella naibabi*** Mey, 2004: 29 AFR

***Stigmella nigrata*** (Meyrick, 1913) Scoble, 1978b: 106 AFR

*Nepticula nigrata* Meyrick, 1913: 326

***Stigmella panconista*** (Meyrick, 1920a) Diškus & Puplesis, 2003: 363 AFR

*Nepticula panconista* Meyrick, 1920a: 312

***Stigmella parinarella*** Vári, 1955: 337 AFR

***Stigmella perplexa*** (Janse, 1948) Scoble, 1978b: 103 AFR

*Nepticula perplexa* Janse, 1948: 172

***Stigmella platyzona*** Vári, 1963: 67 AFR

***Stigmella porphyreuta*** (Meyrick, 1917a) Scoble, 1978b: 110 AFR

*Nepticula porphyreuta* Meyrick, 1917a: 13

***Stigmella pretoriata*** Scoble, 1978b: 109 AFR

***Stigmella protosema*** (Meyrick, 1921b) Scoble, 1978b: 109 AFR

*Nepticula protosema* Meyrick, 1921b: 140

***Stigmella rhomboivora*** Gustafsson, 1985: 167 AFR

***Stigmella rhynchosiella*** Vári, 1955: 338 AFR

***Stigmella urbica*** (Meyrick, 1913) Scoble, 1978b: 103 AFR

*Nepticula urbica* Meyrick, 1913: 326

***Stigmella uwusebi*** Mey, 2004: 30 AFR

***Stigmella varii*** Scoble, 1978b: 95 AFR

***Stigmella wollofella*** (Gustafsson, 1972) Gustafsson, 1985: 174 ^24^ AFR

*Nepticula wollofella* Gustafsson, 1972: 158

*Nepticula mandingella* Gustafsson, 1972: 157 **syn. n.** ^24^

*Stigmella mandingella* (Gustafsson, 1972) Diškus & Puplesis, 2003: 366

***Stigmella worcesteri*** Scoble, 1983: 16 RN for *Stigmella pallida* Scoble, 1978 AFR

*Stigmella pallida* Scoble, 1978b: 105 JSH of *Stigmella pallida* (Braun, 1917)

Australian species

***Stigmella leucargyra*** (Meyrick, 1906b) Nielsen, 1996: 16 AUS

*Nepticula leucargyra* Meyrick, 1906b: 57

***Stigmella symmora*** (Meyrick, 1906b) Nielsen, 1996: 16 AUS

*Nepticula symmora* Meyrick, 1906b: 59

Oriental species

***Stigmella aeriventris*** (Meyrick, 1932) Diškus & Puplesis, 2003: 364 OR

*Nepticula aeriventris* Meyrick, 1932: 312

***Stigmella alicia*** (Meyrick, 1928) Diškus & Puplesis, 2003: 364 OR

*Nepticula alicia* Meyrick, 1928b: 461

***Stigmella argyrodoxa*** (Meyrick, 1918) Fletcher, 1933: 83 OR

*Nepticula argyrodoxa* Meyrick, 1918b: 181

***Stigmella auxozona*** (Meyrick, 1934) Diškus & Puplesis, 2003: 365 OR

*Nepticula auxozona* Meyrick, 1934a: 468

***Stigmella elachistarcha*** (Meyrick, 1934) Diškus & Puplesis, 2003: 365 OR

*Nepticula elachistarcha* Meyrick, 1934a: 467

***Stigmella hoplometalla*** (Meyrick, 1934) Puplesis & Diškus, 2003a: 215 OR

*Nepticula hoplometalla* Meyrick, 1934a: 467

***Stigmella ipomoeella*** (Gustafsson, 1976) Diškus & Puplesis, 2003: 328 OR

*Nepticula ipomoeella* Gustafsson, 1976: 45

***Stigmella neodora*** (Meyrick, 1918) Diškus & Puplesis, 2003: 363 OR

*Nepticula neodora* Meyrick, 1918b: 182

***Stigmella oligosperma*** (Meyrick, 1934) Diškus & Puplesis, 2003: 365 OR

*Nepticula oligosperma* Meyrick, 1934a: 468

***Stigmella polydoxa*** (Meyrick, 1911) Diškus & Puplesis, 2003: 363 OR

*Nepticula polydoxa* Meyrick, 1911c: 107

### Core *Stigmella* ^3^

***Stigmella rhamnella*/*lapponica*/*sanguisorbae* cluster**

***Stigmella tiliella* group** (Puplesis et al., 2002: 63)

***Stigmella tiliella*** (Braun, 1912) Newton & Wilkinson, 1982: 442 NEA

*Nepticula tiliella* Braun, 1912: 90

***Stigmella kimae*** Puplesis & Robinson, 2000: 35 NEO

***Stigmella rhamnella* group** (new)

***Stigmella alaternella*** (Le Marchand, 1937) Klimesch, 1948b: 63 WP

*Nepticula alaternella* Le Marchand, 1937: 234

***Stigmella armeniana*** Puplesis, 1994: 90 WP

***Stigmella catharticella*** (Stainton, 1853) Beirne, 1945: 199 WP

*Nepticula catharticella* Stainton, 1853: 3955

***Stigmella crenulatae*** (Klimesch, 1975) van Nieukerken, 1986a: 8 WP

*Nepticula crenulatae* Klimesch, 1975c: 2

***Stigmella kopetdagica*** Puplesis, 1994: 92 WP

***Stigmella pyrellicola*** (Klimesch, 1978) van Nieukerken, 1986a: 9 WP

*Nepticula pyrellicola* Klimesch, 1978b: 264

***Stigmella rhamnella*** (Herrich-Schäffer, 1860) Klimesch, 1951b: 56 WP

*Nepticula rhamnella* Herrich-Schäffer, 1860: 60

Nepticula rhamnella var. rhamnipumilae Klimesch, 1950a: 49

***Stigmella rhamnophila*** (Amsel, 1934) van Nieukerken, 1986a: 8 WP

*Nepticula rhamnella rhamnophila* Amsel, 1934: 317

‡ *Nepticula rhamnophila* Amsel & Hering, 1931: 142 NNLM

***Stigmella klimeschi*** Puplesis, 1988: 274 EP

***Stigmella kurotsubarai*** Kemperman & Wilkinson, 1985: 15 EP

***Stigmella taigae*** Puplesis, 1984a: 112 EP

***Stigmella condaliafoliella*** (Busck, 1900) Grossbeck, 1917: 145 NEA

*Nepticula condaliafoliella* Busck, 1900: 238

***Stigmella diffasciae*** (Braun, 1910) Newton & Wilkinson, 1982: 398 NEA

*Nepticula diffasciae* Braun, 1910: 172

***Stigmella inconspicuella*** Newton & Wilkinson, 1982: 400 NEA

***Stigmella rhamnicola*** (Braun, 1916) Newton & Wilkinson, 1982: 393 NEA

*Nepticula rhamnella* Braun, 1912: 96 JPH of *Nepticula rhamnella* Herrich-Schäffer, 1860

*Nepticula rhamnicola* Braun, 1916: 55 RN for *Nepticula rhamnella* Braun, 1912

***Stigmella maya*** Remeikis & Stonis in Stonis et al., 2013b: 224 NEO

***Stigmella sanguisorbae* group** (van Nieukerken, 1986a: 10)

*Stigmella rosaefoliella* group (Wilkinson & Scoble, 1979: 14)

***Stigmella muricatella*** (Klimesch, 1978) van Nieukerken, 1986a: 9 WP,EP

*Nepticula muricatella* Klimesch, 1978b: 266

***Stigmella polymorpha*** Puplesis & Diškus, 2003a: 183 WP

***Stigmella rolandi*** van Nieukerken, 1990c: 239 WP,EP

***Stigmella sanguisorbae*** (Wocke, 1865) Gerasimov, 1952: 258 WP

*Nepticula sanguisorbae* Wocke, 1865: 269

***Stigmella thuringiaca*** (Petry, 1904) Gerasimov, 1952: 263 WP,EP

*Nepticula thuringiaca* Petry, 1904: 267

*Nepticula nickerli* Rebel in Nickerl, 1908: 116 (syn: Rebel, 1909: (269))

***Stigmella fasciola*** Puplesis & Diškus, 2003a: 185 EP

***Stigmella trisyllaba*** Puplesis in Puplesis et al., 1992: 51 EP

***Stigmella rosaefoliella*** (Clemens, 1861) Wilkinson & Scoble, 1979: 14 ^25^ NEA

*Nepticula rosaefoliella* Clemens, 1861: 85

*Stigmella rosaefoliella rosaefoliella* (Clemens, 1861) Wilkinson & Scoble, 1979: 14^25^

***Stigmella lapponica* group** (Johansson, 1971: 245)

*Stigmella malella* group (Johansson, 1971: 245)

***Stigmella confusella*** (Wood & Walsingham, 1894) Vári, 1944a: 215 WP,EP,NEA

*Nepticula confusella* Wood & Walsingham, 1894: 272

***Stigmella lapponica*** (Wocke, 1862) Fletcher & Clutterbuck, 1945: 61 WP,EP,NEA

*Nepticula lapponica* Wocke, 1862: 251

*Nepticula lapponicella* Herrich-Schäffer, 1863c: 23 UE

*Nepticula lusatica* Schütze, 1905: 204 (syn: Johansson & Nielsen, 1990: 141)

*Nepticula vossensis* Grønlien, 1928: 217 (syn: Krogerus, 1971: 31)

*Stigmella lusatica* (Schütze, 1905) Beirne, 1945: 200

*Stigmella vossensis* (Grønlien, 1928) Gerasimov, 1952: 269

***Stigmella malella*** (Stainton, 1854) Beirne, 1945: 199 WP

*Nepticula malella* Stainton, 1854: 304

*Nepticula angustella* Heinemann & Wocke, [1876]: 756 ^6^ (syn: van Nieukerken & Johansson, 1987: 461)

*Nepticula nigrobrunnella* Groschke, 1939: 716 (syn: van Nieukerken & Johansson, 1987: 461)

*Nepticula nigrobrunella* auct. ISS

*Stigmella nigrobrunnella* (Groschke, 1939) Hering, 1957: 837

‡ Nepticula malella var. prunicola Skala, 1939f: 126 NN

***Stigmella maloidica*** Puplesis in Puplesis & Arutyunova, 1991: 573 EP

***Stigmella braunella*** (Jones, 1933) Wilkinson & Scoble, 1979: 13 NEA

*Nepticula braunella* Jones, 1933: 49

***Stigmella slingerlandella*** (Kearfott, 1908) Wilkinson & Scoble, 1979: 19 NEA

*Nepticula slingerlandella* Kearfott, 1908: 187

**unplaced in *rhamnella*/*lapponica*/*sanguisorbae* cluster**

***Stigmella boehmeriae*** Kemperman & Wilkinson, 1985: 54 EP

***Stigmella costaricensis*** van Nieukerken & Nishida in van Nieukerken et al., 2016: 19 NEO

***Stigmella intronia*** van Nieukerken & Nishida in van Nieukerken et al., 2016: 21 NEO

### *Stigmella salicis* cluster

***Stigmella ogygia* group** (new) ^26^

***Stigmella aigialeia*** Donner & Wilkinson, 1989: 17 AUS

***Stigmella aliena*** Donner & Wilkinson, 1989: 17 AUS

***Stigmella atrata*** Donner & Wilkinson, 1989: 18 AUS

***Stigmella cassiniae*** Donner & Wilkinson, 1989: 18 AUS

***Stigmella childi*** Donner & Wilkinson, 1989: 19 AUS

***Stigmella cypracma*** (Meyrick, 1916) Dugdale, 1988: 53 AUS

*Nepticula cypracma* Meyrick, 1916c: 419

*Nepticula perissopa* Meyrick, 1919: 354 (syn: Donner & Wilkinson, 1989: 20)

*Stigmella perissopa* (Meyrick, 1919) Dugdale, 1988: 54

***Stigmella erysibodea*** Donner & Wilkinson, 1989: 21 AUS

***Stigmella fulva*** (Watt, 1921) Dugdale, 1988: 53 AUS

*Nepticula fulva* Watt, 1921: 215

***Stigmella hakekeae*** Donner & Wilkinson, 1989: 22 AUS

***Stigmella hamishella*** Donner & Wilkinson, 1989: 23 AUS

***Stigmella hoheriae*** Donner & Wilkinson, 1989: 24 AUS

***Stigmella ilsea*** Donner & Wilkinson, 1989: 25 AUS

***Stigmella insignis*** (Philpott, 1927) Dugdale, 1988: 53 AUS

*Nepticula insignis* Philpott, 1927: 89

***Stigmella kaimanua*** Donner & Wilkinson, 1989: 26 AUS

***Stigmella laquaeorum*** (Dugdale, 1971) Dugdale, 1988: 53 AUS

*Nepticula laquaeorum* Dugdale, 1971: 117

***Stigmella lucida*** (Philpott, 1919) Dugdale, 1988: 54 AUS

*Nepticula lucida* Philpott, 1919: 225

***Stigmella maoriella*** (Walker, 1864) Dugdale, 1988: 54 AUS

*Tinea maoriella* Walker, 1864: 1008

***Stigmella ogygia*** (Meyrick, 1889) Dugdale, 1988: 54 AUS

*Nepticula ogygia* Meyrick, 1889: 187

*Nepticula erechtitus* Watt, 1924: 686 (syn: Donner & Wilkinson, 1989: 20)

*Stigmella erechtitus* (Watt, 1924) Dugdale, 1988: 53

***Stigmella oriastra*** (Meyrick, 1917) Dugdale, 1988: 54 AUS

*Nepticula oriastra* Meyrick, 1917b: 247

***Stigmella palaga*** Donner & Wilkinson, 1989: 31 AUS

***Stigmella platina*** Donner & Wilkinson, 1989: 32 AUS

***Stigmella progama*** (Meyrick, 1924) Dugdale, 1988: 54 AUS

*Nepticula progama* Meyrick, 1924a: 662

***Stigmella progonopis*** (Meyrick, 1921) Dugdale, 1988: 54 AUS

*Nepticula progonopis* Meyrick, 1921c: 336

***Stigmella propalaea*** (Meyrick, 1889) Dugdale, 1988: 54 AUS

*Nepticula propalaea* Meyrick, 1889: 187

***Stigmella sophorae*** (Hudson, 1939) Dugdale, 1988: 54 AUS

*Nepticula sophorae* Hudson, 1939: 469

***Stigmella tricentra*** (Meyrick, 1889) Dugdale, 1988: 54 AUS

*Nepticula tricentra* Meyrick, 1889: 187

***Stigmella watti*** Donner & Wilkinson, 1989: 35 AUS

***Stigmella epicosma* group** (new) ^27^

***Stigmella andina*** (Meyrick, 1915) Davis, 1984: 18 NEO

*Nepticula andina* Meyrick, 1915a: 255

***Stigmella baccharicola*** Diškus & Stonis in Stonis et al., 2016b: 119 NEO

***Stigmella bipartita*** Diškus & Stonis in Stonis et al., 2016b: 107 NEO

***Stigmella confertae*** Diškus & Stonis in Stonis et al., 2016b: 124 NEO

***Stigmella costalimai*** (Bourquin, 1961) Davis, 1984: 18 ^28^ NEO

*Nepticula costalimai* Bourquin, 1961: 31

***Stigmella cuprata*** (Meyrick, 1915) Davis, 1984: 18 NEO

*Nepticula cuprata* Meyrick, 1915a: 255

***Stigmella emarginatae*** Diškus & Stonis in Stonis et al., 2016b: 104 NEO

***Stigmella epicosma*** (Meyrick, 1915) Davis, 1984: 18 NEO

*Nepticula epicosma* Meyrick, 1915a: 255

***Stigmella guittonae*** (Bourquin, 1961) Davis, 1984: 18 ^28^ NEO

*Nepticula guittonae* Bourquin, 1961: 32

***Stigmella hamata*** Puplesis & Robinson, 2000: 30 NEO

***Stigmella imperatoria*** Puplesis & Robinson, 2000: 30 NEO

***Stigmella johannis*** (Zeller, 1877) Davis, 1984: 18 NEO

*Nepticula johannis* Zeller, 1877: 454

***Stigmella latifoliae*** Remeikis, Diškus & Stonis in Stonis et al., 2016b: 115 NEO

***Stigmella marmorea*** Puplesis & Robinson, 2000: 26 NEO

***Stigmella mevia*** Remeikis & Stonis in Stonis & Remeikis, 2016: 311 NEO

***Stigmella montanotropica*** Puplesis & Diškus in Puplesis et al., 2002: 23 NEO

***Stigmella nubimontana*** Puplesis & Diškus in Puplesis et al., 2002: 24 NEO

***Stigmella olyritis*** (Meyrick, 1915) Davis, 1984: 18 NEO

*Nepticula olyritis* Meyrick, 1915a: 256

***Stigmella pangorica*** Diškus & Stonis in Stonis et al., 2015a: 580 NEO

***Stigmella peruanica*** Puplesis & Robinson, 2000: 27 NEO

***Stigmella podanthae*** Diškus & Stonis in Stonis et al., 2016a: 120 NEO

***Stigmella racemifera*** Šimkevičiūtė & Stonis in Šimkevičiūtė et al., 2009: 270 NEO

***Stigmella rubeta*** Puplesis & Diškus in Puplesis et al., 2002: 24 NEO

***Stigmella rudis*** Puplesis & Robinson, 2000: 26 NEO

***Stigmella schoorli*** Puplesis & Robinson, 2000: 29 NEO

***Stigmella serpentina*** Diškus & Stonis in Stonis et al., 2015a: 576 NEO

***Stigmella sinuosa*** Remeikis & Stonis in Stonis & Remeikis, 2016: 310 NEO

***Stigmella tripartita*** Diškus & Stonis in Stonis et al., 2016b: 110 NEO

***Stigmella salicis* group** (Johansson, 1971: 244)

*Stigmella fuscotibiella* group (Newton & Wilkinson, 1982: 385)

***Stigmella aiderensis*** Puplesis, 1988: 277 WP

***Stigmella arbusculae*** (Klimesch, 1951) Hering, 1957: 930 WP

*Nepticula arbusculae* Klimesch, 1951c: 149

***Stigmella assimilella*** (Zeller, 1848) Fletcher & Clutterbuck, 1945: 61 WP,EP

*Nepticula assimilella* Zeller, 1848: 327

*Nepticula tremulaefoliella* Sorhagen, 1922: 48 (syn: Johansson & Nielsen, 1990: 200)

*Stigmella tremulaefoliella* (Sorhagen, 1922) Gerasimov, 1952: 265

‡ *Lyonetia nigricornella* Mann in Zeller, 1848: 327 NN

***Stigmella benanderella*** (Wolff, 1955) Hering, 1957: 930 WP

*Nepticula benanderella* Wolff, 1955b: 49

‡ *Nepticula scandicella* Jonasson in Krogerus et al., 1971: 30 NN

***Stigmella flavescens*** Puplesis, 1994: 131 WP, EP

***Stigmella myrtillella*** (Stainton, 1857b) Vári, 1944a: 215 WP

*Nepticula myrtillella* Stainton, 1857b: 44

‡ Nepticula myrtillella var. uliginosi Skala, 1941b: 80 NNLM

***Stigmella obliquella*** (Heinemann, 1862) Vári, 1944a: 215 WP,EP

*Nepticula obliquella* Heinemann, 1862b: 316

*Nepticula wockeella* Heinemann, 1871: 223 (syn: Johansson & Nielsen, 1990: 198)

*Nepticula diversa* Glitz, 1872: 24 (syn: Glitz, 1887: 277)

*Stigmella babylonicae* Hartig, 1949: 94 (syn: van Nieukerken, 1986a: 11)

*Stigmella wockeella* (Heinemann, 1871) Gerasimov, 1952: 269

***Stigmella pallidiciliella*** Klimesch, 1948a: 165 ^29^ WP

*Nepticula purpureae* Skala, 1948: 121 (syn: van Nieukerken, 1986a: 11)

*Nepticula pallidiciliella* (Klimesch, 1948a) Wolff, 1955a: 86

***Stigmella salicis*** (Stainton, 1854) Fletcher & Clutterbuck, 1945: 60 ^30^ WP,EP,NEA

*Nepticula salicis* Stainton, 1854: 302

*Nepticula salicella* Herrich-Schäffer, 1855a: 354 UE

*Nepticula salicivorella* Doubleday, 1859: 36 UE

*Nepticula uniformis* Heinemann, 1871: 210 (syn: van Nieukerken, 1986a: 11)

*Nepticula dewitziella* Sorhagen, 1885: 285 (syn: Johansson & Nielsen, 1990: 192)

*Nepticula auritella* Skala, 1939f: 128 (syn: van Nieukerken, 1986a: 11)

*Stigmella libiezi* Dufrane, 1949: 8 (syn: van Nieukerken, 1986a: 11)

*Stigmella uniformis* (Heinemann, 1871) Gerasimov, 1952: 267

*Stigmella auritella* (Skala, 1939) Hering, 1957: 929

‡ Nepticula salicis ab. crombruggheella Dufrane, 1930: 30

‡ Nepticula salicis ab. februella Crombrugghe, 1907: 14

‡ Nepticula salicis ab. interrupta Skala, 1933a: 32

***Stigmella trimaculella*** (Haworth, 1828) Fletcher & Clutterbuck, 1945: 61 WP,EP

*Tinea trimaculella* Haworth, 1828: 583

*Lyonetia rufella* Zeller, 1839: 215 (syn: Herrich-Schäffer, 1855a: 358)

*Nepticula populella* Herrich-Schäffer, 1855a: 357 (syn: Frey, 1856: 381)

*Nepticula albicornella* Kollar in Nowicki, 1860: 231 (syn: Rebel, 1901: 228)

*Nepticula gilvella* Rössler, 1867: 395 (syn: van Nieukerken & Johansson, 1987: 461)

*Nepticula populicola* Sorhagen, 1922: 88 (syn: Skala, 1948: 121)

*Stigmella subtrimaculella* Dufrane, 1949: 10 (syn: Borkowski, 1969: 107)

*Microsetia trimaculella* (Haworth, 1828) Stephens, 1834: 269

*Nepticula trimaculella* (Haworth, 1828) Stainton, 1849: 29

*Nepticula rufella* (Zeller, 1839) Zeller, 1848: 328

*Stigmella populicola* (Sorhagen, 1922) Gerasimov, 1952: 252

‡ Nepticula trimaculella ab. semipictella Steudel in Steudel & Hoffmann, 1882: 244

***Stigmella vimineticola*** (Frey, 1856) Fletcher & Clutterbuck, 1945: 60 WP

*Nepticula vimineticola* Frey, 1856: 382

***Stigmella zelleriella*** (Snellen, 1875) van Nieukerken, 1983a: 60 WP

*Nepticula zelleriella* Snellen, 1875: 116

*Nepticula repentiella* Wolff, 1955a: 82 (syn: van Nieukerken, 1983a: 60)

*Nepticula lappovimella* Svensson, 1976: 204 (syn: van Nieukerken, 1983a: 60)

*Stigmella repentiella* (Wolff, 1955) Hering, 1957: 929

*Stigmella lappovimella* (Svensson, 1976) Svensson, 1985: 78

***Stigmella azusa*** Hirano, 2010: 129 EP

***Stigmella johanssoni*** Puplesis & Diškus, 1996c: 181 EP

***Stigmella juratae*** Puplesis, 1988: 279 EP

***Stigmella kondarai*** Puplesis, 1988: 277 EP

***Stigmella sexcornuta*** Rocienė & Stonis in Stonis & Rocienė, 2014: 205 EP

***Stigmella tenryuensis*** Hirano, 2014: 26 EP

***Stigmella tranocrossa*** Kemperman & Wilkinson, 1985: 27 EP

*Stigmella ussurica* Puplesis, 1987: 8 (syn: Puplesis, 1994: 128)

***Stigmella vittata*** Kemperman & Wilkinson, 1985: 28 EP

***Stigmella fibigeri*** Puplesis & Diškus, 2003a: 209 OR

***Stigmella aromella*** Wilkinson & Scoble, 1979: 27 NEA

***Stigmella fuscotibiella*** (Clemens, 1862) Wilkinson & Scoble, 1979: 23 NEA

*Nepticula fuscotibiella* Clemens, 1862: 133

*Nepticula ciliaefuscella* Chambers, 1873: 128 (syn: Chambers, 1875a: 117)

*Nepticula discolorella* Braun, 1912: 86 (syn: Braun, 1917: 185)

***Stigmella pallida*** (Braun, 1912) Newton & Wilkinson, 1982: 418 NEA

*Nepticula pallida* Braun, 1912: 85

***Stigmella populetorum*** (Frey & Boll, 1878) Wilkinson & Scoble, 1979: 26 NEA

*Nepticula populetorum* Frey & Boll, 1878: 276

***Stigmella molinensis*** van Nieukerken & Snyers in van Nieukerken et al., 2016: 22 NEO

### *Stigmella quercipulchella*/*anomalella*/*oxyacanthella* cluster

***Stigmella quercipulchella* group** (Wilkinson & Scoble, 1979: 65)

***Stigmella altella*** (Braun, 1914) Wilkinson & Newton, 1981: 58 NEA

*Nepticula altella* Braun, 1914: 21

***Stigmella quercipulchella*** (Chambers, 1882) Wilkinson & Scoble, 1979: 65 NEA

*Nepticula quercipulchella* Chambers, 1882: 105

*Nepticula terminella* Braun, 1914: 23 (syn: Wilkinson & Scoble, 1979: 65)

***Stigmella variella*** (Braun, 1910) Wilkinson & Scoble, 1979: 67 NEA

*Nepticula variella* Braun, 1910: 173

***Stigmella guatemalensis*** Diškus & Stonis in Stonis et al., 2013c: 10 NEO

***Stigmella anomalella* group** (Johansson, 1971: 245)

***Stigmella anomalella*** (Goeze, 1783) Walsingham, 1908a: 1008 ^2^ WP,EP,[NEA]

*Phalaena anomalella* Goeze, 1783: 168

*Phalaena grisearosae* Retzius, 1783: 55

*Tinea penicilla* Thunberg, 1794: 88 (syn: Karsholt & Nielsen, 1986: 452)

*Tinea rosella* Schrank, 1802: 139 (syn: Stainton, 1854: 297)

*Nepticula aeneella* Heinemann, 1862a: 254 (syn: Schoorl et al., 1985: 98)

*Nepticula fletcheri* Tutt, 1899: 211 (syn: Krogerus, 1971: 30)

*Nepticula laticuniculella* Sauber, 1904: 55 (syn: Hering, 1957: 902)

*Stigmella rubicurrens* Walsingham, 1908a: 1009 ^2^ (syn: van Nieukerken & Johansson, 1987: 461)

*Nepticula rosarum* Sorhagen, 1922: 30 (syn: van Nieukerken & Johansson, 1987: 470)

*Nepticula zermattensis* Weber, 1936: 668 (syn: van Nieukerken, 1986a: 9)

*Nepticula helbigi* Hartig, 1941: 160 (syn: van Nieukerken & Johansson, 1987: 468)

*Stigmella caulescentella* Klimesch, 1948a: 162 ^29^ (syn: van Nieukerken, 1986a: 9)

*Stigmella anomalella pacifica* Puplesis, 1987: 10

*Nepticula anomalella* (Goeze, 1783) Stainton, 1854: 297

*Dysnepticula anomalella* (Goeze, 1783) Börner, 1925: 370

*Nepticula rosella* (Schrank, 1802) Sand, 1879: 200

*Stigmella rosella* (Schrank, 1802) Walsingham, 1908a: 1008^1^

*Stigmella fletcheri* (Tutt, 1899) Fletcher & Clutterbuck, 1945: 59

*Nepticula rubicurrens* (Walsingham, 1908a) Rebel, 1910: 373

*Stigmella rosarum* (Sorhagen, 1922) Gerasimov, 1952: 256

*Stigmella zermattensis* (Weber, 1936) Gerasimov, 1952: 270

*Stigmella anomalella fletcheri* (Tutt, 1899) Hering, 1957: 902

*Stigmella anomalella helbigi* (Hartig, 1941) Hering, 1957: 902

***Stigmella centifoliella*** (Zeller, 1848) Beirne, 1945: 199 ^31^ WP,[NEA]

‡ *Nepticula centifoliella* Heyden, 1843: 208 NN

*Nepticula centifoliella* Zeller, 1848: 315

*Nepticula hodgkinsoni* Stainton, 1884: 103 (syn: Bradley et al., 1972: 2)

*Stigmella rosaefoliella pectocatena* Wilkinson & Scoble, 1979: 18 **syn. n.** ^31^

*Stigmella hodgkinsoni* (Stainton, 1884) Gerasimov, 1952: 243

***Stigmella spinosissimae*** (Waters, 1928) Beirne, 1945: 198 WP,EP

*Nepticula spinosissimae* Waters, 1928: 105

***Stigmella hybnerella* group** (Johansson, 1971: 245)

*Stigmella nitidella* group (Johansson, 1971: 245)

*Stigmella paradoxa* group (Emmet, 1976: 238)

*Stigmella irregularis* group (Puplesis, 1994: 61)

***Stigmella hybnerella*** (Hübner, 1796) Fletcher & Clutterbuck, 1945: 59 WP

*Tinea hybnerella* Hübner, 1796: pl. 34: 236

*Caloptilia ampelipennella* Hübner, [1825]: 427 URN

*Tinea posticella* Haworth, 1828: 584 (syn: Stainton, 1849: 29)

*Oecophora gratiosella* Duponchel, 1843: 323 (syn: Fletcher & Clutterbuck, 1945: 59)

*Nepticula ignobilella* Stainton, 1849: 29 (syn: Emmet, 1974c: 77)

*Nepticula latifasciella* Herrich-Schäffer, 1855a: 352 (syn: Herrich-Schäffer, 1855a: 352)

*Microsetia posticella* (Haworth, 1828) Stephens, 1834: 269

*Lyonetia hübnerella* (Hübner, 1796) Zeller, 1839: 215

*Nepticula gratiosella* (Duponchel, 1843) Stainton, 1849: 29

*Lithocolletis gratiosella* (Duponchel, 1843) Bruand, [1851]: 86

*Stigmella gratiosella* (Duponchel, 1843) Beirne, 1945: 200

*Stigmella ignobilella* (Stainton, 1849) Fletcher & Clutterbuck, 1945: 60

*Nepticula ignobiliella* auct. ISS

***Stigmella mespilicola*** (Frey, 1856) Klimesch, 1948b: 57 (Fig. 26) WP

*Nepticula mespilicola* Frey, 1856: 392

*Nepticula ariella* Herrich-Schäffer, 1860: 60 (syn: Frey, 1880: 422)

*Stigmella ariella* (Herrich-Schäffer, 1860) Klimesch, 1948b: 57

***Stigmella irregularis*** Puplesis, 1994: 61 WP

***Stigmella inopinata*** A. Laštuvka & Z. Laštuvka, 1990: 197 WP

***Stigmella paradoxa*** (Frey, 1858) Emmet, 1970: 3 WP

*Nepticula paradoxa* Frey, 1858a: 14

*Nepticula nitidella* Heinemann, 1862a: 257 (syn: Heinemann & Wocke, [1876]: 734) ^6^

*Stigmella juryi* Puplesis, 1991: 125 (syn: van Nieukerken et al., 2004a: 140)

*Stigmella nitidella* (Heinemann, 1862) Gerasimov, 1952: 249

***Stigmella pyrivora*** Gustafsson, 1981: 457 WP

***Stigmella malifoliella*** Puplesis in Puplesis & Arutyunova, 1991: 571 EP

***Stigmella montana*** Puplesis, 1991: 126 EP

***Stigmella taeniola*** (Braun, 1925) Newton & Wilkinson, 1982: 382 NEA

*Nepticula taeniola* Braun, 1925b: 226

***Stigmella stigmaciella*** Wilkinson & Scoble, 1979: 38 NEA

***Stigmella incognitella* group** (new)

*Stigmella pomella* group (Johansson, 1971: 244)

***Stigmella azaroli*** (Klimesch, 1978) van Nieukerken, 1986a: 13 ^32^ WP

*Nepticula azaroli* Klimesch, 1978b: 261

***Stigmella fuscacalyptriella*** Puplesis, 1994: 149 WP

***Stigmella incognitella*** (Herrich-Schäffer, 1855) van Nieukerken, 1986a: 13 WP

*Nepticula incognitella* Herrich-Schäffer, 1855a: 349

*Nepticula pomella* Vaughan, 1858: 43 (syn: van Nieukerken, 1986a: 13)

*Nepticula mali* Hering, 1932: 568 (syn: van Nieukerken, 1986a: 13)

*Stigmella mali* (Hering, 1932) Gerasimov, 1952: 247

*Stigmella pomella* (Vaughan, 1858) Fletcher & Clutterbuck, 1945: 58

***Stigmella perpygmaeella*** (Doubleday, 1859) Karsholt & Nielsen, 1976: 18 ^32^ WP

*Tinea pygmaeella* Haworth, 1828: 586; JPH of *Tinea pygmaeella* [Denis & Schiffermüller], 1775), now *Argyresthia pygmaeella* (Argyresthiidae)

*Nepticula perpygmaeella* Doubleday, 1859: 36; RN for *Tinea pygmaeella* Haworth, 1828

*Microsetia pygmaeella* (Haworth, 1828) Stephens, 1834: 269

*Nepticula pygmaeella* (Haworth, 1828) Stainton, 1853: 3958

*Stigmella pygmaeella* (Haworth, 1828) Klimesch, 1951b: 55

***Stigmella elegantiae*** Puplesis & Diškus, 2003a: 210 OR

***Stigmella oxyacanthella* group** (Johansson, 1971: 245)

*Stigmella crataegifoliella* group (Wilkinson & Scoble, 1979: 29)

***Stigmella caspica*** Puplesis, 1994: 109 WP

***Stigmella crataegella*** (Klimesch, 1936) Klimesch, 1948b: 62 WP

*Nepticula crataegella* Klimesch, 1936: 200

*Stigmella indigena* Puplesis, 1994: 111 (syn: Puplesis et al., 1996: 192)

*Nepticula gratiosella* sensu Tutt, 1899: 253

***Stigmella desperatella*** (Frey, 1856) Beirne, 1945: 200 WP

*Nepticula desperatella* Frey, 1856: 374

*Nepticula pyricola* Wocke, 1877: 49 (syn: Schoorl et al., 1985: 94)

*Stigmella pyricola* (Wocke, 1877) Klimesch, 1951b: 57

***Stigmella hahniella*** (Wörz, 1937) Gerasimov, 1952: 241 WP

*Nepticula hahniella* Wörz, 1937: 290

***Stigmella lanceolata*** Puplesis, 1994: 111 WP

***Stigmella magdalenae*** (Klimesch, 1950) Emmet, 1979: 25 ^33^ WP

Nepticula nylandriella var. magdalenae Klimesch, 1950b: 72

*Nepticula nylandriella* auct. [misapplied, before 1972] (syn: Borkowski, 1975: 523)

*Stigmella nylandriella magdalenae* (Klimesch, 1950b) Klimesch, 1961: 752

*Nepticula magdalenae* Klimesch, 1950b (Borkowski, 1975: 523)

***Stigmella minusculella*** (Herrich-Schäffer, 1855) Beirne, 1945: 198 WP,[NEA]

*Nepticula minusculella* Herrich-Schäffer, 1855a: 348

*Nepticula chalybeia* Braun, 1914: 20 (syn: Schoorl et al., 1985: 92)

*Nepticula embonella* Klimesch, 1978b: 259 (syn: Schoorl et al., 1985: 92)

*Stigmella chalybeia* (Braun, 1914) Wilkinson & Scoble, 1979: 35

***Stigmella nylandriella*** (Tengström, 1848) Beirne, 1945: 200 ^34^ WP

*Lyonetia nylandriella* Tengström, 1848: 152

*Nepticula aucupariae* Frey, 1857: 376 (syn: Borkowski, 1975: 522)

*Nepticula aucupariella* Porritt, 1883: 172 UE

*Nepticula nylandriella* (Tengström, 1848) Herrich-Schäffer, 1855: 359 ^34^

*Stigmella aucupariae* (Frey, 1857) Beirne, 1945: 199

***Stigmella oxyacanthella*** (Stainton, 1854) Beirne, 1945: 200 ^35^ WP,[NEA]

*Nepticula oxyacanthella* Stainton, 1854: 298

*Nepticula oxyacanthaecolella* Doubleday, 1859: 298 URN

*Micropteryx pomivorella* Packard, 1870: 237 **syn. n.** ^35^

*Nepticula cotoneastri* Sorhagen, 1922: 42 (syn: Schoorl et al., 1985: 87)

*Nepticula aeneella* auct. [misapplied, before 1985]

*Stigmella aeneella* auct. [misapplied, before 1985]

*Nepticula pomivorella* (Packard, 1870) Busck, 1901: 52

*Stigmella pomivorella* (Packard, 1870) Wilkinson & Scoble, 1979: 33

*Stigmella cotoneastri* (Sorhagen, 1922) Klimesch, 1948b: 60

‡ *Nepticula chaenomelis* Skala, 1936: 79 NNLM (syn: van Nieukerken, 1986a: 10)

‡ Nepticula oxyacanthella var. mespili Skala, 1940: 144 NNLM

‡ Nepticula oxyacanthella var. oxymalella Skala, 1933b: 130 NNLM

‡ Nepticula oxyacanthella var. oxysorbi Skala, 1933b: 130 NNLM

‡ *Stigmella chaenomelis* (Skala, 1936) Hering, 1957: 275

***Stigmella pyri*** (Glitz, 1865) Vári, 1944a: 214 WP

*Nepticula pyri* Glitz, 1865: 42

***Stigmella regiella*** (Herrich-Schäffer, 1855) Vári, 1944a: 214 WP

*Nepticula regiella* Herrich-Schäffer, 1855a: 351

*Nepticula corvimontana* Hering, 1935: 6 (syn.: Borkowski, 1969: 103)

*Stigmella corvimontana* (Hering, 1935) Gerasimov, 1952: 234

***Stigmella stettinensis*** (Heinemann, 1871) Gerasimov, 1952: 262 WP

*Nepticula stettinensis* Heinemann, 1871: 210

***Stigmella torminalis*** (Wood, 1890) Beirne, 1945: 200 WP

*Nepticula torminalis* Wood, 1890: 209

***Stigmella alaurulenta*** Kemperman & Wilkinson, 1985: 23 EP

***Stigmella aurora*** Puplesis, 1984a: 119 WP,EP

***Stigmella chaenomelae*** Kemperman & Wilkinson, 1985: 23 EP

***Stigmella crataegi*** Gerasimov, 1937: 283 EP

***Stigmella hissariella*** Puplesis, 1994: 112 EP

***Stigmella honshui*** Kemperman & Wilkinson, 1985: 24 EP

***Stigmella micromelis*** Puplesis, 1985c: 59 ^36^ EP

*Stigmella crataegivora* Puplesis, 1985c: 60 **syn. n.** ^36^

***Stigmella nostrata*** Puplesis, 1984a: 113 EP

***Stigmella sorbivora*** Kemperman & Wilkinson, 1985: 25 EP

***Stigmella zumii*** Kemperman & Wilkinson, 1985: 26 EP

***Stigmella amelanchierella*** (Clemens, 1861) Davis & Wilkinson, 1983: 3 ^37^ NEA

*Nepticula amelanchierella* Clemens, 1861: 84

***Stigmella crataegifoliella*** (Clemens, 1861) Wilkinson & Scoble, 1979: 30 NEA

*Nepticula crataegifoliella* Clemens, 1861: 83

***Stigmella heteromelis*** Newton & Wilkinson, 1982: 405 NEA

***Stigmella purpuratella*** (Braun, 1917) Newton & Wilkinson, 1982: 381 ^38^ NEA

*Nepticula purpuratella* Braun, 1917: 176

*Stigmella scinanella* Wilkinson & Scoble, 1979: 36 **syn. n.** ^38^

***Stigmella scintillans*** (Braun, 1917) Wilkinson & Scoble, 1979: 33 NEA

*Nepticula scintillans* Braun, 1917: 167

***Stigmella argentifasciella* group** (new)

***Stigmella argentifasciella*** (Braun, 1912) Newton & Wilkinson, 1982: 453 NEA

*Nepticula argentifasciella* Braun, 1912: 100

***Stigmella aurella*/*ruficapitella* cluster** ^39^

***Stigmella styracicolella* group** (new)

***Stigmella styracicolella*** (Klimesch, 1978) van Nieukerken, 1986a: 14 WP

*Nepticula styracicolella* Klimesch, 1978b: 267

***Stigmella egonokii*** Kemperman & Wilkinson, 1985: 53 EP

***Stigmella speciosa* group** (new) ^39^

***Stigmella kuznetzovi*** Puplesis, 1994: 152 WP

***Stigmella speciosa*** (Frey, 1858) Walsingham, 1916: 159 WP

*Nepticula speciosa* Frey, 1858b: 27

*Nepticula pseudoplatanella* Weber, 1936: 671 (syn: Borkowski, 1969: 98)

*Stigmella pseudoplatanella* (Weber, 1936) Gerasimov, 1952: 254

‡ Nepticula aceris var. pseudoplatanella Skala, 1933b: 132 NNLM

‡ Nepticula speciosa var. monspessulani Skala, 1939d: 144 NNLM

***Stigmella lonicerarum*** (Frey, 1857) Gerasimov, 1952: 246 WP

*Nepticula lonicerarum* Frey, 1857: 383

Nepticula lonicerarum var. lentinensis Skala, 1935: 79 (syn: van Nieukerken, 1986a: 13)

Nepticula lonicerarum var. livonica Skala, 1935: 79 (syn: van Nieukerken, 1986a: 13)

Nepticula lonicerarum var. teutonica Skala, 1935: 79 (syn: van Nieukerken, 1986a: 13)

***Stigmella monticulella*** Puplesis, 1984a: 114 ^40^ EP

*Stigmella gracilipae* Hirano, 2014: 22 **syn. n.** ^40^

***Stigmella aurella* group** (Johansson, 1971: 243) ^39^

*Stigmella lediella* group (Puplesis, 1984b:583)

***Stigmella aeneofasciella*** (Herrich-Schäffer, 1855) Gerasimov, 1952: 222 WP

*Nepticula aeneofasciella* Herrich-Schäffer, 1855a: 353

*Nepticula aeneofasciata* Frey, 1856: 376 UE

***Stigmella aurella*** (Fabricius, 1775) Walsingham, 1908a: 1009 ^2^ WP

*Tinea aurella* Fabricius, 1775: 666

*Nepticula fragariella* Heinemann, 1862a: 263 (syn: Borkowksi, 1975: 503)

*Nepticula nitens* Fologne, 1862: 164 (syn: Klimesch, 1981: 114)

*Nepticula gei* Wocke, 1871: 336 (syn: Borkowksi, 1975: 503)

*Nepticula albicomella* Heinemann & Wocke, [1876]: 748 ^6^ (syn: Johansson & Nielsen, 1990: 207)

*Nepticula fruticosella* Müller-Rutz in Vorbrodt & Müller-Rutz, 1914: 591 (syn: van Nieukerken, 1986a: 12)

*Microsetia aurella* (Fabricius, 1775) Stephens, 1834: 268

*Nepticula aurella* (Fabricius, 1775) Heyden, 1843: 209

*Stigmella fragariella* (Heinemann, 1862a) Vári, 1944a: 214

*Stigmella nitens* (Fologne, 1862) Vári, 1944a: 214

*Stigmella gei* (Wocke, 1871) Gerasimov, 1952: 240

*Stigmella fruticosella* (Müller-Rutz in Vorbrodt & Müller-Rutz, 1914) Gerasimov, 1952: 240

‡ Nepticula gei ab. semicolorella Eppelsheim, 1891: 351 NNLM

‡ Nepticula gei var. geirubi Skala, 1940: 143 NNLM

***Stigmella auromarginella*** (Richardson, 1890) Gerasimov, 1952: 229 WP

*Nepticula auromarginella* Richardson, 1890: 30

***Stigmella dryadella*** (Hofmann, 1868) Klimesch, 1951b: 58 WP

*Nepticula dryadella* Hofmann, 1868: 29

***Stigmella filipendulae*** (Wocke, 1871) Gerasimov, 1952: 238 ^41^ WP,EP

*Nepticula filipendulae* Wocke, 1871: 338

*Nepticula ulmariae* Wocke, 1879: 79 (syn: van Nieukerken et al., 2012a: 250)

*Stigmella palmatae* Puplesis, 1984a: 115 **syn. n.** ^41^

*Stigmella ulmariae* (Wocke, 1879) Gerasimov, 1952: 266

***Stigmella geimontani*** (Klimesch, 1940) Klimesch, 1961: 754 WP

*Nepticula geimontani* Klimesch, 1940b: 89

***Stigmella lediella*** (Schleich, 1867) Gerasimov, 1952: 245 ^42^ WP,EP

*Nepticula lediella* Schleich, 1867: 449

*Stigmella magica* Puplesis, 1985c: 63 (syn: Puplesis, 1994: 146)

*Stigmella rhododendri* Puplesis, 1985c: 65 (syn: Puplesis, 1994: 146)

*Stigmella sesplicata* Kemperman & Wilkinson, 1985: 36 **syn. n.** ^42^

‡ Nepticula lediella ab. auromarginata Petersen, 1930

‡ *Stigmella rhododendrifolia* Dovnar-Zapolski & Tomilova, 1978: 29 NNLM; **syn. n.** ^42^

***Stigmella poterii*** (Stainton, 1857) Fletcher & Clutterbuck, 1945: 59 WP

*Nepticula poterii* Stainton, 1857c: 116

*Nepticula poteriella* Doubleday, 1859: 36 UE

*Nepticula comari* Wocke, 1862: 253 (syn: Borkowksi, 1975: 506)

*Nepticula geminella* Frey, 1870: 288 (syn: Karsholt & Nielsen, 1976: 17)

*Nepticula palustrella* Frey, 1870: 287 (syn: van Nieukerken, 1986a: 12)

*Nepticula occultella* Heinemann, 1871: 215 (syn: Borkowksi, 1975: 506)

*Nepticula tengströmi* Nolcken, 1871: 776 (syn: Borkowksi, 1975: 506)

*Nepticula potentillae* Glitz, 1872: 24 (syn with *occultella*: Heinemann &Wocke, [1876]: 749)

*Nepticula diffinis* Wocke, 1874: 100 (syn: Borkowksi, 1975: 506)

*Nepticula serella* Stainton, 1888a: 260 (syn: Borkowksi, 1975: 506)

*Nepticula elisabethella* Szőcs, 1957: 321 (syn: van Nieukerken, 1986a: 12)

*Stigmella comari* (Wocke, 1862) Gerasimov, 1952: 234

*Stigmella geminella* (Frey, 1870) Gerasimov, 1952: 240

*Stigmella occultella* (Heinemann, 1871) Gerasimov, 1952: 250

*Stigmella tengstroemi* (Nolcken, 1871) Gerasimov, 1952: 263

*Stigmella diffinis* (Wocke, 1874) Gerasimov, 1952: 236

*Stigmella serella* (Stainton, 1888a) Gerasimov, 1952: 259

***Stigmella pretiosa*** (Heinemann, 1862) Gerasimov, 1952: 253 WP

*Nepticula pretiosa* Heinemann, 1862a: 261

*Nepticula bollii* Frey, 1873: 144 (syn: van Nieukerken, 1986a: 12)

*Stigmella geimontani tatrensis* Borkowski, 1970b: 546 (syn: Borkowksi, 1975: 507)

*Stigmella bollii* (Frey, 1873) Gerasimov, 1952: 231

***Stigmella splendidissimella*** (Herrich-Schäffer, 1855) Klimesch, 1951b: 58 WP

*Nepticula splendidissimella* Herrich-Schäffer, 1855a: 353

*Nepticula splendidissima* Frey, 1856: 393 UE

*Nepticula dulcella* Heinemann, 1862a: 267 (syn: Karsholt & Nielsen, 1976: 17)

*Nepticula inaequalis* Heinemann, 1862b: 302 (syn: Johansson & Nielsen, 1990: 209)

*Nepticula fragarivora* Carolsfeld-Krause, 1944: 158 (syn: Karsholt & Nielsen, 1976: 17)

*Stigmella dulcella* (Heinemann, 1862a) Gerasimov, 1952: 237

*Stigmella inaequalis* (Heinemann, 1862b) Gerasimov, 1952: 244

*Stigmella fragarivora* (Carolsfeld-Krause, 1944) Hering, 1957: 455

‡ *Nepticula peterseniella* Skala, 1941b: 78 NN (syn: van Nieukerken, 1986a: 12)

‡ *Stigmella fragarivora peterseniella* (Skala, 1941b) Hering, 1957: 455

***Stigmella stelviana*** (Weber, 1938) Klimesch, 1948b: 67 WP

‡ *Nepticula stelviana* Wocke, 1881: 205 NN

*Nepticula stelviana* Weber, 1938: 5

*Nepticula crantziella* Weber, 1945: 401 (syn: Klimesch, 1950a: 27)

*Stigmella crantziella* (Weber, 1945) Klimesch, 1948b: 69

***Stigmella tormentillella*** (Herrich-Schäffer, 1860) Gerasimov, 1952: 264 WP

*Nepticula tormentillella* Herrich-Schäffer, 1860: 60

***Stigmella acrochaetia*** Kemperman & Wilkinson, 1985: 31 EP

***Stigmella alikurokoi*** Kemperman & Wilkinson, 1985: 31 EP

***Stigmella ichigoiella*** Kemperman & Wilkinson, 1985: 35 EP

***Stigmella longispina*** Puplesis, 1994: 166 ^43^ WP,EP

***Stigmella spiculifera*** Kemperman & Wilkinson, 1985: 37 ^44^ EP

*Stigmella oa* Kemperman & Wilkinson, 1985: 52 **syn. n.** ^44^

***Stigmella villosella*** (Clemens, 1861) Newton & Wilkinson, 1982: 410 NEA

*Nepticula villosella* Clemens, 1861: 84

*Nepticula dallasiana* Frey & Boll, 1876: 288 (syn: Braun, 1917: 174)

***Stigmella sorbi* group** (Johansson, 1971: 244) ^36^

*Stigmella amygdali* group (van Nieukerken, 1986a: 13)

***Stigmella amygdali*** (Klimesch, 1978) van Nieukerken, 1986a: 13 WP

*Nepticula amygdali* Klimesch, 1978b: 264

***Stigmella cerasi*** Puplesis & Diškus, 1996b: 178 WP

***Stigmella plagicolella*** (Stainton, 1854) Fletcher & Clutterbuck, 1945: 60 WP

*Nepticula plagicolella* Stainton, 1854: 303

‡ Nepticula plagicolella var. avianella Skala, 1934c: 30 NNLM

‡ *Stigmella plagicolella avianella* (Skala, 1934c) Hering, 1957: 839 NNLM

***Stigmella sorbi*** (Stainton, 1861) Fletcher & Clutterbuck, 1945: 60 WP,EP

*Nepticula sorbi* Stainton, 1861: 91

*Nepticula sorbiella* Porritt, 1883: 171 UE

Nepticula sorbi var. cotoneastrella Weber, 1936: 670

*Stigmella cotoneastrella* (Weber, 1936) Hering, 1957: 338

‡ Nepticula plagicolella var. malicola Skala, 1939d: 95 NNLM

‡ *Stigmella plagicolella malicola* (Skala, 1939) Hering, 1957: 664 NNLM

***Stigmella aflatuniae*** Puplesis & Diškus, 1996b: 180 EP

***Stigmella azukinashii*** Hirano, 2014: 25 EP

***Stigmella hamamelella*** Hirano, 2014: 20 EP

***Stigmella lurida*** Puplesis, 1994: 132 ^45^ EP

***Stigmella motiekaitisi*** Puplesis, 1994: 135 WP,EP

***Stigmella pourthiaeella*** Hirano, 2014: 24 EP

***Stigmella subsorbi*** Puplesis, 1994: 134 EP

***Stigmella tenebrica*** Puplesis & Diškus, 2003a: 214 OR

***Stigmella lemniscella* group** (new) ^39^

*Stigmella marginicolella* group (Johansson, 1971: 243)

***Stigmella continuella*** (Stainton, 1856) Fletcher & Clutterbuck, 1945: 59 WP,EP

*Nepticula continuella* Stainton, 1856: 42

*Stigmella uigurica* Puplesis, 1985c: 62 (syn: Puplesis, 1994: 137)

***Stigmella lemniscella*** (Zeller, 1839) van Nieukerken, 1986a: 11 WP

*Lyonetia lemniscella* Zeller, 1839: 215

*Nepticula marginicolella* Stainton, 1853: 3958 (syn: van Nieukerken, 1986a: 12)

*Nepticula suberosella* Toll, 1934b: 76 (syn: Hering, 1957: 1089)

*Nepticula fulvomacula* Skala, 1936: 79 (syn: Borkowski, 1969: 114)

*Nepticula lemniscella* (Zeller, 1839) Zeller, 1848: 313

*Stigmella marginicolella* (Stainton, 1853) Fletcher & Clutterbuck, 1945: 59

*Stigmella fulvomacula* (Skala, 1936) Gerasimov, 1952: 239

***Stigmella zagulaevi*** Puplesis, 1994: 139 WP

***Stigmella gimmonella*** (Matsumura, 1931) Kuroko, 1982a: 448 EP

*Nepticula gimmonella* Matsumura, 1931: 1114

***Stigmella talassica*** Puplesis in Puplesis et al., 1992: 54 EP

***Stigmella apicialbella*** (Chambers, 1873) Newton & Wilkinson, 1982: 413 NEA

*Nepticula apicialbella* Chambers, 1873: 127

*Nepticula leucostigma* Braun, 1912: 88 (syn: Braun, 1914: 21)

***Stigmella floslactella* group** (Johansson, 1971: 244) ^39^

***Stigmella carpinella*** (Heinemann, 1862) Gerasimov, 1952: 232 WP

*Nepticula carpinella* Heinemann, 1862a: 251

***Stigmella floslactella*** (Haworth, 1828) Fletcher & Clutterbuck, 1945: 61 WP

*Tinea floslactella* Haworth, 1828: 585

*Nepticula saxatilella* Grönlien, 1932: 114 (syn: van Nieukerken & Johansson, 1987: 461)

*Microsetia floslactella* (Haworth, 1828) Stephens, 1834: 268

*Nepticula floslactella* (Haworth, 1828) Stainton, 1849: 29

*Stigmella saxatilella* (Grönlien, 1932) Gerasimov, 1952: 258

‡ Stigmella floslactella f. interrupta Dufrane, 1949: 10

***Stigmella johanssonella*** A. Laštuvka & Z. Laštuvka, 1997: 70 WP

***Stigmella tityrella*** (Stainton, 1854) Hering, 1957: 439 WP

*Nepticula tityrella* Stainton, 1854: 304

*Nepticula turicella* Herrich-Schäffer, 1855a: 355 (syn: Carolsfeld-Krausé, 1949: 310)

*Nepticula turicensis* Frey, 1856: 391 UE

*Nepticula castanella* Stainton, 1859a: 123 (syn: van Nieukerken & Johansson, 1987: 461)

*Nepticula hemargyrella* auct. [misapplied] (syn: Carolsfeld-Krausé, 1949: 304)

*Stigmella turicella* (Herrich-Schäffer, 1855a) Fletcher & Clutterbuck, 1945: 60

*Stigmella castanella* (Stainton, 1859a) Gerasimov, 1952: 232

***Stigmella ruficapitella* group - s.l.** (Johansson, 1971: 245) ^39^

*Stigmella hemargyrella* group (Johansson, 1971: 244)

*Stigmella procrastinella* group (Wilkinson & Scoble, 1979: 69)

*Stigmella castanopsiella* group (Puplesis, 1984b: 583)

***Stigmella hemargyrella*** (Kollar, 1832) Gerasimov, 1952: 242 WP

*Oecophora hemargyrella* Kollar, 1832: 98

*Nepticula basalella* Herrich-Schäffer, 1855a: 354 (syn: Carolsfeld-Krausé, 1949: 310)

*Nepticula fagella* Herrich-Schäffer, 1855a: 354 (syn: van Nieukerken & Johansson, 1987: 461)

*Nepticula fagi* Frey, 1856: 384 (syn: van Nieukerken & Johansson, 1987: 461)

*Nepticula nobilella* Heinemann & Wocke, [1876]: 755 ^6^ (syn: van Nieukerken & Johansson, 1987: 461)

*Nepticula fulgens* Stainton, 1888b: 12 (syn: Carolsfeld-Krausé, 1949: 310)

*Nepticula tityrella* auct. [misapplied] (see: Carolsfeld-Krausé, 1949: 307)

*Lyonetia hemargyrella* (Kollar, 1832) Zeller, 1839: 215

*Nepticula hemargyrella* (Kollar, 1832) Zeller, 1848: 323

*Stigmella basalella* (Herrich-Schäffer, 1855) Fletcher & Clutterbuck, 1945: 60

***Stigmella castanopsiella*** (Kuroko, 1978) Kuroko, 1982a: 448 EP

*Nepticula castanopsiella* Kuroko, 1978: 1

***Stigmella kurokoi*** Puplesis, 1984b: 594 EP

*Stigmella valvaurigemmata* Kemperman & Wilkinson, 1985: 45 (syn: Puplesis, 1994: 161)

***Stigmella lithocarpella*** van Nieukerken & Liu, 2000: 169 EP

***Stigmella vandrieli*** van Nieukerken & Liu, 2000: 171 EP

***Stigmella circumargentea*** van Nieukerken & Liu, 2000: 165 EP

***Stigmella kao*** van Nieukerken & Liu, 2000: 166 EP,OR

***Stigmella alba*** Wilkinson & Scoble, 1979: 73 NEA

***Stigmella procrastinella*** (Braun, 1927) Wilkinson & Scoble, 1979: 70 NEA

*Nepticula procrastinella* Braun, 1927: 59

***Stigmella humboldti*** Remeikis & Stonis, 2015: 412 ^46^ NEO

***Stigmella ruficapitella* group - s.s.** (Johansson, 1971: 245) ^39^

*Stigmella atricapitella* group (Emmet, 1976: 239)

*Stigmella caesurifasciella* group (Kemperman & Wilkinson, 1985: 38)

*Stigmella suberivora* group (Kemperman & Wilkinson, 1985: 38)

***Stigmella atricapitella*** (Haworth, 1828) Beirne, 1945: 198 WP

*Tinea atricapitella* Haworth, 1828: 585

*Nepticula discrepans* Sorhagen, 1922: 41 (syn: van Nieukerken & Johansson, 1987: 461)

*Microsetia atricapitella* (Haworth, 1828) Stephens, 1834: 269

*Nepticula atricapitella* (Haworth, 1828) Stainton, 1849: 28

*Stigmella discrepans* (Sorhagen, 1922) Gerasimov, 1952: 237

***Stigmella basiguttella*** (Heinemann, 1862) Vári, 1944b: xxv WP

*Nepticula basiguttella* Heinemann, 1862a: 258

*Stigmella cerricolella* Klimesch, 1948a: 160 ^29^ (syn: Johansson, 1971: 253)

*Nepticula cerricolella* (Klimesch, 1948) Johansson, 1971: 253

*Nepticula basiguttella cerricolella* (Klimesch, 1948) Johansson, 1971: 253

***Stigmella bicuspidata*** van Nieukerken & Johansson, 2003: 341 WP

***Stigmella cocciferae*** van Nieukerken & Johansson, 2003: 329 WP

***Stigmella dorsiguttella*** (Johansson, 1971) Pröse, 1984: 107 WP

*Nepticula dorsiguttella* Johansson, 1971: 251

***Stigmella eberhardi*** (Johansson, 1971) Kasy, 1979: 4 WP

*Nepticula eberhardi* Johansson, 1971: 258

***Stigmella fasciata*** van Nieukerken & Johansson, 2003: 321 WP

***Stigmella ilicifoliella*** (Mendes, 1918) Gómez Bustillo, 1981: 18 WP

*Nepticula ilicifoliella* Mendes, 1918: 127

*Stigmella ilicivora nigra* Dufrane, 1955: 192 (syn: van Nieukerken & Johansson, 2003: 326)

***Stigmella karsholti*** van Nieukerken & Johansson, 2003: 343 WP

***Stigmella macrolepidella*** (Klimesch, 1978) van Nieukerken, 1986b: 13 WP

*Nepticula macrolepidella* Klimesch, 1978b: 257

***Stigmella roborella*** (Johansson, 1971) Emmet, 1976: 241 WP

*Nepticula roborella* Johansson, 1971: 258

*Nepticula ruficapitella* auct. partim before 1971

***Stigmella ruficapitella*** (Haworth, 1828) Beirne, 1945: 198 WP

*Tinea ruficapitella* Haworth, 1828: 586

*Tinea violacella* Haworth, 1828: 585 (syn: Stainton, 1849: 28)

*Microsetia ruficapitella* (Haworth, 1828) Stephens, 1834: 269

*Nepticula ruficapitella* (Haworth, 1828) Stainton, 1849: 28

*Microsetia violaceella* (Haworth, 1828) Stephens, 1834: 269

‡ *Nepticula lamprotornella* Heyden in Herrich-Schäffer, 1855b: 69 NN (syn: Herrich-Schäffer, 1855b: 69)

‡ Nepticula ruficapitella var. ruficastaneae Skala, 1949: 129 NNLM

***Stigmella samiatella*** (Zeller, 1839) Vári, 1950: 180 ^47^ WP

*Lyonetia samiatella* Zeller, 1839: 215

*Nepticula chaoniella* Herrich-Schäffer, 1863b: 170 NO **syn. n.** ^47^

*Nepticula quercella* Herrich-Schäffer, 1863a: 23 NN (syn: Segerer, 1997: 190) ^47^

*Nepticula samiatella* (Zeller, 1839) Zeller, 1848: 303

***Stigmella suberivora*** (Stainton, 1869) Beirne, 1945: 197 WP

*Nepticula suberivora* Stainton, 1869b: 228

*Nepticula aureocapitella* Millière, 1876 (syn: van Nieukerken & Johansson, 1987: 461)

*Nepticula aureocaputella* Millière, 1876: 374 IOS

*Nepticula ilicivora* Peyerimhoff, 1871: 413 (syn: Johansson, 1971: 246)

*Nepticula ilicella* Walsingham, 1891: 152 (syn: van Nieukerken, 2004: 111)

*Stigmella ilicivora* (Peyerimhoff, 1871) Gerasimov, 1952: 244

*Stigmella ilicella* (Walsingham, 1891) Le Marchand, 1946b: 284

***Stigmella svenssoni*** (Johansson, 1971) Emmet, 1976: 243 WP

*Nepticula svenssoni* Johansson, 1971: 249

***Stigmella szoecsiella*** (Borkowski, 1972) van Nieukerken, 1986a: 13 WP

*Nepticula szoecsiella* Borkowski, 1972b: 776

***Stigmella tristis*** (Wocke, 1862) Gerasimov, 1952: 265 WP

*Nepticula tristis* Wocke, 1862: 251

***Stigmella trojana*** Z. Laštuvka & A. Laštuvka, 1998: 313 WP

***Stigmella zangherii*** (Klimesch, 1951) Zangheri, 1969: 1014 WP

*Nepticula zangherii* Klimesch, 1951d: 61

***Stigmella acuta*** Diškus, Navickaitė & Remeikis in Stonis et al., 2013e: 202 EP

***Stigmella aladina*** Puplesis, 1984a: 115 EP

*Stigmella quercifaga* Kemperman & Wilkinson, 1985: 44 (syn: van Nieukerken & Liu, 2000: 161)

***Stigmella azuminoensis*** Hirano, 2010: 125 EP

***Stigmella caesurifasciella*** Kemperman & Wilkinson, 1985: 38 EP

*Stigmella egregilustrata* Kemperman & Wilkinson, 1985: 39 (syn: van Nieukerken & Liu, 2000: 174)

***Stigmella clisiotophora*** Kemperman & Wilkinson, 1985: 48 EP

***Stigmella crenatiella*** Hirano, 2010: 125 EP

***Stigmella dentatae*** Puplesis, 1984a: 114 EP

*Stigmella pulla* Kemperman & Wilkinson, 1985: 43 (syn: Puplesis, 1994: 162)

***Stigmella fervida*** Puplesis, 1984b: 593 EP

***Stigmella fumida*** Kemperman & Wilkinson, 1985: 42 EP

*Stigmella chrysopterella* Kemperman & Wilkinson, 1985: 48 (syn: van Nieukerken & Liu, 2000: 157)

*Stigmella kurii* Kemperman & Wilkinson, 1985: 51 (syn: van Nieukerken & Liu, 2000: 157)

***Stigmella hisaii*** Kuroko, 2004: 238 EP

***Stigmella hisakoae*** Hirano, 2010: 126 EP

***Stigmella kasyi*** van Nieukerken & Johansson, 2003: 331 EP

***Stigmella omelkoi*** Puplesis, 1984b: 593 EP

*Stigmella kumatai* Kemperman & Wilkinson, 1985: 50 (syn: Puplesis, 1994: 163)

### Unplaced Species groups

***Stigmella barbata* group** (Puplesis et al., 2002: 63)

***Stigmella plumosetaeella*** Newton & Wilkinson, 1982: 455 NEA

***Stigmella austroamericana*** Puplesis & Diškus in Puplesis et al., 2002: 25 NEO

***Stigmella barbata*** Puplesis & Robinson, 2000: 37 NEO

***Stigmella purpurimaculae* group** (Remeikis & Stonis in Stonis et al., 2014: 351)

***Stigmella cana*** Remeikis & Stonis in Stonis et al., 2014: 324 NEO

***Stigmella concreta*** Remeikis & Stonis in Stonis et al., 2014: 328 NEO

***Stigmella pseudoconcreta*** Remeikis & Stonis in Stonis et al., 2014: 329 NEO

***Stigmella purpurimaculae*** Remeikis & Stonis in Stonis et al., 2014: 323 NEO

***Stigmella quadrata*** Remeikis & Stonis in Stonis et al., 2014: 329 NEO

***Stigmella sceptra*** Remeikis & Stonis in Stonis et al., 2014: 327 NEO

***Stigmella truncata*** Remeikis & Stonis in Stonis et al., 2014: 326 NEO

### *Stigmella* unplaced and ungrouped species

***Stigmella arbatella*** (Chrétien, 1922) Rungs, 1979: 25 WP

*Nepticula arbatella* Chrétien, 1922: 373

***Stigmella georgiana*** Puplesis, 1994: 165 WP

***Stigmella grandistyla*** Puplesis, 1994: 113 WP

***Stigmella brutea*** Remeikis & Stonis in Stonis et al., 2014: 331 NEO

***Stigmella hylomaga*** (Meyrick, 1931a) Davis, 1984: 18 NEO

*Nepticula hylomaga* Meyrick, 1931a: 415

***Stigmella pruinosa*** Puplesis & Robinson, 2000: 38 NEO

***Stigmella pseudodigitata*** Remeikis & Stonis in Stonis et al., 2014: 332 NEO

***Stigmella semilactea*** Remeikis & Stonis in Stonis et al., 2014: 330 NEO

***Ozadelpha*** van Nieukerken in van Nieukerken et al., 2016: 26 (TS/OD: *Ozadelpha conostegiae* van Nieukerken & Nishida, 2016)

***Ozadelpha conostegiae*** van Nieukerken & Nishida in van Nieukerken et al., 2016: 28 NEO

***Ozadelpha guajavae*** (Puplesis & Diškus, 2002) van Nieukerken et al., 2016: 27 NEO

*Enteucha guajavae* Puplesis & Diškus in Puplesis et al., 2002: 22

***Ozadelpha ovata*** (Puplesis & Robinson, 2000) van Nieukerken et al., 2016: 27 NEO

*Stigmella ovata* Puplesis & Robinson, 2000: 39

***Roscidotoga*** Hoare, 2000a: 293 (TS/OD: *Roscidotoga callicomae* Hoare, 2000)

***Roscidotoga callicomae*** Hoare, 2000a: 295 (Fig. 27) AUS

***Roscidotoga eucryphiae*** Hoare, 2000a: 296 AUS

***Roscidotoga lamingtonia*** van Nieukerken, van den Berg & Hoare, 2011: 194 AUS

***Roscidotoga sapphiripes*** Hoare, 2000a: 297 AUS

***Casanovula*** Hoare in Hoare & van Nieukerken, 2013: 24 (TS/OD: *Pectinivalva brevipalpa* Hoare, 2013) **stat. n.**

***Casanovula brevipalpa*** (Hoare, 2013) **comb. n.** AUS

*Pectinivalva brevipalpa* Hoare in Hoare & van Nieukerken, 2013: 27

***Casanovula minotaurus*** (Hoare, 2013) **comb. n.** AUS

*Pectinivalva minotaurus* Hoare in Hoare & van Nieukerken, 2013: 29

***Menurella*** Hoare in Hoare & van Nieukerken, 2013: 35 (TS/OD: *Pectinivalva scotodes* Hoare, 2013) **stat. n.**

***Menurella acmenae*** (Hoare, 2013) **comb. n.** AUS

*Pectinivalva acmenae* Hoare in Hoare & van Nieukerken, 2013: 41

***Menurella anazona*** (Meyrick, 1906) **comb. n.** AUS

*Nepticula anazona* Meyrick, 1906b: 58

*Pectinivalva anazona* (Meyrick, 1906) Nielsen, 1996: 16

***Menurella funeralis*** (Meyrick, 1906) **comb. n.** AUS

*Nepticula funeralis* Meyrick, 1906b: 59

*Pectinivalva funeralis* (Meyrick, 1906) Nielsen, 1996: 16

***Menurella libera*** (Meyrick, 1906) **comb. n.** (Fig. 28) AUS

*Nepticula libera* Meyrick, 1906b: 61

*Pectinivalva libera* (Meyrick, 1906) Nielsen, 1996: 16

***Menurella planetis*** (Meyrick, 1906) **comb. n.** AUS

*Nepticula planetis* Meyrick, 1906b: 58

*Pectinivalva planetis* (Meyrick, 1906) Nielsen, 1996: 16

***Menurella primigena*** (Meyrick, 1906) **comb. n.** AUS

*Nepticula primigena* Meyrick, 1906b: 58

*Pectinivalva primigena* (Meyrick, 1906) Nielsen, 1996: 16

***Menurella quintiniae*** (Hoare & van Nieukerken, 2013) **comb. n.** AUS

*Pectinivalva quintiniae* Hoare & van Nieukerken, 2013: 47

***Menurella scotodes*** (Hoare, 2013) **comb. n.** AUS

*Pectinivalva scotodes* Hoare in Hoare & van Nieukerken, 2013: 37

***Menurella trepida*** (Meyrick, 1906) **comb. n.** AUS

*Nepticula trepida* Meyrick, 1906b: 61

*Pectinivalva trepida* (Meyrick, 1906) Nielsen, 1996: 16

***Menurella tribulatrix*** (van Nieukerken & Hoare, 2013) **comb. n.** AUS

*Pectinivalva tribulatrix* van Nieukerken & Hoare in Hoare & van Nieukerken, 2013: 48

***Menurella warburtonensis*** (Wilson, 1939) **comb. n.** AUS

*Nepticula warburtonensis* Wilson, 1939: 239

*Pectinivalva warburtonensis* (Wilson, 1939) Nielsen, 1996: 16

***Menurella xenadelpha*** (van Nieukerken & Hoare, 2013) **comb. n.** OR

*Pectinivalva xenadelpha* van Nieukerken & Hoare in Hoare & van Nieukerken, 2013: 44

***Pectinivalva*** Scoble, 1983: 12

***Pectinivalva caenodora*** (Meyrick, 1906) Nielsen, 1996: 16 (Fig. 29) AUS

*Nepticula caenodora* Meyrick, 1906b: 58

***Pectinivalva chalcitis*** (Meyrick, 1906) Nielsen, 1996: 16 AUS

*Nepticula chalcitis* Meyrick, 1906b: 60

***Pectinivalva commoni*** Scoble, 1983: 13 AUS

***Pectinivalva endocapna*** (Meyrick, 1906) Nielsen, 1996: 16 AUS

*Nepticula endocapna* Meyrick, 1906b: 60

***Pectinivalva gilva*** (Meyrick, 1906b) Nielsen, 1996: 16 AUS

*Nepticula gilva* Meyrick, 1906b: 59

***Pectinivalva melanotis*** (Meyrick, 1906) Nielsen, 1996: 16 AUS

*Nepticula melanotis* Meyrick, 1906b: 59

***Pectinivalva mystaconota*** Hoare in Hoare & van Nieukerken, 2013: 20 AUS

***Neotrifurcula*** van Nieukerken, 2016 in van Nieukerken et al., 2016: 36 (TS/OD: *Neotrifurcula gielisorum* van Nieukerken, 2016)

***Neotrifurcula gielisorum*** van Nieukerken, 2016 in van Nieukerken et al., 2016: 38 NEO

***Hesperolyra*** van Nieukerken, 2016 in van Nieukerken et al., 2016: 44 (TS/OD: *Fomoria diskusi* Puplesis & Robinson, 2000)

*Fomoria molybditis* group Puplesis et al., 2002

***Hesperolyra diskusi*** (Puplesis & Robinson, 2000) van Nieukerken et al., 2016: 44 NEO

*Fomoria diskusi* Puplesis & Robinson, 2000: 43

***Hesperolyra molybditis*** (Zeller, 1877) van Nieukerken et al., 2016: 46 NEO

*Nepticula molybditis* Zeller, 1877: 453

*Stigmella molybditis* (Zeller, 1877) Davis, 1984: 18

*Fomoria molybditis* (Zeller, 1877) Puplesis & Robinson, 2000: 43

***Hesperolyra repanda*** (Puplesis & Diškus, 2002) van Nieukerken et al., 2016: 46 NEO

*Fomoria repanda* Puplesis & Diškus in Puplesis et al., 2002: 26

***Hesperolyra saopaulensis*** van Nieukerken, 2016 in van Nieukerken et al., 2016: 52 NEO

***Bohemannia*** Stainton, 1859a: 439 (TS/M: *Nepticula quadrimaculella* Boheman, 1853)

*Scoliaula* Meyrick, 1895: 727; URN for *Bohemannia* Stainton, 1859 (TS/OD,M: *Nepticula quadrimaculella* Boheman, 1853)

***Bohemannia aschaueri*** Fischer, 2013: 88 WP†

***Bohemannia butzmanni*** Fischer, 2013: 86 WP†

***Bohemannia auriciliella*** (Joannis, 1909) van Nieukerken, 1986a: 16 (Fig. 31) WP

*Nepticula auriciliella* Joannis, 1909: 822

*Ectoedemia bradfordi* Emmet, 1974: 269 (syn: van Nieukerken, 1986a: 16)

*Stigmella auriciliella* (Joannis, 1909) Lhomme, [1963]: 1192

***Bohemannia pulverosella*** (Stainton, 1849) van Nieukerken, 1982: 105 ^48^ WP,EP,[NEA]

*Trifurcula pulverosella* Stainton, 1849: 30

*Bohemannia piotra* Puplesis, 1984b: 586 **syn. n.**^48^

*Nepticula pulverosella* (Stainton, 1849) Meyrick, 1895: 726

*Stigmella pulverosella* (Stainton, 1849) Fletcher & Clutterbuck, 1945: 61

*Dechtiria pulverosella* (Stainton, 1849) Beirne, 1945: 206

*Ectoedemia pulverosella* (Stainton, 1849) Bradley et al., 1972: 3

*Scoliaula pulverosella* (Stainton, 1849) Borkowski, 1975: 489

‡ *Nepticula cineretella* Frey in Herrich-Schäffer, 1855b: 70 NN (syn: Herrich-Schäffer, 1855b: 70)

***Bohemannia quadrimaculella*** (Boheman, 1853) Stainton, 1859a: 439 ^49^ WP

*Nepticula quadrimaculella* Boheman, 1853: 167

*Bucculatrix antispilella* Meess, 1907: 129 (syn: Disqué, 1912: 75) ^49^

*Scoliaula quadrimaculella* (Boheman, 1853) Meyrick, 1895: 727

*Trifurcula quadrimaculella* (Boheman, 1853) Johansson, 1971: 246

***Bohemannia ussuriella*** Puplesis, 1984b: 588 EP

***Bohemannia manschurella*** Puplesis, 1984b: 587 ^50^ EP

*Bohemannia nipponicella* Hirano, 2010: 129 **syn. n.** ^50^

***Bohemannia nubila*** Puplesis, 1985c: 69 EP

***Bohemannia suiphunella*** Puplesis, 1984b: 588 EP

***Areticulata*** Scoble, 1983: 11 (key), 40 (TS/OD,M: *Areticulata leucosideae* Scoble, 1983)

***Areticulata leucosideae*** Scoble, 1983: 40 AFR

***Glaucolepis*** Braun, 1917: 161 (key), 201 (TS/OD,M: *Nepticula saccharella* Braun, 1912)

*Fedalmia* Beirne, 1945: 207 (TS/OD,M: *Nepticula headleyella* Stainton, 1854) (syn: Puplesis, 1985a: 11)

*Sinopticula* Yang, 1989: 79 [81] (TS/OD,M: *Sinopticula sinica* Yang, 1989: 80) (syn: van Nieukerken & Puplesis, 1991: 202)

***Glaucolepis raikhonae* group** (van Nieukerken & Puplesis, 1991: 202)

***Glaucolepis melanoptera*** (van Nieukerken & Puplesis, 1991) Puplesis, 1994: 219 WP

*Trifurcula melanoptera* van Nieukerken & Puplesis, 1991

***Glaucolepis oishiella*** (Matsumura, 1931) **comb. n.** ^51^ EP

*Trifurcula oishiella* Matsumura, 1931: 1114

*Sinopticula sinica* Yang, 1989: 80 **syn. n.** ^51^

*Trifurcula sinica* (Yang, 1989) van Nieukerken & Puplesis, 1991: 205

*Glaucolepis sinica* (Yang, 1989) Diškus & Puplesis, 2003: 404

***Glaucolepis raikhonae*** Puplesis, 1985c: 71 EP

*Trifurcula raikhonae* (Puplesis, 1985) van Nieukerken, 1986a: 68

***Glaucolepis saccharella* group** (new)

***Glaucolepis saccharella*** (Braun, 1912) Braun, 1917: 201 NEA

*Nepticula saccharella* Braun, 1912: 97

*Trifurcula saccharella* (Braun, 1912) van Nieukerken, 1986a: 68

***Glaucolepis headleyella* group** (Puplesis, 1994: 219)

***Glaucolepis albiflorella*** (Klimesch, 1978) Diškus & Puplesis, 2003: 406 WP

*Trifurcula albiflorella* Klimesch, 1978b: 274

***Glaucolepis alypella*** (Klimesch, 1975) Diškus & Puplesis, 2003: 406 WP

*Trifurcula alypella* Klimesch, 1975c: 12

***Glaucolepis andalusica*** (Z. Laštuvka & A. Laštuvka, 2007) **comb. n.** WP

*Trifurcula andalusica* Z. Laštuvka & A. Laštuvka, 2007: 102

***Glaucolepis bleonella*** (Chrétien, 1904) Puplesis, 1994: 219 WP

*Nepticula bleonella* Chrétien, 1904: 164

*Stigmella bleonella* (Chrétien, 1904) Gerasimov, 1952: 231

*Ectoedemia bleonella* (Chrétien, 1904) Klimesch, 1975a: 861

*Trifurcula bleonella* (Chrétien, 1904) Leraut, 1980: 49

***Glaucolepis bupleurella*** (Chrétien, 1907) Diškus & Puplesis, 2003: 405 WP

*Nepticula bupleurella* Chrétien, 1907: 91

*Stigmella bupleurella* (Chrétien, 1907) Gerasimov, 1952: 232

*Ectoedemia bupleurella* (Chrétien, 1907) Klimesch, 1975a: 863

*Trifurcula bupleurella* (Chrétien, 1907) Leraut, 1980: 49

***Glaucolepis chretieni*** (Z. Laštůvka, A. Laštůvka & van Nieukerken, 2013) **comb. n.** WP

*Trifurcula chretieni* Z. Laštůvka, A. Laštůvka & van Nieukerken, 2013: 198

***Glaucolepis corleyi*** (Z. Laštuvka & A. Laštuvka, 2007) **comb. n.** WP

*Trifurcula corleyi* Z. Laštuvka & A. Laštuvka, 2007: 102

***Glaucolepis globulariae*** (Klimesch, 1975) Diškus & Puplesis, 2003: 406 WP

*Trifurcula globulariae* Klimesch, 1975c: 7

***Glaucolepis hamirella*** (Chrétien, 1915) Diškus & Puplesis, 2003: 406 ^52^ WP

*Nepticula hamirella* Chrétien, 1915: 364

*Ectoedemia hamirella* (Chrétien, 1915) Klimesch, 1975a: 863

*Trifurcula hamirella* (Chrétien, 1915) van Nieukerken, 1986a: 15

***Glaucolepis saturejae*** (Parenti, 1963) Diškus & Puplesis, 2003: 406 ^52^ WP

*Stigmella saturejae* Parenti, 1963: 101

*Fedalmia saturejae* (Parenti, 1963) Klimesch, 1976: 45

*Trifurcula saturejae* (Parenti, 1963) van Nieukerken, 1986a: 15

***Glaucolepis headleyella*** (Stainton, 1854) Puplesis, 1994: 219 WP

*Nepticula headleyella* Stainton, 1854: 298

*Nepticula argyrostigma* Frey, 1856: 379 (syn: Frey, 1880: 425)

*Nepticula dubiella* Hauder, 1912: 273 (syn: Klimesch, 1948b: 76)

*Trifurcula rodella* Svensson, 1982: 299 (syn: van Nieukerken, 1986a: 15)

*Fedalmia headleyella* (Stainton, 1854) Beirne, 1945: 207

*Stigmella headleyella* (Stainton, 1854) Klimesch, 1948b: 76

*Trifurcula headleyella* (Stainton, 1854) Johansson, 1971: 245

*Stigmella dubiella* (Hauder, 1912) Klimesch, 1948b: 76

***Glaucolepis helladica*** (Z. Laštuvka & A. Laštuvka, 2007) **comb. n.** WP

*Trifurcula helladica* Z. Laštuvka & A. Laštuvka, 2007: 101

***Glaucolepis istriae*** (A. Laštuvka & Z. Laštuvka, 2000) Diškus & Puplesis, 2003: 406 WP

*Trifurcula istriae* A. Laštuvka & Z. Laštuvka, 2000a: 290

***Glaucolepis kalavritana*** (Z. Laštuvka & A. Laštuvka, 1998) Diškus & Puplesis, 2003: 407 WP

*Trifurcula kalavritana* (Z. Laštuvka & A. Laštuvka, 1998): 314

***Glaucolepis lavandulae*** (Z. Laštuvka & A. Laštuvka, 2007) **comb. n.** WP

*Trifurcula lavandulae* Z. Laštuvka & A. Laštuvka, 2007: 104

***Glaucolepis liskai*** (A. Laštuvka & Z. Laštuvka, 2000) Diškus & Puplesis, 2003: 407 WP

*Trifurcula liskai* A. Laštuvka & Z. Laštuvka, 2000a: 291

***Glaucolepis lituanica*** (Ivinskis & van Nieukerken, 2012) **comb. n.** (Fig. 30) WP

*Trifurcula lituanica* Ivinskis & van Nieukerken, 2012: 43

***Glaucolepis magna*** (A. Laštuvka & Z. Laštuvka, 1997) Diškus & Puplesis, 2003: 407 ^53^ WP

*Trifurcula magna* (A. Laštuvka & Z. Laštuvka, 1997): 132

*Trifurcula collinella* Nel, 2012: 24 **syn. n.** ^53^

***Glaucolepis megaphallus*** (van Nieukerken, Z. Laštůvka & A. Laštůvka, 2013) **comb. n.** WP

*Trifurcula megaphallus* van Nieukerken, Z. Laštůvka & A. Laštůvka in Z. Laštůvka et al., 2013: 195

***Glaucolepis micromeriae*** (Walsingham, 1908) Diškus & Puplesis, 2003: 405 WP

*Stigmella micromeriae* Walsingham, 1908a: 1010^1^

*Nepticula micromeriae* (Walsingham, 1908) Rebel, 1910: 374

*Trifurcula micromeriae* (Walsingham, 1908) Klimesch, 1977: 196

***Glaucolepis montana*** (Z. Laštuvka, A. Laštuvka & van Nieukerken) **comb. n.** WP

*Trifurcula montana* Z. Laštuvka, A. Laštuvka & van Nieukerken in Z. & A. Laštuvka, 2007: 103

***Glaucolepis pederi*** (Z. Laštuvka & A. Laštuvka, 2007) **comb. n.** WP

*Trifurcula pederi* Z. Laštuvka & A. Laštuvka, 2007: 102

***Glaucolepis rosmarinella*** (Chrétien, 1914) Diškus & Puplesis, 2003: 405 WP

*Nepticula rosmarinella* Chrétien, 1914: 270

*Stigmella rosmarinella* (Chrétien, 1914) Gerasimov, 1952: 256

*Trifurcula rosmarinella* (Chrétien, 1914) Klimesch, 1975b: 23

***Glaucolepis salicinae*** (Klimesch, 1975) Diškus & Puplesis, 2003: 406 WP

*Trifurcula salicinae* Klimesch, 1975c: 10

***Glaucolepis salvifoliae*** (Z. Laštuvka & A. Laštuvka, 2007) **comb. n.** WP

*Trifurcula salvifoliae* Z. Laštuvka & A. Laštuvka, 2007: 103

***Glaucolepis sanctaecrucis*** (Walsingham, 1908) Diškus & Puplesis, 2003: 405 WP

*Stigmella sanctaecrucis* Walsingham, 1908a: 1010^1^

*Nepticula sanctaecrucis* (Walsingham, 1908) Rebel, 1910: 374

*Fedalmia sanctaecrucis* (Walsingham, 1908) Klimesch, 1976: 44

*Trifurcula sanctaecrucis* (Walsingham, 1908) Klimesch, 1977: 196

***Glaucolepis sanctibenedicti*** (Klimesch, 1979) Diškus & Puplesis, 2003: 406 WP

*Trifurcula sanctibenedicti* Klimesch, 1979: 24

***Glaucolepis siciliae*** (Z. Laštůvka, A. Laštůvka & van Nieukerken, 2013) **comb. n.** WP

*Trifurcula siciliae* Z. Laštůvka, A. Laštůvka & van Nieukerken, 2013: 201

***Glaucolepis stoechadella*** (Klimesch, 1975) Diškus & Puplesis, 2003: 406 WP

*Trifurcula stoechadella* Klimesch, 1975c: 23

***Glaucolepis teucriella*** (Chrétien, 1914) Diškus & Puplesis, 2003: 405 WP

*Nepticula teucriella* Chrétien, 1914: 270

*Stigmella teucriella* (Chrétien, 1914) Gerasimov, 1952: 263

*Trifurcula teucriella* (Chrétien, 1914) Leraut, 1980: 49

***Glaucolepis thymi*** (Szőcs, 1965) Diškus & Puplesis, 2003: 406 WP

*Nepticula thymi* Szőcs, 1965: 89

*Fedalmia thymi* Borkowski, 1970a: 74; JSH of *Nepticula thymi* Szőcs, 1965

*Trifurcula thymi* (Szőcs, 1965) van Nieukerken, 1986a: 15

***Glaucolepis trilobella*** (Klimesch, 1978) Diškus & Puplesis, 2003: 406 WP

*Trifurcula trilobella* Klimesch, 1978b: 271

***Glaucolepis zollikofferiela*** (Chrétien, 1914) Diškus & Puplesis, 2003: 405 WP

*Nepticula zollikofferiela* Chrétien, 1914: 271

*Stigmella zollikofferiela* (Chrétien, 1914) Gerasimov, 1952: 270

*Ectoedemia zollikofferiela* (Chrétien, 1914) Klimesch, 1975a: 862

*Trifurcula zollikofferiela* (Chrétien, 1914) van Nieukerken, 1986a: 15

***Glaucolepis rusticula*** (Meyrick, 1916) Diškus & Puplesis, 2003: 406 OR

*Nepticula rusticula* Meyrick, 1916b: 7

*Trifurcula rusticula* (Meyrick, 1916) online comb.

**Unassigned to group** ^54^

***Glaucolepis aerifica*** (Meyrick, 1915) Puplesis & Robinson, 2000: 56 NEO

*Nepticula aerifica* Meyrick, 1915a: 255

*Stigmella aerifica* (Meyrick, 1915) Davis, 1984: 18

*Trifurcula aerifica* (Meyrick, 1915) online comb.

***Glaucolepis argentosa*** Puplesis & Robinson, 2000: 57 ^54^ NEO

*Trifurcula argentosa* (Puplesis & Robinson, 2000) online comb.

***Trifurcula*** Zeller, 1848: 249 (key), 330 (TS/SD (Tutt, 1899: 355): *Trifurcula pallidella* Zeller, 1848)

*Trifurcella* Chambers, 1878: 165 ISS

*Levarchama* Beirne, 1945: 206 (TS/OD: *Nepticula cryptella* Stainton, 1856) (syn: Johansson, 1971: 246)

***Trifurcula cryptella* group** (new)

***Trifurcula anthyllidella*** Klimesch, 1975c: 14 WP

***Trifurcula cryptella*** (Stainton, 1856) Johansson, 1971: 246 WP

*Nepticula cryptella* Stainton, 1856: 41

*Nepticula trifolii* Sorhagen, 1885: 280 (syn: Hering, 1957: 1067)

*Stigmella cryptella* (Stainton, 1856) Fletcher & Clutterbuck, 1945: 61

*Levarchama cryptella* (Stainton, 1856) Beirne, 1945: 207

***Trifurcula eurema*** (Tutt, 1899) Johansson, 1971: 246 WP

*Nepticula eurema* Tutt, 1899: 332

*Nepticula heurema* Meess, 1910: 481 UE

*Nepticula dorycniella* Suire, 1928: 128 (syn: van Nieukerken, 1986a: 15)

*Nepticula gozmanyi* Szőcs, 1959: 417 (syn: van Nieukerken, 1986a: 15)

*Levarchama eurema* (Tutt, 1899) Beirne, 1945: 207

*Stigmella eurema* (Tutt, 1899) Klimesch, 1951b: 66

*Stigmella heurema* (Meess, 1910) Gerasimov, 1952: 243

*Stigmella dorycniella* (Suire, 1928) Klimesch, 1951b: 66

***Trifurcula manygoza*** van Nieukerken, A. Laštuvka & Z. Laštuvka in van Nieukerken, 2007b: 125 WP

***Trifurcula ortneri*** (Klimesch, 1951) van Nieukerken, 1986a: 15 WP

*Nepticula ortneri* Klimesch, 1951b: 66

*Stigmella ortneri* (Klimesch, 1951) Klimesch, 1961: 763

***Trifurcula peloponnesica*** van Nieukerken, 2007b: 118 WP

***Trifurcula ridiculosa*** (Walsingham, 1908) Klimesch, 1975b: 15 WP

*Stigmella ridiculosa* Walsingham, 1908a: 1011^1^

*Nepticula ridiculosa* (Walsingham, 1908) Rebel, 1910: 364

***Trifurcula subnitidella* group** (van Nieukerken, 1990b: 208)

***Trifurcula coronillae*** van Nieukerken, 1990b: 217 WP

***Trifurcula iberica*** van Nieukerken, 1990b: 228 (Fig. 32) WP

***Trifurcula josefklimeschi*** van Nieukerken, 1990b: 225 WP

***Trifurcula luteola*** van Nieukerken, 1990b: 215 WP

***Trifurcula puplesisi*** van Nieukerken, 1990b: 215 WP,EP

***Trifurcula silviae*** van Nieukerken, 1990b: 230 WP

***Trifurcula subnitidella*** (Duponchel, 1843) van Nieukerken & Johansson, 1987: 462 WP

*Elachista subnitidella* Duponchel, 1843: 326

*Trifurcula griseella* Wolff, 1957: 21 (syn: van Nieukerken & Johansson, 1987: 462)

*Lyonetia subnitidella* (Duponchel, 1843) Duponchel, 1844: 378

*Nepticula subnitidella* (Duponchel, 1843) Zeller, 1848: 305

***Trifurcula victoris*** van Nieukerken, 1990b: 219 WP

***Trifurcula pallidella* group** (van Nieukerken, 1990b: 208)

***Trifurcula aurella*** Rebel, 1933: 82 WP

***Trifurcula austriaca*** van Nieukerken, 1990b: 213 WP

***Trifurcula baldensis*** A. Laštuvka & Z. Laštuvka, 2005a: 8 WP

***Trifurcula beirnei*** Puplesis, 1984a: 124 WP

***Trifurcula bicolorella*** (Chrétien, 1915) **comb. n.** ^55^ WP

*Bucculatrix bicolorella* Chrétien, 1915: 364

***Trifurcula calycotomella*** A. Laštuvka & Z. Laštuvka, 1997: 148 WP

***Trifurcula chamaecytisi*** A. Laštuvka & Z. Laštuvka, 1994: 207 WP

***Trifurcula corothamni*** A. Laštuvka & Z. Laštuvka, 1994: 202 WP

***Trifurcula cytisanthi*** A. Laštuvka & Z. Laštuvka, 2005a: 8 WP

***Trifurcula etnensis*** A. Laštuvka & Z. Laštuvka, 2005a: 7 WP

***Trifurcula graeca*** Z. Laštuvka & A. Laštuvka, 1998: 315 WP

***Trifurcula immundella*** (Zeller, 1839) Zeller, 1848: 332 WP

*Lyonetia immundella* Zeller, 1839: 215

***Trifurcula macedonica*** Z. Laštuvka & A. Laštuvka, 1998: 315 WP

***Trifurcula moravica*** A. Laštuvka & Z. Laštuvka, 1994: 205 WP

***Trifurcula orientella*** Klimesch, 1953a: 168 WP

***Trifurcula pallidella*** (Duponchel, 1843) Joannis, 1915: 129 WP

*Oecophora pallidella* Duponchel, 1843: 339

*Trifurcula pallidella* Zeller, 1848: 332; JSH of *Trifurcula pallidella* (Duponchel, 1843)

*Lithocolletis pallidella* (Duponchel, 1843) Bruand, [1851]: 86

*Trifurcula incognitella* Toll, 1936: 409 (syn: van Nieukerken, 1986a: 15)

‡ [*no genus] pallidulella* Herrich-Schäffer, 1853: pl 108 NN

***Trifurcula serotinella*** Herrich-Schäffer, 1855a: 359 WP

*Trifurcula confertella* Fuchs, 1895: 47 (syn: van Nieukerken, 1986a: 15)

***Trifurcula squamatella*** Stainton, 1849: 30 WP

*Trifurcula maxima* Klimesch, 1953a: 167 (syn: van Nieukerken, 1987b: 180)

***Trifurcula trasaghica*** A. Laštuvka & Z. Laštuvka, 2005a: 9 WP

***Trifurcula barbertonensis* group** (new)

***Trifurcula barbertonensis*** Scoble, 1980a: 142 AFR

***Trifurcula pullus*** Scoble, 1980a: 140 AFR

***Fomoria*** Beirne, 1945: 208 (TS/OD: *Nepticula weaveri* Stainton, 1855)

***Fomoria vannifera* group** (Hoare, 2000b: 300)

*Fomoria asiatica* group (Puplesis, 1994: 208)

***Fomoria asiatica*** Puplesis, 1988: 27 EP

*Ectoedemia asiatica* (Puplesis, 1988) Hoare, 2000a: 301

***Fomoria glycystrota*** (Meyrick, 1928) Diškus & Puplesis, 2003: 385 OR

*Nepticula glycystrota* Meyrick, 1928b: 462

*Ectoedemia glycystrota* (Meyrick, 1928b) Hoare, 2000a: 302

***Fomoria fuscata*** (Janse, 1948) Diškus & Puplesis, 2003: 385 AFR

*Nepticula fuscata* Janse, 1948: 165

*Ectoedemia fuscata* (Janse, 1948) Scoble, 1983: 36

***Fomoria hobohmi*** (Janse, 1948) Diškus & Puplesis, 2003: 385 AFR

*Nepticula hobohmi* Janse, 1948: 167

*Ectoedemia hobohmi* (Janse, 1948) Scoble, 1983: 38

***Fomoria kharuxabi*** (Mey, 2004) **comb. n.** AFR

*Ectoedemia kharuxabi* Mey, 2004: 32

***Fomoria uisebi*** (Mey, 2004) **comb. n.** AFR

*Ectoedemia uisebi* Mey, 2004: 32

***Fomoria vannifera*** (Meyrick, 1914) Diškus & Puplesis, 2003: 385 AFR

*Nepticula vannifera* Meyrick, 1914: 203

*Ectoedemia vannifera* (Meyrick, 1914) Scoble, 1983: 37

***Fomoria hadronycha*** (Hoare, 2000) Diškus & Puplesis, 2003: 385 AUS

*Ectoedemia hadronycha* Hoare, 2000b: 307

***Fomoria pelops*** (Hoare, 2000) Diškus & Puplesis, 2003: 385 AUS

*Ectoedemia pelops* Hoare, 2000b: 304

***Fomoria squamibunda*** (Hoare, 2000) Diškus & Puplesis, 2003: 385 AUS

*Ectoedemia squamibunda* Hoare, 2000b: 304

***Fomoria groschkei* group** (Hoare, 2000b: 313)

***Fomoria aegaeica*** (Z. Laštuvka, A. Laštuvka & Johansson, 1998) Diškus & Puplesis, 2003: 391 WP

*Ectoedemia aegaeica* Z. Laštuvka, A. Laštuvka & Johansson in Z. & A. Laštuvka, 1998: 316

***Fomoria groschkei*** (Skala, 1943) Diškus & Puplesis, 2003: 388 WP

*Nepticula groschkei* Skala, 1943: 86

*Stigmella groschkei* (Skala, 1943) Klimesch, 1948b: 77

*Ectoedemia groschkei* (Skala, 1943) van Nieukerken, 1986a: 17

***Fomoria thermae*** (Scoble, 1983) Diškus & Puplesis, 2003: 390 AFR

*Ectoedemia thermae* Scoble, 1983: 36

***Fomoria weaveri* group** (Puplesis, 1994: 205)

***Fomoria degeeri*** (van Nieukerken, 2008) **comb. n.** WP

*Ectoedemia degeeri* van Nieukerken, 2008: 124

***Fomoria deschkai*** (Klimesch, 1978) Diškus & Puplesis, 2003: 384 WP

*Trifurcula deschkai* Klimesch, 1978b: 274

*Ectoedemia deschkai* (Klimesch, 1978b) van Nieukerken, 1986a: 17

***Fomoria empetrifolii*** (A. Laštuvka & Z. Laštuvka, 2000) Diškus & Puplesis, 2003: 385 WP

*Ectoedemia empetrifolii* A. Laštuvka & Z. Laštuvka, 2000b: 22

***Fomoria eriki*** (A. Laštuvka & Z. Laštuvka, 2000) Diškus & Puplesis, 2003: 385 WP

*Ectoedemia eriki* A. Laštuvka & Z. Laštuvka, 2000b: 21

***Fomoria luisae*** Klimesch, 1978a: 89 WP

*Ectoedemia luisae* (Klimesch, 1978) van Nieukerken, 1986a: 17

***Fomoria septembrella*** (Stainton, 1849) Beirne, 1945: 209 WP

*Nepticula septembrella* Stainton, 1849: 29

*Stigmella septembrella* (Stainton, 1849) Fletcher & Clutterbuck, 1945: 61

*Trifurcula septembrella* (Stainton, 1849) Johansson, 1971: 246

*Ectoedemia septembrella* (Stainton, 1849) Scoble, 1983: 32

***Fomoria variicapitella*** (Chrétien, 1908) Diškus & Puplesis, 2003: 384 WP

*Nepticula variicapitella* Chrétien, 1908: 363

*Stigmella variicapitella* (Chrétien, 1908) Gerasimov, 1952: 263

*Trifurcula variicapitella* (Chrétien, 1908) Klimesch, 1977: 197

*Ectoedemia variicapitella* (Chrétien, 1908) van Nieukerken, 1986a: 17

***Fomoria weaveri*** (Stainton, 1855) Beirne, 1945: 209 (Fig. 33) WP,EP,NEA

*Nepticula weaveri* Stainton, 1855: 49

*Nepticula weaweri* Herrich-Schäffer, 1855a: 346 ISS

*Nepticula weaverella* Doubleday, 1859: 36 UE

*Stigmella weaveri* (Stainton, 1855) Gerasimov, 1952: 269

*Trifurcula weaveri* (Stainton, 1855) Johansson, 1971: 246

*Ectoedemia weaveri* (Stainton, 1855) Scoble, 1983: 32

‡ Fomoria weaveri f. fuliginella Vári, 1947: 523

***Fomoria festivitatis*** (van Nieukerken, 2008) **comb. n.** OR, EP

*Ectoedemia festivitatis* van Nieukerken, 2008: 117

***Fomoria hypericifolia*** Kuroko, 1982: 49 EP

*Ectoedemia hypericifolia* (Kuroko, 1982) van Nieukerken, 1986a: 84

***Fomoria permira*** Puplesis, 1984b: 592 EP

*Ectoedemia permira* (Puplesis, 1984) van Nieukerken, 1986a: 84

***Fomoria hypericella*** (Braun, 1925) Wilkinson, 1979: 84 NEA

*Nepticula hypericella* Braun, 1925a: 17

*Ectoedemia hypericella* (Braun, 1925a) van Nieukerken, 2008: 116

***Fomoria pteliaeella*** (Chambers, 1880) Wilkinson, 1979: 84 NEA

*Nepticula pteliaeella* Chambers, 1880c: 137

*Ectoedemia pteliaeella* (Chambers, 1880) van Nieukerken, 2008: 117

***Fomoria ruwenzoriensis*** (Bradley, 1965) **comb. n.** AFR

*Stigmella ruwenzoriensis* Bradley, 1965: 120

*Acalyptris ruwenzoriensis* (Bradley, 1965) Diškus & Puplesis, 2003: 394

*Ectoedemia ruwenzoriensis* (Bradley, 1965) van Nieukerken, 2008: 117

***Fomoria lacrimulae* group** (Diškus & Puplesis, 2003: 386)

***Fomoria lacrimulae*** Puplesis & Diškus, 1996c: 185 WP

*Ectoedemia lacrimulae* (Puplesis & Diškus, 1996) online comb.

***Fomoria knysnaensis*** (Scoble, 1983) Diškus & Puplesis, 2003: 386 AFR

*Ectoedemia knysnaensis* Scoble, 1983: 33

**African unplaced *Fomoria***

***Fomoria alexandria*** (Scoble, 1983) Diškus & Puplesis, 2003: 390 AFR

*Ectoedemia alexandria* Scoble, 1983: 35

***Fomoria gambiana*** (Gustafsson, 1972) Diškus & Puplesis, 2003: 389 AFR

*Nepticula gambiana* Gustafsson, 1972: 156

*Ectoedemia gambiana* (Gustafsson, 1972) online comb.

***Fomoria incisaevora*** (Scoble, 1983) Diškus & Puplesis, 2003: 390 AFR

*Ectoedemia incisaevora* Scoble, 1983: 35

***Fomoria indicaevora*** (Scoble, 1983) Diškus & Puplesis, 2003: 390 AFR

*Ectoedemia indicaevora* Scoble, 1983: 33

***Fomoria leptodictyae*** (Scoble, 1983) Diškus & Puplesis, 2003: 390 AFR

*Ectoedemia leptodictyae* Scoble, 1983: 35

***Fomoria lucidae*** (Scoble, 1983) Diškus & Puplesis, 2003: 390 AFR

*Ectoedemia lucidae* Scoble, 1983: 34

***Fomoria malelanensis*** (Scoble, 1983) Diškus & Puplesis, 2003: 390 AFR

*Ectoedemia malelanensis* Scoble, 1983: 36

***Fomoria myrtinaecola*** (Scoble, 1983) Diškus & Puplesis, 2003: 390 AFR

*Ectoedemia myrtinaecola* Scoble, 1983: 34

***Fomoria oleivora*** (Vári, 1955) Diškus & Puplesis, 2003: 388 AFR

*Stigmella oleivora* Vári, 1955: 336

*Ectoedemia oleivora* (Vári, 1955) Scoble, 1983: 32

***Fomoria pappeivora*** (Vári, 1963) Diškus & Puplesis, 2003: 388 AFR

*Stigmella pappeivora* Vári, 1963: 68

*Ectoedemia pappeivora* (Vári, 1963) Scoble, 1983: 32

***Fomoria portensis*** (Scoble, 1983) Diškus & Puplesis, 2003: 390 AFR

*Ectoedemia portensis* Scoble, 1983: 36

***Fomoria primaria*** (Meyrick, 1913) Diškus & Puplesis, 2003: 388 AFR

*Nepticula primaria* Meyrick, 1913: 326

*Ectoedemia primaria* (Meyrick, 1913) Scoble, 1983: 38

***Fomoria scobleella*** (Minet, 2004) **comb. n.** ^56^ AFR

*Ectoedemia scoblei* Minet, 1990: 220; JPH of *Ectoedemia scoblei* Puplesis, 1984a

*Ectoedemia scobleella* Minet, 2004: 366; RN for *Ectoedemia scoblei* Minet, 1990

*Fomoria scoblei* (Minet, 1990) Diškus & Puplesis, 2003: 391

***Fomoria tecomariae*** (Vári, 1955) Diškus & Puplesis, 2003: 388 AFR

*Stigmella tecomariae* Vári, 1955: 333

*Ectoedemia tecomariae* (Vári, 1955) Scoble, 1983: 34

**other unplaced *Fomoria***

***Fomoria viridissimella*** (Caradja, 1920) Diškus & Puplesis, 2003: 385 WP

*Nepticula viridissimella* Caradja, 1920: 162

*Nepticula nowakowskii* Toll, 1957: 199 (syn: van Nieukerken, 1987a: 142)

*Ectoedemia viridissimella* (Caradja, 1920) van Nieukerken, 1987a: 142

*Ectoedemia nowakowskii* (Toll, 1957) van Nieukerken, 1986a: 17

***Fomoria argyraspis*** (Puplesis & Diškus, 1995) **comb. n.** ^57^ EP

*Acalyptris argyraspis* Puplesis & Diškus, 1995: 51

***Fomoria flavimacula*** Puplesis & Diškus, 1996c: 183 EP

*Ectoedemia flavimacula* (Puplesis & Diškus, 1996c) online comb.

***Fomoria sporadopa*** (Meyrick, 1911) **comb. n.** ^58^ OR

*Nepticula sporadopa* Meyrick, 1911c: 108

*Acalyptris sporadopa* (Meyrick, 1911) Diškus & Puplesis, 2003: 393

***Fomoria tabulosa*** Puplesis & Diškus in Puplesis et al., 2002: 27 NEO

***Muhabbetana*** Koçak & Kemal, 2007: 5 **stat. n.**; RN for *Laqueus* Scoble, 1983

*Laqueus* Scoble, 1983: 12 (key), 20; JH of *Laqueus* Dall, 1870 (Brachiopoda) (TS/OD: *Nepticula grandinosa* Meyrick, 1911) [as subgenus of *Ectoedemia*]

***Muhabbetana grandinosa* group** (new)

***Muhabbetana furcella*** (Scoble, 1983) **comb. n.** AFR

*Ectoedemia furcella* Scoble, 1983: 24

*Fomoria furcella* (Scoble, 1983) Diškus & Puplesis, 2003: 387

***Muhabbetana grandinosa*** (Meyrick, 1911) **comb. n.** AFR

*Nepticula grandinosa* Meyrick, 1911a: 236

*Ectoedemia grandinosa* (Meyrick, 1911) Scoble, 1983: 21

*Fomoria grandinosa* (Meyrick, 1911) Diškus & Puplesis, 2003: 386

***Muhabbetana guerkiae*** (Scoble, 1983) **comb. n.** AFR

*Ectoedemia guerkiae* Scoble, 1983: 22

*Fomoria guerkiae* (Scoble, 1983) Diškus & Puplesis, 2003: 387

***Muhabbetana jupiteri*** (Scoble, 1983) **comb. n.** AFR

*Ectoedemia jupiteri* Scoble, 1983: 25

*Fomoria jupiteri* (Scoble, 1983) Diškus & Puplesis, 2003: 387

***Muhabbetana macrochaeta*** (Meyrick, 1921) **comb. n.** AFR

*Nepticula macrochaeta* Meyrick, 1921b: 140

*Ectoedemia macrochaeta* (Meyrick, 1921) Scoble, 1983: 23

*Fomoria macrochaeta* (Meyrick, 1921) Diškus & Puplesis, 2003: 387

***Muhabbetana maritima*** (Scoble, 1983) **comb. n.** AFR

*Ectoedemia maritima* Scoble, 1983: 24

*Fomoria maritima* (Scoble, 1983) Diškus & Puplesis, 2003: 387

***Muhabbetana scabridae*** (Scoble, 1983) **comb. n.** AFR

*Ectoedemia scabridae* Scoble, 1983: 24

*Fomoria scabridae* (Scoble, 1983) Diškus & Puplesis, 2003: 387

***Muhabbetana simiicola*** (Scoble, 1983) **comb. n.** AFR

*Ectoedemia simiicola* Scoble, 1983: 22

*Fomoria simiicola* (Scoble, 1983) Diškus & Puplesis, 2003: 387

***Muhabbetana stimulata*** (Meyrick, 1913) **comb. n.** AFR

*Nepticula stimulata* Meyrick, 1913: 326

*Ectoedemia stimulata* (Meyrick, 1913) Scoble, 1983: 21

*Fomoria stimulata* (Meyrick, 1913) Diškus & Puplesis, 2003: 386

***Muhabbetana umdoniella*** (Scoble, 1983) **comb. n.** AFR

*Ectoedemia umdoniella* Scoble, 1983: 24

*Fomoria umdoniella* (Scoble, 1983) Diškus & Puplesis, 2003: 387

***Muhabbetana wilkinsoni*** (Scoble, 1983) **comb. n.** AFR

*Ectoedemia wilkinsoni* Scoble, 1983: 21

*Fomoria wilkinsoni* (Scoble, 1983) Diškus & Puplesis, 2003: 387

***Muhabbetana euphorbiella* group** (new)

***Muhabbetana euphorbiella*** (Stainton, 1869) **comb. n.** WP

*Nepticula euphorbiella* Stainton, 1869b: 229

*Nepticula tergestina* Klimesch, 1940a: 79 (syn: Laštůvka & Laštůvka, 1997: 167)

*Stigmella euphorbiella* (Stainton, 1869) Gerasimov, 1952: 238

*Ectoedemia euphorbiella* (Stainton, 1869) van Nieukerken, 1986a: 17

*Fomoria euphorbiella* (Stainton, 1869) Diškus & Puplesis, 2003: 384

*Stigmella tergestina* (Klimesch, 1940) Hering, 1957: 434

*Ectoedemia tergestina* (Klimesch, 1940) van Nieukerken, 1986a: 17

***Muhabbetana jubae*** (Walsingham, 1908) **comb. n.** WP

*Stigmella jubae* Walsingham, 1908a: 1011 ^2^

*Nepticula jubae* (Walsingham, 1908) Rebel, 1910: 364

*Trifurcula jubae* (Walsingham, 1908) Klimesch, 1977: 197

*Ectoedemia jubae* (Walsingham, 1908) van Nieukerken, 1986b: 17

*Fomoria jubae* (Walsingham, 1908) Diškus & Puplesis, 2003: 384

***Muhabbetana nigrifasciata*** (Walsingham, 1908) **comb. n.** WP

*Stigmella nigrifasciata* Walsingham, 1908a: 1011 ^2^

*Nepticula nigrifasciata* (Walsingham, 1908) Rebel, 1910: 364

*Dechtiria nigrifasciata* (Walsingham, 1908) Klimesch, 1972: 1

*Trifurcula nigrifasciata* (Walsingham, 1908) Klimesch, 1977: 200

*Fomoria nigrifasciata* (Walsingham, 1908) Diškus & Puplesis, 2003: 387

*Ectoedemia nigrifasciata* (Walsingham, 1908) van Nieukerken, 1986b: 17

***Muhabbetana vincamajorella*** (Hartig, 1964) **comb. n.** WP

*Nepticula vincamajorella* Hartig, 1964: 8

*Fomoria vincamajorella* (Hartig, 1964) Diškus & Puplesis, 2003: 389

*Ectoedemia vincamajorella* (Hartig, 1964) van Nieukerken, 1986a: 17

***Muhabbetana* – unplaced species**

***Muhabbetana bicarina*** (Scoble, 1983) **comb. n.** AFR

*Ectoedemia bicarina* Scoble, 1983: 27

*Fomoria bicarina* (Scoble, 1983) Diškus & Puplesis, 2003: 389

***Muhabbetana capensis*** (Scoble, 1983) **comb. n.** AFR

*Ectoedemia capensis* Scoble, 1983: 28

*Fomoria capensis* (Scoble, 1983) Diškus & Puplesis, 2003: 389

***Muhabbetana craspedota*** (Vári, 1963) **comb. n.** AFR

*Stigmella craspedota* Vári, 1963: 73

*Ectoedemia craspedota* (Vári, 1963) Scoble, 1983: 27

*Fomoria craspedota* (Vári, 1963) Diškus & Puplesis, 2003: 388

***Muhabbetana crispae*** (Scoble, 1983) **comb. n.** AFR

*Ectoedemia crispae* Scoble, 1983: 31

*Fomoria crispae* (Scoble, 1983) Diškus & Puplesis, 2003: 390

***Muhabbetana denticulata*** (Scoble, 1983) **comb. n.** AFR

*Ectoedemia denticulata* Scoble, 1983: 26

*Fomoria denticulata* (Scoble, 1983) Diškus & Puplesis, 2003: 389

***Muhabbetana digitata*** (Scoble, 1983) **comb. n.** AFR

*Ectoedemia digitata* Scoble, 1983: 27

*Fomoria digitata* (Scoble, 1983) Diškus & Puplesis, 2003: 389

***Muhabbetana gymnosporiae*** (Vári, 1955) **comb. n.** AFR

*Stigmella gymnosporiae* Vári, 1955: 334

*Ectoedemia gymnosporiae* (Vári, 1955) Scoble, 1983: 29

*Fomoria gymnosporiae* (Vári, 1955) Diškus & Puplesis, 2003: 388

***Muhabbetana insulata*** (Meyrick, 1911) **comb. n.** AFR

*Nepticula insulata* Meyrick, 1911b: 79

*Ectoedemia insulata* (Meyrick, 1911) Scoble, 1983: 29

*Fomoria insulata* (Meyrick, 1911) Diškus & Puplesis, 2003: 388

***Muhabbetana kowynensis*** (Scoble, 1983) **comb. n.** AFR

*Ectoedemia kowynensis* Scoble, 1983: 30

*Fomoria kowynensis* (Scoble, 1983) Diškus & Puplesis, 2003: 389

***Muhabbetana limburgensis*** (Scoble, 1983) **comb. n.** AFR

*Ectoedemia limburgensis* Scoble, 1983: 28

*Fomoria limburgensis* (Scoble, 1983) Diškus & Puplesis, 2003: 389

***Muhabbetana nigrisquama*** (Scoble, 1983) **comb. n.** AFR

*Ectoedemia nigrisquama* Scoble, 1983: 26

*Fomoria nigrisquama* (Scoble, 1983) Diškus & Puplesis, 2003: 389

***Muhabbetana nylstroomensis*** (Scoble, 1983) **comb. n.** AFR

*Ectoedemia nylstroomensis* Scoble, 1983: 30

*Fomoria nylstroomensis* (Scoble, 1983) Diškus & Puplesis, 2003: 389

***Muhabbetana psarodes*** (Vári, 1963) **comb. n.** AFR

*Stigmella psarodes* Vári, 1963: 70

*Ectoedemia psarodes* (Vári, 1963) Scoble, 1983: 29

*Fomoria psarodes* (Vári, 1963) Diškus & Puplesis, 2003: 388

***Muhabbetana rhabdophora*** (Scoble, 1983) **comb. n.** AFR

*Ectoedemia rhabdophora* Scoble, 1983: 31

*Fomoria rhabdophora* (Scoble, 1983) Diškus & Puplesis, 2003: 390

***Muhabbetana royenicola*** (Vári, 1955) **comb. n.** AFR

*Stigmella royenicola* Vári, 1955: 335

*Ectoedemia royenicola* (Vári, 1955) Scoble, 1983: 25

*Fomoria royenicola* (Vári, 1955) Diškus & Puplesis, 2003: 388

***Muhabbetana subnitescens*** (Meyrick, 1937) **comb. n.** AFR

*Trifurcula subnitescens* Meyrick, 1937: 90

*Ectoedemia subnitescens* (Meyrick, 1937) Scoble, 1983: 28

*Fomoria subnitescens* (Meyrick, 1937) Diškus & Puplesis, 2003: 387

***Muhabbetana undatae*** (Scoble, 1983) **comb. n.** AFR

*Ectoedemia undatae* Scoble, 1983: 27

*Fomoria undatae* (Scoble, 1983) Diškus & Puplesis, 2003: 389

***Parafomoria*** Borkowski, 1975: 498 (TS/OD: *Nepticula helianthemella* Herrich-Schäffer, 1860: 60)

*Parafomoria* van Nieukerken, 1983b: 454 JH of *Parafomoria* Borkowski, 1975

***Parafomoria liguricella* group** (new)

***Parafomoria ladaniphila*** (Mendes, 1910) van Nieukerken, 1983b: 468 WP

*Nepticula ladaniphila* Mendes, 1910a: 102

*Stigmella ladaniphila* (Mendes, 1910) Klimesch, 1948a: 170

*Ectoedemia ladaniphila* (Mendes, 1910) Gómez Bustillo, 1981: 19

***Parafomoria liguricella*** (Klimesch, 1948) van Nieukerken, 1983b: 466 ^29^ WP

*Stigmella liguricella* Klimesch, 1948a: 170 ^29^

***Parafomoria tingitella*** (Walsingham, 1904) van Nieukerken, 1983b: 469 WP

*Nepticula tingitella* Walsingham, 1904: 8

*Stigmella tingitella* (Walsingham, 1904) Gerasimov, 1952: 264

***Parafomoria helianthemella* group** (new)

***Parafomoria cistivora*** (Peyerimhoff, 1871) van Nieukerken, 1983b: 458 WP

*Nepticula cistivora* Peyerimhoff, 1871: 414

*Stigmella cistivora* (Peyerimhoff, 1871) Suire, 1951: 71

***Parafomoria fumanae*** A. Laštuvka & Z. Laštuvka, 2005b: 15 WP

***Parafomoria halimivora*** van Nieukerken, 1985a: 24 WP

***Parafomoria helianthemella*** (Herrich-Schäffer, 1860) Borkowski, 1975: 498 (Fig. 34) WP

*Nepticula helianthemella* Herrich-Schäffer, 1860: 60

*Stigmella helianthemella* (Herrich-Schäffer, 1860) Klimesch, 1948a: 171

*Trifurcula helianthemella* (Herrich-Schäffer, 1860) Leraut, 1980: 49

***Parafomoria pseudocistivora*** van Nieukerken, 1983b: 460 WP

***Etainia*** Beirne, 1945: 208 (TS/OD: *Lyonetia sericopeza* Zeller, 1839)

*Obrussa* Braun, 1915: 196 JH of *Obrussa* Heyden, 1891 (Lepidoptera: Geometridae) (TS/M: *Nepticula ochrefasciella* Chambers, 1873) (syn: van Nieukerken, 1986a: 16)

***Etainia albibimaculella*** (Larsen, 1927) Puplesis & Diškus, 1996a: 5 WP,NEA

*Nepticula albibimaculella* Larsen, 1927: 5

*Stigmella albibimaculella* (Larsen, 1927) Hering, 1957: 112

*Trifurcula albibimaculella* (Larsen, 1927) Johansson, 1971: 246

*Ectoedemia albibimaculella* (Larsen, 1927) van Nieukerken, 1986a: 16

***Etainia biarmata*** Puplesis, 1994: 233 WP

*Ectoedemia biarmata* (Puplesis, 1994) van Nieukerken & Laštůvka, 2002: 89

***Etainia decentella*** (Herrich-Schäffer, 1855) Beirne, 1945: 207 WP

*Nepticula decentella* Herrich-Schäffer, 1855a: 358

*Nepticula monspessulanella* Jäckh, 1951: 171 (syn: van Nieukerken, 1986a: 16)

*Stigmella decentella* (Herrich-Schäffer, 1855) Gerasimov, 1952: 234

*Trifurcula decentella* (Herrich-Schäffer, 1855) Johansson, 1971: 246

*Ectoedemia decentella* (Herrich-Schäffer, 1855) van Nieukerken, 1986a: 16

*Stigmella monspessulanella* (Jäckh, 1951) Hering, 1957: 19

***Etainia leptognathos*** Puplesis & Diškus, 1996a: 44 WP

*Ectoedemia leptognathos* (Puplesis & Diškus, 1996) van Nieukerken & Laštůvka, 2002: 89

***Etainia louisella*** (Sircom, 1849) Bradley et al., 1972: 3 WP

*Nepticula louisella* Sircom, 1849: XIII

*Nepticula sphendamni* Hering, 1937: 561 (syn: van Nieukerken, 1986a: 16)

*Ectoedemia louisella* (Sircom, 1849) van Nieukerken, 1986a: 16

*Stigmella sphendamni* (Hering, 1937) Klimesch, 1951b: 64

*Trifurcula sphendamni* (Hering, 1937) Johansson, 1971: 246

*Etainia sphendamni* (Hering, 1937) Bradley et al., 1972: 3

***Etainia obtusa*** Puplesis & Diškus, 1996a: 46 WP

*Ectoedemia obtusa* (Puplesis & Diškus, 1996) Krenek, 2000: 36

***Etainia sericopeza*** (Zeller, 1839) Beirne, 1945: 207 (Fig. 35) WP,[NEA]

*Lyonetia sericopeza* Zeller, 1839: 215

*Oecophora sericopezella* Duponchel, 1843: 344 UE

*Tinea maryella* Duponchel, 1843: 464 (syn: Frey, 1857: 402)

*Nepticula acerella* Goureau, 1860: xxiii (syn: Joannis, 1915: 131)

*Nepticula sericopeza* (Zeller, 1839) Heyden, 1843: 209

*Stigmella sericopeza* (Zeller, 1839) Walsingham, 1916: 160

*Trifurcula sericopeza* (Zeller, 1839) Johansson, 1971: 246

*Obrussa sericopeza* (Zeller, 1839) Wilkinson & Scoble, 1979: 101

*Ectoedemia sericopeza* (Zeller, 1839) van Nieukerken, 1986b: 16

*Lyonetia sericopezella* (Duponchel, 1843) Duponchel, 1844: 378

‡ Stigmella sericopeza f. palliolella Le Marchand, 1944: 358

***Etainia capesella*** (Puplesis in Puplesis & Ivinskis, 1985) Puplesis, 1994: 232 EP

*Obrussa capesella* Puplesis in Puplesis & Ivinskis, 1985: 39

*Ectoedemia capesella* (Puplesis in Puplesis & Ivinskis, 1985) Hirano, 2013: 93

***Etainia peterseni*** (Puplesis in Puplesis & Ivinskis, 1985) Puplesis, 1994: 231 EP

*Obrussa peterseni* Puplesis in Puplesis & Ivinskis, 1985: 41

*Ectoedemia peterseni* (Puplesis in Puplesis & Ivinskis, 1985) Hirano, 2013: 92

***Etainia sabina*** (Puplesis in Puplesis & Ivinskis, 1985) Puplesis, 1994: 231 EP

*Obrussa sabina* Puplesis in Puplesis & Ivinskis, 1985: 43

*Ectoedemia sabina* (Puplesis in Puplesis & Ivinskis, 1985) online comb.

***Etainia trifasciata*** (Matsumura, 1931) Diškus & Puplesis, 2003: 408 ^59^ EP

*Nepticula trifasciata* Matsumura, 1931: 1114

*Obrussa tigrinella* Puplesis in Puplesis & Ivinskis, 1985: 40 **syn. n.** ^59^

*Stigmella trifasciata* (Matsumura, 1931) Kuroko, 1982a: 448

*Ectoedemia trifasciata* (Matsumura, 1931) Hirano, 2013: 93

*Ectoedemia tigrinella* (Puplesis in Puplesis & Ivinskis, 1985) Hirano, 2013: 92

*Etainia tigrinella* (Puplesis in Puplesis & Ivinskis, 1985) Puplesis, 1994: 232

***Etainia crypsixantha*** (Meyrick, 1918) Vári & Kroon, 1986: 153 AFR

*Nepticula crypsixantha* Meyrick, 1918a: 43

*Obrussa crypsixantha* (Meyrick, 1918) Scoble, 1983: 17

*Ectoedemia crypsixantha* (Meyrick, 1918) online comb.

***Etainia krugerensis*** (Scoble, 1983) Vári & Kroon, 1986: 153 AFR

*Obrussa krugerensis* Scoble, 1983: 19

*Ectoedemia krugerensis* (Scoble, 1983) online comb.

***Etainia nigricapitella*** (Janse, 1948) Vári & Kroon, 1986: 153 AFR

*Nepticula nigricapitella* Janse, 1948: 170

*Obrussa nigricapitella* (Janse, 1948) Scoble, 1983: 18

*Ectoedemia nigricapitella* (Janse, 1948) online comb.

***Etainia zimbabwiensis*** (Scoble, 1983) Vári & Kroon, 1986: 153 AFR

*Obrussa zimbabwiensis* Scoble, 1983: 18

*Ectoedemia zimbabwiensis* (Scoble, 1983) online comb.

***Etainia ochrefasciella*** (Chambers, 1873) Puplesis & Diškus, 1996a: 4 NEA

*Nepticula ochrefasciella* Chambers, 1873: 128

*Obrussa ochrefasciella* (Chambers, 1873) Braun, 1915: 196

*Ectoedemia ochrefasciella* (Chambers, 1873) van Nieukerken, 1986a: 19

***Acalyptris*** Meyrick, 1921a: 410 (TS/OD,M: *Acalyptris psammophricta* Meyrick, 1921: 410)

*Microcalyptris* Braun, 1925b: 224 (TS/OD,M: *Microcalyptris scirpi* Braun, 1925: 225) (syn: van Nieukerken, 1986a: 14)

*Weberia* Müller-Rutz, 1934a: 122 JH of *Weberia* Robineau-Desvoidy, 1830 (Diptera: Tachinidae) (TS/OD,M: *Weberia platani* Müller-Rutz, 1934: 122) (syn: van Nieukerken, 1986a: 14)

*Niepeltia* Strand, 1934: 241; RN for *Weberia* Müller-Rutz, 1934 (syn: van Nieukerken, 1986a: 14)

*Weberina* Müller-Rutz, 1934b: Errata, 148; RN for *Weberia* Müller-Rutz, 1934 (syn: van Nieukerken, 1986a: 14)

***Acalyptris scirpi* group** (new)

***Acalyptris bicornutus*** (Davis, 1978) Puplesis & Robinson, 2000: 53 NEA

*Microcalyptris bicornutus* Davis, 1978: 212

***Acalyptris bipinnatellus*** (Wilkinson, 1979) van Nieukerken, 1986a: 16 NEA

*Microcalyptris bipinnatellus* Wilkinson, 1979: 75

***Acalyptris lotella*** (Wagner, 1987) Diškus & Puplesis, 2003: 397 NEA

*Microcalyptris lotella* Wagner, 1987: 278

***Acalyptris punctulata*** (Braun, 1910) Diškus & Puplesis, 2003: 393 NEA

*Nepticula punctulata* Braun, 1910: 174

*Microcalyptris punctulata* (Braun, 1910) Wilkinson, 1979: 71

***Acalyptris scirpi*** (Braun, 1925) Diškus & Puplesis, 2003: 393 NEA

*Microcalyptris scirpi* Braun, 1925b: 225

***Acalyptris thoracealbella*** (Chambers, 1873) Diškus & Puplesis, 2003: 393 NEA

*Microcalyptris thoracealbella* (Chambers, 1873) Davis, 1978: 214

*Nepticula thoracealbella* Chambers, 1873: 127

*Nepticula badiocapitella* Chambers, 1876: 160 (syn: Braun, 1917: 189)

***Acalyptris tenuijuxtus*** (Davis, 1978) Puplesis & Robinson, 2000: 51 NEA,NEO

*Microcalyptris tenuijuxtus* Davis, 1978: 216

***Acalyptris basicornis*** Remeikis & Stonis in Stonis et al., 2013d: 102 NEO

***Acalyptris basihastatus*** Puplesis & Diškus in Puplesis et al., 2002: 29 NEO

***Acalyptris bifidus*** Puplesis & Robinson, 2000: 50 NEO

***Acalyptris bovicorneus*** Puplesis & Robinson, 2000: 45 NEO

***Acalyptris caribbicus*** Diškus & Stonis in Stonis et al., 2013d: 106 NEO

***Acalyptris dominicanus*** Remeikis & Stonis in Stonis & Remeikis, 2015: 85 NEO

***Acalyptris fortis*** Puplesis & Robinson, 2000: 47 NEO

***Acalyptris hispidus*** Puplesis & Robinson, 2000: 48 NEO

***Acalyptris janzeni*** van Nieukerken & Nishida in van Nieukerken et al., 2016: 55 NEO

***Acalyptris lascuevella*** Puplesis & Robinson, 2000: 49 NEO

***Acalyptris laxibasis*** Puplesis & Robinson, 2000: 52 NEO

***Acalyptris martinheringi*** Puplesis & Robinson, 2000: 46 NEO

***Acalyptris nigrisignum*** Remeikis & Stonis in Stonis & Remeikis, 2015: 79 NEO

***Acalyptris novenarius*** Puplesis & Robinson, 2000: 48 NEO

***Acalyptris paradividua*** Šimkevičiūtė & Stonis in Šimkevičiūtė et al., 2009: 272 NEO

***Acalyptris peteni*** Diškus & Stonis in Stonis et al., 2013d: 102 NEO

***Acalyptris pseudohastatus*** Puplesis & Diškus in Puplesis et al., 2002: 30 NEO

***Acalyptris statuarius*** Diškus & Stonis in Stonis et al., 2013d: 109 NEO

***Acalyptris terrificus*** Šimkevičiūtė & Stonis in Šimkevičiūtė et al., 2009: 275 NEO

***Acalyptris trifidus*** Puplesis & Robinson, 2000: 50 NEO

***Acalyptris trigonijuxtus*** Remeikis & Stonis in Stonis & Remeikis, 2015: 83 NEO

***Acalyptris unicornis*** Puplesis & Robinson, 2000: 51 NEO

***Acalyptris staticis* group** (van Nieukerken, 2007a: 17)

***Acalyptris lesbia*** van Nieukerken & Hull in van Nieukerken, 2007a: 22 WP

***Acalyptris limoniastri*** van Nieukerken, 2007a: 23 WP

***Acalyptris limonii*** Z. Laštuvka & A. Laštuvka, 1998: 314 WP

***Acalyptris maritima*** A. Laštuvka & Z. Laštuvka, 1997: 119 WP

***Acalyptris pyrenaica*** A. Laštuvka & Z. Laštuvka, 1993: 158 WP

***Acalyptris staticis*** (Walsingham, 1908) van Nieukerken, 1986b: 14 WP

*Stigmella staticis* Walsingham, 1908a: 1009 ^1^

*Nepticula staticis* (Walsingham, 1908) Rebel, 1910: 373

***Acalyptris psammophricta* group** (new)

*Acalyptris repeteki* group (Puplesis, 1988: 509)

***Acalyptris falkovitshi*** (Puplesis, 1984) van Nieukerken, 1986a: 14 ^60^ WP,EP

*Microcalyptris falkovitshi* Puplesis, 1984c: 499

*Microcalyptris turanicus* Puplesis, 1984c: 497 (syn: van Nieukerken, 2010: 501)

*Microcalyptris vittatus* Puplesis, 1984c: 491 **syn. n.** ^60^

*Microcalyptris arenosus* Falkovitsh, 1986: 168 **syn. n.** ^60^

*Acalyptris turanicus* (Puplesis, 1984) van Nieukerken, 1986a: 14

*Acalyptris vittatus* (Puplesis, 1984) van Nieukerken, 1986a: 14

*Acalyptris arenosus* (Falkovitsh, 1986) Puplesis, 1990: 66

***Acalyptris galinae*** (Puplesis, 1984) van Nieukerken, 1986a: 14 WP,EP

*Microcalyptris galinae* Puplesis, 1984c: 502

*Microcalyptris galinae mesasiaticus* Puplesis, 1984c: 503

*Acalyptris galinae mesasiaticus* (Puplesis, 1984) Puplesis, 1990: 84

***Acalyptris pallens*** (Puplesis, 1984) van Nieukerken, 1986a: 14 WP,EP

*Microcalyptris pallens* Puplesis, 1984c: 501

***Acalyptris psammophricta*** Meyrick, 1921a: 410 WP,EP,OR

*Microcalyptris lvovskyi* Puplesis, 1984c: 494 (syn: van Nieukerken, 2010: 501)

*Acalyptris lvovskyi* (Puplesis, 1984) van Nieukerken, 1986a: 14

***Acalyptris repeteki*** (Puplesis, 1984) van Nieukerken, 1986a: 14 WP

*Microcalyptris repeteki* Puplesis, 1984c: 494

***Acalyptris turcomanicus*** (Puplesis, 1984) van Nieukerken, 1986a: 14 WP

*Microcalyptris turcomanicus* Puplesis, 1984c: 499

***Acalyptris shafirkanus* group** (Puplesis, 1988: 506)

***Acalyptris brevis*** Puplesis, 1990: 86 WP

***Acalyptris desertellus*** (Puplesis, 1984) van Nieukerken, 1986a: 14 WP

*Microcalyptris desertellus* Puplesis, 1984c: 493

***Acalyptris egidijui*** Puplesis, 1990: 87 WP

***Acalyptris kizilkumi*** (Falkovitsh, 1986) Puplesis, 1990: 86 WP,EP

*Microcalyptris kizilkumi* Falkovitsh, 1986: 167

***Acalyptris piculus*** Puplesis, 1990: 85 EP

***Acalyptris shafirkanus*** (Puplesis, 1984) van Nieukerken, 1986a: 14 WP

*Microcalyptris shafirkanus* Puplesis, 1984c: 493

***Acalyptris vannieukerkeni*** Puplesis, 1994: 218 WP

***Acalyptris platani* group** (van Nieukerken, 2007a: 7)

***Acalyptris gielisi*** van Nieukerken, 2010: 500 WP

***Acalyptris loranthella*** (Klimesch, 1937) van Nieukerken, 1986a: 14 WP

*Nepticula loranthella* Klimesch, 1937: 33

*Stigmella loranthella* (Klimesch, 1937) Klimesch, 1948b: 78

*Weberina loranthella* (Klimesch, 1937) Szőcs, 1978: 268

*Niepeltia loranthella* (Klimesch, 1937) van Achterberg, 1983: 30

***Acalyptris minimella*** (Rebel, 1926) van Nieukerken, 1986a: 14 WP

*Trifurcula minimella* Rebel, 1926: (110)

*Weberina lentiscella* Groschke, 1944: 117 (syn: Klimesch, 1978a: 256)

*Nepticula minimella* (Rebel, 1926); Klimesch, 1953a: 162 JSH of *Nepticula minimella* Chambers, 1873

*Niepeltia lentiscella* (Groschke, 1944) Hering, 1957: 781

*Niepeltia minimella* (Rebel, 1926) Scoble, 1980a: 207

***Acalyptris pistaciae*** van Nieukerken, 2007a: 14 WP

***Acalyptris platani*** (Müller-Rutz, 1934) van Nieukerken, 1986a: 14 (Fig. 36) WP

*Weberia platani* Müller-Rutz, 1934a: 122

*Niepeltia platani* (Müller-Rutz, 1934) Strand, 1934: 241

*Weberina platani* (Müller-Rutz, 1934) Müller-Rutz, 1934b: slip

*Trifurcula platani* (Müller-Rutz, 1934) Klimesch, 1978a: 253

***Acalyptris acontarcha*** (Meyrick, 1926) Diškus & Puplesis, 2003: 393 OR

*Nepticula acontarcha* Meyrick, 1926: 295

*Stigmella acontarcha* (Meyrick, 1926) Fletcher, 1933: 82

***Acalyptris auratilis*** Puplesis & Diškus, 2003a: 219 OR

***Acalyptris clinomochla*** (Meyrick, 1934) Diškus & Puplesis, 2003: 394 OR

*Nepticula clinomochla* Meyrick, 1934a: 468

*Trifurcula clinomochla* (Meyrick, 1934) Gustafsson, 1976: 49

*Niepeltia clinomochla* (Meyrick, 1934) Scoble, 1980a: 216

***Acalyptris heteranthes*** (Meyrick, 1926) Diškus & Puplesis, 2003: 393 OR

*Nepticula heteranthes* Meyrick, 1926: 296

***Acalyptris melanospila*** (Meyrick, 1934) Puplesis & Diškus, 2003a: 218 OR

*Nepticula melanospila* Meyrick, 1934a: 468

***Acalyptris nigripexus*** Puplesis & Diškus, 2003: 220 OR

***Acalyptris acumenta*** (Scoble, 1980) Vári et al., 2002: 8 AFR

*Niepeltia acumenta* Scoble, 1980b: 213

***Acalyptris bispinata*** (Scoble, 1980) Vári et al., 2002: 8 AFR

*Niepeltia bispinata* Scoble, 1980b: 213

***Acalyptris combretella*** (Vári, 1955) Vári et al., 2002: 8 AFR

*Stigmella combretella* Vári, 1955: 332

*Niepeltia combretella* (Vári, 1955) Scoble, 1980a: 206

***Acalyptris fagarivora*** (Vári, 1955) Vári et al., 2002: 8 AFR

*Stigmella fagarivora* Vári, 1955: 334

*Niepeltia fagarivora* (Vári, 1955) Scoble, 1980a: 209

***Acalyptris fulva*** (Scoble, 1980) Vári et al., 2002: 8 AFR

*Niepeltia fulva* Scoble, 1980b: 214

***Acalyptris fuscofascia*** (Scoble, 1980) Vári et al., 2002: 8 AFR

*Niepeltia fuscofascia* Scoble, 1980b: 210

***Acalyptris krooni*** (Scoble, 1980) Vári et al., 2002: 8 AFR

*Niepeltia krooni* Scoble, 1980b: 212

***Acalyptris krugeri*** (Vári, 1963) Diškus & Puplesis, 2003: 394 AFR

*Stigmella krugeri* Vári, 1963: 71

***Acalyptris lanneivora*** (Vári, 1955) Vári et al., 2002: 8 AFR

*Stigmella lanneivora* Vári, 1955: 332

*Niepeltia lanneivora* (Vári, 1955) Scoble, 1980a: 215

***Acalyptris lorantivora*** (Janse, 1948) Vári et al., 2002: 8 AFR

*Nepticula lorantivora* Janse, 1948: 169

*Niepeltia lorantivora* (Janse, 1948) Scoble, 1980a: 211

***Acalyptris lundiensis*** (Scoble, 1980) Vári et al., 2002: 8 AFR

*Niepeltia lundiensis* Scoble, 1980b: 214

***Acalyptris mariepsensis*** (Scoble, 1980) Vári et al., 2002: 8 AFR

*Niepeltia mariepsensis* Scoble, 1980b: 214

***Acalyptris molleivora*** (Scoble, 1980) Vári et al., 2002: 8 AFR

*Niepeltia molleivora* Scoble, 1980b: 207

***Acalyptris obliquella*** (Scoble, 1980) Vári et al., 2002: 8 AFR

*Niepeltia obliquella* Scoble, 1980b: 209

***Acalyptris pundaensis*** (Scoble, 1980) Vári et al., 2002: 8 AFR

*Niepeltia pundaensis* Scoble, 1980b: 211

***Acalyptris rubiaevora*** (Scoble, 1980) Vári et al., 2002: 8 AFR

*Niepeltia rubiaevora* Scoble, 1980b: 208

***Acalyptris sellata*** (Scoble, 1980) Vári et al., 2002: 8 AFR

*Niepeltia sellata* Scoble, 1980b: 213

***Acalyptris umdoniensis*** (Scoble, 1980) Vári et al., 2002: 8 AFR

*Niepeltia umdoniensis* Scoble, 1980b: 210

***Acalyptris vacuolata*** (Scoble, 1980) Vári et al., 2002: 8 AFR

*Niepeltia vacuolata* Scoble, 1980b: 215

***Acalyptris vepricola*** (Vári, 1963) Vári et al., 2002: 8 AFR

*Stigmella vepricola* Vári, 1963: 68

*Niepeltia vepricola* (Vári, 1963) Scoble, 1983: 44

***Acalyptris vumbaensis*** (Scoble, 1980) Vári et al., 2002: 8 AFR

*Niepeltia vumbaensis* Scoble, 1980b: 207

***Acalyptris zeyheriae*** (Scoble, 1980) Vári et al., 2002: 8 AFR

*Niepeltia zeyheriae* Scoble, 1980b: 208

***Acalyptris latipennata* group** (Puplesis et al., 2002: 66)

***Acalyptris dividua*** Puplesis & Robinson, 2000: 54 NEO

***Acalyptris ecuadoriana*** Puplesis & Diškus in Puplesis et al., 2002: 27 NEO

***Acalyptris latipennata*** (Puplesis & Robinson, 2000) Puplesis et al., 2002: 66 NEO

*Fomoria latipennata* Puplesis & Robinson, 2000: 45

***Acalyptris onorei*** Puplesis & Diškus in Puplesis et al., 2002: 28 NEO

**unplaced *Acalyptris*** ^61^

***Acalyptris distaleus*** (Wilkinson, 1979) Diškus & Puplesis, 2003: 395 ^61^ NEA

*Microcalyptris distaleus* Wilkinson, 1979: 78

***Acalyptris postalatratus*** (Wilkinson, 1979) Diškus & Puplesis, 2003: 395 NEA

*Microcalyptris postalatratus* Wilkinson, 1979: 77

***Acalyptris amazonius*** Puplesis & Diškus in Puplesis et al., 2002: 32 NEO

***Acalyptris articulosus*** Puplesis & Diškus in Puplesis et al., 2002: 30 NEO

***Acalyptris insolentis*** Puplesis & Diškus in Puplesis et al., 2002: 33 NEO

***Acalyptris platygnathos*** Puplesis & Robinson, 2000: 54 NEO

***Acalyptris rotundus*** Puplesis & Diškus in Puplesis et al., 2002: 31 NEO

***Acalyptris yucatani*** Remeikis & Stonis in Stonis et al., 2013b: 227 NEO

***Zimmermannia*** Hering, 1940: 266 (TS/OD,M: *Ectoedemia liebwerdella* Zimmermann, 1940) ^62^

*Ectoedemia castaneae* group (Wilkinson & Newton, 1981: 72)

***Zimmermannia amani*** (Svensson, 1966) **comb. n.** WP,EP

*Ectoedemia amani* Svensson, 1966: 200

*Ectoedemia emendata* Puplesis, 1985c: 69 (syn: Puplesis, 1994: 15)

*Trifurcula amani* (Svensson, 1966) Johansson, 1971: 245

***Zimmermannia atrifrontella*** (Stainton, 1851) **comb. n.** (Fig. 37) WP

*Trifurcula atrifrontella* Stainton, 1851: 11

*Zimmermannia heringiella* Doets, 1947: 504 (syn: Klimesch, 1953a: 191)

*Ectoedemia atrifrontella* (Stainton, 1851) Klimesch, 1953b: 191

*Ectoedemia heringiella* (Doets, 1947) Klimesch, 1953b: 191

***Zimmermannia hispanica*** (van Nieukerken, 1985) **comb. n.** WP

*Ectoedemia hispanica* van Nieukerken, 1985b: 22

***Zimmermannia liebwerdella*** (Zimmermann, 1940) Hering, 1940: 266 WP

*Ectoedemia liebwerdella* Zimmermann, 1940: 264

***Zimmermannia liguricella*** (Klimesch, 1953) **comb. n.** WP

*Ectoedemia liguricella* Klimesch, 1953b: 194

***Zimmermannia longicaudella*** (Klimesch, 1953) **comb. n.** WP

*Ectoedemia longicaudella* Klimesch, 1953b: 193

*Stigmella peiuii* Nemeş, 1972: 153 (syn: van Nieukerken, 1985b: 21)

*Trifurcula longicaudella* (Klimesch, 1953) Johansson, 1971: 245

***Zimmermannia monemvasiae*** (van Nieukerken, 1985) **comb. n.** WP

*Ectoedemia monemvasiae* van Nieukerken, 1985b: 23

***Zimmermannia reichli*** (Z. Laštuvka & A. Laštuvka, 1998) **comb. n.** WP

*Ectoedemia reichli* Z. Laštuvka & A. Laštuvka, 1998: 316

***Zimmermannia vivesi*** (A. Laštuvka, Z. Laštuvka & van Nieukerken, 2010) **comb. n.** WP

*Ectoedemia vivesi* A. Laštuvka, Z. Laštuvka & van Nieukerken in van Nieukerken et al., 2010: 12

***Zimmermannia admiranda*** (Puplesis, 1984) **comb. n.** EP

*Ectoedemia admiranda* Puplesis, 1984b: 588

***Zimmermannia nuristanica*** (van Nieukerken, 1985) **comb. n.** EP

*Ectoedemia nuristanica* van Nieukerken, 1985b: 25

***Zimmermannia sivickisi*** (Puplesis, 1984) **comb. n.** EP

*Ectoedemia sivickisi* Puplesis, 1984b: 590

*Ectoedemia laura* Puplesis, 1985c: 68 (syn: Rocienė & Stonis, 2013: 108)

***Zimmermannia bosquella*** (Chambers, 1878) **stat rev., comb. n.** ^62^ NEA

*Nepticula bosquella* Chambers, 1878a: 106

*Nepticula bosqueella* Chambers, 1878b: 157 ISS

*Ectoedemia castaneae* Busck, 1913: 103 **syn. n.** ^62^

*Ectoedemia heinrichi* Busck, 1914: 149 **syn. n.** ^62^

*Ectoedemia helenella* Wilkinson, 1981: 105 **syn. n.** ^62^

*Ectoedemia bosquella* (Chambers, 1878) Braun, 1917: 200

*Opostega bosqueella* (Chambers, 1878) Dyar et al., 1903: 547

*Ectoedemia obrutella* sensu Wilkinson & Newton, 1981: 72 [misapplied]

***Zimmermannia grandisella*** (Chambers, 1880) **comb. n.** ^62^ NEA

*Nepticula grandisella* Chambers, 1880a: 193

*Ectoedemia chloranthis* Meyrick, 1928b: 462 **syn. n.** ^62^

*Ectoedemia acanthella* Wilkinson & Newton, 1981: 75 **syn. n.** ^62^

*Ectoedemia grandisella* (Chambers, 1880) Wilkinson, 1981: 96

***Zimmermannia mesoloba*** (Davis, 1978) **comb. n.** ^62^ NEA

*Ectoedemia mesoloba* Davis, 1978: 209

*Ectoedemia coruscella* Wilkinson, 1981: 99 **syn. n.** ^62^

***Zimmermannia obrutella*** (Zeller, 1873) **comb. n.** ^62^ NEA

*Trifurcula obrutella* Zeller, 1873: 316

*Ectoedemia piperella* Wilkinson & Newton, 1981: 77 **syn. n.** ^62^

*Ectoedemia reneella* Wilkinson, 1981: 104 **syn. n.** ^62^

*Ectoedemia obrutella* (Zeller, 1873) Busck, 1913: 103

***Zimmermannia phleophaga*** (Busck, 1914) **comb. n.** NEA

*Ectoedemia phleophaga* Busck, 1914: 3

***Ectoedemia*** Busck, 1907: 97 (TS/OD,M: *Ectoedemia populella* Busck, 1907: 98)

*Dechtiria* Beirne, 1945: 204 (TS/OD: *Tinea subbimaculella* Haworth, 1828: 583) (syn: Svensson, 1966: 200)

***Ectoedemia commiphorella* group** (Doorenweerd et al., 2015: 9)

***Ectoedemia commiphorella*** Scoble, 1978a: 82 AFR

***Ectoedemia expeditionis*** Mey, 2004: 30 AFR

***Ectoedemia mauni*** Scoble, 1979: 36 AFR

***Ectoedemia nigrimacula*** (Janse, 1948) Scoble, 1978a: 84 AFR

*Nepticula nigrimacula* Janse, 1948: 171

***Ectoedemia tersiusi*** Mey, 2004: 31 AFR

***Ectoedemia terebinthivora* group** (van Nieukerken, 1985b: 63)

***Ectoedemia terebinthivora*** (Klimesch, 1975) van Nieukerken, 1985b: 63 WP

*Trifurcula terebinthivora* Klimesch, 1975c: 19

***Ectoedemia populella* group** (Wilkinson & Newton, 1981: 41)

***Ectoedemia intimella*** (Zeller, 1848) Bradley et al., 1972: 3 WP

*Nepticula intimella* Zeller, 1848: 323

*Stigmella intimella* (Zeller, 1848) Fletcher & Clutterbuck, 1945: 61

*Dechtiria intimella* (Zeller, 1848) Beirne, 1945: 205

*Trifurcula intimella* (Zeller, 1848) Johansson, 1971: 245

***Ectoedemia insularis*** Puplesis, 1985c: 68 ^63^ EP

***Ectoedemia sinevi*** Puplesis, 1985c: 67 ^64^ EP

***Ectoedemia populella*** Busck, 1907: 98 NEA

***Ectoedemia hannoverella*** (Glitz, 1872) Borkowski, 1972a: Fig. 7 WP,EP

*Nepticula hannoverella* Glitz, 1872: 25

*Stigmella hannoverella* (Glitz, 1872) Klimesch, 1951b: 64

*Trifurcula hannoverella* (Glitz, 1872) Johansson, 1971: 245

***Ectoedemia canutus*** Wilkinson & Scoble, 1979: 81 NEA

***Ectoedemia turbidella*** (Zeller, 1848) Bradley et al., 1972: 3 ^65^ WP

Nepticula argyropeza var. turbidella Zeller, 1848: 321

‡ [no genus] *argyropeza* Herrich-Schäffer, 1853: pl106 NN

*Nepticula argyropezella* Herrich-Schäffer, 1855a: 357 UE

*Nepticula populi-albae* Hering, 1935: 7 (syn: van Nieukerken, 1985b: 31)

*Stigmella marionella* Ford, 1950: 39 (syn: Bradley et al., 1972: 3)

*Ectoedemia similigena* Puplesis, 1994: 180 **syn. n.** ^65^

*Dechtiria turbidella* (Zeller, 1848) Vári, 1950: 182

*Stigmella turbidella* (Zeller, 1848) Klimesch, 1951b: 64

*Trifurcula turbidella* (Zeller, 1848) Johansson, 1971: 245

*Stigmella populialbae* (Hering, 1935) Gerasimov, 1952: 252

*Ectoedemia populialbae* (Hering, 1935) Borkowski, 1975: 495

***Ectoedemia albida*** Puplesis, 1994: 179 WP

***Ectoedemia klimeschi*** (Skala, 1933) Borkowski, 1975: 495 (Fig. 38) WP

*Nepticula klimeschi* Skala, 1933a: 31

*Stigmella niculescui* Nemeş, 1970: 33 (syn: van Nieukerken, 1985b: 34)

*Stigmella klimeschi* (Skala, 1933) Gerasimov, 1952: 244

***Ectoedemia argyropeza*** (Zeller, 1839) Bradley et al., 1972: 3 WP,EP,[NEA]

*Lyonetia argyropeza* Zeller, 1839: 215

*Lyonetia argyropezella* Duponchel, 1844: 378 UE

*Nepticula apicella* Stainton, 1854: 300 (syn: Heinemann & Wocke, [1876]: 768) ^6^

*Nepticula argyropezella* Doubleday, 1859: 36 UE

*Nepticula turbulentella* Wocke, 1861: 129 URN

*Nepticula simplicella* Heinemann, 1862b: 319 (syn: van Nieukerken, 1985b: 35)

*Ectoedemia argyropeza downesi* Wilkinson & Scoble, 1979: 80

*Nepticula argyropeza* (Zeller, 1839) Zeller, 1848: 320

*Stigmella argyropeza* (Zeller, 1839) Fletcher & Clutterbuck, 1945: 61

*Dechtiria argyropeza* (Zeller, 1839) Emmet, 1971a: 243

*Trifurcula argyropeza* (Zeller, 1839) Johansson, 1971: 245

‡ Nepticula argyropeza ab. houzeaui Dufrane, 1942: 11

‡ Nepticula argyropeza ab. morosella Steudel in Steudel & Hoffmann, 1882: 244

***Ectoedemia subbimaculella* group - satellite taxa** (Doorenweerd et al., 2015)

*Ectoedemia preisseckeri* group (van Nieukerken, 1985b: 37)

***Ectoedemia arisi*** Puplesis, 1984a: 120 EP

***Ectoedemia scoblei*** Puplesis, 1984a: 122 EP

***Ectoedemia christopheri*** Puplesis, 1985c: 69; RN for *Ectoedemia wilkinsoni* Puplesis, 1984a EP

*Ectoedemia wilkinsoni* Puplesis, 1984a: 122; JPH of *Ectoedemia wilkinsoni* Scoble, 1983

***Ectoedemia trinotata*** (Braun, 1914) Wilkinson & Newton, 1981: 46 NEA

*Nepticula trinotata* Braun, 1914: 18

***Ectoedemia philipi*** Puplesis, 1984b: 590 EP

***Ectoedemia preisseckeri*** (Klimesch, 1941) Borkowski, 1975: 493 WP,EP

*Nepticula preisseckeri* Klimesch, 1941: 162

*Stigmella preisseckeri* (Klimesch, 1941) Hering, 1957: 1092

***Ectoedemia quadrinotata*** (Braun, 1917) Wilkinson & Scoble, 1979: 95 NEA

*Nepticula quadrinotata* Braun, 1917: 168

***Ectoedemia subbimaculella* group** (van Nieukerken, 1985b: 43)

***Ectoedemia gilvipennella*** (Klimesch, 1948) van Nieukerken, 1985b: 45 ^29^ WP

*Stigmella gilvipennella* Klimesch, 1948a: 168 ^29^

*Nepticula gilvipennella* (Klimesch, 1948) Szőcs, 1968: 228

***Ectoedemia quinquella*** (Bedell, 1848) Bradley et al., 1972: 2 WP

*Microsetia quinquella* Bedell, 1848: 1986

*Nepticula quinquella* (Bedell, 1848) Stainton, 1849: 29

*Dechtiria quinquella* (Bedell, 1848) Beirne, 1945: 206

*Stigmella quinquella* (Bedell, 1848) Gerasimov, 1952: 255

*Trifurcula quinquella* (Bedell, 1848) Johansson, 1971: 245

***Ectoedemia coscoja*** van Nieukerken, A. Laštuvka & Z. Laštuvka, 2010: 45 WP

***Ectoedemia algeriensis*** van Nieukerken, 1985b: 44 WP

***Ectoedemia leucothorax*** van Nieukerken, 1985b: 46 WP

***Ectoedemia haraldi*** (Soffner, 1942) Klimesch, 1975a: 864 WP

*Nepticula haraldi* Soffner, 1942: 56

*Stigmella prinophyllella* Le Marchand, 1946: 285 (syn: Le Marchand, 1948: 298)

*Stigmella haraldi* (Soffner, 1942) Hering, 1957: 867

*Trifurcula haraldi* (Soffner, 1942) Leraut, 1980: 49

***Ectoedemia pseudoilicis*** Z. Laštuvka & A. Laštuvka, 1998: 317 WP

***Ectoedemia ilicis*** (Mendes, 1910b) van Nieukerken, 1985b: 48 WP

*Nepticula ilicis* Mendes, 1910b: 164

*Stigmella ilicis* (Mendes, 1910b) Gerasimov, 1952: 243

***Ectoedemia heringella*** (Mariani, 1939) van Nieukerken, 1985b: 49 WP

*Nepticula heringella* Mariani, 1939: 5

*Stigmella heringella* (Mariani, 1939) Hering, 1957: 868

‡ Nepticula heringella f. alliatae Mariani, 1939: 7

***Ectoedemia alnifoliae*** van Nieukerken, 1985b: 50 WP

***Ectoedemia aligera*** Puplesis, 1985c: 67 EP

***Ectoedemia ermolaevi*** Puplesis, 1985c: 68 EP

***Ectoedemia cerviparadisicola*** Sato in Shinozaki et al., 2012: 578 EP

***Ectoedemia maculata*** Puplesis, 1987: 11 EP

***Ectoedemia rufifrontella*** (Caradja, 1920) van Nieukerken, 1987a: 142 WP

*Trifurcula rufifrontella* Caradja, 1920: 161

*Nepticula nigrosparsella* Klimesch, 1940b: 91 (syn: van Nieukerken, 1987a: 142)

*Stigmella nigrosparsella* (Klimesch, 1940b) Klimesch, 1948a: 170

*Ectoedemia nigrosparsella* (Klimesch, 1940b) Kasy, 1983: 5

*Ectoedemia albifasciella* complex (van Nieukerken, 1985b: 52) (next 4 species)

***Ectoedemia pubescivora*** (Weber, 1937) van Nieukerken, 1985b: 55 WP

*Nepticula pubescivora* Weber, 1937: 212

*Stigmella pubescivora* (Weber, 1937) Klimesch, 1948b: 73

*Trifurcula pubescivora* (Weber, 1937) Kasy, 1979: 4

***Ectoedemia albifasciella*** (Heinemann, 1871) Bradley et al., 1972: 3 WP

*Nepticula albifasciella* Heinemann, 1871: 222

*Nepticula subapicella* Stainton, 1886: 238 (syn: Emmet, 1974b: 274)

*Dechtiria albifasciella* (Heinemann, 1871) Beirne, 1945: 205

*Stigmella albifasciella* (Heinemann, 1871) Klimesch, 1951b: 66

*Trifurcula albifasciella* (Heinemann, 1871) Johansson, 1971: 245

***Ectoedemia contorta*** van Nieukerken, 1985b: 55 WP

***Ectoedemia cerris*** (Zimmermann, 1944) Szőcs, 1978: 266 WP

*Nepticula cerris* Zimmermann, 1944: 121

*Nepticula montissancti* Skala, 1948: 121 (syn: van Nieukerken, 1985b: 54)

*Stigmella cerris* (Zimmermann, 1944) Hering, 1957: 866

*Ectoedemia subbimaculella* complex (van Nieukerken, 1985b: 56) (next 4 species)

***Ectoedemia subbimaculella*** (Haworth, 1828) Bradley et al., 1972: 3 WP

*Tinea subbimaculella* Haworth, 1828: 583

‡ *Microsetia nigrociliella* Stephens, 1829: 208 NN (syn: van Nieukerken, 1985b: 57)

*Microsetia nigrociliella* Stephens, 1834: 267 (syn: van Nieukerken, 1985b: 57)

‡ *Nepticula cursoriella* Heyden, 1843: 209 NN (syn: Herrich-Schäffer, 1855a: 356)

*Nepticula cursoriella* Zeller, 1848: 326 (syn: Herrich-Schäffer, 1855a: 356)

*Nepticula bistrimaculella* Heyden, 1861: 40 (syn: van Nieukerken & Johansson, 1987: 462)

*Microsetia subbimaculella* (Haworth, 1828) Stephens, 1829: 208

*Nepticula subbimaculella* (Haworth, 1828) Stainton, 1849: 29

*Stigmella subbimaculella* (Haworth, 1828) Fletcher & Clutterbuck, 1945: 61

*Dechtiria subbimaculella* (Haworth, 1828) Beirne, 1945: 206

*Trifurcula subbimaculella* (Haworth, 1828) Johansson, 1971: 245

*Stigmella bistrimaculella* (Heyden, 1861) Gerasimov, 1952: 231

***Ectoedemia phyllotomella*** (Klimesch, 1948) van Nieukerken, 1985b: 62 ^29^ WP

*Stigmella phyllotomella* Klimesch, 1948a: 166 ^29^

***Ectoedemia heringi*** (Toll, 1934) Borkowski, 1975: 491 WP

*Nepticula heringi* Toll, 1934a: 3

*Nepticula quercifoliae* Toll, 1934b: 71 (syn: Borkowski, 1975: 491)

*Nepticula sativella* Klimesch, 1936: 208 (syn: van Nieukerken, 1985b: 59)

*Nepticula zimmermanni* Hering, 1942: 26 (syn: van Nieukerken, 1985b: 59)

*Stigmella heringi* (Toll, 1934) Hering, 1957: 867

*Trifurcula heringi* (Toll, 1934) Kasy, 1979: 4

*Stigmella sativella* (Klimesch, 1936) Klimesch, 1948b: 74

*Stigmella quercifoliae* (Toll, 1934) Hering, 1957: 867

*Ectoedemia quercifoliae* (Toll, 1934) Bradley et al., 1972: 3

*Stigmella zimmermanni* (Hering, 1942) Klimesch, 1951a: 65

*Trifurcula zimmermanni* (Hering, 1942) Kasy, 1979: 4

*Ectoedemia zimmermanni* (Hering, 1942) Szőcs, 1981: 210

***Ectoedemia liechtensteini*** (Zimmermann, 1944) Szőcs, 1978: 266 WP

*Nepticula liechtensteini* Zimmermann, 1944: 119

*Stigmella liechtensteini* (Zimmermann, 1944) Hering, 1957: 866

***Ectoedemia platanella* group** (Wilkinson & Newton, 1981: 51)

***Ectoedemia similella*** (Braun, 1917) Wilkinson & Newton, 1981: 56 NEA

*Nepticula similella* Braun, 1917: 188

***Ectoedemia platanella*** (Clemens, 1861) Wilkinson & Scoble, 1979: 89 NEA

*Nepticula platanella* Clemens, 1861: 83

*Nepticula maximella* Chambers, 1873: 126 (syn: Braun, 1917: 187)

***Ectoedemia clemensella*** (Chambers, 1873) Wilkinson & Scoble, 1979: 86 NEA

*Nepticula clemensella* Chambers, 1873: 125

***Ectoedemia virgulae*** (Braun, 1927) Wilkinson & Newton, 1981: 59 NEA

*Nepticula virgulae* Braun, 1927: 198

***Ectoedemia ornatella* group** (Puplesis, 1984b: 584)

***Ectoedemia ivinskisi*** Puplesis, 1984a: 120 EP

***Ectoedemia olvina*** Puplesis, 1984a: 119 EP

***Ectoedemia ornatella*** Puplesis, 1984a: 120 EP

***Ectoedemia suberis* group** (van Nieukerken, 1985b: 38)

***Ectoedemia chasanella*** Puplesis, 1984a: 124 EP

***Ectoedemia aegilopidella*** (Klimesch, 1978) van Nieukerken, 1985b: 42 WP

*Trifurcula aegilopidella* Klimesch, 1978b: 269

***Ectoedemia caradjai*** (Groschke, 1944) Szőcs, 1981: 211 WP

*Nepticula caradjai* Groschke, 1944: 118

*Stigmella caradjai* (Groschke, 1944) Klimesch, 1951b: 65

*Trifurcula caradjai* (Groschke, 1944) Klimesch, 1978a: 250

***Ectoedemia andalusiae*** van Nieukerken, 1985b: 41 WP

***Ectoedemia suberis*** (Stainton, 1869) van Nieukerken, 1985b: 40 WP

*Nepticula suberis* Stainton, 1869b: 229

*Nepticula viridella* Mendes, 1910b: 165 (syn: van Nieukerken, 1985b: 40)

*Stigmella suberis* (Stainton, 1869) Gerasimov, 1952: 262

*Stigmella viridella* (Mendes, 1910) Gerasimov, 1952: 260

***Ectoedemia phaeolepis*** van Nieukerken, A. Laštuvka & Z. Laštuvka, 2010: 38 WP

***Ectoedemia hendrikseni*** A. Laštuvka, Z. Laštuvka & van Nieukerken in van Nieukerken et al., 2010: 31 WP

***Ectoedemia heckfordi*** van Nieukerken, A. Laštuvka & Z. Laštuvka, 2010: 34 WP

***Ectoedemia ortiva*** Rocienė & Stonis, 2013: 76 EP

***Ectoedemia paraortiva*** Rocienė & Stonis in Stonis & Rocienė, 2013: 210 EP

***Ectoedemia angulifasciella* group** (Wilkinson et al., 1983: 211)

*Ectoedemia rubifoliella* group (Wilkinson & Newton, 1981: 61)

*Ectoedemia occultella* group (van Nieukerken, 1985b: 78)

***Ectoedemia hexapetalae*** (Szőcs, 1957) van Nieukerken, 1985b: 68 WP

Nepticula utensis var. hexapetalae Szőcs, 1957: 322

*Nepticula hexapetalae* Szőcs, 1957 (Szőcs, 1965:79)

*Trifurcula hexapetalae* (Szőcs, 1957) Kasy, 1980: 47

***Ectoedemia rosae*** van Nieukerken & Berggren, 2011: 182 WP

***Ectoedemia rosiphila*** Puplesis in Puplesis et al., 1992: 55 EP

***Ectoedemia marmaropa*** (Braun, 1925) Wilkinson & Newton, 1981: 49 NEA

*Nepticula marmaropa* Braun, 1925b: 225

***Ectoedemia spiraeae*** Gregor & Povolny, 1983: 174 ^66^ WP,EP?

‡ *Stigmella spireae* Gregor & Povolny, 1955: 124 NNLM

‡ *Nepticula spireae* (Gregor & Povolny, 1955) Szőcs, 1968: 229 NNLM

***Ectoedemia jacutica*** Puplesis, 1988: 26 ^66^ EP

***Ectoedemia agrimoniae*** (Frey, 1858) Bradley et al., 1972: 2 WP

*Nepticula agrimoniae* Frey, 1858c: 44

*Nepticula agrimoniae* Hofmann, 1858: 188

*Nepticula agrimoniella* Herrich-Schäffer, 1860: 60 UE

*Dechtiria agrimoniae* (Frey, 1858) Beirne, 1945: 205

*Stigmella agrimoniae* (Frey, 1858) Gerasimov, 1952: 224

*Trifurcula agrimoniae* (Frey, 1858) Johansson, 1971: 245

*Stigmella agrimoniella* (Herrich-Schäffer, 1860) Le Marchand, 1946a: 217

‡ *Nepticula agrimomella* Rössler, 1881: 337 ISS

***Ectoedemia nyssaefoliella*** (Chambers, 1880) Wilkinson & Newton, 1981: 67 NEA

*Nepticula nyssaefoliella* Chambers, 1880b: 66

***Ectoedemia pilosae*** Puplesis, 1984a: 123 EP

***Ectoedemia picturata*** Puplesis, 1985c: 65 EP

***Ectoedemia minimella*** (Zetterstedt, 1839) van Nieukerken, 1985b: 80 ^67^ WP,EP,NEA

*Elachista minimella* Zetterstedt, 1839: 1011

*Nepticula woolhopiella* Stainton, 1887: 262 (syn: van Nieukerken, 1985b: 80)

*Nepticula canadensis* Braun, 1917: 185 **syn. n.** ^67^

*Nepticula viridicola* Weber, 1938: 211 (syn: van Nieukerken, 1985b: 80)

*Nepticula vividicola* Weber, 1938: 211 IOS

*Stigmella woolhopiella* (Stainton, 1887) Fletcher & Clutterbuck, 1945: 60

*Dechtiria woolhopiella* (Stainton, 1887) Beirne, 1945: 205

*Trifurcula woolhopiella* (Stainton, 1887) Johansson, 1971: 245

*Ectoedemia woolhopiella* (Stainton, 1887) Borkowski, 1975: 493

*Ectoedemia mediofasciella* auct. [misapplied] Bradley et al., 1972: 2

*Trifurcula mediofasciella* auct. [misapplied] Karsholt & Nielsen, 1976: 18

*Stigmella viridicola* (Weber, 1938) Klimesch, 1948b: 70

*Stigmella canadensis* (Braun, 1917) Davis & Wilkinson, 1983: 3

*Ectoedemia canadensis* (Braun, 1917) Wilkinson, 1981: 94

***Ectoedemia occultella*** (Linnaeus, 1767) Robinson & Nielsen, 1983: 221 ^68^ WP,EP,NEA

*Phalaena occultella* Linnaeus, 1767: 899

*Tinea strigilella* Thunberg, 1794: 87 (syn: Robinson & Nielsen, 1983: 221)

*Tinea mucidella* Hübner, 1817: pl. 65: Fig. 435 (syn: Zeller, 1839: 215)

*Tinea mediofasciella* Haworth, 1828: 584 (syn: van Nieukerken, 1985b: 78)

*Lyonetia argentipedella* Zeller, 1839: 215 (syn: Robinson & Nielsen, 1983: 221)

*Nepticula flexuosella* Fologne, 1859: 140 (syn: van Nieukerken & Johansson, 1987: 462)

*Nepticula lindquisti* Freeman, 1962: 899 (syn: van Nieukerken, 1985b: 80) ^68^

*Elachista mucidella* (Hübner, 1817) Treitschke, 1833: 179

*Lyonetia mucidella* (Hübner, 1817) Duponchel, 1844: 378

*Nepticula argentipedella* (Zeller, 1839) Heyden, 1843: 209

*Stigmella argentipedella* (Zeller, 1839) Fletcher & Clutterbuck, 1945: 60

*Dechtiria argentipedella* (Zeller, 1839) Beirne, 1945: 205

*Ectoedemia argentipedella* (Zeller, 1839) Bradley et al., 1972: 2

*Trifurcula argentipedella* (Zeller, 1839) Johansson, 1971: 245

*Ectoedemia lindquisti* (Freeman, 1962) Wilkinson & Scoble, 1979: 83

*Microsetia mediofasciella* (Haworth, 1828) Stephens, 1829: 208

***Ectoedemia angulifasciella*** (Stainton, 1849) Bradley et al., 1972: 2 (Fig. 38) WP

*Nepticula angulifasciella* Stainton, 1849: 29

*Nepticula schleichiella* Frey, 1870: 286 (syn: van Nieukerken, 1985b: 69)

*Nepticula brunniella* Sauber, 1904: 55 (syn: van Nieukerken, 1985b: 69)

*Nepticula utensis* Weber, 1937: 669 (syn: van Nieukerken, 1985b: 69)

*Nepticula minorella* Zimmermann, 1944: 118 (syn: van Nieukerken, 1985b: 69)

*Stigmella angulifasciella* (Stainton, 1849) Vári, 1944b: xxv

*Dechtiria angulifasciella* (Stainton, 1849) Beirne, 1945: 205

*Trifurcula angulifasciella* (Stainton, 1849) Johansson, 1971: 245

*Stigmella schleichiella* (Frey, 1870) Gerasimov, 1952: 259

*Stigmella utensis* (Weber, 1937) Klimesch, 1948b: 72

*Stigmella minorella* (Zimmermann, 1944) Klimesch, 1961: 739

*Ectoedemia rubivora* complex (van Nieukerken et al., 2012a: 7) (next 3 species)

***Ectoedemia arcuatella*** (Herrich-Schäffer, 1855) Bradley et al., 1972: 2 WP,EP

*Nepticula arcuatella* Herrich-Schäffer, 1855a: 354

*Nepticula arcuosella* Doubleday, 1859: 36 UE

*Stigmella arcuatella* (Herrich-Schäffer, 1855) Fletcher & Clutterbuck, 1945: 60

*Dechtiria arcuatella* (Herrich-Schäffer, 1855) Beirne, 1945: 206

*Trifurcula arcuatella* (Herrich-Schäffer, 1855) Johansson, 1971: 245

***Ectoedemia atricollis*** (Stainton, 1857) Bradley et al., 1972: 2 WP,EP

*Nepticula atricollis* Stainton, 1857a: 112

*Nepticula atricolella* Doubleday, 1859: 36 UE

*Nepticula aterrima* Wocke, 1865: 270 (syn: van Nieukerken, 1985b: 71)

*Nepticula staphyleae* Zimmermann, 1944: 117 (syn: van Nieukerken, 1985b: 71)

*Stigmella atricollis* (Stainton, 1857) Vári, 1944b: xxv

*Dechtiria atricollis* (Stainton, 1857) Vári, 1951: 197

*Trifurcula atricollis* (Stainton, 1857) Johansson, 1971: 245

*Stigmella aterrima* (Wocke, 1865) Gerasimov, 1952: 228

*Stigmella staphyleae* (Zimmermann, 1944) Hering, 1957: 1027

*Ectoedemia staphyleae* (Zimmermann, 1944) Borkowski, 1975: 493

‡ *Nepticula malivora* Toll, 1934b: 70 NNLM (syn: Skala, 1948: 121)

‡ Nepticula atricollis var. aterrimoides Skala, 1940: 143 NNLM (syn: Skala, 1948: 121)

‡ Nepticula atricollis var. prunivora Skala, 1941b: 77 NNLM

***Ectoedemia rubivora*** (Wocke, 1860) Bradley et al., 1972: 2 WP

*Nepticula rubivora* Wocke, 1860: 132

*Stigmella rubivora* (Wocke, 1860) Fletcher & Clutterbuck, 1945: 60

*Dechtiria rubivora* (Wocke, 1860) Beirne, 1945: 205

*Trifurcula rubivora* (Wocke, 1860) Johansson, 1971: 245

***Ectoedemia spinosella*** (Joannis, 1908) Bradley et al., 1972: 2 WP

*Nepticula spinosella* Joannis, 1908: 328

*Ectoedemia albiformae* Puplesis & Diškus, 2003a: 186 (syn: van Nieukerken et al., 2010: 70)

*Stigmella spinosella* (Joannis, 1908) Klimesch, 1951b: 62

*Dechtiria spinosella* (Joannis, 1908) Emmet, 1971b: 244

*Trifurcula spinosella* (Joannis, 1908) Johansson, 1971: 245

***Ectoedemia mahalebella*** (Klimesch, 1936) Szőcs, 1978: 266 WP

*Nepticula mahalebella* Klimesch, 1936: 207

*Stigmella mahalebella* (Klimesch, 1936) Lhomme, 1945: 155

***Ectoedemia erythrogenella*** (Joannis, 1908) Emmet, 1974a: 129 WP

*Nepticula erythrogenella* Joannis, 1908: 327

*Stigmella erythrogenella* (Joannis, 1908) Gerasimov, 1952: 238

*Trifurcula erythrogenella* (Joannis, 1908) Leraut, 1980: 49

‡ Stigmella erythrogenella ab. juncta Dufrane, 1949: 9

***Ectoedemia rubifoliella*** (Clemens, 1860) Wilkinson & Scoble, 1979: 90 NEA

*Nepticula rubifoliella* Clemens, 1860: 214

***Ectoedemia ulmella*** (Braun, 1912) Wilkinson & Scoble, 1979: 91 ^69^ NEA

*Nepticula ulmella* Braun, 1912: 87

*Ectoedemia andrella* Wilkinson, 1981: 102 **syn. n.** ^69^

***Ectoedemia ingloria*** Puplesis, 1988: 280 EP

***Ectoedemia insignata*** Puplesis, 1988: 281 EP

***Ectoedemia petrosa*** Puplesis, 1988: 282 EP

***Ectoedemia tadshikiella*** Puplesis, 1988: 25 WP,EP

***Ectoedemia* - unplaced species**

***Ectoedemia fuscivittata*** Puplesis & Robinson, 2000: 42 NEO

***Stigmellites*** Kernbach, 1967: 104 (TS/OD,M: *Stigmellites heringi* Kernbach, 1967)

*Ophiheliconoma* Krassilov, 2008: 100 (TS/OD,M: *Ophiheliconoma resupinata* Krassilov, 2008) (syn: Doorenweerd et al., 2015a: 309)

***Stigmellites almeidae*** (Martins-Neto, 1989) Doorenweerd et al., 2015a: 315 NEO†

*Nepticula almeidae* Martins-Neto, 1989: 381

*Stigmella almeidae* (Martins-Neto, 1989) Sohn et al., 2012: 22

***Stigmellites baltica*** Kozlov, 1988: 30 WP†

***Stigmellites carpiniorientalis*** Straus, 1977: 60 WP†

***Stigmellites centennis*** Jarzembowski, 1989: 448 WP†

***Stigmellites fossilis*** (Heyden, 1862) Kozlov, 1988: 31 WP†

*Nepticula fossilis* Heyden, 1862: 77

***Stigmellites gossi*** Jarzembowski, 1989: 448 WP†

***Stigmellites heringi*** Kernbach, 1967: 104 WP†

***Stigmellites kzyldzharica*** Kozlov, 1988: 32 EP†

***Stigmellites messelensis*** Straus, 1976: 445 WP†

***Stigmellites pliotityrella*** Kernbach, 1967: 105 WP†

***Stigmellites resupinata*** (Krassilov, 2008) Doorenweerd et al., 2015a: 309 WP†

*Ophiheliconoma resupinata* Krassilov, 2008: 100

***Stigmellites samsonovi*** Kozlov, 1988: 33 EP†

***Stigmellites serpentina*** Kozlov, 1988: 32 EP†

***Stigmellites sharovi*** Kozlov, 1988: 33 EP†

***Stigmellites tyshchenkoi*** Kozlov, 1988: 33 EP†

***Stigmellites zelkovae*** Straus, 1977: 61 WP†

**Nomina dubia et oblita**

***Nepticula alpinella*** Herrich-Schäffer, 1863b: 170 ^70^ NO WP

***Nepticula alticolella*** Herrich-Schäffer, 1863c: 182 ^70^ NO WP

***Nepticula reuttiella*** Herrich-Schäffer, 1863c: 182 ^70^ NO WP

***Nepticula oritis*** Meyrick, 1910: 229 ^71^ ND OR

***Nepticula xuthomitra*** Meyrick, 1921b: 140 ^72^ ND AFR

***Nepticula anguinella*** Clemens, 1861: 85 ^73^ ND NEA

*Ectoedemia anguinella* (Clemens, 1861) Wilkinson, 1981: 98 ND

***Nepticula platea*** Clemens, 1861: 85 ^73^ ND NEA

*Ectoedemia platea* (Clemens, 1861) Wilkinson, 1981: 98 ND

**unplaced unavailable names** ^74^

‡ *Nepticula brunensis* Skala, 1939g: 144 NNLM WP

‡ *Nepticula buhri* Skala, 1938: 43 NNLM WP

‡ *Nepticula sorbifoliella* Skala, 1939g: 144 NNLM WP

‡ *Nepticula tentationis* Hoffmann, 1893: 215 NN WP

‡ *Nepticula ulmi* Skala, 1934a: 51 NNLM WP

‡ *Stigmella acernella* Dovnar-Zapolski & Tomilova, 1978: 27 NNLM EP

‡ *Stigmella amygdaliella* Dovnar-Zapolski, 1969: 23 NNLM EP

‡ *Stigmella apocynella* Gerasimov, 1937: 284 NNLM EP

‡ *Stigmella atraphaxidella* Dovnar-Zapolski, 1969: 29 NNLM EP

‡ *Stigmella betulivora* Dovnar-Zapolski, 1969: 32 NNLM EP

‡ *Stigmella crataegifolia* Dovnar-Zapolski, 1969: 49 NNLM EP

‡ *Stigmella loniceraefolia* Dovnar-Zapolski, 1969: 67 NNLM EP

‡ *Stigmella loniceraevora* Dovnar-Zapolski, 1969: 67 NNLM EP

‡ *Stigmella prunivora* Dovnar-Zapolski, 1969: 90 NNLM WP

‡ *Stigmella pseudoanomalella* Dovnar-Zapolski, 1969: 94 NNLM EP

‡ *Stigmella roseifolia* Dovnar-Zapolski, 1969: 94 NNLM EP

‡ *Stigmella roseivora* Dovnar-Zapolski, 1969: 94 NNLM EP

‡ *Stigmella rosella* Dovnar-Zapolski, 1969: 95 NNLM EP

FAMILY **OPOSTEGIDAE** Meyrick, 1893: 479 (TG: *Opostega* Zeller, 1839)

Family Opostegides Meyrick, 1893: 479 (TG: *Opostega* Zeller, 1839)

Subfamily Opostegoidinae Kozlov, 1987: 856 (TG: *Opostegoides* Kozlov, 1985) **syn. n.**

Subfamily Oposteginae Meyrick, 1893 (TG: *Opostega* Zeller, 1839)

***Notiopostega*** Davis, 1989: 30 (TS/OD,M: *Notiopostega atrata* Davis, 1989)

***Notiopostega atrata*** Davis, 1989: 32 NEO

***Eosopostega*** Davis, 1989: 41 (TS/OD,M: *Eosopostega issikii* Davis, 1989)

***Eosopostega issikii*** Davis, 1989: 42 EP

***Eosopostega armigera*** Puplesis & Robinson, 1999: 29 OR

***Neopostega*** Davis & Stonis, 2007: 34 (TS/OD: *Neopostega petila* Davis & Stonis, 2007: 38)

***Neopostega asymmetra*** Davis & Stonis, 2007: 37 NEO

***Neopostega distola*** Davis & Stonis, 2007: 39 NEO

***Neopostega falcata*** Davis & Stonis, 2007: 36 NEO

***Neopostega longispina*** Davis & Stonis, 2007: 36 NEO

***Neopostega nigrita*** Heppner & Davis, 2009: 31 NEO

***Neopostega petila*** Davis & Stonis, 2007: 38 NEO

***Paralopostega*** Davis, 1989: 52 (TS/OD: *Opostega callosa* Swezey, 1921)

***Paralopostega callosa*** (Swezey, 1921) Davis, 1989: 72 AUS

*Opostega callosa* Swezey, 1921: 532

***Paralopostega dives*** (Walsingham, 1907) Davis, 1989: 72 AUS

*Opostega dives* Walsingham, 1907: 711

***Paralopostega filiforma*** (Swezey, 1921) Davis, 1989: 72 AUS

*Opostega filiforma* Swezey, 1921: 534

***Paralopostega maculata*** (Walsingham, 1907) Davis, 1989: 72 AUS

*Opostega maculata* Walsingham, 1907: 711

***Paralopostega peleana*** (Swezey, 1921) Davis, 1989: 73 AUS

*Opostega peleana* Swezey, 1921: 534

***Paralopostega serpentina*** (Swezey, 1921) Davis, 1989: 73 AUS

*Opostega serpentina* Swezey, 1921: 533

***Opostegoides*** Kozlov, 1985: 54 (TS/OD: *Opostega minodensis* Kuroko, 1982) ^75^

***Opostegoides menthinella*** (Mann, 1855) Davis, 1989: 72 WP

*Opostega menthinella* Mann, 1855: 568

*Opostega snelleni* Nolcken, 1882: 197 (syn: van Nieukerken, 1996: 300)

***Opostegoides albella*** Sinev, 1990: 102 EP

***Opostegoides bicolorella*** Sinev, 1990: 105 EP

***Opostegoides minodensis*** (Kuroko, 1982) Kozlov, 1985: 54 EP

*Opostega minodensis* Kuroko, 1982: 50, 448

***Opostegoides omelkoi*** Kozlov, 1985: 57 EP

***Opostegoides padiensis*** Sinev, 1990: 105 EP

***Opostegoides sinevi*** Kozlov, 1985: 55 EP

***Opostegoides argentisoma*** Puplesis & Robinson, 1999: 22 OR

***Opostegoides auriptera*** Puplesis & Robinson, 1999: 28 OR

***Opostegoides cameroni*** Puplesis & Robinson, 1999: 27 OR

***Opostegoides epistolaris*** (Meyrick, 1911b) Puplesis & Robinson, 1999: 20 OR

*Opostega epistolaris* Meyrick, 1911b: 108

***Opostegoides flavimacula*** Puplesis & Robinson, 1999: 27 OR

***Opostegoides gorgonea*** Puplesis & Robinson, 1999: 22 OR

***Opostegoides index*** (Meyrick, 1922) Puplesis & Robinson, 1999: 20 OR

*Opostega index* Meyrick, 1922: 557

***Opostegoides longipedicella*** Puplesis & Robinson, 1999: 26 OR

***Opostegoides malaysiensis*** Davis, 1989: 52 OR

***Opostegoides nephelozona*** (Meyrick, 1915) Puplesis & Robinson, 1999: 19 OR

*Opostega nephelozona* Meyrick, 1915b: 352

***Opostegoides pelorrhoa*** (Meyrick, 1915) Puplesis & Robinson, 1999: 18 OR

*Opostega pelorrhoa* Meyrick, 1915b: 352

***Opostegoides spinifera*** Puplesis & Robinson, 1999: 26 OR

***Opostegoides tetroa*** (Meyrick, 1907) Puplesis & Robinson, 1999: 18 OR

*Opostega tetroa* Meyrick, 1907: 986

***Opostegoides thailandica*** Puplesis & Robinson, 1999: 23 OR

***Opostegoides uvida*** (Meyrick, 1915) Puplesis & Robinson, 1999: 19 OR

*Opostega uvida* Meyrick, 1915b: 352

***Opostegoides granifera*** (Meyrick, 1913) **comb. n.** ^75^ AFR

*Opostega granifera* Meyrick, 1913: 327

***Opostegoides melitardis*** (Meyrick, 1918) **comb. n.** ^75^ AFR

*Opostega melitardis* Meyrick, 1918a: 41

***Opostegoides pelocrossa*** (Meyrick, 1928) **comb. n.** ^75^ AFR

*Opostega pelocrossa* Meyrick, 1928a: 396

***Opostegoides praefusca*** (Meyrick, 1913) **comb. n.** ^75^ AFR

*Opostega praefusca* Meyrick, 1913: 327

***Opostegoides gephyraea*** (Meyrick, 1880) Davis, 1989: 72 AUS

*Opostega gephyraea* Meyrick, 1880: 176

***Opostegoides scioterma*** (Meyrick, 1920) Kozlov, 1985: 55 NEA

*Opostega scioterma* Meyrick, 1920c: 358

***Opostega*** Zeller, 1839: 214 (TS/SD (Walsingham, 1914: 349): *Elachista salaciella* Treitschke, 1833)

***Opostega cretatella*** Chrétien, 1915: 364 ^76^ WP,EP

*Opostega rezniki* Kozlov, 1985: 51 **syn. n.** ^76^

***Opostega kuznetzovi*** Kozlov, 1985: 53 WP,EP

***Opostega salaciella*** (Treitschke, 1833) Zeller, 1939: 214 WP

*Elachista salaciella* Treitschke, 1833: 180

*Opostega reliquella* Zeller, 1848: 282

***Opostega spatulella*** Herrich-Schäffer, 1855a: 360 (Fig. 39) WP,EP

*Opostega nepticulella* Bruand, 1859: 691 (syn: Leraut, 1997: 80)

*Opostega bimaculatella* N.R. Rothschild, 1912: 29 (syn: van Nieukerken, 1990a: 368)

*Opostega costantiniella* Costantini in Turati, 1923: 70 (syn: van Nieukerken, 1990a: 368)

*Opostega angulata* Gerasimov, 1930: 45 (syn: Puplesis et al., 1996: 192)

*Opostega heringella* Mariani, 1937: 12 (syn: van Nieukerken, 1990a: 368)

***Opostega stekolnikovi*** Kozlov, 1985: 53 WP

***Opostega afghani*** Davis, 1989: 62 EP

***Opostega chalcophylla*** Meyrick, 1910: 229 OR

“*Opostega*” (unplaced African species) ^77^

**“*Opostega” cirrhacma*** Meyrick, 1911a: 237 AFR

**“*Opostega” diplardis*** Meyrick, 1921b: 123 AFR

**“*Opostega” radiosa*** Meyrick, 1913: 327 AFR

“*Opostega*” (unplaced Australian species) ^77^

**“*Opostega” arthrota*** Meyrick, 1915b: 352 AUS

**“*Opostega” atypa*** Turner, 1923: 179 AUS

**“*Opostega” basilissa*** Meyrick, 1893: 606 AUS

**“*Opostega” brithys*** Turner, 1923: 179 AUS

**“*Opostega” chalcoplethes*** Turner, 1923: 178 AUS

**“*Opostega” chalinias*** Meyrick, 1893: 607 AUS

**“*Opostega” chordacta*** Meyrick, 1915b: 351 AUS

**“*Opostega” diorthota*** Meyrick, 1893: 607 AUS

**“*Opostega” horaria*** Meyrick, 1921d: 457 AUS

**“*Opostega” luticilia*** Meyrick, 1915b: 351 AUS

**“*Opostega” monotypa*** Turner, 1923: 179 AUS

**“*Opostega” nubifera*** Turner, 1900: 23 AUS

**“*Opostega” orestias*** Meyrick, 1880: 175 AUS

**“*Opostega” phaeospila*** Turner, 1923: 179 AUS

**“*Opostega” scoliozona*** Meyrick, 1915b: 351 AUS

**“*Opostega” stiriella*** Meyrick, 1880: 175 AUS

**“*Opostega” xenodoxa*** Meyrick, 1893: 608 AUS

***Pseudopostega*** Kozlov, 1985: 53 (TS/OD: *Tinea auritella* Hübner, 1813)

Palearctic species

***Pseudopostega auritella*** (Hübner, 1813) Davis, 1989: 76 WP,EP

*Tinea auritella* Hübner, 1813: Pl. 57: Fig. 387

*Leucoptera auritella* (Hübner, 1813) Hübner, 1825: 426

*Opostega auritella* (Hübner, 1813) Zeller, 1939: 214

***Pseudopostega chalcopepla*** (Walsingham, 1908) van Nieukerken, 1996: 27 ^78^ WP

*Opostega chalcopepla* Walsingham, 1908b: 228

*Pseudopostega cyrneochalcopepla* Nel & Varenne, 2012: 11 **syn. n.** ^78^

‡ *Opostega rosmarinella* Staudinger, 1894 (syn: Walsingham, 1908b: 228) NN

***Pseudopostega crepusculella*** (Zeller, 1839) Davis, 1989: 76 (Fig. 40) WP,EP

*Opostega crepusculella* Zeller, 1839: 214

*Oecophora crepusculella* Duponchel, 1843: 337 JSH

*Opostega crepusculella lvovskyi* Kozlov, 1985: 54

Oriental species

***Pseudopostega alleni*** Puplesis & Robinson, 1999: 40 OR

***Pseudopostega amphivittata*** Puplesis & Robinson, 1999: 39 OR

***Pseudopostega brevicaudata*** Remeikis & Stonis in Stonis et al., 2013a: 183 OR

***Pseudopostega epactaea*** (Meyrick, 1907) Puplesis & Robinson, 1999: 32 OR

*Opostega epactaea* Meyrick, 1907: 985

***Pseudopostega euryntis*** (Meyrick, 1907) Puplesis & Robinson, 1999: 44 OR

*Opostega euryntis* Meyrick, 1907: 985

***Pseudopostega frigida*** (Meyrick, 1906) Puplesis & Robinson, 1999: 32 OR

*Opostega frigida* Meyrick, 1906a: 416

***Pseudopostega fungina*** Puplesis & Robinson, 1999: 42 OR

***Pseudopostega indonesica*** Puplesis & Robinson, 1999: 41 OR

***Pseudopostega javae*** Puplesis & Robinson, 1999: 39 OR

***Pseudopostega machaerias*** (Meyrick, 1907) Puplesis & Robinson, 1999: 30 OR

*Opostega machaerias* Meyrick, 1907: 986

***Pseudopostega myxodes*** (Meyrick, 1916) Puplesis & Robinson, 1999: 34 OR

*Opostega myxodes* Meyrick, 1916a: 619

***Pseudopostega nepalensis*** Puplesis & Robinson, 1999: 37 OR

***Pseudopostega nigrimaculella*** Puplesis & Robinson, 1999: 40 OR

***Pseudopostega parvilineata*** Puplesis & Robinson, 1999: 31 OR

***Pseudopostega saturella*** Puplesis & Robinson, 1999: 38 OR

***Pseudopostega similantis*** Puplesis & Robinson, 1999: 33 OR

***Pseudopostega spilodes*** (Meyrick, 1915b) Puplesis & Robinson, 1999: 45 OR

*Opostega spilodes* Meyrick, 1915b: 351

***Pseudopostega strigulata*** Puplesis & Robinson, 1999: 45 OR

***Pseudopostega subviolacea*** (Meyrick, 1920) Puplesis & Robinson, 1999: 45 OR

*Opostega subviolacea* Meyrick, 1920c: 357

***Pseudopostega sumbae*** Puplesis & Robinson, 1999: 37 OR

***Pseudopostega velifera*** (Meyrick, 1920) Puplesis & Robinson, 1999: 34 OR

*Opostega velifera* Meyrick, 1920c: 357

***Pseudopostega zelopa*** (Meyrick, 1905) Puplesis & Robinson, 1999: 43 OR

*Opostega zelopa* Meyrick, 1905: 613

African species **^79^**

***Pseudopostega amphimitra*** (Meyrick, 1913) **comb. n.** ^79^ AFR

*Opostega amphimitra* Meyrick, 1913: 328

***Pseudopostega bellicosa*** (Meyrick, 1911a) Davis, 1989: 76 AFR

*Opostega bellicosa* Meyrick, 1911a: 236

***Pseudopostega clastozona*** (Meyrick, 1913) Davis, 1989: 76 AFR

*Opostega clastozona* Meyrick, 1913: 327

***Pseudopostega idiocoma*** (Meyrick, 1918) **comb. n.** ^79^ AFR

*Opostega idiocoma* Meyrick, 1918a: 42

***Pseudopostega orophoxantha*** (Meyrick, 1921) **comb. n.** ^79^ AFR

*Opostega orophoxantha* Meyrick, 1921b: 124

***Pseudopostega phaeosoma*** (Meyrick, 1928) **comb. n.** ^79^ AFR

*Opostega phaeosoma* (Meyrick, 1928a): 396

***Pseudopostega symbolica*** (Meyrick, 1914) **comb. n.** ^79^ AFR

*Opostega symbolica* Meyrick, 1914: 203

***Pseudopostega tincta*** (Meyrick, 1918) **comb. n.** ^79^ AFR

*Opostega tincta* Meyrick, 1918a: 41

Nearctic species

***Pseudopostega acidata*** (Meyrick, 1915) Davis, 1989: 75 NEA,NEO

*Opostega acidata* Meyrick, 1915a: 240

***Pseudopostega albogaleriella*** (Clemens, 1862) Davis, 1989: 76 NEA

*Opostega albogaleriella* Clemens, 1862: 131

*Opostega napaeella* Clemens, 1872: 42 (syn: Davis, 1983: 3)

*Opostega bistrigulella* Braun, 1918: 245 (syn: Davis & Stonis, 2007: 71)

*Opostega nonstrigella* Chambers, 1881: 296 (syn: Forbes, 1923: 161)

*Pseudopostega napaeella* (Clemens, 1872) Davis, 1989: 76

*Pseudopostega bistrigulella* (Braun, 1918) Davis, 1989: 76

*Pseudopostega nonstrigella* (Chambers, 1881) Davis, 1989: 76

***Pseudopostega cretea*** (Meyrick, 1920) Davis, 1989: 76 NEA

*Opostega cretea* Meyrick, 1920c: 358

***Pseudopostega floridensis*** Davis & Stonis, 2007: 57 NEA

***Pseudopostega kempella*** (Eyer, 1967) Davis, 1989: 76 NEA,NEO

*Opostega kempella* Eyer, 1967: 39

***Pseudopostega parakempella*** Davis & Stonis, 2007: 100 NEA,NEO

***Pseudopostega quadristrigella*** (Chambers, 1875) Davis, 1989: 77 NEA

*Opostega quadristrigella* Chambers, 1875b: 106

*Opostega accessoriella* Frey & Boll, 1876: 216 (syn: McDunnough, 1939: 100)

*Pseudopostega accessoriella* (Frey & Boll, 1876) Davis, 1989: 75

***Pseudopostega texana*** Davis & Stonis, 2007: 115 NEA

***Pseudopostega venticola*** (Walsingham, 1897) Davis, 1989: 77 NEA,NEO

*Opostega venticola* Walsingham, 1897: 140

Neotropic species

***Pseudopostega abrupta*** (Walsingham, 1897) Davis, 1989: 75 NEO

*Opostega abrupta* Walsingham, 1897: 139

***Pseudopostega acrodicra*** Davis & Stonis, 2007: 122 NEO

***Pseudopostega acuminata*** Davis & Stonis, 2007: 89 NEO

***Pseudopostega adusta*** (Walsingham, 1897) Davis, 1989: 76 NEO

*Opostega adusta* Walsingham, 1897: 140

***Pseudopostega apoclina*** Davis & Stonis, 2007: 131 NEO

*Pseudopostega latifurcata apoclina* Davis & Stonis, 2007: 131

***Pseudopostega apotoma*** Davis & Stonis, 2007: 65 NEO

***Pseudopostega attenuata*** Davis & Stonis, 2007: 76 NEO

***Pseudopostega beckeri*** Davis & Stonis, 2007: 136 NEO

***Pseudopostega bicornuta*** Davis & Stonis, 2007: 138 NEO

***Pseudopostega bidorsalis*** Davis & Stonis, 2007: 127 NEO

***Pseudopostega brachybasis*** Davis & Stonis, 2007: 142 NEO

***Pseudopostega breviapicula*** Davis & Stonis, 2007: 85 NEO

***Pseudopostega brevifurcata*** Davis & Stonis, 2007: 120 NEO

***Pseudopostega brevivalva*** Davis & Stonis, 2007: 121 NEO

***Pseudopostega caulifurcata*** Davis & Stonis, 2007: 123 NEO

***Pseudopostega clavata*** Davis & Stonis, 2007: 105 NEO

***Pseudopostega colognatha*** Davis & Stonis, 2007: 90 NEO

***Pseudopostega concava*** Davis & Stonis, 2007: 119 NEO

***Pseudopostega congruens*** (Walsingham, 1914) Davis, 1989: 76 NEO

*Opostega congruens* Walsingham, 1914: 350

***Pseudopostega conicula*** Davis & Stonis, 2007: 78 NEO

***Pseudopostega constricta*** Davis & Stonis, 2007: 141 NEO

***Pseudopostega contigua*** Davis & Stonis, 2007: 129 NEO

***Pseudopostega crassifurcata*** Davis & Stonis, 2007: 117 NEO

***Pseudopostega curtarama*** Davis & Stonis, 2007: 116 NEO

***Pseudopostega denticulata*** Davis & Stonis, 2007: 74 NEO

***Pseudopostega didyma*** Davis & Stonis, 2007: 109 NEO

***Pseudopostega diskusi*** Davis & Stonis, 2007: 67 NEO

***Pseudopostega divaricata*** Davis & Stonis, 2007: 128 NEO

***Pseudopostega dorsalis*** Davis & Stonis, 2007: 98 NEO

*Pseudopostega dorsalis dorsalis* Davis & Stonis, 2007: 98 NEO

***Pseudopostega duplicata*** Davis & Stonis, 2007: 108 NEO

***Pseudopostega ecuadoriana*** Davis & Stonis, 2007: 134 NEO

***Pseudopostega elachista*** (Walsingham, 1914) Davis, 1989: 76 NEO

*Opostega elachista* Walsingham, 1914: 350

***Pseudopostega fasciata*** Davis & Stonis, 2007: 99 NEO

*Pseudopostega dorsalis fasciata* Davis & Stonis, 2007: 99

***Pseudopostega ferruginea*** Davis & Stonis, 2007: 54 NEO

***Pseudopostega fumida*** Davis & Stonis, 2007: 62 NEO

***Pseudopostega galapagosae*** Davis & Stonis, 2007: 93 NEO

***Pseudopostega gracilis*** Davis & Stonis, 2007: 63 NEO

***Pseudopostega lateriplicata*** Davis & Stonis, 2007: 59 NEO

***Pseudopostega latiapicula*** Davis & Stonis, 2007: 133 NEO

***Pseudopostega latifurcata*** Davis & Stonis, 2007: 130 NEO

*Pseudopostega latifurcata latifurcata* Davis & Stonis, 2007: 130 NEO

***Pseudopostega latiplana*** Remeikis & Stonis in Remeikis et al., 2009: 283 NEO

***Pseudopostega latisaccula*** Davis & Stonis, 2007: 75 NEO

***Pseudopostega lobata*** Davis & Stonis, 2007: 104 NEO

***Pseudopostega longifurcata*** Davis & Stonis, 2007: 141 NEO

***Pseudopostega longipedicella*** Davis & Stonis, 2007: 102 NEO

***Pseudopostega mexicana*** Remeikis & Stonis in Remeikis et al., 2009: 282 NEO

***Pseudopostega microacris*** Davis & Stonis, 2007: 61 NEO

***Pseudopostega microlepta*** (Meyrick, 1915) Davis, 1989: 76 NEO

*Opostega microlepta* Meyrick, 1915a: 239

***Pseudopostega mignonae*** Davis & Stonis, 2007: 86 NEO

***Pseudopostega monosperma*** (Meyrick, 1931) Davis, 1989: 76 NEO

*Opostega monosperma* Meyrick, 1931b: 162

***Pseudopostega monstruosa*** Davis & Stonis, 2007: 68 NEO

***Pseudopostega obtusa*** Davis & Stonis, 2007: 91 NEO

***Pseudopostega ovatula*** Davis & Stonis, 2007: 52 NEO

***Pseudopostega paraplicatella*** Davis & Stonis, 2007: 82 NEO

***Pseudopostega paromias*** (Meyrick, 1915a) Davis, 1989: 77 NEO

*Opostega paromias* Meyrick, 1915a: 240

***Pseudopostega perdigna*** (Walsingham, 1914) Davis, 1989: 77 NEO

*Opostega perdigna* Walsingham, 1914: 349

***Pseudopostega pexa*** (Meyrick, 1920) Davis, 1989: 77 NEO

*Opostega pexa* Meyrick, 1920c: 358

***Pseudopostega plicatella*** Davis & Stonis, 2007: 82 NEO

***Pseudopostega pontifex*** (Meyrick, 1915) Davis, 1989: 77 NEO

*Opostega pontifex* Meyrick, 1915a: 240

***Pseudopostega protomochla*** (Meyrick, 1935) Davis, 1989: 77 NEO

*Opostega protomochla* Meyrick, 1935: 567

***Pseudopostega pumila*** (Walsingham, 1914) Davis, 1989: 77 NEO

*Opostega pumila* Walsingham, 1914: 350

***Pseudopostega resimafurcata*** Davis & Stonis, 2007: 124 NEO

***Pseudopostega robusta*** Remeikis & Stonis in Remeikis et al., 2009: 281 NEO

***Pseudopostega rotunda*** Davis & Stonis, 2007: 51 NEO

***Pseudopostega sacculata*** (Meyrick, 1915) Davis, 1989: 77 NEO

*Opostega sacculata* Meyrick, 1915a: 240

***Pseudopostega saltatrix*** (Walsingham, 1897) Davis, 1989: 77 NEO

*Opostega saltatrix* Walsingham, 1897: 140

***Pseudopostega sectila*** Davis & Stonis, 2007: 113 NEO

***Pseudopostega serrata*** Davis & Stonis, 2007: 52 NEO

***Pseudopostega spatulata*** Davis & Stonis, 2007: 70 NEO

***Pseudopostega sublobata*** Davis & Stonis, 2007: 107 NEO

***Pseudopostega subtila*** Davis & Stonis, 2007: 88 NEO

***Pseudopostega suffuscula*** Davis & Stonis, 2007: 139 NEO

***Pseudopostega tanygnatha*** Davis & Stonis, 2007: 90 NEO

***Pseudopostega tenuifurcata*** Davis & Stonis, 2007: 112 NEO

***Pseudopostega triangularis*** Davis & Stonis, 2007: 79 NEO

***Pseudopostega trinidadensis*** (Busck, 1910) Davis, 1989: 77 NEO

*Opostega trinidadensis* Busck, 1910: 245

***Pseudopostega truncata*** Davis & Stonis, 2007: 67 NEO

***Pseudopostega tucumanae*** Davis & Stonis, 2007: 64 NEO

***Pseudopostega turquinoensis*** Davis & Stonis, 2007: 119 NEO

***Pseudopostega uncinata*** Davis & Stonis, 2007: 60 NEO

**Taxa excluded from Nepticuloidea**

See further van Nieukerken & Johansson (1987), Davis (1989), Puplesis & Robinson (1999) and for the fossils Doorenweerd et al. (2015b).

FAMILY **ARGYRESTHIIDAE**

***Argyresthia abdominalis*** Zeller, 1839: 205 WP

*Nepticula abdominalella* (Duponchel, [1845]) Bruand, 1859: 686

FAMILY **BUCCULATRICIDAE**

***Bucculatrix cristatella*** (Zeller, 1839) Zeller, 1848: 300 WP

*Lyonetia concolorella* Tengström, 1848 (syn: Rebel, 1901: 220)

*Nepticula concolorella* (Tengström, 1848) Heydenreich, 1851: 92

***Bucculatrix frangutella*** (Goeze, 1783) WP

*Elachista rhamnifoliella* Treitschke, 1833: 183

*Opostega rhamnifoliella* (Treitschke, 1833) Bruand, [1851]: 86

***Bucculatrix centrospila*** (Turner, 1923) Davis, 1989: 2 AUS

*Opostega centrospila* Turner, 1923: 179

FAMILY **COSMOPTERIGIDAE**

***Stagmatophora heydeniella*** (Fischer von Röslerstamm, 1841) WP

*Oecophora heydeniella* Fischer von Röslerstamm, 1841: 256

‡ *Opostega torquillaepennella* Bruand, [1851]: 86 NN (syn: Bruand, [1851]: 86)

FAMILY **GELECHIIDAE**

***Nepticula belfrageella*** Chambers, 1875a: 75 ^80^ NO NEA

*Stigmella belfrageella* (Chambers, 1875) Newton & Wilkinson, 1982: 456 NO

FAMILY **GRACILLARIIDAE**

***Metriochroa latifoliella*** (Millière, 1886) Vári, 1961: 196 WP

*Nepticula latifoliella* Millière, 1886: 220

***Phyllocnistis saligna*** (Zeller, 1839) WP

*Tinea cerasifoliella* Hübner, 1796: pl 28: 190 NO (syn: Stainton, 1848: 2158)

*Opostega saligna* Zeller, 1839: 214

*Opostega salicifoliella* Duponchel, 1844: 377

*Opostega salignatella* Bruand, [1851]: 86 UE

*Opostega lugdunensella* Bruand, 1859: 691

*Opostega cerasifoliella* (Hübner, 1796) Bruand, 1859: 691

***Phyllocnistis unipunctella*** (Stephens, 1834) WP

*Argyromyges unipunctella* Stephens, 1834: 260

*Opostega suffusella* Zeller, 1847: 894 [type species of *Phyllocnistis*]

‡ *Opostega tremulella* Fischer von Röslerstamm in Zeller, 1843: 21 NN (syn: Zeller, 1848: 266)

*Opostega tremulella* Heeger, 1852: 278

***Phyllocnistis argentella*** (Bradley, 1957) Puplesis & Robinson, 1999: 18 AUS

*Opostega argentella* Bradley, 1957: 108

***Phyllonorycter populifoliella*** (Treitschke, 1833) WP

*Elachista populifoliella* Treitschke, 1833: 188

*Nepticula pilosissimella* Bruand, 1859 : 686

FAMILY **HELIOZELIDAE**

***Heliozela sericiella*** (Haworth, 1828) WP

*Tinea sericiella* Haworth, 1828: 585

*Aechmia saltatricella* Fischer von Röslerstamm, 1841: 249

*Nepticula saltatricella* (Fischer von Röslerstamm, 1841) Bruand, 1859: 687

***Heliozela lithargyrellum*** (Zeller, 1850) WP

*Tinagma lithargyrellum* Zeller, 1850: 158

*Nepticula lithargyrella* (Zeller, 1850) Bruand, 1859: 687

FAMILY **LYONETIIDAE**

***Leucoptera malifoliella*** (O. Costa, 1836) WP

*Elachista malifoliella* O. Costa, 1836: [239] Elachista 3

*Opostega scitella* Zeller, 1839: 214 (syn: Stainton, 1869a: 269)

***Leucoptera sinuella*** (Reutti, 1853) WP

*Cemiostoma sinuella* Reutti, 1853: 208

*Cerniostoma susinella* Herrich-Schäffer, 1855: 342

*Opostega susinella* (Herrich-Schäffer, 1855) Bruand, 1859: 691

***Leucoptera spartifoliella*** (Hübner, 1813) Hübner, 1825: 426 WP

*Tinea spartifoliella* Hübner, 1813: 49

*Opostega spartifoliella* (Hübner, 1813) Zeller, 1839: 214

***Leucoptera phaeopasta*** (Turner, 1923) Davis, 1989: 2 AUS

*Opostega phaeopasta* Turner, 1923: 180

***Lyonetia clerkella*** (Linnaeus, 1758) WP,EP

*Phalaena clerkella* Linnaeus, 1758: 542

*Opostega magnimaculella* Bruand, 1859: 691 (syn: Leraut, 1997: 101)

***Lyonetia leucoprepes*** (Bradley, 1961) Davis, 1989: 2 AUS

*Opostega leucoprepes* Bradley, 1961: 160

***Petasobathra ischnophaea*** (Meyrick, 1930) Davis, 1989: 2 OR

*Opostega ischnophaea* Meyrick, 1930: 7

FAMILY **TISCHERIIDAE**

***Coptotriche angusticollella*** (Duponchel, 1843) Diškus & Puplesis, 2003: 430 WP,EP

*Nepticula suberoidella* Walsingham, 1891: 152 (syn: Diškus & Puplesis, 2003: 430)

*Stigmella suberoidella* (Walsingham, 1891) Le Marchand, 1946b: 284

(see van Nieukerken, 2004: 112)

FAMILY **UNKNOWN**

***Tinea minimella*** O.G. Costa, 1836: [230] Tinea 18, JH of *Tinea minimella* [Denis & Schiffermüller], 1775, now *Nemophora minimella* (Adelidae) WP

*Nepticula minimella* (O.G. Costa, 1836) Stainton, 1869a: 267

FAMILY **UNKNOWN, may be Trichoptera, Hydroptilidae**

***Tinea commatella*** Schrank, 1802: 133 ND WP

*Nepticula commatella* (Schrank, 1802) Stainton et al., 1855: 264

**UNPLACED FOSSILS**

***Tinea araliae*** Fritsch, 1882: 6 [may be Gracillariidae] WP†

*Stigmellites araliae* (Fritsch, 1882) Kozlov, 1988: 30

***Foliofossor cranei*** Jarzembowski, 1989: 448 WP†

***Troponoma curvitracta*** Krassilov, 2008: 101 WP†

***Troponoma festunata*** Krassilov, 2008: 102 WP†

### Notes

**1** The genus *Manoneura* Davis, 1979 had been synonymised with *Enteucha* Meyrick, 1915 by van Nieukerken (1986a), but resurrected as separate genus by Puplesis and Robinson (2000) on the basis of its aberrant genitalia. In two independent molecular analyses, the type species *Manoneura basidactyla* (Davis, 1978) clearly groups inside the genus *Enteucha* (Regier et al. 2015, Doorenweerd et al. 2016a) and we thus regard it here again as synonym.

**2** The paper by Walsingham on the Tenerife fauna (Walsingham 1908a), where also the validity of the name *Stigmella* was established, has often been cited as Walsingham, 1907. This paper is published in the last issue of the Entomologist’s Monthly Magazine for 1907, but issued on June, 4^th^, 1908 as can be seen on the wrappers of volume 1908 (1), page iv (http://biodiversitylibrary.org/page/31209657) (see also Sattler 1973), thus the citations should be Walsingham 1908.

**3** The genus *Stigmella* is divided into two large clades (Doorenweerd et al. 2016a) that are termed respectively “core *Stigmella*” for the clade containing the type species of both *Stigmella* and *Nepticula* (*Stigmella anomalella* and *Stigmella aurella*) and non-core *Stigmella* for the other clade (containing the type species of *Astigmella*: *Stigmella naturnella*). We refrain from recognising different genera, since recognising these clades morphologically is not always possible. Several species groups could not be placed due to lack of molecular information, they are listed at the end of *Stigmella*.

**4** *Stigmella resplendensella* (Chambers, 1875) was placed as species with uncertain affinities due to the lacking abdomen in the lectotype (Newton and Wilkinson 1982). We re-examined the lectotype and could match the characteristic metallic forewing colour pattern to two males and one female we had on loan, of which the female had been barcoded (see also BugGuide: http://bugguide.net/node/view/391014/bgpage). Barcode and genitalia confirm that *Stigmella resplendensella* is closely related to *Stigmella unifasciella* (Chambers, 1875), as earlier suggested by Braun (1917). Further details to be published elsewhere. Material: Lectotype female, MCZ-ENT00014954 (designated by Newton & Wilkinson, 1982: 456, [USA: Kentucky, Covington], captured May 23rd, under hackberry trees (Celtis occidentalis), V.T. Chambers, Type14954 [head and abdomen missing].

**5** We prefer the name *prunifoliella* group rather than the older “*prunetorum* group”, since the *prunifoliella* group contains several North American species, whereas the name “*prunetorum* group” was based on a single European species only.

**6** The date of publication of “Die Schmetterlinge Deutschlands und der Schweiz. Zweite Abtheilung. Kleinschmetterlinge. Band 2. Die Motten und Federmotten. Heft 2” has previously often been cited as 1877, the date that also figures on the Title page (https://archive.org/details/dieschmetterlin01heingoog). However, already Kirby concluded in the Zoological Record 13 (published 1878) on page 187: “Band ii, Die Motten ........ . though bearing date 1877, was published not later than November, 1876” (see also Sattler 1973). We therefore cite the paper as Heinemann and Wocke ([1876]).

**7** *Stigmella acerna* Puplesis, 1988. We synonymise the unavailable name *Stigmella acerifoliella* Dovnar-Zapolski, 1969, which was collected from *Acer turcomanicum* in Turmenistan, Kopet-Dag, the same type locality and host as for *Stigmella acerna* and with a similar mine form. The paper by Dovnar-Zapolski (1969) is poorly known, but contains a number of new names for Nepticulidae and other leafminers, all based on the mine alone. They are therefore not available (ICZN art. 13.6.2), and in many cases it is impossible to determine the identity of these names with the little information provided, but they do provide some interesting records.

**8** *Stigmella ulmivora* (Fologne, 1860). Hofmann (1858) named a species *Nepticula ulmella* HS. from Regensburg. This name should be regarded as an unavailable name (*nomen nudum*), since there is no description nor indication. This name is also a *nomen oblitum*, never cited again until Segerer (1997) synonymised it with *Stigmella ulmivora*. Thus no further action needs to be taken to reverse precedence to avoid rejecting the junior synonym *Stigmella ulmivora* or junior homonym *Nepticula ulmella* Braun, 1917 (now *Ectoedemia ulmella*).

**9** Stonis and Rocienė (2014) placed *Stigmella multispicata* Rocienė & Stonis, 2014 in the *Stigmella malella* group (that previously also contained the species of our *Stigmella rhamnella* group and in their vision also the *prunifoliella* group), but in fact the species is in all aspects extremely similar to *Stigmella ulmivora*, that only can be separated by details in the genitalia. It is therefore moved here, and it is highly likely that it feeds on *Ulmus*.

**Figures 41–47.**
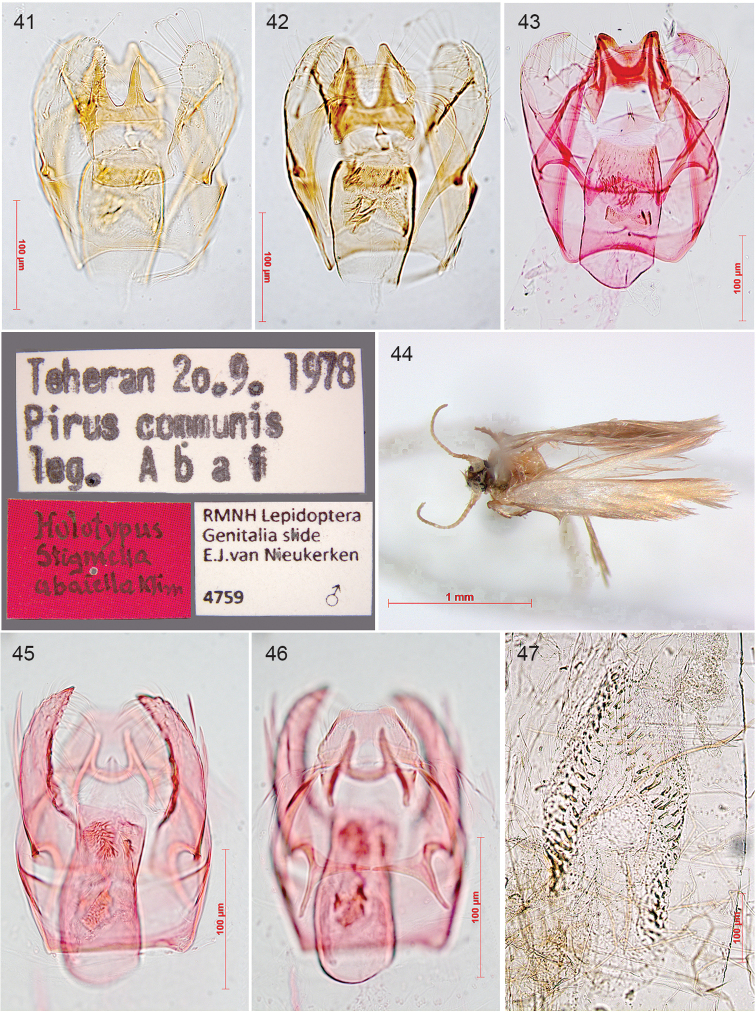
*Stigmella* species. **41–43** *Stigmella palionisi* Puplesis, 1984 **41, 42** Male genitalia of paratype of *Stigmella nakamurai* Kemperman & Wilkinson, Japan, slide VU 0790 **43** Male genitalia of specimen from Russia, Primorye, slide JCK8111. **44–47** *Stigmella abaiella* Klimesch, 1979 **44** Holotype male with labels **45, 46** Male genitalia of holotype, slide EvN4759 **47** Female genitalia, paratype, slide Klimesch 866.

**10** *Stigmella palionisi* Puplesis, 1984. We synonymise *Stigmella nakamurai* Kemperman & Wilkinson, 1985 from Japan with *Stigmella palionisi* from Russia: Primorye, on the basis of a comparison of descriptions, a male paratype slide of *Stigmella nakamurai* (Fig. 41, 42) and detailed photos of *Stigmella palionisi* genitalia (Rocienė and Stonis 2013; Stonis and Rocienė 2013) (also Fig. 43). This also confirms *Ulmus* as host for *Stigmella palionisi*. This species is also morphologically very similar to European *Stigmella viscerella* (Stainton, 1853), but the mines are somewhat different. Material: 1♂, Paratype *Stigmella nakamurai*, Hokkaido, Sapporo, em. 20.viii.1981, S. Nakamura, Host 0360 Ulmus davidiana v. japonica, slide VU no. 0790 (collection Sapporo).

**11** *Stigmella macrocarpae* (Freeman, 1967). We use the junior name for this North American oak mining species, earlier known as *Stigmella latifasciella* (Chambers, 1878), because its original combination *Nepticula latifasciella* is a junior primary homonym of *Nepticula latifasciella* Herrich-Schäffer, 1855, a junior synonym of *Stigmella hybnerella* (Hübner, 1796).

**12** *Stigmella birgittae* Gustafsson, 1985. We place the unavailable name *Nepticula amseli* Skala, 1941 as synonym under *Stigmella birgittae*. *Nepticula amseli* was described from mines from *Zizyphus spina-christi* in Jericho, Palestine. *Stigmella birgittae* is a common and widespread species in the Middle East on this host (van Nieukerken 2010).

**13** *Stigmella abaiella* Klimesch, 1979 and *Stigmella ficulnea* Puplesis & Krasnilnikova, 1994. The illustrated genitalia of both species are extremely similar. Study of the holotype and some paratypes of *Stigmella abaiella* confirmed this (Figs 44–47). It is possible that this represents a single species, but we hesitate to synonymize them, since *Stigmella abaiella* allegedly was reared from *Pyrus* and *Stigmella ficulnea* clearly is a *Ficus* miner. Since these hosts are totally unrelated, it is not very likely that one species feeds on both. Dr. Mansour Abaii (Teheran, e-mail 26.xii.2015 to EvN) confirmed that the identification of *Pyrus* must be correct, but no mines are kept. Awaiting further information from freshly collected larvae, we keep the species separate, but a synonymy is not excluded.

Material: Holotype ♂ *Stigmella abaiella*, Iran, Teheran, 20.ix.1978, Abai, *Pyrus communis*, Genitalia slide EvN4759 (Staatliches Museum für Naturkunde Karlsruhe).

**14** *Stigmella paliurella* Gerasimov, 1937. Previously the author for this species was given as (Klimesch, 1941), because Gerasimov (1937) based the name on the leafmine (van Nieukerken 1986a), following the rule that names based on the work of an animal published after 1930 are excluded from zoological nomenclature (ICZN art. 13.6.2). However, since Gerasimov also describes characters of the larva, the description fulfils the code (article 13.1), despite the brief description (van Nieukerken 2013) and the name is available and valid.

**15** *Stigmella turbatrix* Puplesis, 1994. We synonymise the unavailable name *Stigmella celtivora* Dovnar-Zapolski, 1969, which was described from leafmines on *Celtis* in Kazakhstan, Alma-Ata. See also note 4.

**Figures 48–56:**
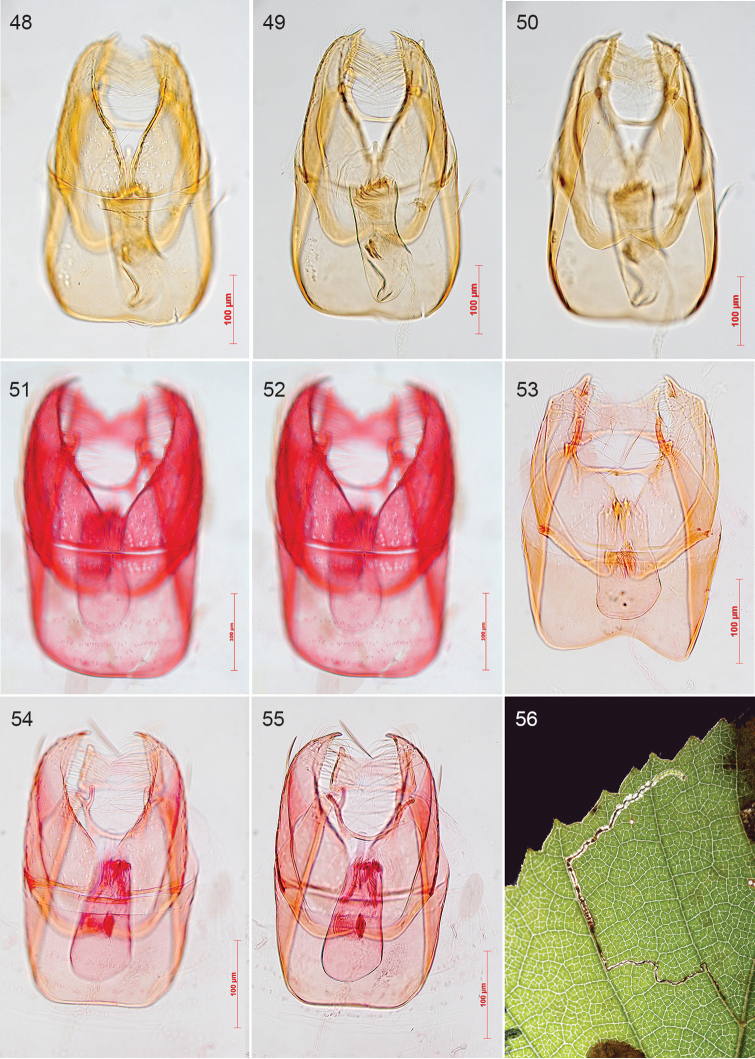
*Stigmella microtheriella*, male genitalia and leafmine (**56**). **48–50** Holotype of *Stigmella cathepostis* Kemperman & Wilkinson, 1985, slide VU0783 **51–52** slide EvN4629, Korea (Jeollanam-do) Wando Island, Hwaheung-ri, from *Carpinus tschonoskii*, slide EvN4629 **53** Russia, Primorye, slide JCK8117 **54–55** Greece (Messinía) Taygetos Mts, from *Ostrya carpinifolia*, slide EvN4430 **56** larva in leafmine on *Betula platyphylla*, Japan, Hokkaido, RMNH.INS.29748.

**Figure 57.**
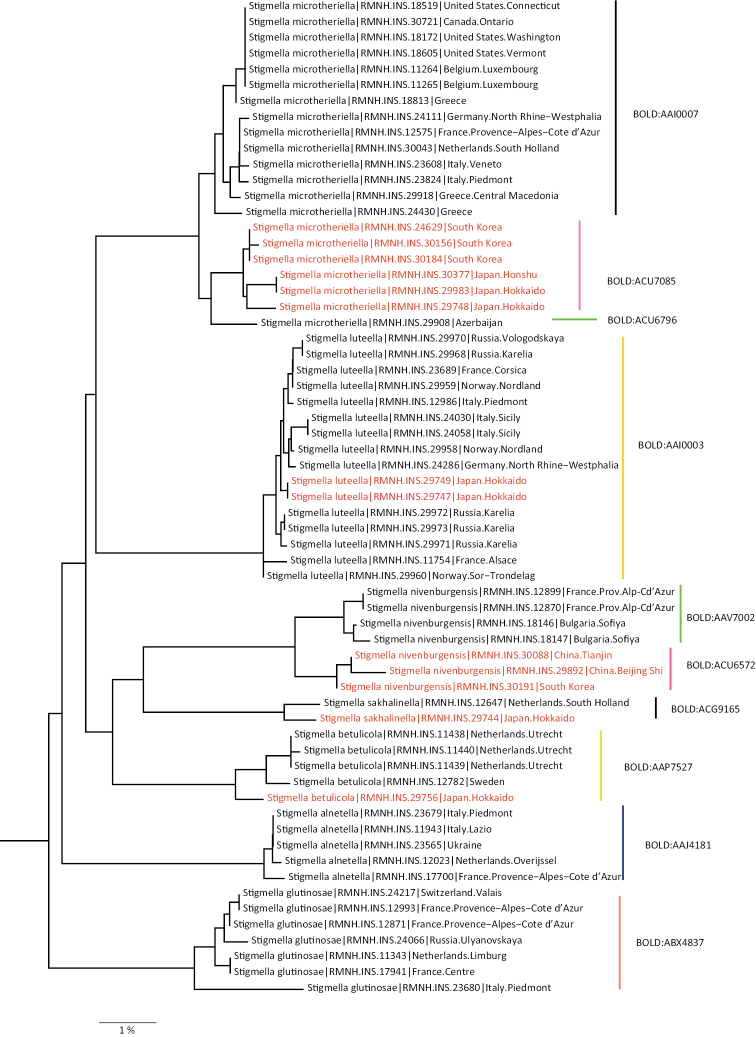
Neighbor Joining tree of DNA Barcodes of Palearctic members of the *Stigmella betulicola* group. East Palearctic records are presented in red, West Palearctic and imported North American records in black. Barcode Identification numbers are given at the right.

**16** *Stigmella microtheriella* (Stainton, 1854) was recorded from China (van Nieukerken and Liu 2000) and Japan (Hirano 2013). During our recent fieldwork in Korea and Japan we found larvae on *Carpinus*, *Corylus* and *Betula* (Hokkaido: RMNH.INS.29748; new host record, Fig. 56) (in Japan also found on *Ostrya*, N. Hirano pers. comm.) of which the DNA barcode shows a very short distance (1.11%) to European *Stigmella microtheriella*, still clustering together (BIN Korea/Japan: BOLD:ACU7085, Europe: BOLD:AAI0007) (see Fig. 57). We reared both males and females from these Asian specimens, whereas in most of Europe *Stigmella microtheriella* is parthenogenetic (but we reared males from Greece and Laštůvka and Laštůvka 1997 recorded males from Croatia). Originally we considered the presence of *Stigmella microtheriella* in East Asia as an introduction, but the separate, but similar barcode, suggests the species is an indigenous element of the East Asian fauna. By comparing male genitalia to the type of *Stigmella cathepostis* Kemperman & Wilkinson, 1985 (Figs 48–50), we realised that the latter is nothing else than male *Stigmella microtheriella* and hence synonymise it here, even though the uncus of east Palearctic specimens seems to have a deeper indentation than European males (compare Figs 48–53 with 54 and 55). *Stigmella cathepostis* was also recorded from Russia: Primorye (Rocienė and Stonis 2013), and we here also illustrate a male from Primorye (Fig. 53). It is unclear whether *Stigmella microtheriella* is a trans Palearctic species, since its best known hosts, *Corylus*, *Carpinus* and *Ostrya* do not have a continuous distribution, but show a gap between the Urals and the middle of China (Sokolov et al. 1980; Fang et al. 2011). A continuous distribution, however, might still be a possibility, since apparently *Betula*, that occurs throughout Siberia, can be an alternative host (see above and Fig. 56). Recently, *Stigmella microtheriella* was also recorded from North America (Eiseman and van Nieukerken 2015), but DNA barcodes show that here indeed it is an introduced species, as it is in New Zealand (Donner and Wilkinson 1989).

Material: Holotype ♂: [Japan, Kyushu], Hikosan, Buzen, 30.vii.1954, H. Kuroko, Host: *Carpinus tschonoskii* Maxim., genitalia slide VU no. 0783 (Entomological Laboratory, University of Osaka Prefecture).

**17** *Stigmella nivenburgensis* (Preissecker, 1942). We here synonymise *Stigmella populnea* Kemperman & Wilkinson, 1985. We found *Stigmella nivenburgensis* commonly on *Salix* in South Korea and China and DNA barcodes (BOLD:ACU6572) are rather close to European specimens (BOLD:AAV7002) (see Fig. 57). Although we did not yet collect mines on *Salix* in Japan, we consider it more likely that the mine on *Populus* from which the single specimen of *Stigmella populnea* was reared is *Stigmella nivenburgensis*, rather than a separate species, as in many other leafminers that feed both on *Salix* and *Populus*. The female genitalia are not different from European specimens, but are not very diagnostic in this group. The male genitalia figured as *Stigmella betulicola* from Russia: Primorye (Rocienė and Stonis 2013) belong in our opinion in fact also to *Stigmella nivenburgensis*.

**18** *Stigmella cornuta* Rocienė & Stonis, 2013. The authors of this Chinese *Quercus* feeding species erected a separate species group for it (Stonis et al. 2013e), but we think that the morphology fits well in the somewhat enlarged *betulicola* group as we define it here. Also *Stigmella xystodes* has relatively large cornuti, comparable to *Stigmella cornuta*.

**19** *Stigmella caryaefoliella* (Clemens, 1861). Wilkinson and Scoble (1979) synonymized *Stigmella caryaefoliella* and *Stigmella obscurella* (Braun, 1912) (see note 17) with *ostryaefoliella* on the basis of the male genitalia. We remove these synonyms here and recognize three good species feeding on different hosts and having quite different DNA barcodes and morphologies. Details to be published elsewhere.

**20** *Stigmella myricafoliella* (Busck, 1900) was described from Florida, Palm Beach, and reared from leafmines on *Morella cerifera* (as *Myrica cerifera*). We revise the synonymy of *Nepticula obscurella* Braun, 1912 as a synonym of *Stigmella myricafoliella*, on the basis of the host plant and genitalia. Details to be published elsewhere.

**21** *Stigmella ostryaefoliella* (Clemens, 1861). Wilkinson and Scoble (1979) synonymized *Stigmella caryaefoliella* (Clemens, 1861) and *Stigmella obscurella* (Braun, 1912) (see notes 16 and 17) with *ostryaefoliella* on the basis of the male genitalia. We remove these synonyms here and recognize three good species feeding on different hosts and having quite different DNA barcodes and morphologies. Details to be published elsewhere.

**Figures 58–62.**
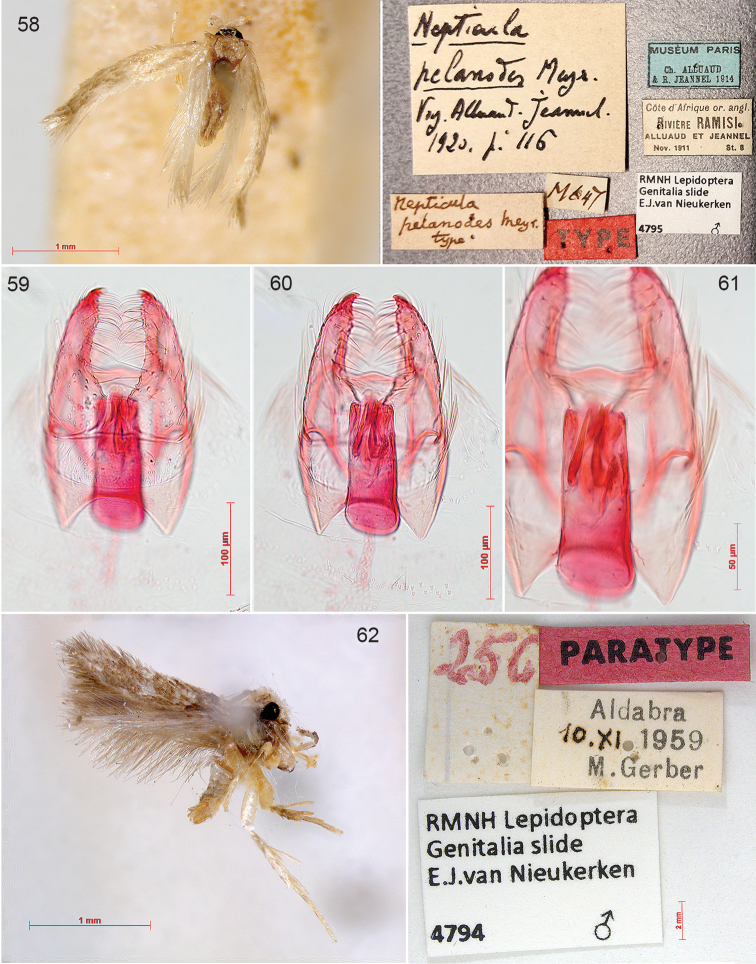
African *Stigmella* species. **58–61** *Stigmella pelanodes* (Meyrick, 1920), male holotype, labels and genitalia, slide EvN4795. **62** *Stigmella tropicatella* Legrand, 1965, male paratype and labels.

**22** *Stigmella pelanodes* (Meyrick, 1920). The holotype was examined by EvN (Figs 58–61): the genitalia are rather similar to *Stigmella xystodes*, we therefore place this species in the *betulicola*-group, together with several other African species that had been placed there before (Diškus and Puplesis 2003).

Material: Holotype ♂: [Kenya, Kwale] *Côte d’Afrique or. angl.*, Rivière RAMISI, ALLUAUD ET JEANNEL, Nov. 1911, St. 8;TYPE; “M.647”[Meyrick’s hand]; MUSÉUM PARIS, Ch. ALUAUD & R. JEANNEL, 1914; Nepticula pelanodes Meyr. type; RMNH Lepidoptera Genitalia Slide EvN4795 ♂ (MNHN).

**Figures 63–69.**
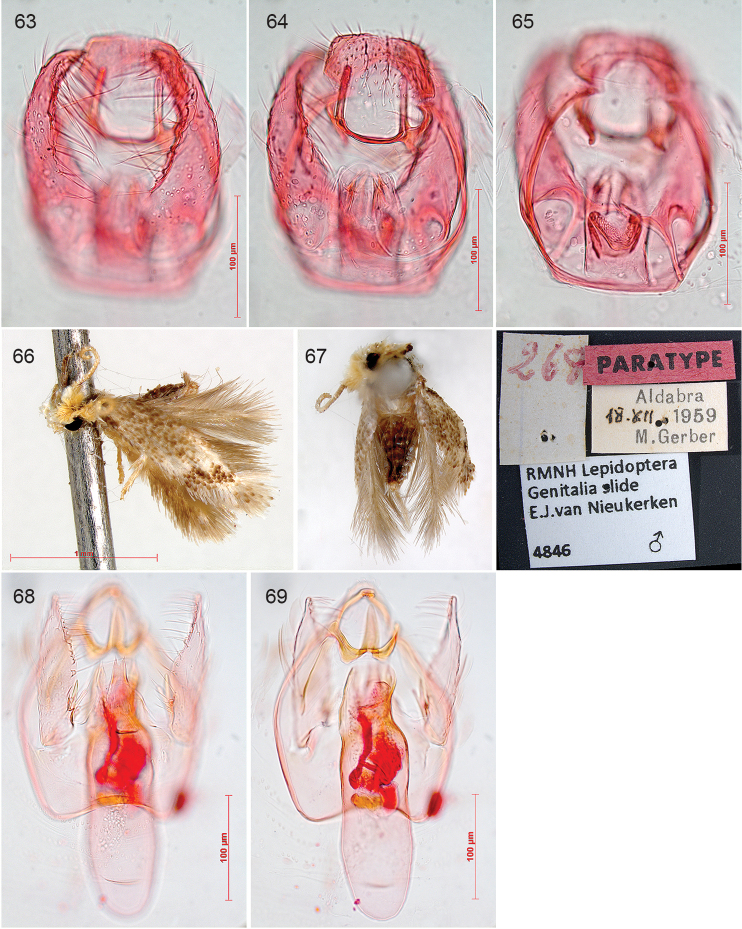
*Stigmella tropicatella* Legrand, 1965, paratypes. **63–65** Male genitalia of male in Fig. 62, slide EvN4794, on which identity of species is based **66–69** *Acalyptris* sp., misidentified paratype of *Stigmella tropicatella*, male, labels and male genitalia, slide EvN4846.

**23** *Stigmella tropicatella* Legrand, 1965 is the only nepticulid species known from the islands in the Indian Ocean off the African continent (Madagascar excluded) as found on the atoll of Aldabra. The holotype, unfortunately, is almost completely destroyed, only a head and part of the thorax remain on the minuten pin. There are several paratypes, three of which EvN examined. Two are males of a *Stigmella*, the third is a male of an unnamed *Acalyptris*. Comparing the remains of the holotype and the description, we are convinced that the *Stigmella* paratypes are the real *Stigmella tropicatella*, and hence base the identity on these (Figs 62–65). It is a typical tropical *Stigmella* species in the *betulicola* group, rather similar to *Stigmella satarensis* Scoble, 1978. The unnamed *Acalyptris* is illustrated in Figs 66–69.

Material: Adult Holotype, [specimen almost completly disappeared, only a head on pin, [Seychelles], Aldabra, 11.xi.1959, M. Gerber (Muséum National d’Histoire Naturelle, Paris). Further specimens see S1.

**Figures 70–75.**
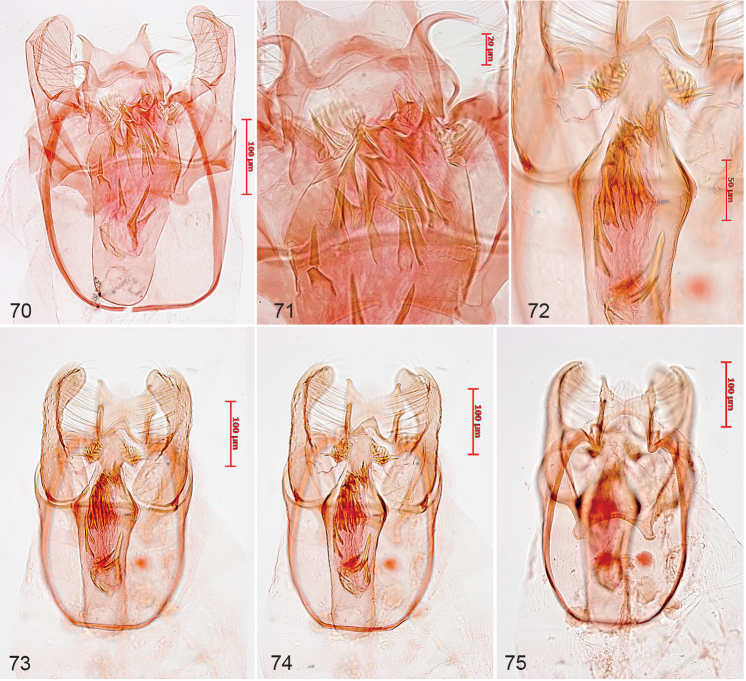
*Stigmella wollofella* (Gustafsson, 1972), male genitalia. **70–71** *Nepticula mandingella* Gustafsson, 1972, holotype, slide NHRS4874 **72–75** *Nepticula wollofella* Gustafsson, 1972, holotype, slide NHRS5103.

**24** *Stigmella wollofella* (Gustafsson, 1972). Study of the slides of the holotypes of *Nepticula wollofella* Gustafsson, 1972 (Figs 72–75) and *Nepticula mandingella* Gustafsson, 1972 (Figs 70–71), both collected together in Gambia, showed that the genitalia are in fact identical, but the slide of *Stigmella mandingella* is more squashed, obscuring some of the characters. Gustafsson (1985) gave the shape of the juxta as distinguishing character. The juxta is arrow shaped, with many small spines on the lateral apexes of the arrow head. These were not figured in the original drawing of *Nepticula mandingella*, but are present in the slide, although difficult to separate from spines on the phallus tube. Because both *Nepticula mandingella* and *Nepticula wollofella* were described in the same publication, we determine here as first reviser the relative priority of *Nepticula wollofella* (ICZN article 24.2) and synonymise *Nepticula mandingella*. The much better genitalia preparation of *Nepticula wollofella* was the first criterion and the second one is that the name *wollofella* has been used again by Gustafsson (1985) when describing the biology of the species as leafminer of *Zizyphus*.

Material: Holotype ♂ *Nepticula wollofella* Gustafsson, Gambia: Gambia river between Bathurst and Basse Santa Su., on riverboat M/S Lady Wright in flash light, 5.xii.1970, B. Gustafsson Genitalia slide 5103 (NHRS). Holotype ♂ *Nepticula mandingella* Gustafsson, Gambia: Gambia river between Bathurst and Basse Santa Su., on riverboat M/S Lady Wright in flash light, 5.xii.1970, B. Gustafsson Genitalia slide 4874 (NHRS).

**25** *Stigmella rosaefoliella* (Clemens, 1862). The subspecies *Stigmella rosaefoliella pectocatena* Wilkinson & Scoble, 1979 is removed as a synonym to *Stigmella centifoliella*, see there.

**26** *Stigmella ogygia* group. Since our studies show that all New Zealand *Stigmella* species – as far as studied - belong to one monophyletic clade, we group them as the *Stigmella ogygia* group, based on the widespread and common *Stigmella ogygia* (Meyrick, 1889), which is readily recognizable on host-plant alone (herbaceous *Senecio*, Asteraceae).

**27** *Stigmella epicosma* group. Puplesis and Robinson (2000) placed many Neotropical species in an enlarged *salicis* group because of the similarity in male genitalia. Our phylogeny (Doorenweerd et al. 2016a) gives the two examined Neotropical species of this group (both Asteraceae feeders) as sister to the *salicis* group s.str., whereas the only Neotropical *Salix* feeder *Stigmella molinensis* van Nieukerken & Snyers, 2016 is sister to the remaining Holarctic *salicis* group members. Because of the strong difference in host plant choice, all *salicis* group members but one feed on Salicaceae, whereas the Neotropic species feed on Asteraceae and various other families, and because of the strong apomorphy of the signa band in the female genitalia for the *salicis* group, we separate all Neotropic species (apart from *molinensis*) and place them in their own *epicosma* group.

**28** *Stigmella costalimai* (Bourquin, 1962) and *Stigmella guittonae* (Bourquin, 1962) are tentatively moved to the *epicosma* group because their host plants belong to Asteraceae, resp. *Tessaria integrifolia* Ruiz & Pav. and *Senecio bonariensis* Hook. & Arn. (Bourquin 1961) and so far all known Neotropic Asteraceae feeders belong to this group. Puplesis and Robinson (2000) overlooked the original host plant data for *Stigmella costalimai*. The record of *Ludwigia longifolia* (DC.) H.Hara (Onagraceae) as one of the host plants for *Stigmella guittonae* by Bourquin (as *Jussiaea longifolia*) is most likely an error: just after this description he wrote the description of a Momphid, *Psacophora orfilai*, making leafmines on that host. Momphidae frequently feed on Onagraceae.

**29** Species described by Klimesch in his paper “Neue *Stigmella*-Arten (Lep., Stigmellidae)” in the “Zeitschrift der Wiener Entomologischen Gesellschaft vol 31(9–12) have been incorrectly cited by us and others as Klimesch, 1946 (van Nieukerken 1986a; 2013; Diškus and Puplesis 2003). However, this issue, 9–12 of volume 31, for the year 1946 was published on 15 March 1948 as can be seen on the first page of that issue, 129 (http://www.zobodat.at/pdf/ZOEV_31_0129.pdf) (“Ausgegeben 15.März 1948”).

**30** *Stigmella salicis* (Stainton, 1854) forms a complex of species that requires revision: some synonyms may be one of the constituent species, others have to be described as new (van Nieukerken et al. 2012a).

**31** *Stigmella centifoliella* (Zeller, 1848). *Nepticula centifoliella* was first named by Heyden (1843) when describing *Nepticula*. Since this was a short message during a meeting, there is no description, and only a vague indication to unspecified earlier papers (“Von einer Art (der *Centifoliella*) kannten schon de Geer und Goeze die eigenthümlich gebildete Raupe”). We consider this insufficient to make the name available, and thus keep Zeller, 1848, who described the species in detail, as author (see also Wilkinson 1978). We synonymise *Stigmella rosaefoliella pectocatena* Wilkinson & Scoble, 1979 from Canada with *Stigmella centifoliella*. The authors overlooked the fact that this represents in fact the introduced European species, which is rather different from North American *Stigmella rosaefoliella*, that only externally superficially resembles it and has very different genitalia. Details will be published elsewhere.

Material: Holotype ♀ *Stigmella rosaefoliella pectocatena* Wilkinson & Scoble: Canada, Ontario, Ottawa, emerged 24.ix.1962, leafmines on *Rosa* sp., 62-11, Freeman & Lewis, genitalia slide CNC3292 [not found] (Canadian National Collection of Insects, Arachnids, and Nematodes) .

**Figure 76.**
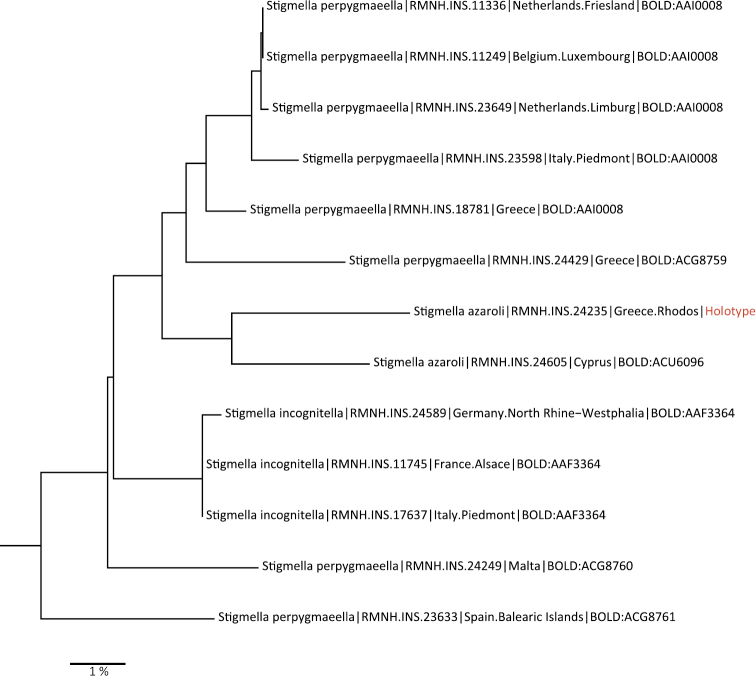
Neighbor Joining tree of DNA Barcodes of the *Stigmella incognitella* group, showing the paraphyly with regards to *Stigmella azaroli* and *Stigmella incognitella* and large distances for specimens identified as *Stigmella perpygmaeella* from Malta and Mallorca. Specimens from Cyprus are identified as *Stigmella azaroli*, of which the holotype from Rhodos was also barcoded.

**32** *Stigmella azaroli* (Klimesch, 1978) is very similar to *Stigmella perpygmaeella*, but the moth is usually much paler. DNA barcodes of *Stigmella perpygmaeella*, *incognitella* and *azaroli* form a tangled cluster, where *Stigmella perpygmaeella* is paraphyletic, and island forms have very different barcodes from continental populations (Fig. 76). In this unclear situation we rather do not synonymise *Stigmella azaroli*, until more reared material and DNA analyses are available (BIN’s: *Stigmella perpygmaeella*, most of Europe: BOLD:AAI0008, Greece, Holotype *Stigmella azaroli*: BOLD:ACG8759, *Stigmella azaroli* Cyprus: BOLD:ACU6096, *Stigmella incognitella*: BOLD:AAF3364, “*Stigmella perpygmaeella*” Malta: BOLD:ACG8760, Mallorca: BOLD:ACG8761).

Material: Holotype ♂ *Nepticula azaroli*: Greece, Rodos, Rodini, 22.ix.1972, mines on *Crataegus azarolus*, em. 11.x.1972, J. Klimesch, Genitalia slide EvN4235, DNA barcode RMNH.INS.24235 (Zoologische Staatssammlung München).

**33** *Stigmella magdalenae* (Klimesch, 1950). This species was known as *Stigmella nylandriella* or *Nepticula nylandriella* in most literature prior to Borkowski (1975) and in Britain prior to Emmet (1979). See Schoorl et al. (1985).

**34** *Stigmella nylandriella* (Tengström, 1848). Before the type of *Lyonetia nylandriella* was re-examined in the 1970’s, this species was known as *Stigmella aucupariae* (Frey, 1857) and *Stigmella* or *Nepticula nylandriella* referred to the species now known as *Stigmella magdalenae* (Borkowski 1975; Schoorl et al. 1985).

**35** *Stigmella oxyacanthella* (Stainton, 1854). The finding of larvae on *Crataegus*, *Malus* and *Amelanchier* in North America apparently having the same DNA barcode as European *Stigmella oxyacanthella* (BOLD:AAF3421) led EvN to re-examine specimens identified as *Stigmella pomivorella* (Packard, 1870) in CNC and USNM. All show genitalia inseparable from *Stigmella oxyacanthella*, and we thus synonymise *Stigmella pomivorella*. This leafminer apparently was already introduced in the USA during the 19^th^ century. Details will be published elsewhere.

**36** *Stigmella micromelis* Puplesis, 1985. After the finding that Siberian larvae on *Crataegus* share the barcode with Korean and Japanese larvae found on *Aria alnifolia* (= *Sorbus alnifolia* or *Micromelis alnifolia*) (BOLD:ACK9547), and the fact that genitalia of *Stigmella micromelis* and *Stigmella crataegivora* Puplesis, 1985 are indistinguishable, we synonymise both species here. Since both species were named in the same publication, we here determine as First Reviser (ICZN art 24.2) that *Stigmella micromelis* has priority over *crataegivora*. Photos of the genitalia of the types were given by Stonis and Rocienė (2013). A paper with more details is in preparation.

**37** *Stigmella amelanchierella* (Clemens, 1862) **stat. rev**. *Stigmella amelanchierella* was described on the basis of leafmines only. Subsequent authors have been unable to rear or to identify the species and Newton and Wilkinson (1982) were even uncertain about its generic status. We found several times leafmines with green larvae on *Amelanchier*, that fit Clemens’ description well. DNA barcodes of these showed two different clusters, both closely related to other members of the *oxyacanthella* group, such as *Stigmella crataegifoliella*, one occurring more west in Colorado and Canada (BOLD:ACG5879), another east in Tennessee, Virginia and Massachusetts (BOLD:ACG8835). We consider it likely that the last group represents the real *Stigmella amelanchierella*, since Clemens described it from Pennsylvania. Even though we have not yet studied a single adult, we consider it likely to be a separate species in the *Stigmella oxyacanthella* group, of which the identity is based on the DNA barcode.

**38** *Stigmella purpuratella* (Braun, 1917). After study of the holotype slides we synonymise *Stigmella scinanella* Wilkinson & Scoble, 1979 with *Stigmella purpuratella*. Already Newton and Wilkinson (1982) suggested both species cannot be distinguished, except by the paler and less iridescent forewings of *Stigmella purpuratella*. This difference can easily be explained by the much older age of the *purpuratella* specimens, but this text did not seem to be supported by the very different drawings of the genitalia of both species in the same paper. After checking the slides it can be concluded that the valvae of *Stigmella scinanella* were drawn incorrectly, and the drawing of the phallus of *Stigmella purpuratella*, which is a reconstruction of a broken phallus, is turned upside down, making the characteristic longer cornuti pointing posteriorly rather than anteriorly, and the phallus tube is an incorrect reconstruction. The species makes leafmines on *Crataegus* that are inseparable from those of *Stigmella crataegifoliella*. Figures will be published elsewhere.

Material: Holotype ♂ *Nepticula purpuratella*: (United States), Pennsylvania, Pittsburgh, 30.v.1906, Engel, Genitalia slide CNC3467 P. Newton (USNM).

Holotype ♂ *Stigmella scinanella*: [Canada], Ontario, Normandale, mines 26.vii.1956 on *Malus*, 56-154, Freeman & Lewis, Genitalia slide MIC7102 (=CNC2972) (Canadian National Collection of Insects, Arachnids, and Nematodes) .

**39** *Stigmella aurella*/*ruficapitella* cluster. The remaining *Stigmella* species form a well supported clade in our multi-gene molecular analyses (Doorenweerd et al. 2016a; Doorenweerd et al. 2016b), that is also relatively well recognisable by genitalia characters, such as the presence of a manica (phallocrypt) in the male genitalia and a large accessory sac in the female genitalia. However, within this cluster the relationships are confusing and many unsupported clades appear in various analyses, often at different places. Some supported groups stand out, including the complete *aurella* group (including *Stigmella lediella*), the *Stigmella floslactella* group and the core *Stigmella ruficapitella* group s.s. Also part of the *lemniscella* group (formerly *marginicolella* group) is well supported: viz. *Stigmella continuella*, *Stigmella lemniscella* + *Stigmella zelkoviella*, usually with poorer support linked to *Stigmella apicialbella* and an unnamed *Betula* feeding North American species, but always never with *Stigmella gimmonella* included (only one specimen sequenced). The other Fagaceae mining species, including the North American *Stigmella procrastinella* and *Stigmella alba* almost always form a poorly supported clade together with the core *ruficapitella* group, which we therefore together classify as the *Stigmella ruficapitella* group s.l., that is here restricted to Fagaceae feeding species only. *Stigmella speciosa*, *lonicerarum* and relatives, previously placed with the *ruficapitella* group, always group outside. A *Stigmella sorbi* group is never recognised in molecular analyses and even the morphologically similar *Stigmella sorbi* and *Stigmella plagicolella* are never placed close to each other. On the other hand, *Stigmella amygdali*, previously placed in a monotypic species group, always groups with moderate support together with *Stigmella plagicolella*, forming a *Prunus* feeding clade. This situation can currently not be easily translated into monophyletic species groups, and apart from the well supported ones, we recognise tentatively a *Stigmella speciosa* group for the non-Fagaceae miners previously placed in the *ruficapitella* group (or the *hemargyrella* group) and an enlarged *sorbi* group, at the moment a kind of waste bin for mainly Rosaceae feeding species, but also including *Stigmella hamamelella* on *Hamamelis*. We leave *Stigmella zagulaevi* from the Caucasus and *Stigmella talassica* from the Tyan Shan in the *lemniscella* group, although we are not convinced that this is the correct placement.

**40** *Stigmella monticulella* Puplesis, 1984. We synonymise here *Stigmella gracilipae* Hirano, 2014 from Japan. Both taxa make linear leafmines on *Lonicera*¸ occur in East Asia and have inseparable genitalia (Stonis and Rocienė 2013; Hirano 2014). The species is also close to *Stigmella lonicerarum* (Frey, 1857) from Europe.

**41** *Stigmella filipendulae* (Wocke, 1871). After *Stigmella ulmariae* (Wocke, 1879) had earlier been synonymized (van Nieukerken et al. 2012a), we also synonymise *Stigmella palmatae* Puplesis, 1984 from Russia: Primorye, feeding on *Filipendula palmatae*, on the basis of the similar genitalia (Stonis and Rocienė 2013). *Stigmella filipendulae* is apparently a widespread Palearctic *Filipendula* feeding species.

**42** *Stigmella lediella* (Schleich, 1867). Puplesis (1994) already synonymized the two *Rhododendron* feeding species from Russia: Primorye, and here we also add the Japanese *Rhododendron* feeder to the synonymy: *Stigmella sesplicata* Kemperman & Wilkinson, 1985. The original description only dealt with females, but meanwhile male genitalia have been described (Hirano 2013) and are identical to *Stigmella lediella*. We have no DNA barcodes yet from Japan, but those from Korea are almost identical to those from Europe and have the same BIN (BOLD:AAL6954). *Stigmella rhododendrifolia* Dovnar-Zapolski & Tomilova, 1978 is an unavailable name, based on leafmines from Siberia. Considering the fact that all *Rhododendron* leafmines belonging to *Stigmella* from eastern Europe to Japan apparently are all *Stigmella lediella*, we conclude that this name also belongs here.

**Figures 77–79.**
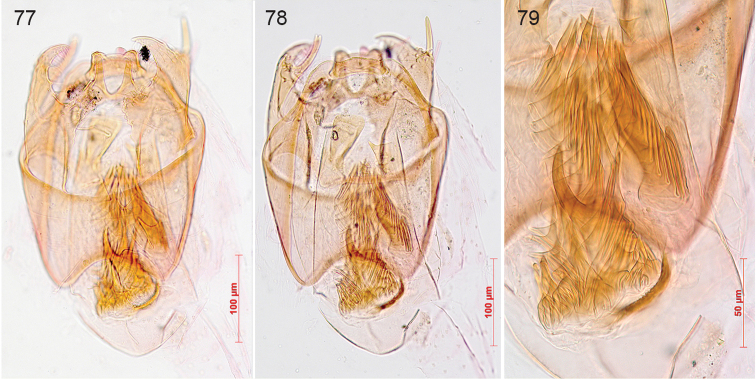
*Stigmella longispina* Puplesis, 1994, male genitalia, Tajikistan, 30 km N Dushanbe, Kondara, 20.viii.1989, R. Puplesis, slide JCK8318, RMNH.INS.15381.

**43** *Stigmella longispina* Puplesis, 1994 is here moved to the *aurella* group on the basis of similarity in its male genitalia as shown in a single studied specimen from Tajikistan (RMNH.INS.15381) (Figs 77–79).

**44** *Stigmella spiculifera* Kemperman & Wilkinson, 1985. *Stigmella oa* Kemperman & Wilkinson, 1985 was described on the basis of a single female from Japan. After carefully comparing descriptions and figures, we can only conclude that it is indistinguishable from the *Rubus* feeding *Stigmella spiculifera* and hence synonymize it here.

**45** *Stigmella lurida* Puplesis, 1994. On the basis of DNA analysis of one female from Altai, that consistently groups with *Stigmella sorbi*, we place *Stigmella lurida* in the *Stigmella sorbi* group (RMNH.INS.24818).

**46** *Stigmella humboldti* Remeikis & Stonis, 2015 was described from a single female reared from *Quercus humboldti* in Colombia. The authors could not place this species in a known group, due to the spiny signa on the female bursa. Since we see some resemblance with the spiny signa of *Stigmella procrastinella* and *Stigmella alba*, both shown to be *Quercus* feeders as well (DNA barcode data), we place *Stigmella humboldti* close to these species.

**47** *Stigmella samiatella* (Zeller, 1839). We list here two previously overlooked Herrich-Schäffer names: *Nepticula quercella* Herrich-Schäffer, 1863a was merely published as a name in a checklist (abbreviated as *Nepticula querc*.: Herrich-Schäffer followed the rule of fixed endings, -ella for Tineina, so that everybody knew this to be written as *quercella*). This unavailable nomen nudum was synonymised by Segerer (1997). *Nepticula chaoniella* Herrich-Schäffer, 1863b was described in a paper on the Lepidoptera of Engadin, in comparison with another new species, *Nepticula alpinella* (see note 67). Herrich-Schäffer wrote: “Durch letzteres Merkmal unterscheidet sie sich auf den ersten Blick von der eichenbewohnenden *chaoniella* m. (früher unter *samiatella*, aber ohne verdickte Schuppen der männlichen Hfl.)” [By the last character it is separated on first sight from the oak feeding *chaoniella* m[ihi] (earlier under *samiatella*, but without thicker scales in the male hindwing)]. Like many early authors Herrich-Schäffer mixed several oak mining *Stigmella* species and separates his *chaoniella* from male *atricapitella* or *ruficapitella* [named by him *samiatella*], that both show these thicker (androconial) scales. From this it is clear that *chaoniella* can only be a synonym of *Stigmella samiatella*. Further the name *Nepticula chaoniella* can be considered a nomen oblitum, we are not aware of any later use of this name.

**48** *Bohemannia pulverosella* (Stainton, 1849). We synonymise *Bohemannia piotra* Puplesis, 1984 from Russia: Primorye, because it is indistinguishable in genitalia from *pulverosella*, and makes similar mines on *Malus* as European *pulverosella*. We expect that the species has a continuous distribution throughout Siberia. In Europe most populations of *Bohemannia pulverosella* seem to be parthenogenetic without any males, and only a few males are known (van Nieukerken and Johansson 1990).

**49** *Bohemannia quadrimaculella* (Boheman, 1853). The publication year for the original paper has been cited as 1851, 1852 or 1853. Boheman’s paper was published in the volume for the year 1851 that according to the title page was published in 1853. On the title page of the article is printed: “Inlemnad den 6 Mars 1852 [submitted on March 6, 1852], probably causing the incorrect references to 1852. The synonymy of *Bucculatrix antispilella* Meess, 1907 established by van Nieukerken and Johansson (1990), who incorrectly cited 1910 as year of description, in fact was already spotted shortly after its description by Disqué (1912) (see Hausenblas 2009).

**50** *Bohemannia manschurella* Puplesis, 1984. We synonymize here *Bohemannia nipponicella* Hirano, 2010 from Japan with *Bohemannia manschurella* from Russia: Primorye. Hirano (2010) described *Bohemannia nipponicella* as different in the cornuti, comparing it to the original description of *Bohemannia manschurella*, but by comparing the photos of the types of both species (Hirano 2010; Stonis and Rocienė 2013) and the study of material from Russia and Japan, no difference can be seen at all.

**51** *Glaucolepis oishiella* (Matsumura, 1931) **comb. n.** The female holotype of *Trifurcula oishiella* Matsumura 1931 was examined, and compared to the description of *Sinopticula sinica* Yang 1989; both species were described as gall maker on *Prunus*. Further, material collected in China and Japan helped to establish that this concerns a single species, with gall making larvae on *Prunus*. Further details will be published elsewhere.

*Translation of the original description in Japanese by Matsumura*:

“Head orange yellow, setae on both sides grey-white. Antenna grey, base of several segments with dark rings. Body and forewing dark brown with a little purple colour ribbon and also with reflection. Scales large, tip of wing spoon-like with long hairscales. Hindwing dark. Legs brown. Hindtibia dorsally with half erect setae. Larvae on *Prunus*, boring into branches, making a gall. In mid-June to late-June in Japan, Honshu”. [*Translated by Liu Youqiao, August 1996*]

Material: Holotype *Trifurcula oishiella* ♀, Japan, Honshu, reared from galls on *Prunus*, T. Oishi, Genitalia slide EvN2916 (Entomological Institute, Hokkaido University).

**Figures 80–85.**
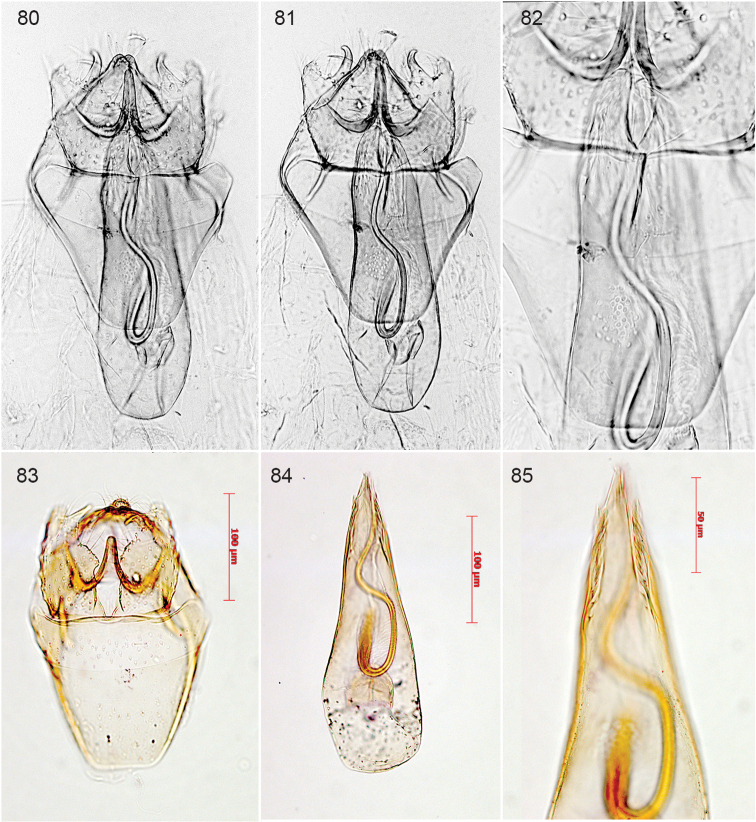
*Glaucolepis* species, male genitalia. 80–82 *Glaucolepis hamirella* (Chrétien, 1915), lectotype, slide KL0718 **83–85** *Glaucolepis saturejae* (Parenti), Italy (Asti) fraz. Valmanera, Oasi WWF, 13.vii.2006, G. Baldizzone, slide EvN3749.

**52** *Glaucolepis hamirella* (Chrétien) and *Glaucolepis saturejae* (Parenti). The holotype of *Glaucolepis hamirella* is very similar to male *Glaucolepis saturejae* (Figs 80–85), and a synonymy was considered. However, DNA barcodes of Italian populations are very different from those from Morocco, Cyprus and Turkey together, and both clusters show again a lot of variation. A detailed morphological and molecular study is required to solve the status of this complex and until then we keep the species separate. Since the type of *Glaucolepis hamirella* is from North Africa and *saturejae* from northern Italy, we consider the Turkish/Moroccan cluster as *Glaucolepis hamirella*, the others as *Glaucolepis saturejae*. Some specimens of *Glaucolepis saturejae* studied by EvN earlier may carry labels with the name *Trifurcula hamirella*.

Material: Holotype *Nepticula hamirella*: ♂, Algeria: Biskra, v.1909, Chrétien, Genitalia slide KL0718 (MNHN).

**Figures 86–89.**
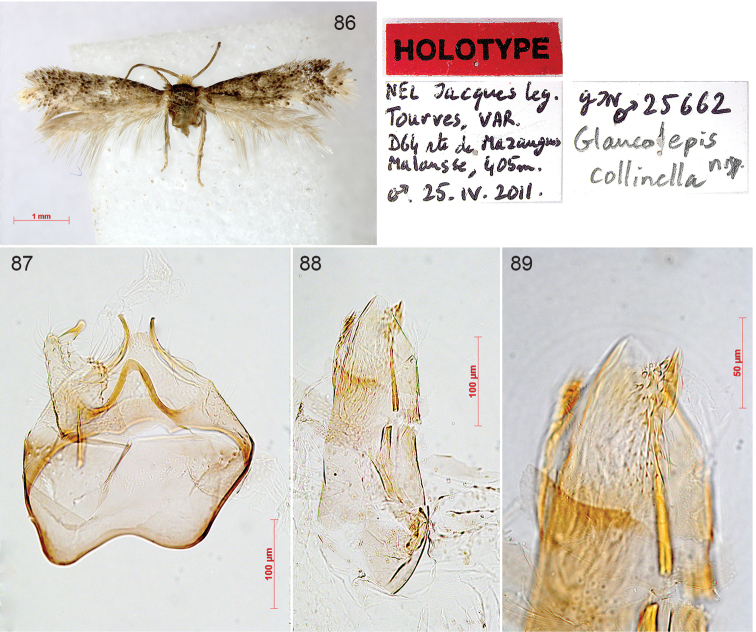
*Glaucolepis magna* (Laštuvka, A. & Z., 1997), holotype of *Trifurcula collinella* Nel, 2012, adult, labels and male genitalia.

**53** *Glaucolepis magna* (A. Laštůvka & Z. Laštůvka, 1997). The recently described *Trifurcula collinella* Nel, 2012 from France is here synonymised with *Glaucolepis magna*. The holotype was examined (Figs 86–89), and the relatively large moth shows clearly the typical dorsal spot of this species and the genitalia are similar (e.g. Laštůvka and Laštůvka 1997; van Nieukerken et al. 2006). The long processes of the tegumen, as described by Nel (2012), are in fact the arms of the uncus, that is broken in the middle (Fig. 87).

Material: Holotype *Trifurcula collinella* ♂, France, Var, Tourves, D64, Rte de Mazaugues, Malausse, 25.iv.2011, Jacques Nel, Genitalia slide JN25662 (J. Nel, personal collection, later to be deposited in the Tiroler Landesmuseum Ferdinandeum).

**Figure 90.**
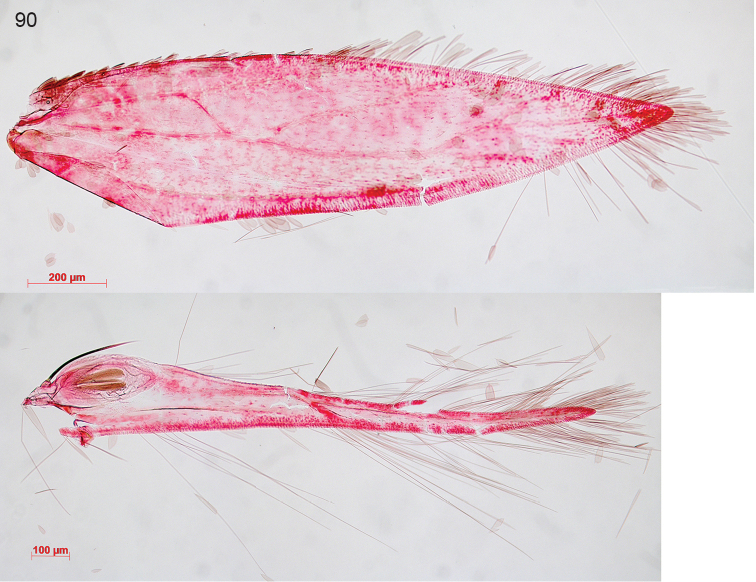
*Glaucolepis argentosa* Puplesis & Robinson, 2000, paratype, venation of right wings, slide 4502, RMNH.INS. 24502. Note the absence of Cu in forewing, absence of trifurculate Rs+M in hindwing and presence of two groups of special scales on humeral lobe.

**54** *Glaucolepis*: “unassigned to group”. The two Neotropical species placed in *Glaucolepis* (Puplesis and Robinson 2000) almost certainly do not belong there. The male genitalia show some resemblance to species in the *Glaucolepis raikhonae* group. However, we examined three males of *Glaucolepis argentosa* Puplesis & Robinson, 2000, which show none of the apomorphies of *Glaucolepis* + *Trifurcula* (van Nieukerken 1986b), the trifurculate hindwing (Fig. 90), “velvet patch on underside hindwing” or the three pairs of abdominal tufts. The hindwing does show two remarkable groups of special scales on the humeral lobe (Fig. 90). It is possible that this species forms yet another Neotropical generic group. Unfortunately we have only fragments of the DNA barcode, that do not provide enough information on its taxonomic place. *Glaucolepis aerifica* again has very different genitalia and may belong to an entirely different clade.

**Figures 91–92.**
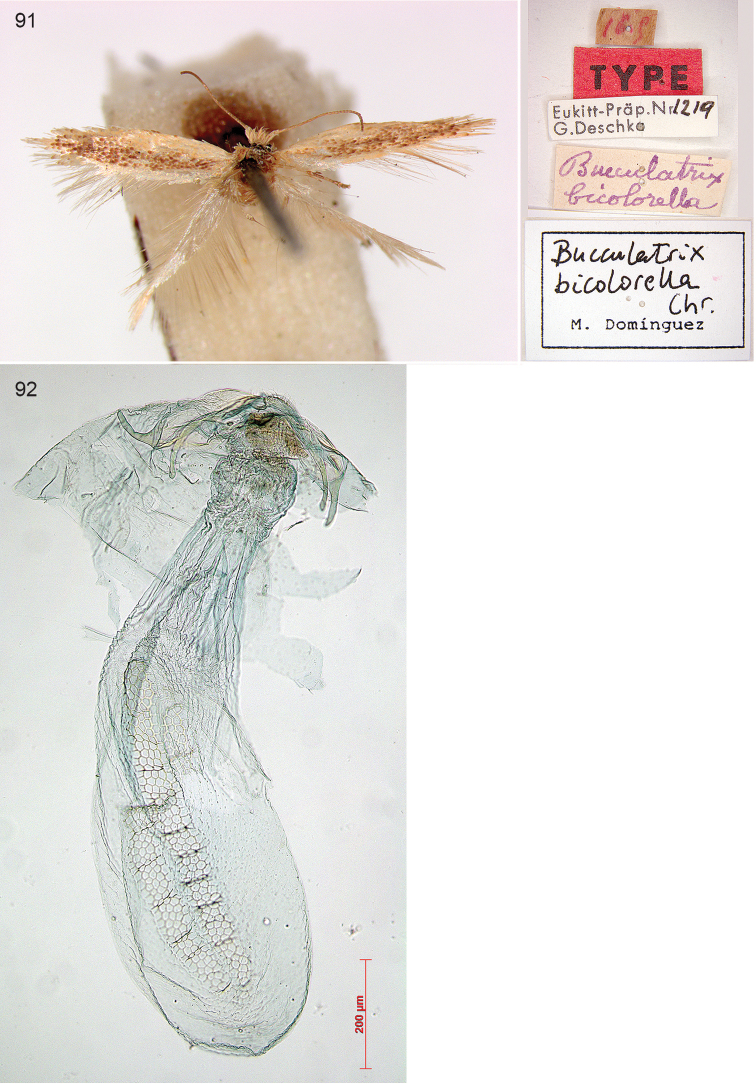
*Trifurcula bicolorella* (Chrétien, 1915), holotype female, labels and genitalia, slide Deschka 1219.

**55** *Trifurcula bicolorella* (Chrétien, 1915) **comb. n.** This species was described as *Bucculatrix* and not recognized as nepticulid, until Zdeno Tókar (Slovakia) identified it as a *Trifurcula* when studying sketches of Western Palearctic *Bucculatrix* genitalia made by Gerfried Deschka. Unfortunately the holotype (Figs 91, 92) is a female, and female genitalia are not very diagnostic in this group, and no males are available. The wing markings resemble *Trifurcula aurella* Rebel, 1933, that feeds on *Spartium junceum*, and one unnamed species from Spain that feeds on *Retama* species. On the type locality most likely only *Retama* species occur, more North African material is needed to establish the identity of this species firmly.

Material: Holotype ♀, [Tunisia, Gafsa, Mai 1907–1912], “169”, “Type”, “Eukitt-Präp. Nr. 1219 G. Deschka, “Bucculatrix bicolorella” (Muséum National d’Histoire Naturella, Paris).

**Figures 93–97.**
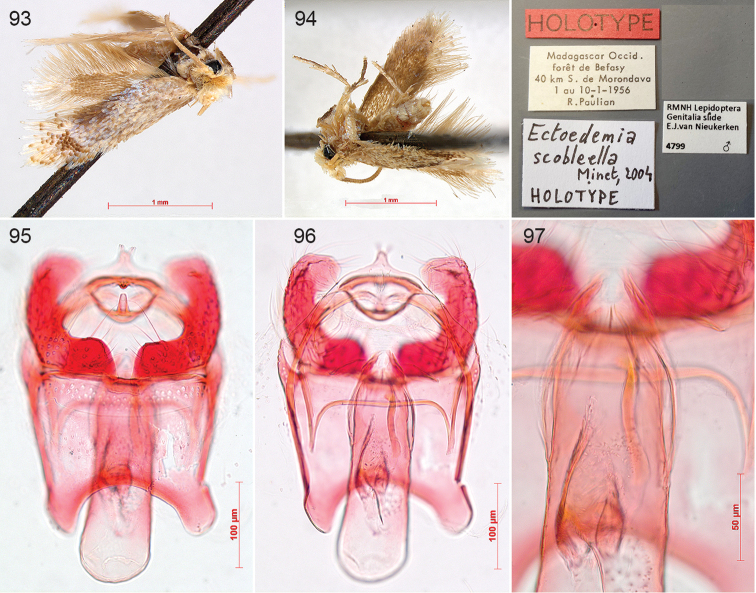
*Fomoria scobleella* (Minet, 2004), holotype male, labels and male genitalia.

**56** *Ectoedemia scobleella* Minet, 2004 (replacement name for the junior homonym *Ectoedemia scoblei* Minet, 1990) is here recombined with *Fomoria* on the basis of examination of the type series (Figs 93–97).

Material: Holotype ♂, Madagascar, Forêt de Befasy, 40 km S. de Morondava, 1–10.i.1956, R. Paulian, Genitalia slide EvN4799 (Muséum National d’Histoire Naturelle, Paris).

**57** *Fomoria argyraspis* (Puplesis & Diškus, 1995) **comb. n.** We here tentatively move this species from *Acalyptris* to *Fomoria* on the basis of the genitalia and externals that are very similar to a number of *Fomoria* species and not to any other *Acalyptris* species.

**Figures 98–101.**
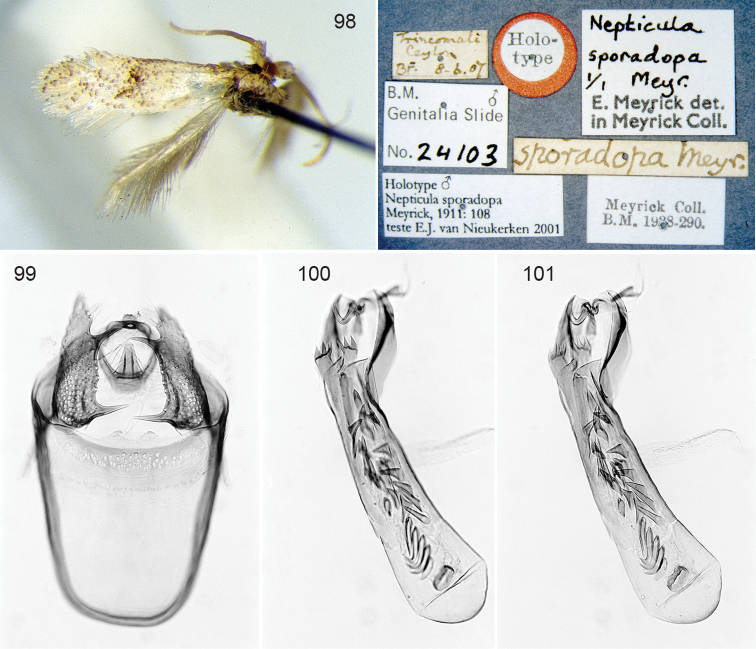
*Fomoria sporadopa* (Meyrick, 1911), holotype male, labels and male genitalia.

**58** *Fomoria sporadopa* (Meyrick, 1911) **comb. n.** This species from Sri Lanka is very difficult to place. EvN studied the holotype, the only known specimen, in 1986 (Figs 98–101). The venation, studied in the intact specimen shows a curved main trunk of R+Rs+M but further details were hardly visible; the abdomen has a triangular S2a (van Nieukerken 1986b), both speaking against placement in *Acalyptris* where Diškus and Puplesis (2003) placed it. The genitalia are rather peculiar, without transtilla, and elaborate carinal processes. For the time being we place this in *Fomoria*, that comes closest, but it may well belong to a separate clade.

Material: Holotype ♂, [Sri Lanka, Eastern Province] Ceylon, Trincomali, 8.vi.1907, BF [T. Bainbrigge Fletcher], Genitalia slide BM24103 (Natural History Museum, London).

**59** *Etainia trifasciata* (Matsumura, 1931). Although the holotype of *Nepticula trifasciata* Matsumura is badly damaged, the forewing pattern is clearly identical to that of *Obrussa tigrinella* Puplesis, 1985. Since this species is common in Japan, and no other species with the same colour pattern occur there, we can safely conclude there is only one trifasciate *Etainia* in Japan, hence we synonymise *Obrussa tigrinella* here.

*Translation of the original description in Japanese by Matsumura*:

“Body and wing with grey-yellow and white with reflection. Head orange yellow, both sides silvery white. Antenna grey white. Forewing darker with three bands. One near wing base, one in middle and a little one outwards. Third fascia at the apex. Terminal and dorsal cilia grey-yellow white. Costal fringe darker. Hindwing darker. Legs dark grey. Seems to be the smallest moth in Japan. Early September. Hokkaido.” [*Translated by Liu Youqiao, August 1996*]

**60** *Acalyptris falkovitshi* (Puplesis, 1984). We synonymize here *Microcalyptris arenosus* Falkovitsh, 1986 and *Microcalyptris vittatus* Puplesis, 1984, after earlier *Microcalyptris turanicus* Puplesis, 1984 was synonymized with *Acalyptris falkovitshi* (van Nieukerken 2010). We act as First Reviser (ICZN article 24.2) to identify *Microcalyptris falkovitshi* as having priority to the other species that were published in the same paper (*vittatus* and *turanicus*). The slight differences given earlier (Puplesis 1990; 1994) do not hold when a larger sample is studied: the sclerotization of the T-shaped sclerites on segments 4–8 may vary, probably depending on age. This is the larger species in this group, usually with a longitudinally brown stripe on forewings, a pointed gnathos and conspicuous tufts on tergites 4–8.

**61** Unplaced *Acalyptris*. Several of these are morphologically rather different from other *Acalyptris* species. *Acalyptris distaleus* almost certainly does not belong in this genus, it is part of a group of species, occurring all in southern California and Arizona.

**62** *Zimmermannia*. The North American species of *Zimmermannia*, previously recognised as the *Ectoedemia castaneae* group, were revised within the revision of *Ectoedemia* (Wilkinson 1981; Wilkinson and Newton 1981). They recognised in all 12 species, but failed to provide sufficient diagnostic characters or keys to separate all these often extremely similar species, several of which were based on a single specimen only. Whereas some lectotypes were selected, a major shortcoming was that the types of the oldest name in this group, *Trifurcula obrutella* Zeller, had not been examined, and the synonymy of *Nepticula bosquella* Chambers with *obrutella*, earlier suggested by Busck (1903) was simply accepted. Comparing the descriptions it is clear that Zeller described the pale headed species (“Kopf bleich lehmgelblich”= Head pale loam yellowish), whereas Chambers described the black headed species. Examination of the *obrutella* types confirms this and leads to a change of the interpretation of *obrutella* as the species currently known as *Ectoedemia piperella* Wilkinson & Newton, 1981, thus reviving *obrutella’s* former synonym *bosquella* as the valid name for the black headed species. Also the identity of the Lectotype of *Nepticula grandisella* Chambers, 1880 (indicated as Holotype by Wilkinson 1981) lead to a re-interpretation of this name, here synonymised with *Ectoedemia chloranthis* Meyrick, 1928 and *Ectoedemia acanthella* Wilkinson & Newton, 1981.

To establish the identities of these old names firmly, we designate lectotypes below for *Trifurcula obrutella* and *Nepticula grandisella*. As a result of our revision we reduce the number of valid species in Eastern North America to five, synonymising eight names, and reviving two old names. We will more extensively diagnose and illustrate these species in a forthcoming publication. There are more North American species in this genus, particularly in the West and South, but these are all unnamed species. Material: *Nepticula grandisella* Chambers: Lectotype ♂ (designated here), MCZ-ENT00001302, [United States], Texas, [Bosque Co., Norse, Gustav W. Belfrage], Chambers, Type 1302, Genitalia slide CNC 3495 (Museum of Comparative Zoology, Harvard University).

*Trifurcula obrutella* Zeller: Lectotype ♂ (designated here), MCZ-ENT00014248, [United States], Texas, Dallas, Boll, Type 14248, Genitalia slide DRD 2936 (Museum of Comparative Zoology, Harvard University). For the data of the other types in this group see Wilkinson and Newton (1981).

**63** *Ectoedemia insularis* Puplesis, 1985 was described from two specimens from Sakhalin. We transfer the species here to the *populella* group. In the male genitalia it resembles *Ectoedemia intimella* (Zeller, 1848) closely, a species also recorded from east Asia, and even Sakhalin. We compared genitalia of specimens reared from *intimella*-like leafmines on *Salix* in South Korea, and also examined light collected adults from Korea and an earlier reported female from Japan (van Nieukerken et al. 2012b). The male genitalia of these are identical to those of the *Ectoedemia insularis* holotype, illustrated by Stonis and Rocienė (2013), and show a slight, but constant difference to European populations: European *Ectoedemia intimella* has a wider gnathos and the ventral carinae are wider apart than in *insularis*. Adult externals, larva and biology of East Asian and European populations are identical, but there is a large DNA barcoding gap (more than 5%). On the base of these findings we consider all East Asian “*intimella*” as *Ectoedemia insularis* and keep both tentatively as separate species. Study of populations and their phylogeography between East Asia and Europe is needed to establish the status of these populations more firmly. BIN’s: *Ectoedemia insularis*: BOLD:AAD0468, *Ectoedemia intimella*: BOLD:AAD0467.

**64** *Ectoedemia sinevi* Puplesis, 1985. This species is placed in the *populella* group on the basis of its male genitalia, and DNA barcodes of material that we consider conspecific. This species will be redescribed in a forthcoming paper on Japanese *Ectoedemia* (Hirano et al. in prep.).

**65** *Ectoedemia turbidella* (Zeller, 1848). *Ectoedemia similigena* Puplesis, 1994 was described from a single series from Yalta on the Crimea. Earlier we found the slight differences in genitalia just sufficient to keep it tentatively as a separate species (van Nieukerken et al. 2010). Meanwhile, we have been able to obtain a partial DNA barcode of the dissected and illustrated paratype male (RMNH.INS.23924, genitalia slide EvN3924). The 420 basepairs are almost the same as in Western European specimens of *Ectoedemia turbidella*, with just 2 basepairs difference, and BOLD classifies the barcode in the same BIN (BOLD:AAD4374). This small difference over a large geographic gap, together with the weak differences observed earlier, prompt us to reverse our earlier opinion, and synonymize *Ectoedemia similigena* with *Ectoedemia turbidella*.

**66** *Ectoedemia spiraeae* Gregor & Povolný & *jacutica* Puplesis, 1988. Recently, Stonis et al. (2015b) reinstated *Ectoedemia jacutica* Puplesis, 1988 as valid species, after we had synonymised it earlier with *Ectoedemia spiraeae* (van Nieukerken et al. 2010). In China and Japan we (and Japanese collectors) collected a species on *Spiraea* and *Aruncus* that we hitherto identified as *Ectoedemia spiraeae*, although it has a large barcode gap with European specimens (van Nieukerken et al. 2012b). Considering the distribution being rather close to the type locality Yakutsk and some morphological similarities, we consider it likely that the Japanese and NE Chinese populations belong to *Ectoedemia jacutica* (with BIN BOLD:AAI9354). The situation is comparable to that of *Ectoedemia insularis* and *Ectoedemia intimella* (above) and thus studies of populations between these are needed for more final conclusions. For now we consider specimens from the much more western Altai (van Nieukerken et al. 2010) to belong to *Ectoedemia spiraeae*.

**67** *Ectoedemia minimella* (Zetterstedt, 1839). The North American *Ectoedemia canadensis* (Braun, 1917) is here synonymised with *Ectoedemia minimella*. Details will be published elsewhere.

**68** *Ectoedemia occultella* (Linnaeus, 1767). This is one of the most widespread Nepticulidae, occurring from westernmost Europe to Japan and throughout northern North America, extending south in the mountain ranges to Colorado and Tennessee. The north American form was described as *Ectoedemia lindquisti* (Freeman, 1962), but we see no reason to treat North American populations differently from Palearctic ones. DNA barcodes: Europe to Mongolia: BOLD:AAD0469; Japan: BOLD:ACU6927; North America: BOLD:AAH4532, distance 2.73%.

**69** *Ectoedemia ulmella* (Braun, 1912). We synonymize *Ectoedemia andrella* Wilkinson, 1981 on the basis of the description and illustrations of the holotype. The genitalia are indistinguishable, and also the characteristic androconial scales on the hindwing are identical.

**70** *Nepticula alpinella* Herrich-Schäffer, 1863b, *Nepticula alticolella* Herrich-Schäffer, 1863c and *Nepticula reuttiella* Herrich-Schäffer, 1863c. These three names were described in a paper dealing with a collecting trip to Engadin, in the Swiss Alps, and are available names. However, they have never appeared again in the literature, apart from Snellen (1873), who listed the names as missing from the Staudinger & Wocke catalogue (Wocke 1871), and these names can thus easily be considered Nomina Oblita (ICZN art. 23.9) whenever they are threatening stability of any junior synonyms. Only the name *reuttiella* was picked up in the cardindex of the London Natural History Museum (Beccaloni et al. 2005), and as a consequence appears on some websites that are copying these names uncritically. Looking at the descriptions and localities, *Nepticula alpinella* may refer to *Stigmella thuringiaca*, *Nepticula alticolella* could be *Stigmella stelviana* or *dryadella* and *Nepticula reuttiella* an abberant *Stigmella prunetorum*.

**71** *Nepticula oritis* Meyrick, 1910. This species was described from a single specimen from India, Himachal Pradesh, Simla Hills. It is probably a fasciate *Stigmella* species, of which there are many, that can only be identified with genitalia. Meyrick (1910) stated in the introduction that the material was from the Indian Museum (in Kolkota), but we failed to get any information on the whereabouts of the holotype, and therefore this species is better treated as a *Nomen oblitum*, until a type turns up.

**72** *Nepticula xuthomitra* Meyrick, 1921. Meyrick described this species on the basis of one specimen from Pretoria, that unfortunately is no longer present in TM or BMNH. The description is too vague to identify the species with any certainty, and since it is very well possible that *Nepticula xuthomitra* is in fact a senior synonym of one of the currently recognized valid species, it is best regarded as a *Nomen oblitum*. Once a synonymy is established, it is best to follow up with an action as described in ICZN 23.9, if this name threatens the stability of nomenclature of another species.

**73** *Nepticula anguinella* Clemens, 1861 and *Nepticula platea* Clemens, 1861. These names were given to larvae in incomplete leafmines on oaks. We think that the description can be best interpreted as *Stigmella* species, even though Wilkinson (1981) recombined them with *Ectoedemia*. He did this on the base of the larva having “ten square brown or blackish spots”, indeed a character of several *Ectoedemia* species. However, we do not know of any oak feeding *Ectoedemia* in North America with this character, certainly the common *Ectoedemia similella* Braun does not have such plates. *Stigmella* larvae that feed with the venter upwards, such as those in the *saginella* group, also show dots: the often conspicuous ganglia. Since North American *Stigmella* oak mines are all very similar, there is no way to identify these species on the basis of the very short descriptions, and by absence of any reared adults or other type material we think it is better to leave these names as *Nomina oblita*. If at any time, a synonymy of these species can be established, these names almost certainly have priority to the currently valid names; we therefore strongly advise taxonomists then to reverse priority according to ICZN 23.9, declare these names as *Nomen oblitum*, and declare the junior synonym as *Nomen protectum*.

**74** *Unplaced unavailable names*. These names are partly copied from van Nieukerken (1986a) and we add a whole series of names by Dovnar-Zapol’skij from Russia and Central Asia, based on leafmines only, in two poorly known publications (Dovnar-Zapolski 1969; Dovnar-Zapolski and Tomilova 1978). Several of these almost certainly belong to known species, but *Stigmella atraphaxidella* refers to an interesting new host association (*Atraphaxis*, Polygonaceae). The few names that we could identify have been listed under the respective valid names (*Stigmella acerna*, *Stigmella turbatrix*, *Stigmella lediella*, see notes 4, 12 and 39).

**Figures 102–105.**
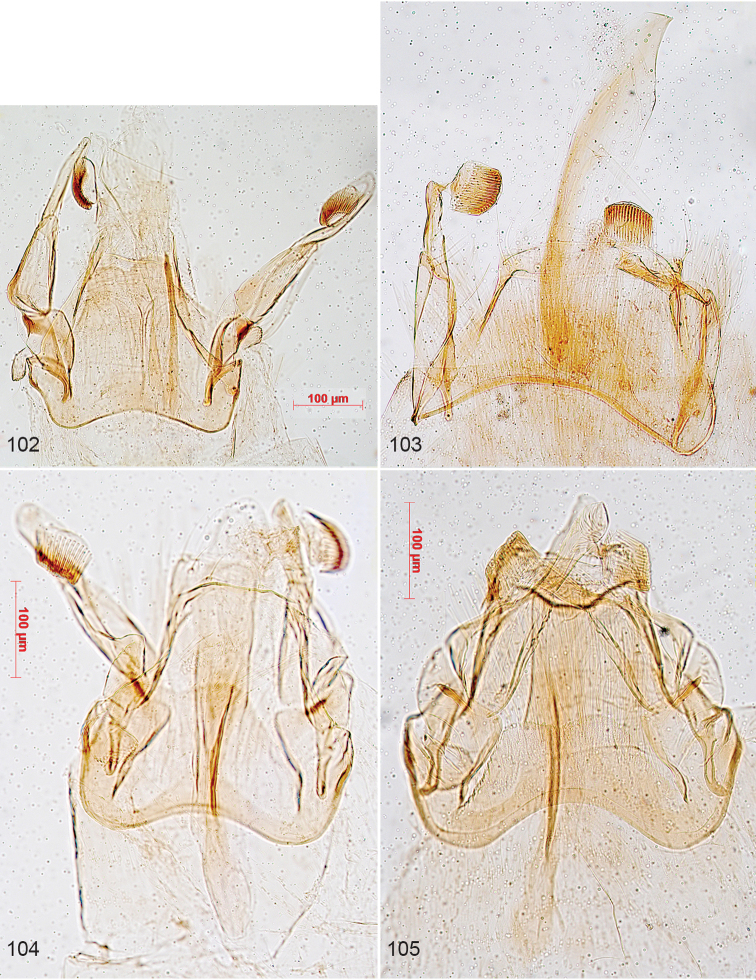
South African *Opostegoides* species, male genitalia. **102** *Opostegoides granifera* (Meyrick, 1913), holotype, slide TM4044 **103** *Opostegoides melitardis* (Meyrick, 1918), holotype, slide TM4037 **104** *Opostegoides pelocrossa* (Meyrick, 1928), holotype, slide TM4042 **105** *Opostegoides praefusca* (Meyrick, 1913), holotype, slide TM4043.

**75** *Opostegoides*. We recombine four South African species, now in *Opostega*, with this genus after study of genitalia slides of type material, prepared by L. Vári: *Opostegoides granifera* (Meyrick, 1913) **comb. n.** (Fig. 102), *Opostega melitardis* (Meyrick, 1918) **comb. n.** (Fig. 103), *Opostega pelocrossa* (Meyrick, 1928) **comb. n.** (Fig. 104), *Opostega praefusca* (Meyrick, 1913) **comb. n.** (Fig. 105).

**76** *Opostega cretatella* Chrétien, 1915. Unfortunately no type material of this species could be found. The species has been described from Algeria, Biskra, in April, on the northern edge of the Sahara. It is a very large species, with a wingspan of 14.5 mm. When comparing the description with that of *Opostega rezniki* Kozlov, 1985, the similar large size (16–17 mm) and uniform colour pattern with a single dorsal dot is striking. Also *Opostega rezniki* is described from a desert/steppe habitat, in Kazakhstan, 150 km NNE of Almaty, Sarytaukum, flying in mid-May. We consider it very likely that they represent the same species, with a similar distribution as many desert dwelling species, such as *Acalyptris psammophricta* (see van Nieukerken 2010) and thus synonymise them here. Obviously a confirmation from new material collected in North Africa would be welcome.

**77** “*Opostega*”. Until the eighties of the last century all Opostegids were placed in the single genus *Opostega* Zeller. After the generic revision (Davis 1989) most species have been moved to *Pseudopostega* or *Opostegoides*, but a revision of the African and Australian species has not yet taken place. Since *Opostega* s.str. is really a rather small Palearctic genus, and probably most Australian and African belong to *Pseudopostega* or *Opostegoides*, we prefer to place the species that have not yet been examined in detail tentatively in a separate “*Opostega*”. We have been able to place several South African species correctly, since Lajos Vári had already dissected many types, present in the Ditsong Museum of Natural History in Pretoria (former Transvaal Museum).

**78** *Pseudopostega chalcopepla* (Walsingham, 1908). We synonymize *Pseudopostega cyrneochalcopepla* Nel & Varenne, 2012 on the basis of virtual identical genitalia (Figs 113, 114) and external characters. We examined and barcoded the paratype. The barcodes of the two specimens from Mediterranean islands (Corsica and Cyprus: RMNH.INS.24603) show large barcode distances to the mainland populations, but otherwise we find no differences. For now we prefer to keep these all in one variable species, until more is known of these and other island populations. Island populations often have large barcoding gaps to mainland populations, which taken alone is in our opinion not sufficient for species status.

**Figures 106–114.**
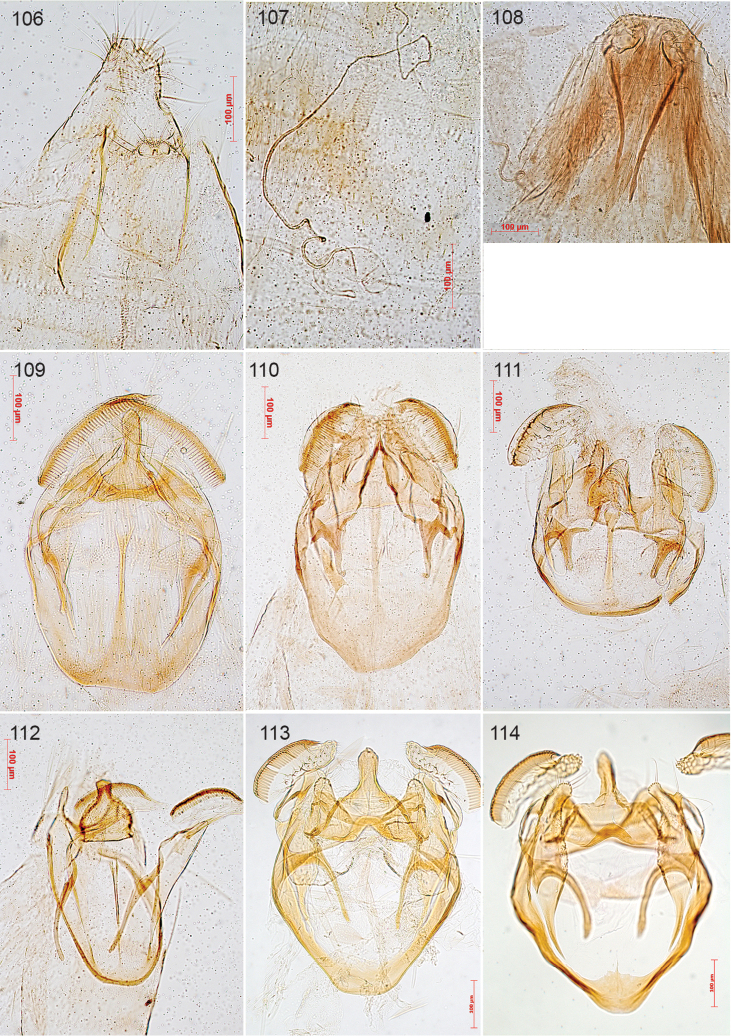
*Pseudopostega* species, female (106–108) and male genitalia. **106, 107** *Pseudopostega idiocoma* (Meyrick, 1918), syntype, slide TM4036 **108** *Pseudopostega orophoxantha* (Meyrick, 1921), holotype, slide TM4034 **109** *Pseudopostega amphimitra* (Meyrick, 1913), syntype, slide TM4039 **110** *Pseudopostega phaeosoma* (Meyrick, 1928), holotype, slide TM4046 **111** *Pseudopostega symbolica* (Meyrick, 1914), holotype, slide TM4038 **112** *Pseudopostega tincta* (Meyrick, 1918), holotype, slide TM4035 **113, 114** *Pseudopostega chalcopepla* (Walsingham, 1908): **113** paratype of *Pseudopostega cyrneochalcopepla* Nel & Varenne, 2012, slide J Nel 24980 **114** France, Var, Mazaugues, slide EvN4279.

Material: *Pseudopostega cyrneochalcopepla*: 1♂ paratype, [France, Corsica] Pertusato, Bonifacio, 24.v.2011, P. Varenne, DNA extracted from 1 leg, RMNH.INS.550071, collection J. Nel (later to be deposited in the Tiroler Landesmuseum Ferdinandeum).

**79** *Pseudopostega* (African species). We recombine six South African species, now in *Opostega*, with this genus after studying genitalia slides of type material, prepared by L. Vári: *Pseudopostega amphimitra* (Meyrick, 1913) **comb. n.** (Fig. 109), *Pseudopostega idiocoma* (Meyrick, 1918) **comb. n.** (Fig. 106, 107), *Pseudopostega orophoxantha* (Meyrick, 1921) **comb. n.** (Fig. 108), *Pseudopostega phaeosoma* (Meyrick, 1928) **comb. n.** (Fig. 109), *Pseudopostega symbolica* (Meyrick, 1914) **comb. n.** and *Pseudopostega tincta* (Meyrick, 1918) **comb. n.**

**80** *Nepticula belfrageella* Chambers, 1875a. The slide labelled “Type” is not a nepticulid, but belongs to an unidentified Gelechiidae (personal communication J.F. Landry, D. Adamski). Also the description most likely does not refer to a nepticulid species, so we exclude it here.

Material: Genitalia slide ♂ examined, CNC3496, *Nepticula belfrageella* Cham, #99, #1555, no specimen [note in pencil], TYPE, G.L.[ewis], remounted by PJN.[ewton] (USNM).

## Note added in proof

Just before publication, another new species of Nepticulidae was published, and two more described, but not named (Stonis et al. 2016c). The following species should be added to page 115, the *Stigmella epicosma* group. The species has also been added to the online databases.

*Stigmella polylepiella* Diškus & Stonis in Stonis et al., 2016c: 86 NEO

Stonis JR, Diškus A, Remeikis A, Karsholt O (2016c) Do leaf-mining Nepticulidae occur in the natural but so threatened Andean *Polylepis* forests? Biologija 62: 83–97. doi: 10.6001/biologija.v62i2.3334

## Supplementary materials

Supplementary material 1 Specimen data for Catalogue of Nepticuloidea with BOLD data Data type: Microsoft Excel (xls). Explanation note: The 213 records comprise specimens that have either been cited briefly or completely in the Notes, or have been used more implicitly for the texts and figures. Records are listed alphabetically by species, country, stateProvince, AdminDivision2, AdminDivision3, Locality. This dataset is made available under the Open Database License (http://opendatacommons.org/licenses/odbl/1.0/). The Open Database License (ODbL) is a license agreement intended to allow users to freely share, modify, and use this Dataset while maintaining this same freedom for others, provided that the original source and author(s) are credited.Erik J. van Nieukerken, Camiel Doorenweerd, Robert J. B. Hoare, Donald R. Davis

Supplementary material 2
BOLD TaxonID Tree of the dataset Lepidoptera - Nepticuloidea of the World 2016 [DS-NEPCAT] Data type: PDF file Explanation note: A Neighbor joining tree of COI sequences, with Kimura 2 Parameter as distance model for sequences linger than 200 bp, 3203 records. This dataset is made available under the Open Database License (http://opendatacommons.org/licenses/odbl/1.0/). The Open Database License (ODbL) is a license agreement intended to allow users to freely share, modify, and use this Dataset while maintaining this same freedom for others, provided that the original source and author(s) are credited.Erik J. van Nieukerken, Camiel Doorenweerd, Robert J. B. Hoare, Donald R. Davis
