# Supplementary material for: Revised classification and catalogue of global Nepticulidae and Opostegidae (Lepidoptera, Nepticuloidea)
Source: Zookeys. 2016 Oct 31;(628):65–246. doi: 10.3897/zookeys.628.9799 (PMC5126388; doi:10.3897/zookeys.628.9799)
Supplement: Supplementary material 2 — BOLD TaxonID Tree of the dataset Lepidoptera - Nepticuloidea of the World 2016 [DS-NEPCAT] [file zookeys-628-065-s002.pdf]

# BOLD TaxonID Tree

| | | |
|---|---|---|
| Title | : | Lepidoptera - Nepticuloidea of the World 2016 [DS-NEPCAT] |
| Date | : | 18-August-2016 |
| Data Type | : | Nucleotide |
| Distance Model | : | Kimura 2 Parameter |
| Marker | : | COI-5P |
| Codon Positions | : | 1st, 2nd, 3rd |
| Labels | : | Country & Province, SampleID, BIN uri |
| Filters | : | Length > 200 |
| Colorization | : | [blue]=Stop Codons [red]=Contamination or misidentification |

| | | |
|---|---|---|
| Sequence Count | : | 3203 |
| Species count | : | 776 |
| Genus count | : | 24 |
| Family count | : | 2 |
| Unidentified | : | 2 |

| | | |
|---|---|---|
| BIN Count | : | 900 |

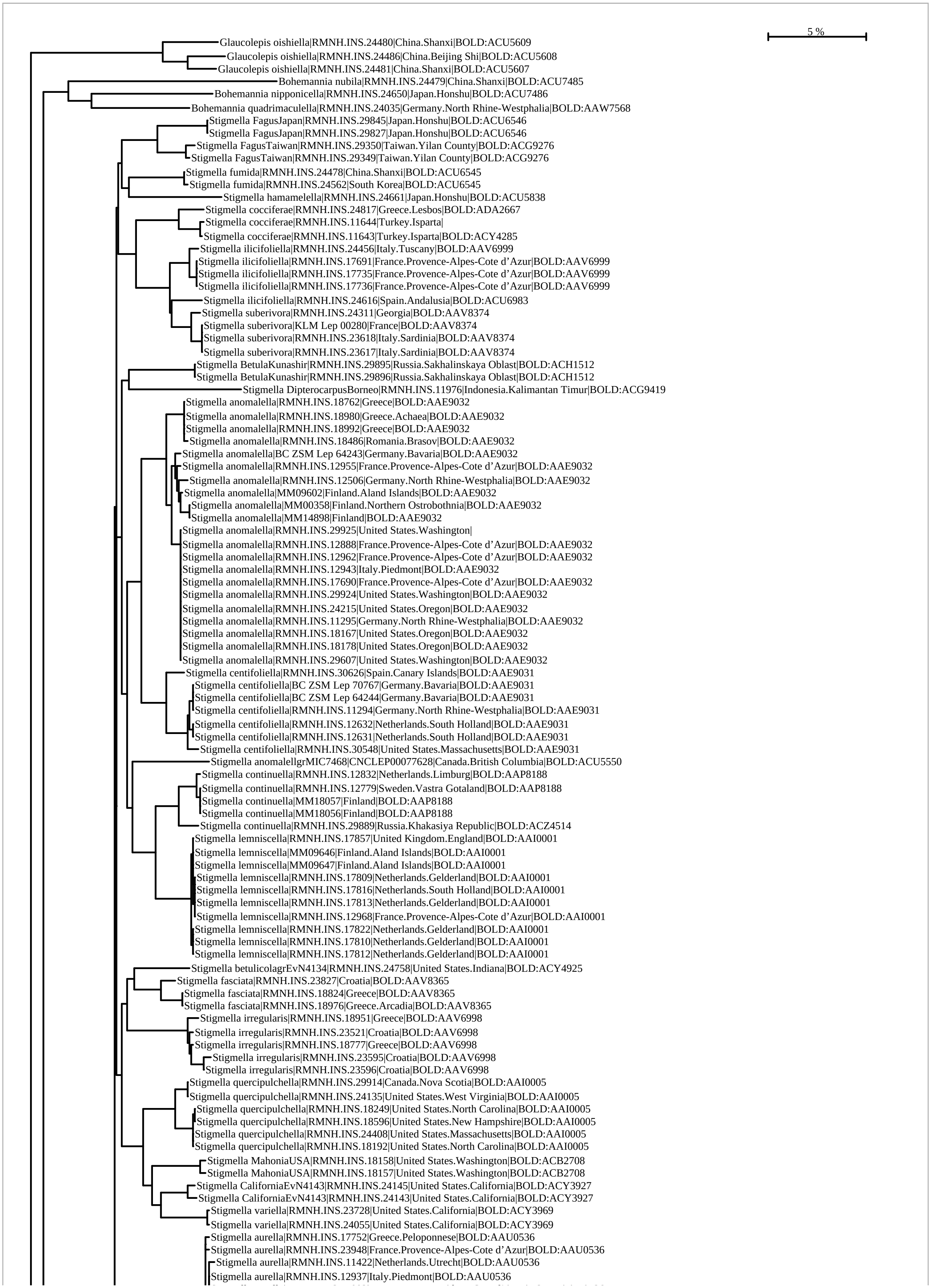

5 %
Glaucolepis oishiella|RMNH.INS.24480|China.Shanxi|BOLD:ACU5609
Glaucolepis oishiella|RMNH.INS.24486|China.Beijing Shi|BOLD:ACU5608
Glaucolepis oishiella|RMNH.INS.24481|China.Shanxi|BOLD:ACU5607
Bohemannia nubila|RMNH.INS.24479|China.Shanxi|BOLD:ACU7485
Bohemannia nipponicella|RMNH.INS.24650|Japan.Honshu|BOLD:ACU7486
Bohemannia quadrimaculella|RMNH.INS.24035|Germany.North Rhine-Westphalia|BOLD:AAW7568
Stigmella FagusJapan|RMNH.INS.29845|Japan.Honshu|BOLD:ACU6546
Stigmella FagusJapan|RMNH.INS.29827|Japan.Honshu|BOLD:ACU6546
Stigmella FagusTaiwan|RMNH.INS.29350|Taiwan.Yilan County|BOLD:ACG9276
Stigmella FagusTaiwan|RMNH.INS.29349|Taiwan.Yilan County|BOLD:ACG9276
Stigmella fumida|RMNH.INS.24478|China.Shanxi|BOLD:ACU6545
Stigmella fumida|RMNH.INS.24562|South Korea|BOLD:ACU6545
Stigmella hamamelella|RMNH.INS.24661|Japan.Honshu|BOLD:ACU5838
Stigmella cocciferae|RMNH.INS.24817|Greece.Lesbos|BOLD:ADA2667
Stigmella cocciferae|RMNH.INS.11644|Turkey.Isparta|
Stigmella cocciferae|RMNH.INS.11643|Turkey.Isparta|BOLD:ACY4285
Stigmella ilicifoliella|RMNH.INS.24456|Italy.Tuscany|BOLD:AAV6999
Stigmella ilicifoliella|RMNH.INS.17691|France.Provence-Alpes-Cote d'Azur|BOLD:AAV6999
Stigmella ilicifoliella|RMNH.INS.17735|France.Provence-Alpes-Cote d'Azur|BOLD:AAV6999
Stigmella ilicifoliella|RMNH.INS.17736|France.Provence-Alpes-Cote d'Azur|BOLD:AAV6999
Stigmella ilicifoliella|RMNH.INS.24616|Spain.Andalusia|BOLD:ACU6983
Stigmella suberivora|RMNH.INS.24311|Georgia|BOLD:AAV8374
Stigmella suberivora|KLM Lep 00280|France|BOLD:AAV8374
Stigmella suberivora|RMNH.INS.23618|Italy.Sardinia|BOLD:AAV8374
Stigmella suberivora|RMNH.INS.23617|Italy.Sardinia|BOLD:AAV8374
Stigmella BetulaKunashir|RMNH.INS.29895|Russia.Sakhalinskaya Oblast|BOLD:ACH1512
Stigmella BetulaKunashir|RMNH.INS.29896|Russia.Sakhalinskaya Oblast|BOLD:ACH1512
Stigmella DipterocarpusBorneo|RMNH.INS.11976|Indonesia.Kalimantan Timur|BOLD:ACG9419
Stigmella anomalella|RMNH.INS.18762|Greece|BOLD:AAE9032
Stigmella anomalella|RMNH.INS.18980|Greece.Achaea|BOLD:AAE9032
Stigmella anomalella|RMNH.INS.18992|Greece|BOLD:AAE9032
Stigmella anomalella|RMNH.INS.18486|Romania.Brasov|BOLD:AAE9032
Stigmella anomalella|BC ZSM Lep 64243|Germany.Bavaria|BOLD:AAE9032
Stigmella anomalella|RMNH.INS.12955|France.Provence-Alpes-Cote d'Azur|BOLD:AAE9032
Stigmella anomalella|RMNH.INS.12506|Germany.North Rhine-Westphalia|BOLD:AAE9032
Stigmella anomalella|MM09602|Finland.Aland Islands|BOLD:AAE9032
Stigmella anomalella|MM00358|Finland.Northern Ostrobothnia|BOLD:AAE9032
Stigmella anomalella|MM14898|Finland|BOLD:AAE9032
Stigmella anomalella|RMNH.INS.29925|United States.Washington|
Stigmella anomalella|RMNH.INS.12888|France.Provence-Alpes-Cote d'Azur|BOLD:AAE9032
Stigmella anomalella|RMNH.INS.12962|France.Provence-Alpes-Cote d'Azur|BOLD:AAE9032
Stigmella anomalella|RMNH.INS.12943|Italy.Piedmont|BOLD:AAE9032
Stigmella anomalella|RMNH.INS.17690|France.Provence-Alpes-Cote d'Azur|BOLD:AAE9032
Stigmella anomalella|RMNH.INS.29924|United States.Washington|BOLD:AAE9032
Stigmella anomalella|RMNH.INS.24215|United States.Oregon|BOLD:AAE9032
Stigmella anomalella|RMNH.INS.11295|Germany.North Rhine-Westphalia|BOLD:AAE9032
Stigmella anomalella|RMNH.INS.18167|United States.Oregon|BOLD:AAE9032
Stigmella anomalella|RMNH.INS.18178|United States.Oregon|BOLD:AAE9032
Stigmella anomalella|RMNH.INS.29607|United States.Washington|BOLD:AAE9032
Stigmella centifoliella|RMNH.INS.30626|Spain.Canary Islands|BOLD:AAE9031
Stigmella centifoliella|BC ZSM Lep 70767|Germany.Bavaria|BOLD:AAE9031
Stigmella centifoliella|BC ZSM Lep 64244|Germany.Bavaria|BOLD:AAE9031
Stigmella centifoliella|RMNH.INS.11294|Germany.North Rhine-Westphalia|BOLD:AAE9031
Stigmella centifoliella|RMNH.INS.12632|Netherlands.South Holland|BOLD:AAE9031
Stigmella centifoliella|RMNH.INS.12631|Netherlands.South Holland|BOLD:AAE9031
Stigmella centifoliella|RMNH.INS.30548|United States.Massachusetts|BOLD:AAE9031
Stigmella anomalellgrMIC7468|CNCLEP00077628|Canada.British Columbia|BOLD:ACU5550
Stigmella continuella|RMNH.INS.12832|Netherlands.Limburg|BOLD:AAP8188
Stigmella continuella|RMNH.INS.12779|Sweden.Vastra Gotaland|BOLD:AAP8188
Stigmella continuella|MM18057|Finland|BOLD:AAP8188
Stigmella continuella|MM18056|Finland|BOLD:AAP8188
Stigmella continuella|RMNH.INS.29889|Russia.Khakasiya Republic|BOLD:ACZ4514
Stigmella lemniscella|RMNH.INS.17857|United Kingdom.England|BOLD:AAI0001
Stigmella lemniscella|MM09646|Finland.Aland Islands|BOLD:AAI0001
Stigmella lemniscella|MM09647|Finland.Aland Islands|BOLD:AAI0001
Stigmella lemniscella|RMNH.INS.17809|Netherlands.Gelderland|BOLD:AAI0001
Stigmella lemniscella|RMNH.INS.17816|Netherlands.South Holland|BOLD:AAI0001
Stigmella lemniscella|RMNH.INS.17813|Netherlands.Gelderland|BOLD:AAI0001
Stigmella lemniscella|RMNH.INS.12968|France.Provence-Alpes-Cote d'Azur|BOLD:AAI0001
Stigmella lemniscella|RMNH.INS.17822|Netherlands.Gelderland|BOLD:AAI0001
Stigmella lemniscella|RMNH.INS.17810|Netherlands.Gelderland|BOLD:AAI0001
Stigmella lemniscella|RMNH.INS.17812|Netherlands.Gelderland|BOLD:AAI0001
Stigmella betulicolagrEvN4134|RMNH.INS.24758|United States.Indiana|BOLD:ACY4925
Stigmella fasciata|RMNH.INS.23827|Croatia|BOLD:AAV8365
Stigmella fasciata|RMNH.INS.18824|Greece|BOLD:AAV8365
Stigmella fasciata|RMNH.INS.18976|Greece.Arcadia|BOLD:AAV8365
Stigmella irregularis|RMNH.INS.18951|Greece|BOLD:AAV6998
Stigmella irregularis|RMNH.INS.23521|Croatia|BOLD:AAV6998
Stigmella irregularis|RMNH.INS.18777|Greece|BOLD:AAV6998
Stigmella irregularis|RMNH.INS.23595|Croatia|BOLD:AAV6998
Stigmella irregularis|RMNH.INS.23596|Croatia|BOLD:AAV6998
Stigmella quercipulchella|RMNH.INS.29914|Canada.Nova Scotia|BOLD:AAI0005
Stigmella quercipulchella|RMNH.INS.24135|United States.West Virginia|BOLD:AAI0005
Stigmella quercipulchella|RMNH.INS.18249|United States.North Carolina|BOLD:AAI0005
Stigmella quercipulchella|RMNH.INS.18596|United States.New Hampshire|BOLD:AAI0005
Stigmella quercipulchella|RMNH.INS.24408|United States.Massachusetts|BOLD:AAI0005
Stigmella quercipulchella|RMNH.INS.18192|United States.North Carolina|BOLD:AAI0005
Stigmella MahoniaUSA|RMNH.INS.18158|United States.Washington|BOLD:ACB2708
Stigmella MahoniaUSA|RMNH.INS.18157|United States.Washington|BOLD:ACB2708
Stigmella CaliforniaEvN4143|RMNH.INS.24145|United States.California|BOLD:ACY3927
Stigmella CaliforniaEvN4143|RMNH.INS.24143|United States.California|BOLD:ACY3927
Stigmella variella|RMNH.INS.23728|United States.California|BOLD:ACY3969
Stigmella variella|RMNH.INS.24055|United States.California|BOLD:ACY3969
Stigmella aurella|RMNH.INS.17752|Greece.Peloponnese|BOLD:AAU0536
Stigmella aurella|RMNH.INS.23948|France.Provence-Alpes-Cote d'Azur|BOLD:AAU0536
Stigmella aurella|RMNH.INS.11422|Netherlands.Utrecht|BOLD:AAU0536
Stigmella aurella|RMNH.INS.12937|Italy.Piedmont|BOLD:AAU0536

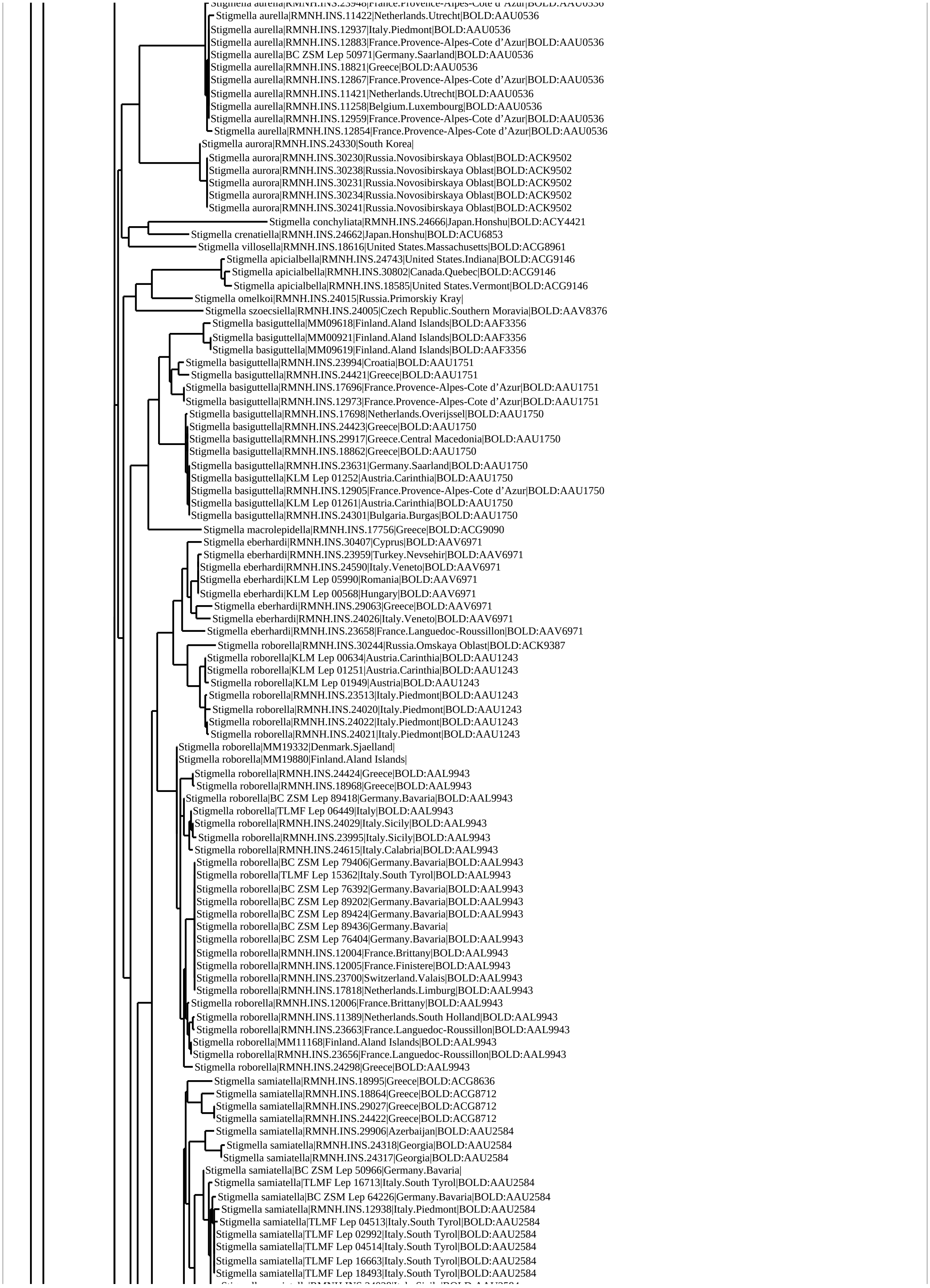
Stigmella aurella|RMNH.INS.23948|France.Provence-Alpes-Cote d'Azur|BOLD:AAU0536
Stigmella aurella|RMNH.INS.11422|Netherlands.Utrecht|BOLD:AAU0536
Stigmella aurella|RMNH.INS.12937|Italy.Piedmont|BOLD:AAU0536
Stigmella aurella|RMNH.INS.12883|France.Provence-Alpes-Cote d'Azur|BOLD:AAU0536
Stigmella aurella|BC ZSM Lep 50971|Germany.Saarland|BOLD:AAU0536
Stigmella aurella|RMNH.INS.18821|Greece|BOLD:AAU0536
Stigmella aurella|RMNH.INS.12867|France.Provence-Alpes-Cote d'Azur|BOLD:AAU0536
Stigmella aurella|RMNH.INS.11421|Netherlands.Utrecht|BOLD:AAU0536
Stigmella aurella|RMNH.INS.11258|Belgium.Luxembourg|BOLD:AAU0536
Stigmella aurella|RMNH.INS.12959|France.Provence-Alpes-Cote d'Azur|BOLD:AAU0536
Stigmella aurella|RMNH.INS.12854|France.Provence-Alpes-Cote d'Azur|BOLD:AAU0536
Stigmella aurora|RMNH.INS.24330|South Korea|
Stigmella aurora|RMNH.INS.30230|Russia.Novosibirskaya Oblast|BOLD:ACK9502
Stigmella aurora|RMNH.INS.30238|Russia.Novosibirskaya Oblast|BOLD:ACK9502
Stigmella aurora|RMNH.INS.30231|Russia.Novosibirskaya Oblast|BOLD:ACK9502
Stigmella aurora|RMNH.INS.30234|Russia.Novosibirskaya Oblast|BOLD:ACK9502
Stigmella aurora|RMNH.INS.30241|Russia.Novosibirskaya Oblast|BOLD:ACK9502
Stigmella conchyliata|RMNH.INS.24666|Japan.Honshu|BOLD:ACY4421
Stigmella crenatiella|RMNH.INS.24662|Japan.Honshu|BOLD:ACU6853
Stigmella villosella|RMNH.INS.18616|United States.Massachusetts|BOLD:ACG8961
Stigmella apicialbella|RMNH.INS.24743|United States.Indiana|BOLD:ACG9146
Stigmella apicialbella|RMNH.INS.30802|Canada.Quebec|BOLD:ACG9146
Stigmella apicialbella|RMNH.INS.18585|United States.Vermont|BOLD:ACG9146
Stigmella omelkoi|RMNH.INS.24015|Russia.Primorskiy Kray|
Stigmella szoecsiella|RMNH.INS.24005|Czech Republic.Southern Moravia|BOLD:AAV8376
Stigmella basiguttella|MM09618|Finland.Aland Islands|BOLD:AAF3356
Stigmella basiguttella|MM00921|Finland.Aland Islands|BOLD:AAF3356
Stigmella basiguttella|MM09619|Finland.Aland Islands|BOLD:AAF3356
Stigmella basiguttella|RMNH.INS.23994|Croatia|BOLD:AAU1751
Stigmella basiguttella|RMNH.INS.24421|Greece|BOLD:AAU1751
Stigmella basiguttella|RMNH.INS.17696|France.Provence-Alpes-Cote d'Azur|BOLD:AAU1751
Stigmella basiguttella|RMNH.INS.12973|France.Provence-Alpes-Cote d'Azur|BOLD:AAU1751
Stigmella basiguttella|RMNH.INS.17698|Netherlands.Overijssel|BOLD:AAU1750
Stigmella basiguttella|RMNH.INS.24423|Greece|BOLD:AAU1750
Stigmella basiguttella|RMNH.INS.29917|Greece.Central Macedonia|BOLD:AAU1750
Stigmella basiguttella|RMNH.INS.18862|Greece|BOLD:AAU1750
Stigmella basiguttella|RMNH.INS.23631|Germany.Saarland|BOLD:AAU1750
Stigmella basiguttella|KLM Lep 01252|Austria.Carinthia|BOLD:AAU1750
Stigmella basiguttella|RMNH.INS.12905|France.Provence-Alpes-Cote d'Azur|BOLD:AAU1750
Stigmella basiguttella|KLM Lep 01261|Austria.Carinthia|BOLD:AAU1750
Stigmella basiguttella|RMNH.INS.24301|Bulgaria.Burgas|BOLD:AAU1750
Stigmella macrolepidella|RMNH.INS.17756|Greece|BOLD:ACG9090
Stigmella eberhardi|RMNH.INS.30407|Cyprus|BOLD:AAV6971
Stigmella eberhardi|RMNH.INS.23959|Turkey.Nevsehir|BOLD:AAV6971
Stigmella eberhardi|RMNH.INS.24590|Italy.Veneto|BOLD:AAV6971
Stigmella eberhardi|KLM Lep 05990|Romania|BOLD:AAV6971
Stigmella eberhardi|KLM Lep 00568|Hungary|BOLD:AAV6971
Stigmella eberhardi|RMNH.INS.29063|Greece|BOLD:AAV6971
Stigmella eberhardi|RMNH.INS.24026|Italy.Veneto|BOLD:AAV6971
Stigmella eberhardi|RMNH.INS.23658|France.Languedoc-Roussillon|BOLD:AAV6971
Stigmella roborella|RMNH.INS.30244|Russia.Omskaya Oblast|BOLD:ACK9387
Stigmella roborella|KLM Lep 00634|Austria.Carinthia|BOLD:AAU1243
Stigmella roborella|KLM Lep 01251|Austria.Carinthia|BOLD:AAU1243
Stigmella roborella|KLM Lep 01949|Austria|BOLD:AAU1243
Stigmella roborella|RMNH.INS.23513|Italy.Piedmont|BOLD:AAU1243
Stigmella roborella|RMNH.INS.24020|Italy.Piedmont|BOLD:AAU1243
Stigmella roborella|RMNH.INS.24022|Italy.Piedmont|BOLD:AAU1243
Stigmella roborella|RMNH.INS.24021|Italy.Piedmont|BOLD:AAU1243
Stigmella roborella|MM19332|Denmark.Sjaelland|
Stigmella roborella|MM19880|Finland.Aland Islands|
Stigmella roborella|RMNH.INS.24424|Greece|BOLD:AAL9943
Stigmella roborella|RMNH.INS.18968|Greece|BOLD:AAL9943
Stigmella roborella|BC ZSM Lep 89418|Germany.Bavaria|BOLD:AAL9943
Stigmella roborella|TLMF Lep 06449|Italy|BOLD:AAL9943
Stigmella roborella|RMNH.INS.24029|Italy.Sicily|BOLD:AAL9943
Stigmella roborella|RMNH.INS.23995|Italy.Sicily|BOLD:AAL9943
Stigmella roborella|RMNH.INS.24615|Italy.Calabria|BOLD:AAL9943
Stigmella roborella|BC ZSM Lep 79406|Germany.Bavaria|BOLD:AAL9943
Stigmella roborella|TLMF Lep 15362|Italy.South Tyrol|BOLD:AAL9943
Stigmella roborella|BC ZSM Lep 76392|Germany.Bavaria|BOLD:AAL9943
Stigmella roborella|BC ZSM Lep 89202|Germany.Bavaria|BOLD:AAL9943
Stigmella roborella|BC ZSM Lep 89424|Germany.Bavaria|BOLD:AAL9943
Stigmella roborella|BC ZSM Lep 89436|Germany.Bavaria|
Stigmella roborella|BC ZSM Lep 76404|Germany.Bavaria|BOLD:AAL9943
Stigmella roborella|RMNH.INS.12004|France.Brittany|BOLD:AAL9943
Stigmella roborella|RMNH.INS.12005|France.Finistere|BOLD:AAL9943
Stigmella roborella|RMNH.INS.23700|Switzerland.Valais|BOLD:AAL9943
Stigmella roborella|RMNH.INS.17818|Netherlands.Limburg|BOLD:AAL9943
Stigmella roborella|RMNH.INS.12006|France.Brittany|BOLD:AAL9943
Stigmella roborella|RMNH.INS.11389|Netherlands.South Holland|BOLD:AAL9943
Stigmella roborella|RMNH.INS.23663|France.Languedoc-Roussillon|BOLD:AAL9943
Stigmella roborella|MM11168|Finland.Aland Islands|BOLD:AAL9943
Stigmella roborella|RMNH.INS.23656|France.Languedoc-Roussillon|BOLD:AAL9943
Stigmella roborella|RMNH.INS.24298|Greece|BOLD:AAL9943
Stigmella samiatella|RMNH.INS.18995|Greece|BOLD:ACG8636
Stigmella samiatella|RMNH.INS.18864|Greece|BOLD:ACG8712
Stigmella samiatella|RMNH.INS.29027|Greece|BOLD:ACG8712
Stigmella samiatella|RMNH.INS.24422|Greece|BOLD:ACG8712
Stigmella samiatella|RMNH.INS.29906|Azerbaijan|BOLD:AAU2584
Stigmella samiatella|RMNH.INS.24318|Georgia|BOLD:AAU2584
Stigmella samiatella|RMNH.INS.24317|Georgia|BOLD:AAU2584
Stigmella samiatella|BC ZSM Lep 50966|Germany.Bavaria|
Stigmella samiatella|TLMF Lep 16713|Italy.South Tyrol|BOLD:AAU2584
Stigmella samiatella|BC ZSM Lep 64226|Germany.Bavaria|BOLD:AAU2584
Stigmella samiatella|RMNH.INS.12938|Italy.Piedmont|BOLD:AAU2584
Stigmella samiatella|TLMF Lep 04513|Italy.South Tyrol|BOLD:AAU2584
Stigmella samiatella|TLMF Lep 02992|Italy.South Tyrol|BOLD:AAU2584
Stigmella samiatella|TLMF Lep 04514|Italy.South Tyrol|BOLD:AAU2584
Stigmella samiatella|TLMF Lep 16663|Italy.South Tyrol|BOLD:AAU2584
Stigmella samiatella|TLMF Lep 18493|Italy.South Tyrol|BOLD:AAU2584

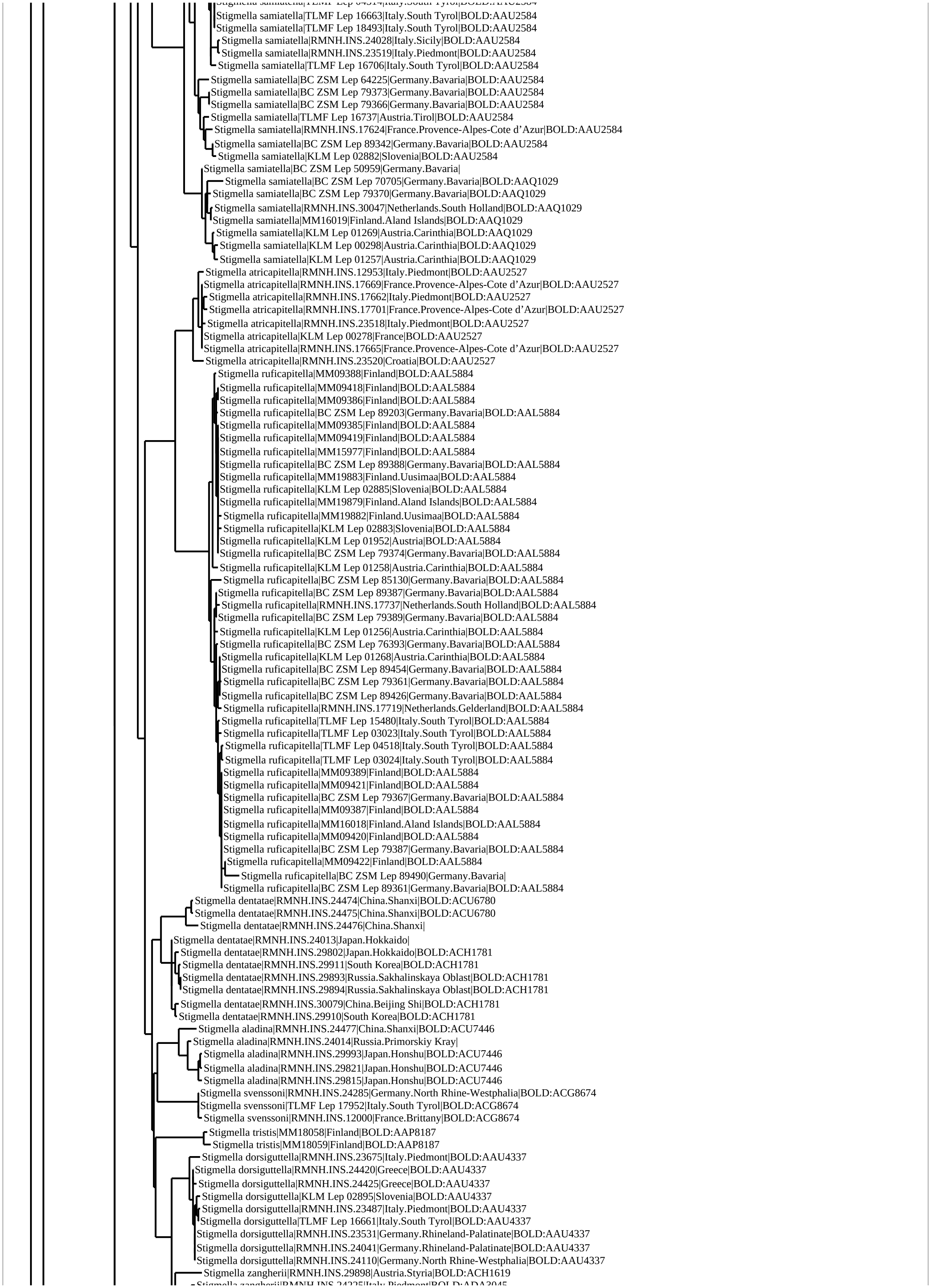
Stigmella samiatella|TLMF Lep 04514|Italy.South Tyrol|BOLD:AAU2584
Stigmella samiatella|TLMF Lep 16663|Italy.South Tyrol|BOLD:AAU2584
Stigmella samiatella|TLMF Lep 18493|Italy.South Tyrol|BOLD:AAU2584
Stigmella samiatella|RMNH.INS.24028|Italy.Sicily|BOLD:AAU2584
Stigmella samiatella|RMNH.INS.23519|Italy.Piedmont|BOLD:AAU2584
Stigmella samiatella|TLMF Lep 16706|Italy.South Tyrol|BOLD:AAU2584
Stigmella samiatella|BC ZSM Lep 64225|Germany.Bavaria|BOLD:AAU2584
Stigmella samiatella|BC ZSM Lep 79373|Germany.Bavaria|BOLD:AAU2584
Stigmella samiatella|BC ZSM Lep 79366|Germany.Bavaria|BOLD:AAU2584
Stigmella samiatella|TLMF Lep 16737|Austria.Tirol|BOLD:AAU2584
Stigmella samiatella|RMNH.INS.17624|France.Provence-Alpes-Cote d'Azur|BOLD:AAU2584
Stigmella samiatella|BC ZSM Lep 89342|Germany.Bavaria|BOLD:AAU2584
Stigmella samiatella|KLM Lep 02882|Slovenia|BOLD:AAU2584
Stigmella samiatella|BC ZSM Lep 50959|Germany.Bavaria|
Stigmella samiatella|BC ZSM Lep 70705|Germany.Bavaria|BOLD:AAQ1029
Stigmella samiatella|BC ZSM Lep 79370|Germany.Bavaria|BOLD:AAQ1029
Stigmella samiatella|RMNH.INS.30047|Netherlands.South Holland|BOLD:AAQ1029
Stigmella samiatella|MM16019|Finland.Aland Islands|BOLD:AAQ1029
Stigmella samiatella|KLM Lep 01269|Austria.Carinthia|BOLD:AAQ1029
Stigmella samiatella|KLM Lep 00298|Austria.Carinthia|BOLD:AAQ1029
Stigmella samiatella|KLM Lep 01257|Austria.Carinthia|BOLD:AAQ1029
Stigmella atricapitella|RMNH.INS.12953|Italy.Piedmont|BOLD:AAU2527
Stigmella atricapitella|RMNH.INS.17669|France.Provence-Alpes-Cote d'Azur|BOLD:AAU2527
Stigmella atricapitella|RMNH.INS.17662|Italy.Piedmont|BOLD:AAU2527
Stigmella atricapitella|RMNH.INS.17701|France.Provence-Alpes-Cote d'Azur|BOLD:AAU2527
Stigmella atricapitella|RMNH.INS.23518|Italy.Piedmont|BOLD:AAU2527
Stigmella atricapitella|KLM Lep 00278|France|BOLD:AAU2527
Stigmella atricapitella|RMNH.INS.17665|France.Provence-Alpes-Cote d'Azur|BOLD:AAU2527
Stigmella atricapitella|RMNH.INS.23520|Croatia|BOLD:AAU2527
Stigmella ruficapitella|MM09388|Finland|BOLD:AAL5884
Stigmella ruficapitella|MM09418|Finland|BOLD:AAL5884
Stigmella ruficapitella|MM09386|Finland|BOLD:AAL5884
Stigmella ruficapitella|BC ZSM Lep 89203|Germany.Bavaria|BOLD:AAL5884
Stigmella ruficapitella|MM09385|Finland|BOLD:AAL5884
Stigmella ruficapitella|MM09419|Finland|BOLD:AAL5884
Stigmella ruficapitella|MM15977|Finland|BOLD:AAL5884
Stigmella ruficapitella|BC ZSM Lep 89388|Germany.Bavaria|BOLD:AAL5884
Stigmella ruficapitella|MM19883|Finland.Uusimaa|BOLD:AAL5884
Stigmella ruficapitella|KLM Lep 02885|Slovenia|BOLD:AAL5884
Stigmella ruficapitella|MM19879|Finland.Aland Islands|BOLD:AAL5884
Stigmella ruficapitella|MM19882|Finland.Uusimaa|BOLD:AAL5884
Stigmella ruficapitella|KLM Lep 02883|Slovenia|BOLD:AAL5884
Stigmella ruficapitella|KLM Lep 01952|Austria|BOLD:AAL5884
Stigmella ruficapitella|BC ZSM Lep 79374|Germany.Bavaria|BOLD:AAL5884
Stigmella ruficapitella|KLM Lep 01258|Austria.Carinthia|BOLD:AAL5884
Stigmella ruficapitella|BC ZSM Lep 85130|Germany.Bavaria|BOLD:AAL5884
Stigmella ruficapitella|BC ZSM Lep 89387|Germany.Bavaria|BOLD:AAL5884
Stigmella ruficapitella|RMNH.INS.17737|Netherlands.South Holland|BOLD:AAL5884
Stigmella ruficapitella|BC ZSM Lep 79389|Germany.Bavaria|BOLD:AAL5884
Stigmella ruficapitella|KLM Lep 01256|Austria.Carinthia|BOLD:AAL5884
Stigmella ruficapitella|BC ZSM Lep 76393|Germany.Bavaria|BOLD:AAL5884
Stigmella ruficapitella|KLM Lep 01268|Austria.Carinthia|BOLD:AAL5884
Stigmella ruficapitella|BC ZSM Lep 89454|Germany.Bavaria|BOLD:AAL5884
Stigmella ruficapitella|BC ZSM Lep 79361|Germany.Bavaria|BOLD:AAL5884
Stigmella ruficapitella|BC ZSM Lep 89426|Germany.Bavaria|BOLD:AAL5884
Stigmella ruficapitella|RMNH.INS.17719|Netherlands.Gelderland|BOLD:AAL5884
Stigmella ruficapitella|TLMF Lep 15480|Italy.South Tyrol|BOLD:AAL5884
Stigmella ruficapitella|TLMF Lep 03023|Italy.South Tyrol|BOLD:AAL5884
Stigmella ruficapitella|TLMF Lep 04518|Italy.South Tyrol|BOLD:AAL5884
Stigmella ruficapitella|TLMF Lep 03024|Italy.South Tyrol|BOLD:AAL5884
Stigmella ruficapitella|MM09389|Finland|BOLD:AAL5884
Stigmella ruficapitella|MM09421|Finland|BOLD:AAL5884
Stigmella ruficapitella|BC ZSM Lep 79367|Germany.Bavaria|BOLD:AAL5884
Stigmella ruficapitella|MM09387|Finland|BOLD:AAL5884
Stigmella ruficapitella|MM16018|Finland.Aland Islands|BOLD:AAL5884
Stigmella ruficapitella|MM09420|Finland|BOLD:AAL5884
Stigmella ruficapitella|BC ZSM Lep 79387|Germany.Bavaria|BOLD:AAL5884
Stigmella ruficapitella|MM09422|Finland|BOLD:AAL5884
Stigmella ruficapitella|BC ZSM Lep 89490|Germany.Bavaria|
Stigmella ruficapitella|BC ZSM Lep 89361|Germany.Bavaria|BOLD:AAL5884
Stigmella dentatae|RMNH.INS.24474|China.Shanxi|BOLD:ACU6780
Stigmella dentatae|RMNH.INS.24475|China.Shanxi|BOLD:ACU6780
Stigmella dentatae|RMNH.INS.24476|China.Shanxi|
Stigmella dentatae|RMNH.INS.24013|Japan.Hokkaido|
Stigmella dentatae|RMNH.INS.29802|Japan.Hokkaido|BOLD:ACH1781
Stigmella dentatae|RMNH.INS.29911|South Korea|BOLD:ACH1781
Stigmella dentatae|RMNH.INS.29893|Russia.Sakhalinskaya Oblast|BOLD:ACH1781
Stigmella dentatae|RMNH.INS.29894|Russia.Sakhalinskaya Oblast|BOLD:ACH1781
Stigmella dentatae|RMNH.INS.30079|China.Beijing Shi|BOLD:ACH1781
Stigmella dentatae|RMNH.INS.29910|South Korea|BOLD:ACH1781
Stigmella aladina|RMNH.INS.24477|China.Shanxi|BOLD:ACU7446
Stigmella aladina|RMNH.INS.24014|Russia.Primorskiy Kray|
Stigmella aladina|RMNH.INS.29993|Japan.Honshu|BOLD:ACU7446
Stigmella aladina|RMNH.INS.29821|Japan.Honshu|BOLD:ACU7446
Stigmella aladina|RMNH.INS.29815|Japan.Honshu|BOLD:ACU7446
Stigmella svenssoni|RMNH.INS.24285|Germany.North Rhine-Westphalia|BOLD:ACG8674
Stigmella svenssoni|TLMF Lep 17952|Italy.South Tyrol|BOLD:ACG8674
Stigmella svenssoni|RMNH.INS.12000|France.Brittany|BOLD:ACG8674
Stigmella tristis|MM18058|Finland|BOLD:AAP8187
Stigmella tristis|MM18059|Finland|BOLD:AAP8187
Stigmella dorsiguttella|RMNH.INS.23675|Italy.Piedmont|BOLD:AAU4337
Stigmella dorsiguttella|RMNH.INS.24420|Greece|BOLD:AAU4337
Stigmella dorsiguttella|RMNH.INS.24425|Greece|BOLD:AAU4337
Stigmella dorsiguttella|KLM Lep 02895|Slovenia|BOLD:AAU4337
Stigmella dorsiguttella|RMNH.INS.23487|Italy.Piedmont|BOLD:AAU4337
Stigmella dorsiguttella|TLMF Lep 16661|Italy.South Tyrol|BOLD:AAU4337
Stigmella dorsiguttella|RMNH.INS.23531|Germany.Rhineland-Palatinate|BOLD:AAU4337
Stigmella dorsiguttella|RMNH.INS.24041|Germany.Rhineland-Palatinate|BOLD:AAU4337
Stigmella dorsiguttella|RMNH.INS.24110|Germany.North Rhine-Westphalia|BOLD:AAU4337
Stigmella zangherii|RMNH.INS.29898|Austria.Styria|BOLD:ACH1619
Stigmella zangherii|RMNH.INS.24225|Italy.Piedmont|BOLD:ADA3045

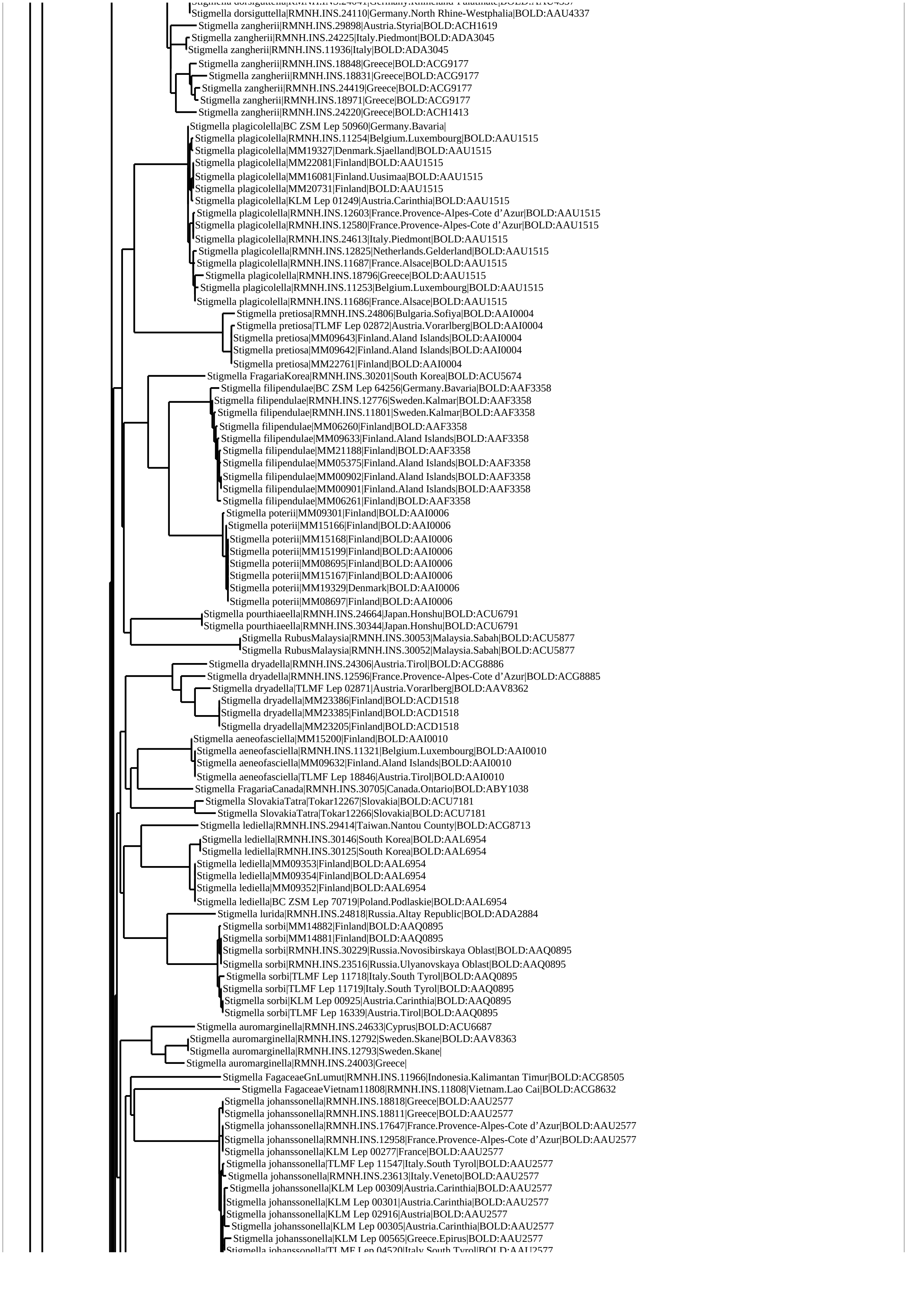
Stigmella dorsiguttella|RMNH.INS.24110|Germany.North Rhine-Westphalia|BOLD:AAU4337
Stigmella zangherii|RMNH.INS.29898|Austria.Styria|BOLD:ACH1619
Stigmella zangherii|RMNH.INS.24225|Italy.Piedmont|BOLD:ADA3045
Stigmella zangherii|RMNH.INS.11936|Italy|BOLD:ADA3045
Stigmella zangherii|RMNH.INS.18848|Greece|BOLD:ACG9177
Stigmella zangherii|RMNH.INS.18831|Greece|BOLD:ACG9177
Stigmella zangherii|RMNH.INS.24419|Greece|BOLD:ACG9177
Stigmella zangherii|RMNH.INS.18971|Greece|BOLD:ACG9177
Stigmella zangherii|RMNH.INS.24220|Greece|BOLD:ACH1413
Stigmella plagicolella|BC ZSM Lep 50960|Germany.Bavaria|
Stigmella plagicolella|RMNH.INS.11254|Belgium.Luxembourg|BOLD:AAU1515
Stigmella plagicolella|MM19327|Denmark.Sjaelland|BOLD:AAU1515
Stigmella plagicolella|MM22081|Finland|BOLD:AAU1515
Stigmella plagicolella|MM16081|Finland.Uusimaa|BOLD:AAU1515
Stigmella plagicolella|MM20731|Finland|BOLD:AAU1515
Stigmella plagicolella|KLM Lep 01249|Austria.Carinthia|BOLD:AAU1515
Stigmella plagicolella|RMNH.INS.12603|France.Provence-Alpes-Cote d'Azur|BOLD:AAU1515
Stigmella plagicolella|RMNH.INS.12580|France.Provence-Alpes-Cote d'Azur|BOLD:AAU1515
Stigmella plagicolella|RMNH.INS.24613|Italy.Piedmont|BOLD:AAU1515
Stigmella plagicolella|RMNH.INS.12825|Netherlands.Gelderland|BOLD:AAU1515
Stigmella plagicolella|RMNH.INS.11687|France.Alsace|BOLD:AAU1515
Stigmella plagicolella|RMNH.INS.18796|Greece|BOLD:AAU1515
Stigmella plagicolella|RMNH.INS.11253|Belgium.Luxembourg|BOLD:AAU1515
Stigmella plagicolella|RMNH.INS.11686|France.Alsace|BOLD:AAU1515
Stigmella pretiosa|RMNH.INS.24806|Bulgaria.Sofiya|BOLD:AAI0004
Stigmella pretiosa|TLMF Lep 02872|Austria.Vorarlberg|BOLD:AAI0004
Stigmella pretiosa|MM09643|Finland.Aland Islands|BOLD:AAI0004
Stigmella pretiosa|MM09642|Finland.Aland Islands|BOLD:AAI0004
Stigmella pretiosa|MM22761|Finland|BOLD:AAI0004
Stigmella FragariaKorea|RMNH.INS.30201|South Korea|BOLD:ACU5674
Stigmella filipendulae|BC ZSM Lep 64256|Germany.Bavaria|BOLD:AAF3358
Stigmella filipendulae|RMNH.INS.12776|Sweden.Kalmar|BOLD:AAF3358
Stigmella filipendulae|RMNH.INS.11801|Sweden.Kalmar|BOLD:AAF3358
Stigmella filipendulae|MM06260|Finland|BOLD:AAF3358
Stigmella filipendulae|MM09633|Finland.Aland Islands|BOLD:AAF3358
Stigmella filipendulae|MM21188|Finland|BOLD:AAF3358
Stigmella filipendulae|MM05375|Finland.Aland Islands|BOLD:AAF3358
Stigmella filipendulae|MM00902|Finland.Aland Islands|BOLD:AAF3358
Stigmella filipendulae|MM00901|Finland.Aland Islands|BOLD:AAF3358
Stigmella filipendulae|MM06261|Finland|BOLD:AAF3358
Stigmella poterii|MM09301|Finland|BOLD:AAI0006
Stigmella poterii|MM15166|Finland|BOLD:AAI0006
Stigmella poterii|MM15168|Finland|BOLD:AAI0006
Stigmella poterii|MM15199|Finland|BOLD:AAI0006
Stigmella poterii|MM08695|Finland|BOLD:AAI0006
Stigmella poterii|MM15167|Finland|BOLD:AAI0006
Stigmella poterii|MM19329|Denmark|BOLD:AAI0006
Stigmella poterii|MM08697|Finland|BOLD:AAI0006
Stigmella pourthiaeella|RMNH.INS.24664|Japan.Honshu|BOLD:ACU6791
Stigmella pourthiaeella|RMNH.INS.30344|Japan.Honshu|BOLD:ACU6791
Stigmella RubusMalaysia|RMNH.INS.30053|Malaysia.Sabah|BOLD:ACU5877
Stigmella RubusMalaysia|RMNH.INS.30052|Malaysia.Sabah|BOLD:ACU5877
Stigmella dryadella|RMNH.INS.24306|Austria.Tirol|BOLD:ACG8886
Stigmella dryadella|RMNH.INS.12596|France.Provence-Alpes-Cote d'Azur|BOLD:ACG8885
Stigmella dryadella|TLMF Lep 02871|Austria.Vorarlberg|BOLD:AAV8362
Stigmella dryadella|MM23386|Finland|BOLD:ACD1518
Stigmella dryadella|MM23385|Finland|BOLD:ACD1518
Stigmella dryadella|MM23205|Finland|BOLD:ACD1518
Stigmella aeneofasciella|MM15200|Finland|BOLD:AAI0010
Stigmella aeneofasciella|RMNH.INS.11321|Belgium.Luxembourg|BOLD:AAI0010
Stigmella aeneofasciella|MM09632|Finland.Aland Islands|BOLD:AAI0010
Stigmella aeneofasciella|TLMF Lep 18846|Austria.Tirol|BOLD:AAI0010
Stigmella FragariaCanada|RMNH.INS.30705|Canada.Ontario|BOLD:ABY1038
Stigmella SlovakiaTatra|Tokar12267|Slovakia|BOLD:ACU7181
Stigmella SlovakiaTatra|Tokar12266|Slovakia|BOLD:ACU7181
Stigmella lediella|RMNH.INS.29414|Taiwan.Nantou County|BOLD:ACG8713
Stigmella lediella|RMNH.INS.30146|South Korea|BOLD:AAL6954
Stigmella lediella|RMNH.INS.30125|South Korea|BOLD:AAL6954
Stigmella lediella|MM09353|Finland|BOLD:AAL6954
Stigmella lediella|MM09354|Finland|BOLD:AAL6954
Stigmella lediella|MM09352|Finland|BOLD:AAL6954
Stigmella lediella|BC ZSM Lep 70719|Poland.Podlaskie|BOLD:AAL6954
Stigmella lurida|RMNH.INS.24818|Russia.Altay Republic|BOLD:ADA2884
Stigmella sorbi|MM14882|Finland|BOLD:AAQ0895
Stigmella sorbi|MM14881|Finland|BOLD:AAQ0895
Stigmella sorbi|RMNH.INS.30229|Russia.Novosibirskaya Oblast|BOLD:AAQ0895
Stigmella sorbi|RMNH.INS.23516|Russia.Ulyanovskaya Oblast|BOLD:AAQ0895
Stigmella sorbi|TLMF Lep 11718|Italy.South Tyrol|BOLD:AAQ0895
Stigmella sorbi|TLMF Lep 11719|Italy.South Tyrol|BOLD:AAQ0895
Stigmella sorbi|KLM Lep 00925|Austria.Carinthia|BOLD:AAQ0895
Stigmella sorbi|TLMF Lep 16339|Austria.Tirol|BOLD:AAQ0895
Stigmella auromarginella|RMNH.INS.24633|Cyprus|BOLD:ACU6687
Stigmella auromarginella|RMNH.INS.12792|Sweden.Skane|BOLD:AAV8363
Stigmella auromarginella|RMNH.INS.12793|Sweden.Skane|
Stigmella auromarginella|RMNH.INS.24003|Greece|
Stigmella FagaceaeGnLumut|RMNH.INS.11966|Indonesia.Kalimantan Timur|BOLD:ACG8505
Stigmella FagaceaeVietnam11808|RMNH.INS.11808|Vietnam.Lao Cai|BOLD:ACG8632
Stigmella johanssonella|RMNH.INS.18818|Greece|BOLD:AAU2577
Stigmella johanssonella|RMNH.INS.18811|Greece|BOLD:AAU2577
Stigmella johanssonella|RMNH.INS.17647|France.Provence-Alpes-Cote d'Azur|BOLD:AAU2577
Stigmella johanssonella|RMNH.INS.12958|France.Provence-Alpes-Cote d'Azur|BOLD:AAU2577
Stigmella johanssonella|KLM Lep 00277|France|BOLD:AAU2577
Stigmella johanssonella|TLMF Lep 11547|Italy.South Tyrol|BOLD:AAU2577
Stigmella johanssonella|RMNH.INS.23613|Italy.Veneto|BOLD:AAU2577
Stigmella johanssonella|KLM Lep 00309|Austria.Carinthia|BOLD:AAU2577
Stigmella johanssonella|KLM Lep 00301|Austria.Carinthia|BOLD:AAU2577
Stigmella johanssonella|KLM Lep 02916|Austria|BOLD:AAU2577
Stigmella johanssonella|KLM Lep 00305|Austria.Carinthia|BOLD:AAU2577
Stigmella johanssonella|KLM Lep 00565|Greece.Epirus|BOLD:AAU2577
Stigmella johanssonella|TLMF Lep 04520|Italy.South Tyrol|BOLD:AAU2577

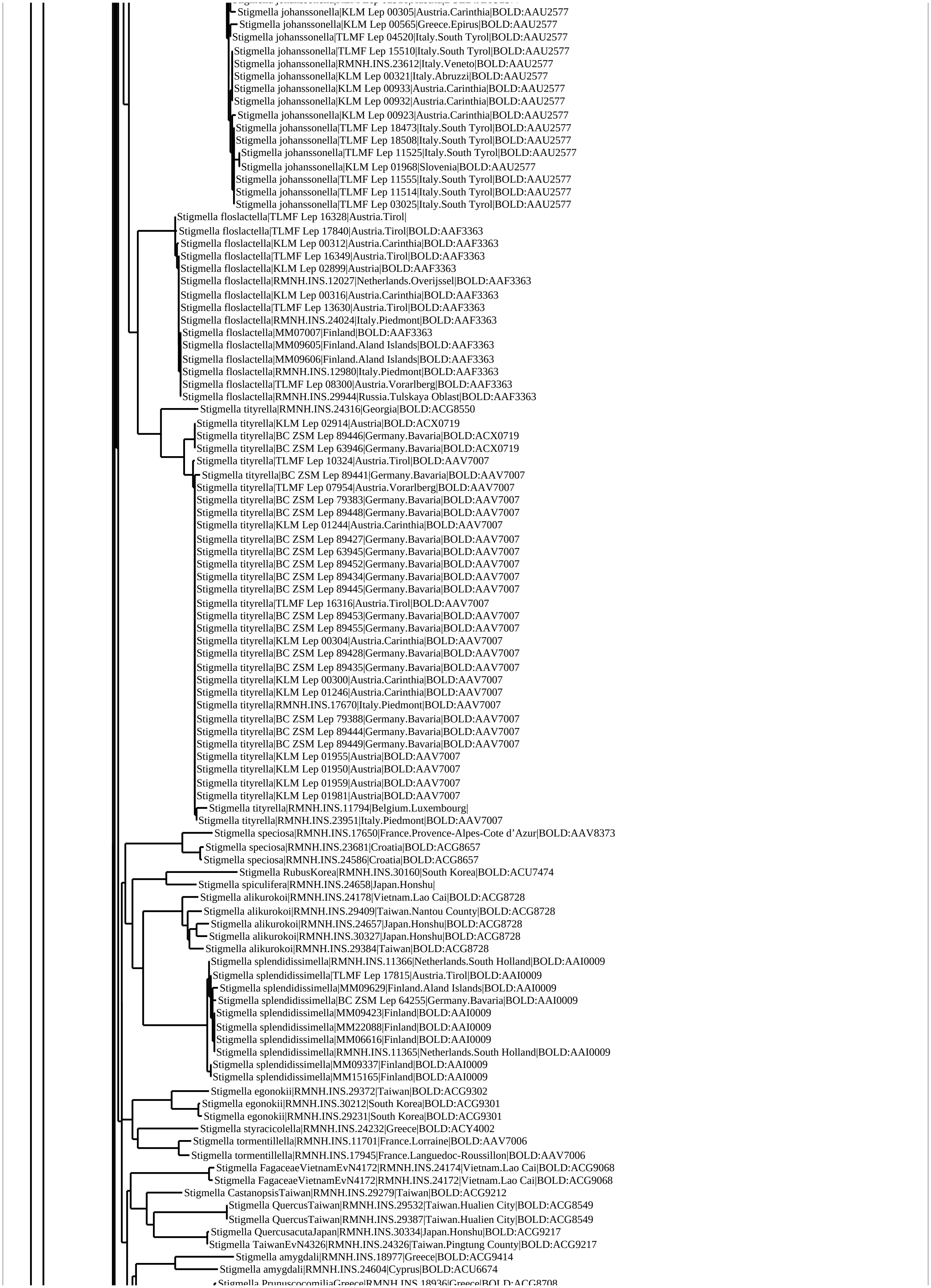
Stigmella johanssonella|KLM Lep 00305|Austria.Carinthia|BOLD:AAU2577
Stigmella johanssonella|KLM Lep 00565|Greece.Epirus|BOLD:AAU2577
Stigmella johanssonella|TLMF Lep 04520|Italy.South Tyrol|BOLD:AAU2577
Stigmella johanssonella|TLMF Lep 15510|Italy.South Tyrol|BOLD:AAU2577
Stigmella johanssonella|RMNH.INS.23612|Italy.Veneto|BOLD:AAU2577
Stigmella johanssonella|KLM Lep 00321|Italy.Abruzzi|BOLD:AAU2577
Stigmella johanssonella|KLM Lep 00933|Austria.Carinthia|BOLD:AAU2577
Stigmella johanssonella|KLM Lep 00932|Austria.Carinthia|BOLD:AAU2577
Stigmella johanssonella|KLM Lep 00923|Austria.Carinthia|BOLD:AAU2577
Stigmella johanssonella|TLMF Lep 18473|Italy.South Tyrol|BOLD:AAU2577
Stigmella johanssonella|TLMF Lep 18508|Italy.South Tyrol|BOLD:AAU2577
Stigmella johanssonella|TLMF Lep 11525|Italy.South Tyrol|BOLD:AAU2577
Stigmella johanssonella|KLM Lep 01968|Slovenia|BOLD:AAU2577
Stigmella johanssonella|TLMF Lep 11555|Italy.South Tyrol|BOLD:AAU2577
Stigmella johanssonella|TLMF Lep 11514|Italy.South Tyrol|BOLD:AAU2577
Stigmella johanssonella|TLMF Lep 03025|Italy.South Tyrol|BOLD:AAU2577
Stigmella floslactella|TLMF Lep 16328|Austria.Tirol|
Stigmella floslactella|TLMF Lep 17840|Austria.Tirol|BOLD:AAF3363
Stigmella floslactella|KLM Lep 00312|Austria.Carinthia|BOLD:AAF3363
Stigmella floslactella|TLMF Lep 16349|Austria.Tirol|BOLD:AAF3363
Stigmella floslactella|KLM Lep 02899|Austria|BOLD:AAF3363
Stigmella floslactella|RMNH.INS.12027|Netherlands.Overijssel|BOLD:AAF3363
Stigmella floslactella|KLM Lep 00316|Austria.Carinthia|BOLD:AAF3363
Stigmella floslactella|TLMF Lep 13630|Austria.Tirol|BOLD:AAF3363
Stigmella floslactella|RMNH.INS.24024|Italy.Piedmont|BOLD:AAF3363
Stigmella floslactella|MM07007|Finland|BOLD:AAF3363
Stigmella floslactella|MM09605|Finland.Aland Islands|BOLD:AAF3363
Stigmella floslactella|MM09606|Finland.Aland Islands|BOLD:AAF3363
Stigmella floslactella|RMNH.INS.12980|Italy.Piedmont|BOLD:AAF3363
Stigmella floslactella|TLMF Lep 08300|Austria.Vorarlberg|BOLD:AAF3363
Stigmella floslactella|RMNH.INS.29944|Russia.Tulskaya Oblast|BOLD:AAF3363
Stigmella tityrella|RMNH.INS.24316|Georgia|BOLD:ACG8550
Stigmella tityrella|KLM Lep 02914|Austria|BOLD:ACX0719
Stigmella tityrella|BC ZSM Lep 89446|Germany.Bavaria|BOLD:ACX0719
Stigmella tityrella|BC ZSM Lep 63946|Germany.Bavaria|BOLD:ACX0719
Stigmella tityrella|TLMF Lep 10324|Austria.Tirol|BOLD:AAV7007
Stigmella tityrella|BC ZSM Lep 89441|Germany.Bavaria|BOLD:AAV7007
Stigmella tityrella|TLMF Lep 07954|Austria.Vorarlberg|BOLD:AAV7007
Stigmella tityrella|BC ZSM Lep 79383|Germany.Bavaria|BOLD:AAV7007
Stigmella tityrella|BC ZSM Lep 89448|Germany.Bavaria|BOLD:AAV7007
Stigmella tityrella|KLM Lep 01244|Austria.Carinthia|BOLD:AAV7007
Stigmella tityrella|BC ZSM Lep 89427|Germany.Bavaria|BOLD:AAV7007
Stigmella tityrella|BC ZSM Lep 63945|Germany.Bavaria|BOLD:AAV7007
Stigmella tityrella|BC ZSM Lep 89452|Germany.Bavaria|BOLD:AAV7007
Stigmella tityrella|BC ZSM Lep 89434|Germany.Bavaria|BOLD:AAV7007
Stigmella tityrella|BC ZSM Lep 89445|Germany.Bavaria|BOLD:AAV7007
Stigmella tityrella|TLMF Lep 16316|Austria.Tirol|BOLD:AAV7007
Stigmella tityrella|BC ZSM Lep 89453|Germany.Bavaria|BOLD:AAV7007
Stigmella tityrella|BC ZSM Lep 89455|Germany.Bavaria|BOLD:AAV7007
Stigmella tityrella|KLM Lep 00304|Austria.Carinthia|BOLD:AAV7007
Stigmella tityrella|BC ZSM Lep 89428|Germany.Bavaria|BOLD:AAV7007
Stigmella tityrella|BC ZSM Lep 89435|Germany.Bavaria|BOLD:AAV7007
Stigmella tityrella|KLM Lep 00300|Austria.Carinthia|BOLD:AAV7007
Stigmella tityrella|KLM Lep 01246|Austria.Carinthia|BOLD:AAV7007
Stigmella tityrella|RMNH.INS.17670|Italy.Piedmont|BOLD:AAV7007
Stigmella tityrella|BC ZSM Lep 79388|Germany.Bavaria|BOLD:AAV7007
Stigmella tityrella|BC ZSM Lep 89444|Germany.Bavaria|BOLD:AAV7007
Stigmella tityrella|BC ZSM Lep 89449|Germany.Bavaria|BOLD:AAV7007
Stigmella tityrella|KLM Lep 01955|Austria|BOLD:AAV7007
Stigmella tityrella|KLM Lep 01950|Austria|BOLD:AAV7007
Stigmella tityrella|KLM Lep 01959|Austria|BOLD:AAV7007
Stigmella tityrella|KLM Lep 01981|Austria|BOLD:AAV7007
Stigmella tityrella|RMNH.INS.11794|Belgium.Luxembourg|
Stigmella tityrella|RMNH.INS.23951|Italy.Piedmont|BOLD:AAV7007
Stigmella speciosa|RMNH.INS.17650|France.Provence-Alpes-Cote d'Azur|BOLD:AAV8373
Stigmella speciosa|RMNH.INS.23681|Croatia|BOLD:ACG8657
Stigmella speciosa|RMNH.INS.24586|Croatia|BOLD:ACG8657
Stigmella RubusKorea|RMNH.INS.30160|South Korea|BOLD:ACU7474
Stigmella spiculifera|RMNH.INS.24658|Japan.Honshu|
Stigmella alikurokoi|RMNH.INS.24178|Vietnam.Lao Cai|BOLD:ACG8728
Stigmella alikurokoi|RMNH.INS.29409|Taiwan.Nantou County|BOLD:ACG8728
Stigmella alikurokoi|RMNH.INS.24657|Japan.Honshu|BOLD:ACG8728
Stigmella alikurokoi|RMNH.INS.30327|Japan.Honshu|BOLD:ACG8728
Stigmella alikurokoi|RMNH.INS.29384|Taiwan|BOLD:ACG8728
Stigmella splendidissimella|RMNH.INS.11366|Netherlands.South Holland|BOLD:AAI0009
Stigmella splendidissimella|TLMF Lep 17815|Austria.Tirol|BOLD:AAI0009
Stigmella splendidissimella|MM09629|Finland.Aland Islands|BOLD:AAI0009
Stigmella splendidissimella|BC ZSM Lep 64255|Germany.Bavaria|BOLD:AAI0009
Stigmella splendidissimella|MM09423|Finland|BOLD:AAI0009
Stigmella splendidissimella|MM22088|Finland|BOLD:AAI0009
Stigmella splendidissimella|MM06616|Finland|BOLD:AAI0009
Stigmella splendidissimella|RMNH.INS.11365|Netherlands.South Holland|BOLD:AAI0009
Stigmella splendidissimella|MM09337|Finland|BOLD:AAI0009
Stigmella splendidissimella|MM15165|Finland|BOLD:AAI0009
Stigmella egonokii|RMNH.INS.29372|Taiwan|BOLD:ACG9302
Stigmella egonokii|RMNH.INS.30212|South Korea|BOLD:ACG9301
Stigmella egonokii|RMNH.INS.29231|South Korea|BOLD:ACG9301
Stigmella styracicolella|RMNH.INS.24232|Greece|BOLD:ACY4002
Stigmella tormentillella|RMNH.INS.11701|France.Lorraine|BOLD:AAV7006
Stigmella tormentillella|RMNH.INS.17945|France.Languedoc-Roussillon|BOLD:AAV7006
Stigmella FagaceaeVietnamEvN4172|RMNH.INS.24174|Vietnam.Lao Cai|BOLD:ACG9068
Stigmella FagaceaeVietnamEvN4172|RMNH.INS.24172|Vietnam.Lao Cai|BOLD:ACG9068
Stigmella CastanopsisTaiwan|RMNH.INS.29279|Taiwan|BOLD:ACG9212
Stigmella QuercusTaiwan|RMNH.INS.29532|Taiwan.Hualien City|BOLD:ACG8549
Stigmella QuercusTaiwan|RMNH.INS.29387|Taiwan.Hualien City|BOLD:ACG8549
Stigmella QuercusacutaJapan|RMNH.INS.30334|Japan.Honshu|BOLD:ACG9217
Stigmella TaiwanEvN4326|RMNH.INS.24326|Taiwan.Pingtung County|BOLD:ACG9217
Stigmella amygdali|RMNH.INS.18977|Greece|BOLD:ACG9414
Stigmella amygdali|RMNH.INS.24604|Cyprus|BOLD:ACU6674
Stigmella PrunuscocomiliaGreece|RMNH.INS.18936|Greece|BOLD:ACG8708

Stigmella amygdali|RMNH.INS.18977|Greece|BOLD:ACG9414
Stigmella amygdali|RMNH.INS.24604|Cyprus|BOLD:ACU6674
Stigmella PrunuscocomiliaGreece|RMNH.INS.18936|Greece|BOLD:ACG8708
Stigmella PrunuscocomiliaGreece|RMNH.INS.18972|Greece|BOLD:ACG8708
Stigmella zelkoviella|RMNH.INS.29552|Taiwan|BOLD:ACG9089
Stigmella ChinaEvN4473|RMNH.INS.24473|China.Yunnan|BOLD:ACU6708
Stigmella LyoniaTaiwan|RMNH.INS.29461|Taiwan|BOLD:ACG8771
Stigmella LyoniaTaiwan|RMNH.INS.29462|Taiwan|BOLD:ACG8771
Stigmella kao|RMNH.INS.29281|Taiwan|BOLD:ACG8647
Stigmella kao|RMNH.INS.29277|Taiwan|BOLD:ACG8647
Stigmella NepalEvN4113|RMNH.INS.24113|Nepal|BOLD:ACY4210
Stigmella CastanopsisVietnam|RMNH.INS.11805|Vietnam.Lao Cai|BOLD:ACG9305
Stigmella CastanopsisVietnam|RMNH.INS.24175|Vietnam.Lao Cai|BOLD:ACG9305
Stigmella hemargyrella|BC ZSM Lep 64202|Germany.Bavaria|BOLD:AAV6973
Stigmella hemargyrella|BC ZSM Lep 79380|Germany.Bavaria|BOLD:AAV6973
Stigmella hemargyrella|TLMF Lep 07492|Austria.Vorarlberg|BOLD:AAV6973
Stigmella hemargyrella|BC ZSM Lep 89442|Germany.Bavaria|BOLD:AAV6973
Stigmella hemargyrella|BC ZSM Lep 70702|Germany.Bavaria|BOLD:AAV6973
Stigmella hemargyrella|KLM Lep 01243|Austria.Carinthia|BOLD:AAV6973
Stigmella hemargyrella|BC ZSM Lep 89447|Germany.Bavaria|BOLD:AAV6973
Stigmella hemargyrella|BC ZSM Lep 64222|Germany.Bavaria|BOLD:AAV6973
Stigmella hemargyrella|KLM Lep 01255|Austria.Carinthia|BOLD:AAV6973
Stigmella hemargyrella|KLM Lep 01953|Austria|BOLD:AAV6973
Stigmella hemargyrella|KLM Lep 01951|Austria|BOLD:AAV6973
Stigmella hemargyrella|BC ZSM Lep 89443|Germany.Bavaria|BOLD:AAV6973
Stigmella hemargyrella|BC ZSM Lep 76434|Germany.Bavaria|BOLD:AAV6973
Stigmella hemargyrella|BC ZSM Lep 76389|Germany.Bavaria|BOLD:AAV6973
Stigmella hemargyrella|BC ZSM Lep 76451|Germany.Bavaria|BOLD:AAV6973
Stigmella hemargyrella|KLM Lep 01241|Austria.Carinthia|BOLD:AAV6973
Stigmella hemargyrella|KLM Lep 00928|Austria.Carinthia|BOLD:AAV6973
Stigmella hemargyrella|BC ZSM Lep 89340|Germany.Bavaria|BOLD:AAV6973
Stigmella hemargyrella|BC ZSM Lep 89432|Germany.Bavaria|BOLD:AAV6973
Stigmella hemargyrella|KLM Lep 02917|Austria|BOLD:AAV6973
Stigmella hemargyrella|KLM Lep 02915|Austria|BOLD:AAV6973
Stigmella hemargyrella|RMNH.INS.11430|Netherlands.Utrecht|BOLD:AAV6973
Stigmella hemargyrella|BC ZSM Lep 89431|Germany.Bavaria|BOLD:AAV6973
Stigmella hemargyrella|BC ZSM Lep 89450|Germany.Bavaria|BOLD:AAV6973
Stigmella hemargyrella|RMNH.INS.24585|Italy.Piedmont|BOLD:AAV6973
Stigmella hemargyrella|KLM Lep 00569|Hungary|BOLD:AAV6973
Stigmella hemargyrella|RMNH.INS.17611|Italy.Piedmont|BOLD:AAV6973
Stigmella hemargyrella|BC ZSM Lep 70717|Germany.Brandenburg|BOLD:AAV6973
Stigmella hemargyrella|TLMF Lep 10323|Austria.Tirol|BOLD:AAV6973
Stigmella hemargyrella|RMNH.INS.24594|Italy.Campania|BOLD:AAV6973
Stigmella hemargyrella|TLMF Lep 18477|Italy.South Tyrol|BOLD:AAV6973
Stigmella hemargyrella|KLM Lep 01958|Austria|BOLD:AAV6973
Stigmella hemargyrella|KLM Lep 01967|Slovenia|BOLD:AAV6973
Stigmella kurokoi|RMNH.INS.30142|South Korea|BOLD:ACU7198
Stigmella kurokoi|RMNH.INS.24560|South Korea|BOLD:ACU7197
Stigmella kurokoi|RMNH.INS.30015|Japan.Honshu|BOLD:ACU7197
Stigmella MoroccoEvN4043|RMNH.INS.24043|Morocco|BOLD:ACG8544
Stigmella alba|RMNH.INS.29624|United States.California|BOLD:ACG9281
Stigmella alba|RMNH.INS.24746|Canada.British Columbia|BOLD:ACG9281
Stigmella procrastinella|RMNH.INS.18322|United States.North Carolina|BOLD:ACG9281
Stigmella procrastinella|RMNH.INS.18326|United States.North Carolina|BOLD:ACG9281
Stigmella procrastinella|RMNH.INS.18243|United States.North Carolina|BOLD:ACG9281
Stigmella procrastinella|RMNH.INS.18190|United States.North Carolina|BOLD:ACG9281
Stigmella procrastinella|RMNH.INS.18316|United States.North Carolina|BOLD:ACG9281
Stigmella procrastinella|RMNH.INS.18248|United States.North Carolina|BOLD:ACG9281
Stigmella procrastinella|RMNH.INS.18314|United States.North Carolina|BOLD:ACG9281
Stigmella QuercusalbaVT|RMNH.INS.18638|United States.Vermont|BOLD:ACG9197
Stigmella atrata|RMNH.INS.24516|New Zealand|BOLD:ACU6020
Stigmella PlagianthusNZ|RMNH.INS.24517|New Zealand|BOLD:ACU5776
Stigmella PlagianthusNZ|RMNH.INS.24518|New Zealand|BOLD:ACU5776
Stigmella hakekeae|RMNH.INS.24513|New Zealand|BOLD:ACU5929
Stigmella ilsea|RMNH.INS.24515|New Zealand|BOLD:ACU6327
Stigmella ilsea|RMNH.INS.24514|New Zealand|BOLD:ACU6327
Stigmella RubusVietnam|RMNH.INS.24177|Vietnam.Lao Cai|BOLD:ACY4136
Stigmella hoheriae|RMNH.INS.11780|New Zealand.Auckland|BOLD:ACG8838
Stigmella hoheriae|RMNH.INS.11778|New Zealand.Auckland|BOLD:ACG8838
Stigmella sophorae|RMNH.INS.11779|New Zealand.Auckland|BOLD:ACG9085
Stigmella carpinella|BC ZSM Lep 76391|Germany.Bavaria|BOLD:ACH1840
Stigmella carpinella|BC ZSM Lep 76390|Germany.Bavaria|BOLD:ACH1840
Stigmella carpinella|RMNH.INS.29909|Azerbaijan|BOLD:ACH1840
Stigmella TrixisChile|RMNH.INS.24584|Chile|BOLD:ACU7421
Stigmella TrixisChile|RMNH.INS.24583|Chile|BOLD:ACU7421
Stigmella epicosma|RMNH.INS.24545|Chile|BOLD:ACU6296
Stigmella spinosissimae|RMNH.INS.24635|United Kingdom.England|BOLD:ACH1678
Stigmella spinosissimae|RMNH.INS.29948|Russia.Arkhangelskaya Oblast|BOLD:ACH1678
Stigmella spinosissimae|RMNH.INS.29946|Russia.Arkhangelskaya Oblast|BOLD:ACH1678
Stigmella salicisgrEvN4053|RMNH.INS.24053|United States.California|BOLD:ACY4871
Stigmella SalixKorea|RMNH.INS.30154|South Korea|BOLD:ACU5536
Stigmella molinensis|RMNH.INS.24219|Peru.Lima|BOLD:ACG9223
Stigmella molinensis|RMNH.INS.24812|Peru.Lima|BOLD:ACG9223
Stigmella molinensis|RMNH.INS.24218|Peru.Lima|BOLD:ACG9223
Stigmella benanderella|MM13939|Finland|BOLD:AAJ4185
Stigmella benanderella|MM14871|Finland|BOLD:AAJ4185
Stigmella benanderella|MM14868|Finland|BOLD:AAJ4185
Stigmella benanderella|MM14870|Finland|BOLD:AAJ4185
Stigmella SalixCO|RMNH.INS.29196|United States.Colorado|BOLD:ACG9406
Stigmella SalixPopCA|RMNH.INS.29936|United States.California|BOLD:ACH1777
Stigmella SalixPopCA|RMNH.INS.29930|United States.California|BOLD:ACH1777
Stigmella pallidiciliella|RMNH.INS.24619|Czech Republic|BOLD:ACU5916
Stigmella assimilella|TLMF Lep 16715|Italy.South Tyrol|BOLD:AAJ4391
Stigmella assimilella|RMNH.INS.12624|Netherlands.South Holland|BOLD:AAJ4391
Stigmella assimilella|RMNH.INS.30588|Germany.Saxony|BOLD:AAJ4391
Stigmella assimilella|RMNH.INS.12625|Netherlands.South Holland|BOLD:AAJ4391
Stigmella assimilella|MM06865|Finland.Aland Islands|BOLD:AAJ4391
Stigmella assimilella|RMNH.INS.12860|France.Provence-Alpes-Cote d'Azur|BOLD:AAJ4391
Stigmella assimilella|MM18054|Finland|BOLD:AAJ4391
Stigmella assimilella|TLMF Lep 18529|Italy.South Tyrol|BOLD:AAJ4391

Stigmella assimilella|RMNH.INS.12860|France.Provence-Alpes-Cote d'Azur|BOLD:AAJ4391
Stigmella assimilella|MM18054|Finland|BOLD:AAJ4391
Stigmella assimilella|TLMF Lep 18529|Italy.South Tyrol|BOLD:AAJ4391
Stigmella assimilella|TLMF Lep 15582|Italy.South Tyrol|BOLD:AAJ4391
Stigmella assimilella|TLMF Lep 18507|Italy.South Tyrol|BOLD:AAJ4391
Stigmella assimilella|BC ZSM Lep 89404|Germany.Bavaria|BOLD:AAJ4391
Stigmella assimilella|MM18055|Finland|BOLD:AAJ4391
Stigmella assimilella|TLMF Lep 18135|Italy.South Tyrol|BOLD:AAJ4391
Stigmella assimilella|TLMF Lep 02094|Italy.South Tyrol|BOLD:AAJ4391
Stigmella assimilella|TLMF Lep 02362|Italy.South Tyrol|BOLD:AAJ4391
Stigmella assimilella|TLMF Lep 18534|Italy.South Tyrol|BOLD:AAJ4391
Stigmella salixmyrtilloides_MM|MM21277|Finland|
Stigmella populetorum|CNCLEP00077718|Canada.British Columbia|BOLD:ABW0946
Stigmella populetorum|RMNH.INS.18594|United States.Vermont|BOLD:ABW0946
Stigmella populetorum|RMNH.INS.24131|United States.Montana|
Stigmella populetorum|RMNH.INS.30769|Canada.Quebec|BOLD:ABW0946
Stigmella populetorum|RMNH.INS.18595|United States.Vermont|BOLD:ABW0946
Stigmella populetorum|RMNH.INS.29883|Canada.Alberta|BOLD:ABW0946
Stigmella populetorum|RMNH.INS.30707|Canada.Ontario|BOLD:ABW0946
Stigmella populetorum|RMNH.INS.30750|Canada.Ontario|BOLD:ABW0946
Stigmella populetorum|RMNH.INS.30755|Canada.Ontario|BOLD:ABW0946
Stigmella populetorum|RMNH.INS.23724|Canada.Alberta|BOLD:ABW0946
Stigmella populetorum|RMNH.INS.24814|United States.Vermont|BOLD:ABW0946
Stigmella PopulusSD|RMNH.INS.30760|Canada.Quebec|BOLD:ACG9311
Stigmella PopulusSD|RMNH.INS.29603|United States.South Dakota|BOLD:ACG9311
Stigmella tranocrossa|RMNH.INS.30085|China.Tianjin|BOLD:ACU7132
Stigmella tranocrossa|RMNH.INS.24471|China.Shanxi|BOLD:ACU7132
Stigmella SalixAL|RMNH.INS.18367|United States.Alabama|BOLD:ACG9405
Stigmella PopulusCO|RMNH.INS.24785|Canada.Alberta|BOLD:ACY4954
Stigmella PopulusCO|RMNH.INS.29195|United States.Colorado|BOLD:AAY6974
Stigmella trimaculella|BC ZSM Lep 89407|Germany.Bavaria|BOLD:AAP7528
Stigmella trimaculella|RMNH.INS.12926|Italy.Piedmont|BOLD:AAP7528
Stigmella trimaculella|RMNH.INS.12623|Netherlands.South Holland|BOLD:AAP7528
Stigmella trimaculella|MM15181|Finland|BOLD:AAP7528
Stigmella trimaculella|TLMF Lep 09375|Austria.Tirol|BOLD:AAP7528
Stigmella trimaculella|RMNH.INS.18787|Greece|BOLD:AAP7528
Stigmella trimaculella|BC ZSM Lep 76458|Germany.Bavaria|BOLD:AAP7528
Stigmella trimaculella|RMNH.INS.30249|Russia.Novosibirskaya Oblast|BOLD:AAP7528
Stigmella trimaculella|MM15179|Finland|BOLD:AAP7528
Stigmella trimaculella|MM15180|Finland|BOLD:AAP7528
Stigmella azusa|RMNH.INS.24654|Japan.Honshu|BOLD:ACU6688
Stigmella azusa|RMNH.INS.29800|Japan.Hokkaido|BOLD:ACU6688
Stigmella vittata|RMNH.INS.29752|Japan.Hokkaido|BOLD:ACU6787
Stigmella vittata|RMNH.INS.29753|Japan.Hokkaido|BOLD:ACU6787
Stigmella vittata|RMNH.INS.24655|Japan.Honshu|BOLD:ACU6787
Stigmella salicisSpain|RMNH.INS.24289|Spain.Andalusia|BOLD:ACG8968
Stigmella saliciscluster5|RMNH.INS.29076|France.Rhone-Alpes|BOLD:AAT9424
Stigmella saliciscluster5|RMNH.INS.12001|France.Brittany|BOLD:AAT9424
Stigmella saliciscluster5|RMNH.INS.12002|France.Brittany|BOLD:AAT9424
Stigmella fuscotibiella|RMNH.INS.24416|United States.New Hampshire|BOLD:ACF2453
Stigmella fuscotibiella|RMNH.INS.18693|United States.New Hampshire|BOLD:ACF2453
Stigmella fuscotibiella|RMNH.INS.30714|Canada.Ontario|BOLD:ACF2453
Stigmella fuscotibiella|RMNH.INS.18592|United States.Vermont|BOLD:ACF2453
Stigmella myrtillella|KLM Lep 00924|Austria.Carinthia|BOLD:AAJ4189
Stigmella myrtillella|TLMF Lep 09216|Austria.Vorarlberg|BOLD:AAJ4189
Stigmella myrtillella|TLMF Lep 15194|Austria.Tirol|BOLD:AAJ4189
Stigmella myrtillella|BC ZSM Lep 89192|Germany.Bavaria|BOLD:AAJ4189
Stigmella myrtillella|RMNH.INS.24611|Italy.Piedmont|BOLD:AAJ4189
Stigmella myrtillella|RMNH.INS.12907|France.Provence-Alpes-Cote d'Azur|BOLD:AAJ4189
Stigmella myrtillella|RMNH.INS.24592|Italy.Campania|BOLD:AAJ4189
Stigmella myrtillella|MM24359|Finland|BOLD:AAJ4189
Stigmella myrtillella|MM21040|Finland|BOLD:AAJ4189
Stigmella myrtillella|MM24360|Finland|BOLD:AAJ4189
Stigmella myrtillella|MM24358|Finland|BOLD:AAJ4189
Stigmella myrtillella|MM24356|Finland|BOLD:AAJ4189
Stigmella myrtillella|RMNH.INS.12780|Sweden.Vastra Gotaland|BOLD:AAJ4189
Stigmella myrtillella|RMNH.INS.11436|Netherlands.Utrecht|BOLD:AAJ4189
Stigmella myrtillella|RMNH.INS.12772|Sweden.Kronoberg|BOLD:AAJ4189
Stigmella myrtillella|RMNH.INS.24287|Germany.Lower Saxony|BOLD:AAJ4189
Stigmella myrtillella|MM15174|Finland|BOLD:AAJ4189
Stigmella myrtillella|MM06330|Finland|BOLD:AAJ4189
Stigmella myrtillella|RMNH.INS.24288|Germany|BOLD:AAJ4189
Stigmella myrtillella|MM00936|Finland.Northern Ostrobothnia|BOLD:AAJ4189
Stigmella myrtillella|TLMF Lep 17955|Austria.Tirol|BOLD:AAJ4189
Stigmella myrtillella|RMNH.INS.29966|Russia.Murmanskaya Oblast|BOLD:AAJ4189
Stigmella myrtillella|KLM Lep 00319|Austria.Carinthia|BOLD:AAJ4189
Stigmella myrtillella|BC ZSM Lep 50943|Germany.Bavaria|BOLD:AAJ4189
Stigmella myrtillella|KLM Lep 01946|Austria|BOLD:AAJ4189
Stigmella myrtillella|TLMF Lep 18594|Italy.South Tyrol|BOLD:AAJ4189
Stigmella myrtillella|MM24726|Finland|BOLD:AAJ4189
Stigmella myrtillella|MM00935|Finland.Northern Ostrobothnia|BOLD:AAJ4189
Stigmella myrtillella|RMNH.INS.12771|Sweden.Kronoberg|BOLD:AAJ4189
Stigmella salicisSardinia|RMNH.INS.23622|Italy.Sardinia|BOLD:AAJ4189
Stigmella obliquella|RMNH.INS.18795|Greece|BOLD:AAJ4210
Stigmella obliquella|MM24427|Finland|BOLD:AAJ4210
Stigmella obliquella|MM15185|Finland|BOLD:AAJ4210
Stigmella obliquella|MM15173|Finland|BOLD:AAJ4210
Stigmella obliquella|MM15172|Finland|BOLD:AAJ4210
Stigmella obliquella|KLM Lep 00295|Austria.Carinthia|BOLD:AAJ4210
Stigmella obliquella|RMNH.INS.11339|Belgium.Luik (Liege)|BOLD:AAJ4210
Stigmella obliquella|KLM Lep 01264|Austria.Carinthia|BOLD:AAJ4210
Stigmella obliquella|KLM Lep 00281|Austria.Carinthia|BOLD:AAJ4210
Stigmella obliquella|BC ZSM Lep 89385|Germany.Bavaria|BOLD:AAJ4210
Stigmella obliquella|RMNH.INS.11340|Belgium.Luik (Liege)|BOLD:AAJ4210
Stigmella obliquella|RMNH.INS.17791|Finland.Northern Ostrobothnia|BOLD:AAJ4210
Stigmella obliquella|MM08696|Finland|BOLD:AAJ4210
Stigmella obliquella|RMNH.INS.18145|Bulgaria.Sofiya|BOLD:AAJ4210
Stigmella obliquella|RMNH.INS.12770|United Kingdom.England|BOLD:AAJ4210
Stigmella obliquella|BC ZSM Lep 89414|Germany.Bavaria|BOLD:AAJ4210
Stigmella obliquella|RMNH.INS.12634|Netherlands.South Holland|BOLD:AAJ4210

Stigmella obliquella|RMNH.INS.12770|United Kingdom.England|BOLD:AAJ4210
Stigmella obliquella|BC ZSM Lep 89414|Germany.Bavaria|BOLD:AAJ4210
Stigmella obliquella|RMNH.INS.12634|Netherlands.South Holland|BOLD:AAJ4210
Stigmella arbusculae|RMNH.INS.24617|Slovakia|BOLD:AAJ4187
Stigmella arbusculae|MM21282|Finland|BOLD:AAJ4187
Stigmella arbusculae|MM21195|Finland|BOLD:AAJ4187
Stigmella arbusculae|MM03444|Finland.Lapland|BOLD:AAJ4187
Stigmella arbusculae|MM21283|Finland|BOLD:AAJ4187
Stigmella saliciscluster2|MM22821|Finland|BOLD:AAC8507
Stigmella saliciscluster2|BC ZSM Lep 89386|Germany.Bavaria|BOLD:AAC8507
Stigmella saliciscluster2|TLMF Lep 15068|Austria.Tirol|BOLD:AAC8507
Stigmella saliciscluster2|MM24413|Finland|BOLD:AAC8507
Stigmella saliciscluster2|RMNH.INS.23740|Norway|BOLD:AAC8507
Stigmella saliciscluster2|TLMF Lep 15067|Austria.Tirol|BOLD:AAC8507
Stigmella saliciscluster2|MM24390|Finland|BOLD:AAC8507
Stigmella saliciscluster2|MM24388|Finland|BOLD:AAC8507
Stigmella saliciscluster2|MM24382|Finland|BOLD:AAC8507
Stigmella saliciscluster2|MM24410|Finland|BOLD:AAC8507
Stigmella saliciscluster2|MM24389|Finland|BOLD:AAC8507
Stigmella saliciscluster2|MM24384|Finland|BOLD:AAC8507
Stigmella saliciscluster2|MM24380|Finland|BOLD:AAC8507
Stigmella saliciscluster2|MM24385|Finland|BOLD:AAC8507
Stigmella saliciscluster2|RMNH.INS.17601|France.Provence-Alpes-Cote d'Azur|BOLD:AAC8507
Stigmella saliciscluster2|KLM Lep 02911|Austria|BOLD:AAC8507
Stigmella saliciscluster2|RMNH.INS.24819|Germany.North Rhine-Westphalia|BOLD:AAC8507
Stigmella saliciscluster2|TLMF Lep 17837|Austria.Tirol|BOLD:AAC8507
Stigmella saliciscluster2|KLM Lep 00303|Austria.Carinthia|BOLD:AAC8507
Stigmella saliciscluster2|TLMF Lep 18083|Austria.Tirol|BOLD:AAC8507
Stigmella saliciscluster2|KLM Lep 00926|Austria.Carinthia|BOLD:AAC8507
Stigmella saliciscluster2|BC ZSM Lep 89419|Germany.Bavaria|BOLD:AAC8507
Stigmella saliciscluster2|KLM Lep 02926|Austria|BOLD:AAC8507
Stigmella saliciscluster2|MM24364|Finland|BOLD:AAC8507
Stigmella saliciscluster2|KLM Lep 02921|Austria|BOLD:AAC8507
Stigmella saliciscluster2|TLMF Lep 16662|Italy.South Tyrol|BOLD:AAC8507
Stigmella saliciscluster2|RMNH.INS.29904|Russia.Volgogradskaya Oblast|BOLD:AAC8507
Stigmella saliciscluster2|KLM Lep 00636|Austria.Carinthia|BOLD:AAC8507
Stigmella saliciscluster2|MM22069|Finland|BOLD:AAC8507
Stigmella saliciscluster2|BC ZSM Lep 89401|Germany.Bavaria|BOLD:AAC8507
Stigmella saliciscluster2|MM22070|Finland|BOLD:AAC8507
Stigmella saliciscluster2|BC ZSM Lep 79385|Germany.Bavaria|
Stigmella saliciscluster2|TLMF Lep 18081|Austria.Tirol|BOLD:AAC8507
Stigmella saliciscluster2|TLMF Lep 17900|Austria.Tirol|BOLD:AAC8507
Stigmella saliciscluster2|KLM Lep 01979|Austria|BOLD:AAC8507
Stigmella saliciscluster2|TLMF Lep 15069|Austria.Tirol|BOLD:AAC8507
Stigmella saliciscluster2|RMNH.INS.24809|Luxembourg|BOLD:AAC8507
Stigmella saliciscluster2|MM09625|Finland.Aland Islands|BOLD:AAC8507
Stigmella saliciscluster2|MM24381|Finland|BOLD:AAC8507
Stigmella saliciscluster2|BC ZSM Lep 70746|Germany.Bavaria|BOLD:AAC8507
Stigmella saliciscluster2|MM22082|Finland|BOLD:AAC8507
Stigmella saliciscluster2|MM24412|Finland|BOLD:AAC8507
Stigmella saliciscluster2|MM21041|Finland|BOLD:AAC8507
Stigmella saliciscluster2|BC ZSM Lep 89437|Germany.Bavaria|BOLD:AAC8507
Stigmella saliciscluster2|MM22072|Finland|BOLD:AAC8507
Stigmella saliciscluster2|MM21275|Finland|BOLD:AAC8507
Stigmella saliciscluster2|KLM Lep 02920|Austria|BOLD:AAC8507
Stigmella saliciscluster2|TLMF Lep 15289|Austria.Tirol|BOLD:AAC8507
Stigmella saliciscluster2|BC ZSM Lep 64223|Germany.Bavaria|BOLD:AAC8507
Stigmella saliciscluster2|RMNH.INS.29683|Bulgaria|BOLD:AAC8507
Stigmella saliciscluster2|RMNH.INS.29689|Bulgaria.Kyustendil|BOLD:AAC8507
Stigmella saliciscluster2|BC ZSM Lep 85132|Germany.Bavaria|BOLD:AAC8507
Stigmella saliciscluster3|MM20739|Finland|BOLD:AAC8506
Stigmella saliciscluster3|MM24397|Finland|BOLD:AAC8506
Stigmella saliciscluster3|RMNH.INS.24630|Finland|BOLD:AAC8506
Stigmella saliciscluster3|MM24425|Finland|BOLD:AAC8506
Stigmella saliciscluster3|MM22084|Finland|BOLD:AAC8506
Stigmella saliciscluster3|RMNH.INS.12009|France.Brittany|BOLD:AAC8506
Stigmella saliciscluster3|MM24398|Finland|BOLD:AAC8506
Stigmella saliciscluster3|MM23304|Finland|BOLD:AAC8506
Stigmella saliciscluster3|MM09622|Finland.Aland Islands|BOLD:AAC8506
Stigmella saliciscluster3|MM23308|Finland|BOLD:AAC8506
Stigmella saliciscluster3|MM23302|Finland|BOLD:AAC8506
Stigmella saliciscluster3|MM24417|Finland|BOLD:AAC8506
Stigmella saliciscluster3|MM23305|Finland|BOLD:AAC8506
Stigmella saliciscluster3|MM23511|Finland|BOLD:AAC8506
Stigmella saliciscluster3|MM22085|Finland|BOLD:AAC8506
Stigmella saliciscluster3|MM09623|Finland.Aland Islands|BOLD:AAC8506
Stigmella saliciscluster3|MM24418|Finland|BOLD:AAC8506
Stigmella saliciscluster3|MM20740|Finland|BOLD:AAC8506
Stigmella saliciscluster3|MM23303|Finland|BOLD:AAC8506
Stigmella saliciscluster3|MM10521|Finland|BOLD:AAC8506
Stigmella saliciscluster3|MM24415|Finland|BOLD:AAC8506
Stigmella saliciscluster3|MM23307|Finland|BOLD:AAC8506
Stigmella saliciscluster3|MM24422|Finland|BOLD:AAC8506
Stigmella saliciscluster3|MM24399|Finland|BOLD:AAC8506
Stigmella saliciscluster3|MM23190|Finland|BOLD:AAC8506
Stigmella saliciscluster3|MM24416|Finland|BOLD:AAC8506
Stigmella saliciscluster3|RMNH.INS.24228|France.Provence-Alpes-Cote d'Azur|BOLD:AAC8506
Stigmella saliciscluster3|RMNH.INS.12909|France.Provence-Alpes-Cote d'Azur|BOLD:AAC8506
Stigmella saliciscluster3|RMNH.INS.12908|France.Provence-Alpes-Cote d'Azur|BOLD:AAC8506
Stigmella saliciscluster3|MM23301|Finland|BOLD:AAC8506
Stigmella salicis|MM14709|Finland|
Stigmella salicis|CNCLEP00026624|Canada.Yukon Territory|BOLD:AAC8505
Stigmella salicis|RMNH.INS.24216|Canada.Yukon Territory|BOLD:AAC8505
Stigmella salicis|RMNH.INS.12641|Netherlands.South Holland|BOLD:AAC8505
Stigmella salicis|RMNH.INS.29212|South Korea|BOLD:AAC8505
Stigmella salicis|MM09383|Finland|BOLD:AAC8505
Stigmella salicis|TLMF Lep 12013|Italy.South Tyrol|BOLD:AAC8505
Stigmella salicis|TLMF Lep 10321|Austria.Tirol|BOLD:AAC8505
Stigmella salicis|KLM Lep 00315|Austria.Carinthia|BOLD:AAC8505
Stigmella salicis|TLMF Lep 12014|Italy.South Tyrol|BOLD:AAC8505

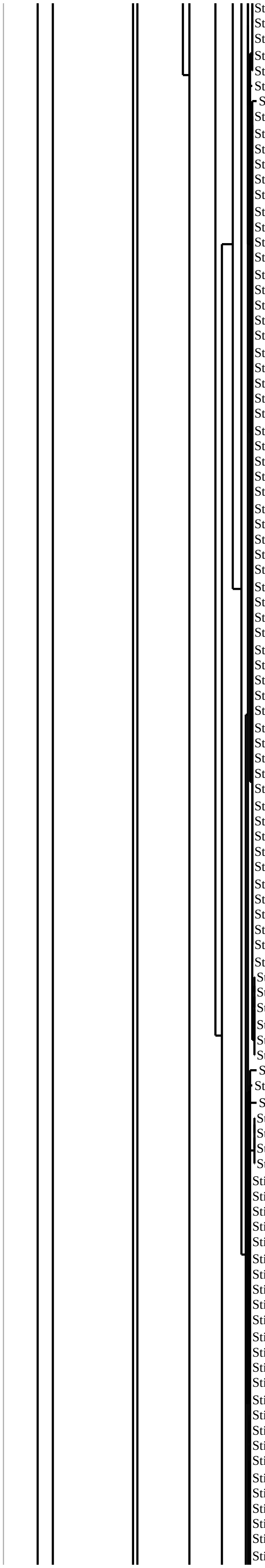

Stigmella salicis|TLMF Lep 10321|Austria.Tirol|BOLD:AAC8505
Stigmella salicis|KLM Lep 00315|Austria.Carinthia|BOLD:AAC8505
Stigmella salicis|TLMF Lep 12014|Italy.South Tyrol|BOLD:AAC8505
Stigmella salicis|RMNH.INS.23822|Germany.North Rhine-Westphalia|BOLD:AAC8505
Stigmella salicis|RMNH.INS.12769|United Kingdom.England|BOLD:AAC8505
Stigmella salicis|MM24373|Finland|BOLD:AAC8505
Stigmella salicis|MM24406|Finland|BOLD:AAC8505
Stigmella salicis|RMNH.INS.11241|Netherlands.Zeeland|BOLD:AAC8505
Stigmella salicis|MM24414|Finland|BOLD:AAC8505
Stigmella salicis|MM24428|Finland|BOLD:AAC8505
Stigmella salicis|MM23178|Finland|BOLD:AAC8505
Stigmella salicis|MM24400|Finland|BOLD:AAC8505
Stigmella salicis|MM16629|Finland|BOLD:AAC8505
Stigmella salicis|MM24372|Finland|BOLD:AAC8505
Stigmella salicis|MM09436|Finland|BOLD:AAC8505
Stigmella salicis|MM24363|Finland|BOLD:AAC8505
Stigmella salicis|MM21276|Finland|BOLD:AAC8505
Stigmella salicis|MM10522|Finland|BOLD:AAC8505
Stigmella salicis|MM24395|Finland|BOLD:AAC8505
Stigmella salicis|MM20743|Finland|BOLD:AAC8505
Stigmella salicis|MM24419|Finland|BOLD:AAC8505
Stigmella salicis|MM21193|Finland|BOLD:AAC8505
Stigmella salicis|MM23780|Finland|BOLD:AAC8505
Stigmella salicis|MM24432|Finland|BOLD:AAC8505
Stigmella salicis|MM23306|Finland|BOLD:AAC8505
Stigmella salicis|MM20744|Finland|BOLD:AAC8505
Stigmella salicis|MM24434|Finland|BOLD:AAC8505
Stigmella salicis|MM24421|Finland|BOLD:AAC8505
Stigmella salicis|MM24401|Finland|BOLD:AAC8505
Stigmella salicis|RMNH.INS.11370|Netherlands.South Holland|BOLD:AAC8505
Stigmella salicis|MM24433|Finland|BOLD:AAC8505
Stigmella salicis|MM24393|Finland|BOLD:AAC8505
Stigmella salicis|MM20745|Finland|BOLD:AAC8505
Stigmella salicis|MM24402|Finland|BOLD:AAC8505
Stigmella salicis|MM20741|Finland|BOLD:AAC8505
Stigmella salicis|MM24362|Finland|BOLD:AAC8505
Stigmella salicis|MM21280|Finland|BOLD:AAC8505
Stigmella salicis|MM20746|Finland|BOLD:AAC8505
Stigmella salicis|MM24426|Finland|BOLD:AAC8505
Stigmella salicis|MM24403|Finland|BOLD:AAC8505
Stigmella salicis|MM24378|Finland|BOLD:AAC8505
Stigmella salicis|MM00940|Finland.Northern Ostrobothnia|BOLD:AAC8505
Stigmella salicis|MM24394|Finland|BOLD:AAC8505
Stigmella salicis|MM24420|Finland|BOLD:AAC8505
Stigmella salicis|MM21039|Finland|BOLD:AAC8505
Stigmella salicis|MM23204|Finland|BOLD:AAC8505
Stigmella salicis|MM24391|Finland|BOLD:AAC8505
Stigmella salicis|MM07006|Finland|BOLD:AAC8505
Stigmella salicis|MM24392|Finland|BOLD:AAC8505
Stigmella salicis|MM24371|Finland|BOLD:AAC8505
Stigmella salicis|MM14055|Finland|BOLD:AAC8505
Stigmella salicis|MM22079|Finland|BOLD:AAC8505
Stigmella salicis|MM09304|Finland|BOLD:AAC8505
Stigmella salicis|MM24411|Finland|BOLD:AAC8505
Stigmella salicis|MM24377|Finland|BOLD:AAC8505
Stigmella salicis|MM22890|Finland|BOLD:AAC8505
Stigmella salicis|MM24375|Finland|BOLD:AAC8505
Stigmella salicis|MM21038|Finland|BOLD:AAC8505
Stigmella salicis|MM24407|Finland|BOLD:AAC8505
Stigmella salicis|MM24423|Finland|BOLD:AAC8505
Stigmella salicis|MM24352|Finland|BOLD:AAC8505
Stigmella salicis|RMNH.INS.24820|Germany.North Rhine-Westphalia|BOLD:AAC8505
Stigmella salicis|TLMF Lep 09970|Austria.Vorarlberg|BOLD:AAC8505
Stigmella salicis|MM24396|Finland|BOLD:AAC8505
Stigmella salicis|MM22078|Finland|BOLD:AAC8505
Stigmella salicis|MM09432|Finland|BOLD:AAC8505
Stigmella salicis|MM24369|Finland|BOLD:AAC8505
Stigmella salicis|MM09431|Finland|BOLD:AAC8505
Stigmella salicis|MM22077|Finland|BOLD:AAC8505
Stigmella salicis|MM24365|Finland|BOLD:AAC8505
Stigmella salicis|TLMF Lep 10322|Austria.Tirol|BOLD:AAC8505
Stigmella salicis|MM22076|Finland|BOLD:AAC8505
Stigmella salicis|MM22075|Finland|BOLD:AAC8505
Stigmella salicis|MM22073|Finland|BOLD:AAC8505
Stigmella salicis|MM22074|Finland|BOLD:AAC8505
Stigmella salicis|RMNH.INS.17789|Finland.Northern Ostrobothnia|BOLD:AAC8505
Stigmella salicis|RMNH.INS.12659|Sweden.Lappland|BOLD:AAC8505
Stigmella salicis|RMNH.INS.17790|Finland.Lapland|BOLD:AAC8505
Stigmella salicis|RMNH.INS.23930|United Kingdom.England|BOLD:AAC8505
Stigmella salicis|RMNH.INS.12660|Sweden.Lappland|BOLD:AAC8505
Stigmella salicis|MM24429|Finland|BOLD:AAC8505
Stigmella salicis|MM24353|Finland|BOLD:AAC8505
Stigmella salicis|MM24404|Finland|BOLD:AAC8505
Stigmella salicis|MM02641|Finland.South Karelia|BOLD:AAC8505
Stigmella salicis|MM23194|Finland|BOLD:AAC8505
Stigmella salicis|MM24376|Finland|BOLD:AAC8505
Stigmella salicis|MM22071|Finland|BOLD:AAC8505
Stigmella salicis|MM24405|Finland|BOLD:AAC8505
Stigmella salicis|MM20742|Finland|BOLD:AAC8505
Stigmella salicis|MM23193|Finland|BOLD:AAC8505
Stigmella salicis|MM09384|Finland|BOLD:AAC8505
Stigmella salicis|MM21279|Finland|BOLD:AAC8505
Stigmella salicis|MM24366|Finland|BOLD:AAC8505
Stigmella salicis|MM24368|Finland|BOLD:AAC8505
Stigmella salicis|MM24409|Finland|BOLD:AAC8505
Stigmella salicis|MM24430|Finland|BOLD:AAC8505
Stigmella salicis|MM24354|Finland|BOLD:AAC8505
Stigmella salicis|MM09624|Finland.Aland Islands|BOLD:AAC8505
Stigmella salicis|MM14191|Finland|BOLD:AAC8505
Stigmella salicis|MM24408|Finland|BOLD:AAC8505

Stigmella salicis|MM09624|Finland.Aland Islands|BOLD:AAC8505
Stigmella salicis|MM14191|Finland|BOLD:AAC8505
Stigmella salicis|MM24408|Finland|BOLD:AAC8505
Stigmella salicis|MM24383|Finland|BOLD:AAC8505
Stigmella salicis|MM24379|Finland|BOLD:AAC8505
Stigmella salicis|MM24386|Finland|BOLD:AAC8505
Stigmella salicis|MM24370|Finland|BOLD:AAC8505
Stigmella salicis|MM22824|Finland|BOLD:AAC8505
Stigmella salicis|MM06304|Finland|BOLD:AAC8505
Stigmella salicis|MM24424|Finland|BOLD:AAC8505
Stigmella salicis|MM24374|Finland|BOLD:AAC8505
Stigmella salicis|MM23195|Finland|BOLD:AAC8505
Stigmella salicis|RMNH.INS.17877|United Kingdom.England|BOLD:AAC8505
Stigmella salicis|RMNH.INS.17878|United Kingdom.England|BOLD:AAC8505
Stigmella salicis|MM21278|Finland|BOLD:AAC8505
Stigmella salicis|RMNH.INS.12768|United Kingdom.England|BOLD:AAC8505
Stigmella salicis|MM23963|Finland|BOLD:AAC8505
Stigmella salicis|MM24431|Finland|BOLD:AAC8505
Stigmella salicis|RMNH.INS.18745|Germany.North Rhine-Westphalia|BOLD:AAC8505
Stigmella SalixTaiwan|RMNH.INS.29395|Taiwan.Hualien City|BOLD:AAC8505
Stigmella SalixTaiwan|RMNH.INS.29455|Taiwan.Nantou County|BOLD:AAC8505
Stigmella saliciscplxEvN4742|RMNH.INS.24742|United States.Indiana|BOLD:ACY4522
Stigmella SalixJapanCD13047|RMNH.INS.30087|China.Tianjin|BOLD:ACU6083
Stigmella SalixJapanCD13047|RMNH.INS.29799|Japan.Hokkaido|BOLD:ACU6083
Stigmella SalixJapanCD13047|RMNH.INS.29797|Japan.Hokkaido|BOLD:ACU6083
Stigmella tenryuensis|RMNH.INS.24653|Japan.Honshu|BOLD:ACU5537
Stigmella saliciscluster6|RMNH.INS.18757|United Kingdom.England|BOLD:AAT9425
Stigmella saliciscluster6|RMNH.INS.12774|United Kingdom.England|BOLD:AAT9425
Stigmella saliciscluster6|RMNH.INS.12775|United Kingdom.England|BOLD:AAT9425
Stigmella saliciscluster6|RMNH.INS.23929|United Kingdom.England|BOLD:AAT9425
Stigmella SalixSD|CNCLEP00029458|Canada.British Columbia|BOLD:AAH4281
Stigmella SalixSD|RMNH.INS.29605|United States.South Dakota|BOLD:AAH4281
Stigmella SalixSD|CNCLEP00069468|Canada.British Columbia|BOLD:AAH4281
Stigmella vimineticola|BC ZSM Lep 100101|Germany.Bavaria|
Stigmella vimineticola|BC ZSM Lep 100102|Germany.Bavaria|BOLD:AAU4353
Stigmella vimineticola|TLMF Lep 18071|Austria.Tirol|BOLD:AAU4353
Stigmella vimineticola|TLMF Lep 08876|Austria.Tirol|BOLD:AAU4353
Stigmella vimineticola|TLMF Lep 15122|Italy.South Tyrol|BOLD:AAU4353
Stigmella vimineticola|BC ZSM Lep 100103|Germany.Bavaria|BOLD:AAU4353
Stigmella vimineticola|TLMF Lep 13382|Austria.Tirol|BOLD:AAU4353
Stigmella vimineticola|TLMF Lep 15193|Austria.Tirol|BOLD:AAU4353
Stigmella vimineticola|RMNH.INS.24620|Slovakia|BOLD:AAU4353
Stigmella vimineticola|TLMF Lep 09374|Austria.Tirol|BOLD:AAU4353
Stigmella vimineticola|TLMF Lep 18236|Austria.Tirol|BOLD:AAU4353
Stigmella vimineticola|TLMF Lep 14909|Austria|BOLD:AAU4353
Stigmella vimineticola|TLMF Lep 07333|Austria.Tirol|BOLD:AAU4353
Stigmella vimineticola|TLMF Lep 13383|Austria.Tirol|BOLD:AAU4353
Stigmella vimineticola|RMNH.INS.24810|Switzerland.Valais|BOLD:AAU4353
Stigmella vimineticola|TLMF Lep 07337|Austria.Tirol|BOLD:AAU4353
Stigmella vimineticola|RMNH.INS.17605|France.Provence-Alpes-Cote d'Azur|BOLD:AAU4353
Stigmella vimineticola|RMNH.INS.24555|Italy.Friuli-Venezia Giulia|BOLD:AAU4353
Stigmella vimineticola|BC ZSM Lep 89496|Italy.Friuli-Venezia Giulia|BOLD:AAU4353
Stigmella vimineticola|TLMF Lep 16680|Italy.South Tyrol|BOLD:AAU4353
Stigmella vimineticola|RMNH.INS.17684|Italy.Piedmont|BOLD:AAU4353
Stigmella vimineticola|RMNH.INS.24591|Italy.Friuli-Venezia Giulia|BOLD:AAU4353
Stigmella vimineticola|TLMF Lep 16681|Italy.South Tyrol|BOLD:AAU4353
Stigmella vimineticola|TLMF Lep 17953|Italy.South Tyrol|BOLD:AAU4353
Stigmella vimineticola|TLMF Lep 15625|Italy.South Tyrol|BOLD:AAU4353
Stigmella zelleriella|RMNH.INS.12750|Sweden.Halland|BOLD:AAI0000
Stigmella zelleriella|RMNH.INS.12747|Sweden.Halland|BOLD:AAI0000
Stigmella zelleriella|RMNH.INS.12748|Sweden.Halland|BOLD:AAI0000
Stigmella zelleriella|RMNH.INS.12749|Sweden.Halland|BOLD:AAI0000
Stigmella zelleriella|RMNH.INS.12751|Sweden.Halland|BOLD:AAI0000
Stigmella zelleriella|MM09373|Finland|BOLD:AAI0000
Stigmella zelleriella|RMNH.INS.12757|Finland.Northern Ostrobothnia|BOLD:AAI0000
Stigmella zelleriella|RMNH.INS.12755|Finland.Northern Ostrobothnia|BOLD:AAI0000
Stigmella zelleriella|RMNH.INS.11377|Netherlands.South Holland|BOLD:AAI0000
Stigmella zelleriella|MM09372|Finland|BOLD:AAI0000
Stigmella zelleriella|MM14708|Finland|BOLD:AAI0000
Stigmella zelleriella|MM09371|Finland|BOLD:AAI0000
Stigmella zelleriella|MM09370|Finland|BOLD:AAI0000
Stigmella zelleriella|MM14846|Finland|BOLD:AAI0000
Stigmella zelleriella|MM09365|Finland|BOLD:AAI0000
Stigmella zelleriella|MM09364|Finland|BOLD:AAI0000
Stigmella zelleriella|RMNH.INS.11931|Netherlands.North Holland|BOLD:AAI0000
Stigmella zelleriella|RMNH.INS.11932|Netherlands.North Holland|BOLD:AAI0000
Stigmella zelleriella|RMNH.INS.12754|Finland.Northern Ostrobothnia|BOLD:AAI0000
Stigmella zelleriella|RMNH.INS.12758|Finland.Lapland|BOLD:AAI0000
Stigmella zelleriella|RMNH.INS.12753|Finland.Lapland|BOLD:AAI0000
Stigmella zelleriella|RMNH.INS.17795|Finland.Northern Ostrobothnia|BOLD:AAI0000
Stigmella zelleriella|RMNH.INS.12689|Sweden.Lappland|BOLD:AAI0000
Stigmella zelleriella|RMNH.INS.12756|Finland.Northern Ostrobothnia|BOLD:AAI0000
Stigmella zelleriella|RMNH.INS.17792|Finland.Northern Ostrobothnia|BOLD:AAI0000
Stigmella zelleriella|MM14707|Finland|BOLD:AAI0000
Stigmella zelleriella|RMNH.INS.12759|Finland.Lapland|BOLD:AAI0000
Stigmella zelleriella|RMNH.INS.12681|Sweden.Lappland|BOLD:AAI0000
Stigmella zelleriella|RMNH.INS.12752|Finland.Lapland|BOLD:AAI0000
Stigmella zelleriella|RMNH.INS.12680|Sweden.Lappland|BOLD:AAI0000
Stigmella QuercusglaucaTaiwan|RMNH.INS.29434|Taiwan.Nantou County|BOLD:ACG9344
Stigmella argentifasciella|RMNH.INS.18545|United States.New York|BOLD:ACG8834
Stigmella argentifasciella|RMNH.INS.30747|Canada.Ontario|BOLD:ACG8834
Stigmella argentifasciella|RMNH.INS.24406|United States.New York|BOLD:ACG8834
Stigmella BorneoEvN4565|RMNH.INS.24565|Indonesia.Kalimantan Timur|BOLD:ACU6018
Stigmella Berchemiascandens|RMNH.INS.29937|United States.Tennessee|BOLD:ACH1549
Stigmella rhamnicola|RMNH.INS.18577|United States.Vermont|BOLD:AAU7678
Stigmella rhamnicola|RMNH.INS.24410|United States.Vermont|BOLD:AAU7678
Stigmella rhamnicola|RMNH.INS.23726|Canada.Saskatchewan|BOLD:AAU7678
Stigmella rhamnicola|RMNH.INS.30757|Canada.Ontario|BOLD:AAU7678
Stigmella rhamnicola|RMNH.INS.24411|United States.Vermont|BOLD:AAU7678
Stigmella rhamnicola|RMNH.INS.24789|Canada.Ontario|BOLD:AAU7678
[illegible]

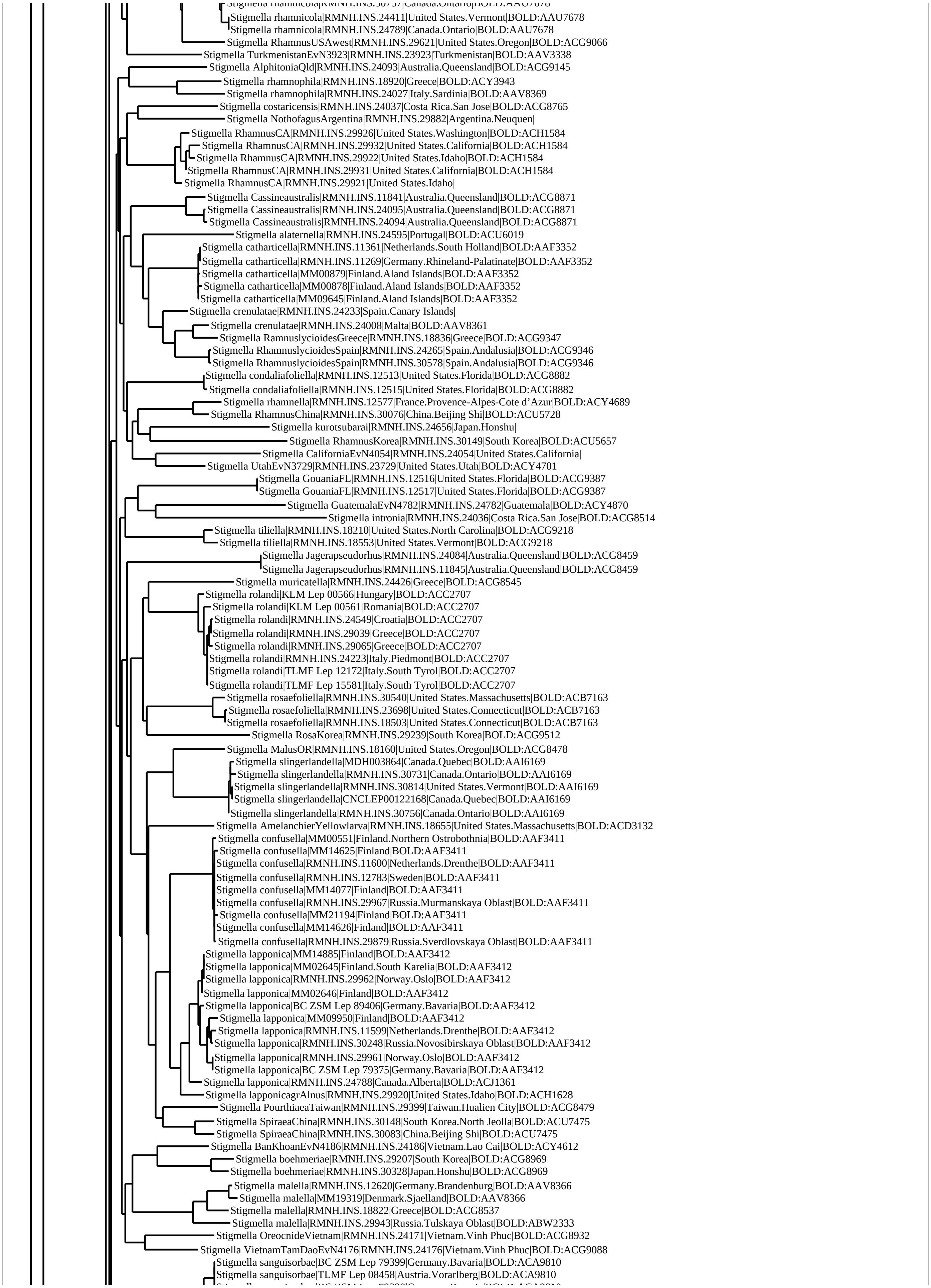
Stigmella rhamnicola|RMNH.INS.30757|Canada.Ontario|BOLD:AAU7678
Stigmella rhamnicola|RMNH.INS.24411|United States.Vermont|BOLD:AAU7678
Stigmella rhamnicola|RMNH.INS.24789|Canada.Ontario|BOLD:AAU7678
Stigmella RhamnusUSAwest|RMNH.INS.29621|United States.Oregon|BOLD:ACG9066
Stigmella TurkmenistanEvN3923|RMNH.INS.23923|Turkmenistan|BOLD:AAV3338
Stigmella AlphitoniaQld|RMNH.INS.24093|Australia.Queensland|BOLD:ACG9145
Stigmella rhamnophila|RMNH.INS.18920|Greece|BOLD:ACY3943
Stigmella rhamnophila|RMNH.INS.24027|Italy.Sardinia|BOLD:AAV8369
Stigmella costaricensis|RMNH.INS.24037|Costa Rica.San Jose|BOLD:ACG8765
Stigmella NothofagusArgentina|RMNH.INS.29882|Argentina.Neuquen|
Stigmella RhamnusCA|RMNH.INS.29926|United States.Washington|BOLD:ACH1584
Stigmella RhamnusCA|RMNH.INS.29932|United States.California|BOLD:ACH1584
Stigmella RhamnusCA|RMNH.INS.29922|United States.Idaho|BOLD:ACH1584
Stigmella RhamnusCA|RMNH.INS.29931|United States.California|BOLD:ACH1584
Stigmella RhamnusCA|RMNH.INS.29921|United States.Idaho|
Stigmella Cassineaustralis|RMNH.INS.11841|Australia.Queensland|BOLD:ACG8871
Stigmella Cassineaustralis|RMNH.INS.24095|Australia.Queensland|BOLD:ACG8871
Stigmella Cassineaustralis|RMNH.INS.24094|Australia.Queensland|BOLD:ACG8871
Stigmella alaternella|RMNH.INS.24595|Portugal|BOLD:ACU6019
Stigmella catharticella|RMNH.INS.11361|Netherlands.South Holland|BOLD:AAF3352
Stigmella catharticella|RMNH.INS.11269|Germany.Rhineland-Palatinate|BOLD:AAF3352
Stigmella catharticella|MM00879|Finland.Aland Islands|BOLD:AAF3352
Stigmella catharticella|MM00878|Finland.Aland Islands|BOLD:AAF3352
Stigmella catharticella|MM09645|Finland.Aland Islands|BOLD:AAF3352
Stigmella crenulatae|RMNH.INS.24233|Spain.Canary Islands|
Stigmella crenulatae|RMNH.INS.24008|Malta|BOLD:AAV8361
Stigmella RamnuslycioidesGreece|RMNH.INS.18836|Greece|BOLD:ACG9347
Stigmella RhamnuslycioidesSpain|RMNH.INS.24265|Spain.Andalusia|BOLD:ACG9346
Stigmella RhamnuslycioidesSpain|RMNH.INS.30578|Spain.Andalusia|BOLD:ACG9346
Stigmella condaliafoliella|RMNH.INS.12513|United States.Florida|BOLD:ACG8882
Stigmella condaliafoliella|RMNH.INS.12515|United States.Florida|BOLD:ACG8882
Stigmella rhamnella|RMNH.INS.12577|France.Provence-Alpes-Cote d'Azur|BOLD:ACY4689
Stigmella RhamnusChina|RMNH.INS.30076|China.Beijing Shi|BOLD:ACU5728
Stigmella kurotsubarai|RMNH.INS.24656|Japan.Honshu|
Stigmella RhamnusKorea|RMNH.INS.30149|South Korea|BOLD:ACU5657
Stigmella CaliforniaEvN4054|RMNH.INS.24054|United States.California|
Stigmella UtahEvN3729|RMNH.INS.23729|United States.Utah|BOLD:ACY4701
Stigmella GouaniaFL|RMNH.INS.12516|United States.Florida|BOLD:ACG9387
Stigmella GouaniaFL|RMNH.INS.12517|United States.Florida|BOLD:ACG9387
Stigmella GuatemalaEvN4782|RMNH.INS.24782|Guatemala|BOLD:ACY4870
Stigmella intronia|RMNH.INS.24036|Costa Rica.San Jose|BOLD:ACG8514
Stigmella tiliella|RMNH.INS.18210|United States.North Carolina|BOLD:ACG9218
Stigmella tiliella|RMNH.INS.18553|United States.Vermont|BOLD:ACG9218
Stigmella Jagerapseudorhus|RMNH.INS.24084|Australia.Queensland|BOLD:ACG8459
Stigmella Jagerapseudorhus|RMNH.INS.11845|Australia.Queensland|BOLD:ACG8459
Stigmella muricatella|RMNH.INS.24426|Greece|BOLD:ACG8545
Stigmella rolandi|KLM Lep 00566|Hungary|BOLD:ACC2707
Stigmella rolandi|KLM Lep 00561|Romania|BOLD:ACC2707
Stigmella rolandi|RMNH.INS.24549|Croatia|BOLD:ACC2707
Stigmella rolandi|RMNH.INS.29039|Greece|BOLD:ACC2707
Stigmella rolandi|RMNH.INS.29065|Greece|BOLD:ACC2707
Stigmella rolandi|RMNH.INS.24223|Italy.Piedmont|BOLD:ACC2707
Stigmella rolandi|TLMF Lep 12172|Italy.South Tyrol|BOLD:ACC2707
Stigmella rolandi|TLMF Lep 15581|Italy.South Tyrol|BOLD:ACC2707
Stigmella rosaefoliella|RMNH.INS.30540|United States.Massachusetts|BOLD:ACB7163
Stigmella rosaefoliella|RMNH.INS.23698|United States.Connecticut|BOLD:ACB7163
Stigmella rosaefoliella|RMNH.INS.18503|United States.Connecticut|BOLD:ACB7163
Stigmella RosaKorea|RMNH.INS.29239|South Korea|BOLD:ACG9512
Stigmella MalusOR|RMNH.INS.18160|United States.Oregon|BOLD:ACG8478
Stigmella slingerlandella|MDH003864|Canada.Quebec|BOLD:AAI6169
Stigmella slingerlandella|RMNH.INS.30731|Canada.Ontario|BOLD:AAI6169
Stigmella slingerlandella|RMNH.INS.30814|United States.Vermont|BOLD:AAI6169
Stigmella slingerlandella|CNCLEP00122168|Canada.Quebec|BOLD:AAI6169
Stigmella slingerlandella|RMNH.INS.30756|Canada.Ontario|BOLD:AAI6169
Stigmella AmelanchierYellowlarva|RMNH.INS.18655|United States.Massachusetts|BOLD:ACD3132
Stigmella confusella|MM00551|Finland.Northern Ostrobothnia|BOLD:AAF3411
Stigmella confusella|MM14625|Finland|BOLD:AAF3411
Stigmella confusella|RMNH.INS.11600|Netherlands.Drenthe|BOLD:AAF3411
Stigmella confusella|RMNH.INS.12783|Sweden|BOLD:AAF3411
Stigmella confusella|MM14077|Finland|BOLD:AAF3411
Stigmella confusella|RMNH.INS.29967|Russia.Murmanskaya Oblast|BOLD:AAF3411
Stigmella confusella|MM21194|Finland|BOLD:AAF3411
Stigmella confusella|MM14626|Finland|BOLD:AAF3411
Stigmella confusella|RMNH.INS.29879|Russia.Sverdlovskaya Oblast|BOLD:AAF3411
Stigmella lapponica|MM14885|Finland|BOLD:AAF3412
Stigmella lapponica|MM02645|Finland.South Karelia|BOLD:AAF3412
Stigmella lapponica|RMNH.INS.29962|Norway.Oslo|BOLD:AAF3412
Stigmella lapponica|MM02646|Finland|BOLD:AAF3412
Stigmella lapponica|BC ZSM Lep 89406|Germany.Bavaria|BOLD:AAF3412
Stigmella lapponica|MM09950|Finland|BOLD:AAF3412
Stigmella lapponica|RMNH.INS.11599|Netherlands.Drenthe|BOLD:AAF3412
Stigmella lapponica|RMNH.INS.30248|Russia.Novosibirskaya Oblast|BOLD:AAF3412
Stigmella lapponica|RMNH.INS.29961|Norway.Oslo|BOLD:AAF3412
Stigmella lapponica|BC ZSM Lep 79375|Germany.Bavaria|BOLD:AAF3412
Stigmella lapponica|RMNH.INS.24788|Canada.Alberta|BOLD:ACJ1361
Stigmella lapponicagrAlnus|RMNH.INS.29920|United States.Idaho|BOLD:ACH1628
Stigmella PourthiaeaTaiwan|RMNH.INS.29399|Taiwan.Hualien City|BOLD:ACG8479
Stigmella SpiraeaChina|RMNH.INS.30148|South Korea.North Jeolla|BOLD:ACU7475
Stigmella SpiraeaChina|RMNH.INS.30083|China.Beijing Shi|BOLD:ACU7475
Stigmella BanKhoanEvN4186|RMNH.INS.24186|Vietnam.Lao Cai|BOLD:ACY4612
Stigmella boehmeriae|RMNH.INS.29207|South Korea|BOLD:ACG8969
Stigmella boehmeriae|RMNH.INS.30328|Japan.Honshu|BOLD:ACG8969
Stigmella malella|RMNH.INS.12620|Germany.Brandenburg|BOLD:AAV8366
Stigmella malella|MM19319|Denmark.Sjaelland|BOLD:AAV8366
Stigmella malella|RMNH.INS.18822|Greece|BOLD:ACG8537
Stigmella malella|RMNH.INS.29943|Russia.Tulskaya Oblast|BOLD:ABW2333
Stigmella OreocnideVietnam|RMNH.INS.24171|Vietnam.Vinh Phuc|BOLD:ACG8932
Stigmella VietnamTamDaoEvN4176|RMNH.INS.24176|Vietnam.Vinh Phuc|BOLD:ACG9088
Stigmella sanguisorbae|BC ZSM Lep 79399|Germany.Bavaria|BOLD:ACA9810
Stigmella sanguisorbae|TLMF Lep 08458|Austria.Vorarlberg|BOLD:ACA9810
[illegible]

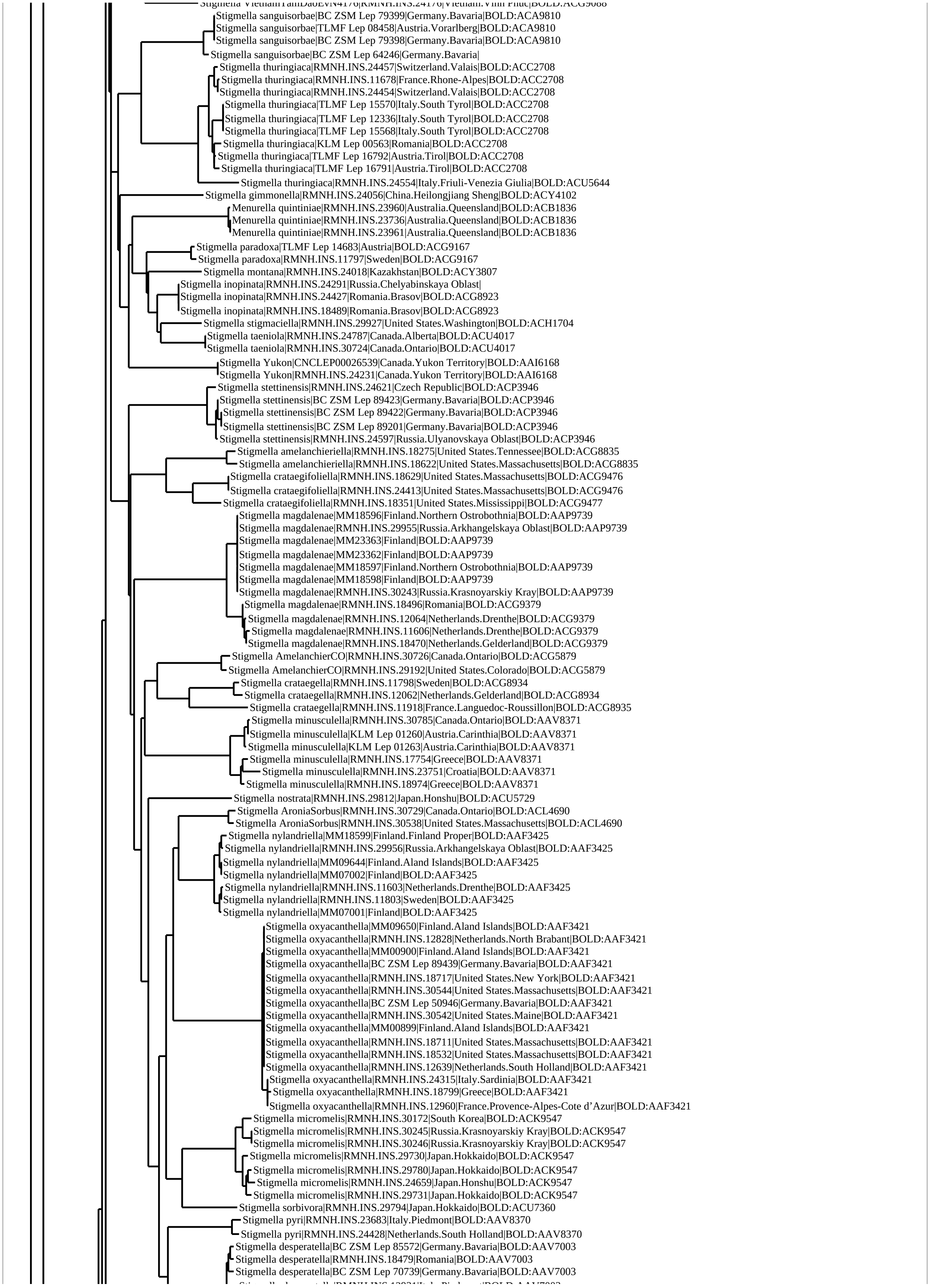
Stigmella sanguisorbae|BC ZSM Lep 79399|Germany.Bavaria|BOLD:ACA9810
Stigmella sanguisorbae|TLMF Lep 08458|Austria.Vorarlberg|BOLD:ACA9810
Stigmella sanguisorbae|BC ZSM Lep 79398|Germany.Bavaria|BOLD:ACA9810
Stigmella sanguisorbae|BC ZSM Lep 64246|Germany.Bavaria|
Stigmella thuringiaca|RMNH.INS.24457|Switzerland.Valais|BOLD:ACC2708
Stigmella thuringiaca|RMNH.INS.11678|France.Rhone-Alpes|BOLD:ACC2708
Stigmella thuringiaca|RMNH.INS.24454|Switzerland.Valais|BOLD:ACC2708
Stigmella thuringiaca|TLMF Lep 15570|Italy.South Tyrol|BOLD:ACC2708
Stigmella thuringiaca|TLMF Lep 12336|Italy.South Tyrol|BOLD:ACC2708
Stigmella thuringiaca|TLMF Lep 15568|Italy.South Tyrol|BOLD:ACC2708
Stigmella thuringiaca|KLM Lep 00563|Romania|BOLD:ACC2708
Stigmella thuringiaca|TLMF Lep 16792|Austria.Tirol|BOLD:ACC2708
Stigmella thuringiaca|TLMF Lep 16791|Austria.Tirol|BOLD:ACC2708
Stigmella thuringiaca|RMNH.INS.24554|Italy.Friuli-Venezia Giulia|BOLD:ACU5644
Stigmella gimmonella|RMNH.INS.24056|China.Heilongjiang Sheng|BOLD:ACY4102
Menurella quintiniae|RMNH.INS.23960|Australia.Queensland|BOLD:ACB1836
Menurella quintiniae|RMNH.INS.23736|Australia.Queensland|BOLD:ACB1836
Menurella quintiniae|RMNH.INS.23961|Australia.Queensland|BOLD:ACB1836
Stigmella paradoxa|TLMF Lep 14683|Austria|BOLD:ACG9167
Stigmella paradoxa|RMNH.INS.11797|Sweden|BOLD:ACG9167
Stigmella montana|RMNH.INS.24018|Kazakhstan|BOLD:ACY3807
Stigmella inopinata|RMNH.INS.24291|Russia.Chelyabinskaya Oblast|
Stigmella inopinata|RMNH.INS.24427|Romania.Brasov|BOLD:ACG8923
Stigmella inopinata|RMNH.INS.18489|Romania.Brasov|BOLD:ACG8923
Stigmella stigmaciella|RMNH.INS.29927|United States.Washington|BOLD:ACH1704
Stigmella taeniola|RMNH.INS.24787|Canada.Alberta|BOLD:ACU4017
Stigmella taeniola|RMNH.INS.30724|Canada.Ontario|BOLD:ACU4017
Stigmella Yukon|CNCLEP00026539|Canada.Yukon Territory|BOLD:AAI6168
Stigmella Yukon|RMNH.INS.24231|Canada.Yukon Territory|BOLD:AAI6168
Stigmella stettinensis|RMNH.INS.24621|Czech Republic|BOLD:ACP3946
Stigmella stettinensis|BC ZSM Lep 89423|Germany.Bavaria|BOLD:ACP3946
Stigmella stettinensis|BC ZSM Lep 89422|Germany.Bavaria|BOLD:ACP3946
Stigmella stettinensis|BC ZSM Lep 89201|Germany.Bavaria|BOLD:ACP3946
Stigmella stettinensis|RMNH.INS.24597|Russia.Ulyanovskaya Oblast|BOLD:ACP3946
Stigmella amelanchieriella|RMNH.INS.18275|United States.Tennessee|BOLD:ACG8835
Stigmella amelanchieriella|RMNH.INS.18622|United States.Massachusetts|BOLD:ACG8835
Stigmella crataegifoliella|RMNH.INS.18629|United States.Massachusetts|BOLD:ACG9476
Stigmella crataegifoliella|RMNH.INS.24413|United States.Massachusetts|BOLD:ACG9476
Stigmella crataegifoliella|RMNH.INS.18351|United States.Mississippi|BOLD:ACG9477
Stigmella magdalenae|MM18596|Finland.Northern Ostrobothnia|BOLD:AAP9739
Stigmella magdalenae|RMNH.INS.29955|Russia.Arkhangelskaya Oblast|BOLD:AAP9739
Stigmella magdalenae|MM23363|Finland|BOLD:AAP9739
Stigmella magdalenae|MM23362|Finland|BOLD:AAP9739
Stigmella magdalenae|MM18597|Finland.Northern Ostrobothnia|BOLD:AAP9739
Stigmella magdalenae|MM18598|Finland|BOLD:AAP9739
Stigmella magdalenae|RMNH.INS.30243|Russia.Krasnoyarskiy Kray|BOLD:AAP9739
Stigmella magdalenae|RMNH.INS.18496|Romania|BOLD:ACG9379
Stigmella magdalenae|RMNH.INS.12064|Netherlands.Drenthe|BOLD:ACG9379
Stigmella magdalenae|RMNH.INS.11606|Netherlands.Drenthe|BOLD:ACG9379
Stigmella magdalenae|RMNH.INS.18470|Netherlands.Gelderland|BOLD:ACG9379
Stigmella AmelanchierCO|RMNH.INS.30726|Canada.Ontario|BOLD:ACG5879
Stigmella AmelanchierCO|RMNH.INS.29192|United States.Colorado|BOLD:ACG5879
Stigmella crataegella|RMNH.INS.11798|Sweden|BOLD:ACG8934
Stigmella crataegella|RMNH.INS.12062|Netherlands.Gelderland|BOLD:ACG8934
Stigmella crataegella|RMNH.INS.11918|France.Languedoc-Roussillon|BOLD:ACG8935
Stigmella minusculella|RMNH.INS.30785|Canada.Ontario|BOLD:AAV8371
Stigmella minusculella|KLM Lep 01260|Austria.Carinthia|BOLD:AAV8371
Stigmella minusculella|KLM Lep 01263|Austria.Carinthia|BOLD:AAV8371
Stigmella minusculella|RMNH.INS.17754|Greece|BOLD:AAV8371
Stigmella minusculella|RMNH.INS.23751|Croatia|BOLD:AAV8371
Stigmella minusculella|RMNH.INS.18974|Greece|BOLD:AAV8371
Stigmella nostrata|RMNH.INS.29812|Japan.Honshu|BOLD:ACU5729
Stigmella AroniaSorbus|RMNH.INS.30729|Canada.Ontario|BOLD:ACL4690
Stigmella AroniaSorbus|RMNH.INS.30538|United States.Massachusetts|BOLD:ACL4690
Stigmella nylandriella|MM18599|Finland.Finland Proper|BOLD:AAF3425
Stigmella nylandriella|RMNH.INS.29956|Russia.Arkhangelskaya Oblast|BOLD:AAF3425
Stigmella nylandriella|MM09644|Finland.Aland Islands|BOLD:AAF3425
Stigmella nylandriella|MM07002|Finland|BOLD:AAF3425
Stigmella nylandriella|RMNH.INS.11603|Netherlands.Drenthe|BOLD:AAF3425
Stigmella nylandriella|RMNH.INS.11803|Sweden|BOLD:AAF3425
Stigmella nylandriella|MM07001|Finland|BOLD:AAF3425
Stigmella oxyacanthella|MM09650|Finland.Aland Islands|BOLD:AAF3421
Stigmella oxyacanthella|RMNH.INS.12828|Netherlands.North Brabant|BOLD:AAF3421
Stigmella oxyacanthella|MM00900|Finland.Aland Islands|BOLD:AAF3421
Stigmella oxyacanthella|BC ZSM Lep 89439|Germany.Bavaria|BOLD:AAF3421
Stigmella oxyacanthella|RMNH.INS.18717|United States.New York|BOLD:AAF3421
Stigmella oxyacanthella|RMNH.INS.30544|United States.Massachusetts|BOLD:AAF3421
Stigmella oxyacanthella|BC ZSM Lep 50946|Germany.Bavaria|BOLD:AAF3421
Stigmella oxyacanthella|RMNH.INS.30542|United States.Maine|BOLD:AAF3421
Stigmella oxyacanthella|MM00899|Finland.Aland Islands|BOLD:AAF3421
Stigmella oxyacanthella|RMNH.INS.18711|United States.Massachusetts|BOLD:AAF3421
Stigmella oxyacanthella|RMNH.INS.18532|United States.Massachusetts|BOLD:AAF3421
Stigmella oxyacanthella|RMNH.INS.12639|Netherlands.South Holland|BOLD:AAF3421
Stigmella oxyacanthella|RMNH.INS.24315|Italy.Sardinia|BOLD:AAF3421
Stigmella oxyacanthella|RMNH.INS.18799|Greece|BOLD:AAF3421
Stigmella oxyacanthella|RMNH.INS.12960|France.Provence-Alpes-Cote d'Azur|BOLD:AAF3421
Stigmella micromelis|RMNH.INS.30172|South Korea|BOLD:ACK9547
Stigmella micromelis|RMNH.INS.30245|Russia.Krasnoyarskiy Kray|BOLD:ACK9547
Stigmella micromelis|RMNH.INS.30246|Russia.Krasnoyarskiy Kray|BOLD:ACK9547
Stigmella micromelis|RMNH.INS.29730|Japan.Hokkaido|BOLD:ACK9547
Stigmella micromelis|RMNH.INS.29780|Japan.Hokkaido|BOLD:ACK9547
Stigmella micromelis|RMNH.INS.24659|Japan.Honshu|BOLD:ACK9547
Stigmella micromelis|RMNH.INS.29731|Japan.Hokkaido|BOLD:ACK9547
Stigmella sorbivora|RMNH.INS.29794|Japan.Hokkaido|BOLD:ACU7360
Stigmella pyri|RMNH.INS.23683|Italy.Piedmont|BOLD:AAV8370
Stigmella pyri|RMNH.INS.24428|Netherlands.South Holland|BOLD:AAV8370
Stigmella desperatella|BC ZSM Lep 85572|Germany.Bavaria|BOLD:AAV7003
Stigmella desperatella|RMNH.INS.18479|Romania|BOLD:AAV7003
Stigmella desperatella|BC ZSM Lep 70739|Germany.Bavaria|BOLD:AAV7003

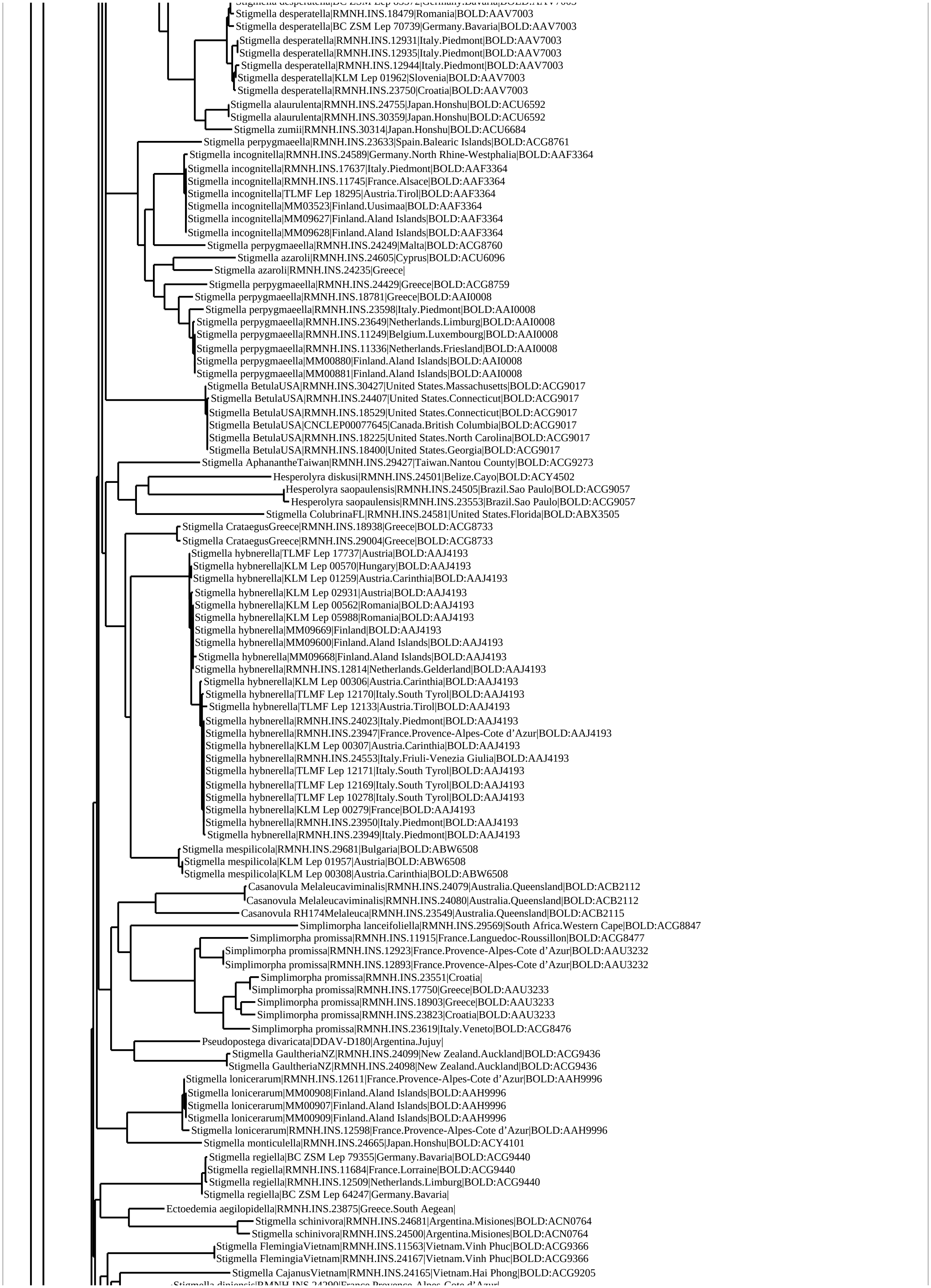
Stigmella desperatella|RMNH.INS.18479|Romania|BOLD:AAV7003
Stigmella desperatella|BC ZSM Lep 70739|Germany.Bavaria|BOLD:AAV7003
Stigmella desperatella|RMNH.INS.12931|Italy.Piedmont|BOLD:AAV7003
Stigmella desperatella|RMNH.INS.12935|Italy.Piedmont|BOLD:AAV7003
Stigmella desperatella|RMNH.INS.12944|Italy.Piedmont|BOLD:AAV7003
Stigmella desperatella|KLM Lep 01962|Slovenia|BOLD:AAV7003
Stigmella desperatella|RMNH.INS.23750|Croatia|BOLD:AAV7003
Stigmella alaurulenta|RMNH.INS.24755|Japan.Honshu|BOLD:ACU6592
Stigmella alaurulenta|RMNH.INS.30359|Japan.Honshu|BOLD:ACU6592
Stigmella zumii|RMNH.INS.30314|Japan.Honshu|BOLD:ACU6684
Stigmella perpygmaeella|RMNH.INS.23633|Spain.Balearic Islands|BOLD:ACG8761
Stigmella incognitella|RMNH.INS.24589|Germany.North Rhine-Westphalia|BOLD:AAF3364
Stigmella incognitella|RMNH.INS.17637|Italy.Piedmont|BOLD:AAF3364
Stigmella incognitella|RMNH.INS.11745|France.Alsace|BOLD:AAF3364
Stigmella incognitella|TLMF Lep 18295|Austria.Tirol|BOLD:AAF3364
Stigmella incognitella|MM03523|Finland.Uusimaa|BOLD:AAF3364
Stigmella incognitella|MM09627|Finland.Aland Islands|BOLD:AAF3364
Stigmella incognitella|MM09628|Finland.Aland Islands|BOLD:AAF3364
Stigmella perpygmaeella|RMNH.INS.24249|Malta|BOLD:ACG8760
Stigmella azaroli|RMNH.INS.24605|Cyprus|BOLD:ACU6096
Stigmella azaroli|RMNH.INS.24235|Greece|
Stigmella perpygmaeella|RMNH.INS.24429|Greece|BOLD:ACG8759
Stigmella perpygmaeella|RMNH.INS.18781|Greece|BOLD:AAI0008
Stigmella perpygmaeella|RMNH.INS.23598|Italy.Piedmont|BOLD:AAI0008
Stigmella perpygmaeella|RMNH.INS.23649|Netherlands.Limburg|BOLD:AAI0008
Stigmella perpygmaeella|RMNH.INS.11249|Belgium.Luxembourg|BOLD:AAI0008
Stigmella perpygmaeella|RMNH.INS.11336|Netherlands.Friesland|BOLD:AAI0008
Stigmella perpygmaeella|MM00880|Finland.Aland Islands|BOLD:AAI0008
Stigmella perpygmaeella|MM00881|Finland.Aland Islands|BOLD:AAI0008
Stigmella BetulaUSA|RMNH.INS.30427|United States.Massachusetts|BOLD:ACG9017
Stigmella BetulaUSA|RMNH.INS.24407|United States.Connecticut|BOLD:ACG9017
Stigmella BetulaUSA|RMNH.INS.18529|United States.Connecticut|BOLD:ACG9017
Stigmella BetulaUSA|CNCLEP00077645|Canada.British Columbia|BOLD:ACG9017
Stigmella BetulaUSA|RMNH.INS.18225|United States.North Carolina|BOLD:ACG9017
Stigmella BetulaUSA|RMNH.INS.18400|United States.Georgia|BOLD:ACG9017
Stigmella AphanantheTaiwan|RMNH.INS.29427|Taiwan.Nantou County|BOLD:ACG9273
Hesperolyra diskusi|RMNH.INS.24501|Belize.Cayo|BOLD:ACY4502
Hesperolyra saopaulensis|RMNH.INS.24505|Brazil.Sao Paulo|BOLD:ACG9057
Hesperolyra saopaulensis|RMNH.INS.23553|Brazil.Sao Paulo|BOLD:ACG9057
Stigmella ColubrinaFL|RMNH.INS.24581|United States.Florida|BOLD:ABX3505
Stigmella CrataegusGreece|RMNH.INS.18938|Greece|BOLD:ACG8733
Stigmella CrataegusGreece|RMNH.INS.29004|Greece|BOLD:ACG8733
Stigmella hybnerella|TLMF Lep 17737|Austria|BOLD:AAJ4193
Stigmella hybnerella|KLM Lep 00570|Hungary|BOLD:AAJ4193
Stigmella hybnerella|KLM Lep 01259|Austria.Carinthia|BOLD:AAJ4193
Stigmella hybnerella|KLM Lep 02931|Austria|BOLD:AAJ4193
Stigmella hybnerella|KLM Lep 00562|Romania|BOLD:AAJ4193
Stigmella hybnerella|KLM Lep 05988|Romania|BOLD:AAJ4193
Stigmella hybnerella|MM09669|Finland|BOLD:AAJ4193
Stigmella hybnerella|MM09600|Finland.Aland Islands|BOLD:AAJ4193
Stigmella hybnerella|MM09668|Finland.Aland Islands|BOLD:AAJ4193
Stigmella hybnerella|RMNH.INS.12814|Netherlands.Gelderland|BOLD:AAJ4193
Stigmella hybnerella|KLM Lep 00306|Austria.Carinthia|BOLD:AAJ4193
Stigmella hybnerella|TLMF Lep 12170|Italy.South Tyrol|BOLD:AAJ4193
Stigmella hybnerella|TLMF Lep 12133|Austria.Tirol|BOLD:AAJ4193
Stigmella hybnerella|RMNH.INS.24023|Italy.Piedmont|BOLD:AAJ4193
Stigmella hybnerella|RMNH.INS.23947|France.Provence-Alpes-Cote d'Azur|BOLD:AAJ4193
Stigmella hybnerella|KLM Lep 00307|Austria.Carinthia|BOLD:AAJ4193
Stigmella hybnerella|RMNH.INS.24553|Italy.Friuli-Venezia Giulia|BOLD:AAJ4193
Stigmella hybnerella|TLMF Lep 12171|Italy.South Tyrol|BOLD:AAJ4193
Stigmella hybnerella|TLMF Lep 12169|Italy.South Tyrol|BOLD:AAJ4193
Stigmella hybnerella|TLMF Lep 10278|Italy.South Tyrol|BOLD:AAJ4193
Stigmella hybnerella|KLM Lep 00279|France|BOLD:AAJ4193
Stigmella hybnerella|RMNH.INS.23950|Italy.Piedmont|BOLD:AAJ4193
Stigmella hybnerella|RMNH.INS.23949|Italy.Piedmont|BOLD:AAJ4193
Stigmella mespilicola|RMNH.INS.29681|Bulgaria|BOLD:ABW6508
Stigmella mespilicola|KLM Lep 01957|Austria|BOLD:ABW6508
Stigmella mespilicola|KLM Lep 00308|Austria.Carinthia|BOLD:ABW6508
Casanovula Melaleucaviminalis|RMNH.INS.24079|Australia.Queensland|BOLD:ACB2112
Casanovula Melaleucaviminalis|RMNH.INS.24080|Australia.Queensland|BOLD:ACB2112
Casanovula RH174Melaleuca|RMNH.INS.23549|Australia.Queensland|BOLD:ACB2115
Simplimorpha lanceifoliella|RMNH.INS.29569|South Africa.Western Cape|BOLD:ACG8847
Simplimorpha promissa|RMNH.INS.11915|France.Languedoc-Roussillon|BOLD:ACG8477
Simplimorpha promissa|RMNH.INS.12923|France.Provence-Alpes-Cote d'Azur|BOLD:AAU3232
Simplimorpha promissa|RMNH.INS.12893|France.Provence-Alpes-Cote d'Azur|BOLD:AAU3232
Simplimorpha promissa|RMNH.INS.23551|Croatia|
Simplimorpha promissa|RMNH.INS.17750|Greece|BOLD:AAU3233
Simplimorpha promissa|RMNH.INS.18903|Greece|BOLD:AAU3233
Simplimorpha promissa|RMNH.INS.23823|Croatia|BOLD:AAU3233
Simplimorpha promissa|RMNH.INS.23619|Italy.Veneto|BOLD:ACG8476
Pseudopostega divaricata|DDAV-D180|Argentina.Jujuy|
Stigmella GaultheriaNZ|RMNH.INS.24099|New Zealand.Auckland|BOLD:ACG9436
Stigmella GaultheriaNZ|RMNH.INS.24098|New Zealand.Auckland|BOLD:ACG9436
Stigmella lonicerarum|RMNH.INS.12611|France.Provence-Alpes-Cote d'Azur|BOLD:AAH9996
Stigmella lonicerarum|MM00908|Finland.Aland Islands|BOLD:AAH9996
Stigmella lonicerarum|MM00907|Finland.Aland Islands|BOLD:AAH9996
Stigmella lonicerarum|MM00909|Finland.Aland Islands|BOLD:AAH9996
Stigmella lonicerarum|RMNH.INS.12598|France.Provence-Alpes-Cote d'Azur|BOLD:AAH9996
Stigmella monticulella|RMNH.INS.24665|Japan.Honshu|BOLD:ACY4101
Stigmella regiella|BC ZSM Lep 79355|Germany.Bavaria|BOLD:ACG9440
Stigmella regiella|RMNH.INS.11684|France.Lorraine|BOLD:ACG9440
Stigmella regiella|RMNH.INS.12509|Netherlands.Limburg|BOLD:ACG9440
Stigmella regiella|BC ZSM Lep 64247|Germany.Bavaria|
Ectoedemia aegilopidella|RMNH.INS.23875|Greece.South Aegean|
Stigmella schinivora|RMNH.INS.24681|Argentina.Misiones|BOLD:ACN0764
Stigmella schinivora|RMNH.INS.24500|Argentina.Misiones|BOLD:ACN0764
Stigmella FlemingiaVietnam|RMNH.INS.11563|Vietnam.Vinh Phuc|BOLD:ACG9366
Stigmella FlemingiaVietnam|RMNH.INS.24167|Vietnam.Vinh Phuc|BOLD:ACG9366
Stigmella CajanusVietnam|RMNH.INS.24165|Vietnam.Hai Phong|BOLD:ACG9205
Stigmella diniensis|RMNH.INS.24290|France.Provence-Alpes-Cote d'Azur|

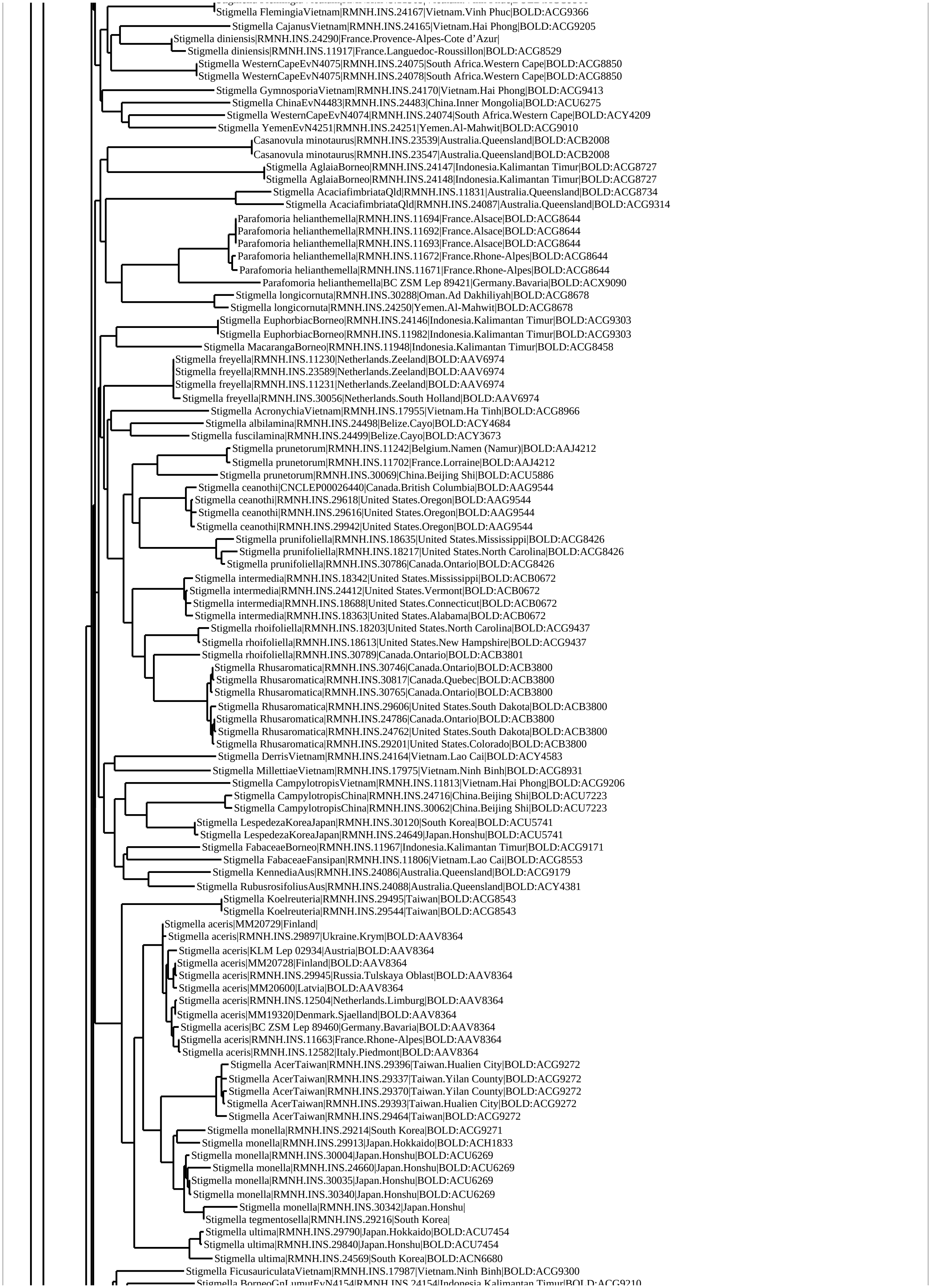
Stigmella FlemingiaVietnam|RMNH.INS.24167|Vietnam.Vinh Phuc|BOLD:ACG9366
Stigmella CajanusVietnam|RMNH.INS.24165|Vietnam.Hai Phong|BOLD:ACG9205
Stigmella diniensis|RMNH.INS.24290|France.Provence-Alpes-Cote d'Azur|
Stigmella diniensis|RMNH.INS.11917|France.Languedoc-Roussillon|BOLD:ACG8529
Stigmella WesternCapeEvN4075|RMNH.INS.24075|South Africa.Western Cape|BOLD:ACG8850
Stigmella WesternCapeEvN4075|RMNH.INS.24078|South Africa.Western Cape|BOLD:ACG8850
Stigmella GymnosporiaVietnam|RMNH.INS.24170|Vietnam.Hai Phong|BOLD:ACG9413
Stigmella ChinaEvN4483|RMNH.INS.24483|China.Inner Mongolia|BOLD:ACU6275
Stigmella WesternCapeEvN4074|RMNH.INS.24074|South Africa.Western Cape|BOLD:ACY4209
Stigmella YemenEvN4251|RMNH.INS.24251|Yemen.Al-Mahwit|BOLD:ACG9010
Casanovula minotaurus|RMNH.INS.23539|Australia.Queensland|BOLD:ACB2008
Casanovula minotaurus|RMNH.INS.23547|Australia.Queensland|BOLD:ACB2008
Stigmella AglaiaBorneo|RMNH.INS.24147|Indonesia.Kalimantan Timur|BOLD:ACG8727
Stigmella AglaiaBorneo|RMNH.INS.24148|Indonesia.Kalimantan Timur|BOLD:ACG8727
Stigmella AcaciafimbriataQld|RMNH.INS.11831|Australia.Queensland|BOLD:ACG8734
Stigmella AcaciafimbriataQld|RMNH.INS.24087|Australia.Queensland|BOLD:ACG9314
Parafomoria helianthemella|RMNH.INS.11694|France.Alsace|BOLD:ACG8644
Parafomoria helianthemella|RMNH.INS.11692|France.Alsace|BOLD:ACG8644
Parafomoria helianthemella|RMNH.INS.11693|France.Alsace|BOLD:ACG8644
Parafomoria helianthemella|RMNH.INS.11672|France.Rhone-Alpes|BOLD:ACG8644
Parafomoria helianthemella|RMNH.INS.11671|France.Rhone-Alpes|BOLD:ACG8644
Parafomoria helianthemella|BC ZSM Lep 89421|Germany.Bavaria|BOLD:ACX9090
Stigmella longicornuta|RMNH.INS.30288|Oman.Ad Dakhiliyah|BOLD:ACG8678
Stigmella longicornuta|RMNH.INS.24250|Yemen.Al-Mahwit|BOLD:ACG8678
Stigmella EuphorbiacBorneo|RMNH.INS.24146|Indonesia.Kalimantan Timur|BOLD:ACG9303
Stigmella EuphorbiacBorneo|RMNH.INS.11982|Indonesia.Kalimantan Timur|BOLD:ACG9303
Stigmella MacarangaBorneo|RMNH.INS.11948|Indonesia.Kalimantan Timur|BOLD:ACG8458
Stigmella freyella|RMNH.INS.11230|Netherlands.Zeeland|BOLD:AAV6974
Stigmella freyella|RMNH.INS.23589|Netherlands.Zeeland|BOLD:AAV6974
Stigmella freyella|RMNH.INS.11231|Netherlands.Zeeland|BOLD:AAV6974
Stigmella freyella|RMNH.INS.30056|Netherlands.South Holland|BOLD:AAV6974
Stigmella AcronychiaVietnam|RMNH.INS.17955|Vietnam.Ha Tinh|BOLD:ACG8966
Stigmella albilamina|RMNH.INS.24498|Belize.Cayo|BOLD:ACY4684
Stigmella fuscilamina|RMNH.INS.24499|Belize.Cayo|BOLD:ACY3673
Stigmella prunetorum|RMNH.INS.11242|Belgium.Namen (Namur)|BOLD:AAJ4212
Stigmella prunetorum|RMNH.INS.11702|France.Lorraine|BOLD:AAJ4212
Stigmella prunetorum|RMNH.INS.30069|China.Beijing Shi|BOLD:ACU5886
Stigmella ceanothi|CNCLEP00026440|Canada.British Columbia|BOLD:AAG9544
Stigmella ceanothi|RMNH.INS.29618|United States.Oregon|BOLD:AAG9544
Stigmella ceanothi|RMNH.INS.29616|United States.Oregon|BOLD:AAG9544
Stigmella ceanothi|RMNH.INS.29942|United States.Oregon|BOLD:AAG9544
Stigmella prunifoliella|RMNH.INS.18635|United States.Mississippi|BOLD:ACG8426
Stigmella prunifoliella|RMNH.INS.18217|United States.North Carolina|BOLD:ACG8426
Stigmella prunifoliella|RMNH.INS.30786|Canada.Ontario|BOLD:ACG8426
Stigmella intermedia|RMNH.INS.18342|United States.Mississippi|BOLD:ACB0672
Stigmella intermedia|RMNH.INS.24412|United States.Vermont|BOLD:ACB0672
Stigmella intermedia|RMNH.INS.18688|United States.Connecticut|BOLD:ACB0672
Stigmella intermedia|RMNH.INS.18363|United States.Alabama|BOLD:ACB0672
Stigmella rhoifoliella|RMNH.INS.18203|United States.North Carolina|BOLD:ACG9437
Stigmella rhoifoliella|RMNH.INS.18613|United States.New Hampshire|BOLD:ACG9437
Stigmella rhoifoliella|RMNH.INS.30789|Canada.Ontario|BOLD:ACB3801
Stigmella Rhusaromatica|RMNH.INS.30746|Canada.Ontario|BOLD:ACB3800
Stigmella Rhusaromatica|RMNH.INS.30817|Canada.Quebec|BOLD:ACB3800
Stigmella Rhusaromatica|RMNH.INS.30765|Canada.Ontario|BOLD:ACB3800
Stigmella Rhusaromatica|RMNH.INS.29606|United States.South Dakota|BOLD:ACB3800
Stigmella Rhusaromatica|RMNH.INS.24786|Canada.Ontario|BOLD:ACB3800
Stigmella Rhusaromatica|RMNH.INS.24762|United States.South Dakota|BOLD:ACB3800
Stigmella Rhusaromatica|RMNH.INS.29201|United States.Colorado|BOLD:ACB3800
Stigmella DerrisVietnam|RMNH.INS.24164|Vietnam.Lao Cai|BOLD:ACY4583
Stigmella MillettiaeVietnam|RMNH.INS.17975|Vietnam.Ninh Binh|BOLD:ACG8931
Stigmella CampylotropisVietnam|RMNH.INS.11813|Vietnam.Hai Phong|BOLD:ACG9206
Stigmella CampylotropisChina|RMNH.INS.24716|China.Beijing Shi|BOLD:ACU7223
Stigmella CampylotropisChina|RMNH.INS.30062|China.Beijing Shi|BOLD:ACU7223
Stigmella LespedezaKoreaJapan|RMNH.INS.30120|South Korea|BOLD:ACU5741
Stigmella LespedezaKoreaJapan|RMNH.INS.24649|Japan.Honshu|BOLD:ACU5741
Stigmella FabaceaeBorneo|RMNH.INS.11967|Indonesia.Kalimantan Timur|BOLD:ACG9171
Stigmella FabaceaeFansipan|RMNH.INS.11806|Vietnam.Lao Cai|BOLD:ACG8553
Stigmella KennediaAus|RMNH.INS.24086|Australia.Queensland|BOLD:ACG9179
Stigmella RubusrosifoliusAus|RMNH.INS.24088|Australia.Queensland|BOLD:ACY4381
Stigmella Koelreuteria|RMNH.INS.29495|Taiwan|BOLD:ACG8543
Stigmella Koelreuteria|RMNH.INS.29544|Taiwan|BOLD:ACG8543
Stigmella aceris|MM20729|Finland|
Stigmella aceris|RMNH.INS.29897|Ukraine.Krym|BOLD:AAV8364
Stigmella aceris|KLM Lep 02934|Austria|BOLD:AAV8364
Stigmella aceris|MM20728|Finland|BOLD:AAV8364
Stigmella aceris|RMNH.INS.29945|Russia.Tulskaya Oblast|BOLD:AAV8364
Stigmella aceris|MM20600|Latvia|BOLD:AAV8364
Stigmella aceris|RMNH.INS.12504|Netherlands.Limburg|BOLD:AAV8364
Stigmella aceris|MM19320|Denmark.Sjaelland|BOLD:AAV8364
Stigmella aceris|BC ZSM Lep 89460|Germany.Bavaria|BOLD:AAV8364
Stigmella aceris|RMNH.INS.11663|France.Rhone-Alpes|BOLD:AAV8364
Stigmella aceris|RMNH.INS.12582|Italy.Piedmont|BOLD:AAV8364
Stigmella AcerTaiwan|RMNH.INS.29396|Taiwan.Hualien City|BOLD:ACG9272
Stigmella AcerTaiwan|RMNH.INS.29337|Taiwan.Yilan County|BOLD:ACG9272
Stigmella AcerTaiwan|RMNH.INS.29370|Taiwan.Yilan County|BOLD:ACG9272
Stigmella AcerTaiwan|RMNH.INS.29393|Taiwan.Hualien City|BOLD:ACG9272
Stigmella AcerTaiwan|RMNH.INS.29464|Taiwan|BOLD:ACG9272
Stigmella monella|RMNH.INS.29214|South Korea|BOLD:ACG9271
Stigmella monella|RMNH.INS.29913|Japan.Hokkaido|BOLD:ACH1833
Stigmella monella|RMNH.INS.30004|Japan.Honshu|BOLD:ACU6269
Stigmella monella|RMNH.INS.24660|Japan.Honshu|BOLD:ACU6269
Stigmella monella|RMNH.INS.30035|Japan.Honshu|BOLD:ACU6269
Stigmella monella|RMNH.INS.30340|Japan.Honshu|BOLD:ACU6269
Stigmella monella|RMNH.INS.30342|Japan.Honshu|
Stigmella tegmentosella|RMNH.INS.29216|South Korea|
Stigmella ultima|RMNH.INS.29790|Japan.Hokkaido|BOLD:ACU7454
Stigmella ultima|RMNH.INS.29840|Japan.Honshu|BOLD:ACU7454
Stigmella ultima|RMNH.INS.24569|South Korea|BOLD:ACN6680
Stigmella FicusauriculataVietnam|RMNH.INS.17987|Vietnam.Ninh Binh|BOLD:ACG9300
Stigmella BorneoGnLumutEvN4154|RMNH.INS.24154|Indonesia.Kalimantan Timur|BOLD:ACG9210

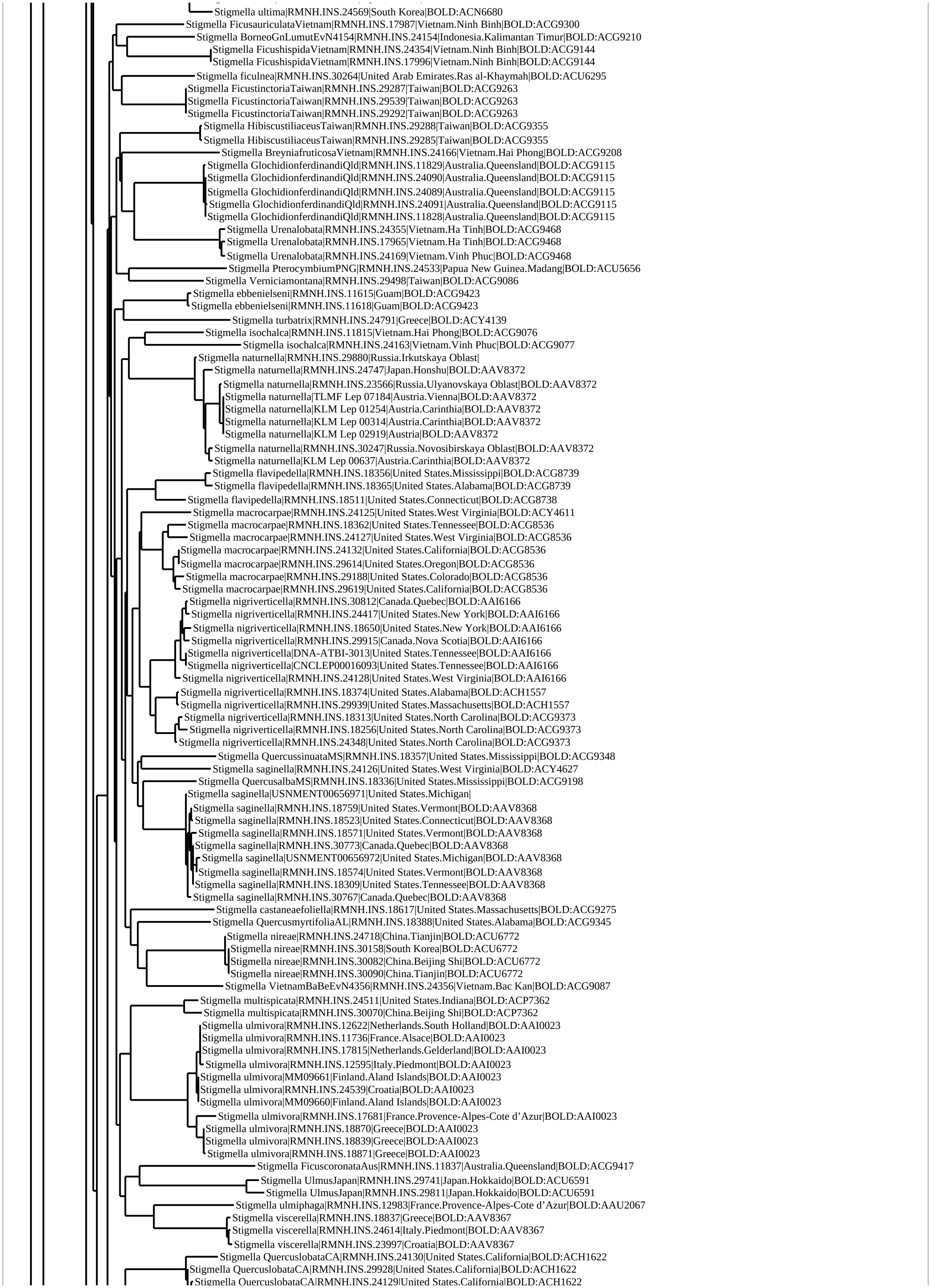
Stigmella ultima|RMNH.INS.24569|South Korea|BOLD:ACN6680
Stigmella FicusauriculataVietnam|RMNH.INS.17987|Vietnam.Ninh Binh|BOLD:ACG9300
Stigmella BorneoGnLumutEvN4154|RMNH.INS.24154|Indonesia.Kalimantan Timur|BOLD:ACG9210
Stigmella FicushispidaVietnam|RMNH.INS.24354|Vietnam.Ninh Binh|BOLD:ACG9144
Stigmella FicushispidaVietnam|RMNH.INS.17996|Vietnam.Ninh Binh|BOLD:ACG9144
Stigmella ficulnea|RMNH.INS.30264|United Arab Emirates.Ras al-Khaymah|BOLD:ACU6295
Stigmella FicustinctoriaTaiwan|RMNH.INS.29287|Taiwan|BOLD:ACG9263
Stigmella FicustinctoriaTaiwan|RMNH.INS.29539|Taiwan|BOLD:ACG9263
Stigmella FicustinctoriaTaiwan|RMNH.INS.29292|Taiwan|BOLD:ACG9263
Stigmella HibiscustiliaceusTaiwan|RMNH.INS.29288|Taiwan|BOLD:ACG9355
Stigmella HibiscustiliaceusTaiwan|RMNH.INS.29285|Taiwan|BOLD:ACG9355
Stigmella BreyniafruticosaVietnam|RMNH.INS.24166|Vietnam.Hai Phong|BOLD:ACG9208
Stigmella GlochidionferdinandiQld|RMNH.INS.11829|Australia.Queensland|BOLD:ACG9115
Stigmella GlochidionferdinandiQld|RMNH.INS.24090|Australia.Queensland|BOLD:ACG9115
Stigmella GlochidionferdinandiQld|RMNH.INS.24089|Australia.Queensland|BOLD:ACG9115
Stigmella GlochidionferdinandiQld|RMNH.INS.24091|Australia.Queensland|BOLD:ACG9115
Stigmella GlochidionferdinandiQld|RMNH.INS.11828|Australia.Queensland|BOLD:ACG9115
Stigmella Urenalobata|RMNH.INS.24355|Vietnam.Ha Tinh|BOLD:ACG9468
Stigmella Urenalobata|RMNH.INS.17965|Vietnam.Ha Tinh|BOLD:ACG9468
Stigmella Urenalobata|RMNH.INS.24169|Vietnam.Vinh Phuc|BOLD:ACG9468
Stigmella PterocymbiumPNG|RMNH.INS.24533|Papua New Guinea.Madang|BOLD:ACU5656
Stigmella Verniciamontana|RMNH.INS.29498|Taiwan|BOLD:ACG9086
Stigmella ebbenielseni|RMNH.INS.11615|Guam|BOLD:ACG9423
Stigmella ebbenielseni|RMNH.INS.11618|Guam|BOLD:ACG9423
Stigmella turbatrix|RMNH.INS.24791|Greece|BOLD:ACY4139
Stigmella isochalca|RMNH.INS.11815|Vietnam.Hai Phong|BOLD:ACG9076
Stigmella isochalca|RMNH.INS.24163|Vietnam.Vinh Phuc|BOLD:ACG9077
Stigmella naturnella|RMNH.INS.29880|Russia.Irkutskaya Oblast|
Stigmella naturnella|RMNH.INS.24747|Japan.Honshu|BOLD:AAV8372
Stigmella naturnella|RMNH.INS.23566|Russia.Ulyanovskaya Oblast|BOLD:AAV8372
Stigmella naturnella|TLMF Lep 07184|Austria.Vienna|BOLD:AAV8372
Stigmella naturnella|KLM Lep 01254|Austria.Carinthia|BOLD:AAV8372
Stigmella naturnella|KLM Lep 00314|Austria.Carinthia|BOLD:AAV8372
Stigmella naturnella|KLM Lep 02919|Austria|BOLD:AAV8372
Stigmella naturnella|RMNH.INS.30247|Russia.Novosibirskaya Oblast|BOLD:AAV8372
Stigmella naturnella|KLM Lep 00637|Austria.Carinthia|BOLD:AAV8372
Stigmella flavipedella|RMNH.INS.18356|United States.Mississippi|BOLD:ACG8739
Stigmella flavipedella|RMNH.INS.18365|United States.Alabama|BOLD:ACG8739
Stigmella flavipedella|RMNH.INS.18511|United States.Connecticut|BOLD:ACG8738
Stigmella macrocarpae|RMNH.INS.24125|United States.West Virginia|BOLD:ACY4611
Stigmella macrocarpae|RMNH.INS.18362|United States.Tennessee|BOLD:ACG8536
Stigmella macrocarpae|RMNH.INS.24127|United States.West Virginia|BOLD:ACG8536
Stigmella macrocarpae|RMNH.INS.24132|United States.California|BOLD:ACG8536
Stigmella macrocarpae|RMNH.INS.29614|United States.Oregon|BOLD:ACG8536
Stigmella macrocarpae|RMNH.INS.29188|United States.Colorado|BOLD:ACG8536
Stigmella macrocarpae|RMNH.INS.29619|United States.California|BOLD:ACG8536
Stigmella nigriverticella|RMNH.INS.30812|Canada.Quebec|BOLD:AAI6166
Stigmella nigriverticella|RMNH.INS.24417|United States.New York|BOLD:AAI6166
Stigmella nigriverticella|RMNH.INS.18650|United States.New York|BOLD:AAI6166
Stigmella nigriverticella|RMNH.INS.29915|Canada.Nova Scotia|BOLD:AAI6166
Stigmella nigriverticella|DNA-ATBI-3013|United States.Tennessee|BOLD:AAI6166
Stigmella nigriverticella|CNCLEP00016093|United States.Tennessee|BOLD:AAI6166
Stigmella nigriverticella|RMNH.INS.24128|United States.West Virginia|BOLD:AAI6166
Stigmella nigriverticella|RMNH.INS.18374|United States.Alabama|BOLD:ACH1557
Stigmella nigriverticella|RMNH.INS.29939|United States.Massachusetts|BOLD:ACH1557
Stigmella nigriverticella|RMNH.INS.18313|United States.North Carolina|BOLD:ACG9373
Stigmella nigriverticella|RMNH.INS.18256|United States.North Carolina|BOLD:ACG9373
Stigmella nigriverticella|RMNH.INS.24348|United States.North Carolina|BOLD:ACG9373
Stigmella QuercussinuataMS|RMNH.INS.18357|United States.Mississippi|BOLD:ACG9348
Stigmella saginella|RMNH.INS.24126|United States.West Virginia|BOLD:ACY4627
Stigmella QuercusalbaMS|RMNH.INS.18336|United States.Mississippi|BOLD:ACG9198
Stigmella saginella|USNMENT00656971|United States.Michigan|
Stigmella saginella|RMNH.INS.18759|United States.Vermont|BOLD:AAV8368
Stigmella saginella|RMNH.INS.18523|United States.Connecticut|BOLD:AAV8368
Stigmella saginella|RMNH.INS.18571|United States.Vermont|BOLD:AAV8368
Stigmella saginella|RMNH.INS.30773|Canada.Quebec|BOLD:AAV8368
Stigmella saginella|USNMENT00656972|United States.Michigan|BOLD:AAV8368
Stigmella saginella|RMNH.INS.18574|United States.Vermont|BOLD:AAV8368
Stigmella saginella|RMNH.INS.18309|United States.Tennessee|BOLD:AAV8368
Stigmella saginella|RMNH.INS.30767|Canada.Quebec|BOLD:AAV8368
Stigmella castaneaefoliella|RMNH.INS.18617|United States.Massachusetts|BOLD:ACG9275
Stigmella QuercusmyrtifoliaAL|RMNH.INS.18388|United States.Alabama|BOLD:ACG9345
Stigmella nireae|RMNH.INS.24718|China.Tianjin|BOLD:ACU6772
Stigmella nireae|RMNH.INS.30158|South Korea|BOLD:ACU6772
Stigmella nireae|RMNH.INS.30082|China.Beijing Shi|BOLD:ACU6772
Stigmella nireae|RMNH.INS.30090|China.Tianjin|BOLD:ACU6772
Stigmella VietnamBaBeEvN4356|RMNH.INS.24356|Vietnam.Bac Kan|BOLD:ACG9087
Stigmella multispicata|RMNH.INS.24511|United States.Indiana|BOLD:ACP7362
Stigmella multispicata|RMNH.INS.30070|China.Beijing Shi|BOLD:ACP7362
Stigmella ulmivora|RMNH.INS.12622|Netherlands.South Holland|BOLD:AAI0023
Stigmella ulmivora|RMNH.INS.11736|France.Alsace|BOLD:AAI0023
Stigmella ulmivora|RMNH.INS.17815|Netherlands.Gelderland|BOLD:AAI0023
Stigmella ulmivora|RMNH.INS.12595|Italy.Piedmont|BOLD:AAI0023
Stigmella ulmivora|MM09661|Finland.Aland Islands|BOLD:AAI0023
Stigmella ulmivora|RMNH.INS.24539|Croatia|BOLD:AAI0023
Stigmella ulmivora|MM09660|Finland.Aland Islands|BOLD:AAI0023
Stigmella ulmivora|RMNH.INS.17681|France.Provence-Alpes-Cote d'Azur|BOLD:AAI0023
Stigmella ulmivora|RMNH.INS.18870|Greece|BOLD:AAI0023
Stigmella ulmivora|RMNH.INS.18839|Greece|BOLD:AAI0023
Stigmella ulmivora|RMNH.INS.18871|Greece|BOLD:AAI0023
Stigmella FicuscoronataAus|RMNH.INS.11837|Australia.Queensland|BOLD:ACG9417
Stigmella UlmusJapan|RMNH.INS.29741|Japan.Hokkaido|BOLD:ACU6591
Stigmella UlmusJapan|RMNH.INS.29811|Japan.Hokkaido|BOLD:ACU6591
Stigmella ulmiphaga|RMNH.INS.12983|France.Provence-Alpes-Cote d'Azur|BOLD:AAU2067
Stigmella viscerella|RMNH.INS.18837|Greece|BOLD:AAV8367
Stigmella viscerella|RMNH.INS.24614|Italy.Piedmont|BOLD:AAV8367
Stigmella viscerella|RMNH.INS.23997|Croatia|BOLD:AAV8367
Stigmella QuercuslobataCA|RMNH.INS.24130|United States.California|BOLD:ACH1622
Stigmella QuercuslobataCA|RMNH.INS.29928|United States.California|BOLD:ACH1622
Stigmella QuercuslobataCA|RMNH.INS.24129|United States.California|BOLD:ACH1622

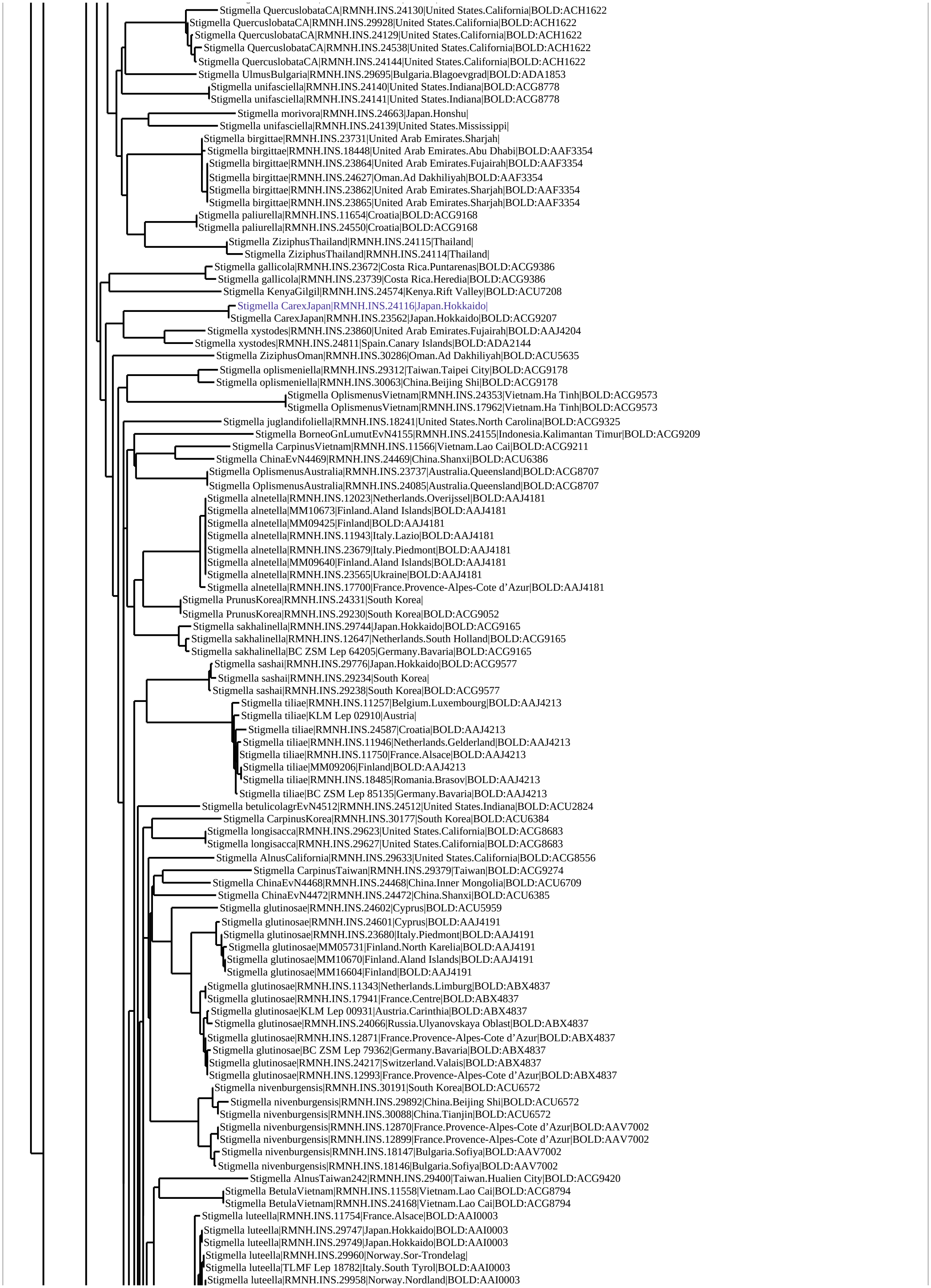
Stigmella QuercuslobataCA|RMNH.INS.24130|United States.California|BOLD:ACH1622
Stigmella QuercuslobataCA|RMNH.INS.29928|United States.California|BOLD:ACH1622
Stigmella QuercuslobataCA|RMNH.INS.24129|United States.California|BOLD:ACH1622
Stigmella QuercuslobataCA|RMNH.INS.24538|United States.California|BOLD:ACH1622
Stigmella QuercuslobataCA|RMNH.INS.24144|United States.California|BOLD:ACH1622
Stigmella UlmusBulgaria|RMNH.INS.29695|Bulgaria.Blagoevgrad|BOLD:ADA1853
Stigmella unifasciella|RMNH.INS.24140|United States.Indiana|BOLD:ACG8778
Stigmella unifasciella|RMNH.INS.24141|United States.Indiana|BOLD:ACG8778
Stigmella morivora|RMNH.INS.24663|Japan.Honshu|
Stigmella unifasciella|RMNH.INS.24139|United States.Mississippi|
Stigmella birgittae|RMNH.INS.23731|United Arab Emirates.Sharjah|
Stigmella birgittae|RMNH.INS.18448|United Arab Emirates.Abu Dhabi|BOLD:AAF3354
Stigmella birgittae|RMNH.INS.23864|United Arab Emirates.Fujairah|BOLD:AAF3354
Stigmella birgittae|RMNH.INS.24627|Oman.Ad Dakhiliyah|BOLD:AAF3354
Stigmella birgittae|RMNH.INS.23862|United Arab Emirates.Sharjah|BOLD:AAF3354
Stigmella birgittae|RMNH.INS.23865|United Arab Emirates.Sharjah|BOLD:AAF3354
Stigmella paliurella|RMNH.INS.11654|Croatia|BOLD:ACG9168
Stigmella paliurella|RMNH.INS.24550|Croatia|BOLD:ACG9168
Stigmella ZiziphusThailand|RMNH.INS.24115|Thailand|
Stigmella ZiziphusThailand|RMNH.INS.24114|Thailand|
Stigmella gallicola|RMNH.INS.23672|Costa Rica.Puntarenas|BOLD:ACG9386
Stigmella gallicola|RMNH.INS.23739|Costa Rica.Heredia|BOLD:ACG9386
Stigmella KenyaGilgil|RMNH.INS.24574|Kenya.Rift Valley|BOLD:ACU7208
Stigmella CarexJapan|RMNH.INS.24116|Japan.Hokkaido|
Stigmella CarexJapan|RMNH.INS.23562|Japan.Hokkaido|BOLD:ACG9207
Stigmella xystodes|RMNH.INS.23860|United Arab Emirates.Fujairah|BOLD:AAJ4204
Stigmella xystodes|RMNH.INS.24811|Spain.Canary Islands|BOLD:ADA2144
Stigmella ZiziphusOman|RMNH.INS.30286|Oman.Ad Dakhiliyah|BOLD:ACU5635
Stigmella oplismeniella|RMNH.INS.29312|Taiwan.Taipei City|BOLD:ACG9178
Stigmella oplismeniella|RMNH.INS.30063|China.Beijing Shi|BOLD:ACG9178
Stigmella OplismenusVietnam|RMNH.INS.24353|Vietnam.Ha Tinh|BOLD:ACG9573
Stigmella OplismenusVietnam|RMNH.INS.17962|Vietnam.Ha Tinh|BOLD:ACG9573
Stigmella juglandifoliella|RMNH.INS.18241|United States.North Carolina|BOLD:ACG9325
Stigmella BorneoGnLumutEvN4155|RMNH.INS.24155|Indonesia.Kalimantan Timur|BOLD:ACG9209
Stigmella CarpinusVietnam|RMNH.INS.11566|Vietnam.Lao Cai|BOLD:ACG9211
Stigmella ChinaEvN4469|RMNH.INS.24469|China.Shanxi|BOLD:ACU6386
Stigmella OplismenusAustralia|RMNH.INS.23737|Australia.Queensland|BOLD:ACG8707
Stigmella OplismenusAustralia|RMNH.INS.24085|Australia.Queensland|BOLD:ACG8707
Stigmella alnetella|RMNH.INS.12023|Netherlands.Overijssel|BOLD:AAJ4181
Stigmella alnetella|MM10673|Finland.Aland Islands|BOLD:AAJ4181
Stigmella alnetella|MM09425|Finland|BOLD:AAJ4181
Stigmella alnetella|RMNH.INS.11943|Italy.Lazio|BOLD:AAJ4181
Stigmella alnetella|RMNH.INS.23679|Italy.Piedmont|BOLD:AAJ4181
Stigmella alnetella|MM09640|Finland.Aland Islands|BOLD:AAJ4181
Stigmella alnetella|RMNH.INS.23565|Ukraine|BOLD:AAJ4181
Stigmella alnetella|RMNH.INS.17700|France.Provence-Alpes-Cote d'Azur|BOLD:AAJ4181
Stigmella PrunusKorea|RMNH.INS.24331|South Korea|
Stigmella PrunusKorea|RMNH.INS.29230|South Korea|BOLD:ACG9052
Stigmella sakhalinella|RMNH.INS.29744|Japan.Hokkaido|BOLD:ACG9165
Stigmella sakhalinella|RMNH.INS.12647|Netherlands.South Holland|BOLD:ACG9165
Stigmella sakhalinella|BC ZSM Lep 64205|Germany.Bavaria|BOLD:ACG9165
Stigmella sashai|RMNH.INS.29776|Japan.Hokkaido|BOLD:ACG9577
Stigmella sashai|RMNH.INS.29234|South Korea|
Stigmella sashai|RMNH.INS.29238|South Korea|BOLD:ACG9577
Stigmella tiliae|RMNH.INS.11257|Belgium.Luxembourg|BOLD:AAJ4213
Stigmella tiliae|KLM Lep 02910|Austria|
Stigmella tiliae|RMNH.INS.24587|Croatia|BOLD:AAJ4213
Stigmella tiliae|RMNH.INS.11946|Netherlands.Gelderland|BOLD:AAJ4213
Stigmella tiliae|RMNH.INS.11750|France.Alsace|BOLD:AAJ4213
Stigmella tiliae|MM09206|Finland|BOLD:AAJ4213
Stigmella tiliae|RMNH.INS.18485|Romania.Brasov|BOLD:AAJ4213
Stigmella tiliae|BC ZSM Lep 85135|Germany.Bavaria|BOLD:AAJ4213
Stigmella betulicolagrEvN4512|RMNH.INS.24512|United States.Indiana|BOLD:ACU2824
Stigmella CarpinusKorea|RMNH.INS.30177|South Korea|BOLD:ACU6384
Stigmella longisacca|RMNH.INS.29623|United States.California|BOLD:ACG8683
Stigmella longisacca|RMNH.INS.29627|United States.California|BOLD:ACG8683
Stigmella AlnusCalifornia|RMNH.INS.29633|United States.California|BOLD:ACG8556
Stigmella CarpinusTaiwan|RMNH.INS.29379|Taiwan|BOLD:ACG9274
Stigmella ChinaEvN4468|RMNH.INS.24468|China.Inner Mongolia|BOLD:ACU6709
Stigmella ChinaEvN4472|RMNH.INS.24472|China.Shanxi|BOLD:ACU6385
Stigmella glutinosae|RMNH.INS.24602|Cyprus|BOLD:ACU5959
Stigmella glutinosae|RMNH.INS.24601|Cyprus|BOLD:AAJ4191
Stigmella glutinosae|RMNH.INS.23680|Italy.Piedmont|BOLD:AAJ4191
Stigmella glutinosae|MM05731|Finland.North Karelia|BOLD:AAJ4191
Stigmella glutinosae|MM10670|Finland.Aland Islands|BOLD:AAJ4191
Stigmella glutinosae|MM16604|Finland|BOLD:AAJ4191
Stigmella glutinosae|RMNH.INS.11343|Netherlands.Limburg|BOLD:ABX4837
Stigmella glutinosae|RMNH.INS.17941|France.Centre|BOLD:ABX4837
Stigmella glutinosae|KLM Lep 00931|Austria.Carinthia|BOLD:ABX4837
Stigmella glutinosae|RMNH.INS.24066|Russia.Ulyanovskaya Oblast|BOLD:ABX4837
Stigmella glutinosae|RMNH.INS.12871|France.Provence-Alpes-Cote d'Azur|BOLD:ABX4837
Stigmella glutinosae|BC ZSM Lep 79362|Germany.Bavaria|BOLD:ABX4837
Stigmella glutinosae|RMNH.INS.24217|Switzerland.Valais|BOLD:ABX4837
Stigmella glutinosae|RMNH.INS.12993|France.Provence-Alpes-Cote d'Azur|BOLD:ABX4837
Stigmella nivenburgensis|RMNH.INS.30191|South Korea|BOLD:ACU6572
Stigmella nivenburgensis|RMNH.INS.29892|China.Beijing Shi|BOLD:ACU6572
Stigmella nivenburgensis|RMNH.INS.30088|China.Tianjin|BOLD:ACU6572
Stigmella nivenburgensis|RMNH.INS.12870|France.Provence-Alpes-Cote d'Azur|BOLD:AAV7002
Stigmella nivenburgensis|RMNH.INS.12899|France.Provence-Alpes-Cote d'Azur|BOLD:AAV7002
Stigmella nivenburgensis|RMNH.INS.18147|Bulgaria.Sofiya|BOLD:AAV7002
Stigmella nivenburgensis|RMNH.INS.18146|Bulgaria.Sofiya|BOLD:AAV7002
Stigmella AlnusTaiwan242|RMNH.INS.29400|Taiwan.Hualien City|BOLD:ACG9420
Stigmella BetulaVietnam|RMNH.INS.11558|Vietnam.Lao Cai|BOLD:ACG8794
Stigmella BetulaVietnam|RMNH.INS.24168|Vietnam.Lao Cai|BOLD:ACG8794
Stigmella luteella|RMNH.INS.11754|France.Alsace|BOLD:AAI0003
Stigmella luteella|RMNH.INS.29747|Japan.Hokkaido|BOLD:AAI0003
Stigmella luteella|RMNH.INS.29749|Japan.Hokkaido|BOLD:AAI0003
Stigmella luteella|RMNH.INS.29960|Norway.Sor-Trondelag|
Stigmella luteella|TLMF Lep 18782|Italy.South Tyrol|BOLD:AAI0003
Stigmella luteella|RMNH.INS.29958|Norway.Nordland|BOLD:AAI0003

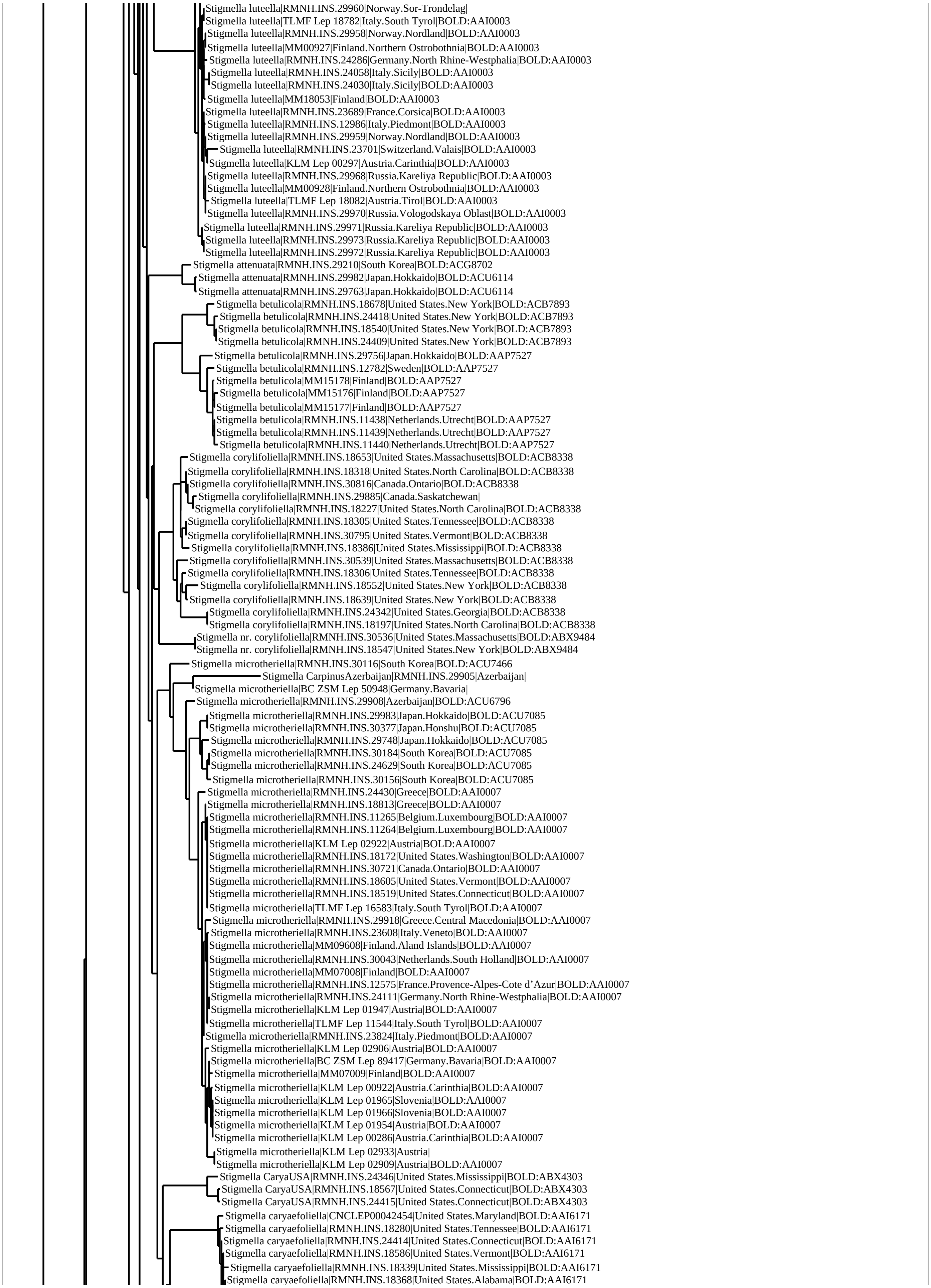
Stigmella luteella|RMNH.INS.29960|Norway.Sor-Trondelag|
Stigmella luteella|TLMF Lep 18782|Italy.South Tyrol|BOLD:AAI0003
Stigmella luteella|RMNH.INS.29958|Norway.Nordland|BOLD:AAI0003
Stigmella luteella|MM00927|Finland.Northern Ostrobothnia|BOLD:AAI0003
Stigmella luteella|RMNH.INS.24286|Germany.North Rhine-Westphalia|BOLD:AAI0003
Stigmella luteella|RMNH.INS.24058|Italy.Sicily|BOLD:AAI0003
Stigmella luteella|RMNH.INS.24030|Italy.Sicily|BOLD:AAI0003
Stigmella luteella|MM18053|Finland|BOLD:AAI0003
Stigmella luteella|RMNH.INS.23689|France.Corsica|BOLD:AAI0003
Stigmella luteella|RMNH.INS.12986|Italy.Piedmont|BOLD:AAI0003
Stigmella luteella|RMNH.INS.29959|Norway.Nordland|BOLD:AAI0003
Stigmella luteella|RMNH.INS.23701|Switzerland.Valais|BOLD:AAI0003
Stigmella luteella|KLM Lep 00297|Austria.Carinthia|BOLD:AAI0003
Stigmella luteella|RMNH.INS.29968|Russia.Kareliya Republic|BOLD:AAI0003
Stigmella luteella|MM00928|Finland.Northern Ostrobothnia|BOLD:AAI0003
Stigmella luteella|TLMF Lep 18082|Austria.Tirol|BOLD:AAI0003
Stigmella luteella|RMNH.INS.29970|Russia.Vologodskaya Oblast|BOLD:AAI0003
Stigmella luteella|RMNH.INS.29971|Russia.Kareliya Republic|BOLD:AAI0003
Stigmella luteella|RMNH.INS.29973|Russia.Kareliya Republic|BOLD:AAI0003
Stigmella luteella|RMNH.INS.29972|Russia.Kareliya Republic|BOLD:AAI0003
Stigmella attenuata|RMNH.INS.29210|South Korea|BOLD:ACG8702
Stigmella attenuata|RMNH.INS.29982|Japan.Hokkaido|BOLD:ACU6114
Stigmella attenuata|RMNH.INS.29763|Japan.Hokkaido|BOLD:ACU6114
Stigmella betulicola|RMNH.INS.18678|United States.New York|BOLD:ACB7893
Stigmella betulicola|RMNH.INS.24418|United States.New York|BOLD:ACB7893
Stigmella betulicola|RMNH.INS.18540|United States.New York|BOLD:ACB7893
Stigmella betulicola|RMNH.INS.24409|United States.New York|BOLD:ACB7893
Stigmella betulicola|RMNH.INS.29756|Japan.Hokkaido|BOLD:AAP7527
Stigmella betulicola|RMNH.INS.12782|Sweden|BOLD:AAP7527
Stigmella betulicola|MM15178|Finland|BOLD:AAP7527
Stigmella betulicola|MM15176|Finland|BOLD:AAP7527
Stigmella betulicola|MM15177|Finland|BOLD:AAP7527
Stigmella betulicola|RMNH.INS.11438|Netherlands.Utrecht|BOLD:AAP7527
Stigmella betulicola|RMNH.INS.11439|Netherlands.Utrecht|BOLD:AAP7527
Stigmella betulicola|RMNH.INS.11440|Netherlands.Utrecht|BOLD:AAP7527
Stigmella corylifoliella|RMNH.INS.18653|United States.Massachusetts|BOLD:ACB8338
Stigmella corylifoliella|RMNH.INS.18318|United States.North Carolina|BOLD:ACB8338
Stigmella corylifoliella|RMNH.INS.30816|Canada.Ontario|BOLD:ACB8338
Stigmella corylifoliella|RMNH.INS.29885|Canada.Saskatchewan|
Stigmella corylifoliella|RMNH.INS.18227|United States.North Carolina|BOLD:ACB8338
Stigmella corylifoliella|RMNH.INS.18305|United States.Tennessee|BOLD:ACB8338
Stigmella corylifoliella|RMNH.INS.30795|United States.Vermont|BOLD:ACB8338
Stigmella corylifoliella|RMNH.INS.18386|United States.Mississippi|BOLD:ACB8338
Stigmella corylifoliella|RMNH.INS.30539|United States.Massachusetts|BOLD:ACB8338
Stigmella corylifoliella|RMNH.INS.18306|United States.Tennessee|BOLD:ACB8338
Stigmella corylifoliella|RMNH.INS.18552|United States.New York|BOLD:ACB8338
Stigmella corylifoliella|RMNH.INS.18639|United States.New York|BOLD:ACB8338
Stigmella corylifoliella|RMNH.INS.24342|United States.Georgia|BOLD:ACB8338
Stigmella corylifoliella|RMNH.INS.18197|United States.North Carolina|BOLD:ACB8338
Stigmella nr. corylifoliella|RMNH.INS.30536|United States.Massachusetts|BOLD:ABX9484
Stigmella nr. corylifoliella|RMNH.INS.18547|United States.New York|BOLD:ABX9484
Stigmella microtheriella|RMNH.INS.30116|South Korea|BOLD:ACU7466
Stigmella CarpinusAzerbaijan|RMNH.INS.29905|Azerbaijan|
Stigmella microtheriella|BC ZSM Lep 50948|Germany.Bavaria|
Stigmella microtheriella|RMNH.INS.29908|Azerbaijan|BOLD:ACU6796
Stigmella microtheriella|RMNH.INS.29983|Japan.Hokkaido|BOLD:ACU7085
Stigmella microtheriella|RMNH.INS.30377|Japan.Honshu|BOLD:ACU7085
Stigmella microtheriella|RMNH.INS.29748|Japan.Hokkaido|BOLD:ACU7085
Stigmella microtheriella|RMNH.INS.30184|South Korea|BOLD:ACU7085
Stigmella microtheriella|RMNH.INS.24629|South Korea|BOLD:ACU7085
Stigmella microtheriella|RMNH.INS.30156|South Korea|BOLD:ACU7085
Stigmella microtheriella|RMNH.INS.24430|Greece|BOLD:AAI0007
Stigmella microtheriella|RMNH.INS.18813|Greece|BOLD:AAI0007
Stigmella microtheriella|RMNH.INS.11265|Belgium.Luxembourg|BOLD:AAI0007
Stigmella microtheriella|RMNH.INS.11264|Belgium.Luxembourg|BOLD:AAI0007
Stigmella microtheriella|KLM Lep 02922|Austria|BOLD:AAI0007
Stigmella microtheriella|RMNH.INS.18172|United States.Washington|BOLD:AAI0007
Stigmella microtheriella|RMNH.INS.30721|Canada.Ontario|BOLD:AAI0007
Stigmella microtheriella|RMNH.INS.18605|United States.Vermont|BOLD:AAI0007
Stigmella microtheriella|RMNH.INS.18519|United States.Connecticut|BOLD:AAI0007
Stigmella microtheriella|TLMF Lep 16583|Italy.South Tyrol|BOLD:AAI0007
Stigmella microtheriella|RMNH.INS.29918|Greece.Central Macedonia|BOLD:AAI0007
Stigmella microtheriella|RMNH.INS.23608|Italy.Veneto|BOLD:AAI0007
Stigmella microtheriella|MM09608|Finland.Aland Islands|BOLD:AAI0007
Stigmella microtheriella|RMNH.INS.30043|Netherlands.South Holland|BOLD:AAI0007
Stigmella microtheriella|MM07008|Finland|BOLD:AAI0007
Stigmella microtheriella|RMNH.INS.12575|France.Provence-Alpes-Cote d'Azur|BOLD:AAI0007
Stigmella microtheriella|RMNH.INS.24111|Germany.North Rhine-Westphalia|BOLD:AAI0007
Stigmella microtheriella|KLM Lep 01947|Austria|BOLD:AAI0007
Stigmella microtheriella|TLMF Lep 11544|Italy.South Tyrol|BOLD:AAI0007
Stigmella microtheriella|RMNH.INS.23824|Italy.Piedmont|BOLD:AAI0007
Stigmella microtheriella|KLM Lep 02906|Austria|BOLD:AAI0007
Stigmella microtheriella|BC ZSM Lep 89417|Germany.Bavaria|BOLD:AAI0007
Stigmella microtheriella|MM07009|Finland|BOLD:AAI0007
Stigmella microtheriella|KLM Lep 00922|Austria.Carinthia|BOLD:AAI0007
Stigmella microtheriella|KLM Lep 01965|Slovenia|BOLD:AAI0007
Stigmella microtheriella|KLM Lep 01966|Slovenia|BOLD:AAI0007
Stigmella microtheriella|KLM Lep 01954|Austria|BOLD:AAI0007
Stigmella microtheriella|KLM Lep 00286|Austria.Carinthia|BOLD:AAI0007
Stigmella microtheriella|KLM Lep 02933|Austria|
Stigmella microtheriella|KLM Lep 02909|Austria|BOLD:AAI0007
Stigmella CaryaUSA|RMNH.INS.24346|United States.Mississippi|BOLD:ABX4303
Stigmella CaryaUSA|RMNH.INS.18567|United States.Connecticut|BOLD:ABX4303
Stigmella CaryaUSA|RMNH.INS.24415|United States.Connecticut|BOLD:ABX4303
Stigmella caryaefoliella|CNCLEP00042454|United States.Maryland|BOLD:AAI6171
Stigmella caryaefoliella|RMNH.INS.18280|United States.Tennessee|BOLD:AAI6171
Stigmella caryaefoliella|RMNH.INS.24414|United States.Connecticut|BOLD:AAI6171
Stigmella caryaefoliella|RMNH.INS.18586|United States.Vermont|BOLD:AAI6171
Stigmella caryaefoliella|RMNH.INS.18339|United States.Mississippi|BOLD:AAI6171
Stigmella caryaefoliella|RMNH.INS.18368|United States.Alabama|BOLD:AAI6171

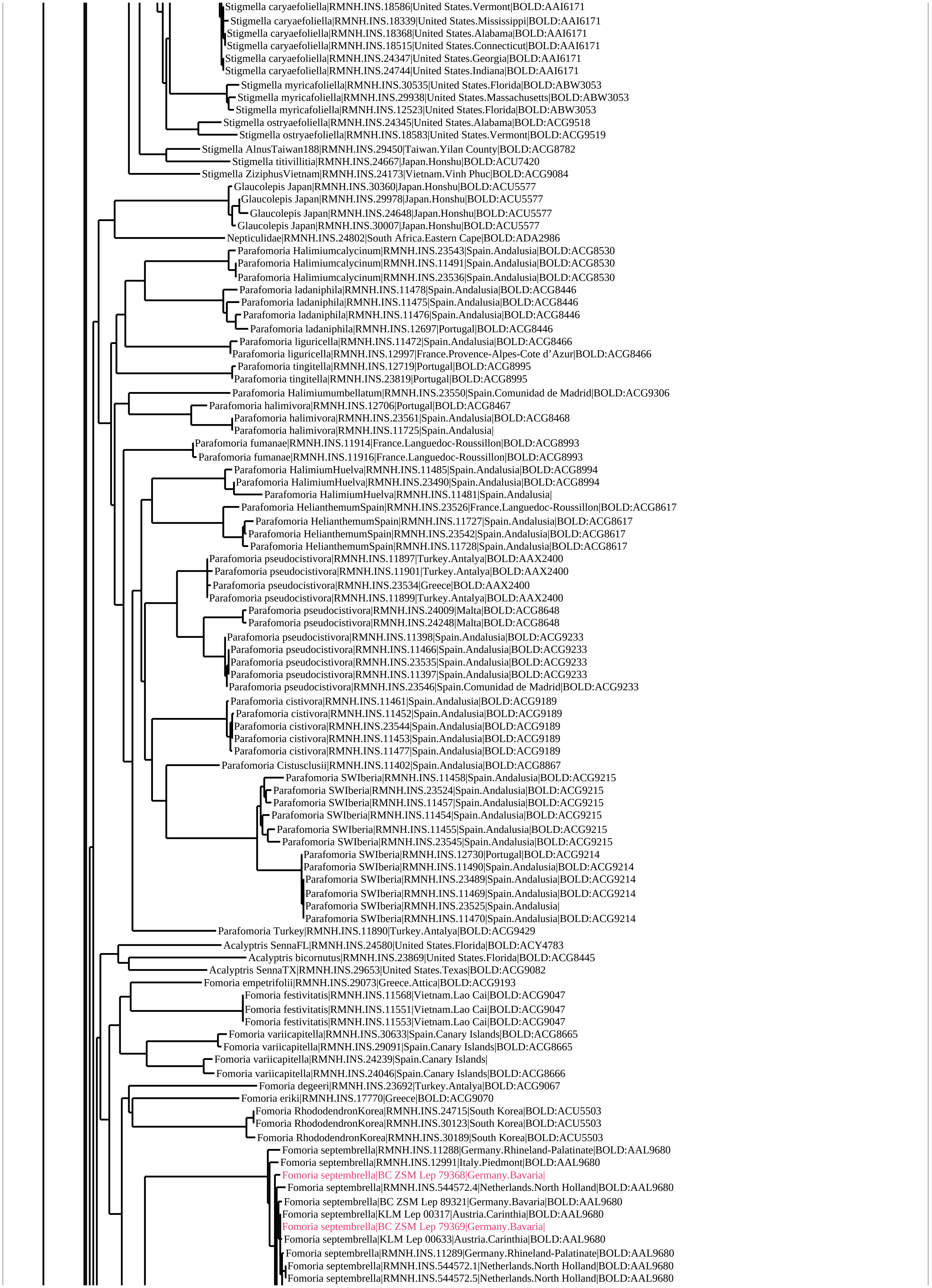
Stigmella caryaefoliella|RMNH.INS.18586|United States.Vermont|BOLD:AAI6171
Stigmella caryaefoliella|RMNH.INS.18339|United States.Mississippi|BOLD:AAI6171
Stigmella caryaefoliella|RMNH.INS.18368|United States.Alabama|BOLD:AAI6171
Stigmella caryaefoliella|RMNH.INS.18515|United States.Connecticut|BOLD:AAI6171
Stigmella caryaefoliella|RMNH.INS.24347|United States.Georgia|BOLD:AAI6171
Stigmella caryaefoliella|RMNH.INS.24744|United States.Indiana|BOLD:AAI6171
Stigmella myricafoliella|RMNH.INS.30535|United States.Florida|BOLD:ABW3053
Stigmella myricafoliella|RMNH.INS.29938|United States.Massachusetts|BOLD:ABW3053
Stigmella myricafoliella|RMNH.INS.12523|United States.Florida|BOLD:ABW3053
Stigmella ostryaefoliella|RMNH.INS.24345|United States.Alabama|BOLD:ACG9518
Stigmella ostryaefoliella|RMNH.INS.18583|United States.Vermont|BOLD:ACG9519
Stigmella AlnusTaiwan188|RMNH.INS.29450|Taiwan.Yilan County|BOLD:ACG8782
Stigmella titivillitia|RMNH.INS.24667|Japan.Honshu|BOLD:ACU7420
Stigmella ZiziphusVietnam|RMNH.INS.24173|Vietnam.Vinh Phuc|BOLD:ACG9084
Glaucolepis Japan|RMNH.INS.30360|Japan.Honshu|BOLD:ACU5577
Glaucolepis Japan|RMNH.INS.29978|Japan.Honshu|BOLD:ACU5577
Glaucolepis Japan|RMNH.INS.24648|Japan.Honshu|BOLD:ACU5577
Glaucolepis Japan|RMNH.INS.30007|Japan.Honshu|BOLD:ACU5577
Nepticulidae|RMNH.INS.24802|South Africa.Eastern Cape|BOLD:ADA2986
Parafomoria Halimiumcalycinum|RMNH.INS.23543|Spain.Andalusia|BOLD:ACG8530
Parafomoria Halimiumcalycinum|RMNH.INS.11491|Spain.Andalusia|BOLD:ACG8530
Parafomoria Halimiumcalycinum|RMNH.INS.23536|Spain.Andalusia|BOLD:ACG8530
Parafomoria ladaniphila|RMNH.INS.11478|Spain.Andalusia|BOLD:ACG8446
Parafomoria ladaniphila|RMNH.INS.11475|Spain.Andalusia|BOLD:ACG8446
Parafomoria ladaniphila|RMNH.INS.11476|Spain.Andalusia|BOLD:ACG8446
Parafomoria ladaniphila|RMNH.INS.12697|Portugal|BOLD:ACG8446
Parafomoria liguricella|RMNH.INS.11472|Spain.Andalusia|BOLD:ACG8466
Parafomoria liguricella|RMNH.INS.12997|France.Provence-Alpes-Cote d'Azur|BOLD:ACG8466
Parafomoria tingitella|RMNH.INS.12719|Portugal|BOLD:ACG8995
Parafomoria tingitella|RMNH.INS.23819|Portugal|BOLD:ACG8995
Parafomoria Halimiumumbellatum|RMNH.INS.23550|Spain.Comunidad de Madrid|BOLD:ACG9306
Parafomoria halimivora|RMNH.INS.12706|Portugal|BOLD:ACG8467
Parafomoria halimivora|RMNH.INS.23561|Spain.Andalusia|BOLD:ACG8468
Parafomoria halimivora|RMNH.INS.11725|Spain.Andalusia|
Parafomoria fumanae|RMNH.INS.11914|France.Languedoc-Roussillon|BOLD:ACG8993
Parafomoria fumanae|RMNH.INS.11916|France.Languedoc-Roussillon|BOLD:ACG8993
Parafomoria HalimiumHuelva|RMNH.INS.11485|Spain.Andalusia|BOLD:ACG8994
Parafomoria HalimiumHuelva|RMNH.INS.23490|Spain.Andalusia|BOLD:ACG8994
Parafomoria HalimiumHuelva|RMNH.INS.11481|Spain.Andalusia|
Parafomoria HelianthemumSpain|RMNH.INS.23526|France.Languedoc-Roussillon|BOLD:ACG8617
Parafomoria HelianthemumSpain|RMNH.INS.11727|Spain.Andalusia|BOLD:ACG8617
Parafomoria HelianthemumSpain|RMNH.INS.23542|Spain.Andalusia|BOLD:ACG8617
Parafomoria HelianthemumSpain|RMNH.INS.11728|Spain.Andalusia|BOLD:ACG8617
Parafomoria pseudocistivora|RMNH.INS.11897|Turkey.Antalya|BOLD:AAX2400
Parafomoria pseudocistivora|RMNH.INS.11901|Turkey.Antalya|BOLD:AAX2400
Parafomoria pseudocistivora|RMNH.INS.23534|Greece|BOLD:AAX2400
Parafomoria pseudocistivora|RMNH.INS.11899|Turkey.Antalya|BOLD:AAX2400
Parafomoria pseudocistivora|RMNH.INS.24009|Malta|BOLD:ACG8648
Parafomoria pseudocistivora|RMNH.INS.24248|Malta|BOLD:ACG8648
Parafomoria pseudocistivora|RMNH.INS.11398|Spain.Andalusia|BOLD:ACG9233
Parafomoria pseudocistivora|RMNH.INS.11466|Spain.Andalusia|BOLD:ACG9233
Parafomoria pseudocistivora|RMNH.INS.23535|Spain.Andalusia|BOLD:ACG9233
Parafomoria pseudocistivora|RMNH.INS.11397|Spain.Andalusia|BOLD:ACG9233
Parafomoria pseudocistivora|RMNH.INS.23546|Spain.Comunidad de Madrid|BOLD:ACG9233
Parafomoria cistivora|RMNH.INS.11461|Spain.Andalusia|BOLD:ACG9189
Parafomoria cistivora|RMNH.INS.11452|Spain.Andalusia|BOLD:ACG9189
Parafomoria cistivora|RMNH.INS.23544|Spain.Andalusia|BOLD:ACG9189
Parafomoria cistivora|RMNH.INS.11453|Spain.Andalusia|BOLD:ACG9189
Parafomoria cistivora|RMNH.INS.11477|Spain.Andalusia|BOLD:ACG9189
Parafomoria Cistusclusii|RMNH.INS.11402|Spain.Andalusia|BOLD:ACG8867
Parafomoria SWIberia|RMNH.INS.11458|Spain.Andalusia|BOLD:ACG9215
Parafomoria SWIberia|RMNH.INS.23524|Spain.Andalusia|BOLD:ACG9215
Parafomoria SWIberia|RMNH.INS.11457|Spain.Andalusia|BOLD:ACG9215
Parafomoria SWIberia|RMNH.INS.11454|Spain.Andalusia|BOLD:ACG9215
Parafomoria SWIberia|RMNH.INS.11455|Spain.Andalusia|BOLD:ACG9215
Parafomoria SWIberia|RMNH.INS.23545|Spain.Andalusia|BOLD:ACG9215
Parafomoria SWIberia|RMNH.INS.12730|Portugal|BOLD:ACG9214
Parafomoria SWIberia|RMNH.INS.11490|Spain.Andalusia|BOLD:ACG9214
Parafomoria SWIberia|RMNH.INS.23489|Spain.Andalusia|BOLD:ACG9214
Parafomoria SWIberia|RMNH.INS.11469|Spain.Andalusia|BOLD:ACG9214
Parafomoria SWIberia|RMNH.INS.23525|Spain.Andalusia|
Parafomoria SWIberia|RMNH.INS.11470|Spain.Andalusia|BOLD:ACG9214
Parafomoria Turkey|RMNH.INS.11890|Turkey.Antalya|BOLD:ACG9429
Acalyptris SennaFL|RMNH.INS.24580|United States.Florida|BOLD:ACY4783
Acalyptris bicornutus|RMNH.INS.23869|United States.Florida|BOLD:ACG8445
Acalyptris SennaTX|RMNH.INS.29653|United States.Texas|BOLD:ACG9082
Fomoria empetrifolii|RMNH.INS.29073|Greece.Attica|BOLD:ACG9193
Fomoria festivitatis|RMNH.INS.11568|Vietnam.Lao Cai|BOLD:ACG9047
Fomoria festivitatis|RMNH.INS.11551|Vietnam.Lao Cai|BOLD:ACG9047
Fomoria festivitatis|RMNH.INS.11553|Vietnam.Lao Cai|BOLD:ACG9047
Fomoria variicapitella|RMNH.INS.30633|Spain.Canary Islands|BOLD:ACG8665
Fomoria variicapitella|RMNH.INS.29091|Spain.Canary Islands|BOLD:ACG8665
Fomoria variicapitella|RMNH.INS.24239|Spain.Canary Islands|
Fomoria variicapitella|RMNH.INS.24046|Spain.Canary Islands|BOLD:ACG8666
Fomoria degeeri|RMNH.INS.23692|Turkey.Antalya|BOLD:ACG9067
Fomoria eriki|RMNH.INS.17770|Greece|BOLD:ACG9070
Fomoria RhododendronKorea|RMNH.INS.24715|South Korea|BOLD:ACU5503
Fomoria RhododendronKorea|RMNH.INS.30123|South Korea|BOLD:ACU5503
Fomoria RhododendronKorea|RMNH.INS.30189|South Korea|BOLD:ACU5503
Fomoria septembrella|RMNH.INS.11288|Germany.Rhineland-Palatinate|BOLD:AAL9680
Fomoria septembrella|RMNH.INS.12991|Italy.Piedmont|BOLD:AAL9680
Fomoria septembrella|BC ZSM Lep 79368|Germany.Bavaria|
Fomoria septembrella|RMNH.INS.544572.4|Netherlands.North Holland|BOLD:AAL9680
Fomoria septembrella|BC ZSM Lep 89321|Germany.Bavaria|BOLD:AAL9680
Fomoria septembrella|KLM Lep 00317|Austria.Carinthia|BOLD:AAL9680
Fomoria septembrella|BC ZSM Lep 79369|Germany.Bavaria|
Fomoria septembrella|KLM Lep 00633|Austria.Carinthia|BOLD:AAL9680
Fomoria septembrella|RMNH.INS.11289|Germany.Rhineland-Palatinate|BOLD:AAL9680
Fomoria septembrella|RMNH.INS.544572.1|Netherlands.North Holland|BOLD:AAL9680
Fomoria septembrella|RMNH.INS.544572.5|Netherlands.North Holland|BOLD:AAL9680

Fomoria septembrella|RMNH.INS.11289|Germany.Rhineland-Palatinate|BOLD:AAL9680
Fomoria septembrella|RMNH.INS.544572.1|Netherlands.North Holland|BOLD:AAL9680
Fomoria septembrella|RMNH.INS.544572.5|Netherlands.North Holland|BOLD:AAL9680
Fomoria septembrella|KLM Lep 02884|Slovenia|BOLD:AAL9680
Fomoria septembrella|KLM Lep 01250|Austria.Carinthia|BOLD:AAL9680
Fomoria septembrella|KLM Lep 01265|Austria.Carinthia|BOLD:AAL9680
Fomoria septembrella|BC ZSM Lep 89438|Germany.Bavaria|BOLD:AAL9680
Fomoria septembrella|BC ZSM Lep 89433|Germany.Bavaria|
Fomoria septembrella|BC ZSM Lep 89132|Germany.Bavaria|BOLD:AAL9680
Fomoria septembrella|BC ZSM Lep 89416|Germany.Bavaria|
Fomoria septembrella|CNCLEP00077690|Canada.British Columbia|BOLD:AAL9680
Fomoria septembrella|RMNH.INS.23694|United States.Washington|BOLD:AAL9680
Fomoria septembrella|MM14836|Finland.Aland Islands|BOLD:AAL9680
Fomoria septembrella|MM14835|Finland.Aland Islands|BOLD:AAL9680
Fomoria septembrella|RMNH.INS.544572.3|Netherlands.North Holland|BOLD:AAL9680
Fomoria septembrella|RMNH.INS.544572.2|Netherlands.North Holland|BOLD:AAL9680
Fomoria septembrella|RMNH.INS.23609|Italy.Piedmont|BOLD:AAL9680
Fomoria weaveri|RMNH.INS.23722|Canada.Alberta|BOLD:AAF6478
Fomoria weaveri|BC ZSM Lep 89360|Germany.Bavaria|BOLD:AAF6478
Fomoria weaveri|MM08062|Finland|BOLD:AAF6478
Fomoria weaveri|MM03165|Finland.Northern Ostrobothnia|BOLD:AAF6478
Fomoria weaveri|MM02640|Finland.South Karelia|BOLD:AAF6478
Bohemannia pulverosella|RMNH.INS.11859|Poland|BOLD:AAI8790
Bohemannia pulverosella|RMNH.INS.11822|Netherlands.South Holland|BOLD:AAI8790
Bohemannia pulverosella|RMNH.INS.11594|Netherlands.South Holland|BOLD:AAI8790
Bohemannia pulverosella|RMNH.INS.11821|Netherlands.South Holland|BOLD:AAI8790
Bohemannia pulverosella|RMNH.INS.30715|Canada.Ontario|BOLD:AAI8790
Bohemannia pulverosella|RMNH.INS.30217|Russia.Krasnoyarskiy Kray|BOLD:AAI8790
Bohemannia pulverosella|MM06852|Finland.Aland Islands|BOLD:AAI8790
Bohemannia pulverosella|MM08667|Finland.Aland Islands|BOLD:AAI8790
Bohemannia pulverosella|MM08668|Finland.Aland Islands|BOLD:AAI8790
Ectoedemia AlnusJapan|RMNH.INS.29854|Japan.Honshu|BOLD:ACU7224
Muhabbetana euphorbiella|RMNH.INS.23559|Greece|BOLD:ACY4518
Muhabbetana euphorbiella|RMNH.INS.24011|Malta|BOLD:ACG8513
Muhabbetana euphorbiella|RMNH.INS.17948|Malta|BOLD:ACG8513
Muhabbetana jubae|RMNH.INS.11610|Spain.Canary Islands|BOLD:ACG8599
Muhabbetana jubae|RMNH.INS.11611|Spain.Canary Islands|BOLD:ACG8599
Muhabbetana jubae|RMNH.INS.24048|Spain.Canary Islands|BOLD:ACG8599
Muhabbetana jubae|RMNH.INS.24596|Spain.Canary Islands|BOLD:ACY4517
Muhabbetana jubae|RMNH.INS.30627|Spain.Canary Islands|BOLD:ADA2165
Muhabbetana jubae|RMNH.INS.29079|Spain.Canary Islands|BOLD:ACG8502
Muhabbetana jubae|RMNH.INS.29559|Spain.Canary Islands|BOLD:ACG8502
Trifurcula barbertonensis|RMNH.INS.24803|South Africa.Eastern Cape|BOLD:ADA2769
Enteucha gilvafascia|RMNH.INS.12572|United States.Florida|BOLD:ABX3463
Menurella xenadelpha|RMNH.INS.11968|Indonesia.Kalimantan Timur|BOLD:ACB2040
Menurella xenadelpha|RMNH.INS.23738|Indonesia.Kalimantan Timur|BOLD:ACB2040
Acalyptris lotella|USNMENT00656961|United States.California|
Acalyptris lotella|USNMENT00656963|United States.California|
Neotrifurcula EvN4504|RMNH.INS.24504|Chile.Valparaiso|BOLD:ACU6693
Neotrifurcula gielisorum|RMNH.INS.23527|Chile|BOLD:ACG8607
Neotrifurcula gielisorum|RMNH.INS.24503|Chile|BOLD:ACG8607
Enteucha acetosae|RMNH.INS.11272|Germany.North Rhine-Westphalia|BOLD:AAV9437
Enteucha acetosae|RMNH.INS.11273|Germany.North Rhine-Westphalia|BOLD:AAV9437
Enteucha PersicariaJapan|RMNH.INS.24646|Japan.Honshu|BOLD:ACU6225
Enteucha PersicariaJapan|RMNH.INS.24631|Japan.Honshu|BOLD:ACU6225
Enteucha PersicariaJapan|RMNH.INS.30014|Japan.Honshu|BOLD:ACU6225
Enteucha PersicariaJapan|RMNH.INS.30013|Japan.Honshu|BOLD:ACU6225
Enteucha PersicariaJapan|RMNH.INS.30381|Japan.Honshu|BOLD:ACU6225
Enteucha BistortaJapan|RMNH.INS.30008|Japan.Honshu|BOLD:ACU6117
Enteucha BistortaJapan|RMNH.INS.24721|Japan.Honshu|BOLD:ACU6117
Enteucha Persicariacapitata|RMNH.INS.24183|Vietnam.Lao Cai|BOLD:ACG8571
Enteucha Persicariacapitata|RMNH.INS.24182|Vietnam.Lao Cai|BOLD:ACG8571
Enteucha Persicariacapitata|RMNH.INS.11543|Vietnam.Lao Cai|BOLD:ACG8571
Enteucha Persicariacapitata|RMNH.INS.11545|Vietnam.Lao Cai|BOLD:ACG8571
Enteucha PersicariachinensisVietnam|RMNH.INS.24185|Vietnam.Lao Cai|BOLD:ACG8832
Enteucha PersicariachinensisVietnam|RMNH.INS.11560|Vietnam.Vinh Phuc|BOLD:ACG8832
Enteucha PersicariachinensisTw|RMNH.INS.24327|Taiwan|BOLD:ACG9184
Enteucha PersicariachinensisTw|RMNH.INS.29270|Taiwan|BOLD:ACG9184
Enteucha PersicariaposumbuTw|RMNH.INS.29422|Taiwan.Nantou County|BOLD:ACG8444
Enteucha PersicariaposumbuVietnam|RMNH.INS.24184|Vietnam.Vinh Phuc|BOLD:ACG8443
Enteucha basidactyla|RMNH.INS.23627|Belize.Cayo|BOLD:ADA7417
Enteucha basidactyla|RMNH.INS.12525|United States.Florida|BOLD:ADA7417
Enteucha basidactyla|RMNH.INS.12524|United States.Florida|BOLD:ADA7417
Enteucha basidactyla|RMNH.INS.12526|United States.Florida|BOLD:ADA7417
Fomoria vannifera|RMNH.INS.24208|Tanzania.Morogoro|BOLD:ACY3786
Fomoria vannifera|RMNH.INS.23696|Tanzania.Morogoro|
Fomoria vannifera|RMNH.INS.24209|Yemen|BOLD:ACW8276
Pseudopostega KenyaRukinga1|RMNH.INS.24578|Kenya.Coast|BOLD:ACU7142
Opostegoides AUSVictoriaEvN4325|RMNH.INS.24325|Australia.Victoria|BOLD:AAW0582
Opostegoides BorneoEvN4564|RMNH.INS.24564|Indonesia.Kalimantan Timur|BOLD:AAL9205
Opostegoides menthinella|RMNH.INS.11639|Spain.Andalusia|
Neopostega falcata|DDAV-D155|Costa Rica.Heredia|BOLD:AAX6553
Neopostega petila|DDAV-D158|Costa Rica.Heredia|
Neopostega distola|DDAV-D156|Brazil.Mato Grosso|BOLD:AAX6557
Opostega spatulella|RMNH.INS.24019|Italy.Piedmont|BOLD:ACG8635
Opostega salaciella|TLMF Lep 16543|Italy.South Tyrol|BOLD:AAE4956
Opostega salaciella|TLMF Lep 10156|Italy.South Tyrol|BOLD:AAE4956
Opostega salaciella|TLMF Lep 17980|Italy.South Tyrol|BOLD:AAE4956
Opostega salaciella|TLMF Lep 11116|Austria.Tirol|BOLD:AAE4956
Opostega salaciella|MM16082|Finland.Uusimaa|BOLD:AAE4956
Opostega salaciella|MM09850|Finland|BOLD:AAE4956
Opostega salaciella|RMNH.INS.11623|Netherlands.North Holland|BOLD:AAE4956
Opostega salaciella|MM03987|Finland.Uusimaa|BOLD:AAE4956
Opostega salaciella|RMNH.INS.11907|Netherlands.North Holland|BOLD:AAE4956
Opostega salaciella|MM13732|Finland|BOLD:AAE4956
Pseudopostega fungina|RMNH.INS.24508|Vietnam.Bac Kan|BOLD:ACU6404
Neopostega asymmetra|DDAV-D154|Brazil.Rio de Janeiro|
Pseudopostega AustraliaNSW|RMNH.INS.24324|Australia.New South Wales|BOLD:ACG9135
Pseudopostega serrata|DDAV-D206|Costa Rica.Heredia|BOLD:AAX6730
Pseudopostega KenyaRukinga2|RMNH.INS.24579|Kenya.Coast|BOLD:ACU5925
[illegible]

Pseudopostega AustraliaNSW|RMNH.INS.24324|Australia.New South Wales|BOLD:ACG9135
Pseudopostega serrata|DDAV-D206|Costa Rica.Heredia|BOLD:AAX6730
Pseudopostega KenyaRukinga2|RMNH.INS.24579|Kenya.Coast|BOLD:ACU5925
Pseudopostega KenyaTheArk|RMNH.INS.24575|Kenya.Central|BOLD:ABW6476
Pseudopostega indonesica|RMNH.INS.24510|Vietnam.Bac Kan|BOLD:ACU7128
Pseudopostega KenyaTuri|RMNH.INS.24577|Kenya.Rift Valley|BOLD:ACU7215
Pseudopostega Turkey|RMNH.INS.24244|Turkey.Denizli|
Pseudopostega albogaleriella|DDAV-D164|United States.California|BOLD:AAF7026
Pseudopostega albogaleriella|DDAV-D163|United States.Louisiana|
Pseudopostega quadristrigella|DDAV-D202|United States.Texas|BOLD:ABY9839
Pseudopostega auritella|RMNH.INS.24563|South Korea|BOLD:ACU7302
Pseudopostega auritella|MM18895|Finland|
Pseudopostega auritella|RMNH.INS.11622|Netherlands.North Holland|BOLD:ABV8371
Pseudopostega auritella|RMNH.INS.11908|Netherlands.North Holland|BOLD:ABV8371
Pseudopostega auritella|MM21143|Finland|BOLD:ABV8371
Pseudopostega chalcopepla|RMNH.INS.24603|Cyprus|BOLD:ACU5903
Pseudopostega chalcopepla|RMNH.INS.550071|France.Corsica|BOLD:ACU8218
Pseudopostega chalcopepla|TLMF Lep 03903|Spain.Comunidad Valenciana|BOLD:AAV2271
Pseudopostega chalcopepla|TLMF Lep 03904|Spain.Comunidad Valenciana|BOLD:AAV2271
Pseudopostega chalcopepla|RMNH.INS.24278|Spain.Aragon|BOLD:AAV2271
Pseudopostega chalcopepla|RMNH.INS.23754|France.Provence-Alpes-Cote d'Azur|BOLD:AAV2271
Pseudopostega chalcopepla|RMNH.INS.24280|France.Languedoc-Roussillon|BOLD:AAV2271
Pseudopostega chalcopepla|RMNH.INS.24279|France.Provence-Alpes-Cote d'Azur|BOLD:AAV2271
Pseudopostega crepusculella|BC ZSM Lep 29136|Germany.Bavaria|BOLD:ABZ6679
Pseudopostega crepusculella|TLMF Lep 17942|Italy.South Tyrol|BOLD:AAE8087
Pseudopostega crepusculella|TLMF Lep 17945|Italy.South Tyrol|BOLD:AAE8087
Pseudopostega crepusculella|TLMF Lep 09199|Austria.Vorarlberg|BOLD:AAE8087
Pseudopostega crepusculella|TLMF Lep 10102|Austria.Tirol|BOLD:AAE8087
Pseudopostega crepusculella|BC ZSM Lep 26946|Germany.Bavaria|BOLD:AAE8087
Pseudopostega crepusculella|MM12486|Finland|BOLD:AAE8088
Pseudopostega crepusculella|MM12485|Finland|BOLD:AAE8088
Pseudopostega crepusculella|MM12490|Finland|BOLD:AAE8088
Pseudopostega crepusculella|TLMF Lep 10251|Austria.Tirol|BOLD:AAE8088
Pseudopostega beckeri|DDAV-D168|Brazil|
Pseudopostega protomochla|DDAV-D201|Argentina.Jujuy|BOLD:AAX6743
Pseudopostega venticola|DDAV-D215|Brazil.Mato Grosso|BOLD:AAX6732
Pseudopostega KenyaWatamu|RMNH.INS.24576|Kenya.Coast|BOLD:ACU7216
Pseudopostega apoclina|DDAV-D195|Costa Rica.Heredia|
Pseudopostega dorsalis|DDAV-D181|Costa Rica.San Jose|
Pseudopostega duplicata|DDAV-D184|Costa Rica.Heredia|BOLD:AAZ2520
Pseudopostega duplicata|DDAV-D185|British Virgin Islands.Tortola|BOLD:AAH8235
Pseudopostega latifurcata|DDAV-D192|British Virgin Islands.Tortola|BOLD:AAZ2964
Pseudopostega lobata|DDAV-D198|Costa Rica.Heredia|BOLD:AAD3851
Pseudopostega longipedicella|DDAV-D199|Costa Rica.Puntarenas|BOLD:AAD3851
Pseudopostega sublobata|DDAV-D208|Costa Rica.Heredia|BOLD:AAX6731
Pseudopostega brevifurcata|DDAV-D172|Costa Rica.Heredia|
Pseudopostega brevivalva|DDAV-D173|Costa Rica.Heredia|BOLD:AAX6755
Pseudopostega latisaccula|DDAV-D196|Dominica|
Pseudopostega attenuata|DDAV-D166|Costa Rica.Heredia|BOLD:AAX6754
Pseudopostega tenuifurcata|DDAV-D211|Costa Rica.Heredia|
Pseudopostega sectila|DDAV-D205|British Virgin Islands.Tortola|
Pseudopostega tucumanae|DDAV-D213|Argentina.Tucuman|
Pseudopostega abrupta|DDAV-D159|British Virgin Islands.Guana Island|
Pseudopostega saltatrix|DDAV-D204|Costa Rica.Heredia|BOLD:AAH8233
Pseudopostega VietnamEvN4507|RMNH.INS.24507|Vietnam.Bac Kan|BOLD:ACU7318
Pseudopostega VietnamEvN4509|RMNH.INS.24509|Vietnam.Bac Kan|BOLD:ACU7317
Ectoedemia CarpinuscordataJapan|RMNH.INS.29912|Japan.Hokkaido|BOLD:ACH1617
Ectoedemia CarpinuscordataJapan|RMNH.INS.29860|Japan.Honshu|BOLD:ACH1617
Ectoedemia CarpinuscordataJapan|RMNH.INS.30322|Japan.Honshu|BOLD:ACH1617
Ectoedemia CarpinusVietnam|RMNH.INS.23902|Vietnam.Lao Cai|BOLD:AAI6178
Ectoedemia CarpinusVietnam|RMNH.INS.11550|Vietnam.Lao Cai|BOLD:AAI6178
Ectoedemia CorylusJapan|RMNH.INS.30324|Japan.Honshu|BOLD:ACU6465
Ectoedemia CorylusJapan|RMNH.INS.29857|Japan.Honshu|BOLD:ACU6465
Ectoedemia quadrinotata|RMNH.INS.18557|United States.New York|BOLD:ACG9442
Ectoedemia quadrinotata|RMNH.INS.18228|United States.North Carolina|BOLD:AAI9358
Ectoedemia quadrinotata|RMNH.INS.18593|United States.Vermont|BOLD:AAI9358
Ectoedemia quadrinotata|RMNH.INS.18641|United States.Vermont|BOLD:AAI9358
Ectoedemia quadrinotata|RMNH.INS.29901|Canada.Ontario|BOLD:AAI9358
Ectoedemia quadrinotata|RMNH.INS.29902|Canada.Ontario|BOLD:AAI9358
Ectoedemia quadrinotata|RMNH.INS.23901|United States.Connecticut|BOLD:AAI9358
Ectoedemia quadrinotata|RMNH.INS.23900|United States.Connecticut|BOLD:AAI9358
Ectoedemia FagusJapan29826|RMNH.INS.29846|Japan.Honshu|BOLD:ACU6353
Ectoedemia FagusJapan29826|RMNH.INS.29826|Japan.Honshu|BOLD:ACU6353
Ectoedemia FagusJapan29847|RMNH.INS.29847|Japan.Honshu|BOLD:ACU7426
Ectoedemia FagusJapan30391|RMNH.INS.30391|Japan.Honshu|BOLD:ACU7320
Ectoedemia philipi|RMNH.INS.30372|Japan.Honshu|BOLD:ACU7171
Ectoedemia philipi|RMNH.INS.29875|Japan.Honshu|BOLD:ACU7171
Ectoedemia philipi|RMNH.INS.29820|Japan.Honshu|BOLD:ACU7171
Ectoedemia philipi|RMNH.INS.29863|Japan.Honshu|BOLD:ACU7171
Ectoedemia TaiwanEvN4065|RMNH.INS.24065|Taiwan.Taiwan Province|
Ectoedemia AnnamocaryaVietnam|RMNH.INS.17989|Vietnam.Ninh Binh|BOLD:AAV1077
Ectoedemia AnnamocaryaVietnam|RMNH.INS.24351|Vietnam.Ninh Binh|BOLD:AAV1077
Ectoedemia preisseckeri|RMNH.INS.24724|Japan.Honshu|BOLD:ACU7393
Ectoedemia preisseckeri|RMNH.INS.29718|Japan.Hokkaido|BOLD:ACU7393
Ectoedemia preisseckeri|RMNH.INS.23871|Czech Republic.Southern Moravia|BOLD:AAI9363
Ectoedemia preisseckeri|RMNH.INS.23870|Czech Republic|BOLD:AAI9363
Ectoedemia preisseckeri|RMNH.INS.24534|Greece|BOLD:AAI9363
Ectoedemia CarpinusJapan|RMNH.INS.30318|Japan.Honshu|BOLD:ACU6065
Ectoedemia CarpinusJapan|RMNH.INS.30321|Japan.Honshu|BOLD:ACU6065
Ectoedemia CarpinusTaiwan|RMNH.INS.24470|China.Shanxi|BOLD:ACG8824
Ectoedemia CarpinusTaiwan|RMNH.INS.29383|Taiwan|BOLD:ACG8824
Ectoedemia CarpinusTaiwan|RMNH.INS.29382|Taiwan|BOLD:ACG8824
Ectoedemia leucothorax|RMNH.INS.11637|Spain.Andalusia|BOLD:AAF6505
Ectoedemia leucothorax|RMNH.INS.23834|Spain.Andalusia|BOLD:AAF6505
Ectoedemia leucothorax|RMNH.INS.23879|Spain.Andalusia|BOLD:AAF6505
Ectoedemia haraldi|RMNH.INS.23874|Spain.Catalonia|BOLD:AAE8152
Ectoedemia haraldi|RMNH.INS.23662|France.Languedoc-Roussillon|BOLD:AAE8152
Ectoedemia haraldi|RMNH.INS.23664|France.Languedoc-Roussillon|BOLD:AAE8152
Ectoedemia haraldi|RMNH.INS.11896|Turkey.Antalya|BOLD:AAE8150
Ectoedemia haraldi|RMNH.INS.24195|Greece|BOLD:AAE8151
[illegible]

Ectoedemia haraldi|RMNH.INS.23664|France.Languedoc-Roussillon|BOLD:AAE8152
Ectoedemia haraldi|RMNH.INS.11896|Turkey.Antalya|BOLD:AAE8150
Ectoedemia haraldi|RMNH.INS.24195|Greece|BOLD:AAE8151
Ectoedemia haraldi|RMNH.INS.23424|Greece|BOLD:AAE8151
Ectoedemia haraldi|RMNH.INS.18765|Greece|BOLD:AAE8151
Ectoedemia haraldi|RMNH.INS.24391|Greece|BOLD:AAE8151
Ectoedemia pseudoilicis|RMNH.INS.23693|Turkey.Antalya|BOLD:AAF6510
Ectoedemia ilicis|RMNH.INS.24045|Morocco|BOLD:ACG9029
Ectoedemia ilicis|RMNH.INS.23667|France.Languedoc-Roussillon|BOLD:AAE8134
Ectoedemia ilicis|RMNH.INS.12714|Portugal.Faro|BOLD:AAE8134
Ectoedemia ilicis|RMNH.INS.12715|Portugal.Faro|BOLD:AAE8134
Ectoedemia ilicis|RMNH.INS.23841|Spain.Aragon|BOLD:AAE8134
Ectoedemia heringella|RMNH.INS.11865|United Kingdom.England|BOLD:AAD0479
Ectoedemia heringella|RMNH.INS.23594|Italy.Liguria|BOLD:AAD0479
Ectoedemia heringella|RMNH.INS.23616|Italy.Veneto|BOLD:AAD0479
Ectoedemia heringella|RMNH.INS.23615|Italy.Veneto|BOLD:AAD0479
Ectoedemia heringella|RMNH.INS.23512|United Kingdom.England|BOLD:AAD0479
Ectoedemia heringella|RMNH.INS.11877|United Kingdom.England|BOLD:AAD0479
Ectoedemia heringella|RMNH.INS.11872|United Kingdom.England|BOLD:AAD0479
Ectoedemia heringella|RMNH.INS.11864|United Kingdom.England|BOLD:AAD0479
Ectoedemia heringella|RMNH.INS.11873|United Kingdom.England|BOLD:AAD0479
Ectoedemia heringella|RMNH.INS.24610|Italy.Calabria|BOLD:AAD0479
Ectoedemia heringella|RMNH.INS.23674|Italy.Piedmont|BOLD:AAD0479
Ectoedemia heringella|RMNH.INS.11876|United Kingdom.England|BOLD:AAD0479
Ectoedemia pseudoilicis|RMNH.INS.23833|Greece.Peloponnese|BOLD:AAF6511
Ectoedemia pseudoilicis|RMNH.INS.23831|Greece.Peloponnese|BOLD:AAF6511
Ectoedemia CastaneaKorea|RMNH.INS.30204|South Korea|BOLD:ACU7428
Ectoedemia algeriensis|RMNH.INS.23842|France.Provence-Alpes-Cote d'Azur|BOLD:AAI9361
Ectoedemia algeriensis|RMNH.INS.23876|France.Languedoc-Roussillon|BOLD:AAI9361
Ectoedemia gilvipennella|RMNH.INS.23878|Czech Republic.Southern Moravia|BOLD:AAI9359
Ectoedemia gilvipennella|RMNH.INS.23840|Czech Republic.Southern Moravia|BOLD:AAI9359
Ectoedemia coscoja|RMNH.INS.23845|Spain.Andalusia|BOLD:AAX9882
Ectoedemia quinquella|RMNH.INS.17868|United Kingdom.England|BOLD:AAF6520
Ectoedemia quinquella|RMNH.INS.11347|Netherlands.Limburg|BOLD:AAF6520
Ectoedemia quinquella|RMNH.INS.17672|France.Provence-Alpes-Cote d'Azur|BOLD:AAF6520
Ectoedemia quinquella|RMNH.INS.30259|Portugal.Minho|BOLD:AAF6520
Ectoedemia quinquella|RMNH.INS.29014|Greece|BOLD:ACG9570
Ectoedemia QuercusvelutinaUSA|RMNH.INS.18254|United States.North Carolina|BOLD:ACG9328
Ectoedemia LithocarpusTaiwan|RMNH.INS.29547|Taiwan|BOLD:ACG9330
Ectoedemia cerris|RMNH.INS.17887|Slovakia|
Ectoedemia albifasciella|BC ZSM Lep 50970|Germany.Bavaria|
Ectoedemia albifasciella|BC ZSM Lep 50964|Germany.Bavaria|
Ectoedemia albifasciella|KLM Lep 02886|Slovenia|BOLD:AAB4064
Ectoedemia albifasciella|RMNH.INS.17845|Poland|BOLD:AAB4064
Ectoedemia albifasciella|KLM Lep 00294|Austria.Carinthia|BOLD:AAB4064
Ectoedemia albifasciella|KLM Lep 02892|Slovenia|BOLD:AAB4064
Ectoedemia albifasciella|TLMF Lep 12526|Austria.Vorarlberg|BOLD:AAB4064
Ectoedemia albifasciella|KLM Lep 02891|Slovenia|BOLD:AAB4064
Ectoedemia albifasciella|BC ZSM Lep 79379|Germany.Bavaria|BOLD:AAB4064
Ectoedemia albifasciella|MM09671|Finland.Aland Islands|BOLD:AAB4064
Ectoedemia albifasciella|RMNH.INS.18134|Bulgaria.Sofiya|BOLD:AAB4064
Ectoedemia albifasciella|RMNH.INS.12805|Netherlands.North Brabant|BOLD:AAB4064
Ectoedemia albifasciella|RMNH.INS.12837|Netherlands.Limburg|BOLD:AAB4064
Ectoedemia albifasciella|RMNH.INS.11425|Netherlands.Utrecht|BOLD:AAB4064
Ectoedemia albifasciella|MM00917|Finland.Aland Islands|BOLD:AAB4064
Ectoedemia albifasciella|BC ZSM Lep 79397|Germany.Bavaria|
Ectoedemia albifasciella|BC ZSM Lep 89402|Germany.Bavaria|BOLD:AAB4064
Ectoedemia albifasciella|KLM Lep 00288|Austria.Carinthia|BOLD:AAB4064
Ectoedemia albifasciella|KLM Lep 00313|Austria.Carinthia|BOLD:AAB4064
Ectoedemia albifasciella|KLM Lep 02901|Austria|BOLD:AAB4064
Ectoedemia albifasciella|TLMF Lep 15115|Austria.Tirol|BOLD:AAB4064
Ectoedemia albifasciella|TLMF Lep 16740|Austria.Tirol|BOLD:AAB4064
Ectoedemia albifasciella|TLMF Lep 15113|Austria.Tirol|BOLD:AAB4064
Ectoedemia albifasciella|RMNH.INS.12974|France.Provence-Alpes-Cote d'Azur|BOLD:AAB4064
Ectoedemia albifasciella|RMNH.INS.11386|Netherlands.South Holland|BOLD:AAB4064
Ectoedemia albifasciella|RMNH.INS.23486|Italy.Piedmont|BOLD:AAB4064
Ectoedemia albifasciella|RMNH.INS.12975|France.Provence-Alpes-Cote d'Azur|BOLD:AAB4064
Ectoedemia albifasciella|RMNH.INS.17855|United Kingdom.England|BOLD:AAB4064
Ectoedemia albifasciella|RMNH.INS.17914|United Kingdom.England|BOLD:AAB4064
Ectoedemia albifasciella|RMNH.INS.17778|Finland.Aland Islands|BOLD:AAB4064
Ectoedemia albifasciella|RMNH.INS.12999|France.Provence-Alpes-Cote d'Azur|BOLD:AAB4064
Ectoedemia albifasciella|RMNH.INS.17807|Finland.Aland Islands|BOLD:AAB4064
Ectoedemia albifasciella|RMNH.INS.12924|France.Provence-Alpes-Cote d'Azur|BOLD:AAB4064
Ectoedemia albifasciella|RMNH.INS.12902|France.Provence-Alpes-Cote d'Azur|BOLD:AAB4064
Ectoedemia albifasciella|TLMF Lep 18069|Austria.Tirol|BOLD:AAB4064
Ectoedemia albifasciella|KLM Lep 02908|Austria|BOLD:AAB4064
Ectoedemia albifasciella|TLMF Lep 14731|Austria|BOLD:AAB4064
Ectoedemia albifasciella|KLM Lep 02913|Austria|BOLD:AAB4064
Ectoedemia albifasciella|KLM Lep 02893|Slovenia|BOLD:AAB4064
Ectoedemia albifasciella|TLMF Lep 18201|Austria.Tirol|BOLD:AAB4064
Ectoedemia albifasciella|KLM Lep 02887|Slovenia|BOLD:AAB4064
Ectoedemia albifasciella|BC ZSM Lep 89457|Germany.Bavaria|BOLD:AAB4064
Ectoedemia albifasciella|BC ZSM Lep 79364|Germany.Bavaria|
Ectoedemia albifasciella|RMNH.INS.30257|Portugal.Minho|BOLD:AAB4064
Ectoedemia albifasciella|RMNH.INS.12798|Netherlands.South Holland|BOLD:AAB4064
Ectoedemia albifasciella|TLMF Lep 15112|Austria.Tirol|BOLD:AAB4064
Ectoedemia albifasciella|KLM Lep 01980|Austria|BOLD:AAB4064
Ectoedemia albifasciella|KLM Lep 01972|Slovenia|BOLD:AAB4064
Ectoedemia albifasciella|RMNH.INS.12949|Italy.Piedmont|BOLD:AAB4064
Ectoedemia contorta|RMNH.INS.30408|Cyprus|BOLD:ACU5570
Ectoedemia albifasciella|RMNH.INS.18865|Greece|BOLD:ABZ3177
Ectoedemia albifasciella|RMNH.INS.12836|Netherlands.Limburg|BOLD:ABZ3177
Ectoedemia albifasciella|RMNH.INS.12644|Netherlands.South Holland|BOLD:ABZ3177
Ectoedemia albifasciella|RMNH.INS.17819|Netherlands.Gelderland|BOLD:ABZ3177
Ectoedemia albifasciella|KLM Lep 00927|Austria.Carinthia|BOLD:ABZ3177
Ectoedemia albifasciella|BC ZSM Lep 79365|Germany.Bavaria|BOLD:ABZ3177
Ectoedemia albifasciella|RMNH.INS.11925|Germany.Lower Saxony|BOLD:ABZ3177
Ectoedemia albifasciella|KLM Lep 01970|Slovenia|BOLD:ABZ3177
Ectoedemia albifasciella|RMNH.INS.11427|Netherlands.Utrecht|BOLD:ABZ3177
Ectoedemia albifasciella|RMNH.INS.12651|Netherlands.Gelderland|BOLD:ABZ3177
Ectoedemia contorta|RMNH.INS.[illegible]|[illegible]|BOLD:[illegible]

Ectoedemia albifasciella|KLM Lep 01970|Slovenia|BOLD:ABZ3177
Ectoedemia albifasciella|RMNH.INS.11427|Netherlands.Utrecht|BOLD:ABZ3177
Ectoedemia albifasciella|RMNH.INS.12651|Netherlands.Gelderland|BOLD:ABZ3177
Ectoedemia contorta|RMNH.INS.23881|Croatia|BOLD:AAH4666
Ectoedemia contorta|RMNH.INS.23835|Croatia|BOLD:AAH4666
Ectoedemia contorta|KLM Lep 02890|Slovenia|BOLD:AAH4666
Ectoedemia contorta|TLMF Lep 15476|Italy.South Tyrol|BOLD:AAH4666
Ectoedemia contorta|BC ZSM Lep 64204|Germany.Bavaria|BOLD:AAH4666
Ectoedemia contorta|TLMF Lep 15475|Italy.South Tyrol|BOLD:AAH4666
Ectoedemia contorta|TLMF Lep 15573|Italy.South Tyrol|BOLD:AAH4666
Ectoedemia contorta|RMNH.INS.12948|Italy.Piedmont|BOLD:AAH4666
Ectoedemia contorta|TLMF Lep 15574|Italy.South Tyrol|BOLD:AAH4666
Ectoedemia contorta|TLMF Lep 17951|Italy.South Tyrol|BOLD:AAH4666
Ectoedemia contorta|TLMF Lep 03034|Italy.South Tyrol|BOLD:AAH4666
Ectoedemia contorta|TLMF Lep 16523|Italy.South Tyrol|BOLD:AAH4666
Ectoedemia cerris|RMNH.INS.24593|Italy.Campania|BOLD:ACU5569
Ectoedemia cerris|RMNH.INS.23836|Czech Republic.Southern Moravia|BOLD:ABZ3179
Ectoedemia cerris|RMNH.INS.23880|Czech Republic.Southern Moravia|BOLD:ABZ3179
Ectoedemia cerris|KLM Lep 02889|Slovenia|BOLD:ABZ3179
Ectoedemia cerris|KLM Lep 02894|Slovenia|BOLD:ABZ3179
Ectoedemia cerris|KLM Lep 01971|Slovenia|BOLD:ABZ3179
Ectoedemia albifasciella|RMNH.INS.23762|Morocco.Tadla-Azilal Region|BOLD:ACE3196
Ectoedemia pubescivora|RMNH.INS.30414|Cyprus|BOLD:ACU5568
Ectoedemia pubescivora|RMNH.INS.24007|Italy.Sicily|BOLD:ACE4977
Ectoedemia pubescivora|RMNH.INS.12900|France.Provence-Alpes-Cote d'Azur|BOLD:ACE4977
Ectoedemia pubescivora|RMNH.INS.23752|Italy.Piedmont|BOLD:ACE4977
Ectoedemia pubescivora|RMNH.INS.23621|Italy.Veneto|BOLD:ACE4977
Ectoedemia pubescivora|RMNH.INS.23620|Italy.Veneto|BOLD:ACE4977
Ectoedemia QuercusmongolicaChina|RMNH.INS.30145|South Korea|BOLD:ACU7123
Ectoedemia QuercusmongolicaChina|RMNH.INS.30072|China.Beijing Shi|BOLD:ACU7123
Ectoedemia QuercusmongolicaKorea|RMNH.INS.30197|South Korea|
Ectoedemia QuercusmongolicaKorea|RMNH.INS.30137|South Korea|BOLD:ACU5750
Ectoedemia rufifrontella|RMNH.INS.17677|France.Provence-Alpes-Cote d'Azur|BOLD:AAE8131
Ectoedemia rufifrontella|RMNH.INS.30413|Cyprus|BOLD:AAE8131
Ectoedemia rufifrontella|KLM Lep 01961|Slovenia|BOLD:AAE8131
Ectoedemia rufifrontella|RMNH.INS.23614|Italy.Veneto|BOLD:AAE8131
Ectoedemia rufifrontella|TLMF Lep 16521|Italy.South Tyrol|BOLD:AAE8131
Ectoedemia rufifrontella|RMNH.INS.23629|France.Provence-Alpes-Cote d'Azur|BOLD:AAE8131
Ectoedemia rufifrontella|RMNH.INS.23666|France.Languedoc-Roussillon|BOLD:AAE8131
Ectoedemia rufifrontella|KLM Lep 01969|Slovenia|BOLD:AAE8131
Ectoedemia alnifoliae|RMNH.INS.24634|Cyprus|BOLD:ACU7410
Ectoedemia alnifoliae|RMNH.INS.11889|Turkey.Antalya|BOLD:AAF6392
Ectoedemia alnifoliae|RMNH.INS.23883|Turkey.Antalya|BOLD:AAF6393
Ectoedemia alnifoliae|RMNH.INS.11893|Turkey.Antalya|BOLD:AAF6393
Ectoedemia Quercuscastaneifolia|RMNH.INS.29907|Azerbaijan|BOLD:ACH1680
Ectoedemia heringi|RMNH.INS.24308|Greece|BOLD:ACG8548
Ectoedemia heringi|RMNH.INS.29050|Greece|BOLD:ACF1459
Ectoedemia heringi|RMNH.INS.18963|Greece|BOLD:ACF1459
Ectoedemia heringi|RMNH.INS.18965|Greece|BOLD:ACF1459
Ectoedemia subbimaculella|RMNH.INS.18954|Greece|BOLD:ACF1459
Ectoedemia subbimaculella|RMNH.INS.24389|Greece|BOLD:ACF1459
Ectoedemia subbimaculella|RMNH.INS.18894|Greece|BOLD:ACF1459
Ectoedemia heringi|RMNH.INS.23514|Italy.Piedmont|BOLD:ACF1459
Ectoedemia heringi|RMNH.INS.17618|France.Provence-Alpes-Cote d'Azur|BOLD:ACF1459
Ectoedemia heringi|KLM Lep 02888|Slovenia|BOLD:ACF1459
Ectoedemia heringi|RMNH.INS.17885|France.Alsace|BOLD:ACF1459
Ectoedemia heringi|RMNH.INS.17846|Poland|BOLD:ACF1459
Ectoedemia heringi|RMNH.INS.23907|France.Alsace|BOLD:ACF1459
Ectoedemia heringi|TLMF Lep 04517|Italy.South Tyrol|BOLD:ACF1459
Ectoedemia heringi|TLMF Lep 15482|Italy.South Tyrol|BOLD:ACF1459
Ectoedemia heringi|TLMF Lep 15481|Italy.South Tyrol|BOLD:ACF1459
Ectoedemia heringi|RMNH.INS.23906|Belgium.Luik (Liege)|BOLD:ACF1459
Ectoedemia liechtensteini|RMNH.INS.11418|Czech Republic|BOLD:ACF1459
Ectoedemia liechtensteini|RMNH.INS.18138|Bulgaria.Sofiya|BOLD:ACF1459
Ectoedemia liechtensteini|RMNH.INS.17890|Czech Republic|BOLD:ACF1459
Ectoedemia liechtensteini|KLM Lep 01963|Slovenia|BOLD:ACF1459
Ectoedemia liechtensteini|RMNH.INS.11417|Czech Republic|BOLD:ACF1459
Ectoedemia phyllotomella|RMNH.INS.11414|Italy.Tuscany|BOLD:ACF1459
Ectoedemia phyllotomella|RMNH.INS.11416|Italy.Tuscany|BOLD:ACF1459
Ectoedemia heringi|RMNH.INS.23908|Spain.Andalusia|BOLD:ACF1460
Ectoedemia heringi|RMNH.INS.23753|Germany.Rhineland-Palatinate|BOLD:AAB7903
Ectoedemia heringi|RMNH.INS.23905|Netherlands.Limburg|BOLD:AAB7903
Ectoedemia heringi|RMNH.INS.11353|Netherlands.Limburg|BOLD:AAB7903
Ectoedemia heringi|RMNH.INS.11352|Netherlands.Limburg|BOLD:AAB7903
Ectoedemia heringi|RMNH.INS.23904|Belgium.Luik (Liege)|BOLD:AAB7903
Ectoedemia heringi|RMNH.INS.11356|Belgium.Luik (Liege)|BOLD:AAB7903
Ectoedemia heringi|RMNH.INS.23910|Germany.Rhineland-Palatinate|BOLD:AAB7903
Ectoedemia heringi|RMNH.INS.24221|Greece|BOLD:ACG9547
Ectoedemia heringi|RMNH.INS.23903|Italy.Veneto|BOLD:ACF0249
Ectoedemia heringi|RMNH.INS.18846|Greece|BOLD:ABZ4694
Ectoedemia heringi|RMNH.INS.18857|Greece|BOLD:ABZ4694
Ectoedemia heringi|RMNH.INS.17766|Greece.Peloponnese|BOLD:ABZ4694
Ectoedemia subbimaculella|RMNH.INS.23843|Hungary|BOLD:ABZ4694
Ectoedemia heringi|RMNH.INS.17767|Greece.Peloponnese|BOLD:ABZ4694
Ectoedemia subbimaculella|RMNH.INS.23882|Hungary|BOLD:ABZ4694
Ectoedemia subbimaculella|RMNH.INS.24006|Italy.Sicily|BOLD:ACE7752
Ectoedemia subbimaculella|RMNH.INS.11383|Netherlands.South Holland|BOLD:ACE7752
Ectoedemia subbimaculella|RMNH.INS.17864|United Kingdom.England|BOLD:ACE7752
Ectoedemia subbimaculella|KLM Lep 01253|Austria.Carinthia|BOLD:ACE7752
Ectoedemia subbimaculella|RMNH.INS.12945|Italy.Piedmont|BOLD:ACE7752
Ectoedemia subbimaculella|RMNH.INS.23661|France.Languedoc-Roussillon|BOLD:ACE7752
Ectoedemia subbimaculella|KLM Lep 01922|Slovenia|BOLD:ACE7752
Ectoedemia subbimaculella|TLMF Lep 14730|Austria|BOLD:ACE7752
Ectoedemia subbimaculella|RMNH.INS.12972|France.Provence-Alpes-Cote d'Azur|BOLD:ACE7752
Ectoedemia subbimaculella|RMNH.INS.23676|Italy.Piedmont|BOLD:ACE7752
Ectoedemia subbimaculella|MM09639|Finland.Aland Islands|BOLD:ACE7752
Ectoedemia subbimaculella|KLM Lep 02923|Austria.Carinthia|BOLD:ACE7752
Ectoedemia subbimaculella|BC ZSM Lep 89456|Germany.Bavaria|BOLD:ACE7752
Ectoedemia subbimaculella|BC ZSM Lep 50945|Germany.Bavaria|BOLD:ACE7752
Ectoedemia subbimaculella|BC ZSM Lep 89425|Germany.Bavaria|BOLD:ACE7752
Ectoedemia subbimaculella|BC ZSM Lep 89155|Germany.Bavaria|BOLD:ACE7752

[illegible]
Ectoedemia subbimaculella|BC ZSM Lep 50945|Germany.Bavaria|BOLD:ACE7752
Ectoedemia subbimaculella|BC ZSM Lep 89425|Germany.Bavaria|BOLD:ACE7752
Ectoedemia subbimaculella|BC ZSM Lep 89155|Germany.Bavaria|BOLD:ACE7752
Ectoedemia subbimaculella|KLM Lep 02907|Austria.Carinthia|BOLD:ACE7752
Ectoedemia subbimaculella|BC ZSM Lep 70766|Germany.Bavaria|BOLD:ACE7752
Ectoedemia subbimaculella|RMNH.INS.11766|France.Lorraine|BOLD:ACE7752
Ectoedemia subbimaculella|KLM Lep 02927|Austria|BOLD:ACE7752
Ectoedemia subbimaculella|BC ZSM Lep 63890|Germany.Bavaria|BOLD:ACE7752
Ectoedemia subbimaculella|TLMF Lep 16739|Austria.Tirol|BOLD:ACE7752
Ectoedemia subbimaculella|RMNH.INS.11329|Netherlands.Friesland|BOLD:ACE7752
Ectoedemia subbimaculella|RMNH.INS.11765|France.Lorraine|BOLD:ACE7752
Ectoedemia subbimaculella|RMNH.INS.17865|United Kingdom.England|BOLD:ACE7752
Ectoedemia subbimaculella|RMNH.INS.23665|France.Languedoc-Roussillon|BOLD:ACE7752
Ectoedemia subbimaculella|RMNH.INS.23909|France.Lorraine|BOLD:ACE7752
Ectoedemia subbimaculella|RMNH.INS.23671|Germany.Brandenburg|BOLD:ACE7752
Ectoedemia subbimaculella|RMNH.INS.17780|Finland.Aland Islands|BOLD:ACE7752
Ectoedemia subbimaculella|KLM Lep 02925|Austria|BOLD:ACE7752
Ectoedemia subbimaculella|RMNH.INS.17921|United Kingdom.England|BOLD:ACE7752
Ectoedemia cerviparadisicola|RMNH.INS.17902|Japan.Honshu|BOLD:AAI6174
Ectoedemia cerviparadisicola|RMNH.INS.17904|Japan.Honshu|BOLD:AAI6174
Ectoedemia cerviparadisicola|RMNH.INS.17903|Japan.Honshu|BOLD:AAI6174
Ectoedemia QuercusJapan|RMNH.INS.29385|Taiwan|BOLD:ACG8489
Ectoedemia QuercusJapan|RMNH.INS.24752|Japan.Honshu|BOLD:ACG8489
Ectoedemia QuercusJapan|RMNH.INS.29536|Taiwan|BOLD:ACG8489
Ectoedemia TaiwanEvN4064|RMNH.INS.24064|Taiwan.Nantou County|BOLD:ACG9368
Ectoedemia arisi|RMNH.INS.29358|Taiwan|BOLD:ACG9190
Ectoedemia arisi|RMNH.INS.23887|China.Henan|BOLD:AAX9877
Ectoedemia arisi|RMNH.INS.29976|Japan.Honshu|BOLD:ACU6755
Ectoedemia arisi|RMNH.INS.29865|Japan.Honshu|BOLD:ACU6755
Ectoedemia arisi|RMNH.INS.24719|Japan.Honshu|BOLD:ACU6755
Ectoedemia JuglansJapan|RMNH.INS.29975|Japan.Honshu|BOLD:ACY4613
Ectoedemia JuglansJapan|RMNH.INS.30373|Japan.Honshu|BOLD:ACY4613
Ectoedemia PterocaryaJapan|RMNH.INS.30319|Japan.Honshu|BOLD:ACU6044
Ectoedemia PterocaryaJapan|RMNH.INS.29856|Japan.Honshu|BOLD:ACU6044
Ectoedemia trinotata|DNA-ATBI-3012|United States.Tennessee|BOLD:AAH4504
Ectoedemia trinotata|RMNH.INS.18297|United States.Tennessee|BOLD:AAH4504
Ectoedemia trinotata|CNCLEP00042451|United States.Maryland|BOLD:AAH4504
Ectoedemia trinotata|RMNH.INS.18350|United States.Mississippi|BOLD:AAH4504
Ectoedemia trinotata|CNCLEP00042508|United States.Maryland|BOLD:AAH4504
Ectoedemia trinotata|RMNH.INS.24398|United States.Vermont|BOLD:AAH4504
Ectoedemia trinotata|CNCLEP00042507|United States.Maryland|BOLD:AAH4504
Ectoedemia trinotata|CNCLEP00042452|United States.Maryland|BOLD:AAH4504
Ectoedemia trinotata|CNCLEP00042453|United States.Maryland|BOLD:AAH4504
Ectoedemia trinotata|RMNH.INS.24397|United States.Connecticut|BOLD:AAH4504
Ectoedemia populella|AC005189|Canada.Quebec|BOLD:AAB9665
Ectoedemia populella|jflandry2321|Canada.Quebec|BOLD:AAB9665
Ectoedemia populella|AC005175|Canada.Quebec|BOLD:AAB9665
Ectoedemia populella|jflandry2032|Canada.Quebec|BOLD:AAB9665
Ectoedemia populella|2006-ONT-0377|Canada.Ontario|BOLD:AAB9665
Ectoedemia populella|jflandry2324|Canada.Quebec|BOLD:AAB9665
Ectoedemia populella|jflandry2322|Canada.Quebec|BOLD:AAB9665
Ectoedemia populella|jflandry2320|Canada.Quebec|BOLD:AAB9665
Ectoedemia populella|jflandry2323|Canada.Quebec|BOLD:AAB9665
Ectoedemia populella|jflandry2319|Canada.Quebec|BOLD:AAB9665
Ectoedemia populella|RMNH.INS.23885|United States.Connecticut|
Ectoedemia populella|RMNH.INS.23886|United States.Connecticut|BOLD:AAB9666
Ectoedemia populella|RMNH.INS.18505|United States.Connecticut|BOLD:AAB9666
Ectoedemia populella|RMNH.INS.24399|United States.Connecticut|BOLD:AAB9666
Ectoedemia populella|AC005132|Canada.Quebec|BOLD:AAB9666
Ectoedemia populella|AC005029|Canada.Quebec|BOLD:AAB9666
Ectoedemia populella|AC005005|Canada.Quebec|BOLD:AAB9666
Ectoedemia populella|AC005019|Canada.Quebec|BOLD:AAB9666
Ectoedemia insularis|RMNH.INS.24332|South Korea|BOLD:AAD0468
Ectoedemia insularis|RMNH.INS.23890|Japan.Hokkaido|BOLD:AAD0468
Ectoedemia insularis|RMNH.INS.24333|South Korea|BOLD:AAD0468
Ectoedemia insularis|RMNH.INS.30117|South Korea|BOLD:AAD0468
Ectoedemia insularis|RMNH.INS.30187|South Korea|BOLD:AAD0468
Ectoedemia intimella|RMNH.INS.11373|Netherlands.South Holland|BOLD:AAD0467
Ectoedemia intimella|BC ZSM Lep 70738|Germany.Bavaria|BOLD:AAD0467
Ectoedemia intimella|RMNH.INS.12773|Netherlands.North Holland|BOLD:AAD0467
Ectoedemia intimella|MM18061|Finland|BOLD:AAD0467
Ectoedemia intimella|MM07013|Finland|BOLD:AAD0467
Ectoedemia intimella|MM01845|Finland.Uusimaa|BOLD:AAD0467
Ectoedemia intimella|RMNH.INS.11737|France.Lorraine|BOLD:AAD0467
Ectoedemia intimella|RMNH.INS.17642|Italy.Piedmont|BOLD:AAD0467
Ectoedemia intimella|MM23779|Finland|BOLD:AAD0467
Ectoedemia intimella|RMNH.INS.17776|Finland.Uusimaa|BOLD:AAD0467
Ectoedemia canutus|RMNH.INS.24061|United States.Arizona|BOLD:AAF6428
Ectoedemia canutus|RMNH.INS.23727|United States.California|BOLD:AAF6428
Ectoedemia canutus|RMNH.INS.24051|United States.California|BOLD:AAF6428
Ectoedemia canutus|RMNH.INS.23725|Canada.Alberta|BOLD:AAF6428
Ectoedemia canutus|jflandry0493|Canada.Quebec|BOLD:AAF6428
Ectoedemia nrcanutus|RMNH.INS.23723|Canada.Alberta|BOLD:AAF6429
Ectoedemia hannoverella|TLMF Lep 09354|Austria.Tirol|BOLD:AAE8153
Ectoedemia hannoverella|RMNH.INS.17838|Germany.Brandenburg|BOLD:AAE8153
Ectoedemia hannoverella|RMNH.INS.11738|France.Alsace|BOLD:AAE8153
Ectoedemia hannoverella|BC ZSM Lep 89440|Germany.Bavaria|BOLD:AAE8153
Ectoedemia hannoverella|BC ZSM Lep 89458|Germany.Bavaria|BOLD:AAE8153
Ectoedemia hannoverella|RMNH.INS.24312|Turkey.Nevsehir|BOLD:AAE8153
Ectoedemia hannoverella|RMNH.INS.12810|Netherlands.North Brabant|BOLD:AAE8153
Ectoedemia hannoverella|RMNH.INS.12856|France.Provence-Alpes-Cote d'Azur|BOLD:AAE8153
Ectoedemia hannoverella|BC ZSM Lep 79372|Germany.Bavaria|BOLD:AAE8153
Ectoedemia hannoverella|BC ZSM Lep 64236|Germany.Bavaria|BOLD:AAE8153
Ectoedemia hannoverella|TLMF Lep 07376|Austria.Tirol|BOLD:AAE8153
Ectoedemia hannoverella|TLMF Lep 07334|Austria.Tirol|BOLD:AAE8153
Ectoedemia hannoverella|BC ZSM Lep 76422|Germany.Bavaria|BOLD:AAE8153
Ectoedemia hannoverella|BC ZSM Lep 79378|Germany.Bavaria|BOLD:AAE8153
Ectoedemia sinevi|RMNH.INS.30164|South Korea|BOLD:ACU7356
Ectoedemia sinevi|RMNH.INS.30165|South Korea|BOLD:ACU7356
Ectoedemia sinevi|RMNH.INS.24651|Japan.Honshu|BOLD:ACU7356

[illegible]
Ectoedemia sinevi|RMNH.INS.30164|South Korea|BOLD:ACU7356
Ectoedemia sinevi|RMNH.INS.30165|South Korea|BOLD:ACU7356
Ectoedemia sinevi|RMNH.INS.24651|Japan.Honshu|BOLD:ACU7356
Ectoedemia argyropeza|BC ZSM Lep 50947|Germany.Bavaria|
Ectoedemia argyropeza|RMNH.INS.24717|South Korea|BOLD:ACU7035
Ectoedemia argyropeza|RMNH.INS.30209|South Korea|BOLD:ACU7035
Ectoedemia argyropeza|RMNH.INS.24751|Japan.Honshu|BOLD:ACU7035
Ectoedemia argyropeza|RMNH.INS.12889|France.Provence-Alpes-Cote d'Azur|BOLD:AAC1036
Ectoedemia argyropeza|RMNH.INS.24310|Bulgaria.Blagoevgrad|BOLD:AAC1036
Ectoedemia argyropeza|RMNH.INS.24295|Georgia|BOLD:ACG8449
Ectoedemia argyropeza|RMNH.INS.24297|Georgia|BOLD:ACG8449
Ectoedemia argyropeza|AC005346|Canada.Quebec|BOLD:AAC1036
Ectoedemia argyropeza|AC005371|Canada.Quebec|BOLD:AAC1036
Ectoedemia argyropeza|TLMF Lep 10399|Austria.Tirol|BOLD:AAC1036
Ectoedemia argyropeza|MM13758|Finland|BOLD:AAC1036
Ectoedemia argyropeza|KLM Lep 02918|Austria.Carinthia|BOLD:AAC1036
Ectoedemia argyropeza|RMNH.INS.11741|France.Alsace|BOLD:AAC1036
Ectoedemia argyropeza|RMNH.INS.11689|France.Lorraine|BOLD:AAC1036
Ectoedemia argyropeza|RMNH.INS.17774|Finland.Uusimaa|BOLD:AAC1036
Ectoedemia argyropeza|jflandry2037|Canada.Quebec|BOLD:AAC1036
Ectoedemia argyropeza|jflandry2038|Canada.Quebec|BOLD:AAC1036
Ectoedemia argyropeza|AC005342|Canada.Quebec|BOLD:AAC1036
Ectoedemia argyropeza|RMNH.INS.18675|United States.New York|BOLD:AAC1036
Ectoedemia argyropeza|BC ZSM Lep 89178|Germany.Bavaria|BOLD:AAC1036
Ectoedemia argyropeza|jflandry2034|Canada.Quebec|BOLD:AAC1036
Ectoedemia argyropeza|jflandry2036|Canada.Quebec|BOLD:AAC1036
Ectoedemia argyropeza|BC ZSM Lep 89487|Germany.Bavaria|BOLD:AAC1036
Ectoedemia argyropeza|BC ZSM Lep 89376|Germany.Bavaria|BOLD:AAC1036
Ectoedemia argyropeza|MM15195|Finland|BOLD:AAC1036
Ectoedemia argyropeza|TLMF Lep 15292|Austria.Tirol|BOLD:AAC1036
Ectoedemia argyropeza|TLMF Lep 15291|Austria.Tirol|BOLD:AAC1036
Ectoedemia argyropeza|TLMF Lep 17843|Austria.Tirol|BOLD:AAC1036
Ectoedemia argyropeza|TLMF Lep 15293|Austria.Tirol|BOLD:AAC1036
Ectoedemia argyropeza|TLMF Lep 17839|Austria.Tirol|BOLD:AAC1036
Ectoedemia argyropeza|BC ZSM Lep 89486|Germany.Bavaria|BOLD:AAC1036
Ectoedemia argyropeza|BC ZSM Lep 79386|Germany.Bavaria|
Ectoedemia argyropeza|BC ZSM Lep 50963|Germany.Bavaria|BOLD:AAC1036
Ectoedemia argyropeza|jflandry2035|Canada.Quebec|BOLD:AAC1036
Ectoedemia argyropeza|MM15194|Finland|BOLD:AAC1036
Ectoedemia argyropeza|jflandry2033|Canada.Quebec|BOLD:AAC1036
Ectoedemia argyropeza|TLMF Lep 17838|Austria.Tirol|BOLD:AAC1036
Ectoedemia argyropeza|TLMF Lep 17841|Austria.Tirol|BOLD:AAC1036
Ectoedemia argyropeza|RMNH.INS.11762|France.Alsace|BOLD:AAC1036
Ectoedemia argyropeza|RMNH.INS.17622|Italy.Piedmont|BOLD:AAC1036
Ectoedemia argyropeza|BC ZSM Lep 79382|Germany.Bavaria|BOLD:AAC1036
Ectoedemia argyropeza|BC ZSM Lep 89403|Germany.Bavaria|
Ectoedemia klimeschi|RMNH.INS.17827|Germany.Brandenburg|BOLD:ABX4998
Ectoedemia klimeschi|RMNH.INS.24299|Greece|BOLD:ABX4998
Ectoedemia klimeschi|RMNH.INS.17828|Germany.Brandenburg|BOLD:ABX4998
Ectoedemia turbidella|RMNH.INS.17834|Germany.Brandenburg|BOLD:AAD4374
Ectoedemia turbidella|BC ZSM Lep 79376|Germany.Bavaria|BOLD:AAD4374
Ectoedemia turbidella|MM09901|Finland|BOLD:AAD4374
Ectoedemia turbidella|RMNH.INS.17833|Germany.Brandenburg|BOLD:AAD4374
Ectoedemia turbidella|RMNH.INS.11392|Netherlands.South Holland|BOLD:AAD4374
Ectoedemia turbidella|BC ZSM Lep 70745|Germany.Bavaria|BOLD:AAD4374
Ectoedemia turbidella|MM09900|Finland|BOLD:AAD4374
Ectoedemia turbidella|MM09899|Finland|BOLD:AAD4374
Ectoedemia turbidella|RMNH.INS.23924|Ukraine|
Ectoedemia turbidella|RMNH.INS.11320|Netherlands.South Holland|BOLD:AAD4374
Ectoedemia angulifasciella|BC ZSM Lep 50944|Germany.Bavaria|
Ectoedemia angulifasciella|MM00885|Finland.Aland Islands|BOLD:AAB6754
Ectoedemia angulifasciella|RMNH.INS.12784|Sweden.Skane|BOLD:AAB6754
Ectoedemia angulifasciella|RMNH.INS.12762|Sweden|BOLD:AAB6754
Ectoedemia angulifasciella|RMNH.INS.12763|Sweden|BOLD:AAB6754
Ectoedemia angulifasciella|RMNH.INS.12765|Sweden|BOLD:AAB6754
Ectoedemia angulifasciella|RMNH.INS.12761|Sweden|BOLD:AAB6754
Ectoedemia angulifasciella|RMNH.INS.12764|Sweden|BOLD:AAB6754
Ectoedemia angulifasciella|RMNH.INS.12766|Sweden|BOLD:AAB6754
Ectoedemia angulifasciella|RMNH.INS.12760|Sweden|BOLD:AAB6754
Ectoedemia angulifasciella|RMNH.INS.12767|Sweden|BOLD:AAB6754
Ectoedemia angulifasciella|RMNH.INS.17918|United Kingdom.England|BOLD:AAB6754
Ectoedemia angulifasciella|RMNH.INS.17803|Finland.Aland Islands|BOLD:AAB6754
Ectoedemia angulifasciella|RMNH.INS.12787|Sweden.Vastra Gotaland|BOLD:AAB6754
Ectoedemia angulifasciella|RMNH.INS.11769|France.Alsace|BOLD:AAB6754
Ectoedemia angulifasciella|RMNH.INS.11768|France.Alsace|BOLD:AAB6754
Ectoedemia angulifasciella|RMNH.INS.12885|France.Provence-Alpes-Cote d'Azur|BOLD:AAB6754
Ectoedemia angulifasciella|RMNH.INS.17861|United Kingdom.England|BOLD:AAB6754
Ectoedemia angulifasciella|TLMF Lep 15214|Austria.Tirol|BOLD:AAB6754
Ectoedemia angulifasciella|BC ZSM Lep 89395|Germany.Bavaria|BOLD:AAB6754
Ectoedemia angulifasciella|TLMF Lep 15300|Austria.Tirol|BOLD:AAB6754
Ectoedemia angulifasciella|RMNH.INS.12917|France.Provence-Alpes-Cote d'Azur|BOLD:AAB6754
Ectoedemia angulifasciella|RMNH.INS.11290|Germany.Rhineland-Palatinate|BOLD:AAB6754
Ectoedemia angulifasciella|MM09617|Finland.Aland Islands|BOLD:AAB6754
Ectoedemia angulifasciella|MM14866|Finland|BOLD:AAB6754
Ectoedemia angulifasciella|KLM Lep 00282|Austria.Carinthia|BOLD:AAB6754
Ectoedemia angulifasciella|KLM Lep 00302|Austria.Carinthia|BOLD:AAB6754
Ectoedemia angulifasciella|KLM Lep 00299|Austria.Carinthia|BOLD:AAB6754
Ectoedemia angulifasciella|RMNH.INS.18760|Greece|BOLD:AAB6754
Ectoedemia PourthiaeaJapan|RMNH.INS.30396|Japan.Honshu|BOLD:ACU5506
Ectoedemia PourthiaeaJapan|RMNH.INS.29874|Japan.Honshu|BOLD:ACU5506
Ectoedemia PourthiaeaTaiwan|RMNH.INS.29353|Taiwan.Yilan County|BOLD:ACG8516
Ectoedemia PourthiaeaTaiwan|RMNH.INS.29402|Taiwan.Hualien City|BOLD:ACG8516
Ectoedemia pilosae|RMNH.INS.29739|Japan.Hokkaido|BOLD:AAP1383
Ectoedemia pilosae|RMNH.INS.17893|China.Heilongjiang Sheng|
Ectoedemia pilosae|RMNH.INS.29257|South Korea|BOLD:AAP1383
Ectoedemia pilosae|RMNH.INS.29256|South Korea|BOLD:AAP1383
Ectoedemia RosaTaiwan|RMNH.INS.29404|Taiwan.Hualien City|BOLD:ACG9450
Ectoedemia RosaTaiwan|RMNH.INS.29415|Taiwan.Nantou County|BOLD:ACG9450
Ectoedemia expeditionis|RMNH.INS.23987|Namibia|BOLD:AAK9936
Ectoedemia PrunusJapan|RMNH.INS.29877|Japan.Honshu|BOLD:ACU6784

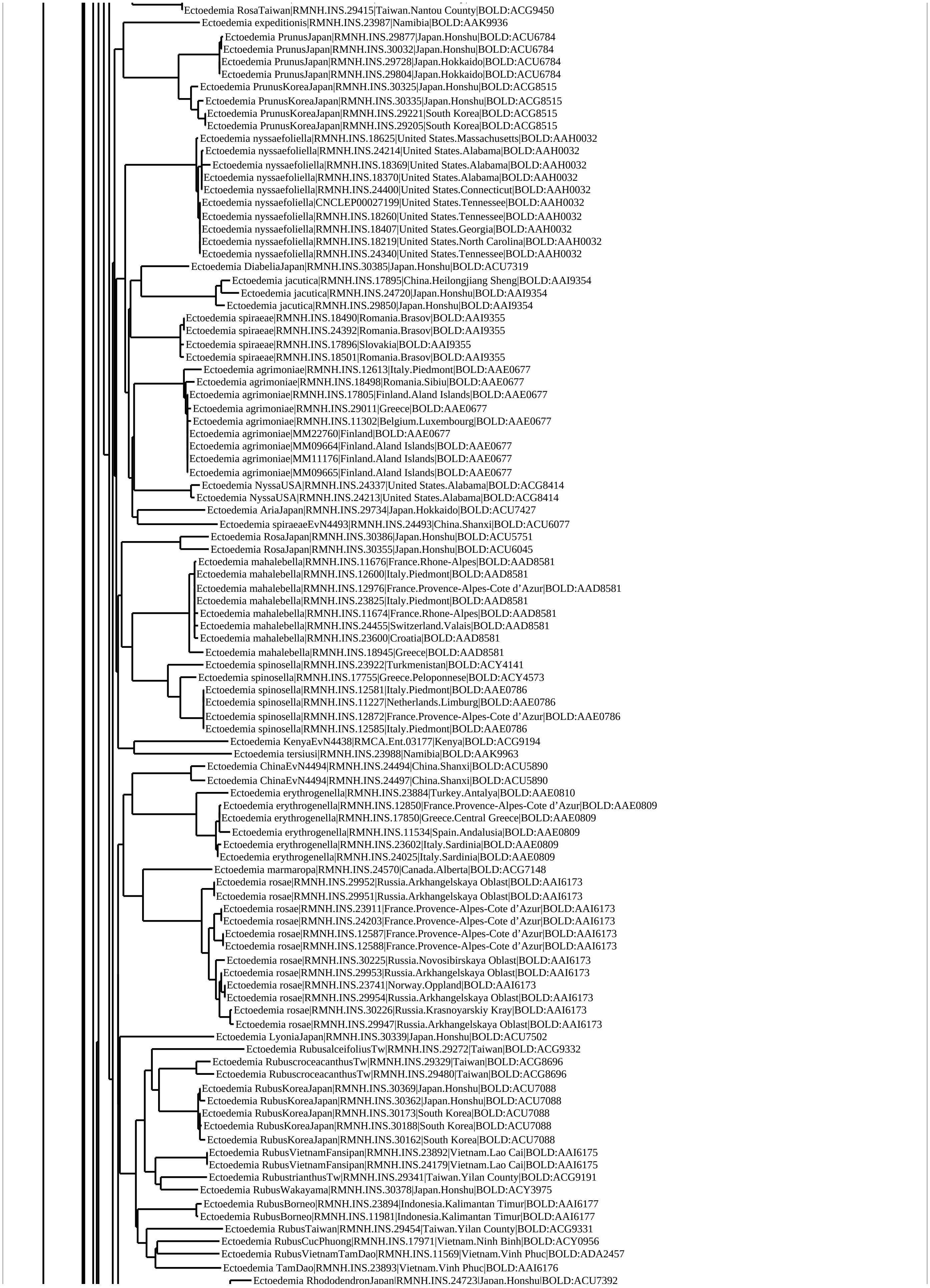
Ectoedemia RosaTaiwan|RMNH.INS.29415|Taiwan.Nantou County|BOLD:ACG9450
Ectoedemia expeditionis|RMNH.INS.23987|Namibia|BOLD:AAK9936
Ectoedemia PrunusJapan|RMNH.INS.29877|Japan.Honshu|BOLD:ACU6784
Ectoedemia PrunusJapan|RMNH.INS.30032|Japan.Honshu|BOLD:ACU6784
Ectoedemia PrunusJapan|RMNH.INS.29728|Japan.Hokkaido|BOLD:ACU6784
Ectoedemia PrunusJapan|RMNH.INS.29804|Japan.Hokkaido|BOLD:ACU6784
Ectoedemia PrunusKoreaJapan|RMNH.INS.30325|Japan.Honshu|BOLD:ACG8515
Ectoedemia PrunusKoreaJapan|RMNH.INS.30335|Japan.Honshu|BOLD:ACG8515
Ectoedemia PrunusKoreaJapan|RMNH.INS.29221|South Korea|BOLD:ACG8515
Ectoedemia PrunusKoreaJapan|RMNH.INS.29205|South Korea|BOLD:ACG8515
Ectoedemia nyssaefoliella|RMNH.INS.18625|United States.Massachusetts|BOLD:AAH0032
Ectoedemia nyssaefoliella|RMNH.INS.24214|United States.Alabama|BOLD:AAH0032
Ectoedemia nyssaefoliella|RMNH.INS.18369|United States.Alabama|BOLD:AAH0032
Ectoedemia nyssaefoliella|RMNH.INS.18370|United States.Alabama|BOLD:AAH0032
Ectoedemia nyssaefoliella|RMNH.INS.24400|United States.Connecticut|BOLD:AAH0032
Ectoedemia nyssaefoliella|CNCLEP00027199|United States.Tennessee|BOLD:AAH0032
Ectoedemia nyssaefoliella|RMNH.INS.18260|United States.Tennessee|BOLD:AAH0032
Ectoedemia nyssaefoliella|RMNH.INS.18407|United States.Georgia|BOLD:AAH0032
Ectoedemia nyssaefoliella|RMNH.INS.18219|United States.North Carolina|BOLD:AAH0032
Ectoedemia nyssaefoliella|RMNH.INS.24340|United States.Tennessee|BOLD:AAH0032
Ectoedemia DiabeliaJapan|RMNH.INS.30385|Japan.Honshu|BOLD:ACU7319
Ectoedemia jacutica|RMNH.INS.17895|China.Heilongjiang Sheng|BOLD:AAI9354
Ectoedemia jacutica|RMNH.INS.24720|Japan.Honshu|BOLD:AAI9354
Ectoedemia jacutica|RMNH.INS.29850|Japan.Honshu|BOLD:AAI9354
Ectoedemia spiraeae|RMNH.INS.18490|Romania.Brasov|BOLD:AAI9355
Ectoedemia spiraeae|RMNH.INS.24392|Romania.Brasov|BOLD:AAI9355
Ectoedemia spiraeae|RMNH.INS.17896|Slovakia|BOLD:AAI9355
Ectoedemia spiraeae|RMNH.INS.18501|Romania.Brasov|BOLD:AAI9355
Ectoedemia agrimoniae|RMNH.INS.12613|Italy.Piedmont|BOLD:AAE0677
Ectoedemia agrimoniae|RMNH.INS.18498|Romania.Sibiu|BOLD:AAE0677
Ectoedemia agrimoniae|RMNH.INS.17805|Finland.Aland Islands|BOLD:AAE0677
Ectoedemia agrimoniae|RMNH.INS.29011|Greece|BOLD:AAE0677
Ectoedemia agrimoniae|RMNH.INS.11302|Belgium.Luxembourg|BOLD:AAE0677
Ectoedemia agrimoniae|MM22760|Finland|BOLD:AAE0677
Ectoedemia agrimoniae|MM09664|Finland.Aland Islands|BOLD:AAE0677
Ectoedemia agrimoniae|MM11176|Finland.Aland Islands|BOLD:AAE0677
Ectoedemia agrimoniae|MM09665|Finland.Aland Islands|BOLD:AAE0677
Ectoedemia NyssaUSA|RMNH.INS.24337|United States.Alabama|BOLD:ACG8414
Ectoedemia NyssaUSA|RMNH.INS.24213|United States.Alabama|BOLD:ACG8414
Ectoedemia AriaJapan|RMNH.INS.29734|Japan.Hokkaido|BOLD:ACU7427
Ectoedemia spiraeaeEvN4493|RMNH.INS.24493|China.Shanxi|BOLD:ACU6077
Ectoedemia RosaJapan|RMNH.INS.30386|Japan.Honshu|BOLD:ACU5751
Ectoedemia RosaJapan|RMNH.INS.30355|Japan.Honshu|BOLD:ACU6045
Ectoedemia mahalebella|RMNH.INS.11676|France.Rhone-Alpes|BOLD:AAD8581
Ectoedemia mahalebella|RMNH.INS.12600|Italy.Piedmont|BOLD:AAD8581
Ectoedemia mahalebella|RMNH.INS.12976|France.Provence-Alpes-Cote d’Azur|BOLD:AAD8581
Ectoedemia mahalebella|RMNH.INS.23825|Italy.Piedmont|BOLD:AAD8581
Ectoedemia mahalebella|RMNH.INS.11674|France.Rhone-Alpes|BOLD:AAD8581
Ectoedemia mahalebella|RMNH.INS.24455|Switzerland.Valais|BOLD:AAD8581
Ectoedemia mahalebella|RMNH.INS.23600|Croatia|BOLD:AAD8581
Ectoedemia mahalebella|RMNH.INS.18945|Greece|BOLD:AAD8581
Ectoedemia spinosella|RMNH.INS.23922|Turkmenistan|BOLD:ACY4141
Ectoedemia spinosella|RMNH.INS.17755|Greece.Peloponnese|BOLD:ACY4573
Ectoedemia spinosella|RMNH.INS.12581|Italy.Piedmont|BOLD:AAE0786
Ectoedemia spinosella|RMNH.INS.11227|Netherlands.Limburg|BOLD:AAE0786
Ectoedemia spinosella|RMNH.INS.12872|France.Provence-Alpes-Cote d’Azur|BOLD:AAE0786
Ectoedemia spinosella|RMNH.INS.12585|Italy.Piedmont|BOLD:AAE0786
Ectoedemia KenyaEvN4438|RMCA.Ent.03177|Kenya|BOLD:ACG9194
Ectoedemia tersiusi|RMNH.INS.23988|Namibia|BOLD:AAK9963
Ectoedemia ChinaEvN4494|RMNH.INS.24494|China.Shanxi|BOLD:ACU5890
Ectoedemia ChinaEvN4494|RMNH.INS.24497|China.Shanxi|BOLD:ACU5890
Ectoedemia erythrogenella|RMNH.INS.23884|Turkey.Antalya|BOLD:AAE0810
Ectoedemia erythrogenella|RMNH.INS.12850|France.Provence-Alpes-Cote d’Azur|BOLD:AAE0809
Ectoedemia erythrogenella|RMNH.INS.17850|Greece.Central Greece|BOLD:AAE0809
Ectoedemia erythrogenella|RMNH.INS.11534|Spain.Andalusia|BOLD:AAE0809
Ectoedemia erythrogenella|RMNH.INS.23602|Italy.Sardinia|BOLD:AAE0809
Ectoedemia erythrogenella|RMNH.INS.24025|Italy.Sardinia|BOLD:AAE0809
Ectoedemia marmaropa|RMNH.INS.24570|Canada.Alberta|BOLD:ACG7148
Ectoedemia rosae|RMNH.INS.29952|Russia.Arkhangelskaya Oblast|BOLD:AAI6173
Ectoedemia rosae|RMNH.INS.29951|Russia.Arkhangelskaya Oblast|BOLD:AAI6173
Ectoedemia rosae|RMNH.INS.23911|France.Provence-Alpes-Cote d’Azur|BOLD:AAI6173
Ectoedemia rosae|RMNH.INS.24203|France.Provence-Alpes-Cote d’Azur|BOLD:AAI6173
Ectoedemia rosae|RMNH.INS.12587|France.Provence-Alpes-Cote d’Azur|BOLD:AAI6173
Ectoedemia rosae|RMNH.INS.12588|France.Provence-Alpes-Cote d’Azur|BOLD:AAI6173
Ectoedemia rosae|RMNH.INS.30225|Russia.Novosibirskaya Oblast|BOLD:AAI6173
Ectoedemia rosae|RMNH.INS.29953|Russia.Arkhangelskaya Oblast|BOLD:AAI6173
Ectoedemia rosae|RMNH.INS.23741|Norway.Oppland|BOLD:AAI6173
Ectoedemia rosae|RMNH.INS.29954|Russia.Arkhangelskaya Oblast|BOLD:AAI6173
Ectoedemia rosae|RMNH.INS.30226|Russia.Krasnoyarskiy Kray|BOLD:AAI6173
Ectoedemia rosae|RMNH.INS.29947|Russia.Arkhangelskaya Oblast|BOLD:AAI6173
Ectoedemia LyoniaJapan|RMNH.INS.30339|Japan.Honshu|BOLD:ACU7502
Ectoedemia RubusalceifoliusTw|RMNH.INS.29272|Taiwan|BOLD:ACG9332
Ectoedemia RubuscroceacanthusTw|RMNH.INS.29329|Taiwan|BOLD:ACG8696
Ectoedemia RubuscroceacanthusTw|RMNH.INS.29480|Taiwan|BOLD:ACG8696
Ectoedemia RubusKoreaJapan|RMNH.INS.30369|Japan.Honshu|BOLD:ACU7088
Ectoedemia RubusKoreaJapan|RMNH.INS.30362|Japan.Honshu|BOLD:ACU7088
Ectoedemia RubusKoreaJapan|RMNH.INS.30173|South Korea|BOLD:ACU7088
Ectoedemia RubusKoreaJapan|RMNH.INS.30188|South Korea|BOLD:ACU7088
Ectoedemia RubusKoreaJapan|RMNH.INS.30162|South Korea|BOLD:ACU7088
Ectoedemia RubusVietnamFansipan|RMNH.INS.23892|Vietnam.Lao Cai|BOLD:AAI6175
Ectoedemia RubusVietnamFansipan|RMNH.INS.24179|Vietnam.Lao Cai|BOLD:AAI6175
Ectoedemia RubustrianthusTw|RMNH.INS.29341|Taiwan.Yilan County|BOLD:ACG9191
Ectoedemia RubusWakayama|RMNH.INS.30378|Japan.Honshu|BOLD:ACY3975
Ectoedemia RubusBorneo|RMNH.INS.23894|Indonesia.Kalimantan Timur|BOLD:AAI6177
Ectoedemia RubusBorneo|RMNH.INS.11981|Indonesia.Kalimantan Timur|BOLD:AAI6177
Ectoedemia RubusTaiwan|RMNH.INS.29454|Taiwan.Yilan County|BOLD:ACG9331
Ectoedemia RubusCucPhuong|RMNH.INS.17971|Vietnam.Ninh Binh|BOLD:ACY0956
Ectoedemia RubusVietnamTamDao|RMNH.INS.11569|Vietnam.Vinh Phuc|BOLD:ADA2457
Ectoedemia TamDao|RMNH.INS.23893|Vietnam.Vinh Phuc|BOLD:AAI6176
Ectoedemia RhododendronJapan|RMNH.INS.24723|Japan.Honshu|BOLD:ACU7392

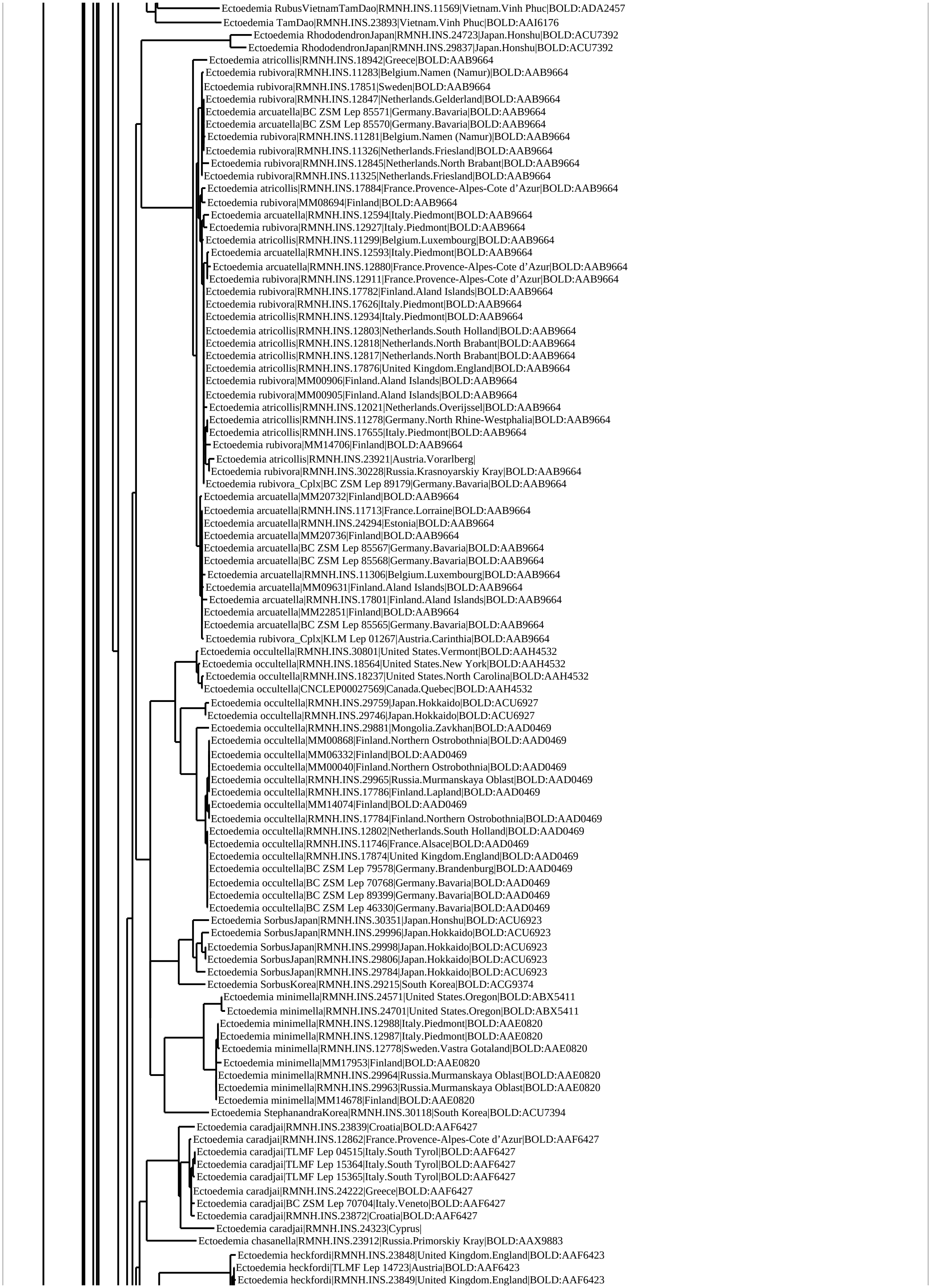
Ectoedemia RubusVietnamTamDao|RMNH.INS.11569|Vietnam.Vinh Phuc|BOLD:ADA2457
Ectoedemia TamDao|RMNH.INS.23893|Vietnam.Vinh Phuc|BOLD:AAI6176
Ectoedemia RhododendronJapan|RMNH.INS.24723|Japan.Honshu|BOLD:ACU7392
Ectoedemia RhododendronJapan|RMNH.INS.29837|Japan.Honshu|BOLD:ACU7392
Ectoedemia atricollis|RMNH.INS.18942|Greece|BOLD:AAB9664
Ectoedemia rubivora|RMNH.INS.11283|Belgium.Namen (Namur)|BOLD:AAB9664
Ectoedemia rubivora|RMNH.INS.17851|Sweden|BOLD:AAB9664
Ectoedemia rubivora|RMNH.INS.12847|Netherlands.Gelderland|BOLD:AAB9664
Ectoedemia arcuatella|BC ZSM Lep 85571|Germany.Bavaria|BOLD:AAB9664
Ectoedemia arcuatella|BC ZSM Lep 85570|Germany.Bavaria|BOLD:AAB9664
Ectoedemia rubivora|RMNH.INS.11281|Belgium.Namen (Namur)|BOLD:AAB9664
Ectoedemia rubivora|RMNH.INS.11326|Netherlands.Friesland|BOLD:AAB9664
Ectoedemia rubivora|RMNH.INS.12845|Netherlands.North Brabant|BOLD:AAB9664
Ectoedemia rubivora|RMNH.INS.11325|Netherlands.Friesland|BOLD:AAB9664
Ectoedemia atricollis|RMNH.INS.17884|France.Provence-Alpes-Cote d'Azur|BOLD:AAB9664
Ectoedemia rubivora|MM08694|Finland|BOLD:AAB9664
Ectoedemia arcuatella|RMNH.INS.12594|Italy.Piedmont|BOLD:AAB9664
Ectoedemia rubivora|RMNH.INS.12927|Italy.Piedmont|BOLD:AAB9664
Ectoedemia atricollis|RMNH.INS.11299|Belgium.Luxembourg|BOLD:AAB9664
Ectoedemia arcuatella|RMNH.INS.12593|Italy.Piedmont|BOLD:AAB9664
Ectoedemia arcuatella|RMNH.INS.12880|France.Provence-Alpes-Cote d'Azur|BOLD:AAB9664
Ectoedemia rubivora|RMNH.INS.12911|France.Provence-Alpes-Cote d'Azur|BOLD:AAB9664
Ectoedemia rubivora|RMNH.INS.17782|Finland.Aland Islands|BOLD:AAB9664
Ectoedemia rubivora|RMNH.INS.17626|Italy.Piedmont|BOLD:AAB9664
Ectoedemia atricollis|RMNH.INS.12934|Italy.Piedmont|BOLD:AAB9664
Ectoedemia atricollis|RMNH.INS.12803|Netherlands.South Holland|BOLD:AAB9664
Ectoedemia atricollis|RMNH.INS.12818|Netherlands.North Brabant|BOLD:AAB9664
Ectoedemia atricollis|RMNH.INS.12817|Netherlands.North Brabant|BOLD:AAB9664
Ectoedemia atricollis|RMNH.INS.17876|United Kingdom.England|BOLD:AAB9664
Ectoedemia rubivora|MM00906|Finland.Aland Islands|BOLD:AAB9664
Ectoedemia rubivora|MM00905|Finland.Aland Islands|BOLD:AAB9664
Ectoedemia atricollis|RMNH.INS.12021|Netherlands.Overijssel|BOLD:AAB9664
Ectoedemia atricollis|RMNH.INS.11278|Germany.North Rhine-Westphalia|BOLD:AAB9664
Ectoedemia atricollis|RMNH.INS.17655|Italy.Piedmont|BOLD:AAB9664
Ectoedemia rubivora|MM14706|Finland|BOLD:AAB9664
Ectoedemia atricollis|RMNH.INS.23921|Austria.Vorarlberg|
Ectoedemia rubivora|RMNH.INS.30228|Russia.Krasnoyarskiy Kray|BOLD:AAB9664
Ectoedemia rubivora_Cplx|BC ZSM Lep 89179|Germany.Bavaria|BOLD:AAB9664
Ectoedemia arcuatella|MM20732|Finland|BOLD:AAB9664
Ectoedemia arcuatella|RMNH.INS.11713|France.Lorraine|BOLD:AAB9664
Ectoedemia arcuatella|RMNH.INS.24294|Estonia|BOLD:AAB9664
Ectoedemia arcuatella|MM20736|Finland|BOLD:AAB9664
Ectoedemia arcuatella|BC ZSM Lep 85567|Germany.Bavaria|BOLD:AAB9664
Ectoedemia arcuatella|BC ZSM Lep 85568|Germany.Bavaria|BOLD:AAB9664
Ectoedemia arcuatella|RMNH.INS.11306|Belgium.Luxembourg|BOLD:AAB9664
Ectoedemia arcuatella|MM09631|Finland.Aland Islands|BOLD:AAB9664
Ectoedemia arcuatella|RMNH.INS.17801|Finland.Aland Islands|BOLD:AAB9664
Ectoedemia arcuatella|MM22851|Finland|BOLD:AAB9664
Ectoedemia arcuatella|BC ZSM Lep 85565|Germany.Bavaria|BOLD:AAB9664
Ectoedemia rubivora_Cplx|KLM Lep 01267|Austria.Carinthia|BOLD:AAB9664
Ectoedemia occultella|RMNH.INS.30801|United States.Vermont|BOLD:AAH4532
Ectoedemia occultella|RMNH.INS.18564|United States.New York|BOLD:AAH4532
Ectoedemia occultella|RMNH.INS.18237|United States.North Carolina|BOLD:AAH4532
Ectoedemia occultella|CNCLEP00027569|Canada.Quebec|BOLD:AAH4532
Ectoedemia occultella|RMNH.INS.29759|Japan.Hokkaido|BOLD:ACU6927
Ectoedemia occultella|RMNH.INS.29746|Japan.Hokkaido|BOLD:ACU6927
Ectoedemia occultella|RMNH.INS.29881|Mongolia.Zavkhan|BOLD:AAD0469
Ectoedemia occultella|MM00868|Finland.Northern Ostrobothnia|BOLD:AAD0469
Ectoedemia occultella|MM06332|Finland|BOLD:AAD0469
Ectoedemia occultella|MM00040|Finland.Northern Ostrobothnia|BOLD:AAD0469
Ectoedemia occultella|RMNH.INS.29965|Russia.Murmanskaya Oblast|BOLD:AAD0469
Ectoedemia occultella|RMNH.INS.17786|Finland.Lapland|BOLD:AAD0469
Ectoedemia occultella|MM14074|Finland|BOLD:AAD0469
Ectoedemia occultella|RMNH.INS.17784|Finland.Northern Ostrobothnia|BOLD:AAD0469
Ectoedemia occultella|RMNH.INS.12802|Netherlands.South Holland|BOLD:AAD0469
Ectoedemia occultella|RMNH.INS.11746|France.Alsace|BOLD:AAD0469
Ectoedemia occultella|RMNH.INS.17874|United Kingdom.England|BOLD:AAD0469
Ectoedemia occultella|BC ZSM Lep 79578|Germany.Brandenburg|BOLD:AAD0469
Ectoedemia occultella|BC ZSM Lep 70768|Germany.Bavaria|BOLD:AAD0469
Ectoedemia occultella|BC ZSM Lep 89399|Germany.Bavaria|BOLD:AAD0469
Ectoedemia occultella|BC ZSM Lep 46330|Germany.Bavaria|BOLD:AAD0469
Ectoedemia SorbusJapan|RMNH.INS.30351|Japan.Honshu|BOLD:ACU6923
Ectoedemia SorbusJapan|RMNH.INS.29996|Japan.Hokkaido|BOLD:ACU6923
Ectoedemia SorbusJapan|RMNH.INS.29998|Japan.Hokkaido|BOLD:ACU6923
Ectoedemia SorbusJapan|RMNH.INS.29806|Japan.Hokkaido|BOLD:ACU6923
Ectoedemia SorbusJapan|RMNH.INS.29784|Japan.Hokkaido|BOLD:ACU6923
Ectoedemia SorbusKorea|RMNH.INS.29215|South Korea|BOLD:ACG9374
Ectoedemia minimella|RMNH.INS.24571|United States.Oregon|BOLD:ABX5411
Ectoedemia minimella|RMNH.INS.24701|United States.Oregon|BOLD:ABX5411
Ectoedemia minimella|RMNH.INS.12988|Italy.Piedmont|BOLD:AAE0820
Ectoedemia minimella|RMNH.INS.12987|Italy.Piedmont|BOLD:AAE0820
Ectoedemia minimella|RMNH.INS.12778|Sweden.Vastra Gotaland|BOLD:AAE0820
Ectoedemia minimella|MM17953|Finland|BOLD:AAE0820
Ectoedemia minimella|RMNH.INS.29964|Russia.Murmanskaya Oblast|BOLD:AAE0820
Ectoedemia minimella|RMNH.INS.29963|Russia.Murmanskaya Oblast|BOLD:AAE0820
Ectoedemia minimella|MM14678|Finland|BOLD:AAE0820
Ectoedemia StephanandraKorea|RMNH.INS.30118|South Korea|BOLD:ACU7394
Ectoedemia caradjai|RMNH.INS.23839|Croatia|BOLD:AAF6427
Ectoedemia caradjai|RMNH.INS.12862|France.Provence-Alpes-Cote d'Azur|BOLD:AAF6427
Ectoedemia caradjai|TLMF Lep 04515|Italy.South Tyrol|BOLD:AAF6427
Ectoedemia caradjai|TLMF Lep 15364|Italy.South Tyrol|BOLD:AAF6427
Ectoedemia caradjai|TLMF Lep 15365|Italy.South Tyrol|BOLD:AAF6427
Ectoedemia caradjai|RMNH.INS.24222|Greece|BOLD:AAF6427
Ectoedemia caradjai|BC ZSM Lep 70704|Italy.Veneto|BOLD:AAF6427
Ectoedemia caradjai|RMNH.INS.23872|Croatia|BOLD:AAF6427
Ectoedemia caradjai|RMNH.INS.24323|Cyprus|
Ectoedemia chasanella|RMNH.INS.23912|Russia.Primorskiy Kray|BOLD:AAX9883
Ectoedemia heckfordi|RMNH.INS.23848|United Kingdom.England|BOLD:AAF6423
Ectoedemia heckfordi|TLMF Lep 14723|Austria|BOLD:AAF6423
Ectoedemia heckfordi|RMNH.INS.23849|United Kingdom.England|BOLD:AAF6423

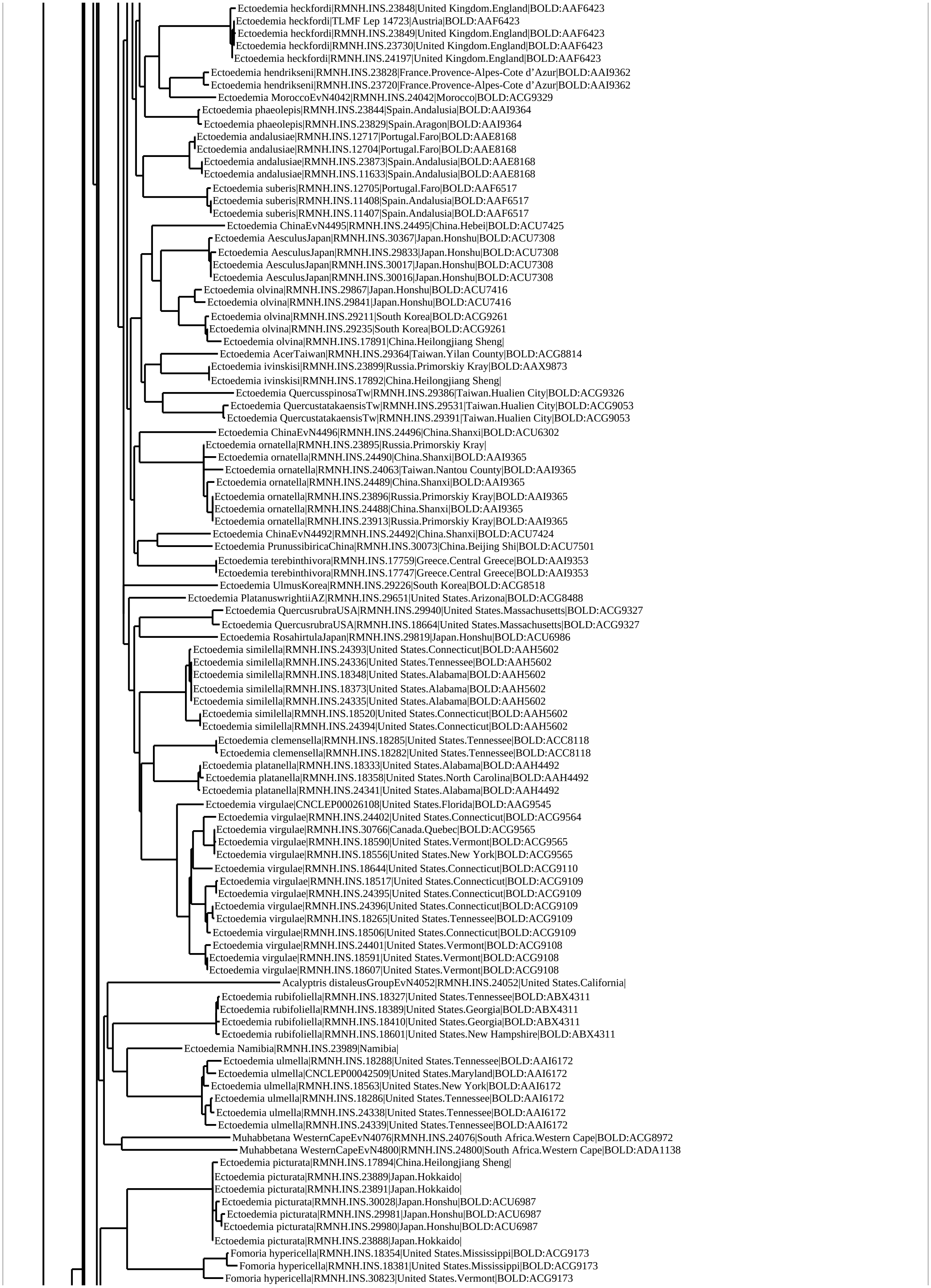
Ectoedemia heckfordi|RMNH.INS.23848|United Kingdom.England|BOLD:AAF6423
Ectoedemia heckfordi|TLMF Lep 14723|Austria|BOLD:AAF6423
Ectoedemia heckfordi|RMNH.INS.23849|United Kingdom.England|BOLD:AAF6423
Ectoedemia heckfordi|RMNH.INS.23730|United Kingdom.England|BOLD:AAF6423
Ectoedemia heckfordi|RMNH.INS.24197|United Kingdom.England|BOLD:AAF6423
Ectoedemia hendrikseni|RMNH.INS.23828|France.Provence-Alpes-Cote d'Azur|BOLD:AAI9362
Ectoedemia hendrikseni|RMNH.INS.23720|France.Provence-Alpes-Cote d'Azur|BOLD:AAI9362
Ectoedemia MoroccoEvN4042|RMNH.INS.24042|Morocco|BOLD:ACG9329
Ectoedemia phaeolepis|RMNH.INS.23844|Spain.Andalusia|BOLD:AAI9364
Ectoedemia phaeolepis|RMNH.INS.23829|Spain.Aragon|BOLD:AAI9364
Ectoedemia andalusiae|RMNH.INS.12717|Portugal.Faro|BOLD:AAE8168
Ectoedemia andalusiae|RMNH.INS.12704|Portugal.Faro|BOLD:AAE8168
Ectoedemia andalusiae|RMNH.INS.23873|Spain.Andalusia|BOLD:AAE8168
Ectoedemia andalusiae|RMNH.INS.11633|Spain.Andalusia|BOLD:AAE8168
Ectoedemia suberis|RMNH.INS.12705|Portugal.Faro|BOLD:AAF6517
Ectoedemia suberis|RMNH.INS.11408|Spain.Andalusia|BOLD:AAF6517
Ectoedemia suberis|RMNH.INS.11407|Spain.Andalusia|BOLD:AAF6517
Ectoedemia ChinaEvN4495|RMNH.INS.24495|China.Hebei|BOLD:ACU7425
Ectoedemia AesculusJapan|RMNH.INS.30367|Japan.Honshu|BOLD:ACU7308
Ectoedemia AesculusJapan|RMNH.INS.29833|Japan.Honshu|BOLD:ACU7308
Ectoedemia AesculusJapan|RMNH.INS.30017|Japan.Honshu|BOLD:ACU7308
Ectoedemia AesculusJapan|RMNH.INS.30016|Japan.Honshu|BOLD:ACU7308
Ectoedemia olvina|RMNH.INS.29867|Japan.Honshu|BOLD:ACU7416
Ectoedemia olvina|RMNH.INS.29841|Japan.Honshu|BOLD:ACU7416
Ectoedemia olvina|RMNH.INS.29211|South Korea|BOLD:ACG9261
Ectoedemia olvina|RMNH.INS.29235|South Korea|BOLD:ACG9261
Ectoedemia olvina|RMNH.INS.17891|China.Heilongjiang Sheng|
Ectoedemia AcerTaiwan|RMNH.INS.29364|Taiwan.Yilan County|BOLD:ACG8814
Ectoedemia ivinskisi|RMNH.INS.23899|Russia.Primorskiy Kray|BOLD:AAX9873
Ectoedemia ivinskisi|RMNH.INS.17892|China.Heilongjiang Sheng|
Ectoedemia QuercusspinosaTw|RMNH.INS.29386|Taiwan.Hualien City|BOLD:ACG9326
Ectoedemia QuercustatakaensisTw|RMNH.INS.29531|Taiwan.Hualien City|BOLD:ACG9053
Ectoedemia QuercustatakaensisTw|RMNH.INS.29391|Taiwan.Hualien City|BOLD:ACG9053
Ectoedemia ChinaEvN4496|RMNH.INS.24496|China.Shanxi|BOLD:ACU6302
Ectoedemia ornatella|RMNH.INS.23895|Russia.Primorskiy Kray|
Ectoedemia ornatella|RMNH.INS.24490|China.Shanxi|BOLD:AAI9365
Ectoedemia ornatella|RMNH.INS.24063|Taiwan.Nantou County|BOLD:AAI9365
Ectoedemia ornatella|RMNH.INS.24489|China.Shanxi|BOLD:AAI9365
Ectoedemia ornatella|RMNH.INS.23896|Russia.Primorskiy Kray|BOLD:AAI9365
Ectoedemia ornatella|RMNH.INS.24488|China.Shanxi|BOLD:AAI9365
Ectoedemia ornatella|RMNH.INS.23913|Russia.Primorskiy Kray|BOLD:AAI9365
Ectoedemia ChinaEvN4492|RMNH.INS.24492|China.Shanxi|BOLD:ACU7424
Ectoedemia PrunussibiricaChina|RMNH.INS.30073|China.Beijing Shi|BOLD:ACU7501
Ectoedemia terebinthivora|RMNH.INS.17759|Greece.Central Greece|BOLD:AAI9353
Ectoedemia terebinthivora|RMNH.INS.17747|Greece.Central Greece|BOLD:AAI9353
Ectoedemia UlmusKorea|RMNH.INS.29226|South Korea|BOLD:ACG8518
Ectoedemia PlatanuswrightiiAZ|RMNH.INS.29651|United States.Arizona|BOLD:ACG8488
Ectoedemia QuercusrubraUSA|RMNH.INS.29940|United States.Massachusetts|BOLD:ACG9327
Ectoedemia QuercusrubraUSA|RMNH.INS.18664|United States.Massachusetts|BOLD:ACG9327
Ectoedemia RosahirtulaJapan|RMNH.INS.29819|Japan.Honshu|BOLD:ACU6986
Ectoedemia similella|RMNH.INS.24393|United States.Connecticut|BOLD:AAH5602
Ectoedemia similella|RMNH.INS.24336|United States.Tennessee|BOLD:AAH5602
Ectoedemia similella|RMNH.INS.18348|United States.Alabama|BOLD:AAH5602
Ectoedemia similella|RMNH.INS.18373|United States.Alabama|BOLD:AAH5602
Ectoedemia similella|RMNH.INS.24335|United States.Alabama|BOLD:AAH5602
Ectoedemia similella|RMNH.INS.18520|United States.Connecticut|BOLD:AAH5602
Ectoedemia similella|RMNH.INS.24394|United States.Connecticut|BOLD:AAH5602
Ectoedemia clemensella|RMNH.INS.18285|United States.Tennessee|BOLD:ACC8118
Ectoedemia clemensella|RMNH.INS.18282|United States.Tennessee|BOLD:ACC8118
Ectoedemia platanella|RMNH.INS.18333|United States.Alabama|BOLD:AAH4492
Ectoedemia platanella|RMNH.INS.18358|United States.North Carolina|BOLD:AAH4492
Ectoedemia platanella|RMNH.INS.24341|United States.Alabama|BOLD:AAH4492
Ectoedemia virgulae|CNCLEP00026108|United States.Florida|BOLD:AAG9545
Ectoedemia virgulae|RMNH.INS.24402|United States.Connecticut|BOLD:ACG9564
Ectoedemia virgulae|RMNH.INS.30766|Canada.Quebec|BOLD:ACG9565
Ectoedemia virgulae|RMNH.INS.18590|United States.Vermont|BOLD:ACG9565
Ectoedemia virgulae|RMNH.INS.18556|United States.New York|BOLD:ACG9565
Ectoedemia virgulae|RMNH.INS.18644|United States.Connecticut|BOLD:ACG9110
Ectoedemia virgulae|RMNH.INS.18517|United States.Connecticut|BOLD:ACG9109
Ectoedemia virgulae|RMNH.INS.24395|United States.Connecticut|BOLD:ACG9109
Ectoedemia virgulae|RMNH.INS.24396|United States.Connecticut|BOLD:ACG9109
Ectoedemia virgulae|RMNH.INS.18265|United States.Tennessee|BOLD:ACG9109
Ectoedemia virgulae|RMNH.INS.18506|United States.Connecticut|BOLD:ACG9109
Ectoedemia virgulae|RMNH.INS.24401|United States.Vermont|BOLD:ACG9108
Ectoedemia virgulae|RMNH.INS.18591|United States.Vermont|BOLD:ACG9108
Ectoedemia virgulae|RMNH.INS.18607|United States.Vermont|BOLD:ACG9108
Acalyptris distaleusGroupEvN4052|RMNH.INS.24052|United States.California|
Ectoedemia rubifoliella|RMNH.INS.18327|United States.Tennessee|BOLD:ABX4311
Ectoedemia rubifoliella|RMNH.INS.18389|United States.Georgia|BOLD:ABX4311
Ectoedemia rubifoliella|RMNH.INS.18410|United States.Georgia|BOLD:ABX4311
Ectoedemia rubifoliella|RMNH.INS.18601|United States.New Hampshire|BOLD:ABX4311
Ectoedemia Namibia|RMNH.INS.23989|Namibia|
Ectoedemia ulmella|RMNH.INS.18288|United States.Tennessee|BOLD:AAI6172
Ectoedemia ulmella|CNCLEP00042509|United States.Maryland|BOLD:AAI6172
Ectoedemia ulmella|RMNH.INS.18563|United States.New York|BOLD:AAI6172
Ectoedemia ulmella|RMNH.INS.18286|United States.Tennessee|BOLD:AAI6172
Ectoedemia ulmella|RMNH.INS.24338|United States.Tennessee|BOLD:AAI6172
Ectoedemia ulmella|RMNH.INS.24339|United States.Tennessee|BOLD:AAI6172
Muhabbetana WesternCapeEvN4076|RMNH.INS.24076|South Africa.Western Cape|BOLD:ACG8972
Muhabbetana WesternCapeEvN4800|RMNH.INS.24800|South Africa.Western Cape|BOLD:ADA1138
Ectoedemia picturata|RMNH.INS.17894|China.Heilongjiang Sheng|
Ectoedemia picturata|RMNH.INS.23889|Japan.Hokkaido|
Ectoedemia picturata|RMNH.INS.23891|Japan.Hokkaido|
Ectoedemia picturata|RMNH.INS.30028|Japan.Honshu|BOLD:ACU6987
Ectoedemia picturata|RMNH.INS.29981|Japan.Honshu|BOLD:ACU6987
Ectoedemia picturata|RMNH.INS.29980|Japan.Honshu|BOLD:ACU6987
Ectoedemia picturata|RMNH.INS.23888|Japan.Hokkaido|
Fomoria hypericella|RMNH.INS.18354|United States.Mississippi|BOLD:ACG9173
Fomoria hypericella|RMNH.INS.18381|United States.Mississippi|BOLD:ACG9173
Fomoria hypericella|RMNH.INS.30823|United States.Vermont|BOLD:ACG9173

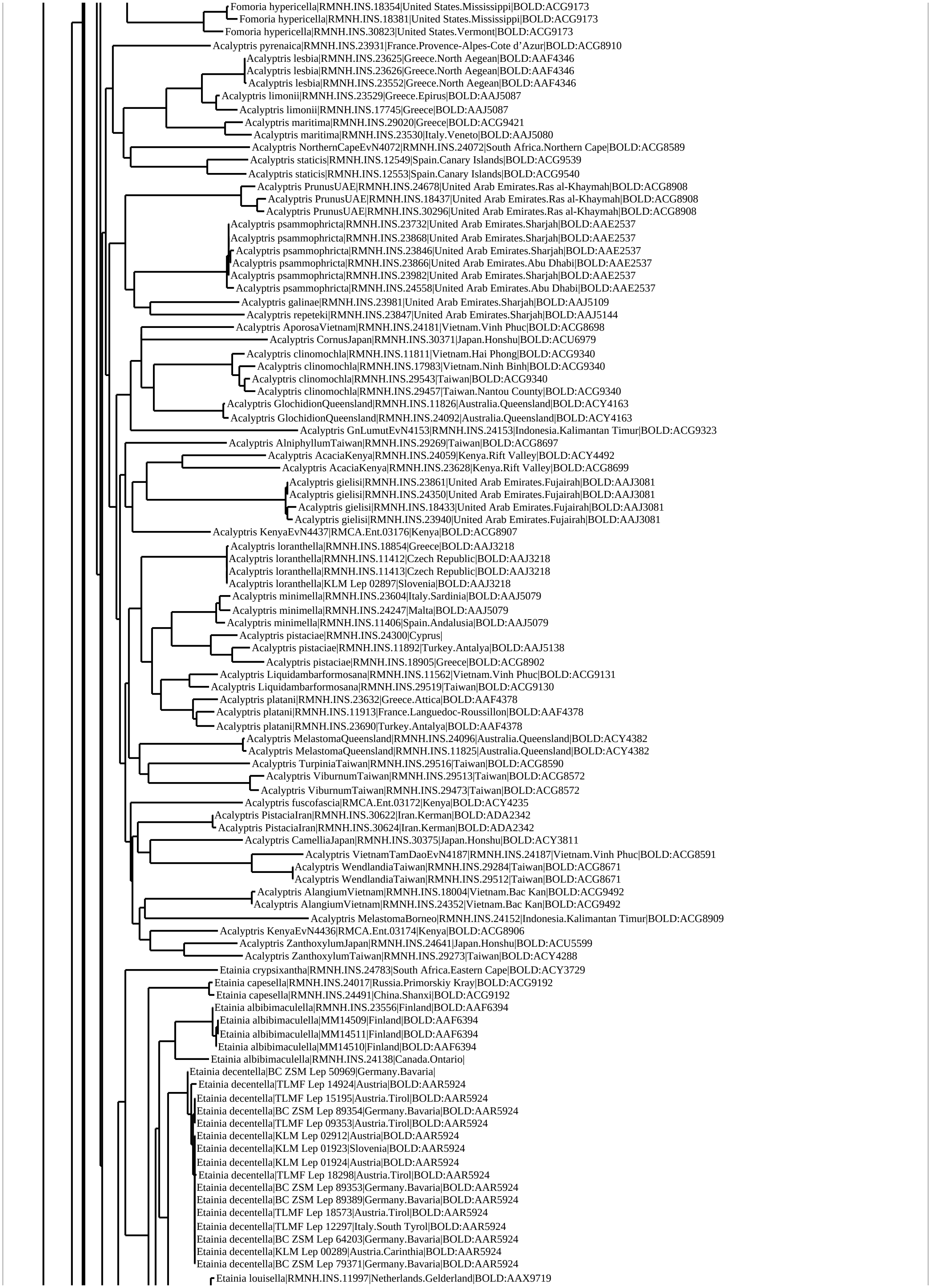
Fomoria hypericella|RMNH.INS.18354|United States.Mississippi|BOLD:ACG9173
Fomoria hypericella|RMNH.INS.18381|United States.Mississippi|BOLD:ACG9173
Fomoria hypericella|RMNH.INS.30823|United States.Vermont|BOLD:ACG9173
Acalyptris pyrenaica|RMNH.INS.23931|France.Provence-Alpes-Cote d'Azur|BOLD:ACG8910
Acalyptris lesbia|RMNH.INS.23625|Greece.North Aegean|BOLD:AAF4346
Acalyptris lesbia|RMNH.INS.23626|Greece.North Aegean|BOLD:AAF4346
Acalyptris lesbia|RMNH.INS.23552|Greece.North Aegean|BOLD:AAF4346
Acalyptris limonii|RMNH.INS.23529|Greece.Epirus|BOLD:AAJ5087
Acalyptris limonii|RMNH.INS.17745|Greece|BOLD:AAJ5087
Acalyptris maritima|RMNH.INS.29020|Greece|BOLD:ACG9421
Acalyptris maritima|RMNH.INS.23530|Italy.Veneto|BOLD:AAJ5080
Acalyptris NorthernCapeEvN4072|RMNH.INS.24072|South Africa.Northern Cape|BOLD:ACG8589
Acalyptris staticis|RMNH.INS.12549|Spain.Canary Islands|BOLD:ACG9539
Acalyptris staticis|RMNH.INS.12553|Spain.Canary Islands|BOLD:ACG9540
Acalyptris PrunusUAE|RMNH.INS.24678|United Arab Emirates.Ras al-Khaymah|BOLD:ACG8908
Acalyptris PrunusUAE|RMNH.INS.18437|United Arab Emirates.Ras al-Khaymah|BOLD:ACG8908
Acalyptris PrunusUAE|RMNH.INS.30296|United Arab Emirates.Ras al-Khaymah|BOLD:ACG8908
Acalyptris psammophricta|RMNH.INS.23732|United Arab Emirates.Sharjah|BOLD:AAE2537
Acalyptris psammophricta|RMNH.INS.23868|United Arab Emirates.Sharjah|BOLD:AAE2537
Acalyptris psammophricta|RMNH.INS.23846|United Arab Emirates.Sharjah|BOLD:AAE2537
Acalyptris psammophricta|RMNH.INS.23866|United Arab Emirates.Abu Dhabi|BOLD:AAE2537
Acalyptris psammophricta|RMNH.INS.23982|United Arab Emirates.Sharjah|BOLD:AAE2537
Acalyptris psammophricta|RMNH.INS.24558|United Arab Emirates.Abu Dhabi|BOLD:AAE2537
Acalyptris galinae|RMNH.INS.23981|United Arab Emirates.Sharjah|BOLD:AAJ5109
Acalyptris repeteki|RMNH.INS.23847|United Arab Emirates.Sharjah|BOLD:AAJ5144
Acalyptris AporosaVietnam|RMNH.INS.24181|Vietnam.Vinh Phuc|BOLD:ACG8698
Acalyptris CornusJapan|RMNH.INS.30371|Japan.Honshu|BOLD:ACU6979
Acalyptris clinomochla|RMNH.INS.11811|Vietnam.Hai Phong|BOLD:ACG9340
Acalyptris clinomochla|RMNH.INS.17983|Vietnam.Ninh Binh|BOLD:ACG9340
Acalyptris clinomochla|RMNH.INS.29543|Taiwan|BOLD:ACG9340
Acalyptris clinomochla|RMNH.INS.29457|Taiwan.Nantou County|BOLD:ACG9340
Acalyptris GlochidionQueensland|RMNH.INS.11826|Australia.Queensland|BOLD:ACY4163
Acalyptris GlochidionQueensland|RMNH.INS.24092|Australia.Queensland|BOLD:ACY4163
Acalyptris GnLumutEvN4153|RMNH.INS.24153|Indonesia.Kalimantan Timur|BOLD:ACG9323
Acalyptris AlniphyllumTaiwan|RMNH.INS.29269|Taiwan|BOLD:ACG8697
Acalyptris AcaciaKenya|RMNH.INS.24059|Kenya.Rift Valley|BOLD:ACY4492
Acalyptris AcaciaKenya|RMNH.INS.23628|Kenya.Rift Valley|BOLD:ACG8699
Acalyptris gielisi|RMNH.INS.23861|United Arab Emirates.Fujairah|BOLD:AAJ3081
Acalyptris gielisi|RMNH.INS.24350|United Arab Emirates.Fujairah|BOLD:AAJ3081
Acalyptris gielisi|RMNH.INS.18433|United Arab Emirates.Fujairah|BOLD:AAJ3081
Acalyptris gielisi|RMNH.INS.23940|United Arab Emirates.Fujairah|BOLD:AAJ3081
Acalyptris KenyaEvN4437|RMCA.Ent.03176|Kenya|BOLD:ACG8907
Acalyptris loranthella|RMNH.INS.18854|Greece|BOLD:AAJ3218
Acalyptris loranthella|RMNH.INS.11412|Czech Republic|BOLD:AAJ3218
Acalyptris loranthella|RMNH.INS.11413|Czech Republic|BOLD:AAJ3218
Acalyptris loranthella|KLM Lep 02897|Slovenia|BOLD:AAJ3218
Acalyptris minimella|RMNH.INS.23604|Italy.Sardinia|BOLD:AAJ5079
Acalyptris minimella|RMNH.INS.24247|Malta|BOLD:AAJ5079
Acalyptris minimella|RMNH.INS.11406|Spain.Andalusia|BOLD:AAJ5079
Acalyptris pistaciae|RMNH.INS.24300|Cyprus|
Acalyptris pistaciae|RMNH.INS.11892|Turkey.Antalya|BOLD:AAJ5138
Acalyptris pistaciae|RMNH.INS.18905|Greece|BOLD:ACG8902
Acalyptris Liquidambarformosana|RMNH.INS.11562|Vietnam.Vinh Phuc|BOLD:ACG9131
Acalyptris Liquidambarformosana|RMNH.INS.29519|Taiwan|BOLD:ACG9130
Acalyptris platani|RMNH.INS.23632|Greece.Attica|BOLD:AAF4378
Acalyptris platani|RMNH.INS.11913|France.Languedoc-Roussillon|BOLD:AAF4378
Acalyptris platani|RMNH.INS.23690|Turkey.Antalya|BOLD:AAF4378
Acalyptris MelastomaQueensland|RMNH.INS.24096|Australia.Queensland|BOLD:ACY4382
Acalyptris MelastomaQueensland|RMNH.INS.11825|Australia.Queensland|BOLD:ACY4382
Acalyptris TurpiniaTaiwan|RMNH.INS.29516|Taiwan|BOLD:ACG8590
Acalyptris ViburnumTaiwan|RMNH.INS.29513|Taiwan|BOLD:ACG8572
Acalyptris ViburnumTaiwan|RMNH.INS.29473|Taiwan|BOLD:ACG8572
Acalyptris fuscofascia|RMCA.Ent.03172|Kenya|BOLD:ACY4235
Acalyptris PistaciaIran|RMNH.INS.30622|Iran.Kerman|BOLD:ADA2342
Acalyptris PistaciaIran|RMNH.INS.30624|Iran.Kerman|BOLD:ADA2342
Acalyptris CamelliaJapan|RMNH.INS.30375|Japan.Honshu|BOLD:ACY3811
Acalyptris VietnamTamDaoEvN4187|RMNH.INS.24187|Vietnam.Vinh Phuc|BOLD:ACG8591
Acalyptris WendlandiaTaiwan|RMNH.INS.29284|Taiwan|BOLD:ACG8671
Acalyptris WendlandiaTaiwan|RMNH.INS.29512|Taiwan|BOLD:ACG8671
Acalyptris AlangiumVietnam|RMNH.INS.18004|Vietnam.Bac Kan|BOLD:ACG9492
Acalyptris AlangiumVietnam|RMNH.INS.24352|Vietnam.Bac Kan|BOLD:ACG9492
Acalyptris MelastomaBorneo|RMNH.INS.24152|Indonesia.Kalimantan Timur|BOLD:ACG8909
Acalyptris KenyaEvN4436|RMCA.Ent.03174|Kenya|BOLD:ACG8906
Acalyptris ZanthoxylumJapan|RMNH.INS.24641|Japan.Honshu|BOLD:ACU5599
Acalyptris ZanthoxylumTaiwan|RMNH.INS.29273|Taiwan|BOLD:ACY4288
Etainia crypsixantha|RMNH.INS.24783|South Africa.Eastern Cape|BOLD:ACY3729
Etainia capesella|RMNH.INS.24017|Russia.Primorskiy Kray|BOLD:ACG9192
Etainia capesella|RMNH.INS.24491|China.Shanxi|BOLD:ACG9192
Etainia albibimaculella|RMNH.INS.23556|Finland|BOLD:AAF6394
Etainia albibimaculella|MM14509|Finland|BOLD:AAF6394
Etainia albibimaculella|MM14511|Finland|BOLD:AAF6394
Etainia albibimaculella|MM14510|Finland|BOLD:AAF6394
Etainia albibimaculella|RMNH.INS.24138|Canada.Ontario|
Etainia decentella|BC ZSM Lep 50969|Germany.Bavaria|
Etainia decentella|TLMF Lep 14924|Austria|BOLD:AAR5924
Etainia decentella|TLMF Lep 15195|Austria.Tirol|BOLD:AAR5924
Etainia decentella|BC ZSM Lep 89354|Germany.Bavaria|BOLD:AAR5924
Etainia decentella|TLMF Lep 09353|Austria.Tirol|BOLD:AAR5924
Etainia decentella|KLM Lep 02912|Austria|BOLD:AAR5924
Etainia decentella|KLM Lep 01923|Slovenia|BOLD:AAR5924
Etainia decentella|KLM Lep 01924|Austria|BOLD:AAR5924
Etainia decentella|TLMF Lep 18298|Austria.Tirol|BOLD:AAR5924
Etainia decentella|BC ZSM Lep 89353|Germany.Bavaria|BOLD:AAR5924
Etainia decentella|BC ZSM Lep 89389|Germany.Bavaria|BOLD:AAR5924
Etainia decentella|TLMF Lep 18573|Austria.Tirol|BOLD:AAR5924
Etainia decentella|TLMF Lep 12297|Italy.South Tyrol|BOLD:AAR5924
Etainia decentella|BC ZSM Lep 64203|Germany.Bavaria|BOLD:AAR5924
Etainia decentella|KLM Lep 00289|Austria.Carinthia|BOLD:AAR5924
Etainia decentella|BC ZSM Lep 79371|Germany.Bavaria|BOLD:AAR5924
Etainia louisella|RMNH.INS.11997|Netherlands.Gelderland|BOLD:AAX9719

Etainia decentella|KLM Lep 00289|Austria.Carinthia|BOLD:AAR5924
Etainia decentella|BC ZSM Lep 79371|Germany.Bavaria|BOLD:AAR5924
Etainia louisella|RMNH.INS.11997|Netherlands.Gelderland|BOLD:AAX9719
Etainia louisella|TLMF Lep 18463|Italy.South Tyrol|BOLD:AAX9719
Etainia louisella|TLMF Lep 18464|Italy.South Tyrol|BOLD:AAX9719
Etainia louisella|RMNH.INS.12604|Italy.Piedmont|BOLD:AAX9719
Etainia louisella|KLM Lep 01262|Austria.Carinthia|BOLD:AAX9719
Etainia GeorgiaEvN4807|RMNH.INS.24807|Georgia|BOLD:ADA2857
Etainia sericopeza|BC ZSM Lep 64195|Germany.Bavaria|BOLD:AAC4337
Etainia sericopeza|RMNH.INS.11596|Netherlands.South Holland|BOLD:AAC4337
Etainia sericopeza|BC ZSM Lep 70718|Germany.Berlin|BOLD:AAC4337
Etainia sericopeza|KLM Lep 02878|Slovenia|BOLD:AAC4337
Etainia sericopeza|TLMF Lep 13740|Austria.Tirol|BOLD:AAC4337
Etainia sericopeza|BC ZSM Lep 63897|Germany.Bavaria|BOLD:AAC4337
Etainia sericopeza|BC ZSM Lep 70731|Germany.Bavaria|BOLD:AAC4337
Etainia sericopeza|BC ZSM Lep 70732|Germany.Bavaria|BOLD:AAC4337
Etainia sericopeza|AC005156|Canada.Quebec|BOLD:AAC4337
Etainia sericopeza|TLMF Lep 14798|Austria|BOLD:AAC4337
Etainia sericopeza|MM17329|Finland|BOLD:AAC4337
Etainia sericopeza|MM21131|Finland|BOLD:AAC4337
Etainia sericopeza|MM14880|Finland|BOLD:AAC4337
Etainia sericopeza|MM21037|Finland|BOLD:AAC4337
Etainia sericopeza|TLMF Lep 18574|Austria.Tirol|BOLD:AAC4337
Etainia sericopeza|BC ZSM Lep 50968|Germany.Bavaria|BOLD:AAC4337
Etainia sericopeza|BC ZSM Lep 28754|Germany.Bavaria|BOLD:AAC4337
Etainia sericopeza|BC ZSM Lep 89146|Germany.Bavaria|BOLD:AAC4337
Etainia sericopeza|BC ZSM Lep 70691|Germany.Bavaria|BOLD:AAC4337
Etainia sericopeza|BC ZSM Lep 89383|Germany.Bavaria|BOLD:AAC4337
Etainia sericopeza|TLMF Lep 07484|Austria.Vorarlberg|BOLD:AAC4337
Etainia sericopeza|CNCLEP00027797|Canada.Ontario|BOLD:AAC4337
Etainia sericopeza|CNCLEP00077873|Canada.British Columbia|BOLD:AAC4337
Etainia sericopeza|CNCLEP00027800|Canada.Ontario|BOLD:AAC4337
Etainia sericopeza|CNCLEP00027804|Canada.Ontario|BOLD:AAC4337
Etainia sericopeza|CNCLEP00077871|Canada.British Columbia|BOLD:AAC4337
Etainia sericopeza|CNCLEP00027803|Canada.Ontario|BOLD:AAC4337
Etainia sericopeza|CNCLEP00027801|Canada.Ontario|BOLD:AAC4337
Etainia sericopeza|CNCLEP00027798|Canada.Ontario|BOLD:AAC4337
Etainia sericopeza|CNCLEP00027796|Canada.Ontario|BOLD:AAC4337
Etainia sericopeza|CNCLEP00027799|Canada.Ontario|BOLD:AAC4337
Etainia sericopeza|CNCLEP00027795|Canada.Ontario|BOLD:AAC4337
Etainia sericopeza|CNCLEP00077872|Canada.British Columbia|BOLD:AAC4337
Etainia sericopeza|CNCLEP00027802|Canada.Ontario|BOLD:AAC4337
Etainia sericopeza|RMNH.INS.18170|United States.Oregon|BOLD:AAC4337
Etainia sericopeza|RMNH.INS.18174|United States.Oregon|BOLD:AAC4337
Etainia ochrefasciella|RMNH.INS.24137|United States.Indiana|BOLD:ABW0989
Etainia ochrefasciella|RMNH.INS.24136|United States.Indiana|BOLD:ABW0989
Etainia peterseni|RMNH.INS.24487|China.Liaoning|BOLD:ACU6250
Etainia Taiwan|RMNH.INS.24328|Taiwan.Hualien County|BOLD:ACG8519
Etainia Taiwan|RMNH.INS.24329|Taiwan.Hualien City|BOLD:ACG8519
Etainia Kenya|RMCA.Ent.03170|Kenya|BOLD:ACG8973
Etainia zimbabwiensis|RMCA.Ent.03168|Kenya|BOLD:ACG8664
Etainia zimbabwiensis|RMCA.Ent.03167|Kenya|BOLD:ACG8664
Fomoria CallicarpaJapan|RMNH.INS.30010|Japan.Honshu|BOLD:ACU6039
Fomoria CallicarpaJapan|RMNH.INS.24644|Japan.Honshu|BOLD:ACU6039
Fomoria CallicarpaJapan|RMNH.INS.30332|Japan.Honshu|BOLD:ACU6040
Fomoria CallicarpaTaiwan|RMNH.INS.29335|Taiwan|BOLD:ACG9014
Fomoria CallicarpaTaiwan|RMNH.INS.29467|Taiwan|BOLD:ACG9435
Fomoria CallicarpaVietnam|RMNH.INS.24180|Vietnam.Vinh Phuc|BOLD:ACG8507
Fomoria CallicarpaVietnam|RMNH.INS.11570|Vietnam.Vinh Phuc|BOLD:ACG8507
Fomoria RadermacheraTaiwan|RMNH.INS.29520|Taiwan|BOLD:ACG9069
Fomoria viridissimella|RMNH.INS.23593|Poland.Lublin|BOLD:ACG8667
Fomoria viridissimella|RMNH.INS.24256|Poland.Lublin|BOLD:ACG8667
Fomoria permira|RMNH.INS.24645|Japan.Honshu|BOLD:ACU5611
Fomoria permira|RMNH.INS.24722|Japan.Honshu|BOLD:ACU5611
Fomoria TetradiumTaiwan|RMNH.INS.29275|Taiwan|BOLD:ACG8975
Glaucolepis argentosa|RMNH.INS.23748|Belize.Cayo|
Muhabbetana EvN3867|RMNH.INS.24253|Yemen|BOLD:AAJ5145
Muhabbetana EvN3867|RMNH.INS.23867|United Arab Emirates.Abu Dhabi|BOLD:AAJ5145
Muhabbetana KenyaEvN4435|RMCA.Ent.03173|Kenya|BOLD:ACY4528
Muhabbetana nigrifasciata|RMNH.INS.30656|Spain.Canary Islands|BOLD:ADA1569
Muhabbetana nigrifasciata|RMNH.INS.11419|Spain.Canary Islands|BOLD:ACG9125
Muhabbetana nigrifasciata|RMNH.INS.11789|Spain.Canary Islands|BOLD:ACG9124
Fomoria ScolopiaQueensland|RMNH.INS.24097|Australia.Queensland|BOLD:ACG8974
Fomoria ScolopiaQueensland|RMNH.INS.11840|Australia.Queensland|BOLD:ACG8974
Stigmella Surinam|RMNH.INS.30587|Suriname.Sipaliwini|BOLD:ACY4001
Fomoria NamibiaEvN4801|RMNH.INS.24801|Namibia|BOLD:ADA2987
Fomoria pteliaella|RMNH.INS.24403|United States.Connecticut|BOLD:ACG9567
Fomoria pteliaella|RMNH.INS.18580|United States.Vermont|BOLD:ACG9567
Fomoria groschkei|RMNH.INS.23691|Turkey.Antalya|BOLD:ACG8929
Fomoria groschkei|RMNH.INS.18843|Greece|BOLD:ACG8929
Fomoria groschkei|RMNH.INS.18985|Greece|BOLD:ACG8929
Fomoria groschkei|RMNH.INS.29047|Greece|BOLD:ACG8929
Fomoria groschkei|RMNH.INS.17757|Greece|BOLD:ACG8929
Fomoria VitexChina|RMNH.INS.30071|China.Beijing Shi|BOLD:ACU5698
Fomoria VitexTaiwan|RMNH.INS.29290|Taiwan|BOLD:ACG9341
Fomoria VitexTaiwan|RMNH.INS.29289|Taiwan|BOLD:ACG9341
Acalyptris ArizonaEvN4682|RMNH.INS.24682|United States.Arizona|BOLD:ACU6204
Acalyptris janzeni|RMNH.INS.23673|Costa Rica.Guanacaste|BOLD:ACG4756
Acalyptris AlbertaEvN4792|RMNH.INS.24792|Canada.Alberta|BOLD:ACY3818
Acalyptris thoracealbella|RMNH.INS.24781|United States.New York|BOLD:ACY4164
Acalyptris bipinnatellus|RMNH.INS.23817|United States.Florida|BOLD:ACG8700
Acalyptris EriophorumCanada|RMNH.INS.30748|Canada.Ontario|BOLD:ACT3149
Acalyptris LicaniaUSA|RMNH.INS.18346|United States.Alabama|BOLD:ACG8770
Acalyptris LicaniaUSA|RMNH.INS.24344|United States.Alabama|BOLD:ACG8770
Acalyptris tenuijuxtus|RMNH.INS.24343|United States.Mississippi|BOLD:ACG8588
Acalyptris tenuijuxtus|RMNH.INS.23818|United States.Florida|BOLD:ACG8812
Acalyptris tenuijuxtus|RMNH.INS.12533|United States.Florida|BOLD:ACG8812
Fomoria EasternCapeEvN4784|RMNH.INS.24784|South Africa.Eastern Cape|BOLD:ACY4527
Zimmermannia liebwerdella|RMNH.INS.24608|Italy.Calabria|BOLD:ABW6511
Zimmermannia liebwerdella|RMNH.INS.24609|Italy.Calabria|BOLD:ABW6511
[illegible]

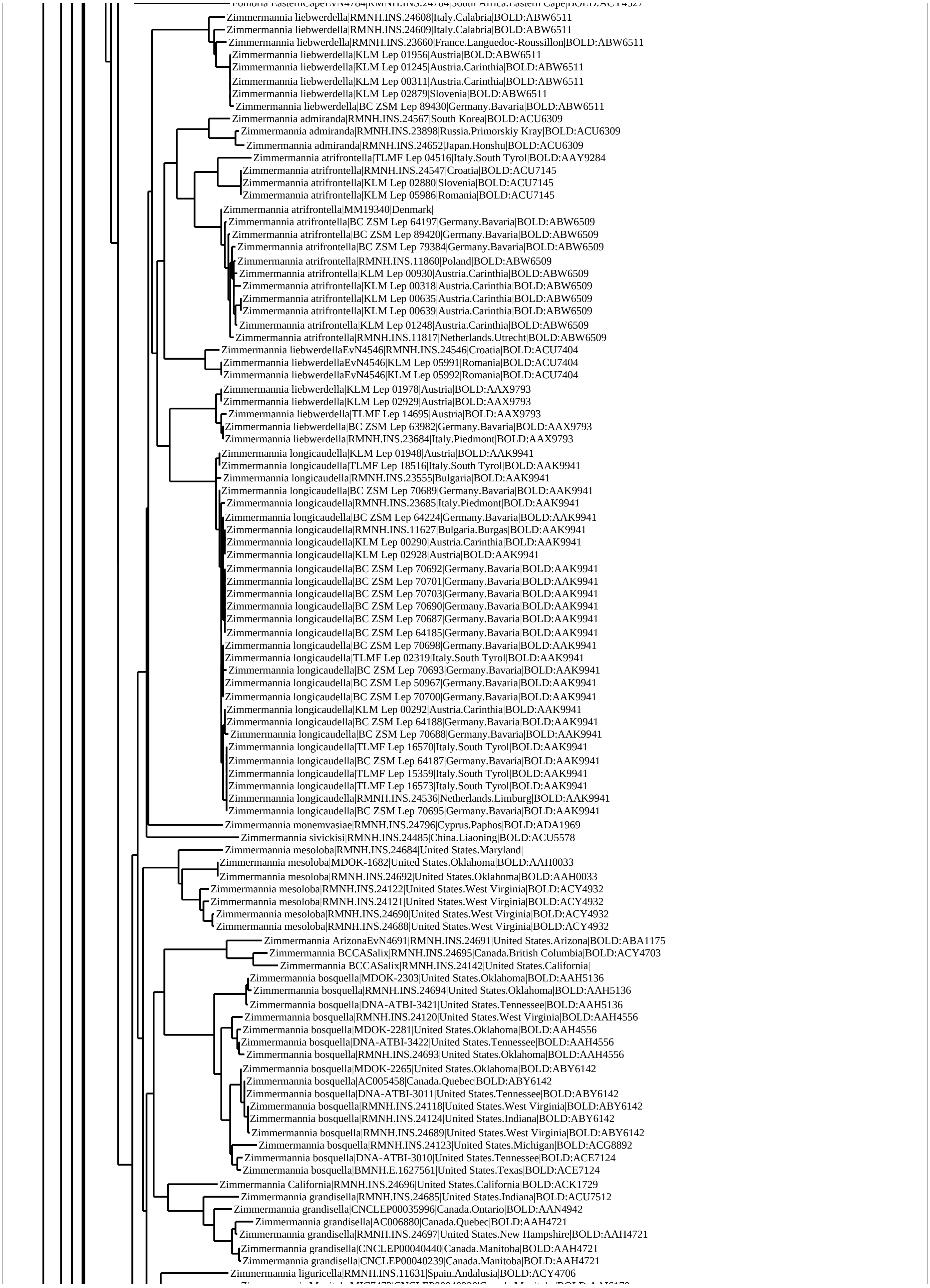
Zimmermannia liebwerdella|RMNH.INS.24608|Italy.Calabria|BOLD:ABW6511
Zimmermannia liebwerdella|RMNH.INS.24609|Italy.Calabria|BOLD:ABW6511
Zimmermannia liebwerdella|RMNH.INS.23660|France.Languedoc-Roussillon|BOLD:ABW6511
Zimmermannia liebwerdella|KLM Lep 01956|Austria|BOLD:ABW6511
Zimmermannia liebwerdella|KLM Lep 01245|Austria.Carinthia|BOLD:ABW6511
Zimmermannia liebwerdella|KLM Lep 00311|Austria.Carinthia|BOLD:ABW6511
Zimmermannia liebwerdella|KLM Lep 02879|Slovenia|BOLD:ABW6511
Zimmermannia liebwerdella|BC ZSM Lep 89430|Germany.Bavaria|BOLD:ABW6511
Zimmermannia admiranda|RMNH.INS.24567|South Korea|BOLD:ACU6309
Zimmermannia admiranda|RMNH.INS.23898|Russia.Primorskiy Kray|BOLD:ACU6309
Zimmermannia admiranda|RMNH.INS.24652|Japan.Honshu|BOLD:ACU6309
Zimmermannia atrifrontella|TLMF Lep 04516|Italy.South Tyrol|BOLD:AAY9284
Zimmermannia atrifrontella|RMNH.INS.24547|Croatia|BOLD:ACU7145
Zimmermannia atrifrontella|KLM Lep 02880|Slovenia|BOLD:ACU7145
Zimmermannia atrifrontella|KLM Lep 05986|Romania|BOLD:ACU7145
Zimmermannia atrifrontella|MM19340|Denmark|
Zimmermannia atrifrontella|BC ZSM Lep 64197|Germany.Bavaria|BOLD:ABW6509
Zimmermannia atrifrontella|BC ZSM Lep 89420|Germany.Bavaria|BOLD:ABW6509
Zimmermannia atrifrontella|BC ZSM Lep 79384|Germany.Bavaria|BOLD:ABW6509
Zimmermannia atrifrontella|RMNH.INS.11860|Poland|BOLD:ABW6509
Zimmermannia atrifrontella|KLM Lep 00930|Austria.Carinthia|BOLD:ABW6509
Zimmermannia atrifrontella|KLM Lep 00318|Austria.Carinthia|BOLD:ABW6509
Zimmermannia atrifrontella|KLM Lep 00635|Austria.Carinthia|BOLD:ABW6509
Zimmermannia atrifrontella|KLM Lep 00639|Austria.Carinthia|BOLD:ABW6509
Zimmermannia atrifrontella|KLM Lep 01248|Austria.Carinthia|BOLD:ABW6509
Zimmermannia atrifrontella|RMNH.INS.11817|Netherlands.Utrecht|BOLD:ABW6509
Zimmermannia liebwerdellaEvN4546|RMNH.INS.24546|Croatia|BOLD:ACU7404
Zimmermannia liebwerdellaEvN4546|KLM Lep 05991|Romania|BOLD:ACU7404
Zimmermannia liebwerdellaEvN4546|KLM Lep 05992|Romania|BOLD:ACU7404
Zimmermannia liebwerdella|KLM Lep 01978|Austria|BOLD:AAX9793
Zimmermannia liebwerdella|KLM Lep 02929|Austria|BOLD:AAX9793
Zimmermannia liebwerdella|TLMF Lep 14695|Austria|BOLD:AAX9793
Zimmermannia liebwerdella|BC ZSM Lep 63982|Germany.Bavaria|BOLD:AAX9793
Zimmermannia liebwerdella|RMNH.INS.23684|Italy.Piedmont|BOLD:AAX9793
Zimmermannia longicaudella|KLM Lep 01948|Austria|BOLD:AAK9941
Zimmermannia longicaudella|TLMF Lep 18516|Italy.South Tyrol|BOLD:AAK9941
Zimmermannia longicaudella|RMNH.INS.23555|Bulgaria|BOLD:AAK9941
Zimmermannia longicaudella|BC ZSM Lep 70689|Germany.Bavaria|BOLD:AAK9941
Zimmermannia longicaudella|RMNH.INS.23685|Italy.Piedmont|BOLD:AAK9941
Zimmermannia longicaudella|BC ZSM Lep 64224|Germany.Bavaria|BOLD:AAK9941
Zimmermannia longicaudella|RMNH.INS.11627|Bulgaria.Burgas|BOLD:AAK9941
Zimmermannia longicaudella|KLM Lep 00290|Austria.Carinthia|BOLD:AAK9941
Zimmermannia longicaudella|KLM Lep 02928|Austria|BOLD:AAK9941
Zimmermannia longicaudella|BC ZSM Lep 70692|Germany.Bavaria|BOLD:AAK9941
Zimmermannia longicaudella|BC ZSM Lep 70701|Germany.Bavaria|BOLD:AAK9941
Zimmermannia longicaudella|BC ZSM Lep 70703|Germany.Bavaria|BOLD:AAK9941
Zimmermannia longicaudella|BC ZSM Lep 70690|Germany.Bavaria|BOLD:AAK9941
Zimmermannia longicaudella|BC ZSM Lep 70687|Germany.Bavaria|BOLD:AAK9941
Zimmermannia longicaudella|BC ZSM Lep 64185|Germany.Bavaria|BOLD:AAK9941
Zimmermannia longicaudella|BC ZSM Lep 70698|Germany.Bavaria|BOLD:AAK9941
Zimmermannia longicaudella|TLMF Lep 02319|Italy.South Tyrol|BOLD:AAK9941
Zimmermannia longicaudella|BC ZSM Lep 70693|Germany.Bavaria|BOLD:AAK9941
Zimmermannia longicaudella|BC ZSM Lep 50967|Germany.Bavaria|BOLD:AAK9941
Zimmermannia longicaudella|BC ZSM Lep 70700|Germany.Bavaria|BOLD:AAK9941
Zimmermannia longicaudella|KLM Lep 00292|Austria.Carinthia|BOLD:AAK9941
Zimmermannia longicaudella|BC ZSM Lep 64188|Germany.Bavaria|BOLD:AAK9941
Zimmermannia longicaudella|BC ZSM Lep 70688|Germany.Bavaria|BOLD:AAK9941
Zimmermannia longicaudella|TLMF Lep 16570|Italy.South Tyrol|BOLD:AAK9941
Zimmermannia longicaudella|BC ZSM Lep 64187|Germany.Bavaria|BOLD:AAK9941
Zimmermannia longicaudella|TLMF Lep 15359|Italy.South Tyrol|BOLD:AAK9941
Zimmermannia longicaudella|TLMF Lep 16573|Italy.South Tyrol|BOLD:AAK9941
Zimmermannia longicaudella|RMNH.INS.24536|Netherlands.Limburg|BOLD:AAK9941
Zimmermannia longicaudella|BC ZSM Lep 70695|Germany.Bavaria|BOLD:AAK9941
Zimmermannia monemvasiae|RMNH.INS.24796|Cyprus.Paphos|BOLD:ADA1969
Zimmermannia sivickisi|RMNH.INS.24485|China.Liaoning|BOLD:ACU5578
Zimmermannia mesoloba|RMNH.INS.24684|United States.Maryland|
Zimmermannia mesoloba|MDOK-1682|United States.Oklahoma|BOLD:AAH0033
Zimmermannia mesoloba|RMNH.INS.24692|United States.Oklahoma|BOLD:AAH0033
Zimmermannia mesoloba|RMNH.INS.24122|United States.West Virginia|BOLD:ACY4932
Zimmermannia mesoloba|RMNH.INS.24121|United States.West Virginia|BOLD:ACY4932
Zimmermannia mesoloba|RMNH.INS.24690|United States.West Virginia|BOLD:ACY4932
Zimmermannia mesoloba|RMNH.INS.24688|United States.West Virginia|BOLD:ACY4932
Zimmermannia ArizonaEvN4691|RMNH.INS.24691|United States.Arizona|BOLD:ABA1175
Zimmermannia BCCASalix|RMNH.INS.24695|Canada.British Columbia|BOLD:ACY4703
Zimmermannia BCCASalix|RMNH.INS.24142|United States.California|
Zimmermannia bosquella|MDOK-2303|United States.Oklahoma|BOLD:AAH5136
Zimmermannia bosquella|RMNH.INS.24694|United States.Oklahoma|BOLD:AAH5136
Zimmermannia bosquella|DNA-ATBI-3421|United States.Tennessee|BOLD:AAH5136
Zimmermannia bosquella|RMNH.INS.24120|United States.West Virginia|BOLD:AAH4556
Zimmermannia bosquella|MDOK-2281|United States.Oklahoma|BOLD:AAH4556
Zimmermannia bosquella|DNA-ATBI-3422|United States.Tennessee|BOLD:AAH4556
Zimmermannia bosquella|RMNH.INS.24693|United States.Oklahoma|BOLD:AAH4556
Zimmermannia bosquella|MDOK-2265|United States.Oklahoma|BOLD:ABY6142
Zimmermannia bosquella|AC005458|Canada.Quebec|BOLD:ABY6142
Zimmermannia bosquella|DNA-ATBI-3011|United States.Tennessee|BOLD:ABY6142
Zimmermannia bosquella|RMNH.INS.24118|United States.West Virginia|BOLD:ABY6142
Zimmermannia bosquella|RMNH.INS.24124|United States.Indiana|BOLD:ABY6142
Zimmermannia bosquella|RMNH.INS.24689|United States.West Virginia|BOLD:ABY6142
Zimmermannia bosquella|RMNH.INS.24123|United States.Michigan|BOLD:ACG8892
Zimmermannia bosquella|DNA-ATBI-3010|United States.Tennessee|BOLD:ACE7124
Zimmermannia bosquella|BMNH.E.1627561|United States.Texas|BOLD:ACE7124
Zimmermannia California|RMNH.INS.24696|United States.California|BOLD:ACK1729
Zimmermannia grandisella|RMNH.INS.24685|United States.Indiana|BOLD:ACU7512
Zimmermannia grandisella|CNCLEP00035996|Canada.Ontario|BOLD:AAN4942
Zimmermannia grandisella|AC006880|Canada.Quebec|BOLD:AAH4721
Zimmermannia grandisella|RMNH.INS.24697|United States.New Hampshire|BOLD:AAH4721
Zimmermannia grandisella|CNCLEP00040440|Canada.Manitoba|BOLD:AAH4721
Zimmermannia grandisella|CNCLEP00040239|Canada.Manitoba|BOLD:AAH4721
Zimmermannia liguricella|RMNH.INS.11631|Spain.Andalusia|BOLD:ACY4706

Zimmermannia grandisella|CNCLEP00040440|Canada.Manitoba|BOLD:AAH4721
Zimmermannia grandisella|CNCLEP00040239|Canada.Manitoba|BOLD:AAH4721
Zimmermannia liguricella|RMNH.INS.11631|Spain.Andalusia|BOLD:ACY4706
Zimmermannia ManitobaMIC7473|CNCLEP00040220|Canada.Manitoba|BOLD:AAI6170
Zimmermannia Nepal|RMNH.INS.24112|Nepal|
Zimmermannia obrutella|DNA-ATBI-3420|United States.Tennessee|BOLD:AAI6167
Zimmermannia obrutella|RMNH.INS.24119|United States.West Virginia|BOLD:AAI6164
Zimmermannia obrutella|DNA-ATBI-3009|United States.Tennessee|BOLD:AAI6164
Zimmermannia obrutella|DNA-ATBI-3263|United States.Tennessee|BOLD:AAX9717
Zimmermannia TaiwanEvN4062|RMNH.INS.24062|Taiwan.Taiwan Province|BOLD:ACY4319
Zimmermannia amani|RMNH.INS.24484|China.Liaoning|BOLD:ACU5524
Zimmermannia amani|RMNH.INS.23678|Croatia|BOLD:AAI9360
Zimmermannia amani|BC ZSM Lep 64220|Germany.Bavaria|BOLD:AAI9360
Zimmermannia amani|RMNH.INS.24548|Croatia|BOLD:AAI9360
Zimmermannia amani|KLM Lep 05989|Romania|BOLD:AAI9360
Zimmermannia amani|RMNH.INS.23943|France.Provence-Alpes-Cote d'Azur|BOLD:AAI9360
Zimmermannia amani|BC ZSM Lep 63862|Germany.Bavaria|BOLD:AAI9360
Zimmermannia amani|RMNH.INS.23820|Germany.Baden-Wuerttemberg|BOLD:AAI9360
Zimmermannia amani|RMNH.INS.24016|China.Heilongjiang Sheng|
Zimmermannia reichli|RMNH.INS.23677|Italy.Piedmont|BOLD:AAX9721
Zimmermannia reichli|TLMF Lep 18470|Italy.South Tyrol|BOLD:AAX9721
Zimmermannia hispanica|RMNH.INS.24805|Morocco.Marrakech-Tensift-El Haouz Region|BOLD:ADA2741
Zimmermannia vivesi|RMNH.INS.23832|Cyprus|BOLD:ACY4629
Ozadelpha EvN4680|RMNH.INS.24680|Costa Rica.Puntarenas|BOLD:ACU6274
Roscidotoga callicomae|RMNH.INS.11834|Australia.Queensland|BOLD:AAZ2495
Roscidotoga callicomae|RMNH.INS.11833|Australia.Queensland|BOLD:AAZ2495
Roscidotoga lamingtonia|RMNH.INS.23735|Australia.Queensland|BOLD:AAZ1940
Roscidotoga lamingtonia|RMNH.INS.11835|Australia.Queensland|BOLD:AAZ1940
Roscidotoga lamingtonia|RMNH.INS.23528|Australia.New South Wales|BOLD:AAZ1940
Stigmella Shorealeprosula|RMNH.INS.11984|Indonesia.Kalimantan Timur|BOLD:ACG9083
Glaucolepis saccharella|RMNH.INS.18308|United States.Tennessee|BOLD:AAH4494
Glaucolepis saccharella|RMNH.INS.18648|United States.Connecticut|BOLD:AAH4494
Glaucolepis saccharella|RMNH.INS.18186|United States.North Carolina|BOLD:AAH4494
Glaucolepis saccharella|RMNH.INS.18401|United States.Georgia|BOLD:AAH4494
Glaucolepis saccharella|RMNH.INS.24404|United States.Georgia|BOLD:AAH4494
Glaucolepis saccharella|RMNH.INS.18402|United States.Georgia|BOLD:AAH4494
Glaucolepis saccharella|RMNH.INS.24405|United States.Vermont|BOLD:AAH4494
Stigmella ShoreapaucifloraEvN4149|RMNH.INS.24150|Indonesia.Kalimantan Timur|BOLD:ACG9428
Stigmella ShoreapaucifloraEvN4149|RMNH.INS.24149|Indonesia.Kalimantan Timur|BOLD:ACG9428
Stigmella ShoreapaucifloraEvN4149|RMNH.INS.11947|Indonesia.Kalimantan Timur|BOLD:ACG9428
Stigmella ShoreapaucifloraEvN4151|RMNH.INS.11991|Indonesia.Kalimantan Timur|BOLD:ACG9487
Stigmella ShoreapaucifloraEvN4151|RMNH.INS.24151|Indonesia.Kalimantan Timur|BOLD:ACG9487
Menurella acmenae|RMNH.INS.23541|Australia.New South Wales|BOLD:ACB1838
Menurella scotodes|RMNH.INS.23548|Australia.Queensland|BOLD:ACB1839
Menurella tribulatrix|RMNH.INS.23963|Australia.Queensland|BOLD:ACB1889
Menurella tribulatrix|RMNH.INS.23962|Australia.Queensland|BOLD:ACB1889
Menurella Eucalyptusjuv|RMNH.INS.24081|Australia.Australian Capital Territory|BOLD:ACB2117
Menurella MtNelsonfasciate|RMNH.INS.24102|Australia.Tasmania|BOLD:ACB1823
Menurella MtNelsonfasciate|RMNH.INS.24103|Australia.Tasmania|BOLD:ACB1823
Menurella MtNelsonfasciate|RMNH.INS.24100|Australia.Tasmania|BOLD:ACB1823
Pectinivalva mystaconota|RMNH.INS.24106|Australia.Tasmania|BOLD:ACB2002
Pectinivalva caenodora|RMNH.INS.24108|Australia.New South Wales|BOLD:ACB2003
Pectinivalva caenodoragroup|RMNH.INS.23540|Australia.Queensland|BOLD:ACB2001
Pectinivalva MtNelsondark|RMNH.INS.24104|Australia.Tasmania|
Pectinivalva MtNelsondark|RMNH.INS.24105|Australia.Tasmania|BOLD:ACB2113
Pectinivalva NSWdark|RMNH.INS.24107|Australia.New South Wales|BOLD:ACB2116
Pectinivalva Canberralinear|RMNH.INS.11846|Australia.Australian Capital Territory|BOLD:ADA1689
Pectinivalva RH148Eucalyptus|RMNH.INS.24083|Australia.Australian Capital Territory|BOLD:ACB2114
Pectinivalva sp. ANIC6|RMNH.INS.24082|Australia.Australian Capital Territory|BOLD:ABX1483
Bohemannia auriciliella|RMNH.INS.23932|Bulgaria.Burgas|BOLD:ACG8823
Bohemannia auriciliella|RMNH.INS.23747|Netherlands.Gelderland|BOLD:ACG8823
Stigmella angustivalva|RMNH.INS.24073|South Africa.Northern Cape|BOLD:ACY4832
Trifurcula euremaEvN3564|RMNH.INS.23564|Ukraine|BOLD:ACG9245
Trifurcula ItalyEvN3946|RMNH.INS.23946|Italy.Piedmont|BOLD:ACG9116
Trifurcula cryptella|RMNH.INS.17652|France.Provence-Alpes-Cote d'Azur|BOLD:ACG9391
Trifurcula cryptella|RMNH.INS.23944|France.Provence-Alpes-Cote d'Azur|BOLD:ACG9391
Trifurcula cryptella|RMNH.INS.17653|France.Provence-Alpes-Cote d'Azur|BOLD:ACG9391
Trifurcula cryptella|RMNH.INS.24561|Switzerland.Graubunden|BOLD:ACG9391
Trifurcula cryptella|BC ZSM Lep 100126|Germany.Bavaria|BOLD:ACG9391
Trifurcula cryptella|TLMF Lep 16582|Italy.South Tyrol|BOLD:ACG9391
Trifurcula cryptella|TLMF Lep 16522|Italy.South Tyrol|BOLD:ACG9391
Trifurcula cryptella|RMNH.INS.24305|Bulgaria.Blagoevgrad|BOLD:ACG9391
Trifurcula eurema|TLMF Lep 15294|Austria.Tirol|BOLD:ACR4608
Trifurcula eurema|RMNH.INS.24226|Croatia|BOLD:ACG8808
Trifurcula eurema|RMNH.INS.23517|Croatia|BOLD:ACG8808
Trifurcula eurema|TLMF Lep 18070|Austria.Tirol|BOLD:ACG8808
Trifurcula eurema|RMNH.INS.24245|Malta|BOLD:ACG8808
Trifurcula eurema|RMNH.INS.24010|Malta|BOLD:ACG8808
Trifurcula eurema|RMNH.INS.11717|Netherlands.South Holland|BOLD:ACG8808
Trifurcula eurema|RMNH.INS.11719|Netherlands.South Holland|BOLD:ACG8808
Trifurcula eurema|RMNH.INS.11718|Netherlands.South Holland|BOLD:ACG8808
Trifurcula eurema|BC ZSM Lep 89145|Germany.Bavaria|BOLD:ACG8808
Trifurcula manygoza|RMNH.INS.24552|Italy.Friuli-Venezia Giulia|BOLD:ACU5606
Trifurcula manygoza|RMNH.INS.24319|Greece|
Trifurcula manygoza|RMNH.INS.23718|Greece|BOLD:ACG8887
Trifurcula manygoza|RMNH.INS.23717|Greece|BOLD:ACG8887
Trifurcula anthyllidella|RMNH.INS.11501|Spain.Andalusia|BOLD:ACG8863
Trifurcula anthyllidella|RMNH.INS.11502|Spain.Andalusia|BOLD:ACG8863
Trifurcula ortneri|RMNH.INS.11503|Spain.Comunidad de Madrid|BOLD:ACG8999
Trifurcula ortneri|RMNH.INS.23668|France.Languedoc-Roussillon|BOLD:ACG8999
Trifurcula ortneri|KLM Lep 02905|Austria|BOLD:ACG8999
Trifurcula ortneri|KLM Lep 01247|Austria.Carinthia|BOLD:ACG8999
Trifurcula ridiculosa|RMNH.INS.12550|Spain.Canary Islands|BOLD:ACG8769
Trifurcula calycotomella|RMNH.INS.24033|Italy.Sardinia|BOLD:ACY3801
Trifurcula calycotomella|RMNH.INS.24612|Italy.Calabria|BOLD:ACU5680
Trifurcula serotinella|RMNH.INS.24227|Germany.Rhineland-Palatinate|BOLD:ACY4397
Trifurcula serotinella|BC ZSM Lep 79394|Germany.Bavaria|BOLD:ACG9007
Trifurcula serotinella|BC ZSM Lep 76452|Germany.Bavaria|BOLD:ACG9007
Trifurcula serotinella|BC ZSM Lep 76456|Germany.Bavaria|BOLD:ACG9007
Trifurcula serotinella|RMNH.INS.11309|Germany.Rhineland-Palatinate|BOLD:ACG9007
Trifurcula SpainEvN3958|RMNH.INS.23958|Spain.Aragon|BOLD:ACY4123

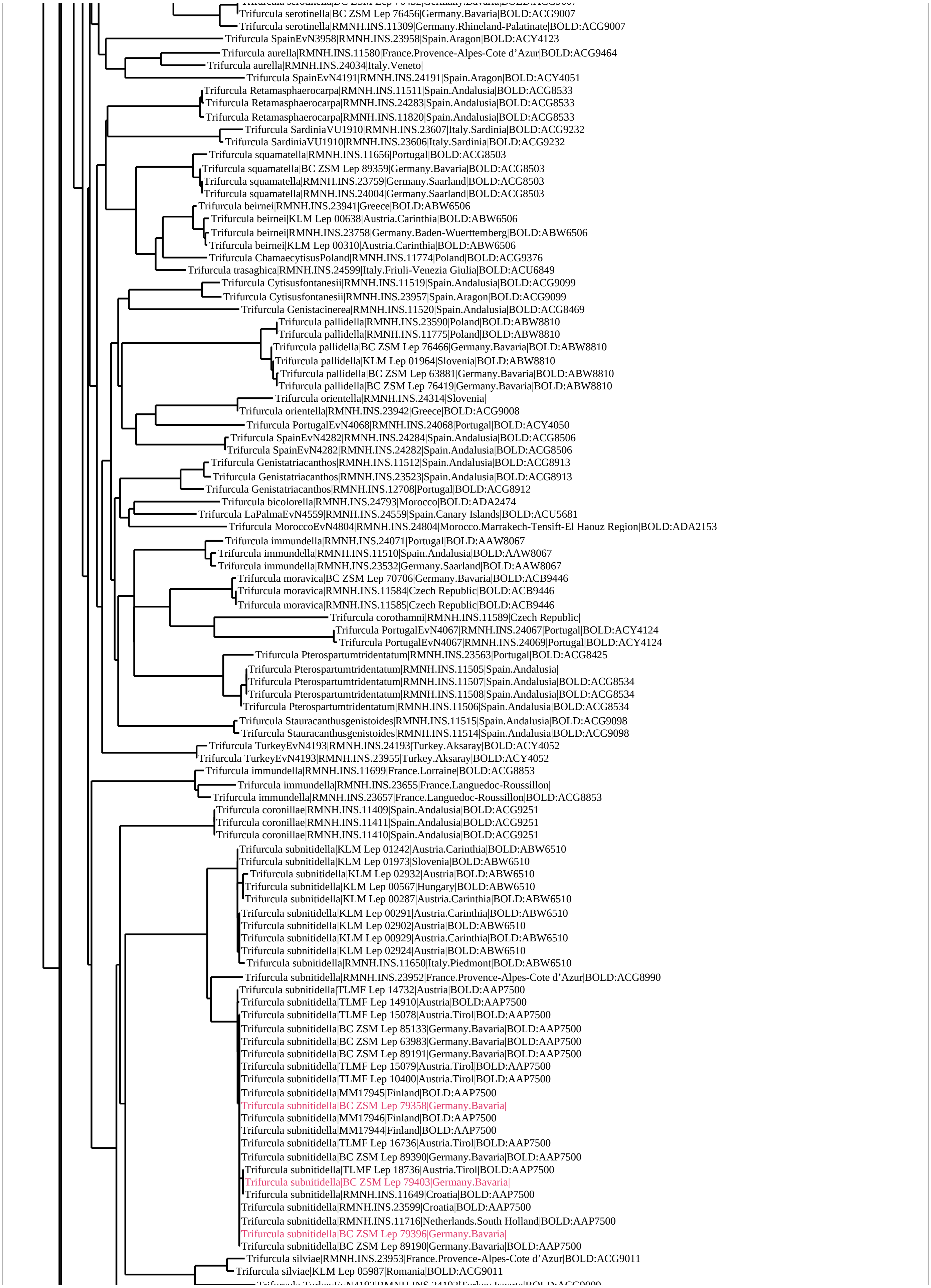
Trifurcula serotinella|BC ZSM Lep 76456|Germany.Bavaria|BOLD:ACG9007
Trifurcula serotinella|RMNH.INS.11309|Germany.Rhineland-Palatinate|BOLD:ACG9007
Trifurcula SpainEvN3958|RMNH.INS.23958|Spain.Aragon|BOLD:ACY4123
Trifurcula aurella|RMNH.INS.11580|France.Provence-Alpes-Cote d'Azur|BOLD:ACG9464
Trifurcula aurella|RMNH.INS.24034|Italy.Veneto|
Trifurcula SpainEvN4191|RMNH.INS.24191|Spain.Aragon|BOLD:ACY4051
Trifurcula Retamasphaerocarpa|RMNH.INS.11511|Spain.Andalusia|BOLD:ACG8533
Trifurcula Retamasphaerocarpa|RMNH.INS.24283|Spain.Andalusia|BOLD:ACG8533
Trifurcula Retamasphaerocarpa|RMNH.INS.11820|Spain.Andalusia|BOLD:ACG8533
Trifurcula SardiniaVU1910|RMNH.INS.23607|Italy.Sardinia|BOLD:ACG9232
Trifurcula SardiniaVU1910|RMNH.INS.23606|Italy.Sardinia|BOLD:ACG9232
Trifurcula squamatella|RMNH.INS.11656|Portugal|BOLD:ACG8503
Trifurcula squamatella|BC ZSM Lep 89359|Germany.Bavaria|BOLD:ACG8503
Trifurcula squamatella|RMNH.INS.23759|Germany.Saarland|BOLD:ACG8503
Trifurcula squamatella|RMNH.INS.24004|Germany.Saarland|BOLD:ACG8503
Trifurcula beirnei|RMNH.INS.23941|Greece|BOLD:ABW6506
Trifurcula beirnei|KLM Lep 00638|Austria.Carinthia|BOLD:ABW6506
Trifurcula beirnei|RMNH.INS.23758|Germany.Baden-Wuerttemberg|BOLD:ABW6506
Trifurcula beirnei|KLM Lep 00310|Austria.Carinthia|BOLD:ABW6506
Trifurcula ChamaecytisusPoland|RMNH.INS.11774|Poland|BOLD:ACG9376
Trifurcula trasaghica|RMNH.INS.24599|Italy.Friuli-Venezia Giulia|BOLD:ACU6849
Trifurcula Cytisusfontanesii|RMNH.INS.11519|Spain.Andalusia|BOLD:ACG9099
Trifurcula Cytisusfontanesii|RMNH.INS.23957|Spain.Aragon|BOLD:ACG9099
Trifurcula Genistacinerea|RMNH.INS.11520|Spain.Andalusia|BOLD:ACG8469
Trifurcula pallidella|RMNH.INS.23590|Poland|BOLD:ABW8810
Trifurcula pallidella|RMNH.INS.11775|Poland|BOLD:ABW8810
Trifurcula pallidella|BC ZSM Lep 76466|Germany.Bavaria|BOLD:ABW8810
Trifurcula pallidella|KLM Lep 01964|Slovenia|BOLD:ABW8810
Trifurcula pallidella|BC ZSM Lep 63881|Germany.Bavaria|BOLD:ABW8810
Trifurcula pallidella|BC ZSM Lep 76419|Germany.Bavaria|BOLD:ABW8810
Trifurcula orientella|RMNH.INS.24314|Slovenia|
Trifurcula orientella|RMNH.INS.23942|Greece|BOLD:ACG9008
Trifurcula PortugalEvN4068|RMNH.INS.24068|Portugal|BOLD:ACY4050
Trifurcula SpainEvN4282|RMNH.INS.24284|Spain.Andalusia|BOLD:ACG8506
Trifurcula SpainEvN4282|RMNH.INS.24282|Spain.Andalusia|BOLD:ACG8506
Trifurcula Genistatriacanthos|RMNH.INS.11512|Spain.Andalusia|BOLD:ACG8913
Trifurcula Genistatriacanthos|RMNH.INS.23523|Spain.Andalusia|BOLD:ACG8913
Trifurcula Genistatriacanthos|RMNH.INS.12708|Portugal|BOLD:ACG8912
Trifurcula bicolorella|RMNH.INS.24793|Morocco|BOLD:ADA2474
Trifurcula LaPalmaEvN4559|RMNH.INS.24559|Spain.Canary Islands|BOLD:ACU5681
Trifurcula MoroccoEvN4804|RMNH.INS.24804|Morocco.Marrakech-Tensift-El Haouz Region|BOLD:ADA2153
Trifurcula immundella|RMNH.INS.24071|Portugal|BOLD:AAW8067
Trifurcula immundella|RMNH.INS.11510|Spain.Andalusia|BOLD:AAW8067
Trifurcula immundella|RMNH.INS.23532|Germany.Saarland|BOLD:AAW8067
Trifurcula moravica|BC ZSM Lep 70706|Germany.Bavaria|BOLD:ACB9446
Trifurcula moravica|RMNH.INS.11584|Czech Republic|BOLD:ACB9446
Trifurcula moravica|RMNH.INS.11585|Czech Republic|BOLD:ACB9446
Trifurcula corothamni|RMNH.INS.11589|Czech Republic|
Trifurcula PortugalEvN4067|RMNH.INS.24067|Portugal|BOLD:ACY4124
Trifurcula PortugalEvN4067|RMNH.INS.24069|Portugal|BOLD:ACY4124
Trifurcula Pterospartumtridentatum|RMNH.INS.23563|Portugal|BOLD:ACG8425
Trifurcula Pterospartumtridentatum|RMNH.INS.11505|Spain.Andalusia|
Trifurcula Pterospartumtridentatum|RMNH.INS.11507|Spain.Andalusia|BOLD:ACG8534
Trifurcula Pterospartumtridentatum|RMNH.INS.11508|Spain.Andalusia|BOLD:ACG8534
Trifurcula Pterospartumtridentatum|RMNH.INS.11506|Spain.Andalusia|BOLD:ACG8534
Trifurcula Stauracanthusgenistoides|RMNH.INS.11515|Spain.Andalusia|BOLD:ACG9098
Trifurcula Stauracanthusgenistoides|RMNH.INS.11514|Spain.Andalusia|BOLD:ACG9098
Trifurcula TurkeyEvN4193|RMNH.INS.24193|Turkey.Aksaray|BOLD:ACY4052
Trifurcula TurkeyEvN4193|RMNH.INS.23955|Turkey.Aksaray|BOLD:ACY4052
Trifurcula immundella|RMNH.INS.11699|France.Lorraine|BOLD:ACG8853
Trifurcula immundella|RMNH.INS.23655|France.Languedoc-Roussillon|
Trifurcula immundella|RMNH.INS.23657|France.Languedoc-Roussillon|BOLD:ACG8853
Trifurcula coronillae|RMNH.INS.11409|Spain.Andalusia|BOLD:ACG9251
Trifurcula coronillae|RMNH.INS.11411|Spain.Andalusia|BOLD:ACG9251
Trifurcula coronillae|RMNH.INS.11410|Spain.Andalusia|BOLD:ACG9251
Trifurcula subnitidella|KLM Lep 01242|Austria.Carinthia|BOLD:ABW6510
Trifurcula subnitidella|KLM Lep 01973|Slovenia|BOLD:ABW6510
Trifurcula subnitidella|KLM Lep 02932|Austria|BOLD:ABW6510
Trifurcula subnitidella|KLM Lep 00567|Hungary|BOLD:ABW6510
Trifurcula subnitidella|KLM Lep 00287|Austria.Carinthia|BOLD:ABW6510
Trifurcula subnitidella|KLM Lep 00291|Austria.Carinthia|BOLD:ABW6510
Trifurcula subnitidella|KLM Lep 02902|Austria|BOLD:ABW6510
Trifurcula subnitidella|KLM Lep 00929|Austria.Carinthia|BOLD:ABW6510
Trifurcula subnitidella|KLM Lep 02924|Austria|BOLD:ABW6510
Trifurcula subnitidella|RMNH.INS.11650|Italy.Piedmont|BOLD:ABW6510
Trifurcula subnitidella|RMNH.INS.23952|France.Provence-Alpes-Cote d'Azur|BOLD:ACG8990
Trifurcula subnitidella|TLMF Lep 14732|Austria|BOLD:AAP7500
Trifurcula subnitidella|TLMF Lep 14910|Austria|BOLD:AAP7500
Trifurcula subnitidella|TLMF Lep 15078|Austria.Tirol|BOLD:AAP7500
Trifurcula subnitidella|BC ZSM Lep 85133|Germany.Bavaria|BOLD:AAP7500
Trifurcula subnitidella|BC ZSM Lep 63983|Germany.Bavaria|BOLD:AAP7500
Trifurcula subnitidella|BC ZSM Lep 89191|Germany.Bavaria|BOLD:AAP7500
Trifurcula subnitidella|TLMF Lep 15079|Austria.Tirol|BOLD:AAP7500
Trifurcula subnitidella|TLMF Lep 10400|Austria.Tirol|BOLD:AAP7500
Trifurcula subnitidella|MM17945|Finland|BOLD:AAP7500
Trifurcula subnitidella|BC ZSM Lep 79358|Germany.Bavaria|
Trifurcula subnitidella|MM17946|Finland|BOLD:AAP7500
Trifurcula subnitidella|MM17944|Finland|BOLD:AAP7500
Trifurcula subnitidella|TLMF Lep 16736|Austria.Tirol|BOLD:AAP7500
Trifurcula subnitidella|BC ZSM Lep 89390|Germany.Bavaria|BOLD:AAP7500
Trifurcula subnitidella|TLMF Lep 18736|Austria.Tirol|BOLD:AAP7500
Trifurcula subnitidella|BC ZSM Lep 79403|Germany.Bavaria|
Trifurcula subnitidella|RMNH.INS.11649|Croatia|BOLD:AAP7500
Trifurcula subnitidella|RMNH.INS.23599|Croatia|BOLD:AAP7500
Trifurcula subnitidella|RMNH.INS.11716|Netherlands.South Holland|BOLD:AAP7500
Trifurcula subnitidella|BC ZSM Lep 79396|Germany.Bavaria|
Trifurcula subnitidella|BC ZSM Lep 89190|Germany.Bavaria|BOLD:AAP7500
Trifurcula silviae|RMNH.INS.23953|France.Provence-Alpes-Cote d'Azur|BOLD:ACG9011
Trifurcula silviae|KLM Lep 05987|Romania|BOLD:ACG9011

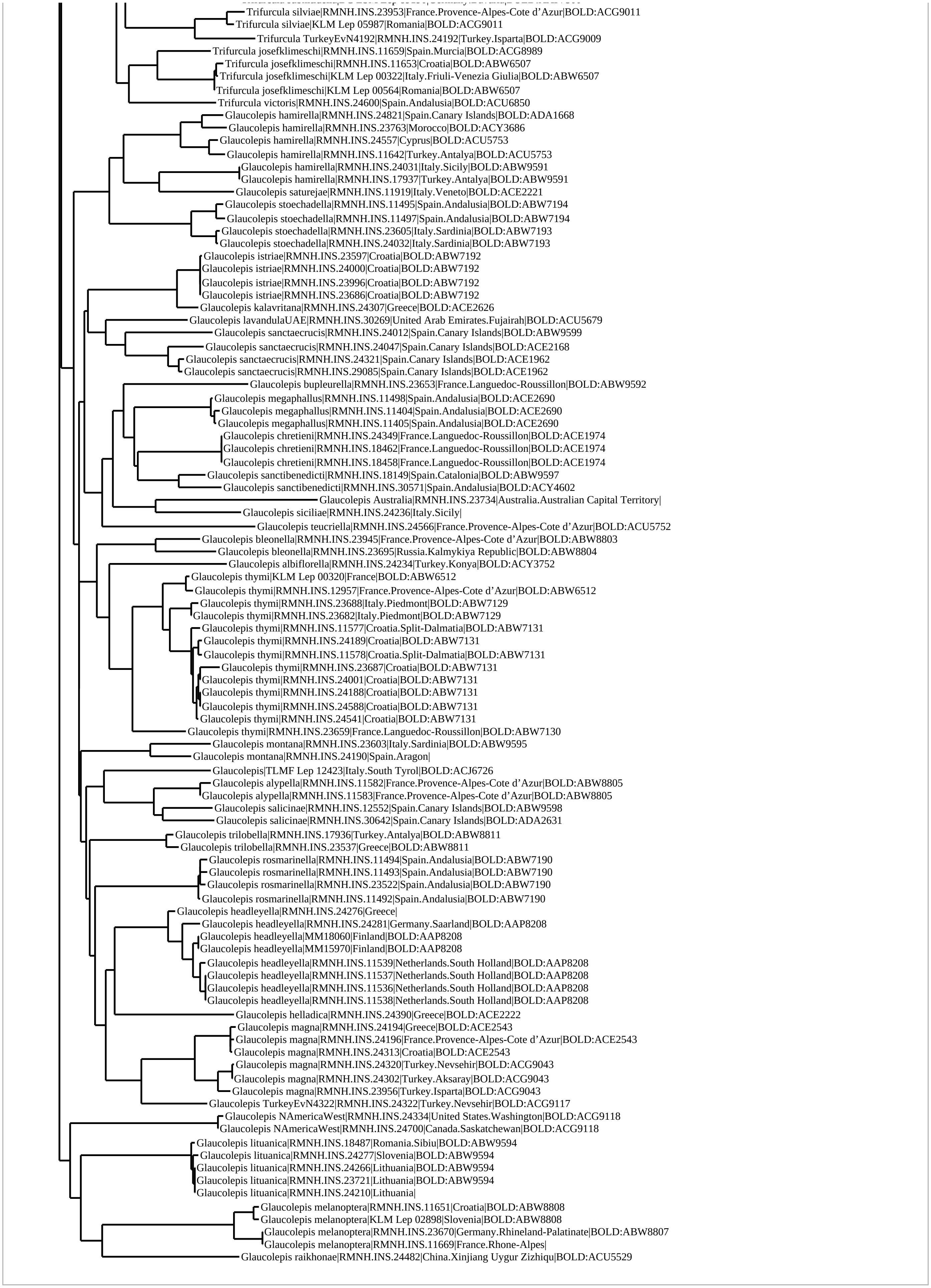
Trifurcula silviae|RMNH.INS.23953|France.Provence-Alpes-Cote d'Azur|BOLD:ACG9011
Trifurcula silviae|KLM Lep 05987|Romania|BOLD:ACG9011
Trifurcula TurkeyEvN4192|RMNH.INS.24192|Turkey.Isparta|BOLD:ACG9009
Trifurcula josefklimeschi|RMNH.INS.11659|Spain.Murcia|BOLD:ACG8989
Trifurcula josefklimeschi|RMNH.INS.11653|Croatia|BOLD:ABW6507
Trifurcula josefklimeschi|KLM Lep 00322|Italy.Friuli-Venezia Giulia|BOLD:ABW6507
Trifurcula josefklimeschi|KLM Lep 00564|Romania|BOLD:ABW6507
Trifurcula victoris|RMNH.INS.24600|Spain.Andalusia|BOLD:ACU6850
Glaucolepis hamirella|RMNH.INS.24821|Spain.Canary Islands|BOLD:ADA1668
Glaucolepis hamirella|RMNH.INS.23763|Morocco|BOLD:ACY3686
Glaucolepis hamirella|RMNH.INS.24557|Cyprus|BOLD:ACU5753
Glaucolepis hamirella|RMNH.INS.11642|Turkey.Antalya|BOLD:ACU5753
Glaucolepis hamirella|RMNH.INS.24031|Italy.Sicily|BOLD:ABW9591
Glaucolepis hamirella|RMNH.INS.17937|Turkey.Antalya|BOLD:ABW9591
Glaucolepis saturejae|RMNH.INS.11919|Italy.Veneto|BOLD:ACE2221
Glaucolepis stoechadella|RMNH.INS.11495|Spain.Andalusia|BOLD:ABW7194
Glaucolepis stoechadella|RMNH.INS.11497|Spain.Andalusia|BOLD:ABW7194
Glaucolepis stoechadella|RMNH.INS.23605|Italy.Sardinia|BOLD:ABW7193
Glaucolepis stoechadella|RMNH.INS.24032|Italy.Sardinia|BOLD:ABW7193
Glaucolepis istriae|RMNH.INS.23597|Croatia|BOLD:ABW7192
Glaucolepis istriae|RMNH.INS.24000|Croatia|BOLD:ABW7192
Glaucolepis istriae|RMNH.INS.23996|Croatia|BOLD:ABW7192
Glaucolepis istriae|RMNH.INS.23686|Croatia|BOLD:ABW7192
Glaucolepis kalavritana|RMNH.INS.24307|Greece|BOLD:ACE2626
Glaucolepis lavandulaUAE|RMNH.INS.30269|United Arab Emirates.Fujairah|BOLD:ACU5679
Glaucolepis sanctaecrucis|RMNH.INS.24012|Spain.Canary Islands|BOLD:ABW9599
Glaucolepis sanctaecrucis|RMNH.INS.24047|Spain.Canary Islands|BOLD:ACE2168
Glaucolepis sanctaecrucis|RMNH.INS.24321|Spain.Canary Islands|BOLD:ACE1962
Glaucolepis sanctaecrucis|RMNH.INS.29085|Spain.Canary Islands|BOLD:ACE1962
Glaucolepis bupleurella|RMNH.INS.23653|France.Languedoc-Roussillon|BOLD:ABW9592
Glaucolepis megaphallus|RMNH.INS.11498|Spain.Andalusia|BOLD:ACE2690
Glaucolepis megaphallus|RMNH.INS.11404|Spain.Andalusia|BOLD:ACE2690
Glaucolepis megaphallus|RMNH.INS.11405|Spain.Andalusia|BOLD:ACE2690
Glaucolepis chretieni|RMNH.INS.24349|France.Languedoc-Roussillon|BOLD:ACE1974
Glaucolepis chretieni|RMNH.INS.18462|France.Languedoc-Roussillon|BOLD:ACE1974
Glaucolepis chretieni|RMNH.INS.18458|France.Languedoc-Roussillon|BOLD:ACE1974
Glaucolepis sanctibenedicti|RMNH.INS.18149|Spain.Catalonia|BOLD:ABW9597
Glaucolepis sanctibenedicti|RMNH.INS.30571|Spain.Andalusia|BOLD:ACY4602
Glaucolepis Australia|RMNH.INS.23734|Australia.Australian Capital Territory|
Glaucolepis siciliae|RMNH.INS.24236|Italy.Sicily|
Glaucolepis teucriella|RMNH.INS.24566|France.Provence-Alpes-Cote d'Azur|BOLD:ACU5752
Glaucolepis bleonella|RMNH.INS.23945|France.Provence-Alpes-Cote d'Azur|BOLD:ABW8803
Glaucolepis bleonella|RMNH.INS.23695|Russia.Kalmykiya Republic|BOLD:ABW8804
Glaucolepis albiflorella|RMNH.INS.24234|Turkey.Konya|BOLD:ACY3752
Glaucolepis thymi|KLM Lep 00320|France|BOLD:ABW6512
Glaucolepis thymi|RMNH.INS.12957|France.Provence-Alpes-Cote d'Azur|BOLD:ABW6512
Glaucolepis thymi|RMNH.INS.23688|Italy.Piedmont|BOLD:ABW7129
Glaucolepis thymi|RMNH.INS.23682|Italy.Piedmont|BOLD:ABW7129
Glaucolepis thymi|RMNH.INS.11577|Croatia.Split-Dalmatia|BOLD:ABW7131
Glaucolepis thymi|RMNH.INS.24189|Croatia|BOLD:ABW7131
Glaucolepis thymi|RMNH.INS.11578|Croatia.Split-Dalmatia|BOLD:ABW7131
Glaucolepis thymi|RMNH.INS.23687|Croatia|BOLD:ABW7131
Glaucolepis thymi|RMNH.INS.24001|Croatia|BOLD:ABW7131
Glaucolepis thymi|RMNH.INS.24188|Croatia|BOLD:ABW7131
Glaucolepis thymi|RMNH.INS.24588|Croatia|BOLD:ABW7131
Glaucolepis thymi|RMNH.INS.24541|Croatia|BOLD:ABW7131
Glaucolepis thymi|RMNH.INS.23659|France.Languedoc-Roussillon|BOLD:ABW7130
Glaucolepis montana|RMNH.INS.23603|Italy.Sardinia|BOLD:ABW9595
Glaucolepis montana|RMNH.INS.24190|Spain.Aragon|
Glaucolepis|TLMF Lep 12423|Italy.South Tyrol|BOLD:ACJ6726
Glaucolepis alypella|RMNH.INS.11582|France.Provence-Alpes-Cote d'Azur|BOLD:ABW8805
Glaucolepis alypella|RMNH.INS.11583|France.Provence-Alpes-Cote d'Azur|BOLD:ABW8805
Glaucolepis salicinae|RMNH.INS.12552|Spain.Canary Islands|BOLD:ABW9598
Glaucolepis salicinae|RMNH.INS.30642|Spain.Canary Islands|BOLD:ADA2631
Glaucolepis trilobella|RMNH.INS.17936|Turkey.Antalya|BOLD:ABW8811
Glaucolepis trilobella|RMNH.INS.23537|Greece|BOLD:ABW8811
Glaucolepis rosmarinella|RMNH.INS.11494|Spain.Andalusia|BOLD:ABW7190
Glaucolepis rosmarinella|RMNH.INS.11493|Spain.Andalusia|BOLD:ABW7190
Glaucolepis rosmarinella|RMNH.INS.23522|Spain.Andalusia|BOLD:ABW7190
Glaucolepis rosmarinella|RMNH.INS.11492|Spain.Andalusia|BOLD:ABW7190
Glaucolepis headleyella|RMNH.INS.24276|Greece|
Glaucolepis headleyella|RMNH.INS.24281|Germany.Saarland|BOLD:AAP8208
Glaucolepis headleyella|MM18060|Finland|BOLD:AAP8208
Glaucolepis headleyella|MM15970|Finland|BOLD:AAP8208
Glaucolepis headleyella|RMNH.INS.11539|Netherlands.South Holland|BOLD:AAP8208
Glaucolepis headleyella|RMNH.INS.11537|Netherlands.South Holland|BOLD:AAP8208
Glaucolepis headleyella|RMNH.INS.11536|Netherlands.South Holland|BOLD:AAP8208
Glaucolepis headleyella|RMNH.INS.11538|Netherlands.South Holland|BOLD:AAP8208
Glaucolepis helladica|RMNH.INS.24390|Greece|BOLD:ACE2222
Glaucolepis magna|RMNH.INS.24194|Greece|BOLD:ACE2543
Glaucolepis magna|RMNH.INS.24196|France.Provence-Alpes-Cote d'Azur|BOLD:ACE2543
Glaucolepis magna|RMNH.INS.24313|Croatia|BOLD:ACE2543
Glaucolepis magna|RMNH.INS.24320|Turkey.Nevsehir|BOLD:ACG9043
Glaucolepis magna|RMNH.INS.24302|Turkey.Aksaray|BOLD:ACG9043
Glaucolepis magna|RMNH.INS.23956|Turkey.Isparta|BOLD:ACG9043
Glaucolepis TurkeyEvN4322|RMNH.INS.24322|Turkey.Nevsehir|BOLD:ACG9117
Glaucolepis NAmericaWest|RMNH.INS.24334|United States.Washington|BOLD:ACG9118
Glaucolepis NAmericaWest|RMNH.INS.24700|Canada.Saskatchewan|BOLD:ACG9118
Glaucolepis lituanica|RMNH.INS.18487|Romania.Sibiu|BOLD:ABW9594
Glaucolepis lituanica|RMNH.INS.24277|Slovenia|BOLD:ABW9594
Glaucolepis lituanica|RMNH.INS.24266|Lithuania|BOLD:ABW9594
Glaucolepis lituanica|RMNH.INS.23721|Lithuania|BOLD:ABW9594
Glaucolepis lituanica|RMNH.INS.24210|Lithuania|
Glaucolepis melanoptera|RMNH.INS.11651|Croatia|BOLD:ABW8808
Glaucolepis melanoptera|KLM Lep 02898|Slovenia|BOLD:ABW8808
Glaucolepis melanoptera|RMNH.INS.23670|Germany.Rhineland-Palatinate|BOLD:ABW8807
Glaucolepis melanoptera|RMNH.INS.11669|France.Rhone-Alpes|
Glaucolepis raikhonae|RMNH.INS.24482|China.Xinjiang Uygur Zizhiqu|BOLD:ACU5529
